# Pleiotropic associations of heterozygosity for the *SERPINA1* Z allele in the UK Biobank

**DOI:** 10.1183/23120541.00049-2021

**Published:** 2021-05-10

**Authors:** Katherine A. Fawcett, Kijoung Song, Guoqing Qian, Aliki-Eleni Farmaki, Richard Packer, Catherine John, Nick Shrine, Raquel Granell, Sue Ring, Nicholas J. Timpson, Laura M. Yerges-Armstrong, Richard Eastell, Louise V. Wain, Robert A. Scott, Martin D. Tobin, Ian P. Hall

**Affiliations:** 1 Dept of Health Sciences, University of Leicester, Leicester, UK; 2 Human Genetics, GlaxoSmithKline, Collegeville, PA, USA; 3 Dept of General Internal Medicine, Ningbo First Hospital, Ningbo City, Zhejiang Province, China; 4 Division of Respiratory Medicine, University of Nottingham, and NIHR Nottingham BRC, NUH NHS Trust, Nottingham, UK; 5 MRC Unit for Lifelong Health and Ageing, Institute of Cardiovascular Science, University College London, London, UK; 6 Medical Research Council Integrative Epidemiology Unit, Population Health Sciences, Bristol Medical School, University of Bristol, Bristol, UK; 7 Population Health Sciences, Bristol Medical School, University of Bristol, Bristol, UK; 8 Dept of Oncology and Metabolism, University of Sheffield, Sheffield, UK; 9 National Institute for Health Research, Leicester Respiratory Biomedical Research Centre, Glenfield Hospital, Leicester, UK; 10 Human Genetics – R&D, GSK Medicines Research Centre, Stevenage, UK; 11 These authors contributed equally

## Abstract

Homozygosity for the *SERPINA1* Z allele causes α_1_-antitrypsin deficiency, a rare condition that can cause lung and liver disease. However, the effects of Z allele heterozygosity on nonrespiratory phenotypes, and on lung function in the general population, remain unclear.

We conducted a large, population-based study to determine Z allele effects on >2400 phenotypes in the UK Biobank (N=303 353).

Z allele heterozygosity was strongly associated with increased height (β=1.02 cm, p=3.91×10^−68^), and with other nonrespiratory phenotypes including increased risk of gall bladder disease, reduced risk of heart disease and lower blood pressure, reduced risk of osteoarthritis and reduced bone mineral density, increased risk of headache and enlarged prostate, as well as with blood biomarkers of liver function. Heterozygosity was associated with higher height-adjusted forced expiratory volume in 1 s (FEV_1_) (β=19.36 mL, p=9.21×10^−4^) and FEV_1_/forced vital capacity (β=0.0031, p=1.22×10^−5^) in nonsmokers, whereas in smokers, this protective effect was abolished. Furthermore, we show for the first time that sex modifies the association of the Z allele on lung function.

We conclude that Z allele heterozygosity and homozygosity exhibit opposing effects on lung function in the UK population, and that these associations are modified by smoking and sex. In exploratory analyses, heterozygosity for the Z allele also showed pleiotropic associations with nonrespiratory health-related traits and disease risk.

## Abstract 2

**Heterozygosity for the *SERPINA1* Z allele is associated with higher lung function in nonsmokers (but this advantage is abolished by smoking) as well as a variety of nonrespiratory diseases and traits, including liver-, heart- and bone-related conditions** https://bit.ly/37XC0Yg

## Introduction

Homozygosity for the *SERPINA1* Z allele (rs28929474(T)) is the commonest cause of severe α_1_-antitrypsin deficiency (AATD) and is a well-established genetic risk factor for lung diseases such as chronic obstructive pulmonary disease (COPD). However, the health consequences of heterozygosity for the Z allele are not as well-understood [1]. Given that approximately one in 30 Europeans is heterozygous for the Z allele, the phenotypic consequences of carriage of this allele could have important public health implications.

Some previous studies have sought to characterise the effect of Z allele heterozygosity on nonrespiratory traits, particularly liver diseases [2–6]. However, these have often been carried out in small sample sizes and/or clinical subgroups. The recent development of phenome-wide association studies (PheWASs) and the availability of the well-phenotyped UK Biobank population-based cohort provides a platform for systematic investigation of the effects of heterozygosity for the Z allele on nonrespiratory traits. PheWASs test the association between genetic variants and a large number of phenotypic traits, including diseases and their subtypes, and potential intermediate phenotypes [7]. This differs from genome-wide association studies, which test a large number of variants across the genome for association with only one trait.

The effect of Z allele heterozygosity on lung function traits and lung disease has been the subject of many studies. Recent COPD case–control and family-based studies have shown reduced lung function and increased risk of COPD in heterozygous current and former smokers [8–11]. However, a population-based study demonstrated no significant reductions in lung function in heterozygous smokers, despite having greater numbers of heterozygous smokers compared to previous studies [12]. It also showed enhanced lung function in heterozygous individuals overall, partially explained by strong association of the Z allele with increased height. This discrepancy may be due, in part, to the fact that identifying and recruiting study participants based on their health status (as in case–control studies [8, 9]) or based on the health status of a family member [10, 11] can lead to causal estimates that are subject to ascertainment bias [13], whereas population-based studies [12, 14] overcome these biases. The effects of the Z allele on lung function in relation to smoking status therefore remain uncertain.

Finally, despite evidence for sex-differential effects of Z allele homozygosity on lung function [15, 16], we are not aware of any studies that have compared the effect of Z allele heterozygosity on lung function in males *versus* females.

We therefore systematically evaluated the effects of Z allele heterozygosity in the UK biobank population, which in total includes >18 000 Z allele heterozygotes. We aimed: 1) to undertake the most extensive PheWAS to date, including blood biomarkers, for Z allele heterozygosity and homozygosity to identify effects beyond the respiratory system; and 2) to fully define the effects of Z allele heterozygosity on lung function measures (forced expiratory volume in 1 s (FEV_1_) and forced vital capacity (FVC)) in smokers and nonsmokers and in males and females.

## Methods

### Cohorts

#### Ethics statement

This study used anonymised data from UK Biobank, which has ethical approval from the UK National Health Service National Research Ethics Service (ref. 11/NW/0382). All participants provided written informed consent. Ethical approval for the Avon Longitudinal Study of Parents and Children (ALSPAC) was obtained from the ALSPAC Ethics and Law Committee and the Local Research Ethics Committees. Written informed consent for the use of data collected *via* questionnaires and clinics was obtained from participants, parents or guardians following the recommendations of the ALSPAC Ethics and Law Committee at the time.

#### UK Biobank

The UK Biobank data resource is described elsewhere (www.ukbiobank.ac.uk). Genotyping was undertaken using the Affymetrix Axiom UK BiLEVE and UK Biobank arrays [17]. Genotypes were imputed based on the Human Reference Consortium panel as described elsewhere [18]. Genotyping quality control was performed as described previously [19]. UK Biobank individuals were selected for inclusion in PheWAS analyses if they met the following criteria: 1) they had no missing data for sex or age; 2) they had genome-wide imputed genetic data for rs28929474; 3) they were of genetically determined European ancestry (see [19] for details); and 4) they were not first- or second-degree relatives. For lung function analyses, individuals were additionally required to have spirometry data that passed quality control, as described previously [19], and to have full data for height and smoking status as well as data derived from direct genotyping for rs28929474 (N=303 353).

#### The Avon Longitudinal Study of Parents and Children

The ALSPAC cohort was invited to participate in this study after the main analyses in UK Biobank had been completed, in order to explore the effects of the Z allele on height and lung function in younger age groups. ALSPAC children were genotyped using the Illumina HumanHap550 quad genome-wide single-nucleotide polymorphism genotyping platform (Illumina Inc., San Diego, CA, USA) by the Wellcome Trust Sanger Institute (Cambridge, UK) and the Laboratory Corporation of America (Burlington, NC, USA), using support from 23andMe. Further details of ALSPAC are available in the supplementary material. Participants were excluded from this study if they had incorrectly recorded sex, minimal or excessive heterozygosity, disproportionate levels of individual missingness (>3%), evidence of cryptic relatedness or non-European ancestry.

### Phenome-wide association studies

To identify whether *SERPINA1* Z allele heterozygosity or homozygosity was associated with respiratory and nonrespiratory traits and diseases, two PheWASs across all available traits were performed. The first compared traits in individuals heterozygous for the Z allele *versus* wild type, and the second compared individuals homozygous for the Z allele *versus* wild type and heterozygotes combined (recessive genetic model). Up to 379 101 individuals were available for these analyses. Traits included UK Biobank baseline measures (from both questionnaires and physical measures), self-reported medication usage and operative procedures, as well as those captured in Office of Population Censuses and Surveys codes from the electronic health record. We also included self-reported disease variables and those from hospital episode statistics (HES) (International Classification of Disease, 10th revision (ICD-10) codes truncated to three-character codes, and combined in block and chapter groups) as well as combining both self-report and hospital-diagnosed diseases, where possible, to maximise power. The analysis included 2411 traits (traits with >200 cases were included [20]). Analyses were conducted in unrelated European-ancestry individuals (KING kinship coefficient of <0.0442), and were adjusted for age, age^2^, sex, genotyping array and 10 ancestry principal components (PCs). Logistic models were fitted for binary outcomes and linear models were fitted for quantitative outcomes (rank transformed to normality). False discovery rates (FDRs) were calculated according to the number of the traits in the analysis.

### Statistical analyses for biomarkers, lung function, COPD and height

For continuous traits, linear regression models adjusted for sex, age, age^2^, the first 10 ancestry-based PCs and genotyping array were tested in R. *SERPINA1* Z genotype (derived from direct genotyping) was coded according to the genetic model tested: either heterozygous (*i.e.* heterozygous *versus* wild type) or recessive. For lung function analyses, ever-smoking status and standing height were also included in the models unless indicated. To test for interaction of Z allele hetero- or homozygosity with ever-smoking status and sex, interaction terms for sex and smoking were added to the model. Association testing with moderate–severe COPD (defined as FEV_1_/FVC <0.7 and FEV_1_<80% predicted) or COPD of Global Initiative for Chronic Obstructive Lung Disease (GOLD) grade 1–4 (defined as FEV_1_/FVC <0.7) was carried out using logistic regression in R adjusting for covariates sex, age, age^2^, ever-smoking status, height, the first 10 ancestry-based PCs and genotyping array. In addition to the biomarkers available from UK Biobank, we calculated estimated glomerular filtration rate (eGFR) using the following formula: if cystatin C (cys)≤0.8, then eGFR=133*((cys/0.8)**−0.499)*(0.996**age)*[0.932 if female]; whereas if cys>0.8, then eGFR=133*((cys/0.8)**−1.328)*(0.996**age)*[0.932 if female].

In ALSPAC, the association of Z allele heterozygosity with height and lung function z-scores were examined using linear regression models including age and gender (and including height in the lung function analyses).

### Polygenic risk scores

Polygenic risk scores for lower FEV_1_/FVC were generated in UK Biobank using Plink version 1.90b3. These were based on 60 730 autosomal variants with minor allele frequency >1% and p<0.05 (for the association with FEV_1_/FVC in UK Biobank), and linkage disequilibrium pruned to r^2^<0.1 in 250-kb windows. Variants were weighted by FEV_1_/FVC β-coefficients from the SpiroMeta consortium cohorts [19].

## Results

### Frequency of the *SERPINA1* Z in UK Biobank

Amongst 411 002 unrelated, European UK Biobank participants with full sex, age, height, smoking status and genotyping data, 16 199 (3.94%) were heterozygous and 129 (0.0314%) were homozygous for the Z allele. There were fewer ZZ homozygotes than expected under Hardy–Weinberg equilibrium in all samples (p=0.004), ever-smokers (p=0.003), individuals >60 years of age (p=0.007) and females (p=0.007) (table S1), consistent with reduced survival.

### PheWASs of the Z allele

**FIGURE 1:**
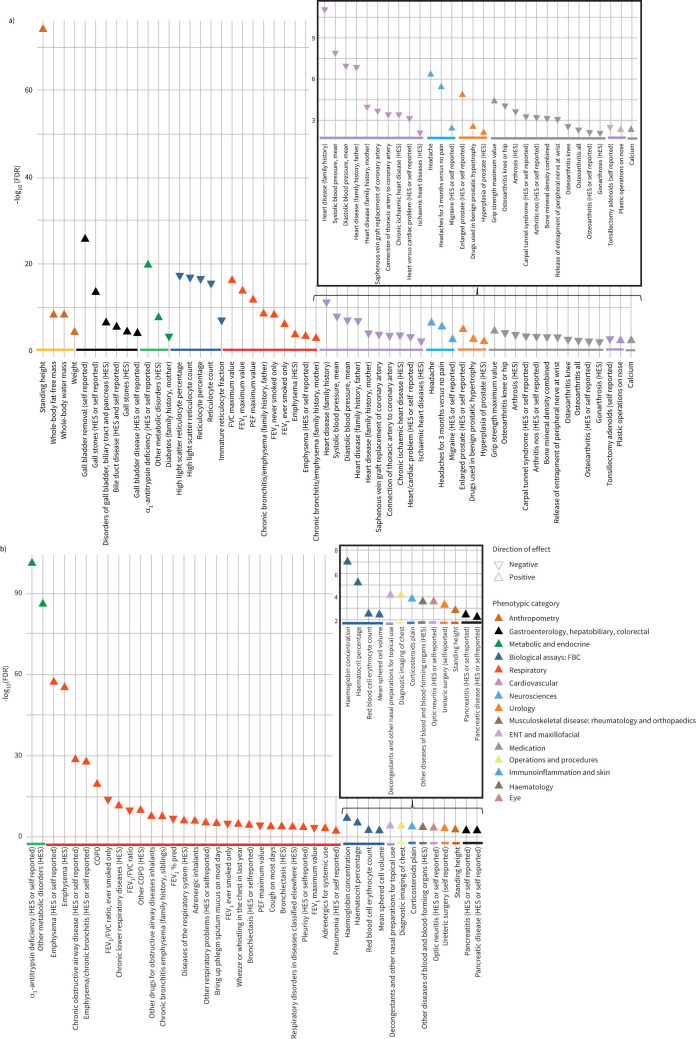
Traits associated with the *SERPINA1 Z* allele in UK Biobank. Traits displayed are those with a false discovery rate (FDR) <0.01 from phenome-wide association study of 2411 phenotypes across 379 101 UK Biobank participants using the a) heterozygous model for the Z allele (wild-type *versus* heterozygous individuals) and b) recessive model for the Z allele (wild-type and heterozygous individuals *versus* individuals homozygous for the Z allele). HES: hospital episode statistics; FBC: full blood count; FVC: forced vital capacity; FEV_1_: forced expiratory volume in 1 s; PEF: peak expiratory flow; ENT: ear, nose and throat.

To explore the effect of Z allele heterozygosity and homozygosity on a broad range of phenotypes, we performed two PheWASs. The phenotypes most strongly associated with Z allele heterozygosity and Z allele homozygosity (FDR <0.01) are shown in figure 1a and b respectively (full results are in tables S2 and S3). Standing height, AATD and various respiratory phenotypes (lung function, reported emphysema, chronic bronchitis and COPD) were strongly associated with the Z allele under either genetic model. However, we also detected strong associations between Z allele heterozygosity and a number of other phenotypes, including increased risk of gall stones (OR 1.35, p=1.69×10^−16^), gall bladder removal (OR 1.51, p=1.85×10^−29^) and bile duct disease (OR 1.25, p=5.42×10^−8^); cardiovascular phenotypes such as lower risk of heart disease (OR 0.84, p=6.66×10^−6^) and family history of heart disease, and lower blood pressure (p=1.02×10^−10^); musculoskeletal phenotypes such as a higher grip strength (p=5.67×10^−7^), lower risk of osteoarthritis, particularly of knee or hip (OR 0.85, p=1.18×10^−6^), lower bone mineral density (p=1.40×10^−5^) and higher risk of osteoporosis (OR 1.17, p=0.001); and higher risk of headache (OR 1.12, p=5.66×10^−9^) and enlarged prostate (OR 1.26, p=1.95×10^−7^). The PheWAS also included blood counts for a variety of different cell types, showing reduced reticulocytes in individuals heterozygous for the Z allele. Z allele homozygosity was associated with increased haemoglobin (p=7.17×10^−10^), haematocrit (p=6.17×10^−8^) and red blood cells (p=5.91×10^−5^), as well as optic neuritis (OR 26.9, p=4.32×10^−6^) and pancreatitis (OR 6.07, p=7.76×10^−5^).

To extend these analyses to traits not previously available for the PheWAS, we investigated the effect of Z allele heterozygosity and homozygosity on 30 key blood biochemistry markers in UK Biobank data (table 1). Heterozygosity for the Z allele was associated with a variety of liver function markers including higher alanine aminotransferase (ALT) (β=1.16 U·L^−1^, p=2.95×10^−25^), higher albumin (β=0.71 g·L^−1^, p=3.11×10^−229^), higher alkaline phosphatase (ALP) (β=2.97 U·L^−1^, p=5.81×10^−44^), higher aspartate aminotransferase (AST) (β=0.71 U·L^−1^, p=2.27×10^−16^) and higher direct bilirubin (β=0.041 μmol·L^−1^, p=1.64×10^−8^). There were also strong associations with calcium (β=0.012 mmol·L^−1^, p=4.01×10^−50^), cystatin C (0.0090 mg·L^−1^, p=8.91×10^−12^), C-reactive protein (β= −0.17 mg·L^−1^, p=1.11×10^−6^) and insulin-like growth factor (IGF)-1 (β= −0.21 nmol·L^−1^, p=2.35×10^−6^). Heterozygotes exhibited higher sex hormone-binding globulin (SHBG) (β=4.53 nmol·L^−1^, p=1.06×10^−95^) and testosterone (β=0.37 nmol·L^−1^, p=1.84×10^−57^). The association with higher testosterone was driven by heterozygosity in males (β±se=0.76±0.045 nmol·L^−1^, p=3.38×10^−64^) but not females (β±se= −0.030±0.0079 nmol·L^−1^, p=0.00015).

**TABLE 1:** Association between the *SERPINA1* Z allele and blood biomarkers in unrelated, European UK Biobank participants

**Biomarker**	**Heterozygous genetic model**		**Recessive genetic model**	
	**β±se**	**p-value**	**β±se**	**p-value**
**Alanine aminotransferase U·L^−1^**	1.163±0.112	2.95×10^−25^	3.037±1.246	0.015
**Albumin g·L^−1^**	0.712±0.022	3.11×10^−229^	0.306±0.243	0.208
**Alkaline phosphatase U·L^−1^**	2.968±0.213	5.81×10^−44^	2.379±2.373	0.316
**Apolipoprotein B g·L^−1^**	−0.0037±0.0021	0.082	0.042±0.024	0.086
**Apolipoprotein A g·L^−1^**	−0.0045±0.0019	0.020	0.019±0.022	0.375
**Aspartate aminotransferase U·L^−1^**	0.707±0.086	2.27×10^−16^	6.353±0.956	3.04×10^−11^
**C-reactive protein mg·L^−1^**	−0.174±0.036	1.11×10^−6^	0.192±0.396	0.628
**Calcium mmol·L^−1^**	0.012±0.00080	4.01×10^−50^	0.01±0.009	0.265
**Cholesterol mmol·L^−1^**	0.0051±0.0092	0.576	0.19±0.102	0.063
**Creatinine μmol·L^−1^**	−0.067±0.129	0.601	−0.94±1.435	0.512
**Cystatin C mg·L^−1^**	0.0090±0.0013	8.91×10^−12^	0.019±0.015	0.204
**Direct bilirubin μmol·L^−1^**	0.041±0.0073	1.64×10^−8^	0.158±0.079	0.046
**eGFR^#^**	−0.241±0.157	0.125	−1.810±1.732	0.296
**γ-Glutamyltransferase U·L^−1^**	0.253±0.342	0.460	8.045±3.818	0.035
**Glucose mmol·L^−1^**	−0.0032±0.010	0.758	−0.133±0.114	0.244
**HbA1c mmol·mol^−1^**	−0.187±0.052	3.40×10^−4^	−1.781±0.588	2.46×10^−3^
**HDL–cholesterol mmol·L^−1^**	0.0015±0.0030	0.605	0.142±0.033	2.01×10^−5^
**IGF-1 nmol·L^−1^**	−0.212±0.045	2.35×10^−6^	−2.245±0.5	7.19×10^−6^
**LDL direct mmol·L^−1^**	0.011±0.007	0.113	0.115±0.079	0.145
**Lipoprotein A nmol·L^−1^**	−0.324±0.452	0.474	−0.151±5.115	0.976
**Oestradiol pmol·L^−1^**	9.577±8.441	0.257	88.051±96.144	0.360
**Phosphate mmol·L^−1^**	0.0027±0.0013	0.042	−0.014±0.015	0.352
**Rheumatoid factor IU·mL^−1^**	0.113±0.529	0.832	−3.27±5.7	0.566
**SHBG nmol·L^−1^**	4.531±0.218	1.06×10^−95^	40.573±2.402	5.33×10^−64^
**Testosterone nmol·L^−1^**	0.37±0.023	1.84×10^−57^	2.986±0.258	6.96×10^−31^
**Total bilirubin μmol·L^−1^**	0.146±0.035	3.54×10^−5^	0.52±0.391	0.184
**Total protein g·L^−1^**	0.578±0.035	1.06×10^−62^	0.459±0.382	0.229
**Triglycerides mmol·L^−1^**	−0.023±0.0082	4.69×10^−3^	−0.324±0.092	4.21×10^−4^
**Urate μmol·L^−1^**	1.096±0.56	0.050	−1.325±6.252	0.832
**Urea mmol·L^−1^**	0.051±0.011	2.69×10^−6^	0.06±0.122	0.620
**Vitamin D nmol·L^−1^**	−0.041±0.175	0.816	−2.547±1.955	0.193

Results are based on linear regression adjusting for sex, age, age^2^, ancestry-based principal components and genotyping array. The heterozygous or recessive genetic model was also included in the regression as shown. eGFR: estimated glomerular filtration rate; HbA1c: glycated haemoglobin; HDL: high-density lipoprotein; IGF: insulin-like growth factor; LDL: low-density lipoprotein; SHBG: sex hormone-binding globulin. ^#^: calculated using the following formula: if cystatin C (cys)≤0.8, then eGFR=133*((cys/0.8)**−0.499)*(0.996**age)*[0.932 if female]; whereas if cys>0.8, then eGFR=133*((cys/0.8)**−1.328)*(0.996**age)*[0.932 if female].

### Z allele heterozygosity is associated with increased lung function in nonsmokers

We tested association of heterozygosity for the *SERPINA1* Z allele with lung function traits in unrelated, European UK Biobank participants with full sex, age, height, smoking status, lung function and genotyping data (table 2). Individuals heterozygous for the Z allele exhibited higher FEV_1_ (β=9.26 mL, p=0.041) but no association with FEV_1_/FVC or FVC compared to wild type (table 2). However, in stratified analyses of UK Biobank never- and ever-smokers, we found that heterozygosity for the Z allele was associated with a large increase in FEV_1_ (β=19.36 mL, p=9.21×10^−4^) and increased FEV_1_/FVC (β=0.0031, p=1.22×10^−5^) in never-smokers, but not in ever-smokers (table 2). The FEV_1_ association in never-smokers was only slightly attenuated by including a quadratic height term in the regression (β=18.85 mL, p=1.23×10^−3^). Statistical tests of Z allele*ever-smoking interactions showed interactions for FEV_1_ (p=0.022) and FEV_1_/FVC (p=1.06×10^−4^) (table S4). Furthermore, heterozygous ever-smokers exhibited a small increased risk of COPD (both moderate–severe COPD (OR 1.16, p=0.005) and COPD GOLD 1–4 (OR 1.12, p=0.001)), whereas heterozygous never-smokers did not (table 2).

**TABLE 2:** Association between the *SERPINA1* Z allele and height, lung function traits, and COPD in unrelated, European UK Biobank participants

**Genetic model**	**Smoking status**	**Test/comparison n**	**Height cm**		**FEV_1_ mL**		**FEV_1_/FVC**		**FVC mL**		**COPD**^#^		**COPD GOLD 1–4**^¶^	
			**β±se**	**p-value**	**β±se**	**p-value**	**β±se**	**p-value**	**β±se**	**p-value**	**OR (95% CI)**	**p-value**	**OR (95% CI)**	**p-value**
**Het**	All	11 877/291 383	1.023±0.059	3.91×10^−68^	9.26±4.53	0.041	0.00091±0.00057	0.108	9.38±5.43	0.084	1.05 (0.96–1.14)	0.268	1.04 (0.98–1.09)	0.171
	Ever-smokers	5357/133 779	1.027±0.087	8.53×10^−32^	−2.63±7.05	0.709	−0.0017±0.00091	0.062	5.47±8.28	0.509	1.16 (1.05–1.29)	0.005	1.12 (1.04–1.20)	0.001
	Never-smokers	6520/157 604	1.022±0.079	3.03×10^−38^	19.36±5.84	9.21×10^−4^	0.0031±0.00071	1.22×10^−5^	12.77±7.15	0.074	0.87 (0.75–1.01)	0.070	0.93 (0.86–1.02)	0.113
**Rec**	All	93/303 260	1.644±0.650	0.011	−249.21±50.22	6.96×10^−7^	−0.051±0.0063	3.54×10^−16^	−132.16±60.09	0.028	7.42 (4.29–12.27)	5.67×10^−14^	4.58 (2.93–7.05)	8.67×10^−12^
	Ever-smokers	35/139 136	1.242±1.062	0.242	−379.21±85.54	9.30×10^−6^	−0.081±0.011	2.00×10^−13^	−177.95±100.35	0.076	9.19 (4.19–19.00)	6.27×10^−9^	6.13 (3.07–12.29)	2.30×10^−7^
	Never-smokers	58/164 124	1.873±0.821	0.023	−174.68±60.68	0.004	−0.034±0.0074	4.95×10^−6^	−107.69±74.23	0.147	5.98 (2.66–12.03)	2.69×10^−6^	3.68 (2.01–6.47)	1.11×10^−5^

Results are based on linear (or logistic, in the case of COPD) regression adjusting for sex, age, age^2^, ancestry-based principal components, genotyping array and, in the case of lung function and COPD, standing height. The heterozygote (Het) or recessive (Rec) genetic model was also included in the regression as shown. FEV_1_: forced expiratory volume in 1 s; FVC: forced vital capacity; GOLD: Global Initiative for Chronic Obstructive Lung Disease. ^#^: spirometrically defined as FEV_1_/FVC <0.7 and FEV_1_<80% predicted; ^¶^: defined as FEV_1_/FVC <0.7.

### The Z allele is strongly associated with height

Heterozygosity for the Z allele was strongly associated with height (β=1.02 cm, p=3.91×10^−68^) (table 2) and, when lung function measures were not adjusted for height, the Z allele heterozygosity had much larger effect estimates for FEV_1_ (β±se=44.72±4.97 mL, p=2.20×10^−19^) and FVC (β±se=61.06±6.18 mL, p=5.18×10^−23^) but not FEV_1_/FVC. These results suggest that carrying one copy of the Z allele confers an advantage primarily (but not entirely) driven by increased height. To investigate the developmental stage at which Z allele heterozygosity influences height, we tested association with height in the ALPSAC cohort at ages 8, 15 and 24 years. The findings were consistent with Z allele heterozygosity influencing height from adolescence and, at all the ages, directions of effect on height and lung function were consistent with those in UK Biobank (table S5).

### Sex modifies the effect of Z allele heterozygosity on lung function

We also assessed whether sex can modify the effect of the Z allele on lung function or height and found that heterozygosity for the Z allele was associated with higher height-adjusted FEV_1_/FVC in females (β±se=0.0028±0.00071, p=5.89×10^−5^) but not in males (β±se= −0.0015±0.0009, p=0.093) (Z allele*sex interaction: p=0.00021 for FEV_1_/FVC) (tables S4 and S6).

### Association between Z allele homozygosity and lung function

In contrast to the lung function-raising effects of carrying one copy of the Z allele, homozygosity for the Z allele has been widely reported to reduce lung function and increase risk of COPD. Similarly, UK Biobank individuals homozygous for the Z allele had significantly lower FEV_1_ (β= −249.21 mL, p=6.96×10^−7^), FEV_1_/FVC (β= −0.051, p=3.54×10^−16^) and FVC (β= −132.16, p=0.028) compared to wild type and heterozygous individuals, and greater risk of COPD (both moderate–severe COPD (OR 7.42, p=5.67×10^−14^) and COPD GOLD 1–4 (OR 4.58, p=8.67×10^−12^)) (table 2). The associations between homozygosity for the Z allele and reduced lung function and increased risk of COPD were stronger in smokers compared to nonsmokers (table 2). Given the strength of these associations, it is interesting to note that 58 out of 93 individuals homozygous for the Z allele did not have spirometrically defined COPD (FEV_1_/FVC <0.7) and four out of 15 homozygous ever-smokers aged >60 years did not have spirometrically defined COPD. We generated polygenic risk scores for lower FEV_1_/FVC by weighting variants according to β-coefficients in the SpiroMeta consortium cohorts [19]. The 58 individuals homozygous for the Z allele but without COPD have an average score percentile of 47.6 compared to 57.0 in those with COPD. This suggests that individuals homozygous for the Z allele but without COPD may have a more protective genetic profile across other genomic loci.

### Diagnosis of AATD in individuals homozygous for the Z allele

We note that only 20 out of 141 individuals who were genotyped ZZ homozygotes in the full UK Biobank cohort had a recorded ICD-10 coding for AATD (E880) in HES. As this could indicate miscoding rather than misdiagnosis, we investigated further in a subset of 65 homozygotes with linked primary care data. Of these 65 ZZ homozygotes, 15 had primary care (Read) codes or ICD-10 codes for AATD and 50 did not. Of the group without AATD codes, four had Read codes or ICD-10 codes for bronchiectasis and an additional six had primary care (Read) codes or ICD-10 codes for COPD, indicating potential misdiagnosis of AATD.

## Discussion

We describe novel phenotypic associations of heterozygosity for the Z allele of *SERPINA1* from a PheWAS of UK Biobank, and present definitive evidence that heterozygosity for the Z allele is associated with greater FEV_1_ and FEV_1_/FVC only in nonsmokers. Our findings have implications for individuals heterozygous for the *SERPINA1* Z allele, and more generally for the study of rare variants and gene–environment interactions.

Our PheWAS examining the consequences of carrying one copy of the *SERPINA1* Z allele revealed potentially important associations with nonrespiratory traits and diseases. Consistent with previous reports [12], we detected a very strong association between Z allele heterozygosity and height (with each additional Z allele adding ∼1 cm to height). The mechanism and timing of the effect of the Z allele on height is not understood. Our findings in the ALSPAC study suggest a possible influence of Z allele heterozygosity on height that is manifest from puberty, although results should be interpreted with caution given the limitations in power in ALSPAC alone. This suggests that sex hormones are a possible mediator of the height-raising effects of the Z allele and, indeed, we detected higher levels of testosterone in individuals heterozygous for the Z allele. However, heterozygosity for the Z allele is associated with increased height in males and females, whereas increased testosterone was only seen in males. Furthermore, Z allele heterozygotes also exhibit higher SHBG, suggesting that the amount of free testosterone will be unchanged. IGF-1 levels relate to height but in our study, the magnitude of the association was very small (<1% average levels) and it was a negative association. It is therefore still not clear why heterozygosity and homozygosity for the Z allele is associated with increased height.

We detected strong associations between heterozygosity for the Z allele and increased risk of gall stones and gall bladder removal. This is consistent with recent reports that the Z allele may be a risk factor for developing gallstone disease [21, 22]. The mechanism underlying this association is unclear but previous reports have suggested it may be related to liver dysfunction in AATD and/or the composition of bile [21, 22]. We found that individuals heterozygous for the Z allele also had increased levels of ALP, which is characteristic of liver disease and biliary tract obstruction due to gall stones. Moreover, the Z allele is associated with elevation of ALT and AST. Higher than normal levels of ALT and AST are hallmarks of liver disease, and are also observed in some patients with gall bladder disease [23]. Higher bilirubin, another potential sign of liver disease, is also exhibited by Z allele carriers. Interestingly, excess bilirubin is thought to contribute to gall stone formation [24], so a causal pathway between the Z allele, liver dysfunction and gall bladder problems is possible.

Heterozygosity for the Z allele was also associated with musculoskeletal phenotypes such as reduced risk of osteoarthritis, increased risk of osteoporosis and lower bone mineral density. Calcium was increased in heterozygotes but, as albumin was also increased, free calcium may not be different in this group. A recent study showed that α_1_-antitrypsin (AAT) can inhibit receptor activator of NF-κB ligand (RANKL)-induced osteoclast formation and bone resorption [25]. As heterozygosity for the Z allele causes reduction in AAT, individuals heterozygous for the Z allele may have increased risk of osteoporosis and lower bone mineral density due to increased RANKL-induced bone resorption. Lower bone mineral density has been associated with a lower risk of osteoarthritis [26, 27].

Heterozygotes for the Z allele also had reduced risk of heart disease (even when adjusted for height) and lower blood pressure, as well as increased risk of headache, consistent with one previous report suggesting cluster headaches may be more frequent in heterozygotes for the Z allele [6]. There was also a marked association between heterozygosity and reduced reticulocyte count and reticulocyte percentage, which, as far as we are aware, has not been reported previously.

Homozygosity for the Z allele was strongly associated with self-reported/HES-recorded AATD and respiratory traits but also haemoglobin concentration and haematocrit percentage, which may be driven by the associations with respiratory disease. Homozygosity was also associated with markedly higher risk of optic neuritis and pancreatitis. These associations should be interpreted with care, especially the association with optic neuritis, as small case numbers may have resulted in poor estimation of effect sizes.

While homozygosity for the Z allele is an established cause of respiratory disease, the effect of heterozygosity on the respiratory health of general populations has been less clear. Our study provides definitive data on the effects of heterozygosity for the Z allele on respiratory traits, showing that heterozygosity is associated with greater lung function in never-smokers (even after height adjustment) but not in smokers. Our results contrast with previous studies [8–11] that did not detect higher lung function in participants heterozygous for the Z allele compared to wild type and with studies that did not detect differential effects by smoking status [12]. There are several possible reasons for these discrepancies. First, the relatively low frequency of the Z allele meant previous studies were underpowered. Our study contained 18 times more heterozygous participants than the previous largest population-based study and 46 times more than the largest case–control study. Whilst one previous study has suggested a beneficial effect of Z allele heterozygosity on lung function [12], it nevertheless did not detect differential effects by smoking status. Again, this is probably due to power. Second, most of the previous studies were conducted in current and former smokers only [8, 9, 11], and would therefore have missed the effect of Z allele heterozygosity in nonsmokers. Third, most previous studies recruited participants based on their health status (as in case–control studies [8, 9]) or the health status of a family member [10, 11, 28]. These approaches can lead to ascertainment biases that might distort causal estimates. A major strength of using the UK Biobank for our study was that participants were not ascertained based on having lung disease, enabling a relatively unbiased assessment of the role of the heterozygosity for the Z allele in lung function. It should be noted, however, that UK Biobank participants tend to be healthier and more highly educated than the UK population as a whole, so detrimental effects of the Z allele may be less pronounced in UK Biobank due to fewer lifestyle risk factors. Fourth, the Z allele is not present on most standard genotyping arrays and therefore, high-quality imputation is required to detect its effects. Furthermore, genetic studies tend to focus on the additive genetic model rather than heterozygous or recessive models. Because we designed the Z allele into the UK Biobank array [17] and compared different genetic models, we were able to robustly examine this association in UK Biobank participants.

The biological mechanism underlying the association between heterozygosity for the Z allele and increased lung function in nonsmokers is not clear. The *SERPINA1* gene encodes AAT, a glycoprotein with an important role in inhibiting proteases, such as neutrophil elastase, which are secreted during inflammation and cause collateral tissue damage. The Z allele produces AAT with reduced antiprotease activity and a propensity to form polymers, which accumulate in liver, thereby reducing the circulating levels of AAT. The polymers themselves have also been shown to be a proinflammatory stimulus in lung tissue [29], which further perpetuates lung inflammation and damage. Homozygosity for the Z allele is the most common cause of severe AATD, whereas heterozygosity for the Z allele has been shown to result in intermediate levels of AAT (∼60% of normal) [30]. One might therefore expect heterozygosity to have, if anything, a detrimental effect on lung function. There is evidence that positive selection has acted at the Z allele locus [12, 31] and mechanisms of heterozygous advantage (through increased height [12] and protection from infectious respiratory diseases by promotion of inflammatory responses [31]) have been proposed. The amplification of the inflammatory response to cigarette smoke may therefore provide a mechanism for eliminating this advantage in smokers. It has also been shown that smoking-induced oxidative modifications to AAT reduce its ability to inhibit neutrophil elastase and turn it into a proinflammatory mediator [32]. Cigarette smoke has been found to accelerate polymerisation of Z-type AAT [33], suggesting a possible mechanism whereby smoking may modify the effect of the Z allele on lung function.

We also report a novel interaction between sex and the *SERPINA1* Z allele heterozygosity on FEV_1_/FVC. Women heterozygous for the Z allele exhibited greater FEV_1_/FVC compared to wild-type, whereas men heterozygous for the Z allele showed no significant difference to wild type. Previous studies have shown that individuals with the fastest decline in lung function amongst AATD patients are more likely to be male [15]. However, the reasons why sex would appear to modify the effect of Z allele heterozygosity on lung function are not clear.

Finally, we confirm that homozygosity for the Z allele is associated with reduced lung function and increased risk of COPD, and that these effects are exacerbated by smoking. These results are consistent with a recently published study in UK Biobank focused on homozygosity for the Z allele [34]. Also consistent with the work of Nakanishi *et al*. [34], we show that only a minority of individuals with the ZZ genotype have been diagnosed with AATD (as has been reported elsewhere [35, 36]), and that those without a diagnosis of AATD, bronchiectasis or COPD have a protective genetic profile at other known lung function loci.

In conclusion, we have demonstrated that heterozygosity for the Z allele is associated with previously unrecognised nonrespiratory phenotypes. Further studies will be required to confirm these findings in independent populations; produce accurate effect estimates; and understand the causal pathways involved, and the potential role of genetic and environmental modifiers. We have also shown that heterozygosity for the Z allele is associated with greater lung function in nonsmokers. Our results demonstrate that large sample sizes are required to study the effects of rare variants, particularly when these effects depend on an environmental stimulus. The opposing directions of effect of Z allele heterozygosity and homozygosity on lung function demonstrate that the additive genetic model is not always appropriate for genetic studies of rarer variants such as the *SERPINA1* Z allele. Our findings suggest that while individuals who are heterozygous for the Z allele may not exhibit symptoms of AATD, they may be more susceptible to smoking-induced lung disease and have altered risk of other nonrespiratory conditions.

## Supplementary material

10.1183/23120541.00049-2021.Supp1 **Please note:** supplementary material is not edited by the Editorial Office, and is uploaded as it has been supplied by the author.
Supplementary material 00049-2021.SUPPLEMENT
Table S1 00049-2021.TableS1
Table S2 00049-2021.TableS2
Table S3 00049-2021.TableS3
Table S4 00049-2021.TableS4
Table S5 00049-2021.TableS5
Table S6 00049-2021.TableS6
