# Supplementary material for: Pleiotropic associations of heterozygosity for the SERPINA1 Z allele in the UK Biobank
Source: ERJ Open Res. 2021 May 10;7(2):00049-2021. doi: 10.1183/23120541.00049-2021 (PMC8107350; doi:10.1183/23120541.00049-2021)
Supplement: Supplementary file 1 [file 00049-2021.SUPPLEMENT.pdf]

Online Data Supplement

Pleiotropic associations of heterozygosity for the *SERPINA1* Z allele in the UK Biobank

Katherine A Fawcett, Kijoung Song, Guoqing Qian, Aliki-Eleni Farmaki, Richard Packer, Catherine John, Nick Shrine, Raquel Granell, Sue Ring, Nicholas J Timpson, Laura M Yerges-Armstrong, Richard Eastell, Louise V Wain, Robert A Scott, Martin D Tobin, Ian P Hall

Supplemental Subjects and Methods

ALSPAC study: original cohort description

Pregnant women resident in Avon, UK with expected dates of delivery 1st April 1991 to 31st December 1992 were invited to take part in the study. The initial number of pregnancies enrolled is 14,541 (for these at least one questionnaire has been returned or a “Children in Focus” clinic had been attended by 19/07/99). Of these initial pregnancies, there was a total of 14,676 foetuses, resulting in 14,062 live births and 13,988 children who were alive at 1 year of age.

When the oldest children were approximately 7 years of age, an attempt was made to bolster the initial sample with eligible cases who had failed to join the study originally. As a result, when considering variables collected from the age of seven onwards (and potentially abstracted from obstetric notes) there are data available for more than the 14,541 pregnancies mentioned above. The number of new pregnancies not in the initial sample (known as Phase I enrolment) that are currently represented on the built files and reflecting enrolment status at the age of 24 is 913 (456, 262 and 195 recruited during Phases II, III and IV respectively), resulting in an additional 913 children being enrolled. The phases of enrolment are described in more detail in the cohort profile paper and its update. The total sample size for analyses using any data collected after the age of seven is therefore 15,454 pregnancies, resulting in 15,589 foetuses. Of these 14,901 were alive at 1 year of age.

A 10% sample of the ALSPAC cohort, known as the Children in Focus (CiF) group, attended clinics at the University of Bristol at various time intervals between 4 to 61 months of age. The CiF group were chosen at random from the last 6 months of ALSPAC births (1432 families attended at least one clinic). Excluded were those mothers who had moved out of the area or were lost to follow-up, and those partaking in another study of infant development in Avon.

Ethical approval for the study was obtained from the ALSPAC Ethics and Law Committee and the Local Research Ethics Committees. Further details are available in the cohort profile papers[1-3]. Study data were collected and managed using REDCap electronic data capture tools[4, 5] hosted at University of Bristol. The study website contains details of all the data that is available through a fully searchable data dictionary and variable search tool: http://www.bristol.ac.uk/alspac/researchers/our-data/

Informed consent for the use of data collected via questionnaires and clinics was obtained from participants following the recommendations of the ALSPAC Ethics and Law Committee at the time.

ALSPAC study: data collection

Outcome 1: Height

Longitudinal measurements of height were obtained from direct measurement in research clinics at age at 8, 15 and 24 years. Standing height was measured to the last complete millilitre by trained clinic staff.

Outcome 2: Lung function Data

Spirometry was performed according to ATS/ERS criteria[6, 7] by trained fieldworkers in a research clinic at ages 8, 15 and 24 years. All flow-volume curves were inspected post hoc for quality assurance. Lung function at ages 15 and 24 years were measured before and 15 minutes after receiving 400 µg of salbutamol[8, 9]. The highest measurement of each lung function parameter, FVC, FEV_1, forced expiratory flow, mid-expiratory phase (FEF_{25-75}), amongst the best three technically acceptable flow-volume curves was used for analyses. Post-bronchodilator lung function variables were used at age 15 and 24 years. Standardised scores (SD-scores) adjusted for sex, age and height at lung function clinic, were used as the lung function outcomes.

PheWAS: overview of study design

Our phenome-wide association study (pheWAS) in UK Biobank tests association between a single genetic variant (the *SERPINA1* Z allele) and a broad range of phenotypic traits available in UK Biobank participants, including those derived from direct measurement such as height, weight, and blood biomarkers, to health conditions self-reported at an assessment centre visit or from hospital inpatient records, also called hospital episode statistics (HES) data. We used logistic regression models to test association between the Z allele and binary health outcomes, and linear regression models to test association between the Z allele and continuous traits. We used minimal adjustments for covariates in order that analyses did not inadvertently adjust for intermediates on a causal pathway or induce collider bias[10]. These models therefore included (i) age, (ii) sex, (iii) genotyping array to account for the two different genotyping arrays used to measure genetic variation in UK Biobank participants, and (iv) ancestry-based principal components to account for fine-scale population structure within UK Biobank participants of European ancestry.

Supplemental References

1. Boyd A, Golding J, Macleod J, Lawlor DA, Fraser A, Henderson J, Molloy L, Ness A, Ring S, Davey Smith G. Cohort Profile: the 'children of the 90s'--the index offspring of the Avon Longitudinal Study of Parents and Children. *Int J Epidemiol* 2013: 42(1): 111-127.
2. Fraser A, Macdonald-Wallis C, Tilling K, Boyd A, Golding J, Davey Smith G, Henderson J, Macleod J, Molloy L, Ness A, Ring S, Nelson SM, Lawlor DA. Cohort Profile: the Avon Longitudinal Study of Parents and Children: ALSPAC mothers cohort. *Int J Epidemiol* 2013: 42(1): 97-110.
3. Northstone K, Lewcock M, Groom A, Boyd A, Macleod J, Timpson N, Wells N. The Avon Longitudinal Study of Parents and Children (ALSPAC): an update on the enrolled sample of index children in 2019. *Wellcome Open Res* 2019: 4: 51.
4. Harris PA, Taylor R, Minor BL, Elliott V, Fernandez M, O'Neal L, McLeod L, Delacqua G, Delacqua F, Kirby J, Duda SN, Consortium RE. The REDCap consortium: Building an international community of software platform partners. *J Biomed Inform* 2019: 95: 103208.
5. Harris PA, Taylor R, Thielke R, Payne J, Gonzalez N, Conde JG. Research electronic data capture (REDCap)--a metadata-driven methodology and workflow process for providing translational research informatics support. *J Biomed Inform* 2009: 42(2): 377-381.
6. Standardization of Spirometry, 1994 Update. American Thoracic Society. *Am J Respir Crit Care Med* 1995: 152(3): 1107-1136.
7. Beydon N, Davis SD, Lombardi E, Allen JL, Arets HG, Aurora P, Bisgaard H, Davis GM, Ducharme FM, Eigen H, Gappa M, Gaultier C, Gustafsson PM, Hall GL, Hantos Z, Healy MJ, Jones MH, Klug B, Lodrup Carlsen KC, McKenzie SA, Marchal F, Mayer OH, Merkus PJ, Morris MG, Oostveen E, Pillow JJ, Seddon PC, Silverman M, Sly PD, Stocks J, Tepper RS, Vilozni D, Wilson NM, American Thoracic Society/European Respiratory Society Working Group on I, Young Children Pulmonary Function T. An official American Thoracic Society/European Respiratory Society statement: pulmonary function testing in preschool children. *Am J Respir Crit Care Med* 2007: 175(12): 1304-1345.
8. Crapo RO, Casaburi R, Coates AL, Enright PL, Hankinson JL, Irvin CG, MacIntyre NR, McKay RT, Wanger JS, Anderson SD, Cockcroft DW, Fish JE, Sterk PJ. Guidelines for methacholine and exercise challenge testing-1999. This official statement of the American Thoracic Society was adopted by the ATS Board of Directors, July 1999. *Am J Respir Crit Care Med* 2000: 161(1): 309-329.
9. Pellegrino R, Decramer M, van Schayck CP, Dekhuijzen PN, Troosters T, van Herwaarden C, Olivieri D, Del Donno M, De Backer W, Lankhorst I, Ardia A. Quality control of spirometry: a lesson from the BRONCUS trial. *Eur Respir J* 2005: 26(6): 1104-1109.
10. VanderWeele TJ. Principles of confounder selection. *Eur J Epidemiol* 2019: 34(3): 211-219.
