# Supplementary material for: Pleiotropic associations of heterozygosity for the SERPINA1 Z allele in the UK Biobank
Source: ERJ Open Res. 2021 May 10;7(2):00049-2021. doi: 10.1183/23120541.00049-2021 (PMC8107350; doi:10.1183/23120541.00049-2021)
Supplement: Supplementary file 2 [file 00049-2021.TableS1.pdf]

Table S1. Frequency of the *SERPINA1* Z allele in 411,002 unrelated European UK Biobank participants

Sex	Smoking status	Age group	MM	MZ	ZZ	N	M	Z	O(MM)	E(MM)	O(MZ)	E(MZ)	O(ZZ)	E(ZZ)	Chi-squared	P
All	All	All	394674	16199	129	411002	0.979979	0.020021	394674	394709.7	16199	16127.52	129	164.7394	8.073513332	**0.004**
	Ever-smokers	All	180774	7264	47	188085	0.98044	0.01956	180774	180799	7264	7214.075	47	71.96236	9.007913227	**0.003**
	Never-smokers	All	213900	8935	82	222917	0.979591	0.020409	213900	213910.9	8935	8913.299	82	92.85048	1.321368824	0.250
	All	40-59 years	219339	8852	76	228267	0.980277	0.019723	219339	219351.8	8852	8826.418	76	88.79078	1.917466848	0.166
		60+ years	175335	7347	53	182735	0.979607	0.020393	175335	175358	7347	7301.012	53	75.99421	7.2502453	**0.007**
	Ever-smokers	40-59 years	92580	3664	23	96267	0.980731	0.019269	92580	92592.74	3664	3638.511	23	35.7446	4.724351694	0.030
		60+ years	88194	3600	24	91818	0.980135	0.019865	88194	88206.23	3600	3575.531	24	36.23446	4.300080537	0.038
	Never-smokers	40-59 years	126759	5188	53	132000	0.979947	0.020053	126759	126759.1	5188	5187.839	53	53.08037	0.000126725	0.991
		60+ years	87141	3747	29	90917	0.979074	0.020926	87141	87151.81	3747	3725.378	29	39.81111	3.06270122	0.080
Males	All	All	182549	7409	64	190022	0.980168	0.019832	182549	182559.7	7409	7387.527	64	74.73657	1.605449841	0.205
	Ever-smokers	All	96014	3850	26	99890	0.980469	0.019531	96014	96026.11	3850	3825.788	26	38.10593	4.000702309	0.045
	Never-smokers	All	86535	3559	38	90132	0.979835	0.020165	86535	86533.65	3559	3561.701	38	36.64965	0.051822323	0.820
	All	40-59 years	98442	3880	33	102355	0.980724	0.019276	98442	98447.03	3880	3869.937	33	38.03164	0.6921198	0.405
		60+ years	84107	3529	31	87667	0.979519	0.020481	84107	84112.77	3529	3517.453	31	36.77348	0.944743965	0.331
Females	All	All	212125	8790	65	220980	0.979817	0.020183	212125	212150	8790	8739.969	65	90.01539	7.241150451	**0.007**
	Ever-smokers	All	84760	3414	21	88195	0.980407	0.019593	84760	84772.86	3414	3388.287	21	33.85661	5.079217263	**0.024**
	Never-smokers	All	127365	5376	44	132785	0.979425	0.020575	127365	127377.2	5376	5351.58	44	56.20984	2.764808602	0.096
	All	40-59 years	120897	4972	43	125912	0.979915	0.020085	120897	120904.8	4972	4956.408	43	50.79612	1.246091786	0.264
		60+ years	91228	3818	22	95068	0.979688	0.020312	91228	91245.22	3818	3783.556	22	39.22204	7.878858049	**0.005**
Males	Ever-smokers	40-59 years	45844	1762	9	47615	0.981308	0.018692	45844	45851.64	1762	1746.729	9	16.63551	3.639395806	0.056
		60+ years	50170	2088	17	52275	0.979703	0.020297	50170	50174.53	2088	2078.931	17	21.5346	0.994835197	0.319
	Never-smokers	40-59 years	52598	2118	24	54740	0.980216	0.019784	52598	52595.43	2118	2123.147	24	21.42654	0.321690548	0.571
		60+ years	33937	1441	14	35392	0.979247	0.020753	33937	33938.24	1441	1438.513	14	15.24328	0.105749132	0.745
Females	Ever-smokers	40-59 years	46736	1902	14	48652	0.980165	0.019835	46736	46741.14	1902	1891.719	14	19.14053	1.437020728	0.231
		60+ years	38024	1512	7	39543	0.980705	0.019295	38024	38031.72	1512	1496.555	7	14.72243	4.211647147	0.040
	Never-smokers	40-59 years	74161	3070	29	77260	0.979757	0.020243	74161	74163.66	3070	3064.679	29	31.66057	0.232914036	0.629
		60+ years	53204	2306	15	55525	0.978964	0.021036	53204	53213.57	2306	2286.861	15	24.56955	3.88912281	0.049
