# Supplementary material for: Pleiotropic associations of heterozygosity for the SERPINA1 Z allele in the UK Biobank
Source: ERJ Open Res. 2021 May 10;7(2):00049-2021. doi: 10.1183/23120541.00049-2021 (PMC8107350; doi:10.1183/23120541.00049-2021)
Supplement: Supplementary file 3 [file 00049-2021.TableS2.pdf]

Table S2. Phenome-wide association study results for *SERPINA1* Z allele heterozygosity in UK Biobank

Category	Phenotype	Effect allele	N	Cases	Controls	OR	Beta	SE	L95	U95	P	FDR
Anthropometry	Standing height	T	378103	NA	NA	NA	0.154838	0.008263	0.138642	0.171034	2.42E-78	5.84E-75
Operations and Procedures	Gall bladder removal (self-reported)	T	378957	14872	364229	1.51	0.412834	0.036632	1.41	1.62	1.85E-29	2.23E-26
Respiratory	Alpha1 antitrypsin deficiency (HES and self-reported)	T	378957	223	378878	5.82	1.76184	0.176835	4.12	8.24	2.21E-23	1.77E-20
Biological assays	High light scatter reticulocyte percentage	T	361631	NA	NA	NA	-0.07962	0.008511	-0.0963	-0.06293	8.41E-21	5.07E-18
Biological assays	High light scatter reticulocyte count	T	361630	NA	NA	NA	-0.07841	0.008514	-0.0951	-0.06173	3.26E-20	1.57E-17
Biological assays	Reticulocyte percentage	T	361630	NA	NA	NA	-0.07775	0.008515	-0.09443	-0.06106	6.82E-20	2.74E-17
Respiratory	FVC maximumValue	T	286064	NA	NA	NA	0.086208	0.009563	0.067463	0.104952	1.98E-19	6.82E-17
Biological assays	Reticulocyte count	T	361630	NA	NA	NA	-0.07516	0.008517	-0.09185	-0.05847	1.09E-18	3.29E-16
Respiratory	FVC maximumValue strict*	T	221584	NA	NA	NA	0.092784	0.010876	0.071467	0.114101	1.45E-17	3.88E-15
Respiratory	FEV1 maximumValue	T	286064	NA	NA	NA	0.079709	0.009571	0.06095	0.098468	8.21E-17	1.98E-14
Digestive system	Gall stones (HES and self-reported)	T	378957	16555	362546	1.35	0.298659	0.036235	1.26	1.45	1.69E-16	3.70E-14
Respiratory	FEV1 maximumValue strict	T	221584	NA	NA	NA	0.088488	0.010886	0.067152	0.109823	4.33E-16	8.70E-14
Respiratory	PEF maximumValue	T	286064	NA	NA	NA	0.073969	0.009581	0.055189	0.092748	1.16E-14	2.15E-12
Family history	Heart disease (family history)	T	378957	NA	NA	NA	-0.06269	0.008312	-0.07898	-0.0464	4.63E-14	7.96E-12
Respiratory	PEF maximumValue strict	T	221584	NA	NA	NA	0.077439	0.0109	0.056074	0.098803	1.21E-12	1.94E-10
Family history	Chronic bronchitis emphysema (family history - father)	T	343458	37574	306018	1.19	0.176499	0.026529	1.13	1.26	2.87E-11	4.32E-09
Anthropometry	Whole body fat free mass	T	372298	NA	NA	NA	0.055154	0.008368	0.038752	0.071555	4.37E-11	6.19E-09
Anthropometry	Whole body water mass	T	372328	NA	NA	NA	0.055014	0.008368	0.038611	0.071416	4.90E-11	6.54E-09
Respiratory	FEV1 never smoked only	T	109805	NA	NA	NA	0.10136	0.015436	0.071105	0.131615	5.16E-11	6.54E-09
Cardiovascular	Systolic blood pressure mean	T	357107	NA	NA	NA	-0.05519	0.008538	-0.07192	-0.03846	1.02E-10	1.23E-08
Metabolic	Other metabolic disorders (HES)	T	378957	309	378792	3.18	1.15769	0.182869	2.22	4.55	2.44E-10	2.80E-08
Respiratory	FEV1 never smoked only strict	T	84245	NA	NA	NA	0.11129	0.017603	0.076789	0.145791	2.58E-10	2.82E-08
Cardiovascular	Diastolic blood pressure mean	T	357110	NA	NA	NA	-0.05215	0.008539	-0.06889	-0.03541	1.01E-09	1.06E-07
Biological assays	Immature reticulocyte fraction	T	361630	NA	NA	NA	-0.05186	0.008503	-0.06852	-0.03519	1.07E-09	1.07E-07
Family history	Heart disease (family history - father)	T	343458	109130	234462	0.89	-0.11598	0.019175	0.858	0.925	1.46E-09	1.41E-07
Summary	Disorders of gallbladder biliary tract and pancreas (HES)	T	378957	16365	362736	1.25	0.220686	0.037509	1.16	1.34	4.02E-09	3.72E-07
Neurosciences	Headache	T	378087	81591	296640	1.12	0.116486	0.019992	1.08	1.17	5.66E-09	5.05E-07
Respiratory	FEV1 ever smoked only	T	175212	NA	NA	NA	0.069583	0.012235	0.045603	0.093562	1.29E-08	1.11E-06
Respiratory	FEV1 ever smoked only strict	T	136671	NA	NA	NA	0.07772	0.013884	0.050508	0.104932	2.17E-08	1.80E-06
Neurosciences	Headaches for 3 months vs no pain	T	330405	33891	296640	1.17	0.154784	0.02832	1.1	1.23	4.61E-08	3.71E-06
Digestive system	Bile duct disease (HES and self-reported)	T	378957	13856	365245	1.25	0.220425	0.040543	1.15	1.35	5.42E-08	4.21E-06
Genitourinary	Enlarged prostate (HES and self-reported)	T	378957	13062	366039	1.26	0.228012	0.043815	1.15	1.37	1.95E-07	1.47E-05
Musculoskeletal	Grip strength maximumValue	T	378178	NA	NA	NA	0.041545	0.008305	0.025266	0.057824	5.67E-07	4.14E-05
Digestive system	Cholelithiasis (HES)	T	378957	12988	366113	1.23	0.209588	0.041986	1.14	1.34	5.98E-07	4.24E-05
Anthropometry	Weight	T	377835	NA	NA	NA	0.04066	0.008326	0.02434	0.05698	1.04E-06	7.18E-05
Musculoskeletal	Osteoarthritis knee or hip	T	339483	27422	312194	0.845	-0.16799	0.03457	0.79	0.905	1.18E-06	7.88E-05
Family history	Heart disease (family history - mother)	T	357389	69487	288042	0.898	-0.10794	0.022461	0.859	0.938	1.54E-06	9.94E-05
Digestive system	Gall bladder disease (HES and self-reported)	T	378957	13958	365143	1.22	0.195992	0.040812	1.12	1.32	1.57E-06	9.94E-05
Operations and Procedures	Saphenous vein graft replacement of coronary artery	T	378957	3223	375878	0.582	-0.54089	0.116223	0.464	0.731	3.26E-06	0.000201
Summary	Arthrosis (HES)	T	378957	40307	338794	0.876	-0.13213	0.02855	0.829	0.927	3.69E-06	0.000217
Respiratory	Emphysema (HES)	T	378957	1571	377530	1.63	0.48683	0.105085	1.32	2	3.61E-06	0.000217
Operations and Procedures	Connection of thoracic artery to coronary artery	T	378957	3408	375693	0.607	-0.49996	0.110758	0.488	0.754	6.36E-06	0.000365
Cardiovascular	Chronic ischaemic heart disease (HES)	T	378957	22951	356150	0.842	-0.17163	0.038103	0.782	0.908	6.66E-06	0.000373
Respiratory	Emphysema (HES and self-reported)	T	378957	1662	377439	1.58	0.458595	0.103436	1.29	1.94	9.27E-06	0.000507
Musculoskeletal	Carpal tunnel syndrome (HES and self-reported)	T	378957	9101	370000	0.764	-0.26947	0.060982	0.678	0.861	9.92E-06	0.000531
Family history	Diabetes (family history - mother)	T	357389	32382	325147	0.871	-0.1384	0.031439	0.819	0.926	1.07E-05	0.000562
Musculoskeletal	Arthritis nos (HES and self-reported)	T	378957	13143	365958	0.804	-0.21859	0.05	0.729	0.886	1.23E-05	0.000631
Cardiovascular	Heartcardiac problem (HES and self-reported)	T	378957	25675	353426	0.855	-0.15622	0.035911	0.797	0.918	1.36E-05	0.000683
Musculoskeletal	BMD Combined	T	338046	NA	NA	NA	-0.03816	0.008784	-0.05537	-0.02094	1.40E-05	0.000689
Operations and Procedures	Release of entrapment of peripheral nerve at wrist	T	378957	8745	370356	0.765	-0.26734	0.06217	0.678	0.865	1.71E-05	0.000822
Family history	Chronic bronchitis emphysema (family history - mother)	T	357389	20553	336976	1.16	0.145529	0.0349	1.08	1.24	3.05E-05	0.00144
Medication	Drugs used in benign prostatic hypertrophy	T	378957	6797	372304	1.26	0.231243	0.057487	1.13	1.41	5.76E-05	0.002667
ENT	Tonsillectomy adenoids (self-reported)	T	378957	59321	319780	0.91	-0.09411	0.023631	0.869	0.953	6.82E-05	0.00304
Musculoskeletal	Osteoarthritis knee	T	330283	18217	312194	0.847	-0.16603	0.041654	0.781	0.919	6.72E-05	0.00304
Neurosciences	Migraine (HES and self-reported)	T	378957	12905	366196	1.18	0.168248	0.042774	1.09	1.29	8.38E-05	0.003669
Medication	Calcium	T	378957	6050	373051	1.26	0.232247	0.060435	1.12	1.42	0.000122	0.005208
Musculoskeletal	Osteoarthritis all	T	370576	58523	312194	0.911	-0.09284	0.024179	0.869	0.956	0.000123	0.005208
Operations and Procedures	Plastic operations on nose	T	378957	781	378320	1.74	0.553883	0.145181	1.31	2.31	0.000136	0.005653
Musculoskeletal	Osteoarthritis (HES and self-reported)	T	378957	58523	320578	0.914	-0.09013	0.024107	0.872	0.958	0.000185	0.007556
Summary	Ischaemic heart diseases (HES)	T	378957	27652	351449	0.88	-0.12728	0.034354	0.823	0.942	0.000211	0.00835
Genitourinary	Hyperplasia of prostate (HES)	T	378957	10244	368857	1.2	0.182831	0.049299	1.09	1.32	0.000208	0.00835
Musculoskeletal	Gonarthrosis (HES)	T	378957	18217	360884	0.859	-0.15248	0.041427	0.792	0.931	0.000233	0.009034
Operations and Procedures	Excision of gall bladder	T	378957	12079	367022	1.18	0.161564	0.044348	1.08	1.28	0.000269	0.010301
Neurosciences	Trapped nervecompressed nerve (HES and self-reported)	T	378957	15824	363277	0.851	-0.16133	0.044443	0.78	0.928	0.000283	0.010665
Anthropometry	Body mass index BMI	T	377691	NA	NA	NA	-0.03011	0.008325	-0.04643	-0.01379	0.000299	0.011072
Digestive system	Pancreatitis (HES and self-reported)	T	378957	2304	376797	1.4	0.333003	0.092432	1.16	1.67	0.000315	0.011496
Digestive system	Pancreatic disease (HES and self-reported)	T	378957	2413	376688	1.38	0.323164	0.090644	1.16	1.65	0.000364	0.013036
Musculoskeletal	Disc problem (HES and self-reported)	T	378957	8039	371062	0.797	-0.22742	0.063845	0.703	0.903	0.000368	0.013036
Operations and Procedures	Diagnostic percutaneous operations on liver	T	378957	1755	377346	1.45	0.370142	0.104367	1.18	1.78	0.00039	0.013432
Operations and Procedures	Endoscopic incision of sphincter of Oddi	T	378957	1801	377300	1.44	0.364936	0.102888	1.18	1.76	0.00039	0.013432
Family history	Chronic bronchitis emphysema (family history)	T	378957	NA	NA	NA	0.029214	0.008303	0.01294	0.045489	0.000434	0.014728
Metabolic	Disorders of lipoprotein metabolism and other lipidaemias (HES)	T	378957	32636	346465	0.896	-0.10962	0.031424	0.843	0.953	0.000486	0.016256
Summary	Diseases of male genital organs (HES)	T	378957	17819	361282	1.14	0.135365	0.039117	1.06	1.24	0.000539	0.017258
Operations and Procedures	Contrast radiology of heart	T	378957	21382	357719	0.875	-0.13354	0.038527	0.811	0.944	0.000528	0.017258
Respiratory	Spontaneous pneumothoraxrecurrent pneumothorax (HES and self-reported)	T	378957	1034	378067	1.58	0.454489	0.131436	1.22	2.04	0.000544	0.017258
Other	Sleeplessness insomnia	T	378457	NA	NA	NA	-0.02881	0.008317	-0.04511	-0.01251	0.000532	0.017258
Musculoskeletal	Other intervertebral disc disorders (HES)	T	378957	6634	372467	0.785	-0.24172	0.070648	0.684	0.902	0.000623	0.019484
NA	Myocardial infarction	T	378957	13488	365613	0.848	-0.16431	0.048807	0.771	0.934	0.000761	0.023504
Genitourinary	Bph benign prostatic hypertrophy (HES and self-reported)	T	378957	10618	368483	1.18	0.16393	0.04886	1.07	1.3	0.000793	0.024194
Digestive system	Liver failurecirrhosis (HES and self-reported)	T	378957	1525	377576	1.45	0.374481	0.112064	1.17	1.81	0.000833	0.025075
Musculoskeletal	Other arthritis (HES)	T	378957	10538	368563	0.834	-0.1812	0.054875	0.749	0.929	0.00096	0.028554
Cardiovascular	Angina pectoris (HES)	T	378957	16491	362610	0.866	-0.14425	0.043835	0.794	0.943	0.000999	0.029355
Cardiovascular	Pulse minimumValue	T	357110	NA	NA	NA	-0.02796	0.008539	-0.04469	-0.01122	0.00106	0.030777
Musculoskeletal	Osteoporosis (HES and self-reported)	T	378957	10107	368994	1.17	0.159544	0.048989	1.07	1.29	0.001127	0.032319
Medication	Drugs affecting bone structure and mineralization	T	378957	7218	371883	1.2	0.181067	0.056898	1.07	1.34	0.001461	0.041404
Medication	Beta blocking agents and other diuretics	T	378957	15555	363546	0.868	-0.1416	0.044758	0.795	0.948	0.001557	0.043123
Family history	Diabetes (family history - father)	T	343458	31628	311964	0.905	-0.09947	0.031424	0.851	0.963	0.001548	0.043123
Neurosciences	Mononeuropathies of upper limb (HES)	T	378957	9665	369436	0.836	-0.17927	0.056892	0.748	0.934	0.001627	0.044096
Gynaecology and Obstetrics	Age at menopause last menstrual period	T	116920	NA	NA	NA	0.046685	0.014818	0.017643	0.075728	0.001629	0.044096
Summary	Inflammatory polyarthropathies (HES)	T	378957	17341	361760	0.876	-0.13225	0.042244	0.807	0.952	0.001745	0.045326
Cancer	Mal neo breast (cancer register)	T	378957	10158	368943	0.839	-0.17511	0.055951	0.752	0.937	0.00175	0.045326
Summary	Mal neoplasm of breast (cancer register)	T	378957	10158	368943	0.839	-0.17511	0.055951	0.752	0.937	0.00175	0.045326
Operations and Procedures	Other division of bone	T	378957	1162	377939	1.48	0.393026	0.125388	1.16	1.89	0.001722	0.045326
Mental health	Neuroticism score	T	307154	NA	NA	NA	-0.02879	0.009216	-0.04685	-0.01073	0.001784	0.045714
Medication	Beta blocking agents and thiazides	T	378957	27196	351905	0.899	-0.10597	0.033968	0.842	0.961	0.001811	0.045923
Neurosciences	Peripheral nerve disorder (HES and self-reported)	T	378957	16736	362365	0.876	-0.1329	0.042732	0.805	0.952	0.00187	0.046934
Musculoskeletal	Tendonitis tendinitis tenosynovitis (HES and self-reported)	T	378957	5613	373488	0.789	-0.23754	0.076472	0.679	0.916	0.001895	0.04706
Cardiovascular	Angina (HES and self-reported)	T	378957	20423	358678	0.887	-0.12035	0.039335	0.821	0.958	0.002216	0.05447
Digestive system	Acute pancreatitis (HES)	T	378957	1763	377338	1.38	0.322185	0.105892	1.12	1.7	0.002346	0.056035
Symptoms	Abnormal results of function studies (HES)	T	378957	3857	375244	1.26	0.228606	0.075147	1.08	1.46	0.002349	0.056035
Gynaecology and Obstetrics	Caesarean section caesarian section (self-reported)	T	378957	19341	359760	0.884	-0.12358	0.040609	0.816	0.957	0.002341	0.056035
Anthropometry	Whole body fat mass	T	371710	NA	NA	NA	0.025445	0.008395	0.008991	0.0419	0.002438	0.057583
Family history	Chronic bronchitis emphysema (family history - sibling)	T	300905	8228	292801	1.18	0.162378	0.053773	1.06	1.31	0.00253	0.059181
Musculoskeletal	Synovitis and tenosynovitis (HES)	T	378957	4728	374373	0.778	-0.25128	0.08372	0.66	0.917	0.002688	0.060509
Cardiovascular	Heart attackmyocardial infarction (HES and self-reported)	T	378957	12068	367033	0.857	-0.15396	0.051236	0.775	0.948	0.002657	0.060509
Digestive system	Rectal or colon adenomapolyps (HES and self-reported)	T	378957	28246	350855	0.905	-0.10003	0.033271	0.848	0.966	0.002643	0.060509
Musculoskeletal	Osteoarthritis hip	T	322846	10780	312194	0.852	-0.16038	0.05341	0.767	0.946	0.002676	0.060509
Operations and Procedures	Endoscopic resection of outlet of male bladder	T	378957	3901	375200	1.25	0.225806	0.075427	1.08	1.45	0.002756	0.061475
Other	Relative age of first facial hair	T	168786	NA	NA	NA	0.037284	0.012477	0.01283	0.061738	0.002805	0.062004
Respiratory	FEV1 FVC ratio never smoked only strict	T	84245	NA	NA	NA	0.05256	0.017632	0.018002	0.087118	0.002873	0.062927
Musculoskeletal	Fracture shaft of femur (HES and self-reported)	T	378957	596	378505	1.64	0.497214	0.16797	1.18	2.29	0.003075	0.066736
Medication	Beta blocking agents	T	378957	27196	351905	0.905	-0.10022	0.033897	0.846	0.967	0.003112	0.066936
Summary	Disorders of sclera cornea iris and ciliary body (HES)	T	378957	1464	377637	1.4	0.338541	0.114709	1.12	1.76	0.003164	0.067453
Operations and Procedures	Total prosthetic replacement of knee joint using cement	T	378957	7800	371301	0.829	-0.18745	0.063572	0.732	0.939	0.003192	0.067453

Operations and Procedures	Colectomy hemicolectomy (self-reported)	T	378957	1636	377465	1.38	0.322367	0.109432	1.11	1.71	0.003221	0.067473
Other	Alcohol dependency (HES and self-reported)	T	378957	5185	373916	0.789	-0.2368	0.081294	0.673	0.925	0.003581	0.074372
Eye	Iridocyclitis (HES)	T	378957	333	378768	1.85	0.616403	0.212239	1.22	2.81	0.003681	0.075787
Mental health	Deliberate selfharmsuicide attempt (HES and self-reported)	T	378957	2635	376466	0.71	-0.34251	0.11833	0.563	0.895	0.003797	0.077525
Operations and Procedures	Attention to stent in organ NOC	T	378957	390	378711	1.78	0.576722	0.199804	1.2	2.63	0.003896	0.078877
Mental health	Mental and behavioural disorders due to use of alcohol (HES)	T	378957	4907	374194	0.786	-0.24022	0.083657	0.668	0.927	0.004086	0.082021
Cardiovascular	Acute myocardial infarction (HES)	T	378957	7305	371796	0.827	-0.19002	0.066251	0.726	0.942	0.004129	0.082198
Biological assays	Platelet count	T	367720	NA	NA	NA	0.024136	0.008434	0.007605	0.040667	0.004214	0.083207
Operations and Procedures	Prostate operation (self-reported)	T	378957	859	378242	1.51	0.412188	0.144266	1.14	2	0.004275	0.083721
Operations and Procedures	Other therapeutic on outlet of male bladder	T	378957	850	378251	1.52	0.418146	0.146554	1.14	2.02	0.004328	0.083785
Respiratory	FEV1 FVC ratio never smoked only	T	109805	NA	NA	NA	0.044085	0.015459	0.013786	0.074384	0.004347	0.083785
Summary	Nerve nerve root and plexus disorders (HES)	T	378957	16103	362998	0.884	-0.12322	0.043349	0.812	0.962	0.004477	0.085598
Infectious disease	Viral and other specified intestinal infections (HES)	T	378957	770	378331	1.54	0.432166	0.152369	1.14	2.08	0.004564	0.086571
Summary	Diseases of liver (HES)	T	378957	4603	374498	1.22	0.198064	0.069983	1.06	1.4	0.004652	0.08756
Immuno-inflammation	Sarcoidosis (HES)	T	378957	489	378612	1.68	0.517463	0.183154	1.17	2.4	0.004724	0.088215
Genitourinary	Prostate problem not cancer (HES and self-reported)	T	378957	11913	367188	1.14	0.132442	0.046957	1.04	1.25	0.004795	0.088855
Cancer	Myeloid leukaemia (cancer register)	T	378957	321	378780	1.84	0.608261	0.216737	1.2	2.81	0.005009	0.091416
Respiratory	Emphysemachronic bronchitis (HES and self-reported)	T	378957	6338	372763	1.19	0.170082	0.060587	1.05	1.33	0.004997	0.091416
Operations and Procedures	Approach to organ through other opening	T	378957	1441	377660	0.623	-0.47372	0.169005	0.447	0.867	0.005063	0.091559
Operations and Procedures	Radiology with contrast	T	378957	12447	366654	1.13	0.123777	0.044189	1.04	1.23	0.005093	0.091559
Musculoskeletal	Other inflammatory spondylopathies (HES)	T	378957	1156	377945	0.58	-0.54464	0.195	0.396	0.85	0.005222	0.093177
Gynaecology and Obstetrics	Breast cysts (HES and self-reported)	T	378957	1673	377428	1.36	0.30548	0.109599	1.09	1.68	0.005316	0.094158
Cancer	Prostate cancer (self-reported)	T	378957	2722	376379	0.733	-0.31025	0.11225	0.588	0.914	0.005711	0.100429
Summary	Spondylopathies (HES)	T	378957	10706	368395	0.863	-0.14751	0.053523	0.777	0.958	0.005851	0.102142
Infectious disease	Helicobacter pylori (HES)	T	378957	748	378353	0.476	-0.7432	0.269943	0.28	0.807	0.005902	0.102284
Other	Poisoning by nonopioid analgesics antipyretics and antirheumatics (HES)	T	378957	1567	377534	0.643	-0.44102	0.160457	0.47	0.881	0.005986	0.103009
Digestive system	Hepatic failure (HES)	T	378957	298	378803	1.85	0.616268	0.22666	1.19	2.89	0.00655	0.111113
Neurosciences	Headaches not migraine (self-reported)	T	378957	3224	375877	1.25	0.22324	0.082105	1.06	1.47	0.006549	0.111113
Medication	Beta blocking agents thiazides and other diuretics	T	378957	15312	363789	0.886	-0.1209	0.044693	0.812	0.967	0.006829	0.114845
Operations and Procedures	Therapeutic endoscopic operations on semilunar cartilage	T	378957	12492	366609	0.875	-0.13377	0.049482	0.794	0.964	0.006865	0.114845
Musculoskeletal	Coxarthrosis (HES)	T	378957	10780	368321	0.867	-0.14229	0.053153	0.782	0.963	0.007429	0.123432
Summary	Other dorsopathies (HES)	T	378957	18099	361002	0.896	-0.10932	0.040871	0.827	0.971	0.007481	0.123434
Cardiovascular	Pulmonary embolism dvt (HES and self-reported)	T	378957	5241	373860	1.19	0.175707	0.065818	1.05	1.36	0.007595	0.124459
Medication	Antacids	T	378957	6182	372919	1.18	0.163399	0.061433	1.04	1.33	0.007819	0.125585
ENT	Cholesteatoma of middle ear (HES)	T	378957	393	378708	1.72	0.540087	0.20323	1.15	2.56	0.007872	0.125585
Neurosciences	Knee pain	T	378087	86052	292179	0.948	-0.05371	0.020201	0.911	0.986	0.007846	0.125585
Cardiovascular	Venous thromboembolic disease (HES and self-reported)	T	378957	3983	375118	1.22	0.198302	0.074548	1.05	1.41	0.007812	0.125585
Musculoskeletal	Other spondylopathies (HES)	T	378957	3685	375416	0.778	-0.25048	0.094448	0.647	0.937	0.008	0.126784
Symptoms	Hepatomegaly and splenomegaly (HES)	T	378957	590	378511	1.58	0.455916	0.172351	1.13	2.21	0.008163	0.128519
Medication	All other therapeutic products	T	378957	3557	375544	1.23	0.20757	0.078707	1.05	1.44	0.008358	0.130413
Respiratory	FEV1 percent pred strict	T	221584	NA	NA	NA	0.028744	0.010905	0.007371	0.050117	0.008391	0.130413
Cardiovascular	Coronary artery bypass grafts cabg (self-reported)	T	378957	2799	376302	0.747	-0.29217	0.111191	0.6	0.928	0.008598	0.132773
Haematology	Diseases of spleen (HES)	T	378957	463	378638	0.368	-0.99927	0.381005	0.174	0.777	0.008723	0.133208
Digestive system	Stomach disorder (HES and self-reported)	T	378957	7348	371753	1.16	0.148799	0.056746	1.04	1.3	0.008737	0.133208
Genitourinary	Other disorders of bladder (HES)	T	378957	8086	371015	1.15	0.141738	0.054301	1.04	1.28	0.009048	0.137093
Genitourinary	Recurrent and persistent haematuria (HES)	T	378957	660	378441	1.54	0.430854	0.165436	1.11	2.13	0.009205	0.137727
Digestive system	Liverbiliarypancreas problem (HES and self-reported)	T	378957	4283	374818	1.21	0.189746	0.072814	1.05	1.39	0.009163	0.137727
Other	Toxic effect of alcohol (HES)	T	378957	912	378189	0.556	-0.58738	0.226365	0.357	0.866	0.009463	0.139857
Operations and Procedures	Primary decompression operations on lumbar spine	T	378957	2461	376640	0.735	-0.30743	0.118402	0.583	0.927	0.009419	0.139857
Genitourinary	Urinary tract infectionkidney infection (HES and self-reported)	T	378957	12655	366446	1.12	0.114096	0.044252	1.03	1.22	0.009928	0.145834
Summary	Certain disorders involving the immune mechanism (HES)	T	378957	976	378125	1.43	0.35696	0.139142	1.09	1.88	0.010305	0.146888
Other	Poisoning by psychotropic drugs (HES)	T	378957	1062	378039	0.594	-0.52085	0.20269	0.399	0.884	0.010179	0.146888
Operations and Procedures	Total prosthetic replacement of hip joint not using cement	T	378957	4094	375007	0.797	-0.22684	0.088399	0.67	0.948	0.010285	0.146888
NA	STEMI	T	378957	3811	375290	0.787	-0.24015	0.093535	0.655	0.945	0.010245	0.146888
Family history	Stroke (family history)	T	378957	NA	NA	NA	-0.02131	0.008301	-0.03758	-0.00504	0.010256	0.146888
Digestive system	Other diseases of stomach and duodenum (HES)	T	378957	6601	372500	1.16	0.152702	0.05963	1.04	1.31	0.010442	0.147516
Cancer	Mal neo liver (cancer register)	T	378957	217	378884	1.95	0.665385	0.260136	1.17	3.24	0.010533	0.147516
Cardiovascular	Leg pain on walking	T	125352	27294	98122	0.912	-0.09161	0.035811	0.851	0.979	0.010522	0.147516
Genitourinary	Iga nephropathy (HES and self-reported)	T	378957	666	378435	1.52	0.421179	0.165388	1.1	2.11	0.010878	0.151469
Summary	Diabetes mellitus (HES)	T	378957	17982	361119	0.901	-0.1043	0.04105	0.831	0.976	0.011059	0.153109
Operations and Procedures	Replacement of hip joint	T	378957	1139	377962	1.38	0.325503	0.128284	1.08	1.78	0.011169	0.153753
Metabolic	Unspecified diabetes mellitus (HES)	T	378957	2120	376981	0.721	-0.32702	0.129313	0.56	0.929	0.011443	0.156626
Operations and Procedures	Other operations on sheath of tendon	T	378957	2908	376193	0.764	-0.26938	0.107477	0.619	0.943	0.012197	0.166009
Eye	Cataract (HES and self-reported)	T	378957	20791	358310	1.09	0.089131	0.03581	1.02	1.17	0.012809	0.173353
Symptoms	Ascites (HES)	T	378957	1446	377655	1.34	0.293798	0.118234	1.06	1.69	0.01296	0.174413
Summary	HESCH Diseases of the musculo and connec tissue (HES)	T	378957	100763	278338	0.953	-0.04772	0.019242	0.918	0.99	0.013133	0.175476
Operations and Procedures	Extracorporeal fragmentation of calculus of kidney	T	378957	1421	377680	0.665	-0.40837	0.164748	0.481	0.918	0.013184	0.175476
Family history	Diabetes (family history - sibling)	T	300905	24485	276544	0.916	-0.0876	0.03539	0.855	0.982	0.013313	0.176218
Cardiovascular	Mean carotid IMT intima medial thickness at 120 degrees	T	1871	NA	NA	NA	0.309672	0.125236	0.064209	0.555135	0.013409	0.176514
Summary	Disorders of lens (HES)	T	378957	18482	360619	1.1	0.092862	0.037748	1.02	1.18	0.013892	0.181873
Cardiovascular	Other peripheral vascular diseases (HES)	T	378957	3304	375797	0.782	-0.24591	0.100658	0.642	0.953	0.014563	0.188614
Summary	Mal neo lymphoid haematopoietic (cancer register)	T	378957	3145	375956	1.23	0.204025	0.083455	1.04	1.44	0.014496	0.188614
Summary	Mood affective disorders (HES)	T	378957	11549	367552	0.883	-0.12471	0.051154	0.799	0.976	0.014768	0.19025
Summary	Other diseases of urinary system (HES)	T	378957	26755	352346	1.08	0.077108	0.031658	1.02	1.15	0.014867	0.190498
Musculoskeletal	Joint pain (HES and self-reported)	T	378957	8344	370757	0.863	-0.14717	0.060491	0.767	0.972	0.014978	0.190915
Cardiovascular	Atherosclerosis (HES)	T	378957	1127	377974	0.633	-0.45744	0.188545	0.437	0.916	0.015259	0.192458
Family history	Parkinson s disease (family history)	T	378957	NA	NA	NA	-0.02011	0.008287	-0.03635	-0.00387	0.015226	0.192458
Haematology	Iron deficiency anaemia (HES)	T	378957	6915	372186	0.851	-0.16149	0.066823	0.746	0.97	0.01566	0.196488
Eye	Intra ocular pressure corneal compensated left	T	81021	NA	NA	NA	-0.0429	0.017772	-0.07774	-0.00807	0.015777	0.196928
Neurosciences	Myasthenia gravis (HES and self-reported)	T	378957	206	378895	1.91	0.647126	0.268443	1.13	3.23	0.015924	0.197732
Summary	Other forms of heart disease (HES)	T	378957	26042	353059	0.921	-0.0827	0.034399	0.861	0.985	0.016213	0.198234
Digestive system	Other diseases of liver (HES)	T	378957	3122	375979	1.22	0.202172	0.084445	1.04	1.44	0.01666	0.198234
Immuno-inflammation	Nail disorders (HES)	T	378957	1050	378051	1.39	0.329649	0.137679	1.06	1.82	0.016651	0.198234
Symptoms	Abnormal findings on diagnostic imaging of CNS (HES)	T	378957	319	378782	1.72	0.542688	0.226057	1.1	2.68	0.016365	0.198234
Medication	Antimigraine preparations	T	378957	5449	373652	1.17	0.155235	0.065057	1.03	1.33	0.017026	0.198234
Operations and Procedures	Spinal fusion (self-reported)	T	378957	517	378584	0.465	-0.76496	0.319176	0.249	0.87	0.016545	0.198234
Cancer	Mal neo prostate (cancer register)	T	378957	6546	372555	0.848	-0.16475	0.069108	0.741	0.971	0.017129	0.198234
Operations and Procedures	High cost musculoskeletal drugs	T	378957	570	378531	1.53	0.424618	0.177153	1.08	2.16	0.016535	0.198234
Medication	Vitamin D	T	376892	14603	362433	1.1	0.099116	0.041518	1.02	1.2	0.016973	0.198234
Genitourinary	Bladder problem not cancer (HES and self-reported)	T	378957	10748	368353	1.12	0.114371	0.047884	1.02	1.23	0.016917	0.198234
Neurosciences	Bells palsyfacial nerve palsy (HES and self-reported)	T	378957	922	378179	1.41	0.343988	0.144385	1.06	1.87	0.017198	0.198234
Immuno-inflammation	Sarcoidosis (HES and self-reported)	T	378957	930	378171	1.41	0.342189	0.143042	1.06	1.86	0.016746	0.198234
Musculoskeletal	Soft tissue inflammation (HES and self-reported)	T	378957	9317	369784	0.873	-0.13575	0.056962	0.781	0.976	0.017169	0.198234
Family history	Alzheimer s disease dementia (family history)	T	378957	NA	NA	NA	-0.01979	0.008292	-0.03604	-0.00354	0.017008	0.198234
Anthropometry	Gynoid tissue fat percentage	T	4100	NA	NA	NA	0.20597	0.086266	0.036888	0.375052	0.016959	0.198234
Anthropometry	Legs tissue fat percentage	T	4100	NA	NA	NA	0.204904	0.086174	0.036004	0.373804	0.017416	0.199788
Haematology	Iron deficiency anaemia (HES and self-reported)	T	378957	8597	370504	0.868	-0.14108	0.059515	0.773	0.976	0.017766	0.20284
Digestive system	Cholecystitis (HES and self-reported)	T	378957	2791	376310	1.23	0.209819	0.088707	1.04	1.47	0.018015	0.204706
Gynaecology and Obstetrics	Bilateral oophorectomy both ovaries removed	T	201515	16364	185221	0.902	-0.10285	0.043532	0.828	0.983	0.018144	0.205208
Digestive system	Gastro oesophageal reflux disease (HES)	T	378957	20891	358210	0.915	-0.08923	0.037892	0.849	0.985	0.01853	0.207617
Eye	Intra ocular pressure Goldmann correlated left	T	81021	NA	NA	NA	-0.04188	0.017777	-0.07672	-0.00704	0.018482	0.207617
Eye	Other eye problems	T	377814	54312	323646	1.06	0.055017	0.02339	1.01	1.11	0.018666	0.208174
Operations and Procedures	Small bowel resection (self-reported)	T	378957	657	378444	1.48	0.392804	0.167423	1.07	2.06	0.018967	0.20894
Operations and Procedures	Removal of other inorganic substance from skin	T	378957	431	378670	0.409	-0.89504	0.381217	0.194	0.863	0.018882	0.20894
Cardiovascular	Peripheral vascular disease (HES and self-reported)	T	378957	1859	377242	0.723	-0.32479	0.138463	0.551	0.948	0.018995	0.20894
Cancer	Ovarian cancer (self-reported)	T	378957	645	378456	0.531	-0.63302	0.270532	0.312	0.902	0.019289	0.211212
Other	Birth weight	T	215451	NA	NA	NA	-0.02573	0.011009	-0.04731	-0.00415	0.019431	0.211811
Operations and Procedures	Spinal laminectomy (self-reported)	T	378957	3219	375882	0.79	-0.23587	0.101027	0.648	0.963	0.019556	0.212056
Operations and Procedures	Other operations on ligament	T	378957	254	378847	0.191	-1.65652	0.709931	0.0475	0.767	0.01963	0.212056
Operations and Procedures	Hip replacement revision (self-reported)	T	378957	6037	373064	0.847	-0.16605	0.071231	0.737	0.974	0.019749	0.212392
Genitourinary	Endometriosis (HES)	T	378957	3557	375544	0.801	-0.22183	0.09541	0.664	0.966	0.020072	0.214905
Other	Relative age voice broke	T	161554	NA	NA	NA	0.029584	0.012744	0.004607	0.054562	0.02026	0.215954
Respiratory	FEV1 percent pred	T	285833	NA	NA	NA	0.022225	0.009591	0.003427	0.041022	0.020485	0.217395
Cardiovascular	Other aneurysm (HES)	T	378957	436	378665	1.59	0.460625	0.19908	1.07	2.34	0.020681	0.21781
Biological assays	Red blood cell erythrocyte distribution width	T	367720	NA	NA	NA	0.019522	0.008439	0.002982	0.036062	0.020705	0.21781
Operations and Procedures	Other therapeutic operations on bile duct	T	378957	284	378817	1.73	0.548452	0.237808	1.09	2.76	0.021095	0.219992
Haematology	Hereditarygenetic haematological disorder (HES and self-reported)	T	378957	463	378638	1.57	0.450648	0.195274	1.07	2.3	0.021012	0.219992

Medication	Beta blocking agents other combinations	T	378957	42301	336800	0.939	-0.0634	0.027583	0.889	0.991	0.021524	0.222119
Musculoskeletal	Spondylosis (HES)	T	378957	6891	372210	0.858	-0.15309	0.066639	0.753	0.978	0.021599	0.222119
Operations and Procedures	Total excision of breast	T	378957	4061	375040	0.816	-0.20366	0.08859	0.686	0.97	0.021512	0.222119
Musculoskeletal	Cervical spondylosis (HES and self-reported)	T	378957	4865	374236	0.831	-0.18517	0.080702	0.709	0.973	0.02176	0.222119
ENT	Vertigo (HES and self-reported)	T	378957	697	378404	1.45	0.374627	0.163201	1.06	2	0.021705	0.222119
Mental health	Depressive episode (HES)	T	378957	10665	368436	0.886	-0.12115	0.053093	0.798	0.983	0.022499	0.224897
Operations and Procedures	Other operations on female perineum	T	378957	887	378214	1.4	0.336563	0.147478	1.05	1.87	0.022482	0.224897
Operations and Procedures	Suture of organ NOC	T	378957	640	378461	1.47	0.387486	0.169668	1.06	2.05	0.022383	0.224897
Cardiovascular	Aortic stenosis (HES and self-reported)	T	378957	1728	377373	0.722	-0.326	0.142641	0.546	0.955	0.022287	0.224897
Family history	Diabetes (family history)	T	378957	NA	NA	NA	-0.01898	0.008298	-0.03524	-0.00271	0.022191	0.224897
Summary	Disorders of psychological development (HES)	T	378957	287	378814	1.72	0.540326	0.237718	1.08	2.74	0.023028	0.225361
Eye	Other disorders of cornea (HES)	T	378957	582	378519	1.5	0.403179	0.177055	1.06	2.12	0.022778	0.225361
Digestive system	Other diseases of anus and rectum (HES)	T	378957	21218	357883	0.918	-0.08547	0.037626	0.853	0.988	0.023107	0.225361
Cardiovascular	Cardiac ablation (self-reported)	T	378957	269	378832	0.266	-1.32307	0.580736	0.0853	0.831	0.022711	0.225361
Digestive system	Bile duct obstructionascending cholangitis (HES and self-reported)	T	378957	936	378165	1.39	0.327967	0.144347	1.05	1.84	0.023082	0.225361
Musculoskeletal	Spinal stenosis (HES and self-reported)	T	378957	3421	375680	0.803	-0.21975	0.096561	0.664	0.97	0.022864	0.225361
Musculoskeletal	Osteoporosis without pathological fracture (HES)	T	378957	5396	373705	1.16	0.149848	0.066234	1.02	1.32	0.023672	0.227967
Neurosciences	Knee pain for 3 months vs no pain	T	354567	62522	292179	0.949	-0.05188	0.022958	0.908	0.993	0.023847	0.227967
Operations and Procedures	Surgical arrest of bleeding from internal nose	T	378957	782	378319	1.42	0.352475	0.155576	1.05	1.93	0.023475	0.227967
Cardiovascular	Deep venous thrombosis dvt (HES and self-reported)	T	378957	8917	370184	1.12	0.117595	0.052031	1.02	1.25	0.023816	0.227967
Musculoskeletal	Disc degeneration (HES and self-reported)	T	378957	2914	376187	0.787	-0.23923	0.105768	0.64	0.969	0.023707	0.227967
Digestive system	Fibrosis and cirrhosis of liver (HES)	T	378957	756	378345	1.43	0.358405	0.159121	1.05	1.95	0.024296	0.231344
Operations and Procedures	Transurethral resection bladder tumour turbt (self-reported)	T	378957	458	378643	1.55	0.439353	0.195514	1.06	2.28	0.024629	0.233144
Musculoskeletal	Back pain (HES and self-reported)	T	378957	14806	364295	0.904	-0.10073	0.04484	0.828	0.987	0.024679	0.233144
Cancer	Mal neo brain (cancer register)	T	378957	407	378694	1.59	0.463518	0.206728	1.06	2.38	0.024951	0.234792
Family history	Heart disease (family history - sibling)	T	300905	29868	271161	0.93	-0.07256	0.03257	0.872	0.991	0.025883	0.241679
Operations and Procedures	Transluminal operations on varicose vein of leg	T	378957	1198	377903	1.34	0.291341	0.130754	1.04	1.73	0.02587	0.241679
Operations and Procedures	Carpal tunnel surgery (self-reported)	T	378957	3953	375148	0.821	-0.19666	0.088723	0.69	0.977	0.026653	0.24229
Other	Hair balding pattern	T	173207	117813	55466	0.943	-0.0587	0.026445	0.895	0.993	0.026443	0.24229
Other	Pattern 1 f	T	173207	55466	117813	1.06	0.058699	0.026445	1.01	1.12	0.026443	0.24229
Other	Pattern 2 f	T	173207	39959	133320	0.935	-0.06699	0.030127	0.882	0.992	0.026173	0.24229
Operations and Procedures	Punch biopsy of skin	T	378957	3326	375775	1.2	0.182491	0.082309	1.02	1.41	0.026612	0.24229
Anthropometry	Hip circumference	T	378245	NA	NA	NA	0.018466	0.008323	0.002154	0.034778	0.026501	0.24229
Eye	Intra ocular pressure Goldmann correlated right	T	81141	NA	NA	NA	-0.0394	0.017737	-0.07416	-0.00464	0.02632	0.24229
Eye	Cataract	T	124323	11723	112653	1.11	0.106854	0.048289	1.01	1.22	0.026911	0.243718
Operations and Procedures	Introduction of abortifacient into uterine cavity	T	378957	623	378478	0.55	-0.59784	0.27171	0.323	0.937	0.027787	0.250711
Summary	Other disorders of kidney and ureter (HES)	T	378957	2376	376725	1.23	0.210246	0.095713	1.02	1.49	0.028047	0.251173
Operations and Procedures	Other local flap of skin	T	378957	1956	377145	1.26	0.229887	0.104639	1.03	1.54	0.028024	0.251173
Musculoskeletal	Spine arthritisspondylitis (HES and self-reported)	T	378957	10404	368697	0.889	-0.118	0.053765	0.8	0.987	0.02818	0.251316
Musculoskeletal	Falls in the last year	T	378025	NA	NA	NA	-0.01827	0.008331	-0.0346	-0.00195	0.028272	0.251316
Cardiovascular	Mitral valve prolapse (HES and self-reported)	T	378957	545	378556	1.49	0.400175	0.182563	1.04	2.13	0.028381	0.251361
Operations and Procedures	Block dissection of lymph nodes	T	378957	5157	373944	0.844	-0.16956	0.077553	0.725	0.983	0.028788	0.254026
Summary	Other diseases of intestines (HES)	T	378957	59649	319452	0.95	-0.05102	0.023423	0.908	0.995	0.029384	0.255654
Summary	Disorders of bone density and structure (HES)	T	378957	8188	370913	1.13	0.118836	0.05463	1.01	1.25	0.029609	0.255654
Digestive system	Other diseases of pancreas (HES)	T	378957	1002	378099	1.36	0.308307	0.141497	1.03	1.8	0.02934	0.255654
Genitourinary	Other disorders of kidney and ureter (HES)	T	378957	2178	376923	1.24	0.216907	0.099643	1.02	1.51	0.029492	0.255654
Operations and Procedures	Approach to organ under image control	T	378957	45067	334034	0.944	-0.05755	0.026446	0.896	0.994	0.029543	0.255654
Musculoskeletal	Sjogrens syndromesicca syndrome (HES and self-reported)	T	378957	614	378487	1.46	0.379649	0.174507	1.04	2.06	0.029589	0.255654
Musculoskeletal	Raynauds phenomenondisease (HES and self-reported)	T	378957	1315	377786	0.696	-0.3631	0.167047	0.501	0.965	0.029731	0.255793
Summary	Mal neo of male genital organs (cancer register)	T	378957	7046	372055	0.867	-0.14328	0.066141	0.761	0.986	0.030286	0.259644
Summary	Metabolic disorders (HES)	T	378957	39911	339190	0.941	-0.0609	0.028231	0.89	0.994	0.030983	0.260061
Metabolic	Non insulin dependent diabetes mellitus (HES)	T	378957	16601	362500	0.912	-0.0917	0.04244	0.84	0.992	0.030726	0.260061
Genitourinary	Other disorders of urinary system (HES)	T	378957	17515	361586	1.09	0.082911	0.038428	1.01	1.17	0.030962	0.260061
Operations and Procedures	Other excision of breast	T	378957	9523	369578	0.885	-0.12198	0.056461	0.792	0.989	0.030739	0.260061
Family history	Prostate cancer (family history)	T	174646	NA	NA	NA	0.026453	0.012256	0.002431	0.050474	0.030902	0.260061
Biological assays	Mean platelet thrombocyte volume	T	367716	NA	NA	NA	-0.01821	0.00844	-0.03476	-0.00167	0.030918	0.260061
Symptoms	Syncope and collapse (HES)	T	378957	8267	370834	0.878	-0.1304	0.060488	0.78	0.988	0.031102	0.260151
Biological assays	Monocyte count	T	367067	NA	NA	NA	0.01818	0.008444	0.001631	0.034729	0.031309	0.260984
Operations and Procedures	Endoscopic extirpation of lesion of colon	T	378957	14469	364632	0.907	-0.09769	0.045583	0.829	0.992	0.032108	0.266718
Metabolic	Type 2 diabetes (HES and self-reported)	T	378957	17351	361750	0.915	-0.08892	0.04152	0.843	0.992	0.032223	0.26675
Other	DIED	T	378957	10919	368182	1.11	0.102585	0.047948	1.01	1.22	0.032395	0.267262
Cardiovascular	Triple heart bypass (self-reported)	T	378957	526	378575	0.536	-0.62438	0.292561	0.302	0.95	0.032827	0.268929
Operations and Procedures	Unilateral excision of adnexa of uterus	T	378957	1862	377239	0.748	-0.29084	0.136358	0.572	0.977	0.032932	0.268929
Cardiovascular	Heart arrhythmia (HES and self-reported)	T	378957	21190	357911	0.923	-0.08051	0.037736	0.857	0.993	0.032883	0.268929
Summary	Abnormal findings on diagnostic imaging (HES)	T	378957	8914	370187	1.12	0.111812	0.052469	1.01	1.24	0.033088	0.269283
Operations and Procedures	Total excision of spleen	T	378957	281	378820	0.344	-1.06733	0.503383	0.128	0.922	0.033979	0.275604
Gynaecology and Obstetrics	Bilateral oophorectomy (self-reported)	T	378957	15863	363238	0.911	-0.09274	0.04393	0.836	0.993	0.034768	0.278705
Operations and Procedures	Therapeutic transluminal operations on vein	T	378957	314	378787	1.63	0.488236	0.231382	1.04	2.56	0.034851	0.278705
Operations and Procedures	Facilitating operations NOC	T	378957	2533	376568	0.788	-0.23857	0.112906	0.631	0.983	0.034603	0.278705
Medication	Calcium	T	377706	25583	352267	1.07	0.068979	0.032737	1	1.14	0.035111	0.278705
Digestive system	Infectiveviral hepatitis (HES and self-reported)	T	378957	1240	377861	0.692	-0.36754	0.174109	0.492	0.974	0.034777	0.278705
Musculoskeletal	Polymyalgia rheumatica (HES and self-reported)	T	378957	1540	377561	1.28	0.244822	0.116152	1.02	1.6	0.03505	0.278705
Respiratory	CopdExacerbation NI HES SPIROMETRY	T	25076	2309	22801	1.25	0.219775	0.104337	1.02	1.53	0.035171	0.278705
Medication	Antispasmodics in combination with analgesics	T	378957	607	378494	1.45	0.371449	0.176832	1.03	2.05	0.035679	0.280781
Medication	Tetracyclines	T	378957	2224	376877	1.23	0.209763	0.099933	1.01	1.5	0.035814	0.280781
Operations and Procedures	Cruciate ligament surgery (self-reported)	T	378957	1888	377213	0.752	-0.28546	0.13606	0.576	0.981	0.035899	0.280781
Operations and Procedures	Other open operations on peritoneum	T	378957	2184	376917	1.23	0.209911	0.100011	1.01	1.5	0.035828	0.280781
Digestive system	Other disorders of peritoneum (HES)	T	378957	2824	376277	1.2	0.186437	0.088968	1.01	1.43	0.036123	0.281615
Operations and Procedures	Prosthesis of lens	T	378957	17908	361193	1.08	0.080538	0.038485	1.01	1.17	0.036373	0.282652
Summary	Diseases of myoneural junction and muscle (HES)	T	378957	482	378619	1.5	0.407278	0.194996	1.03	2.2	0.03674	0.283673
Summary	Mal neo of central nervous system (cancer register)	T	378957	562	378539	1.46	0.380876	0.182333	1.02	2.09	0.036716	0.283673
Medication	Multivitamins combinations	T	378957	19000	360101	0.921	-0.08274	0.039692	0.852	0.995	0.037103	0.284651
Medication	High ceiling diuretics	T	378957	3930	375171	0.83	-0.18668	0.089505	0.696	0.989	0.03701	0.284651
Neurosciences	Hip pain for 3 months vs no pain	T	362543	32691	329991	0.938	-0.06351	0.030546	0.884	0.996	0.037616	0.287671
Operations and Procedures	Transluminal operations on aorta	T	378957	858	378243	0.638	-0.44898	0.216353	0.418	0.975	0.037965	0.28942
Operations and Procedures	Other destruction of lesion of skin of other site	T	378957	282	378819	1.66	0.505731	0.243893	1.03	2.67	0.038119	0.289623
Other	Overall acceleration average	T	81434	NA	NA	NA	-0.0371	0.017905	-0.0722	-0.00201	0.038232	0.289623
Gynaecology and Obstetrics	Termination of pregnancy top (self-reported)	T	378957	945	378156	0.662	-0.41211	0.199295	0.448	0.979	0.038655	0.290997
Biological assays	Mean sphered cell volume	T	361631	NA	NA	NA	0.017602	0.008508	0.000927	0.034278	0.038551	0.290997
Operations and Procedures	Other excision of appendix	T	378957	1270	377831	1.3	0.263393	0.127678	1.01	1.67	0.039117	0.29191
Operations and Procedures	Transluminal heart assist operations	T	378957	275	378826	0.354	-1.03936	0.503881	0.132	0.95	0.039139	0.29191
Operations and Procedures	Urethral catheterisation of bladder	T	378957	10309	368792	1.11	0.101043	0.048978	1.01	1.22	0.039108	0.29191
Mental health	Seen doctor GP for nerves anxiety tension or depression	T	376297	129804	246635	0.964	-0.03689	0.017904	0.931	0.998	0.039344	0.292528
Summary	Diseases of peritoneum (HES)	T	378957	3415	375686	1.18	0.167937	0.081699	1.01	1.39	0.039825	0.293388
Operations and Procedures	Endoscopic bilateral occlusion of fallopian tubes	T	378957	2687	376414	0.794	-0.23019	0.111969	0.638	0.989	0.039797	0.293388
Infectious disease	Septicaemia sepsis (HES and self-reported)	T	378957	3840	375261	1.17	0.159065	0.077335	1.01	1.36	0.039704	0.293388
Eye	Senile cataract (HES)	T	378957	7529	371572	1.12	0.116491	0.056776	1.01	1.26	0.040192	0.294293
Genitourinary	Other inflammation of vagina and vulva (HES)	T	378957	690	378411	0.604	-0.50478	0.245896	0.373	0.977	0.040089	0.294293
Operations and Procedures	Cystectomy (self-reported)	T	378957	1986	377115	1.24	0.213906	0.104495	1.01	1.52	0.040654	0.296776
Summary	Other disorders of the genitourinary system (HES)	T	378957	1063	378038	1.32	0.280904	0.137702	1.01	1.73	0.041356	0.300079
Genitourinary	Postprocedural disorders of genitourinary system (HES)	T	378957	1063	378038	1.32	0.280904	0.137702	1.01	1.73	0.041356	0.300079
Operations and Procedures	Levels of spine	T	378957	6828	372273	0.874	-0.13465	0.066165	0.768	0.995	0.041849	0.302742
Summary	Symptoms and signs involving the urinary system (HES)	T	378957	26525	352576	1.07	0.06513	0.032041	1	1.14	0.042079	0.303499
Operations and Procedures	High cost immunosuppressant drugs	T	378957	602	378499	1.43	0.359066	0.176863	1.01	2.03	0.042337	0.304445
Cardiovascular	Aortic aneurysm repair or stent (self-reported)	T	378957	277	378824	1.64	0.494059	0.244272	1.02	2.65	0.043117	0.309133
Respiratory	Vasomotor and allergic rhinitis (HES)	T	378957	952	378149	1.34	0.293908	0.145605	1.01	1.78	0.043537	0.310313
Operations and Procedures	Skin graft (self-reported)	T	378957	649	378452	0.6	-0.51085	0.253326	0.365	0.986	0.043738	0.310313
Operations and Procedures	Extracapsular extraction of lens	T	378957	17912	361189	1.08	0.077685	0.038534	1	1.17	0.043797	0.310313
Operations and Procedures	Partial excision of liver	T	378957	334	378767	0.435	-0.83181	0.412102	0.194	0.976	0.043543	0.310313
Mental health	Dementia in Alzheimer s disease (HES)	T	378957	223	378878	1.71	0.539279	0.267855	1.01	2.9	0.044081	0.31141
Musculoskeletal	Gout (HES)	T	378957	2920	376181	0.81	-0.21073	0.105059	0.659	0.995	0.04487	0.314024
Musculoskeletal	Other arthrosis (HES)	T	378957	14991	364110	0.915	-0.08909	0.044409	0.839	0.998	0.04485	0.314024
Gynaecology and Obstetrics	Ectopic pregnancy surgery (self-reported)	T	378957	2218	376883	0.784	-0.24293	0.121215	0.618	0.995	0.04506	0.314024
Operations and Procedures	Body region	T	378957	6404	372697	1.13	0.122315	0.060908	1	1.27	0.044622	0.314024
Biological assays	Platelet crit	T	367717	NA	NA	NA	0.016898	0.008433	0.000369	0.033428	0.045103	0.314024
Operations and Procedures	Diagnostic puncture of bone	T	378957	3796	375305	1.17	0.155749	0.077781	1	1.36	0.04524	0.31407
Summary	Diseases of oesophagus stomach and duodenum (HES)	T	378957	55390	323711	0.953	-0.04828	0.024141	0.909	0.999	0.045509	0.314127

Symptoms	Other abnormal immunological findings in serum (HES)	T	378957	247	378854	1.68	0.518015	0.258987	1.01	2.79	0.045484	0.314127
Summary	Number of self reported non cancer illnesses	T	378896	NA	NA	NA	0.016614	0.008313	0.000321	0.032906	0.045649	0.314195
Summary	HESCH Mental and behavioural disorders (HES)	T	378957	29106	349995	0.938	-0.06429	0.032198	0.88	0.999	0.045873	0.314835
Operations and Procedures	Diagnostic endoscopic examination of ileum	T	378957	590	378511	0.583	-0.53956	0.270639	0.343	0.991	0.046189	0.315816
Operations and Procedures	Other operations on cervix uteri	T	378957	247	378854	1.68	0.516008	0.258929	1.01	2.78	0.046278	0.315816
Operations and Procedures	Endoscopic ultrasound examination of bile duct	T	378957	228	378873	1.7	0.532476	0.267443	1.01	2.88	0.046482	0.31592
Other	Townsend deprivation index at recruitment	T	378505	NA	NA	NA	-0.01646	0.008272	-0.03268	-0.00025	0.046555	0.31592
NA	NSTEMI	T	378957	4419	374682	0.847	-0.16653	0.083841	0.718	0.998	0.047005	0.316995
Cardiovascular	Leg claudication intermittent claudication (HES and self-reported)	T	378957	2468	376633	0.795	-0.22976	0.115729	0.633	0.997	0.047109	0.316995
Haematology	Anaemia (HES and self-reported)	T	378957	17268	361833	0.921	-0.08214	0.04135	0.849	0.999	0.046992	0.316995
Medication	Corticosteroids	T	378957	4111	374990	1.16	0.14828	0.075021	1	1.34	0.048096	0.319816
Operations and Procedures	Reconstruction of femoral artery	T	378957	231	378870	0.316	-1.15115	0.581343	0.101	0.988	0.047686	0.319816
Operations and Procedures	Plastic operations on penis	T	378957	394	378707	1.53	0.425779	0.215355	1	2.33	0.04803	0.319816
Digestive system	Anal problem (HES and self-reported)	T	378957	23889	355212	0.933	-0.06981	0.035355	0.87	0.999	0.048324	0.319816
Digestive system	Haemochromatosis (HES and self-reported)	T	378957	635	378466	1.42	0.349445	0.176659	1	2.01	0.04792	0.319816
Musculoskeletal	Fracture tibia (HES and self-reported)	T	378957	1942	377159	1.23	0.209621	0.106119	1	1.52	0.04823	0.319816
Digestive system	Other diseases of biliary tract (HES)	T	378957	1752	377349	1.24	0.218904	0.110992	1	1.55	0.048582	0.319816
Medication	Dopaminergic agents	T	378957	1188	377913	0.71	-0.34296	0.174283	0.504	0.999	0.04909	0.32023
Neurosciences	Back pain for 3 months vs no pain	T	341405	65312	276219	0.957	-0.04442	0.022597	0.915	1	0.049327	0.32023
Cancer	Mal neo pancreas (cancer register)	T	378957	463	378638	1.48	0.389615	0.19868	1	2.18	0.049877	0.32023
Operations and Procedures	Connection between lacrimal apparatus and nose	T	378957	711	378390	0.626	-0.46857	0.238969	0.392	1	0.049901	0.32023
Operations and Procedures	Transluminal operations on femoral artery	T	378957	1076	378025	0.695	-0.36381	0.185607	0.483	1	0.049982	0.32023
Operations and Procedures	Diagnostic imaging procedures	T	378957	15463	363638	1.08	0.079648	0.040625	1	1.17	0.049928	0.32023
Infectious disease	Whooping cough pertussis (HES and self-reported)	T	378957	415	378686	1.5	0.405456	0.206649	1	2.25	0.049757	0.32023
Musculoskeletal	Fracture radius (HES and self-reported)	T	378957	5082	374019	0.859	-0.15199	0.077528	0.738	1	0.049936	0.32023
Musculoskeletal	Fracture upper limb shoulder (HES and self-reported)	T	378957	10515	368586	0.901	-0.10471	0.053165	0.811	0.999	0.048899	0.32023
Biological assays	Monocyte percentage	T	367073	NA	NA	NA	0.016626	0.008445	7.30E-05	0.033179	0.048994	0.32023
Biological assays	Neutrophill percentage	T	367073	NA	NA	NA	-0.01664	0.008445	-0.03319	-8.83E-05	0.048786	0.32023
Genitourinary	Other female pelvic inflammatory diseases (HES)	T	378957	2493	376608	1.2	0.185294	0.09486	0.999	1.45	0.050778	0.324458
Metabolic	Diabetes (HES and self-reported)	T	378957	15907	363194	0.919	-0.08422	0.043139	0.845	1	0.050911	0.324458
Eye	Other disorders of conjunctiva (HES)	T	378957	672	378429	0.621	-0.47709	0.245857	0.383	1	0.052317	0.327857
Musculoskeletal	Seropositive rheumatoid arthritis (HES)	T	378957	529	378572	1.44	0.365721	0.188327	0.997	2.09	0.052144	0.327857
Cardiovascular	Mitral valve repair replacement (self-reported)	T	378957	354	378747	0.476	-0.7422	0.38193	0.225	1.01	0.05198	0.327857
Operations and Procedures	Operations on spinal nerve root	T	378957	2302	376799	0.794	-0.23057	0.118505	0.629	1	0.051698	0.327857
Operations and Procedures	Correction of deformity of eyelid	T	378957	816	378285	1.35	0.30179	0.155486	0.997	1.83	0.052266	0.327857
Operations and Procedures	Transluminal balloon angioplasty of coronary artery	T	378957	2913	376188	0.816	-0.20285	0.104571	0.665	1	0.052397	0.327857
Gynaecology and Obstetrics	Ovarian problem (HES and self-reported)	T	378957	4956	374145	0.859	-0.15222	0.078391	0.736	1	0.052155	0.327857
Medication	Urologicals	T	378957	6031	373070	0.872	-0.13645	0.070563	0.76	1	0.053145	0.329
Cardiovascular	Atrioventricular and left bundle branch block (HES)	T	378957	3286	375815	0.829	-0.18776	0.0972	0.685	1	0.053399	0.329
Symptoms	Retention of urine (HES)	T	378957	6149	372952	1.13	0.120846	0.062553	0.998	1.28	0.053374	0.329
Operations and Procedures	Other therapeutic transluminal operations on vein	T	378957	1371	377730	1.27	0.238028	0.12307	0.997	1.61	0.053102	0.329
Operations and Procedures	Diagnostic endoscopic examination of urethra	T	378957	369	378732	1.53	0.426426	0.220249	0.995	2.36	0.052854	0.329
Musculoskeletal	Sciatica (HES and self-reported)	T	378957	5455	373646	0.866	-0.14422	0.074535	0.748	1	0.053005	0.329
Operations and Procedures	Preprosthetic oral surgery	T	378957	274	378827	1.62	0.483331	0.250713	0.992	2.65	0.053877	0.331093
Cardiovascular	Essential hypertension (HES and self-reported)	T	378957	71076	308025	0.958	-0.04289	0.022298	0.917	1	0.054442	0.333715
Haematology	Neutropenialymphopenia (HES and self-reported)	T	378957	2972	376129	1.18	0.167753	0.087353	0.997	1.4	0.054806	0.335094
Musculoskeletal	Osteopenia (HES and self-reported)	T	378957	990	378111	1.32	0.27393	0.143064	0.994	1.74	0.055526	0.338636
Medication	Antifungals for systemic use	T	378957	843	378258	1.35	0.296396	0.155163	0.992	1.82	0.056104	0.340221
Cardiovascular	Atrial fibrillation and flutter (HES)	T	378957	13484	365617	0.914	-0.0897	0.046951	0.834	1	0.056076	0.340221
Digestive system	Ventral hernia (HES)	T	378957	3093	376008	1.18	0.163764	0.085816	0.996	1.39	0.05635	0.340221
Other	Poisoning by narcotics and psychodysleptics (HES)	T	378957	598	378503	0.597	-0.51641	0.270597	0.351	1.01	0.056339	0.340221
Summary	Disorders of conjunctiva (HES)	T	378957	908	378193	0.679	-0.38653	0.203048	0.456	1.01	0.05696	0.34304
Eye	Other cataract (HES)	T	378957	13094	366007	1.09	0.084144	0.044316	0.997	1.19	0.057599	0.344321
Cancer	Breast cancer (self-reported)	T	378957	8389	370712	0.893	-0.11354	0.0598	0.794	1	0.057601	0.344321
Metabolic	Type 1 diabetes (HES and self-reported)	T	378957	2663	376438	0.812	-0.20789	0.109392	0.656	1.01	0.057377	0.344321
Digestive system	Cholecystitis (HES)	T	378957	2442	376659	1.2	0.181884	0.095971	0.994	1.45	0.058065	0.346233
Summary	Nutritional anaemias (HES)	T	378957	7757	371344	0.889	-0.11731	0.061934	0.788	1	0.058218	0.346291
Metabolic	Hypofunction and other disorders of pituitary gland (HES)	T	378957	359	378742	0.485	-0.72277	0.381855	0.23	1.03	0.058386	0.346434
Symptoms	Pain in throat and chest (HES)	T	378957	28963	350138	0.941	-0.06094	0.032235	0.883	1	0.058698	0.347426
Metabolic	Disorders of mineral metabolism (HES)	T	378957	1975	377126	1.22	0.199657	0.106135	0.992	1.5	0.05995	0.353967
Musculoskeletal	Dorsalgia (HES)	T	378957	13010	366091	0.915	-0.08936	0.047541	0.833	1	0.060145	0.354251
Haematology	Other anaemias (HES)	T	378957	10478	368623	0.905	-0.09936	0.053021	0.816	1	0.06093	0.357242
Operations and Procedures	Excision of salivary gland	T	378957	935	378166	0.689	-0.37293	0.19913	0.466	1.02	0.061097	0.357242
ENT	Speech reception threshold SRT estimate left	T	114644	NA	NA	NA	0.02801	0.014949	-0.00129	0.057309	0.060968	0.357242
Gynaecology and Obstetrics	Age started hormone replacement therapy HRT	T	71364	NA	NA	NA	0.036159	0.019336	-0.00174	0.074057	0.061481	0.358612
Medication	Ace inhibitors combinations	T	378957	54539	324562	0.955	-0.04621	0.024734	0.91	1	0.061704	0.359048
Operations and Procedures	Therapeutic endoscopic operations on peritoneum	T	378957	1599	377502	1.24	0.217605	0.116577	0.989	1.56	0.061955	0.359638
Eye	Disorders of orbit (HES)	T	378957	250	378851	0.392	-0.9368	0.50387	0.146	1.05	0.062997	0.364809
Medication	Diuretics and potassium sparing agents in combination	T	378957	27954	351147	0.941	-0.06117	0.03296	0.882	1	0.063477	0.366703
Summary	Mental disorders due to psychoactive sub (HES)	T	378957	14024	365077	0.918	-0.0858	0.046307	0.838	1	0.063896	0.368241
Summary	HESCH Diseases of the genitourinary system (HES)	T	378957	91318	287783	1.04	0.036098	0.019539	0.998	1.08	0.064682	0.37179
Musculoskeletal	Other necrotizing vasculopathies (HES)	T	378957	542	378559	1.42	0.347538	0.18822	0.979	2.05	0.064828	0.37179
Operations and Procedures	Cardioverter defibrillator introduced through the vein	T	378957	599	378502	0.617	-0.48291	0.26183	0.369	1.03	0.065129	0.37179
Gynaecology and Obstetrics	Breast fibroadenoma (HES and self-reported)	T	378957	1450	377651	0.753	-0.28337	0.153568	0.557	1.02	0.065	0.37179
Musculoskeletal	Disorders of patella (HES)	T	378957	841	378260	1.33	0.2849	0.155182	0.981	1.8	0.066371	0.371878
Musculoskeletal	Internal derangement of knee (HES)	T	378957	13926	365175	0.919	-0.0845	0.045918	0.84	1.01	0.06572	0.371878
Other	Poisoning antiepileptic hypnotic antiparkinsonism drugs (HES)	T	378957	851	378250	0.677	-0.38991	0.211642	0.447	1.03	0.065432	0.371878
Gynaecology and Obstetrics	Hysterectomy (self-reported)	T	378957	37549	341552	0.946	-0.05546	0.030167	0.892	1	0.066014	0.371878
Operations and Procedures	Lipoma removed excision of lipoma (self-reported)	T	378957	600	378501	1.39	0.329357	0.179368	0.978	1.98	0.066326	0.371878
Operations and Procedures	Other biopsy of skin	T	378957	1597	377504	1.24	0.214022	0.116483	0.986	1.56	0.066157	0.371878
Operations and Procedures	Other open repair of ligament	T	378957	420	378681	0.539	-0.61893	0.337135	0.278	1.04	0.066379	0.371878
Musculoskeletal	Fracture forearm wrist (HES and self-reported)	T	378957	8702	370399	0.898	-0.10712	0.058302	0.801	1.01	0.066175	0.371878
Operations and Procedures	Operations on hydrocele sac	T	378957	1029	378072	1.3	0.262267	0.142968	0.982	1.72	0.066588	0.372182
Operations and Procedures	Diagnostic endoscopic examination of knee joint	T	378957	3016	376085	0.829	-0.18765	0.102555	0.678	1.01	0.067283	0.375198
Operations and Procedures	Bone and intramedullary fixation	T	378957	2523	376578	0.814	-0.20577	0.112614	0.653	1.02	0.067666	0.376462
Operations and Procedures	Opening of abdomen	T	378957	940	378161	1.31	0.270455	0.148482	0.98	1.75	0.068536	0.380261
Neurosciences	Benign essential tremor (HES and self-reported)	T	378957	270	378831	0.44	-0.82182	0.451395	0.181	1.06	0.068665	0.380261
Immuno-inflammation	Other erythematous conditions (HES)	T	378957	481	378620	1.43	0.360511	0.198365	0.972	2.12	0.069154	0.382089
Other	Pack years	T	110892	NA	NA	NA	-0.0281	0.015488	-0.05845	0.00226	0.069661	0.384013
Neurosciences	Nerve root and plexus compressions (HES)	T	378957	3581	375520	0.846	-0.16773	0.092522	0.705	1.01	0.069853	0.38419
Operations and Procedures	Other therapeutic endoscopic operations on ureter	T	378957	1932	377169	1.21	0.19392	0.107249	0.984	1.5	0.070585	0.387335
Operations and Procedures	Intracranial haematoma drainage (self-reported)	T	378957	205	378896	1.68	0.518282	0.286868	0.957	2.95	0.07081	0.387685
Gynaecology and Obstetrics	Fertility treatment procedures (self-reported)	T	378957	966	378135	0.707	-0.34686	0.19222	0.485	1.03	0.071154	0.388682
Operations and Procedures	Therapeutic epidural injection	T	378957	4695	374406	0.866	-0.14405	0.079989	0.74	1.01	0.071734	0.390398
Operations and Procedures	Repair of muscle	T	378957	2257	376844	1.2	0.179158	0.099508	0.984	1.45	0.071792	0.390398
Neurosciences	Facial nerve disorders (HES)	T	378957	777	378324	1.33	0.28876	0.160765	0.974	1.83	0.072469	0.391041
Cardiovascular	Essential primary hypertension (HES)	T	378957	70382	308719	0.961	-0.04022	0.022363	0.919	1	0.072079	0.391041
Cardiovascular	Varicose vein surgery (self-reported)	T	378957	15382	363719	1.08	0.073668	0.041027	0.993	1.17	0.072559	0.391041
Operations and Procedures	Arteriotomy approach to organ under image control	T	378957	426	378675	0.562	-0.57568	0.320279	0.3	1.05	0.072268	0.391041
Medication	Lipid modifying agents combinations	T	378957	72923	306178	0.961	-0.04021	0.022502	0.919	1	0.07393	0.395863
Summary	Other diseases of pleura (HES)	T	378957	6040	373061	1.12	0.113156	0.063294	0.989	1.27	0.073811	0.395863
Summary	Congenital malformations of eye ear face and neck (HES)	T	378957	307	378794	0.479	-0.73671	0.41243	0.213	1.07	0.074057	0.395863
Summary	HESCH Certain infectious and parasitic diseases BIN (HES)	T	378957	26714	352387	1.06	0.056954	0.031931	0.994	1.13	0.074476	0.395863
Neurosciences	Disorders of autonomic nervous system (HES)	T	378957	241	378860	0.407	-0.89827	0.503931	0.152	1.09	0.074664	0.395863
Musculoskeletal	Other dorsopathies (HES)	T	378957	482	378619	1.43	0.354277	0.19838	0.966	2.1	0.074123	0.395863
Operations and Procedures	Reduction or fixation of bone fracture (self-reported)	T	378957	17474	361627	1.07	0.068937	0.038662	0.993	1.16	0.074572	0.395863
Operations and Procedures	Other repair of lip	T	378957	211	378890	0.355	-1.03463	0.581625	0.114	1.11	0.075262	0.395863
Operations and Procedures	Other vein related operations	T	378957	4293	374808	1.14	0.13186	0.074032	0.987	1.32	0.074891	0.395863
Operations and Procedures	Diagnostic imaging of abdomen	T	378957	2458	376643	1.19	0.171454	0.09636	0.983	1.43	0.07519	0.395863
Digestive system	Gastritisgastric erosions (HES and self-reported)	T	378957	21127	357974	0.935	-0.06692	0.037582	0.869	1.01	0.074964	0.395863
Family history	Breast cancer (family history - sibling)	T	298527	4866	293787	0.869	-0.14012	0.078885	0.745	1.01	0.075696	0.397281
Metabolic	Insulin dependent diabetes mellitus (HES)	T	378957	2572	376529	0.822	-0.19646	0.110687	0.661	1.02	0.075915	0.397565
Summary	Hypertensive diseases (HES)	T	378957	70604	308497	0.961	-0.03954	0.022334	0.92	1	0.076695	0.400519
Neurosciences	Hip pain	T	378087	48240	329991	0.956	-0.04504	0.02547	0.909	1	0.076978	0.400519
Respiratory	Bronchitis (HES and self-reported)	T	378957	3358	375743	0.844	-0.16933	0.095697	0.7	1.02	0.076814	0.400519
Operations and Procedures	Prosthesis for breast	T	378957	1450	377651	0.765	-0.26843	0.151904	0.568	1.03	0.07721	0.400859
Eye	Other disorders of iris and ciliary body (HES)	T	378957	272	378829	1.56	0.44195	0.250809	0.952	2.54	0.078052	0.401714

Genitourinary	Other disorders of urethra (HES)	T	378957	692	378409	1.35	0.298163	0.169298	0.967	1.88	0.078208	0.401714
Operations and Procedures	Arthroscopy nos (self-reported)	T	378957	8377	370724	0.901	-0.10462	0.059279	0.802	1.01	0.077582	0.401714
Operations and Procedures	Percutaneous transluminal balloon	T	378957	5594	373507	0.879	-0.12912	0.073259	0.761	1.01	0.07798	0.401714
Neurosciences	Maximum digits remembered correctly	T	116599	NA	NA	NA	0.026116	0.014825	-0.00294	0.055173	0.07813	0.401714
Operations and Procedures	Knee replacement revision (self-reported)	T	378957	4614	374487	0.868	-0.141	0.080256	0.742	1.02	0.078938	0.403043
Respiratory	Bring up phlegm sputum mucus on most days	T	95656	8125	87571	0.898	-0.10793	0.061417	0.796	1.01	0.078855	0.403043
Operations and Procedures	Opening into gastrointestinal tract	T	378957	207	378894	0.36	-1.02185	0.581716	0.115	1.13	0.078984	0.403043
Infectious disease	Hivaids (HES and self-reported)	T	378957	309	378792	1.53	0.427575	0.243532	0.951	2.47	0.079136	0.403043
ENT	Hearing aid user	T	230464	11351	219204	1.09	0.083814	0.04785	0.99	1.19	0.079843	0.405678
Operations and Procedures	Diagnostic microendoscopic examination of larynx	T	378957	246	378855	0.415	-0.88059	0.503822	0.154	1.11	0.080494	0.405678
Operations and Procedures	Repair of recurrent incisional hernia	T	378957	225	378876	1.62	0.48286	0.276264	0.943	2.79	0.080496	0.405678
Immuno-inflammation	Connective tissue disorder (HES and self-reported)	T	378957	2304	376797	1.19	0.172594	0.098676	0.979	1.44	0.080274	0.405678
Genitourinary	Polycystic kidney (HES and self-reported)	T	378957	1630	377471	1.22	0.202406	0.115768	0.976	1.54	0.080399	0.405678
Eye	Other serious eye condition	T	124323	6929	117447	0.892	-0.11433	0.065466	0.785	1.01	0.080737	0.406046
Haematology	Pancytopenia (HES and self-reported)	T	378957	406	378695	1.46	0.375034	0.214998	0.955	2.22	0.081097	0.407006
ENT	Dental tooth surgery (self-reported)	T	378957	999	378102	1.29	0.250928	0.144097	0.969	1.7	0.081618	0.408769
Operations and Procedures	Excision of nail	T	378957	608	378493	1.37	0.315157	0.181903	0.959	1.96	0.083176	0.415706
Operations and Procedures	Other total prosthetic replacement of other joint	T	378957	332	378769	0.516	-0.66128	0.382188	0.244	1.09	0.083588	0.416903
Summary	Urticaria and erythema (HES)	T	378957	948	378153	1.29	0.256553	0.148443	0.966	1.73	0.083936	0.41691
Operations and Procedures	Gestational age	T	378957	395	378706	1.46	0.376727	0.217871	0.951	2.23	0.083786	0.41691
Summary	Neurotic stress related and somatoform disorders (HES)	T	378957	6748	372353	0.892	-0.1144	0.066302	0.783	1.02	0.084447	0.41783
Operations and Procedures	Drainage of organ NOC	T	378957	2627	376474	1.17	0.160691	0.093136	0.978	1.41	0.084468	0.41783
Immuno-inflammation	Other disorders of skin and subcutaneous tissue (HES)	T	378957	5075	374026	1.13	0.118107	0.068626	0.984	1.29	0.085247	0.42082
Haematology	Agranulocytosis (HES)	T	378957	2906	376195	1.17	0.152938	0.088921	0.979	1.39	0.085443	0.420924
Genitourinary	Noninflammatory ovary fallop (HES)	T	378957	4666	374435	0.871	-0.13763	0.08022	0.745	1.02	0.086232	0.423947
Cancer	Mal neo oesophagus (cancer register)	T	378957	516	378585	0.618	-0.48188	0.281219	0.356	1.07	0.086613	0.424715
Operations and Procedures	Obliteration of cavity of organ NOC	T	378957	637	378464	0.656	-0.4212	0.246044	0.405	1.06	0.086918	0.424715
Digestive system	Bowel intestinal perforation (HES and self-reported)	T	378957	387	378714	1.46	0.376307	0.21982	0.947	2.24	0.086917	0.424715
Operations and Procedures	Suture of skin of head or neck	T	378957	774	378327	0.685	-0.37849	0.221468	0.444	1.06	0.087447	0.426435
Operations and Procedures	Operations on vitreous body	T	378957	3853	375248	1.14	0.132621	0.0778	0.98	1.33	0.088261	0.427847
Infectious disease	Helicobacter pylori (HES and self-reported)	T	378957	1773	377328	0.79	-0.23621	0.13857	0.602	1.04	0.088269	0.427847
Biological assays	Mean reticulocyte volume	T	361630	NA	NA	NA	-0.01451	0.008512	-0.0312	0.00217	0.08817	0.427847
Genitourinary	Calculus of kidney and ureter (HES)	T	378957	4404	374697	0.868	-0.14114	0.083106	0.738	1.02	0.089447	0.432686
Medication	Androgens	T	378957	555	378546	0.632	-0.45842	0.270961	0.372	1.08	0.090678	0.436887
Metabolic	Other disorders of thyroid (HES)	T	378957	357	378744	0.546	-0.60543	0.357782	0.271	1.1	0.090612	0.436887
Family history	Lung cancer (family history)	T	378957	NA	NA	NA	-0.01402	0.008301	-0.03029	0.002249	0.091202	0.437992
Eye	Intra ocular pressure corneal compensated right	T	81141	NA	NA	NA	-0.02994	0.017729	-0.06469	0.00481	0.091271	0.437992
Summary	Suppurative and necrotic conditions of lower respiratory tract (HES)	T	378957	346	378755	1.48	0.388899	0.23069	0.939	2.32	0.091833	0.438039
Infectious disease	Other septicaemia (HES)	T	378957	3387	375714	1.15	0.14004	0.08303	0.978	1.35	0.091676	0.438039
Operations and Procedures	Barium meal barium swallow (self-reported)	T	378957	221	378880	1.59	0.465761	0.276433	0.927	2.74	0.092008	0.438039
ENT	Mouth ulcers	T	377502	38476	339170	1.05	0.045694	0.027084	0.993	1.1	0.091574	0.438039
Operations and Procedures	Plastic operations on external ear	T	378957	299	378802	0.5	-0.69376	0.412523	0.223	1.12	0.092616	0.440061
Digestive system	Gastritis and duodenitis (HES)	T	378957	26187	352914	0.945	-0.05672	0.033858	0.884	1.01	0.093872	0.441914
Operations and Procedures	Oesphageal fundoplication hiatus hernia surgery (self-reported)	T	378957	906	378195	0.716	-0.33368	0.199236	0.485	1.06	0.093976	0.441914
Cancer	Other types of non Hodgkin s lymphoma (cancer register)	T	378957	408	378693	1.43	0.359856	0.214952	0.94	2.18	0.094106	0.441914
Operations and Procedures	Other excision of adnexa of uterus	T	378957	224	378877	1.59	0.462928	0.276488	0.924	2.73	0.094069	0.441914
Operations and Procedures	Radiology procedures	T	378957	30410	348691	1.05	0.050432	0.030085	0.992	1.12	0.093676	0.441914
Musculoskeletal	Fracture hand (HES and self-reported)	T	378957	1148	377953	0.746	-0.29272	0.174366	0.53	1.05	0.093197	0.441914
Digestive system	Oesophagitis (HES)	T	378957	8251	370850	1.1	0.091925	0.054951	0.984	1.22	0.094359	0.44224
Operations and Procedures	Other blood transfusion	T	378957	4631	374470	0.874	-0.1343	0.08048	0.747	1.02	0.095182	0.444428
Mental health	Obsessive compulsive disorder ocd (HES and self-reported)	T	378957	208	378893	1.61	0.478178	0.286733	0.92	2.83	0.09538	0.444428
Neurosciences	Prospective memory result	T	126215	NA	NA	NA	-0.02375	0.014233	-0.05164	0.00415	0.095228	0.444428
Summary	Mycoses (HES)	T	378957	2468	376633	1.17	0.160429	0.096341	0.972	1.42	0.095867	0.444978
Infectious disease	Candidiasis (HES)	T	378957	1983	377118	1.19	0.177657	0.106627	0.969	1.47	0.095683	0.444978
Gynaecology and Obstetrics	Haemorrhage in early pregnancy (HES)	T	378957	836	378265	0.706	-0.34783	0.209062	0.469	1.06	0.096156	0.445461
Operations and Procedures	Puncture of joint	T	378957	10120	368981	0.914	-0.08943	0.053809	0.823	1.02	0.096497	0.446184
Musculoskeletal	Soft tissue disorders related to use overuse and pressure (HES)	T	378957	1500	377601	0.781	-0.24655	0.148489	0.584	1.05	0.096839	0.446203
Genitourinary	Female genital prolapse (HES)	T	378957	10411	368690	1.09	0.082564	0.049731	0.985	1.2	0.096872	0.446203
Cardiovascular	Leg artery angioplasty stent (self-reported)	T	378957	443	378658	0.603	-0.50596	0.305665	0.331	1.1	0.097866	0.448445
Eye	Macular degeneration	T	124323	2870	121506	1.16	0.14882	0.08982	0.973	1.38	0.097547	0.448445
Gynaecology and Obstetrics	Endometriosis (HES and self-reported)	T	378957	5960	373141	0.89	-0.11684	0.070598	0.775	1.02	0.097917	0.448445
Cardiovascular	Cerebral infarction (HES)	T	378957	2917	376184	0.844	-0.16996	0.102811	0.69	1.03	0.098314	0.449407
Haematology	Other diseases of blood and blood forming organs (HES)	T	378957	563	378538	1.36	0.310096	0.18797	0.943	1.97	0.099002	0.451695
Operations and Procedures	Other reconstruction of joint	T	378957	2374	376727	0.83	-0.18692	0.113477	0.664	1.04	0.099513	0.45317
Respiratory	CopdExacerbation GOLD2orHigher	T	8822	486	8345	1.41	0.342845	0.208473	0.936	2.12	0.100061	0.454806
Summary	Ocular musclesocular movement (HES)	T	378957	2672	376429	1.16	0.151585	0.092422	0.971	1.39	0.100977	0.456386
Cardiovascular	Ischaemic stroke (HES and self-reported)	T	378957	2968	376133	0.846	-0.16709	0.101802	0.693	1.03	0.100734	0.456386
Cancer	Bowens disease (HES and self-reported)	T	378957	617	378484	1.34	0.294181	0.179312	0.944	1.91	0.100878	0.456386
Summary	Neoplasms BIN (cancer register)	T	378957	45225	333876	0.958	-0.04288	0.026237	0.91	1.01	0.102195	0.461026
Operations and Procedures	Other open operations on bile duct	T	378957	1260	377841	1.24	0.214768	0.131632	0.958	1.6	0.102768	0.462744
Operations and Procedures	Artificial opening into stomach	T	378957	356	378745	0.558	-0.58333	0.357786	0.277	1.13	0.10302	0.462824
Digestive system	Pyloric stenosis (HES and self-reported)	T	378957	250	378851	1.54	0.43448	0.266605	0.916	2.6	0.10317	0.462824
Summary	Disorders of synovium and tendon (HES)	T	378957	8361	370740	0.908	-0.096	0.058977	0.809	1.02	0.103589	0.462857
Summary	HESCH Diseases of the nervous system (HES)	T	378957	36198	342903	0.954	-0.04683	0.028813	0.902	1.01	0.10409	0.462857
Symptoms	Hyperhidrosis (HES)	T	378957	544	378557	0.644	-0.44026	0.27091	0.379	1.09	0.104138	0.462857
Operations and Procedures	Cardiac pacemaker system introduced through vein	T	378957	2286	376815	0.827	-0.18978	0.116531	0.658	1.04	0.10341	0.462857
Operations and Procedures	Other drainage of peritoneal cavity	T	378957	2011	377090	1.19	0.17242	0.106059	0.965	1.46	0.104012	0.462857
Mental health	Nervous breakdown (HES and self-reported)	T	378957	629	378472	0.671	-0.39902	0.246072	0.414	1.09	0.104902	0.465394
Operations and Procedures	Ankle surgery (self-reported)	T	378957	1003	378098	0.74	-0.3005	0.18562	0.515	1.07	0.105468	0.465409
Operations and Procedures	Operations on nasolacrimal duct	T	378957	416	378685	1.42	0.347948	0.214852	0.929	2.16	0.105345	0.465409
Operations and Procedures	Total prosthetic replacement of hip joint using cement	T	378957	4901	374200	0.881	-0.12668	0.078252	0.756	1.03	0.105485	0.465409
Summary	Viral hepatitis (HES)	T	378957	525	378576	0.635	-0.45416	0.280982	0.366	1.1	0.10602	0.466914
Biological assays	Basophill percentage	T	367073	NA	NA	NA	0.013616	0.008444	-0.00293	0.030166	0.106849	0.469707
Medication	Hormonal contraceptives for systemic use	T	378957	5576	373525	1.12	0.109933	0.068283	0.976	1.28	0.107406	0.469946
Cancer	Mal melanoma of skin (cancer register)	T	378957	2611	376490	0.84	-0.174	0.108231	0.68	1.04	0.107901	0.469946
Summary	Melanoma and other mal neo of skin (cancer register)	T	378957	2611	376490	0.84	-0.174	0.108231	0.68	1.04	0.107901	0.469946
Operations and Procedures	Abdominal excision of uterus	T	378957	9231	369870	0.913	-0.09106	0.05667	0.817	1.02	0.108074	0.469946
Biological assays	Sodium in urine	T	367322	NA	NA	NA	-0.01357	0.008442	-0.03012	0.002976	0.107939	0.469946
Respiratory	FEV1 FVC ratio strict	T	221584	NA	NA	NA	0.017556	0.010908	-0.00382	0.038935	0.107515	0.469946
Cardiovascular	Nonrheumatic aortic valve disorders (HES)	T	378957	2314	376787	0.83	-0.18582	0.115755	0.662	1.04	0.108424	0.470227
Neurosciences	Back pain	T	378087	102012	276219	0.97	-0.03036	0.018918	0.935	1.01	0.108529	0.470227
Operations and Procedures	Ureteric surgery (self-reported)	T	378957	604	378497	0.666	-0.4062	0.253576	0.405	1.1	0.109185	0.471374
Operations and Procedures	Simple extraction of tooth	T	378957	2335	376766	0.83	-0.18627	0.116232	0.661	1.04	0.109025	0.471374
Operations and Procedures	Other plastic operations on breast	T	378957	1690	377411	0.801	-0.222	0.138831	0.61	1.05	0.109807	0.471524
Neurosciences	Encephalitis (HES and self-reported)	T	378957	415	378686	1.41	0.343552	0.214799	0.925	2.15	0.109728	0.471524
Musculoskeletal	Osteoarthritis non load bearing joints	T	316942	4872	312194	0.884	-0.12342	0.077122	0.76	1.03	0.109521	0.471524
Operations and Procedures	Plastic repair of aortic valve	T	378957	1074	378027	0.753	-0.28322	0.17723	0.532	1.07	0.110037	0.471671
Operations and Procedures	Other excision of lesion of skin	T	378957	31445	347656	1.05	0.047399	0.029692	0.989	1.11	0.110407	0.472416
Medication	Lipid modifying agents plain	T	378957	93186	285915	0.968	-0.03214	0.020186	0.931	1.01	0.111361	0.472733
Cardiovascular	Disorders of circulatory system in disease (HES)	T	378957	256	378845	1.53	0.424507	0.266422	0.907	2.58	0.111078	0.472733
Genitourinary	Inflammatory disease of cervix uteri (HES)	T	378957	1044	378057	0.751	-0.28662	0.179999	0.528	1.07	0.111306	0.472733
Gynaecology and Obstetrics	Other complications of labour and delivery (HES)	T	378957	696	378405	1.31	0.272664	0.171541	0.938	1.84	0.111948	0.472733
ENT	Mouth salivary gland surgery (self-reported)	T	378957	2330	376771	0.831	-0.1849	0.116233	0.662	1.04	0.111655	0.472733
ENT	Wisdom teeth surgery (self-reported)	T	378957	15032	364069	1.07	0.066048	0.041429	0.985	1.16	0.110877	0.472733
Operations and Procedures	Other intravenous transfusion	T	378957	222	378879	0.449	-0.80113	0.504372	0.167	1.21	0.112202	0.472733
Gynaecology and Obstetrics	Vaginal prolapseuterine prolapse (HES and self-reported)	T	378957	11476	367625	1.08	0.075798	0.047691	0.982	1.18	0.111981	0.472733
Metabolic	Hypopituitarism (HES and self-reported)	T	378957	446	378655	0.616	-0.48516	0.305481	0.338	1.12	0.112247	0.472733
Mental health	Bipolar II Disorder	T	91467	531	90984	0.65	-0.43082	0.271497	0.382	1.11	0.112554	0.473198
Summary	Pregnancy with abortive outcome (HES)	T	378957	3363	375738	0.858	-0.15353	0.097003	0.709	1.04	0.11348	0.474485
Respiratory	Bronchitis not specified as acute or chronic (HES)	T	378957	570	378531	0.661	-0.41422	0.261829	0.396	1.1	0.113648	0.474485
Cardiovascular	Atrial fibrillation (HES and self-reported)	T	378957	2928	376173	0.852	-0.16066	0.101431	0.698	1.04	0.113205	0.474485
Mental health	Overall health rating	T	377297	NA	NA	NA	-0.0132	0.008336	-0.02954	0.003137	0.11328	0.474485
Musculoskeletal	Joint disorder (HES and self-reported)	T	378957	27274	351827	0.95	-0.0518	0.032883	0.89	1.01	0.115171	0.480012
Medication	Surgical aids	T	378957	1057	378044	1.25	0.224677	0.142701	0.946	1.66	0.11538	0.480053
Operations and Procedures	Biopsy of breast	T	378957	1566	377535	0.797	-0.22672	0.144249	0.601	1.06	0.11602	0.481883
Medication	Blood glucose lowering drugs excl insulins	T	378957	49513	329588	0.96	-0.0405	0.025814	0.913	1.01	0.116673	0.482602
Digestive system	Dyspepsia (HES)	T	378957	10739	368362	0.922	-0.08153	0.051982	0.832	1.02	0.116794	0.482602

Operations and Procedures	Placement of prosthesis in organ NOC	T	378957	3165	375936	0.857	-0.15392	0.0981	0.707	1.04	0.116639	0.482602
Immuno-inflammation	Psoriasis (HES and self-reported)	T	378957	5583	373518	1.11	0.103893	0.066301	0.974	1.26	0.117117	0.483108
Mental health	Other anxiety disorders (HES)	T	378957	5413	373688	0.891	-0.11559	0.074003	0.771	1.03	0.118291	0.483647
Digestive system	Other disorders of gingiva and edentulous alveolar ridge (HES)	T	378957	283	378818	0.525	-0.64416	0.412777	0.234	1.18	0.118631	0.483647
Medication	Drugs used in addictive disorders	T	378957	648	378453	1.32	0.279801	0.178977	0.931	1.88	0.117975	0.483647
Operations and Procedures	Excision of tonsil	T	378957	1168	377933	0.765	-0.26822	0.171978	0.546	1.07	0.118854	0.483647
Operations and Procedures	Curettage of lesion of skin	T	378957	1788	377313	1.19	0.174532	0.111565	0.957	1.48	0.117725	0.483647
Operations and Procedures	Other closed reduction of fracture of bone	T	378957	1496	377605	0.793	-0.23164	0.148516	0.593	1.06	0.118836	0.483647
Digestive system	Other abdominal problem (HES and self-reported)	T	378957	39890	339211	0.958	-0.04333	0.027723	0.907	1.01	0.118096	0.483647
Musculoskeletal	Dupuytrens contracture (HES and self-reported)	T	378957	3300	375801	1.14	0.132378	0.08468	0.967	1.35	0.117989	0.483647
Mental health	Ever highly irritable argumentative for 2 days	T	123697	22263	101496	0.942	-0.06008	0.038647	0.873	1.02	0.120012	0.487536
Summary	HESCH Endocrine nutritional and metabolic diseases BIN (HES)	T	378957	66768	312333	0.966	-0.03483	0.022446	0.924	1.01	0.120684	0.488825
Immuno-inflammation	Ulcer of lower limb (HES)	T	378957	989	378112	0.75	-0.28821	0.185739	0.521	1.08	0.120735	0.488825
Cardiovascular	Varicose veins (HES and self-reported)	T	378957	11798	367303	1.07	0.072134	0.046594	0.981	1.18	0.121586	0.491444
Operations and Procedures	Primary excision of lumbar intervertebral disc	T	378957	1796	377305	0.813	-0.20699	0.133791	0.625	1.06	0.121846	0.49167
Genitourinary	Kidney stoneureter stonebladder stone (HES and self-reported)	T	378957	6694	372407	0.903	-0.10243	0.066318	0.793	1.03	0.122466	0.493278
Digestive system	Dyspepsia indigestion (HES and self-reported)	T	378957	11162	367939	0.924	-0.07863	0.050934	0.837	1.02	0.122654	0.493278
Cardiovascular	Angiogram not coronary (self-reported)	T	378957	345	378756	0.576	-0.55155	0.357909	0.286	1.16	0.123307	0.495078
Operations and Procedures	Other operations on urethra	T	378957	1489	377612	0.796	-0.22846	0.148522	0.595	1.06	0.123988	0.496984
Operations and Procedures	Open drainage of bladder	T	378957	606	378495	1.32	0.279652	0.181946	0.926	1.89	0.124291	0.49737
Medication	Psycholeptics and psychoanaleptics in combination	T	378957	12227	366874	0.928	-0.07471	0.04868	0.844	1.02	0.124839	0.497969
Operations and Procedures	Other operations on mouth	T	378957	1260	377841	0.779	-0.25009	0.162957	0.566	1.07	0.124854	0.497969
Summary	Pulmonary heart disease and diseases of pulmonary circulation (HES)	T	378957	3992	375109	1.13	0.118078	0.077074	0.968	1.31	0.125521	0.498155
Summary	Diseases of veins lymphatic vessels (HES)	T	378957	35400	343701	1.04	0.043231	0.028216	0.988	1.1	0.125479	0.498155
Cancer	Rectal cancer (self-reported)	T	378957	274	378827	1.48	0.395159	0.257933	0.895	2.46	0.125518	0.498155
Medication	Drugs for peptic ulcer and gastro oesophageal reflux disease gord	T	378957	45634	333467	0.961	-0.03996	0.026212	0.913	1.01	0.127369	0.501359
Metabolic	Ovarian dysfunction (HES)	T	378957	239	378862	0.501	-0.6905	0.452466	0.207	1.22	0.126989	0.501359
Cancer	Neo of other sites (cancer register)	T	378957	206	378895	0.463	-0.77011	0.505091	0.172	1.25	0.127334	0.501359
Operations and Procedures	Therapeutic transluminal operations on other artery	T	378957	774	378327	0.719	-0.33029	0.216516	0.47	1.1	0.127145	0.501359
Operations and Procedures	Endovascular placement of stent	T	378957	370	378731	0.597	-0.51526	0.33772	0.308	1.16	0.127083	0.501359
Metabolic	Thyroid goitre (HES and self-reported)	T	378957	1793	377308	0.817	-0.20232	0.132798	0.63	1.06	0.127632	0.501575
Operations and Procedures	Artificial opening into jejunum	T	378957	294	378807	1.46	0.38031	0.250202	0.896	2.39	0.128508	0.504195
Haematology	Low plateletsplatelet disorder (HES and self-reported)	T	378957	1618	377483	1.2	0.178629	0.117871	0.949	1.51	0.129657	0.507876
Digestive system	Unspecified abdominal hernia (HES)	T	378957	276	378825	0.535	-0.62466	0.4129	0.238	1.2	0.130318	0.508303
Metabolic	Thyroid surgery (self-reported)	T	378957	586	378515	1.32	0.279733	0.184943	0.921	1.9	0.130399	0.508303
Operations and Procedures	Primary open reduction of traumatic dislocation of joint	T	378957	347	378754	1.43	0.357339	0.236226	0.9	2.27	0.130356	0.508303
Neurosciences	Other nervous system in diseases classified elsewhere (HES)	T	378957	960	378141	0.752	-0.28451	0.18882	0.52	1.09	0.131861	0.510906
Operations and Procedures	Examination of bile duct and pancreatic duct	T	378957	2192	376909	1.17	0.154228	0.102342	0.955	1.43	0.131815	0.510906
Cardiovascular	Transient ischaemic attack tia (HES and self-reported)	T	378957	3570	375531	1.13	0.122925	0.081591	0.964	1.33	0.131915	0.510906
Digestive system	Oesophagitisbarretts oesophagus (HES and self-reported)	T	378957	10521	368580	1.08	0.074278	0.049266	0.978	1.19	0.13163	0.510906
Gynaecology and Obstetrics	Preterm delivery (HES)	T	378957	602	378499	1.32	0.276417	0.183988	0.919	1.89	0.133003	0.512728
Operations and Procedures	Packing of cavity of nose	T	378957	1016	378085	1.24	0.218481	0.145432	0.936	1.65	0.133024	0.512728
Infectious disease	Herpes simplex (HES and self-reported)	T	378957	373	378728	0.602	-0.50749	0.337608	0.311	1.17	0.132793	0.512728
Digestive system	Other diseases of intestine (HES)	T	378957	13086	366015	0.932	-0.07072	0.047136	0.85	1.02	0.133516	0.513332
Cardiovascular	Mean carotid IMT intima medial thickness at 150 degrees	T	1843	NA	NA	NA	0.186925	0.124614	-0.05732	0.431168	0.133607	0.513332
Operations and Procedures	Diagnostic endos exam of lwr resp trct rigid brnchscpe	T	378957	452	378649	1.37	0.314941	0.210077	0.908	2.07	0.133831	0.513374
Medication	Ace inhibitors plain	T	378957	38569	340532	0.958	-0.04251	0.028393	0.907	1.01	0.134387	0.514358
Musculoskeletal	Fractured broken bones in last 5 years	T	376884	36458	340570	1.04	0.041639	0.027828	0.987	1.1	0.134582	0.514358
Cardiovascular	Cholesterol lowering medication	T	376081	64923	311299	0.966	-0.03507	0.023445	0.922	1.01	0.134728	0.514358
Biological assays	Lymphocyte percentage	T	367073	NA	NA	NA	0.012624	0.008445	-0.00393	0.029176	0.134965	0.514447
Cardiovascular	Coronary angioplasty ptca stent (self-reported)	T	378957	3922	375179	0.878	-0.13038	0.087284	0.74	1.04	0.135254	0.514734
Eye	LogMAR final right	T	83968	NA	NA	NA	0.025952	0.017423	-0.0082	0.060101	0.136346	0.518072
Musculoskeletal	Psoriatic arthropathy (HES and self-reported)	T	378957	1087	378014	1.24	0.211764	0.142422	0.935	1.63	0.137049	0.519923
Neurosciences	Alzheimer s disease (HES)	T	378957	359	378742	1.41	0.341188	0.230595	0.895	2.21	0.13898	0.525593
Mental health	Risk taking	T	365508	96122	269526	1.03	0.02895	0.01956	0.991	1.07	0.138865	0.525593
ENT	Suppurative and unspecified otitis media (HES)	T	378957	700	378401	1.29	0.25666	0.173699	0.92	1.82	0.139511	0.52595
Operations and Procedures	Other bowel surgery (self-reported)	T	378957	2282	376819	0.842	-0.17187	0.116291	0.67	1.06	0.139424	0.52595
Medication	Potassium sparing agents	T	378957	950	378151	0.757	-0.27833	0.188901	0.523	1.1	0.140643	0.525982
Infectious disease	Streptococcal septicaemia (HES)	T	378957	235	378866	0.514	-0.6654	0.452066	0.212	1.25	0.141048	0.525982
Gynaecology and Obstetrics	Abnormalities of forces of labour (HES)	T	378957	405	378696	0.636	-0.45327	0.307324	0.348	1.16	0.140243	0.525982
Operations and Procedures	Liver surgery (self-reported)	T	378957	301	378800	1.44	0.367984	0.249977	0.885	2.36	0.141001	0.525982
Operations and Procedures	Other upper digestive tract	T	378957	488	378613	1.35	0.297405	0.201858	0.906	2	0.140661	0.525982
Operations and Procedures	Branch of external carotid artery	T	378957	271	378830	1.46	0.3811	0.258119	0.883	2.43	0.139823	0.525982
Operations and Procedures	Bilateral excision of adnexa of uterus	T	378957	8743	370358	0.918	-0.08525	0.057888	0.82	1.03	0.140834	0.525982
Operations and Procedures	Gallstones removed (self-reported)	T	378957	315	378786	1.43	0.357432	0.243108	0.888	2.3	0.141492	0.526823
Operations and Procedures	High cost other cardiovascular drugs	T	378957	535	378566	1.33	0.285506	0.194461	0.909	1.95	0.142053	0.528095
Family history	Stroke (family history - father)	T	343458	51292	292300	0.964	-0.03639	0.024821	0.918	1.01	0.142628	0.529416
Family history	Parkinson s disease (family history - father)	T	338597	8344	330385	0.918	-0.08575	0.058596	0.818	1.03	0.143344	0.530972
Operations and Procedures	Placement of stent in organ NOC	T	378957	1320	377781	1.21	0.18956	0.129576	0.938	1.56	0.143488	0.530972
Operations and Procedures	Other operations on outlet of male bladder	T	378957	6418	372683	0.906	-0.09926	0.067962	0.793	1.03	0.144139	0.532563
Haematology	Other aplastic anaemias (HES)	T	378957	409	378692	1.38	0.319799	0.219484	0.895	2.12	0.145102	0.532852
Cardiovascular	Phlebitis and thrombophlebitis (HES)	T	378957	3502	375599	1.13	0.119861	0.082193	0.96	1.32	0.144762	0.532852
Musculoskeletal	Pagets disease (HES and self-reported)	T	378957	272	378829	0.548	-0.60195	0.412969	0.244	1.23	0.144946	0.532852
Musculoskeletal	Systemic lupus erythematosissle (HES and self-reported)	T	378957	547	378554	1.33	0.283607	0.194371	0.907	1.94	0.144537	0.532852
Eye	Disorders of globe (HES)	T	378957	389	378712	1.38	0.320108	0.219823	0.895	2.12	0.145336	0.532899
Operations and Procedures	Reconstruction of breast	T	378957	1391	377710	0.802	-0.22111	0.152011	0.595	1.08	0.145788	0.533744
Symptoms	Abnormalities of heart beat (HES)	T	378957	7716	371385	0.915	-0.08911	0.061339	0.811	1.03	0.146301	0.533999
ENT	Toothache	T	377502	15330	362316	1.06	0.060128	0.041382	0.979	1.15	0.146225	0.533999
Gynaecology and Obstetrics	Cone biopsy (self-reported)	T	378957	3152	375949	0.868	-0.14121	0.097341	0.717	1.05	0.146868	0.535257
Genitourinary	Scrotal problem not cancer (HES and self-reported)	T	378957	1382	377719	1.2	0.185123	0.127756	0.937	1.55	0.147329	0.536126
Biological assays	Lymphocyte count	T	367067	NA	NA	NA	0.012206	0.008441	-0.00434	0.028751	0.148153	0.538312
Cardiovascular	Rheumatic tricuspid valve diseases (HES)	T	378957	262	378839	1.47	0.383673	0.266211	0.871	2.47	0.149518	0.539259
Immuno-inflammation	Atrophic disorders of skin (HES)	T	378957	2542	376559	1.15	0.137638	0.095495	0.952	1.38	0.149494	0.539259
Operations and Procedures	Endoscopic of lower bowel	T	378957	3536	375565	0.876	-0.13265	0.091931	0.731	1.05	0.149036	0.539259
Genitourinary	Other renalkidney problem (HES and self-reported)	T	378957	5423	373678	1.1	0.096813	0.067176	0.966	1.26	0.149533	0.539259
Biological assays	Basophill count	T	367067	NA	NA	NA	0.01215	0.008413	-0.00434	0.028638	0.148663	0.539259
ENT	Tinnitus tiniitis (HES and self-reported)	T	378957	1088	378013	1.23	0.203095	0.14119	0.929	1.62	0.150307	0.540803
Musculoskeletal	Fracture ulna (HES and self-reported)	T	378957	1643	377458	0.819	-0.19943	0.138678	0.624	1.08	0.15041	0.540803
Medication	Other diagnostic radiopharmaceuticals	T	378957	3824	375277	0.881	-0.12691	0.088395	0.741	1.05	0.151072	0.541566
Operations and Procedures	Therapeutic on outlet of female bladder	T	378957	299	378802	0.577	-0.54958	0.382729	0.273	1.22	0.151015	0.541566
Cardiovascular	Aortic valve disease (HES and self-reported)	T	378957	2405	376696	0.851	-0.16095	0.112279	0.683	1.06	0.151728	0.54311
Summary	Congenital malformations musculoskeletal system (HES)	T	378957	711	378390	0.728	-0.31704	0.221743	0.472	1.12	0.152787	0.544519
Cardiovascular	Pulmonary embolism (HES)	T	378957	3296	375805	1.13	0.120922	0.084641	0.956	1.33	0.153106	0.544519
Cardiovascular	Stroke not specified as haemorrhage or infarction (HES)	T	378957	968	378133	1.24	0.214173	0.149967	0.923	1.66	0.153252	0.544519
Operations and Procedures	Other operations on oesophagus	T	378957	1839	377262	0.828	-0.18819	0.131574	0.64	1.07	0.152639	0.544519
Medication	Ibuprofen e g Nurofen	T	374624	55904	318864	1.03	0.033609	0.023518	0.988	1.08	0.152976	0.544519
Genitourinary	Other disorders of male genital organs (HES)	T	378957	2588	376513	1.15	0.137092	0.09627	0.95	1.39	0.154435	0.546306
Operations and Procedures	Incision of tongue	T	378957	994	378107	1.24	0.211095	0.148262	0.924	1.65	0.154505	0.546306
Operations and Procedures	Injection of therapeutic substance into organ NOC	T	378957	608	378493	0.704	-0.35039	0.246188	0.435	1.14	0.154662	0.546306
Operations and Procedures	Approach through abdominal cavity	T	378957	6488	372613	1.09	0.088035	0.061796	0.967	1.23	0.154269	0.546306
Operations and Procedures	Other release of peripheral nerve	T	378957	324	378777	1.41	0.344904	0.242807	0.877	2.27	0.155465	0.548338
Cancer	Neo of oral cavity and digestive (cancer register)	T	378957	341	378760	1.4	0.335363	0.236397	0.88	2.22	0.156003	0.549432
Operations and Procedures	Diagnostic endoscopic examination of colon	T	378957	48889	330212	0.965	-0.03585	0.025304	0.918	1.01	0.156567	0.549655
Operations and Procedures	Transluminal operations on renal artery	T	378957	241	378860	1.48	0.390466	0.275655	0.861	2.54	0.156629	0.549655
Biological assays	Creatinine enzymatic in urine	T	368096	NA	NA	NA	-0.01195	0.008439	-0.02849	0.00459	0.156751	0.549655
Operations and Procedures	Transluminal operations on iliac artery	T	378957	580	378521	0.698	-0.35915	0.253914	0.425	1.15	0.157228	0.550035
Haematology	Clotting disorderexcessive bleeding (HES and self-reported)	T	378957	2706	376395	1.14	0.131629	0.093079	0.95	1.37	0.157316	0.550035
Operations and Procedures	Combined operations on muscles of eye	T	378957	370	378731	1.38	0.325077	0.230158	0.882	2.17	0.15783	0.551033
Medication	Fish oil including cod liver oil	T	377706	119641	258209	0.975	-0.0258	0.018276	0.94	1.01	0.158089	0.551138
Operations and Procedures	Diagnostic endoscopic examination of lower bowel	T	378957	29456	349645	0.956	-0.0447	0.03173	0.899	1.02	0.158923	0.552753
Operations and Procedures	Other lymph node	T	378957	2930	376171	0.867	-0.14217	0.100942	0.712	1.06	0.159011	0.552753
Symptoms	Pain associated with micturition (HES)	T	378957	1067	378034	0.78	-0.24876	0.177089	0.551	1.1	0.160099	0.555733
Cardiovascular	Heart failurepulmonary odema (HES and self-reported)	T	378957	5221	373880	0.9	-0.10526	0.074996	0.777	1.04	0.160464	0.556198
Musculoskeletal	Fracture neck of femur hip (HES and self-reported)	T	378957	2221	376880	1.15	0.142783	0.101856	0.945	1.41	0.160971	0.557154
Cardiovascular	Paroxysmal tachycardia (HES)	T	378957	2977	376124	0.869	-0.14028	0.100252	0.714	1.06	0.16173	0.558177
Summary	Mal neo of ill secondary unspecified (cancer register)	T	378957	433	378668	1.35	0.30037	0.214574	0.887	2.06	0.16156	0.558177
Medication	Estrogens	T	378957	10090	369011	0.928	-0.07488	0.053643	0.835	1.03	0.162722	0.558322

Summary	Symptoms and signs involving speech and voice (HES)	T		378957	2293	376808	0.852	-0.1607	0.115585	0.679	1.07	0.164444	0.558322
Digestive system	Other functional intestinal disorders (HES)	T		378957	10172	368929	0.929	-0.07412	0.053381	0.836	1.03	0.164999	0.558322
Gynaecology and Obstetrics	Medical abortion (HES)	T		378957	998	378103	0.774	-0.2557	0.184294	0.54	1.11	0.165305	0.558322
Gynaecology and Obstetrics	Breast surgery (self-reported)	T		378957	1320	377781	0.806	-0.21583	0.155514	0.594	1.09	0.165177	0.558322
Operations and Procedures	Hip surgery not replacement (self-reported)	T		378957	1523	377578	1.18	0.168788	0.121094	0.934	1.5	0.163359	0.558322
ENT	Tympanic membrane surgery ear drum repair (self-reported)	T		378957	602	378499	1.29	0.257477	0.184698	0.901	1.86	0.163304	0.558322
Family history	Alzheimer s disease dementia (family history - sibling)	T		300905	1677	299352	0.827	-0.18963	0.136644	0.633	1.08	0.165202	0.558322
Operations and Procedures	Therapeutic endoscopic operations on calculus of kidney	T		378957	417	378684	0.654	-0.42408	0.305785	0.359	1.19	0.165482	0.558322
Operations and Procedures	Diagnostic endoscopic examination of bladder	T		378957	30109	348992	1.04	0.042698	0.030553	0.983	1.11	0.162252	0.558322
Operations and Procedures	High cost haematology and nutrition drugs	T		378957	606	378495	1.29	0.255984	0.184678	0.899	1.86	0.165712	0.558322
Operations and Procedures	Injection of other substance into organ NOC	T		378957	1838	377263	0.834	-0.181	0.129555	0.647	1.08	0.162394	0.558322
NA	Subarachnoid haemorrhage	T		378957	982	378119	1.23	0.208587	0.149801	0.918	1.65	0.163794	0.558322
Neurosciences	Meningitis (HES and self-reported)	T		378957	1714	377387	0.829	-0.18778	0.13501	0.636	1.08	0.16427	0.558322
Digestive system	Diverticular diseasediverticulitis (HES and self-reported)	T		378957	26531	352570	0.954	-0.04673	0.033521	0.894	1.02	0.163277	0.558322
Neurosciences	Polio poliomyelitis (HES and self-reported)	T		378957	280	378821	0.588	-0.53083	0.382952	0.278	1.25	0.165698	0.558322
Digestive system	Bowel intestinal infarction (HES and self-reported)	T		378957	264	378837	0.564	-0.57347	0.413106	0.251	1.27	0.165081	0.558322
Summary	Other disorders of the skin and subcutaneous tissue (HES)	T		378957	14568	364533	1.06	0.058335	0.042222	0.976	1.15	0.167086	0.559042
Symptoms	Abnormal serum enzyme levels (HES)	T		378957	226	378875	1.49	0.395794	0.285985	0.848	2.6	0.166368	0.559042
Gynaecology and Obstetrics	Mastectomy (self-reported)	T		378957	3844	375257	0.886	-0.12097	0.087551	0.746	1.05	0.167078	0.559042
Operations and Procedures	Haemorroidectomy piles surgery banding of piles (self-reported)	T		378957	6713	372388	1.09	0.084213	0.060893	0.965	1.23	0.166672	0.559042
Family history	Bowel cancer (family history - sibling)	T		301109	7001	294235	0.914	-0.0895	0.064707	0.805	1.04	0.166615	0.559042
Medication	Androgens and female sex hormones in combination	T		378957	421	378680	0.656	-0.4214	0.30576	0.36	1.19	0.168135	0.55941
Eye	Retinal disorders in diseases classified elsewhere (HES)	T		378957	1229	377872	0.801	-0.22192	0.161106	0.584	1.1	0.168357	0.55941
Gynaecology and Obstetrics	Salpingectomy (self-reported)	T		378957	258	378843	0.565	-0.57033	0.413332	0.251	1.27	0.167636	0.55941
Operations and Procedures	Operations on external nose	T		378957	3310	375791	1.12	0.116629	0.084627	0.952	1.33	0.168158	0.55941
Mental health	Depression (HES and self-reported)	T		378957	27882	351219	0.956	-0.04487	0.032561	0.897	1.02	0.168174	0.55941
Musculoskeletal	Other systemic involvement of connective tissue (HES)	T		378957	1710	377391	1.17	0.158198	0.115037	0.935	1.47	0.169071	0.561008
Summary	Disorders of muscles (HES)	T		378957	1025	378076	0.781	-0.24671	0.179866	0.549	1.11	0.170171	0.561663
Genitourinary	Pain with female genital (HES)	T		378957	3073	376028	0.873	-0.13542	0.098588	0.72	1.06	0.169556	0.561663
ENT	Loose teeth	T		377502	15091	362555	0.942	-0.06002	0.043762	0.864	1.03	0.170201	0.561663
Digestive system	Femoral hernia (HES and self-reported)	T		378957	643	378458	0.726	-0.31981	0.233112	0.46	1.15	0.170086	0.561663
Operations and Procedures	Excision of vas deferens	T		378957	4286	374815	0.887	-0.1194	0.08729	0.748	1.05	0.171369	0.564744
Genitourinary	Urethral stricture (HES)	T		378957	2967	376134	0.871	-0.13762	0.100733	0.715	1.06	0.171891	0.56569
Operations and Procedures	Closed reduction of fracture of bone and external fixation	T		378957	391	378710	0.646	-0.43757	0.320542	0.344	1.21	0.172224	0.566013
Medication	Progestogens and estrogens in combination	T		378957	8666	370435	1.08	0.074308	0.054616	0.968	1.2	0.173653	0.567844
Operations and Procedures	Exenteration of mastoid air cells	T		378957	445	378656	1.34	0.29162	0.214374	0.879	2.04	0.173724	0.567844
Operations and Procedures	Other operations on rectum	T		378957	894	378207	1.24	0.213246	0.156657	0.91	1.68	0.173443	0.567844
Operations and Procedures	Percutaneous puncture of kidney	T		378957	1437	377664	1.19	0.170905	0.125489	0.928	1.52	0.173227	0.567844
Eye	Iritis (HES and self-reported)	T		378957	479	378622	1.32	0.279423	0.205714	0.884	1.98	0.174366	0.5684
Neurosciences	Meningioma benign meningeal tumour (HES and self-reported)	T		378957	499	378602	0.692	-0.36867	0.271325	0.406	1.18	0.174218	0.5684
Medication	Antidepressants	T		378957	29214	349887	0.958	-0.04315	0.031841	0.9	1.02	0.175346	0.570822
Digestive system	Cysts of oral region (HES)	T		378957	377	378724	0.649	-0.43303	0.320695	0.346	1.22	0.176922	0.5744
Symptoms	Disturbances of skin sensation (HES)	T		378957	2617	376484	0.865	-0.14534	0.107632	0.7	1.07	0.176906	0.5744
Medication	Antipruritics incl antihistamines anesthetics etc	T		378957	592	378509	1.29	0.253218	0.187664	0.892	1.86	0.177236	0.574645
Medication	Chemotherapeutics for topical use	T		378957	1020	378081	0.785	-0.24237	0.179791	0.552	1.12	0.177632	0.575155
Genitourinary	Other abnormal uterine and vaginal bleeding (HES)	T		378957	3736	375365	0.887	-0.12006	0.08919	0.745	1.06	0.178267	0.576437
Summary	Other bacterial diseases (HES)	T		378957	4393	374708	1.11	0.09993	0.0744	0.955	1.28	0.179227	0.577082
Haematology	Purpura and other haemorrhagic conditions (HES)	T		378957	1530	377571	1.18	0.16348	0.121901	0.927	1.5	0.179891	0.577082
Metabolic	Obesity (HES)	T		378957	9864	369237	0.93	-0.07248	0.053913	0.837	1.03	0.178809	0.577082
Cancer	Bladder cancer (self-reported)	T		378957	892	378209	1.23	0.208456	0.15515	0.909	1.67	0.179087	0.577082
Operations and Procedures	Removal of mole skin lesion (self-reported)	T		378957	5310	373791	1.1	0.0914	0.068107	0.959	1.25	0.179595	0.577082
Musculoskeletal	Back problem (HES and self-reported)	T		378957	11524	367577	0.935	-0.06685	0.049853	0.848	1.03	0.179904	0.577082
Musculoskeletal	Fracture fibula (HES and self-reported)	T		378957	749	378352	1.25	0.225812	0.168918	0.9	1.75	0.181284	0.580736
Medication	Antithrombotic agents	T		378957	59142	319959	0.968	-0.03209	0.024033	0.924	1.02	0.181737	0.581077
Medication	Paracetamol	T		374624	81842	292926	1.03	0.027078	0.020283	0.987	1.07	0.181873	0.581077
Genitourinary	Anal fissure (HES and self-reported)	T		378957	269	378832	1.42	0.354143	0.265976	0.846	2.4	0.183029	0.583996
Cardiovascular	Carotid artery surgery endarterectomy (self-reported)	T		378957	226	378875	0.548	-0.6017	0.452496	0.226	1.33	0.183607	0.585065
Operations and Procedures	Renal kidney transplant (self-reported)	T		378957	252	378849	1.44	0.365637	0.275282	0.84	2.47	0.184104	0.585874
Family history	Father s age at death	T		278443	NA	NA	NA	0.012808	0.009662	-0.00613	0.031746	0.184969	0.58785
Operations and Procedures	Hand finger surgery (self-reported)	T		378957	1664	377437	0.835	-0.18046	0.136264	0.639	1.09	0.185395	0.588417
Operations and Procedures	Extirpation of lesion of bone	T		378957	466	378635	0.689	-0.37249	0.281419	0.397	1.2	0.185636	0.588417
Musculoskeletal	Osteonecrosis (HES)	T		378957	512	378589	1.3	0.2661	0.201556	0.879	1.94	0.18676	0.590426
Immuno-inflammation	Chronic skin ulcers (HES and self-reported)	T		378957	1331	377770	0.816	-0.20292	0.153674	0.604	1.1	0.186674	0.590426
Family history	Bowel cancer (family history)	T		378957	NA	NA	NA	-0.01092	0.008285	-0.02716	0.005318	0.187452	0.591837
Eye	Other strabismus (HES)	T		378957	823	378278	1.24	0.213913	0.162385	0.901	1.7	0.187732	0.591946
Neurosciences	Other disorders of brain (HES)	T		378957	1438	377663	1.18	0.16504	0.125448	0.922	1.51	0.188307	0.592162
Operations and Procedures	Repair of other hernia of abdominal wall	T		378957	1344	377757	1.19	0.170118	0.129375	0.92	1.53	0.188538	0.592162
Infectious disease	Measles morbillivirus (HES and self-reported)	T		378957	1296	377805	1.19	0.170411	0.129521	0.92	1.53	0.188274	0.592162
Gynaecology and Obstetrics	Labour and delivery complicated by umbilical cord complications (HES)	T		378957	570	378531	1.28	0.249095	0.189656	0.885	1.86	0.189048	0.59299
Operations and Procedures	Colposcopy biopsy (self-reported)	T		378957	1641	377460	0.835	-0.18053	0.137682	0.637	1.09	0.189786	0.593239
Operations and Procedures	Attention to prosthesis in organ NOC	T		378957	2929	376172	1.13	0.117824	0.089848	0.943	1.34	0.189735	0.593239
Neurosciences	Acute infective polyneuritisguillainbarre syndro (HES and self-reported)	T		378957	290	378811	1.4	0.337535	0.257469	0.846	2.32	0.189866	0.593239
Operations and Procedures	Release of entrapment of peripheral nerve at other site	T		378957	778	378323	1.24	0.218322	0.166629	0.897	1.72	0.19012	0.593263
Gynaecology and Obstetrics	Cervical polyps removed (self-reported)	T		378957	370	378731	1.35	0.301486	0.230359	0.861	2.12	0.190613	0.593812
Operations and Procedures	Plastic excision of skin of head or neck	T		378957	208	378893	1.48	0.389393	0.297645	0.824	2.65	0.190789	0.593812
ENT	Sinus surgery (self-reported)	T		378957	1301	377800	1.19	0.170534	0.130482	0.918	1.53	0.191229	0.594414
Operations and Procedures	Other diagnostic imaging of genitourinary system	T		378957	209	378892	1.48	0.388667	0.297612	0.823	2.64	0.191569	0.594703
Summary	HESCH Diseases of the eye and adnexa (HES)	T		378957	35574	343527	1.04	0.037109	0.028493	0.981	1.1	0.192784	0.595406
Immuno-inflammation	Lichen planus (HES)	T		378957	501	378600	0.702	-0.35384	0.271311	0.412	1.19	0.192176	0.595406
Symptoms	Voice disturbances (HES)	T		378957	934	378167	0.782	-0.24618	0.18892	0.54	1.13	0.192544	0.595406
Operations and Procedures	Plastic operations on cornea	T		378957	258	378843	0.584	-0.53842	0.413245	0.26	1.31	0.19261	0.595406
Family history	High blood pressure (family history - sibling)	T		300905	61052	239977	0.97	-0.03071	0.023592	0.926	1.02	0.193063	0.595504
Operations and Procedures	Diagnostic imaging of chest	T		378957	2157	376944	1.15	0.135762	0.104384	0.933	1.41	0.193396	0.595768
Medication	Selective calcium channel blockers with mainly vascular effects	T		378957	24151	354950	0.955	-0.04555	0.035094	0.892	1.02	0.194295	0.59618
Cardiovascular	Other diseases of pericardium (HES)	T		378957	973	378128	0.789	-0.23707	0.182837	0.551	1.13	0.194767	0.59618
Cancer	Lymphoid leukaemia (cancer register)	T		378957	519	378582	0.712	-0.34007	0.262276	0.426	1.19	0.19476	0.59618
Operations and Procedures	Microscopically controlled excision of lesion of skin	T		378957	920	378181	1.22	0.19881	0.153216	0.903	1.65	0.194431	0.59618
Operations and Procedures	Excision of other fascia	T		378957	2947	376154	1.12	0.116946	0.090165	0.942	1.34	0.194626	0.59618
ENT	Otitis externa (HES)	T		378957	488	378613	1.31	0.266258	0.205587	0.872	1.95	0.195281	0.596695
Digestive system	Embedded and impacted teeth (HES)	T		378957	1708	377393	0.84	-0.17483	0.1351	0.644	1.09	0.195629	0.596695
Musculoskeletal	Fibroblastic disorders (HES)	T		378957	3613	375488	1.11	0.105881	0.081872	0.947	1.31	0.195926	0.596695
Genitourinary	Testicular problems not cancer (HES and self-reported)	T		378957	1283	377818	0.814	-0.20603	0.159266	0.596	1.11	0.195791	0.596695
Summary	Maternal care related to the fetus (HES)	T		378957	6059	373042	1.09	0.089652	0.069371	0.955	1.25	0.196236	0.596885
Summary	Aplastic and other anaemias (HES)	T		378957	11319	367782	0.937	-0.06498	0.050309	0.849	1.03	0.196508	0.596958
Digestive system	Jaundice unknown cause (HES and self-reported)	T		378957	1143	377958	0.806	-0.2159	0.167415	0.58	1.12	0.197195	0.598291
Gynaecology and Obstetrics	Premature rupture of membranes (HES)	T		378957	1011	378090	0.796	-0.22825	0.177134	0.562	1.13	0.19755	0.598614
Operations and Procedures	Primary repair of inguinal hernia	T		378957	13724	365377	1.06	0.057318	0.044507	0.971	1.16	0.197801	0.598621
Eye	Retinal detachment (HES and self-reported)	T		378957	3632	375469	1.11	0.104154	0.080971	0.947	1.3	0.198332	0.599475
Genitourinary	Other disorders of prostate (HES)	T		378957	1235	377866	0.815	-0.20509	0.159633	0.596	1.11	0.198887	0.599648
Operations and Procedures	Procurement of drugs for chemotherapy for neoplasm in Bands 6 10	T		378957	4078	375023	1.1	0.098183	0.076404	0.95	1.28	0.198773	0.599648
Operations and Procedures	Open operations on synovial membrane of joint	T		378957	853	378248	0.778	-0.25141	0.195808	0.53	1.14	0.199161	0.599724
Neurosciences	Dystonia (HES)	T		378957	231	378870	1.44	0.366678	0.285771	0.824	2.53	0.19945	0.599844
Medication	Antiinflammatory antirheumatic agents in combination	T		378957	53678	325423	0.969	-0.03184	0.024868	0.923	1.02	0.200431	0.602043
Digestive system	Diverticular disease of intestine (HES)	T		378957	24930	354171	0.957	-0.044	0.034461	0.894	1.02	0.201644	0.602865
Medication	Macrolides lincosamides and streptogramins	T		378957	736	378365	0.758	-0.2765	0.216689	0.496	1.16	0.201956	0.602865
Operations and Procedures	Hybrid prosthetic replacement of hip joint	T		378957	286	378815	0.613	-0.48903	0.382936	0.29	1.3	0.201589	0.602865
Anthropometry	Body fat percentage	T		372114	NA	NA	NA	0.010691	0.008377	-0.00573	0.027109	0.201866	0.602865
Musculoskeletal	Legs BMD bone mineral density	T		4100	NA	NA	NA	-0.11028	0.086268	-0.27936	0.058811	0.20115	0.602865
Mental health	Anxietypanic attacks (HES and self-reported)	T		378957	10392	368709	0.935	-0.06707	0.052599	0.844	1.04	0.202269	0.603052
Medication	Antiemetics and antinauseants	T		378957	234	378867	1.44	0.361756	0.285652	0.82	2.51	0.205361	0.607136
Summary	Symptoms and signs involving the skin and subcutaneous tissue (HES)	T		378957	6510	372591	0.919	-0.08458	0.066598	0.806	1.05	0.204077	0.607136
Infectious disease	Bacterial infection of unspecified site (HES)	T		378957	696	378405	1.25	0.223126	0.176163	0.885	1.77	0.205302	0.607136
Operations and Procedures	Minimal access to thoracic cavity	T		378957	726	378375	1.24	0.217434	0.171297	0.888	1.74	0.20432	0.607136
Operations and Procedures	Local anaesthetic	T		378957	23563	355538	1.04	0.043107	0.034051	0.977	1.12	0.205525	0.607136
Cardiovascular	High cholesterol (HES and self-reported)	T		378957	62396	316705	0.971	-0.02971	0.023461	0.927	1.02	0.205418	0.607136
Gynaecology and Obstetrics	Benign breast lump (HES and self-reported)	T		378957	2336	376765	0.867	-0.14313	0.113016	0.694	1.08	0.205356	0.607136
Other	Age when periods started menarche	T		198421	NA	NA	NA	0.014466	0.01143	-0.00794	0.036869	0.205655	0.607136

ENT	Disorders of vestibular function (HES)	T	378957	871	378230	1.22	0.200361	0.158482	0.896	1.67	0.206139	0.60782
Operations and Procedures	Therapeutic endoscopic operations on cavity of other joint	T	378957	583	378518	1.27	0.240993	0.190763	0.876	1.85	0.206476	0.608069
Operations and Procedures	Diagnostic endoscopic examination of mediastinum	T	378957	587	378514	1.27	0.239974	0.190794	0.875	1.85	0.208478	0.613216
Genitourinary	Excessive frequent and irregular menstruation (HES)	T	378957	11974	367127	0.939	-0.06246	0.049899	0.852	1.04	0.210683	0.618192
Digestive system	Abdominal hernia (HES and self-reported)	T	378957	20644	358457	1.05	0.045974	0.036725	0.974	1.13	0.210618	0.618192
Summary	Infections sexual mode of transmission (HES)	T	378957	253	378848	0.597	-0.51592	0.413316	0.266	1.34	0.211939	0.621121
Summary	Obesity and other hyperalimentation (HES)	T	378957	9921	369180	0.935	-0.06689	0.053635	0.842	1.04	0.212348	0.621189
Respiratory	Pleural plaque (HES)	T	378957	645	378456	1.25	0.227069	0.182124	0.878	1.79	0.212478	0.621189
Family history	Stroke (family history - sibling)	T	300905	9517	291512	0.933	-0.06888	0.055299	0.838	1.04	0.212945	0.621799
Cancer	Mal neo bladder (cancer register)	T	378957	919	378182	1.21	0.190607	0.153484	0.896	1.63	0.214286	0.624958
Operations and Procedures	Appendicectomy (self-reported)	T	378957	45428	333673	1.03	0.031515	0.025392	0.982	1.08	0.214561	0.625003
Medication	Cardiac stimulants excl cardiac glycosides	T	378957	304	378797	0.641	-0.44436	0.358494	0.318	1.29	0.215157	0.625048
Summary	Symptoms circulatory and respiratory systems (HES)	T	378957	45652	333449	0.968	-0.03216	0.026022	0.92	1.02	0.216562	0.625048
Musculoskeletal	Other joint disorders (HES)	T	378957	11928	367173	0.941	-0.06048	0.048937	0.855	1.04	0.216484	0.625048
Musculoskeletal	Systemic lupus erythematosus (HES)	T	378957	324	378777	1.36	0.308692	0.249493	0.835	2.22	0.215984	0.625048
Operations and Procedures	Reconstruction of breast using abdominal flap	T	378957	275	378826	0.623	-0.47338	0.383152	0.294	1.32	0.216652	0.625048
Operations and Procedures	Injection into varicose vein of leg	T	378957	823	378278	1.23	0.203299	0.164392	0.888	1.69	0.216207	0.625048
Operations and Procedures	Other excision of testis	T	378957	508	378593	1.29	0.254381	0.205623	0.862	1.93	0.216043	0.625048
Respiratory	Pneumothorax (HES and self-reported)	T	378957	906	378195	1.21	0.194233	0.156599	0.893	1.65	0.214859	0.625048
Haematology	Aplastic anaemia (HES and self-reported)	T	378957	429	378672	1.31	0.270422	0.219184	0.853	2.01	0.217291	0.626141
Operations and Procedures	Other operations on mastoid	T	378957	342	378759	1.35	0.29889	0.24242	0.838	2.17	0.217598	0.626277
Summary	Congenital malformations of the circulatory system (HES)	T	378957	1083	378018	1.19	0.176928	0.143746	0.9	1.58	0.218382	0.627742
Digestive system	Other disorders of teeth and supporting structures (HES)	T	378957	2410	376691	1.13	0.122087	0.0993	0.93	1.37	0.218889	0.627742
Operations and Procedures	Manipulation of rectum	T	378957	1760	377341	1.15	0.141996	0.11548	0.919	1.45	0.21884	0.627742
Respiratory	Pulmonary oedema (HES)	T	378957	428	378673	0.698	-0.35916	0.293056	0.393	1.24	0.220356	0.631121
Genitourinary	Polyp of female genital tract (HES)	T	378957	9793	369308	1.06	0.06291	0.051404	0.963	1.18	0.221015	0.631121
Operations and Procedures	Pituitary surgery (self-reported)	T	378957	215	378886	0.575	-0.5535	0.452583	0.237	1.4	0.221338	0.631121
Operations and Procedures	Other operations on breast	T	378957	910	378191	0.794	-0.23127	0.189123	0.548	1.15	0.221377	0.631121
Operations and Procedures	Open drainage of peritoneum	T	378957	297	378804	1.37	0.314985	0.25728	0.828	2.27	0.220843	0.631121
Medication	Progestogens	T	378957	2010	377091	1.14	0.131908	0.108237	0.923	1.41	0.22296	0.631144
Cardiovascular	Arterial embolism and thrombosis (HES)	T	378957	839	378262	0.784	-0.24372	0.199577	0.53	1.16	0.222026	0.631144
Musculoskeletal	Other disorders of muscle (HES)	T	378957	806	378295	0.781	-0.24763	0.203423	0.524	1.16	0.223481	0.631144
Gynaecology and Obstetrics	Long labour (HES)	T	378957	1773	377328	0.851	-0.16097	0.131891	0.657	1.1	0.222283	0.631144
Cancer	Cin pre cancer cells cervix (self-reported)	T	378957	1313	377788	0.831	-0.18529	0.152209	0.617	1.12	0.223478	0.631144
Cardiovascular	Blood pressure medication	T	376081	77608	298614	0.974	-0.02635	0.021636	0.934	1.02	0.223189	0.631144
Digestive system	Oesophageal varicies (HES and self-reported)	T	378957	501	378600	1.29	0.250797	0.205493	0.859	1.92	0.222287	0.631144
Other	Other substance abusedependency (HES and self-reported)	T	378957	10328	368773	0.937	-0.06456	0.053018	0.845	1.04	0.223351	0.631144
Gynaecology and Obstetrics	Other disorders of amniotic fluid and membranes (HES)	T	378957	236	378865	0.604	-0.5042	0.414571	0.268	1.36	0.223909	0.63135
Genitourinary	Renal failure requiring dialysis (HES and self-reported)	T	378957	660	378441	0.759	-0.27634	0.227301	0.486	1.18	0.224078	0.63135
Operations and Procedures	Excision or biopsy of lymph node	T	378957	6160	372941	0.921	-0.08282	0.068293	0.805	1.05	0.225266	0.633955
Mental health	Mental and behavioural disorders due to use of tobacco (HES)	T	378957	10134	368967	0.937	-0.0648	0.053495	0.844	1.04	0.225751	0.634579
Gynaecology and Obstetrics	Other obstructed labour (HES)	T	378957	309	378792	1.36	0.304136	0.25136	0.828	2.22	0.226292	0.635358
Neurosciences	Anorexiabulimiaother eating disorder (HES and self-reported)	T	378957	336	378765	1.34	0.293249	0.242737	0.833	2.16	0.227011	0.636635
Neurosciences	Other extrapyramidal and movement disorders (HES)	T	378957	562	378539	0.743	-0.29734	0.246484	0.458	1.2	0.227695	0.63671
Genitourinary	Tubulo interstitial nephritis not specified as acute or chronic (HES)	T	378957	837	378264	1.22	0.195764	0.162347	0.885	1.67	0.227882	0.63671
Metabolic	Parathyroidectomy (self-reported)	T	378957	531	378570	0.736	-0.30629	0.254123	0.447	1.21	0.228095	0.63671
Respiratory	Asbestosis (HES and self-reported)	T	378957	271	378830	0.63	-0.4623	0.38345	0.297	1.34	0.227964	0.63671
Gynaecology and Obstetrics	Placental disorders (HES)	T	378957	225	378876	1.41	0.345143	0.287568	0.804	2.48	0.230058	0.638396
Medication	Immunosuppressants	T	378957	3722	375379	1.1	0.096793	0.080816	0.94	1.29	0.231036	0.638396
Family history	Prostate cancer (family history - father)	T	338597	25442	313287	0.96	-0.04039	0.033765	0.899	1.03	0.231614	0.638396
Cancer	Carc in situ of other sites (cancer register)	T	378957	561	378540	0.744	-0.2955	0.246691	0.459	1.21	0.230979	0.638396
Operations and Procedures	Other repair of bladder	T	378957	216	378885	1.43	0.355896	0.297301	0.797	2.56	0.231272	0.638396
Operations and Procedures	Operations on bursa	T	378957	1825	377276	0.857	-0.15425	0.128556	0.666	1.1	0.230196	0.638396
Operations and Procedures	Operations on sentinel lymph node	T	378957	1143	377958	0.82	-0.19838	0.165421	0.593	1.13	0.23043	0.638396
Operations and Procedures	Minimal access to other body cavity	T	378957	8899	370202	1.07	0.064142	0.053406	0.96	1.18	0.229739	0.638396
Metabolic	Insulin	T	376081	3934	372288	0.902	-0.10336	0.085971	0.762	1.07	0.229255	0.638396
Digestive system	Umbilical hernia (HES and self-reported)	T	378957	3644	375457	0.898	-0.10713	0.089536	0.754	1.07	0.231481	0.638396
Immuno-inflammation	Lichen planus (HES and self-reported)	T	378957	627	378474	0.756	-0.27934	0.23321	0.479	1.19	0.230985	0.638396
Neurosciences	Stomach or abdominal pain	T	378087	37960	340271	1.03	0.032911	0.027555	0.979	1.09	0.232325	0.639624
Summary	Other diseases of blood and blood forming organs (HES)	T	378957	4191	374910	1.1	0.090819	0.076319	0.943	1.27	0.234047	0.639976
Medication	Anticholinergic agents	T	378957	240	378861	1.4	0.339689	0.285448	0.803	2.46	0.234039	0.639976
Gynaecology and Obstetrics	Endometrial ablation (self-reported)	T	378957	1925	377176	1.14	0.130822	0.109829	0.919	1.41	0.233595	0.639976
Operations and Procedures	Other operations on spine	T	378957	4622	374479	0.91	-0.09387	0.078881	0.78	1.06	0.234019	0.639976
Metabolic	Hyperthyroidismthyrotoxicosis (HES and self-reported)	T	378957	4020	375081	0.904	-0.10117	0.084885	0.765	1.07	0.233336	0.639976
Genitourinary	Nephritis (HES and self-reported)	T	378957	1824	377277	1.14	0.134426	0.112783	0.917	1.43	0.233301	0.639976
Medication	Other sex hormones and modulators of the genital system	T	378957	368	378733	1.32	0.27996	0.236129	0.833	2.1	0.235771	0.641052
Operations and Procedures	Other operations on kidney	T	378957	281	378820	1.37	0.315466	0.265656	0.814	2.31	0.235031	0.641052
Operations and Procedures	Diagnostic endoscopic examination of peritoneum	T	378957	4723	374378	0.911	-0.09366	0.078972	0.78	1.06	0.235615	0.641052
Gynaecology and Obstetrics	Polycystic ovariespolycystic ovarian syndrome (HES and self-reported)	T	378957	580	378521	0.752	-0.28496	0.240245	0.47	1.2	0.23558	0.641052
Gynaecology and Obstetrics	Cervical problem (HES and self-reported)	T	378957	6385	372716	1.08	0.074536	0.062768	0.953	1.22	0.235041	0.641052
ENT	Otalgia and effusion of ear (HES)	T	378957	479	378622	1.28	0.24817	0.209696	0.85	1.93	0.236623	0.642644
Respiratory	Unspecified chronic bronchitis (HES)	T	378957	243	378858	1.4	0.336797	0.285409	0.8	2.45	0.23798	0.644954
Musculoskeletal	Bone disorder (HES and self-reported)	T	378957	5041	374060	1.09	0.082728	0.070109	0.947	1.25	0.238009	0.644954
Operations and Procedures	Diagnostic imaging of genitourinary system	T	378957	293	378808	1.37	0.31264	0.265387	0.813	2.3	0.238775	0.646302
Musculoskeletal	Fracture neck cervical fracture (HES and self-reported)	T	378957	345	378756	1.33	0.285278	0.242374	0.827	2.14	0.23919	0.646699
Cardiovascular	Multiple valve diseases (HES)	T	378957	1492	377609	1.16	0.145317	0.123787	0.907	1.47	0.240426	0.648909
Medication	Other diagnostic agents	T	378957	392	378709	0.699	-0.35876	0.306035	0.383	1.27	0.241085	0.648909
Operations and Procedures	Intramuscular injection	T	378957	1308	377793	0.835	-0.18012	0.15364	0.618	1.13	0.24106	0.648909
Musculoskeletal	Fracture sternum (HES and self-reported)	T	378957	240	378861	0.616	-0.48508	0.413594	0.274	1.38	0.240864	0.648909
Respiratory	FEV1 FVC ratio	T	286064	NA	NA	NA	0.011224	0.009591	-0.00757	0.030021	0.241888	0.650344
Musculoskeletal	Other joint disorder (HES and self-reported)	T	378957	9807	369294	0.939	-0.06315	0.054037	0.844	1.04	0.242533	0.651351
Summary	Other congenital malformations of the digestive system (HES)	T	378957	632	378469	1.24	0.215383	0.184472	0.864	1.78	0.242983	0.651833
Medication	Vitamin a and d incl combinations of the two	T	378957	10919	368182	1.06	0.057015	0.048972	0.962	1.17	0.244328	0.653374
Musculoskeletal	Paget s disease of bone (HES)	T	378957	243	378858	0.618	-0.48153	0.413515	0.275	1.39	0.244227	0.653374
Eye	Corneal resistance factor right	T	81141	NA	NA	NA	-0.02064	0.017731	-0.05539	0.014112	0.244371	0.653374
Other	Sleep duration	T	376654	NA	NA	NA	-0.00968	0.008334	-0.02602	0.006651	0.245251	0.655
Gynaecology and Obstetrics	Antepartum haemorrhage (HES)	T	378957	588	378513	1.25	0.219592	0.189747	0.859	1.81	0.247156	0.658627
Operations and Procedures	Other operations on anterior chamber of eye	T	378957	405	378696	0.712	-0.33954	0.293301	0.401	1.27	0.247005	0.658627
Medication	Antiarrhythmics class i and iii	T	378957	1297	377804	0.837	-0.17741	0.153735	0.62	1.13	0.248506	0.660787
Cancer	Reported occurrences of cancer	T	61089	NA	NA	NA	0.023362	0.020245	-0.01632	0.063043	0.248515	0.660787
Respiratory	Respiratory disorders in diseases classified elsewhere (HES)	T	378957	201	378900	1.43	0.356859	0.310476	0.777	2.63	0.250394	0.661491
Medication	Decongestants and antiallergics	T	378957	420	378681	0.714	-0.33715	0.293085	0.402	1.27	0.249995	0.661491
Cancer	Squamous cell carcinoma (self-reported)	T	378957	470	378631	0.731	-0.31304	0.271587	0.429	1.25	0.249056	0.661491
Operations and Procedures	Removal of epididymal cyst (self-reported)	T	378957	269	378832	0.644	-0.44037	0.383175	0.304	1.36	0.250446	0.661491
Operations and Procedures	Other replacement of aneurysmal segment of aorta	T	378957	295	378806	1.34	0.295908	0.257614	0.811	2.23	0.250702	0.661491
Operations and Procedures	Insertion of skin expander into subcutaneous tissue	T	378957	446	378655	0.723	-0.32411	0.28179	0.416	1.26	0.250073	0.661491
Digestive system	Constipation (HES and self-reported)	T	378957	9731	369370	0.939	-0.06241	0.054245	0.845	1.04	0.249909	0.661491
Summary	Chronic rheumatic heart diseases (HES)	T	378957	1969	377132	1.13	0.124829	0.109032	0.915	1.4	0.252257	0.664139
Respiratory	CopdMappingStrict	T	189997	16733	173340	1.05	0.049322	0.043056	0.966	1.14	0.251997	0.664139
Operations and Procedures	Removal of testicle orchidectomy (self-reported)	T	378957	1211	377890	1.17	0.15854	0.138645	0.893	1.54	0.252833	0.664416
Operations and Procedures	Harvest of other multiple tissue	T	378957	281	378820	1.35	0.303763	0.26569	0.805	2.28	0.252914	0.664416
Digestive system	Gastricstomach ulcers (HES and self-reported)	T	378957	6128	372973	0.925	-0.07847	0.068863	0.808	1.06	0.25448	0.667802
Summary	Disorders of optic nerve and visual pathways (HES)	T	378957	383	378718	0.706	-0.34768	0.306149	0.388	1.29	0.256101	0.668418
Summary	Diseases of arteries arterioles and capillaries (HES)	T	378957	6751	372350	0.929	-0.07418	0.065296	0.817	1.06	0.255917	0.668418
Cancer	Mal neo rectum (cancer register)	T	378957	1203	377898	1.17	0.156091	0.137289	0.893	1.53	0.255557	0.668418
Operations and Procedures	Local anaesthetics for ophthalmology procedures	T	378957	5563	373538	1.08	0.075712	0.066616	0.947	1.23	0.255725	0.668418
Operations and Procedures	Blood withdrawal	T	378957	3031	376070	1.11	0.101232	0.08914	0.929	1.32	0.256102	0.668418
Genitourinary	Chronic nephritic syndrome (HES)	T	378957	791	378310	0.797	-0.22669	0.199772	0.539	1.18	0.256479	0.668677
Digestive system	Peritonitis (HES and self-reported)	T	378957	1989	377112	1.13	0.123406	0.108848	0.914	1.4	0.256901	0.669054
Summary	Disorders of other endocrine glands (HES)	T	378957	2354	376747	0.881	-0.12692	0.112111	0.707	1.1	0.257582	0.670103
Cardiovascular	LV stroke volume	T	3764	NA	NA	NA	0.101269	0.089531	-0.07421	0.27675	0.258009	0.670489
Infectious disease	Chronic viral hepatitis (HES)	T	378957	343	378758	0.683	-0.3813	0.338087	0.352	1.32	0.259398	0.672824
Cardiovascular	Pacemaker insertion (self-reported)	T	378957	287	378814	0.667	-0.40468	0.358868	0.33	1.35	0.259466	0.672824
Digestive system	Oesophageal varices (HES)	T	378957	488	378613	1.27	0.235187	0.209641	0.839	1.91	0.261923	0.676284
Operations and Procedures	Pancreas surgery (self-reported)	T	378957	205	378896	0.601	-0.50891	0.452836	0.247	1.46	0.261088	0.676284
Mental health	Ever depressed for a whole week	T	124001	66758	57306	1.03	0.032851	0.029276	0.976	1.09	0.261822	0.676284
Musculoskeletal	Vasculitis (HES and self-reported)	T	378957	1756	377345	1.14	0.129052	0.114947	0.908	1.43	0.261561	0.676284

Cardiovascular	Heart failure (HES)	T	378957	4854	374247	0.917	-0.08626	0.077071	0.789	1.07	0.263035	0.677702
Operations and Procedures	Other operations on trabecular meshwork of eye	T	378957	236	378865	0.629	-0.46331	0.413747	0.28	1.42	0.262802	0.677702
Summary	Other congenital malformations (HES)	T	378957	615	378486	1.23	0.209381	0.187468	0.854	1.78	0.264044	0.67819
Cardiovascular	Varicose veins of lower extremities (HES)	T	378957	9985	369116	1.06	0.056751	0.050874	0.958	1.17	0.264632	0.67819
Gynaecology and Obstetrics	Myomectomy fibroids removed (self-reported)	T	378957	2071	377030	1.13	0.119245	0.106761	0.914	1.39	0.264021	0.67819
Operations and Procedures	Operations on omentum	T	378957	1948	377153	0.871	-0.13844	0.123969	0.683	1.11	0.264121	0.67819
Operations and Procedures	Other primary fusion of other joint	T	378957	1462	377639	0.851	-0.16093	0.144215	0.642	1.13	0.264456	0.67819
Medication	Opioids	T	378957	102262	276839	1.02	0.020977	0.018818	0.984	1.06	0.264972	0.67834
Eye	Diabetic eye disease (HES and self-reported)	T	378957	1737	377364	0.865	-0.14555	0.130747	0.669	1.12	0.265613	0.679259
Respiratory	Pneumothorax (HES)	T	378957	762	378339	1.21	0.189347	0.171084	0.864	1.69	0.268402	0.682816
Gynaecology and Obstetrics	Spontaneous abortion (HES)	T	378957	1258	377843	0.841	-0.17352	0.157102	0.618	1.14	0.26937	0.682816
Operations and Procedures	Muscle soft tissue surgery (self-reported)	T	378957	10926	368175	1.06	0.053969	0.048743	0.959	1.16	0.268201	0.682816
Operations and Procedures	Arteriovenous shunt	T	378957	297	378804	0.673	-0.39598	0.358608	0.333	1.36	0.269504	0.682816
Cardiovascular	Heart valve problemheart murmur (HES and self-reported)	T	378957	7532	371569	0.934	-0.0678	0.061321	0.829	1.05	0.268894	0.682816
Cardiovascular	Arterial embolism (HES and self-reported)	T	378957	762	378339	0.795	-0.22943	0.207801	0.529	1.19	0.269555	0.682816
Eye	Optic neuritis (HES and self-reported)	T	378957	206	378895	0.606	-0.50142	0.452872	0.249	1.47	0.268212	0.682816
Musculoskeletal	Fracture face orbit eye socket (HES and self-reported)	T	378957	477	378624	0.741	-0.29994	0.271516	0.435	1.26	0.269291	0.682816
Other	Getting up in morning	T	377919	NA	NA	NA	-0.00921	0.008331	-0.02554	0.007116	0.268797	0.682816
Operations and Procedures	Other operations on blood vessel	T	378957	317	378784	0.689	-0.37211	0.338425	0.355	1.34	0.27154	0.687122
Summary	Urolithiasis (HES)	T	378957	5984	373117	0.927	-0.07621	0.069365	0.809	1.06	0.271907	0.687328
Operations and Procedures	Autonomic cardiovascular testing	T	378957	235	378866	0.636	-0.4531	0.413683	0.283	1.43	0.273391	0.690355
Gynaecology and Obstetrics	Mammoplasty cosmetic operation on breast (self-reported)	T	378957	2699	376402	0.892	-0.11389	0.104085	0.728	1.09	0.273884	0.690876
Operations and Procedures	Extirpation of lesion of conjunctiva	T	378957	351	378750	0.704	-0.35045	0.321034	0.375	1.32	0.275004	0.692498
Operations and Procedures	Destruction of lesion of cervix uteri	T	378957	2136	376965	0.879	-0.12902	0.118212	0.697	1.11	0.275102	0.692498
Summary	Coagulation defects purpura and other haemorrhagic conditions (HES)	T	378957	2426	376675	1.11	0.108133	0.09929	0.917	1.35	0.276126	0.69435
Cancer	Non melanoma skin cancer (self-reported)	T	378957	550	378551	0.765	-0.26755	0.246623	0.472	1.24	0.277981	0.695385
Operations and Procedures	Bone surgery joint surgery (self-reported)	T	378957	3136	375965	0.901	-0.10438	0.096206	0.746	1.09	0.277935	0.695385
Summary	Mal neo of digestive organs (cancer register)	T	378957	5957	373144	1.07	0.070511	0.064848	0.945	1.22	0.276893	0.695385
Genitourinary	Kidney nephropathy (HES and self-reported)	T	378957	417	378684	1.28	0.243551	0.224219	0.822	1.98	0.277381	0.695385
Gynaecology and Obstetrics	Dysmenorrhoea dysmenorrhea (HES and self-reported)	T	378957	1606	377495	0.862	-0.14866	0.13691	0.659	1.13	0.277547	0.695385
Genitourinary	Inflammatory disease of uterus except cervix (HES)	T	378957	260	378841	0.66	-0.4153	0.383443	0.311	1.4	0.27877	0.696636
Operations and Procedures	Sampling of lymph nodes	T	378957	2952	376149	0.898	-0.1072	0.099153	0.74	1.09	0.279609	0.698008
Operations and Procedures	Other cardiac pacemaker system	T	378957	1141	377960	0.839	-0.17601	0.163389	0.609	1.16	0.28136	0.701652
Operations and Procedures	Removal of products of delivered uterus	T	378957	339	378762	0.707	-0.34687	0.322269	0.376	1.33	0.281772	0.701713
Immuno-inflammation	Eczemadermatitis (HES and self-reported)	T	378957	11296	367805	1.05	0.051437	0.047807	0.959	1.16	0.281967	0.701713
Summary	Intestinal infectious diseases (HES)	T	378957	8022	371079	1.06	0.060429	0.056433	0.951	1.19	0.284251	0.70321
Mental health	Specific personality disorders (HES)	T	378957	293	378808	0.681	-0.38453	0.358693	0.337	1.38	0.283702	0.70321
Symptoms	Somnolence stupor and coma (HES)	T	378957	636	378465	0.784	-0.24351	0.227436	0.502	1.22	0.28432	0.70321
Symptoms	Enlarged lymph nodes (HES)	T	378957	2200	376901	1.12	0.111622	0.104247	0.911	1.37	0.284285	0.70321
Operations and Procedures	Other caesarean delivery	T	378957	1946	377155	0.875	-0.13354	0.124379	0.686	1.12	0.282982	0.70321
Digestive system	Inguinal hernia (HES and self-reported)	T	378957	15351	363750	1.05	0.045485	0.042467	0.963	1.14	0.28413	0.70321
Mental health	Happiness	T	125989	NA	NA	NA	0.015309	0.014312	-0.01274	0.043361	0.284783	0.703633
Respiratory	Pneumonia due to Streptococcus pneumoniae (HES)	T	378957	234	378867	0.643	-0.44216	0.413736	0.286	1.45	0.285199	0.703939
Cardiovascular	Complications and ill defined descriptions of heart disease (HES)	T	378957	3773	375328	0.911	-0.09327	0.087427	0.767	1.08	0.286069	0.705135
Respiratory	Asthma (HES)	T	378957	23838	355263	0.964	-0.03705	0.034744	0.9	1.03	0.286269	0.705135
Operations and Procedures	Operations on nipple	T	378957	610	378491	0.78	-0.24818	0.233391	0.494	1.23	0.287615	0.707727
Operations and Procedures	Ultrasound scan (self-reported)	T	378957	230	378871	0.644	-0.43963	0.413833	0.286	1.45	0.288088	0.708167
Operations and Procedures	Other operations on anterior abdominal wall	T	378957	600	378501	1.22	0.202115	0.190611	0.842	1.78	0.288983	0.708199
Operations and Procedures	Revisional decompression operations on lumbar spine	T	378957	287	378814	0.684	-0.38046	0.358804	0.338	1.38	0.288979	0.708199
Operations and Procedures	Other open operations on intra articular structure	T	378957	1473	377628	0.859	-0.15149	0.142818	0.65	1.14	0.288812	0.708199
Medication	Omeprazole e g Zanprol	T	374624	22414	352354	0.963	-0.03809	0.036039	0.897	1.03	0.290565	0.711353
Respiratory	Pneumonitis due to solids and liquids (HES)	T	378957	560	378541	1.23	0.208124	0.197626	0.836	1.81	0.292286	0.71484
Operations and Procedures	Other puncture of liver	T	378957	403	378698	1.27	0.241532	0.22961	0.812	2	0.292834	0.715165
Cardiovascular	Subarachnoid haemorrhage (HES and self-reported)	T	378957	936	378165	1.18	0.164491	0.156429	0.868	1.6	0.293013	0.715165
Operations and Procedures	Excision of organ NOC	T	378957	1832	377269	1.13	0.119041	0.113355	0.902	1.41	0.293641	0.715383
Respiratory	Chronic obstructive airways diseasecopd (HES and self-reported)	T	378957	8796	370305	1.06	0.057312	0.054581	0.952	1.18	0.293696	0.715383
Summary	Acute upper respiratory infections (HES)	T	378957	2654	376447	0.895	-0.1105	0.105387	0.728	1.1	0.2944	0.71565
Operations and Procedures	Other operations on tonsil	T	378957	503	378598	0.76	-0.27506	0.26231	0.454	1.27	0.294357	0.71565
Genitourinary	Pyelonephritis (HES and self-reported)	T	378957	1141	377960	1.16	0.14879	0.14228	0.878	1.53	0.295675	0.718025
Musculoskeletal	Scoliosis (HES and self-reported)	T	378957	1135	377966	0.843	-0.1706	0.163306	0.612	1.16	0.296169	0.718501
Respiratory	Pleural plaques not known asbestosis (HES and self-reported)	T	378957	690	378411	1.21	0.187007	0.179189	0.849	1.71	0.296658	0.718963
Operations and Procedures	Dilation of organ NOC	T	378957	512	378589	0.767	-0.26495	0.254186	0.466	1.26	0.297257	0.719691
Summary	Other soft tissue disorders (HES)	T	378957	25100	354001	1.04	0.034416	0.033057	0.97	1.1	0.297834	0.720364
Operations and Procedures	Other leg region	T	378957	287	378814	0.689	-0.37313	0.358797	0.341	1.39	0.298363	0.720919
Operations and Procedures	Operations on urethral orifice	T	378957	464	378637	0.754	-0.28223	0.271674	0.443	1.28	0.298873	0.721428
Respiratory	Pleural effusion (HES)	T	378957	4725	374376	1.08	0.075045	0.072419	0.935	1.24	0.300079	0.723614
Summary	Schizophrenia schizotypal and delusional disorders (HES)	T	378957	899	378202	0.823	-0.19514	0.189029	0.568	1.19	0.301919	0.723782
Infectious disease	Bacterial agents as the cause of diseases (HES)	T	378957	7935	371166	1.06	0.058725	0.056899	0.949	1.19	0.302028	0.723782
Neurosciences	Epilepsy (HES)	T	378957	3387	375714	1.09	0.08803	0.085332	0.924	1.29	0.302252	0.723782
Operations and Procedures	Knee surgery not replacement (self-reported)	T	378957	12555	366546	0.952	-0.04944	0.04776	0.867	1.05	0.300549	0.723782
ENT	Rhinoplasty nose surgery (self-reported)	T	378957	2193	376908	0.887	-0.11947	0.115692	0.707	1.11	0.301748	0.723782
Operations and Procedures	Other excision of right hemicolon	T	378957	1421	377680	0.86	-0.1504	0.145728	0.647	1.14	0.302037	0.723782
Operations and Procedures	Attention to skin expander in subcutaneous tissue	T	378957	431	378670	0.748	-0.29089	0.281935	0.43	1.3	0.302192	0.723782
Respiratory	Pyothorax (HES)	T	378957	288	378813	1.31	0.273698	0.26551	0.781	2.21	0.302616	0.723934
ENT	Thyroiditis (HES and self-reported)	T	378957	442	378659	1.25	0.225591	0.219035	0.816	1.92	0.303043	0.724237
Infectious disease	Herpesviral (HES)	T	378957	232	378869	0.654	-0.42414	0.413783	0.291	1.47	0.305352	0.729032
Operations and Procedures	Excision of lung	T	378957	810	378291	1.19	0.170411	0.166528	0.856	1.64	0.30616	0.729644
Operations and Procedures	Other operations on delivered uterus	T	378957	225	378876	1.36	0.305004	0.298175	0.756	2.43	0.306354	0.729644
Operations and Procedures	Therapeutic endoscopic operations on cavity of knee joint	T	378957	4112	374989	0.919	-0.085	0.083122	0.78	1.08	0.306517	0.729644
Medication	Antipsychotics	T	378957	2920	376181	0.903	-0.10181	0.099767	0.743	1.1	0.30748	0.730951
Operations and Procedures	Extirpation of lesion of vagina	T	378957	675	378426	0.801	-0.22149	0.217123	0.524	1.23	0.307673	0.730951
Eye	Eye surgery (self-reported)	T	378957	3495	375606	0.912	-0.09246	0.090817	0.763	1.09	0.308657	0.732495
Operations and Procedures	Removal of repair material from skin	T	378957	460	378641	0.759	-0.27616	0.271585	0.446	1.29	0.309235	0.732495
Medication	Folic acid or Folate Vit B9	T	376892	8174	368862	0.942	-0.05997	0.058953	0.839	1.06	0.309036	0.732495
Operations and Procedures	Other operations on eyelid	T	378957	931	378170	0.831	-0.18565	0.182953	0.58	1.19	0.310227	0.734123
Digestive system	Gastroenteritisdysentry (HES and self-reported)	T	378957	8121	370980	1.06	0.056788	0.056183	0.948	1.18	0.312123	0.737885
Digestive system	Fissure and fistula of anal and rectal regions (HES)	T	378957	2863	376238	1.1	0.093474	0.092612	0.916	1.32	0.312828	0.738559
Operations and Procedures	Therapeutic spinal puncture	T	378957	253	378848	1.33	0.287431	0.285	0.762	2.33	0.313201	0.738559
Digestive system	Gastrooesophageal reflux gord gastric reflux (HES and self-reported)	T	378957	32601	346500	0.97	-0.03041	0.030157	0.914	1.03	0.313328	0.738559
Medication	Drugs for functional gastrointestinal disorders	T	378957	3769	375332	1.08	0.081423	0.080861	0.926	1.27	0.313961	0.738606
Gynaecology and Obstetrics	Menorrhagia unknown cause (HES and self-reported)	T	378957	12816	366285	0.953	-0.04832	0.047968	0.867	1.05	0.313728	0.738606
Neurosciences	Sleep disorders (HES)	T	378957	4841	374260	0.926	-0.07724	0.076981	0.796	1.08	0.315663	0.738794
Cardiovascular	Rheumatic mitral valve diseases (HES)	T	378957	286	378815	0.698	-0.36012	0.35885	0.345	1.41	0.315606	0.738794
Symptoms	Other symptoms and signs involving the digestive system and abdomen (HES)	T	378957	13806	365295	1.04	0.043739	0.043669	0.959	1.14	0.316535	0.738794
Eye	Cataract extraction lens implant (self-reported)	T	378957	4095	375006	1.08	0.077286	0.07732	0.928	1.26	0.317528	0.738794
Operations and Procedures	Splenectomy (self-reported)	T	378957	791	378310	0.818	-0.20054	0.199661	0.553	1.21	0.315177	0.738794
Operations and Procedures	Rectal sigmoid resection (self-reported)	T	378957	534	378567	1.22	0.20195	0.20138	0.825	1.82	0.315941	0.738794
Operations and Procedures	Spinal cord surgery (self-reported)	T	378957	508	378593	0.775	-0.2546	0.254222	0.471	1.28	0.316599	0.738794
Neurosciences	Facial pains for 3 months vs no pain	T	368534	3287	365389	1.09	0.086533	0.086604	0.92	1.29	0.317707	0.738794
Operations and Procedures	Intrauterine contraceptive device	T	378957	5122	373979	0.928	-0.07504	0.075149	0.801	1.07	0.318025	0.738794
Operations and Procedures	Diagnostic imaging of pelvis	T	378957	1273	377828	1.14	0.134748	0.134736	0.879	1.49	0.317268	0.738794
Operations and Procedures	Chemical destruction of lesion of organ NOC	T	378957	228	378873	0.66	-0.41584	0.413969	0.293	1.49	0.315129	0.738794
Respiratory	Pleural effusion (HES and self-reported)	T	378957	4887	374214	1.07	0.071698	0.071386	0.934	1.24	0.3152	0.738794
Mental health	Insomnia (HES and self-reported)	T	378957	5225	373876	0.929	-0.07385	0.073958	0.803	1.07	0.318028	0.738794
Symptoms	Other symptoms and signs involving the urinary system (HES)	T	378957	4602	374499	1.08	0.073342	0.07368	0.931	1.24	0.319535	0.740866
Symptoms	Abnormal findings on diagnostic imaging of other body structures (HES)	T	378957	2807	376294	0.904	-0.10086	0.101287	0.741	1.1	0.319334	0.740866
Family history	Severe depression (family history - mother)	T	354043	23297	330884	1.03	0.033985	0.034205	0.967	1.11	0.320426	0.742218
Gynaecology and Obstetrics	Perineal laceration during delivery (HES)	T	378957	4945	374156	1.08	0.075045	0.075706	0.929	1.25	0.321552	0.743396
Operations and Procedures	Stabilisation of spine	T	378957	284	378817	1.3	0.263311	0.265564	0.773	2.19	0.321433	0.743396
Medication	Antiglaucoma preparations and miotics	T	378957	4850	374251	0.927	-0.07543	0.076206	0.799	1.08	0.322235	0.744261
Operations and Procedures	Late operations NOC	T	378957	5407	373694	1.07	0.067146	0.067951	0.936	1.22	0.323073	0.745482
Cancer	Carc in situ of cervix (cancer register)	T	378957	2991	376110	0.907	-0.09708	0.098486	0.748	1.1	0.32429	0.747574
Operations and Procedures	Rehabilitation for neurological disorders	T	378957	314	378787	1.29	0.252392	0.256879	0.778	2.13	0.325836	0.75042
Musculoskeletal	Other disorders of cartilage (HES)	T	378957	677	378424	1.19	0.177901	0.181376	0.837	1.7	0.32667	0.750905
Cardiovascular	Stroke (HES and self-reported)	T	378957	8571	370530	0.945	-0.05645	0.057533	0.844	1.06	0.326481	0.750905
Operations and Procedures	Therapeutic endoscopic operations on urethra	T	378957	2234	376867	1.11	0.101527	0.103712	0.903	1.36	0.327614	0.751681
Summary	Number of treatments medications taken	T	378896	NA	NA	NA	-0.00814	0.008313	-0.02443	0.008156	0.327632	0.751681

Summary	Disorders of vitreous body and globe (HES)	T		378957	1565	377536	1.13	0.117966	0.121105	0.887	1.43	0.330018	0.755305
Operations and Procedures	Ct scan (self-reported)	T		378957	307	378794	0.719	-0.32977	0.338535	0.37	1.4	0.33	0.755305
Gynaecology and Obstetrics	Miscarriage (HES and self-reported)	T		378957	1976	377125	0.888	-0.11878	0.121978	0.699	1.13	0.330152	0.755305
Summary	Infectious arthropathies (HES)	T		378957	428	378673	1.24	0.217938	0.224061	0.802	1.93	0.330715	0.755875
Summary	General symptoms and signs (HES)	T		378957	41556	337545	0.974	-0.02617	0.02694	0.924	1.03	0.331371	0.756657
Digestive system	Other diseases of jaws (HES)	T		378957	301	378800	1.29	0.257174	0.265152	0.769	2.17	0.33209	0.757551
Operations and Procedures	Abdominal pelvic adhesion surgery (self-reported)	T		378957	1192	377909	1.14	0.135277	0.139631	0.871	1.51	0.332633	0.757551
Musculoskeletal	Myositismyopathy (HES and self-reported)	T		378957	338	378763	0.733	-0.31115	0.321207	0.39	1.37	0.332706	0.757551
Operations and Procedures	Inguinal femoral hernia repair (self-reported)	T		378957	12611	366490	1.05	0.044741	0.046243	0.955	1.14	0.333285	0.758153
Gynaecology and Obstetrics	Breast cyst abscess removal (self-reported)	T		378957	3620	375481	1.08	0.080134	0.082931	0.921	1.27	0.333904	0.758844
ENT	Other diseases of inner ear (HES)	T		378957	734	378367	0.818	-0.20063	0.207779	0.545	1.23	0.334245	0.758903
Operations and Procedures	Primary repair of tendon	T		378957	2574	376527	0.902	-0.10286	0.106612	0.732	1.11	0.334654	0.759116
Operations and Procedures	Fusion of joint of toe	T		378957	3193	375908	0.913	-0.09138	0.094784	0.758	1.1	0.334986	0.759155
ENT	Labyrinthitis (HES and self-reported)	T		378957	839	378262	0.831	-0.18517	0.192463	0.57	1.21	0.33599	0.760714
Medication	Other systemic drugs for obstructive airway diseases	T		378957	21417	357684	1.03	0.0342	0.035574	0.965	1.11	0.33636	0.760837
Operations and Procedures	Other diagnostic tests	T		378957	994	378107	0.845	-0.16792	0.174826	0.6	1.19	0.336819	0.76116
Operations and Procedures	Excision of ganglion	T		378957	2429	376672	1.1	0.095975	0.100186	0.904	1.34	0.33808	0.762579
Musculoskeletal	Osteomyelitis (HES and self-reported)	T		378957	1062	378039	0.85	-0.16279	0.169851	0.609	1.19	0.337844	0.762579
Genitourinary	Other noninflammatory disorders of vagina (HES)	T		378957	1392	377709	0.87	-0.13947	0.145937	0.653	1.16	0.339229	0.763628
Operations and Procedures	Exploration of vagina	T		378957	1868	377233	0.887	-0.11946	0.125067	0.694	1.13	0.339496	0.763628
Neurosciences	Multiple sclerosis (HES and self-reported)	T		378957	1635	377466	1.12	0.114791	0.120128	0.886	1.42	0.339287	0.763628
Musculoskeletal	Acquired deformities of musculoskeletal (HES)	T		378957	329	378772	1.28	0.244685	0.256602	0.772	2.11	0.340306	0.763947
Operations and Procedures	Fixation of mandible	T		378957	258	378843	0.694	-0.36571	0.383372	0.327	1.47	0.340118	0.763947
Neurosciences	Paraplegia (HES and self-reported)	T		378957	312	378789	0.724	-0.32254	0.338448	0.373	1.41	0.340589	0.763947
Operations and Procedures	Excision of haemorrhoid	T		378957	2721	376380	1.09	0.09036	0.094932	0.909	1.32	0.341175	0.764549
Digestive system	Diseases of tongue (HES)	T		378957	1106	377995	1.15	0.137649	0.145035	0.864	1.52	0.342582	0.766277
Gynaecology and Obstetrics	Cervical polyps (HES and self-reported)	T		378957	3066	376035	1.09	0.08488	0.089424	0.914	1.3	0.342525	0.766277
Medication	Low ceiling diuretics thiazides	T		378957	23557	355544	0.967	-0.03333	0.035192	0.903	1.04	0.343648	0.76684
Medication	Anti acne preparations for topical use	T		378957	1608	377493	0.879	-0.12898	0.136324	0.673	1.15	0.344073	0.76684
Operations and Procedures	Lung removal pneumonectomy lobectomy (self-reported)	T		378957	614	378487	1.2	0.180299	0.190575	0.824	1.74	0.344107	0.76684
Operations and Procedures	Biopsy (self-reported)	T		378957	275	378826	0.712	-0.3397	0.358997	0.352	1.44	0.344016	0.76684
Musculoskeletal	Arthrosis of first carpometacarpal joint (HES)	T		378957	1179	377922	0.86	-0.15063	0.159338	0.629	1.18	0.344481	0.766964
Cardiovascular	Other acute ischaemic heart diseases (HES)	T		378957	1278	377823	1.14	0.128358	0.13602	0.871	1.48	0.345339	0.768164
Operations and Procedures	Reconstruction of nipple and areola	T		378957	637	378464	0.811	-0.20937	0.222271	0.525	1.25	0.346217	0.769407
Medication	Drugs for constipation	T		378957	6110	372991	0.938	-0.06351	0.06804	0.821	1.07	0.350581	0.770323
Mental health	Reaction to severe stress and adjustment disorders (HES)	T		378957	481	378620	0.783	-0.24407	0.26256	0.468	1.31	0.352597	0.770323
Respiratory	Chronic diseases of tonsils and adenoids (HES)	T		378957	904	378197	0.84	-0.17411	0.185996	0.584	1.21	0.349237	0.770323
Digestive system	Vascular disorders of intestine (HES)	T		378957	936	378165	0.845	-0.16837	0.180188	0.594	1.2	0.350089	0.770323
Digestive system	Other inflammatory liver diseases (HES)	T		378957	623	378478	0.809	-0.21181	0.227508	0.518	1.26	0.351847	0.770323
Immuno-inflammation	Other epidermal thickening (HES)	T		378957	608	378493	1.19	0.177149	0.190582	0.822	1.73	0.352623	0.770323
Musculoskeletal	Shoulder lesions (HES)	T		378957	8336	370765	1.05	0.051621	0.05538	0.945	1.17	0.351273	0.770323
Gynaecology and Obstetrics	False labour (HES)	T		378957	973	378128	1.15	0.143961	0.154015	0.854	1.56	0.349933	0.770323
Cancer	Basal cell carcinoma (self-reported)	T		378957	3552	375549	1.08	0.077179	0.082933	0.918	1.27	0.352048	0.770323
Family history	Stroke (family history - mother)	T		357389	49690	307839	0.977	-0.02337	0.025064	0.93	1.03	0.351118	0.770323
Other	Wheeze or whistling in the chest in last year	T		371840	77179	294800	0.981	-0.01961	0.020922	0.941	1.02	0.348591	0.770323
Operations and Procedures	Other adjustment to muscle of eye	T		378957	211	378890	1.34	0.289903	0.309934	0.728	2.45	0.349598	0.770323
Operations and Procedures	Total excision of bladder	T		378957	315	378786	1.27	0.241167	0.256971	0.769	2.11	0.347988	0.770323
Operations and Procedures	Introduction of substance into skin	T		378957	217	378884	0.679	-0.38665	0.41416	0.302	1.53	0.350523	0.770323
Operations and Procedures	Excision of muscle	T		378957	476	378625	0.783	-0.24419	0.262544	0.468	1.31	0.352325	0.770323
Medication	Multivitamins minerals	T		376892	81736	295300	0.981	-0.01895	0.020391	0.943	1.02	0.352705	0.770323
ENT	Chronic laryngitis (HES and self-reported)	T		378957	1014	378087	0.85	-0.16283	0.174758	0.603	1.2	0.351485	0.770323
Anthropometry	Total tissue fat percentage	T		4100	NA	NA	NA	0.080301	0.086035	-0.08833	0.248929	0.350639	0.770323
Biological assays	Haemoglobin concentration	T		367722	NA	NA	NA	0.007927	0.008438	-0.00861	0.024465	0.347518	0.770323
Cardiovascular	Congenital malformations of cardiac septa (HES)	T		378957	521	378580	1.21	0.190591	0.205245	0.809	1.81	0.353094	0.770474
Haematology	Other disorders of white blood cells (HES)	T		378957	309	378792	0.731	-0.31361	0.338494	0.376	1.42	0.354198	0.770789
Musculoskeletal	Enthesopathies of lower limb excluding foot (HES)	T		378957	393	378708	0.762	-0.27198	0.293381	0.429	1.35	0.353893	0.770789
Operations and Procedures	Stomach surgery (self-reported)	T		378957	2197	376904	0.898	-0.10794	0.116392	0.715	1.13	0.353738	0.770789
Digestive system	Sclerosing cholangitis (HES and self-reported)	T		378957	454	378647	1.22	0.202254	0.218872	0.797	1.88	0.355447	0.772112
ENT	Tonsiltis (HES and self-reported)	T		378957	3534	375567	0.921	-0.08271	0.089477	0.773	1.1	0.355303	0.772112
Metabolic	Other nontoxic goitre (HES)	T		378957	1508	377593	0.879	-0.12941	0.140227	0.667	1.16	0.356071	0.77277
Mental health	Other mental disorders due to brain damage (HES)	T		378957	212	378889	1.33	0.284753	0.309935	0.724	2.44	0.358226	0.775147
Symptoms	Unspecified urinary incontinence (HES)	T		378957	2162	376939	1.1	0.097315	0.105896	0.896	1.36	0.358113	0.775147
Operations and Procedures	Other operations on palate	T		378957	1051	378050	0.854	-0.15829	0.172229	0.609	1.2	0.358064	0.775147
Musculoskeletal	Fracture shoulder scapula (HES and self-reported)	T		378957	2722	376379	0.91	-0.09484	0.103273	0.743	1.11	0.358453	0.775147
Digestive system	Inguinal hernia (HES)	T		378957	14392	364709	1.04	0.040212	0.043831	0.955	1.13	0.358908	0.775434
Operations and Procedures	Vaginal operations to support outlet of female bladder	T		378957	3234	375867	0.917	-0.0863	0.094129	0.763	1.1	0.35924	0.775456
Operations and Procedures	Other total prosthetic replacement of knee joint	T		378957	844	378257	0.841	-0.17334	0.189325	0.58	1.22	0.359895	0.776175
Cardiovascular	Other conduction disorders (HES)	T		378957	1981	377120	0.896	-0.11011	0.120534	0.707	1.13	0.360989	0.777838
Medication	Low ceiling diuretics excl thiazides	T		378957	1449	377652	1.12	0.114893	0.126452	0.876	1.44	0.363569	0.77877
Neurosciences	Transient cerebral ischaemic attacks and related syndromes (HES)	T		378957	2348	376753	1.1	0.092282	0.101804	0.898	1.34	0.364689	0.77877
Cardiovascular	Other pulmonary heart diseases (HES)	T		378957	745	378356	1.17	0.157586	0.173533	0.833	1.64	0.363823	0.77877
Digestive system	Other diseases of gallbladder (HES)	T		378957	1619	377482	1.12	0.109754	0.120937	0.88	1.41	0.364125	0.77877
Musculoskeletal	Ankylosing spondylitis (HES)	T		378957	573	378528	1.2	0.178265	0.197443	0.812	1.76	0.366594	0.77877
Symptoms	Flatulence and related conditions (HES)	T		378957	1544	377557	0.882	-0.12541	0.138834	0.672	1.16	0.366382	0.77877
Cancer	Rodent ulcer (self-reported)	T		378957	540	378561	1.2	0.183215	0.201367	0.809	1.78	0.362898	0.77877
Operations and Procedures	Incision of breast	T		378957	615	378486	0.814	-0.20592	0.227665	0.521	1.27	0.365728	0.77877
Operations and Procedures	Operations on turbinate of nose	T		378957	2985	376116	1.09	0.082769	0.091485	0.908	1.3	0.365609	0.77877
Operations and Procedures	Repair of vault of vagina	T		378957	1152	377949	1.14	0.129768	0.142568	0.861	1.51	0.362705	0.77877
Operations and Procedures	Other operations on soft tissue	T		378957	1277	377824	1.13	0.122782	0.135825	0.866	1.48	0.36601	0.77877
Respiratory	Bronchiectasis (HES and self-reported)	T		378957	2109	376992	0.899	-0.10602	0.117298	0.715	1.13	0.366082	0.77877
Digestive system	Peptic ulcer (HES and self-reported)	T		378957	770	378331	0.832	-0.18407	0.20363	0.558	1.24	0.366016	0.77877
Digestive system	Crohns disease (HES and self-reported)	T		378957	1818	377283	1.11	0.103788	0.114695	0.886	1.39	0.365517	0.77877
Musculoskeletal	Fracture pelvis lower limb (HES and self-reported)	T		378957	8374	370727	1.05	0.050355	0.055378	0.943	1.17	0.363196	0.77877
Biological assays	Platelet distribution width	T		367716	NA	NA	NA	0.007636	0.00844	-0.00891	0.024177	0.365596	0.77877
ENT	Nonsuppurative otitis media (HES)	T		378957	959	378142	0.853	-0.15928	0.177414	0.602	1.21	0.369307	0.779449
Cardiovascular	Other venous embolism and thrombosis (HES)	T		378957	367	378734	1.24	0.216233	0.241947	0.773	1.99	0.37147	0.779449
ENT	Acute tonsillitis (HES)	T		378957	512	378589	0.796	-0.22765	0.254318	0.484	1.31	0.370722	0.779449
Immuno-inflammation	Other local infections of skin and subcutaneous tissue (HES)	T		378957	1106	377995	0.862	-0.14839	0.165363	0.623	1.19	0.369533	0.779449
Immuno-inflammation	Other disorders of pigmentation (HES)	T		378957	706	378395	1.17	0.159824	0.178579	0.827	1.67	0.370801	0.779449
Musculoskeletal	Other deforming dorsopathies (HES)	T		378957	1558	377543	0.885	-0.12207	0.13645	0.677	1.16	0.370983	0.779449
Gynaecology and Obstetrics	Prolonged pregnancy (HES)	T		378957	1102	377999	1.14	0.130477	0.14626	0.855	1.52	0.372344	0.779449
Medication	Hormones and related agents	T		378957	756	378345	1.17	0.154974	0.173394	0.831	1.64	0.371445	0.779449
Operations and Procedures	Bone scan (self-reported)	T		378957	213	378888	0.688	-0.37344	0.414403	0.306	1.55	0.367503	0.779449
Cancer	Multiple myeloma (cancer register)	T		378957	459	378642	1.22	0.195416	0.218821	0.792	1.87	0.371836	0.779449
Operations and Procedures	Other operations on lung	T		378957	801	378300	0.839	-0.17497	0.19608	0.572	1.23	0.372211	0.779449
Operations and Procedures	Other operations on perianal region	T		378957	1153	377948	1.14	0.128406	0.143574	0.858	1.51	0.371135	0.779449
Operations and Procedures	Other operations on uterus	T		378957	1103	377998	0.864	-0.14584	0.163503	0.627	1.19	0.372414	0.779449
Operations and Procedures	Other repair of organ NOC	T		378957	893	378208	1.15	0.142966	0.160186	0.843	1.58	0.372124	0.779449
Respiratory	Interstitial lung disease (HES and self-reported)	T		378957	1083	378018	1.14	0.131277	0.14665	0.855	1.52	0.370694	0.779449
Neurosciences	Epilepsy (HES and self-reported)	T		378957	4619	374482	1.07	0.065961	0.073862	0.924	1.23	0.371839	0.779449
Musculoskeletal	Gout (HES and self-reported)	T		378957	6858	372243	0.944	-0.05803	0.064662	0.831	1.07	0.36946	0.779449
Musculoskeletal	Other disorders of bone (HES)	T		378957	1394	377707	0.878	-0.1298	0.145728	0.66	1.17	0.373109	0.780225
Metabolic	Other disorders of fluid electrolyte and acid base balance (HES)	T		378957	4789	374312	0.934	-0.06828	0.076817	0.803	1.09	0.374096	0.780489
Genitourinary	Diseases of Bartholin s gland (HES)	T		378957	665	378436	1.18	0.164031	0.184453	0.821	1.69	0.37385	0.780489
Cardiovascular	LV end diastolic volume	T		3764	NA	NA	NA	0.079657	0.089641	-0.09604	0.255353	0.374207	0.780489
Medication	Vitamin b1 plain and in combination with vitamin b6 and b12	T		378957	3169	375932	0.918	-0.08504	0.095819	0.761	1.11	0.374823	0.781097
Summary	Diseases of middle ear and mastoid (HES)	T		378957	2903	376198	1.08	0.080721	0.092203	0.905	1.3	0.381316	0.78129
Cardiovascular	Other disorders of veins (HES)	T		378957	415	378686	0.781	-0.24763	0.282045	0.449	1.36	0.379955	0.78129
Respiratory	Pleural effusion in conditions classified elsewhere (HES)	T		378957	225	378876	0.694	-0.36572	0.414153	0.308	1.56	0.377202	0.78129
Symptoms	Senility (HES)	T		378957	414	378687	0.781	-0.24677	0.282107	0.449	1.36	0.381726	0.78129
Medication	Antimetabolites	T		378957	2405	376696	1.09	0.088433	0.100677	0.897	1.33	0.379735	0.78129
Cardiovascular	Heart surgery (self-reported)	T		378957	749	378352	0.837	-0.17842	0.203663	0.561	1.25	0.380994	0.78129
Operations and Procedures	Large bowel resection colostomy (self-reported)	T		378957	1039	378062	1.14	0.131162	0.14962	0.85	1.53	0.380683	0.78129
Operations and Procedures	Testicular hydrocoele surgery drainage (self-reported)	T		378957	1140	377961	1.14	0.127053	0.143878	0.856	1.51	0.377201	0.78129
Operations and Procedures	Upper limb surgery (self-reported)	T		378957	2145	376956	1.1	0.093817	0.10697	0.891	1.35	0.380467	0.78129
Gynaecology and Obstetrics	Mastoidectomy mastoid surgery (self-reported)	T		378957	700	378401	1.17	0.157567	0.178658	0.825	1.66	0.377803	0.78129
Operations and Procedures	Incision of eyelid	T		378957	241	378860	1.3	0.260078	0.296412	0.726	2.32	0.380258	0.78129

Operations and Procedures	Excision of sigmoid colon	T		378957	638	378463	0.822	-0.19653	0.222149	0.532	1.27	0.376327	0.78129
Operations and Procedures	Other operations on bladder	T		378957	1638	377463	1.11	0.105353	0.120183	0.878	1.41	0.3807	0.78129
Operations and Procedures	Debridement and irrigation of joint	T		378957	1526	377575	0.885	-0.12218	0.138865	0.674	1.16	0.378924	0.78129
Medication	Aspirin	T		374624	52613	322155	0.978	-0.02187	0.024968	0.932	1.03	0.381177	0.78129
Mental health	Schizophrenia (HES and self-reported)	T		378957	686	378415	1.17	0.161122	0.184126	0.819	1.69	0.38154	0.78129
Respiratory	Empyema (HES and self-reported)	T		378957	299	378802	1.26	0.232536	0.265236	0.75	2.12	0.380642	0.78129
Mental health	Postnatal depression (HES and self-reported)	T		378957	311	378790	1.26	0.227559	0.257128	0.759	2.08	0.376155	0.78129
Musculoskeletal	Other fractures (HES and self-reported)	T		378957	5304	373797	1.06	0.060394	0.069001	0.928	1.22	0.381431	0.78129
Musculoskeletal	L1 L4 BMD bone mineral density	T		4083	NA	NA	NA	-0.0763	0.086284	-0.24541	0.09282	0.376562	0.78129
Family history	Mother s age at death	T		222989	NA	NA	NA	0.009536	0.010759	-0.01155	0.030623	0.375435	0.78129
Musculoskeletal	Musclesoft tissue problem (HES and self-reported)	T		378957	32458	346643	1.03	0.025706	0.029489	0.968	1.09	0.38336	0.783968
Medication	Other cardiac preparations	T		378957	50254	328847	1.02	0.021302	0.024547	0.974	1.07	0.385497	0.787002
NA	Intracerebral haemorrhage	T		378957	792	378309	0.843	-0.17021	0.196112	0.574	1.24	0.385447	0.787002
Digestive system	Rectal prolapse (HES and self-reported)	T		378957	1030	378071	1.14	0.129783	0.149702	0.849	1.53	0.385975	0.787311
Gynaecology and Obstetrics	Unilateral oophorectomy (self-reported)	T		378957	2254	376847	1.09	0.08998	0.103952	0.892	1.34	0.386715	0.787855
Operations and Procedures	Fixation of rectum for prolapse	T		378957	293	378808	0.746	-0.29319	0.338842	0.384	1.45	0.386896	0.787855
Symptoms	Haemorrhage from respiratory passages (HES)	T		378957	4000	375101	1.07	0.068452	0.079209	0.917	1.25	0.387478	0.788374
Operations and Procedures	Diagnostic imaging of digestive tract	T		378957	243	378858	0.718	-0.33134	0.383697	0.338	1.52	0.387835	0.788434
Summary	HESCH Diseases of the skin and subcutaneous tissue (HES)	T		378957	37154	341947	1.02	0.023919	0.027826	0.97	1.08	0.390017	0.791503
Operations and Procedures	Other repair of prolapse of vagina	T		378957	7298	371803	1.05	0.051352	0.059719	0.936	1.18	0.389845	0.791503
Operations and Procedures	Other operations on fallopian tube	T		378957	950	378151	0.856	-0.15598	0.181581	0.599	1.22	0.39033	0.791503
Summary	Behav syn assoc with phys disturb and phys fact (HES)	T		378957	424	378677	1.22	0.19517	0.229356	0.775	1.91	0.394798	0.795294
Digestive system	Umbilical hernia (HES)	T		378957	3437	375664	0.926	-0.07706	0.090975	0.775	1.11	0.39699	0.795294
Musculoskeletal	Scoliosis (HES)	T		378957	942	378159	0.86	-0.15074	0.177552	0.607	1.22	0.395887	0.795294
Genitourinary	Hydrocele and spermatocele (HES)	T		378957	1221	377880	1.13	0.11889	0.139844	0.856	1.48	0.395234	0.795294
Gynaecology and Obstetrics	Unspecified maternal hypertension (HES)	T		378957	540	378561	1.19	0.171951	0.20308	0.798	1.77	0.397152	0.795294
Medication	Antiepileptics	T		378957	6896	372205	0.947	-0.05412	0.063918	0.836	1.07	0.397113	0.795294
Cancer	Car in situ other genital (cancer register)	T		378957	466	378635	0.8	-0.22315	0.262736	0.478	1.34	0.395696	0.795294
Operations and Procedures	Other therapeutic operations on oesophagus	T		378957	331	378770	1.24	0.218118	0.256561	0.752	2.06	0.395236	0.795294
Operations and Procedures	Catheterisation of heart	T		378957	776	378325	1.16	0.146051	0.171056	0.828	1.62	0.393207	0.795294
Operations and Procedures	Primary excision of cervical intervertebral disc	T		378957	805	378296	0.846	-0.16707	0.196002	0.576	1.24	0.393999	0.795294
Gynaecology and Obstetrics	Uterine polyps (HES and self-reported)	T		378957	8016	371085	1.05	0.048175	0.056808	0.939	1.17	0.396423	0.795294
ENT	Vocal cord polyp (HES and self-reported)	T		378957	392	378709	0.779	-0.24913	0.293404	0.439	1.39	0.395826	0.795294
Cardiovascular	Pericarditis (HES and self-reported)	T		378957	1193	377908	0.875	-0.13371	0.157431	0.643	1.19	0.395703	0.795294
Immuno-inflammation	Allergy to house dust mite (HES and self-reported)	T		378957	279	378822	1.26	0.232416	0.274489	0.737	2.16	0.397149	0.795294
Cardiovascular	QRS duration	T		8938	NA	NA	NA	0.048524	0.057018	-0.06323	0.160279	0.394755	0.795294
Medication	Antivertigo preparations	T		378957	1504	377597	1.11	0.106708	0.126288	0.869	1.43	0.398131	0.796593
Gynaecology and Obstetrics	Ectopic pregnancy (HES)	T		378957	264	378837	0.739	-0.30242	0.360181	0.365	1.5	0.401109	0.797912
Symptoms	Cardiac murmurs and other cardiac sounds (HES)	T		378957	965	378136	1.14	0.130078	0.154641	0.841	1.54	0.400257	0.797912
Operations and Procedures	Other operations on retina	T		378957	498	378603	0.807	-0.21418	0.254384	0.49	1.33	0.399819	0.797912
Operations and Procedures	Therapeutic transluminal operations on heart	T		378957	862	378239	0.855	-0.15647	0.186172	0.594	1.23	0.40066	0.797912
Operations and Procedures	Repair of recurrent inguinal hernia	T		378957	1175	377926	1.13	0.118965	0.141286	0.854	1.49	0.39978	0.797912
Digestive system	Inflammatory bowel disease (HES and self-reported)	T		378957	4246	374855	1.07	0.064541	0.076864	0.917	1.24	0.401084	0.797912
Musculoskeletal	Spine BMD bone mineral density	T		4100	NA	NA	NA	-0.07259	0.0863	-0.24174	0.096561	0.400293	0.797912
Symptoms	Unspecified haematuria (HES)	T		378957	14238	364863	1.04	0.036207	0.043183	0.953	1.13	0.401774	0.798576
Digestive system	Crohn s disease (HES)	T		378957	1547	377554	1.11	0.104036	0.124351	0.87	1.42	0.402797	0.79929
Operations and Procedures	Examination of organ NOC	T		378957	581	378520	1.18	0.165214	0.197353	0.801	1.74	0.402507	0.79929
Medication	Vasodilators used in cardiac diseases	T		378957	5385	373716	0.941	-0.06079	0.072844	0.816	1.09	0.404009	0.800376
Operations and Procedures	Soft tissue operations on joint of toe	T		378957	2721	376380	0.918	-0.08558	0.102494	0.751	1.12	0.403718	0.800376
Summary	Disorders of skin appendages (HES)	T		378957	9390	369711	1.04	0.04392	0.052751	0.942	1.16	0.405068	0.800792
Operations and Procedures	Excision of other artery	T		378957	498	378603	1.19	0.174493	0.209641	0.789	1.8	0.405216	0.800792
Operations and Procedures	Other destruction of lesion of skin of head or neck	T		378957	325	378776	0.765	-0.26774	0.321411	0.408	1.44	0.404836	0.800792
Operations and Procedures	Elective caesarean delivery	T		378957	1697	377404	1.11	0.10032	0.120658	0.873	1.4	0.405725	0.801141
Summary	HESCH Chapter IX Diseases of the circulatory system BIN (HES)	T		378957	117038	262063	0.985	-0.01552	0.018743	0.949	1.02	0.407616	0.802272
ENT	Perforation of tympanic membrane (HES)	T		378957	870	378231	1.15	0.135699	0.164123	0.83	1.58	0.408343	0.802272
Mental health	Seen a psychiatrist for nerves anxiety tension or depression	T		377176	43632	333688	0.978	-0.0218	0.026309	0.929	1.03	0.407366	0.802272
Operations and Procedures	Extirpation of lesion of penis	T		378957	221	378880	1.29	0.256084	0.309696	0.704	2.37	0.4083	0.802272
Operations and Procedures	Freeing of tendon	T		378957	652	378449	1.17	0.154845	0.187195	0.809	1.68	0.408131	0.802272
Infectious disease	Mumps epidemic parotitis (HES and self-reported)	T		378957	830	378271	1.15	0.136075	0.164347	0.83	1.58	0.407688	0.802272
Eye	Corneal resistance factor left	T		81021	NA	NA	NA	-0.01469	0.017774	-0.04952	0.02015	0.408629	0.802272
Medication	Antibiotics for topical use	T		378957	1432	377669	1.11	0.106756	0.130165	0.862	1.44	0.412127	0.803122
Medication	Corticosteroids other combinations	T		378957	9688	369413	1.04	0.041813	0.051814	0.942	1.15	0.41968	0.803122
Summary	Other osteopathies (HES)	T		378957	2866	376235	0.921	-0.08217	0.099805	0.757	1.12	0.410319	0.803122
Summary	Congenital malformations of genital organs (HES)	T		378957	628	378473	0.836	-0.17957	0.222194	0.541	1.29	0.418985	0.803122
Eye	Disorders of refraction and accommodation (HES)	T		378957	1639	377462	1.1	0.097707	0.120161	0.871	1.4	0.416142	0.803122
Respiratory	Diseases of vocal cords and larynx (HES)	T		378957	1561	377540	0.895	-0.11095	0.137606	0.683	1.17	0.420064	0.803122
Immuno-inflammation	Cellulitis (HES)	T		378957	6570	372531	0.948	-0.05317	0.065368	0.834	1.08	0.415995	0.803122
Genitourinary	Redundant prepuce phimosis and paraphimosis (HES)	T		378957	1890	377211	1.1	0.093442	0.114975	0.876	1.38	0.416381	0.803122
Gynaecology and Obstetrics	Gestational hypertension with significant proteinuria (HES)	T		378957	289	378812	0.758	-0.27767	0.339916	0.389	1.47	0.413991	0.803122
Gynaecology and Obstetrics	Retained placenta and membranes without haemorrhage (HES)	T		378957	203	378898	0.715	-0.33508	0.415398	0.317	1.61	0.419873	0.803122
Medication	Throat preparations	T		378957	49826	329275	1.02	0.020159	0.024656	0.972	1.07	0.413566	0.803122
Medication	Mydriatics and cycloplegics	T		378957	287	378814	1.25	0.221605	0.274272	0.729	2.14	0.419105	0.803122
Gynaecology and Obstetrics	Gynaecological surgery (self-reported)	T		378957	2168	376933	1.09	0.086545	0.105584	0.887	1.34	0.412397	0.803122
Operations and Procedures	Cystoscopy (self-reported)	T		378957	345	378756	0.779	-0.24989	0.30668	0.427	1.42	0.415181	0.803122
Operations and Procedures	Operations on lower respiratory tract	T		378957	209	378892	0.716	-0.33421	0.414465	0.318	1.61	0.420038	0.803122
Operations and Procedures	Attention to artificial opening into ileum	T		378957	998	378103	1.13	0.124342	0.152884	0.839	1.53	0.416041	0.803122
Operations and Procedures	Other exteriorisation of colon	T		378957	934	378167	1.14	0.130043	0.158224	0.835	1.55	0.411141	0.803122
Operations and Procedures	Diagnostic endoscopic retrograde examination of bile duct	T		378957	309	378792	1.24	0.215174	0.264968	0.738	2.08	0.41675	0.803122
Operations and Procedures	Endoscopic ultrasound examination of pancreas	T		378957	605	378496	1.17	0.158159	0.193799	0.801	1.71	0.414446	0.803122
Operations and Procedures	Other operations on vena cava	T		378957	207	378894	0.713	-0.33851	0.414446	0.316	1.61	0.414052	0.803122
Operations and Procedures	Transplantation of kidney	T		378957	266	378835	1.26	0.233822	0.284642	0.723	2.21	0.411384	0.803122
Operations and Procedures	Other operations on nail bed	T		378957	686	378415	0.84	-0.17414	0.212353	0.554	1.27	0.412201	0.803122
Operations and Procedures	Image controlled operations on abdominal cavity	T		378957	419	378682	1.2	0.185574	0.22942	0.768	1.89	0.418583	0.803122
Operations and Procedures	Biopsy of muscle	T		378957	421	378680	1.21	0.187043	0.229351	0.769	1.89	0.414767	0.803122
Operations and Procedures	Diagnostic endocrinology	T		378957	1212	377889	0.881	-0.12686	0.157307	0.647	1.2	0.419989	0.803122
Operations and Procedures	Other operations on intervertebral disc	T		378957	298	378803	0.759	-0.27531	0.338683	0.391	1.47	0.416283	0.803122
Operations and Procedures	Anaesthetic without surgery	T		378957	299	378802	0.76	-0.27404	0.33869	0.391	1.48	0.41844	0.803122
NA	Stroke	T		378957	8703	370398	0.955	-0.0459	0.056857	0.854	1.07	0.419486	0.803122
Cardiovascular	Pericardial problem (HES and self-reported)	T		378957	1268	377833	0.884	-0.12362	0.152131	0.656	1.19	0.416448	0.803122
Neurosciences	Spinal cord disorder (HES and self-reported)	T		378957	1886	377215	0.905	-0.10016	0.12385	0.71	1.15	0.418692	0.803122
ENT	Nasalsinus disorder (HES and self-reported)	T		378957	11324	367777	1.04	0.0395	0.048311	0.946	1.14	0.413578	0.803122
Gynaecology and Obstetrics	Ectopic pregnancy (HES and self-reported)	T		378957	539	378562	0.82	-0.19788	0.240266	0.512	1.31	0.410183	0.803122
NA	Pediatric asthma under16yo (self-reported)	T		350450	15307	335270	1.03	0.033945	0.041813	0.953	1.12	0.416889	0.803122
Summary	Other nutritional deficiencies (HES)	T		378957	1249	377852	1.12	0.111329	0.138224	0.852	1.47	0.420577	0.803465
ENT	Ent surgery (self-reported)	T		378957	283	378818	1.25	0.220726	0.274376	0.728	2.14	0.421128	0.803881
Haematology	Anaemia in chronic diseases classified elsewhere (HES)	T		378957	657	378444	1.16	0.150093	0.187197	0.805	1.68	0.422674	0.804283
Neurosciences	Paraplegia and tetraplegia (HES)	T		378957	297	378804	0.762	-0.27207	0.338706	0.392	1.48	0.421823	0.804283
Digestive system	Alcoholic liver disease (HES)	T		378957	799	378302	1.15	0.138943	0.173287	0.818	1.61	0.422665	0.804283
Operations and Procedures	Diagnostic transluminal operations on heart	T		378957	1107	377994	0.877	-0.13105	0.163323	0.637	1.21	0.422318	0.804283
Cancer	Mal neo ovary (cancer register)	T		378957	893	378208	0.864	-0.14674	0.183251	0.603	1.24	0.423262	0.804513
Operations and Procedures	Primary repair of incisional hernia	T		378957	1622	377479	1.1	0.097436	0.121729	0.868	1.4	0.423463	0.804513
Gynaecology and Obstetrics	Other abnormal products of conception (HES)	T		378957	1191	377910	0.882	-0.12565	0.157316	0.648	1.2	0.424468	0.80468
Symptoms	Abnormality of white blood cells (HES)	T		378957	236	378865	0.736	-0.30655	0.383882	0.347	1.56	0.424553	0.80468
Operations and Procedures	Antireflux operations	T		378957	820	378281	0.857	-0.1538	0.192532	0.588	1.25	0.424391	0.80468
Digestive system	Postprocedural disorders of digestive system (HES)	T		378957	1429	377672	1.11	0.102968	0.129209	0.86	1.43	0.425504	0.80518
Musculoskeletal	Other disorders of synovium and tendon (HES)	T		378957	3447	375654	1.07	0.067664	0.085027	0.906	1.26	0.426154	0.80518
Gynaecology and Obstetrics	Laser treatment cervix (self-reported)	T		378957	611	378490	0.838	-0.17717	0.222495	0.542	1.3	0.425861	0.80518
Family history	Breast cancer (family history - mother)	T		350478	12118	338496	1.04	0.036975	0.046415	0.947	1.14	0.42567	0.80518
Musculoskeletal	Other crystal arthropathies (HES)	T		378957	369	378732	0.792	-0.23357	0.293749	0.445	1.41	0.426538	0.805274
Other	Pattern 4 f	T		173207	31573	141706	0.975	-0.02569	0.032337	0.915	1.04	0.426952	0.805425
Operations and Procedures	Peripheral nerve surgery (self-reported)	T		378957	794	378307	1.15	0.135612	0.17085	0.819	1.6	0.427343	0.805532
Operations and Procedures	Operations on retinal membrane	T		378957	983	378118	1.13	0.121279	0.153022	0.836	1.52	0.428036	0.805577
Digestive system	Alcoholic liver disease alcoholic cirrhosis (HES and self-reported)	T		378957	824	378277	1.15	0.135462	0.170852	0.819	1.6	0.427859	0.805577
Medication	Other plain vitamin preparations	T		378957	4840	374261	0.941	-0.06068	0.076719	0.81	1.09	0.429014	0.806488
Mental health	Delirium not induced by alcohol and other psychoactive substances (HES)	T		378957	538	378563	0.827	-0.18955	0.240103	0.517	1.32	0.429859	0.806488
Operations and Procedures	Percutaneous examination of bile duct	T		378957	260	378841	1.25	0.224923	0.284842	0.717	2.19	0.429738	0.806488
Operations and Procedures	Operations on epididymis	T		378957	1069	378032	1.13	0.118327	0.149792	0.839	1.51	0.429563	0.806488

Operations and Procedures	Other operations on vulva	T		378957	1300	377801	0.888	-0.11879	0.150627	0.661	1.19	0.430331	0.806745
Musculoskeletal	Other rheumatoid arthritis (HES)	T		378957	3989	375112	1.06	0.06258	0.079423	0.911	1.24	0.430735	0.806875
Digestive system	Irritable bowel syndrome (HES and self-reported)	T		378957	12379	366722	1.04	0.036314	0.046156	0.947	1.14	0.431426	0.807541
Symptoms	Abnormalities of breathing (HES)	T		378957	8413	370688	0.956	-0.0454	0.057866	0.853	1.07	0.432715	0.808614
Medication	Hepatic and reticulo endothelial system	T		378957	1728	377373	0.903	-0.10178	0.129709	0.7	1.16	0.432636	0.808614
Musculoskeletal	Fracture thumb (HES and self-reported)	T		378957	379	378722	0.794	-0.23019	0.293593	0.447	1.41	0.433006	0.808614
Operations and Procedures	Excision of vulva	T		378957	1147	377954	1.12	0.112538	0.143771	0.844	1.48	0.433377	0.809413
Gynaecology and Obstetrics	Infections of genitourinary tract in pregnancy (HES)	T		378957	212	378889	1.28	0.243507	0.311363	0.693	2.35	0.434174	0.80954
ENT	Other disorders of tympanic membrane (HES)	T		378957	333	378768	1.22	0.200329	0.256463	0.739	2.02	0.43473	0.809949
Neurosciences	Parkinsons disease (HES and self-reported)	T		378957	1191	377910	0.886	-0.12149	0.15579	0.653	1.2	0.435507	0.81077
Summary	Disorders of adult personality and behaviour (HES)	T		378957	350	378751	0.788	-0.23873	0.306594	0.432	1.44	0.43619	0.811414
Medication	Antiobesity preparations excl diet products	T		378957	958	378143	0.871	-0.13798	0.177427	0.615	1.23	0.436755	0.811839
Summary	Abnormal findings on examination of blood without diagnosis (HES)	T		378957	7295	371806	1.05	0.046165	0.059543	0.932	1.18	0.438157	0.812897
Mental health	Recurrent depressive disorder (HES)	T		378957	679	378422	0.849	-0.1642	0.212368	0.56	1.29	0.43942	0.812897
Gynaecology and Obstetrics	Maternal care for other known or suspected fetal problems (HES)	T		378957	1716	377385	1.1	0.092721	0.120191	0.867	1.39	0.44044	0.812897
Operations and Procedures	Urethral stricture surgery dilatation (self-reported)	T		378957	205	378896	0.726	-0.32003	0.414518	0.322	1.64	0.440087	0.812897
Mental health	Probable recurrent major depression severe 2	T		54180	7072	47141	1.05	0.04991	0.064356	0.927	1.19	0.438026	0.812897
Cancer	Mal neo stomach (cancer register)	T		378957	377	378724	1.21	0.187013	0.241897	0.75	1.94	0.439456	0.812897
Operations and Procedures	Exteriorisation of trachea	T		378957	817	378284	0.862	-0.1485	0.192606	0.591	1.26	0.440699	0.812897
Operations and Procedures	Other repair of vagina	T		378957	332	378769	1.22	0.198051	0.256617	0.737	2.02	0.440246	0.812897
Gynaecology and Obstetrics	Uterine problem (HES and self-reported)	T		378957	29689	349412	1.02	0.024529	0.031746	0.963	1.09	0.439733	0.812897
ENT	Speech reception threshold SRT estimate right	T		114757	NA	NA	NA	0.011547	0.014905	-0.01767	0.040761	0.438529	0.812897
Genitourinary	Unspecified renal colic (HES)	T		378957	1752	377349	1.1	0.091235	0.118427	0.869	1.38	0.441069	0.812957
Summary	Disorders of breast (HES)	T		378957	4857	374244	0.943	-0.05853	0.076171	0.812	1.1	0.442236	0.813107
Metabolic	Vitamin D deficiency (HES)	T		378957	359	378742	1.21	0.19115	0.248753	0.743	1.97	0.442231	0.813107
Digestive system	Other diseases of digestive system (HES)	T		378957	8428	370673	0.957	-0.0444	0.057756	0.854	1.07	0.442056	0.813107
Musculoskeletal	Disorders of continuity of bone (HES)	T		378957	1398	377703	1.11	0.100789	0.131241	0.855	1.43	0.442506	0.813107
Medication	Antiinflammatory agents	T		378957	18165	360936	0.97	-0.03032	0.039503	0.898	1.05	0.442838	0.813107
Summary	Hernia (HES)	T		378957	42709	336392	1.02	0.020299	0.026485	0.969	1.07	0.443411	0.81327
Musculoskeletal	Prolapsed discslipped disc (HES and self-reported)	T		378957	8220	370881	1.04	0.043001	0.056128	0.935	1.17	0.443602	0.81327
Musculoskeletal	Psoriatic and enteropathic arthropathies (HES)	T		378957	650	378451	1.16	0.145149	0.190249	0.796	1.68	0.445496	0.813647
Family history	Alzheimer s disease dementia (family history - mother)	T		357389	29815	327714	0.976	-0.02393	0.031341	0.918	1.04	0.44518	0.813647
Operations and Procedures	Other repair of peripheral nerve	T		378957	526	378575	0.828	-0.18882	0.246758	0.51	1.34	0.444164	0.813647
Operations and Procedures	Shave biopsy of skin	T		378957	626	378475	1.16	0.145561	0.190436	0.796	1.68	0.444657	0.813647
Eye	Retinal arteryvein occlusion (HES and self-reported)	T		378957	614	378487	0.844	-0.16977	0.222364	0.546	1.3	0.445184	0.813647
Genitourinary	Glomerulnephritis (HES and self-reported)	T		378957	1746	377355	1.09	0.089719	0.117705	0.869	1.38	0.445923	0.813809
Operations and Procedures	Transluminal operations on cerebral artery	T		378957	1001	378100	1.12	0.117588	0.154474	0.831	1.52	0.44653	0.8143
Operations and Procedures	Open excision of prostate	T		378957	2046	377055	0.915	-0.08905	0.117187	0.727	1.15	0.447341	0.815162
Respiratory	Cough on most days	T		95656	13048	82648	0.964	-0.03658	0.048219	0.877	1.06	0.448018	0.815163
Digestive system	Gastrointestinal bleeding (HES and self-reported)	T		378957	8293	370808	0.957	-0.04419	0.058206	0.854	1.07	0.447779	0.815163
Neurosciences	Mononeuropathy in diseases classified elsewhere (HES)	T		378957	205	378896	0.731	-0.31399	0.414587	0.324	1.65	0.448831	0.81541
Operations and Procedures	Examination of oesophagus	T		378957	1296	377805	0.891	-0.11522	0.152048	0.662	1.2	0.448567	0.81541
Operations and Procedures	Manipulation of spine	T		378957	218	378883	1.26	0.233639	0.30967	0.688	2.32	0.450562	0.817938
Gynaecology and Obstetrics	Dilatation and curettage d c (self-reported)	T		378957	21385	357716	1.03	0.027513	0.0365	0.957	1.1	0.450994	0.818106
Operations and Procedures	Extracorporeal fragmentation of calculus of ureter	T		378957	563	378538	0.839	-0.17588	0.233703	0.531	1.33	0.451715	0.818182
Biological assays	Urine albumin creatinine ratio	T		114243	NA	NA	NA	0.011187	0.014856	-0.01793	0.040305	0.451414	0.818182
Medication	Antithyroid preparations	T		378957	334	378767	1.21	0.190023	0.256478	0.731	2	0.458759	0.818629
Summary	Deforming dorsopathies (HES)	T		378957	2539	376562	0.924	-0.07881	0.10503	0.752	1.14	0.453036	0.818629
Neurosciences	Parkinson s disease (HES)	T		378957	1054	378047	0.884	-0.12283	0.165668	0.639	1.22	0.458441	0.818629
Neurosciences	Migraine (HES)	T		378957	2516	376585	0.925	-0.07835	0.106105	0.751	1.14	0.460268	0.818629
Eye	Other disorders of optic (HES)	T		378957	285	378816	0.776	-0.25404	0.338935	0.399	1.51	0.453535	0.818629
Cardiovascular	Other disorders of arteries and arterioles (HES)	T		378957	1296	377805	0.895	-0.11128	0.150569	0.666	1.2	0.459884	0.818629
Digestive system	Peptic ulcer site unspecified (HES)	T		378957	370	378731	1.2	0.184853	0.24863	0.739	1.96	0.457187	0.818629
Immuno-inflammation	Corns and callosities (HES)	T		378957	257	378844	0.766	-0.26654	0.359425	0.379	1.55	0.458353	0.818629
Symptoms	Cough (HES)	T		378957	2614	376487	1.07	0.071918	0.097434	0.888	1.3	0.46044	0.818629
Symptoms	Headache (HES)	T		378957	6991	372110	0.954	-0.04757	0.063342	0.842	1.08	0.452653	0.818629
Cancer	Malignant melanoma (self-reported)	T		378957	2994	376107	1.07	0.067049	0.090802	0.895	1.28	0.460266	0.818629
Gynaecology and Obstetrics	Uterine surgery (self-reported)	T		378957	1594	377507	0.905	-0.09956	0.134296	0.696	1.18	0.458496	0.818629
Family history	High blood pressure (family history - father)	T		343458	74698	268894	0.984	-0.01591	0.021463	0.944	1.03	0.458457	0.818629
Operations and Procedures	Repair of eardrum	T		378957	1054	378047	1.12	0.111819	0.151064	0.832	1.5	0.459175	0.818629
Operations and Procedures	Excision of oesophagus and stomach	T		378957	219	378882	1.26	0.229921	0.309744	0.686	2.31	0.457909	0.818629
Operations and Procedures	Excision of ileum	T		378957	732	378369	1.14	0.131969	0.178434	0.804	1.62	0.459544	0.818629
Operations and Procedures	Destruction of haemorrhoid	T		378957	5384	373717	1.05	0.051457	0.069405	0.919	1.21	0.458453	0.818629
Operations and Procedures	Other evacuation of contents of uterus	T		378957	2583	376518	0.924	-0.07903	0.107177	0.749	1.14	0.46091	0.818629
Operations and Procedures	Primary open reduction of intra articular fracture of bone	T		378957	292	378809	0.777	-0.25172	0.338788	0.4	1.51	0.457488	0.818629
Operations and Procedures	Other interposition reconstruction of joint	T		378957	307	378794	0.787	-0.23981	0.321795	0.419	1.48	0.456134	0.818629
Operations and Procedures	Delivery of oral chemotherapy for neoplasm	T		378957	560	378541	0.84	-0.17392	0.233636	0.532	1.33	0.456638	0.818629
Operations and Procedures	Other destruction of lesion of organ NOC	T		378957	2552	376549	0.925	-0.07821	0.104954	0.753	1.14	0.456174	0.818629
Respiratory	Pleurisy (HES and self-reported)	T		378957	1687	377414	0.908	-0.0964	0.130737	0.703	1.17	0.460919	0.818629
Neurosciences	Dementiaalzheimerscognitive impairment (HES and self-reported)	T		378957	906	378195	1.13	0.119318	0.160402	0.823	1.54	0.456956	0.818629
Musculoskeletal	Sclerodermasystemic sclerosis (HES and self-reported)	T		378957	201	378900	0.736	-0.30698	0.414732	0.326	1.66	0.459186	0.818629
Immuno-inflammation	Cellulitis (HES and self-reported)	T		378957	6668	372433	0.953	-0.04771	0.064738	0.84	1.08	0.461137	0.818629
Neurosciences	Incorect matches summed	T		378957	NA	NA	NA	-0.00621	0.00831	-0.0225	0.010078	0.454968	0.818629
Operations and Procedures	Continuous Infusion of therapeutic substance	T		378957	8393	370708	0.958	-0.0426	0.057953	0.855	1.07	0.462257	0.820013
Medication	Stomatological preparations	T		378957	59623	319478	0.983	-0.01696	0.023653	0.939	1.03	0.473298	0.821031
Medication	Iron preparations	T		378957	19982	359119	0.973	-0.0277	0.037806	0.903	1.05	0.463792	0.821031
Summary	Extrapyramidal and movement disorders (HES)	T		378957	1814	377287	0.914	-0.09013	0.124927	0.715	1.17	0.470621	0.821031
Summary	Symptoms and signs involving the digestive system and abdomen (HES)	T		378957	52090	327011	1.02	0.017657	0.024145	0.971	1.07	0.464598	0.821031
Mental health	Somatoform disorders (HES)	T		378957	381	378720	1.19	0.176518	0.241721	0.743	1.92	0.465235	0.821031
Neurosciences	Inflammatory polyneuropathy (HES)	T		378957	268	378833	1.23	0.207329	0.284593	0.704	2.15	0.466301	0.821031
Neurosciences	Hydrocephalus (HES)	T		378957	402	378699	1.19	0.172602	0.235283	0.749	1.88	0.463197	0.821031
Digestive system	Gastric ulcer (HES)	T		378957	3863	375238	0.94	-0.06175	0.085461	0.795	1.11	0.469945	0.821031
Digestive system	Femoral hernia (HES)	T		378957	558	378543	0.845	-0.16804	0.233687	0.535	1.34	0.472087	0.821031
Immuno-inflammation	Granulomatous disorders of skin and subcutaneous tissue (HES)	T		378957	534	378567	0.841	-0.17296	0.239999	0.526	1.35	0.471126	0.821031
Genitourinary	Orchitis and epididymitis (HES)	T		378957	769	378332	0.866	-0.144	0.199976	0.585	1.28	0.471477	0.821031
Medication	Combinations of antibacterials	T		378957	595	378506	0.849	-0.16342	0.227685	0.544	1.33	0.472906	0.821031
ENT	Nasal sinus nose surgery (self-reported)	T		378957	13231	365870	1.03	0.032384	0.044961	0.946	1.13	0.471359	0.821031
Operations and Procedures	Radical prostatectomy (self-reported)	T		378957	1237	377864	0.896	-0.10932	0.151257	0.666	1.21	0.469853	0.821031
Operations and Procedures	Femoral hernia repair (self-reported)	T		378957	326	378775	1.2	0.185042	0.256659	0.728	1.99	0.470932	0.821031
Family history	Alzheimer s disease dementia (family history - father)	T		343458	15771	327821	0.97	-0.03024	0.042208	0.893	1.05	0.473759	0.821031
Mental health	Probable Recurrent major depression moderate	T		91467	11528	79987	0.964	-0.03718	0.051728	0.871	1.07	0.472266	0.821031
Cancer	Mal neo colon (cancer register)	T		378957	2575	376526	0.927	-0.07604	0.104545	0.755	1.14	0.466999	0.821031
Cancer	Neo of lymphoid haematopoietic (cancer register)	T		378957	292	378809	1.22	0.19872	0.274192	0.713	2.09	0.468606	0.821031
Operations and Procedures	Partial excision of ovary	T		378957	433	378668	1.18	0.165152	0.229563	0.752	1.85	0.471884	0.821031
Operations and Procedures	Opening of chest	T		378957	257	378844	0.773	-0.25734	0.359485	0.382	1.56	0.474078	0.821031
Operations and Procedures	Puncture of pleura	T		378957	2582	376519	1.07	0.071529	0.097939	0.887	1.3	0.465178	0.821031
Operations and Procedures	Primary fusion of other joint of spine	T		378957	465	378636	0.833	-0.18295	0.254655	0.506	1.37	0.472503	0.821031
Operations and Procedures	Secondary reduction of traumatic dislocation of joint	T		378957	235	378866	0.759	-0.2761	0.383893	0.358	1.61	0.472009	0.821031
Operations and Procedures	Puncture of organ NOC	T		378957	315	378786	1.21	0.192069	0.264816	0.721	2.04	0.468271	0.821031
Operations and Procedures	Spinal anaesthetic	T		378957	6624	372477	1.05	0.045754	0.062557	0.926	1.18	0.464542	0.821031
Operations and Procedures	Other anaesthetic	T		378957	5945	373156	0.951	-0.04982	0.06821	0.832	1.09	0.465107	0.821031
Mental health	Ever manic hyper for 2 days	T		123436	7031	116467	0.955	-0.04595	0.064104	0.842	1.08	0.473497	0.821031
Infectious disease	Tuberculosis tb (HES and self-reported)	T		378957	1905	377196	1.09	0.082341	0.113386	0.869	1.36	0.467712	0.821031
Immuno-inflammation	Food intolerance (HES and self-reported)	T		378957	306	378795	1.21	0.191526	0.265035	0.72	2.04	0.4699	0.821031
Cardiovascular	Mitral valve disease (HES and self-reported)	T		378957	3385	375716	1.06	0.062529	0.085805	0.9	1.26	0.466166	0.821031
Musculoskeletal	Bursitis (HES and self-reported)	T		378957	2169	376932	0.921	-0.08258	0.114281	0.736	1.15	0.469905	0.821031
Anthropometry	Waist circumference	T		378288	NA	NA	NA	-0.00598	0.008324	-0.0223	0.010333	0.472289	0.821031
Medication	Selective calcium channel blockers with direct cardiac effects	T		378957	3557	375544	0.939	-0.06339	0.088723	0.789	1.12	0.474957	0.821372
Cardiovascular	Intracerebral haemorrhage (HES)	T		378957	660	378441	0.859	-0.15195	0.212547	0.566	1.3	0.47468	0.821372
Cancer	Mal neo bronchus and lung (cancer register)	T		378957	1742	377359	1.09	0.084611	0.118614	0.863	1.37	0.475637	0.821958
Cancer	Colon cancer sigmoid cancer (self-reported)	T		378957	1220	377881	1.1	0.099492	0.139656	0.84	1.45	0.476213	0.822364
Operations and Procedures	Transurethral resection of prostate turp (self-reported)	T		378957	1932	377169	1.08	0.079364	0.111914	0.869	1.35	0.478229	0.824004
Operations and Procedures	Reconstruction of eyelid	T		378957	382	378719	1.19	0.171481	0.241735	0.739	1.91	0.478093	0.824004
Operations and Procedures	Other operations on pharynx	T		378957	503	378598	0.839	-0.175	0.246943	0.517	1.36	0.478531	0.824004
Operations and Procedures	Plastic excision of skin of abdominal wall	T		378957	228	378873	1.25	0.219723	0.30941	0.679	2.28	0.477619	0.824004
Eye	Glaucoma (HES)	T		378957	4047	375054	1.06	0.055221	0.07841	0.906	1.23	0.481275	0.825245
Symptoms	Convulsions (HES)	T		378957	1802	377299	1.09	0.082924	0.117572	0.863	1.37	0.480621	0.825245

Medication	Antimycotics for systemic use	T	378957	389	378712	1.19	0.170292	0.241596	0.738	1.9	0.480897	0.825245
Operations and Procedures	Nephrectomy kidney removed (self-reported)	T	378957	1353	377748	1.1	0.093983	0.13346	0.846	1.43	0.481307	0.825245
Cardiovascular	Ecg electrocardiogram (self-reported)	T	378957	201	378900	0.747	-0.29229	0.414617	0.331	1.68	0.48084	0.825245
Metabolic	Gestational diabetes (HES and self-reported)	T	378957	363	378738	0.813	-0.20747	0.294019	0.457	1.45	0.480424	0.825245
Summary	Glaucoma (HES)	T	378957	4048	375053	1.06	0.054909	0.07841	0.906	1.23	0.48375	0.825321
Neurosciences	Hemiplegia (HES)	T	378957	1363	377738	1.1	0.093584	0.133451	0.845	1.43	0.483143	0.825321
Family history	Severe depression (family history - father)	T	338597	12638	326091	1.03	0.032106	0.045647	0.944	1.13	0.481844	0.825321
ENT	Hearing difficulty problems	T	363510	94775	268869	1.01	0.013761	0.019625	0.976	1.05	0.483189	0.825321
Operations and Procedures	Other open operations on uterus	T	378957	285	378816	0.788	-0.2381	0.339141	0.405	1.53	0.482641	0.825321
Neurosciences	Neck or shoulder pain	T	378087	92289	285942	1.01	0.013574	0.019373	0.976	1.05	0.48351	0.825321
Neurosciences	Brain haemorrhage (HES and self-reported)	T	378957	1057	378044	0.89	-0.11632	0.16557	0.643	1.23	0.482327	0.825321
Biological assays	Neutrophill count	T	367067	NA	NA	NA	-0.0059	0.008443	-0.02245	0.010647	0.484565	0.826127
Medication	Corticosteroids combinations with antiseptics	T	378957	6117	372984	1.05	0.04513	0.064699	0.922	1.19	0.485464	0.826397
Neurosciences	Disorders of trigeminal nerve (HES)	T	378957	419	378682	0.827	-0.18947	0.272066	0.485	1.41	0.486174	0.826397
Eye	Other disorders of eyelid (HES)	T	378957	5168	373933	1.05	0.048777	0.070139	0.915	1.2	0.486782	0.826397
Gynaecology and Obstetrics	Hysteroscopy biopsy (self-reported)	T	378957	1325	377776	1.1	0.093728	0.134775	0.843	1.43	0.48678	0.826397
Operations and Procedures	Excision of lesion of tissue of brain	T	378957	434	378667	1.17	0.159478	0.229186	0.748	1.84	0.486525	0.826397
Gynaecology and Obstetrics	Pelvic inflammatory disease pid (HES and self-reported)	T	378957	4037	375064	1.06	0.055189	0.079161	0.905	1.23	0.485699	0.826397
Genitourinary	Other disorders of breast (HES)	T	378957	1110	377991	0.894	-0.11185	0.161484	0.652	1.23	0.488521	0.828147
Operations and Procedures	Oesophageal surgery (self-reported)	T	378957	447	378654	0.833	-0.18216	0.262897	0.498	1.4	0.488374	0.828147
Operations and Procedures	Transluminal operations on pulmonary artery	T	378957	411	378690	0.823	-0.1953	0.282177	0.473	1.43	0.488854	0.828147
Musculoskeletal	Fibromyalgia (HES and self-reported)	T	378957	1223	377878	1.1	0.096563	0.139622	0.838	1.45	0.489188	0.828147
Immuno-inflammation	Psoriasis (HES)	T	378957	2058	377043	1.08	0.075968	0.109932	0.87	1.34	0.489534	0.828151
Symptoms	Pain (HES)	T	378957	1292	377809	1.1	0.094147	0.136935	0.84	1.44	0.491751	0.831269
Medication	Corticosteroids and antiinfectives in combination 1	T	378957	5806	373295	1.05	0.045609	0.066386	0.919	1.19	0.492067	0.831269
Summary	Abn exm of fluids subst wo diag (HES)	T	378957	1857	377244	1.08	0.078708	0.11475	0.864	1.35	0.492771	0.831292
Neurosciences	Posttraumatic stress disorder (HES and self-reported)	T	378957	723	378378	0.869	-0.13988	0.203813	0.583	1.3	0.492522	0.831292
Operations and Procedures	Intubation of stomach	T	378957	329	378772	1.2	0.180853	0.264576	0.713	2.01	0.494255	0.833212
Summary	Glucose regulation and pancreatic internal secretion (HES)	T	378957	831	378270	0.879	-0.12921	0.189302	0.606	1.27	0.494898	0.833713
Operations and Procedures	Other operations on spermatic cord	T	378957	598	378503	1.14	0.134617	0.197493	0.777	1.68	0.495474	0.8341
Immuno-inflammation	Urticaria (HES and self-reported)	T	378957	530	378571	1.15	0.141841	0.209163	0.765	1.74	0.497684	0.837235
ENT	Adenoid surgery adenoidectomy (self-reported)	T	378957	2205	376896	1.07	0.070802	0.104761	0.874	1.32	0.499142	0.839102
Operations and Procedures	Extirpation of lesion of upper gastrointestinal tract	T	378957	1015	378086	1.11	0.104234	0.154509	0.82	1.5	0.499923	0.839829
Operations and Procedures	Therapeutic endoscopic operations on other joint structure	T	378957	4753	374348	1.05	0.049	0.07294	0.91	1.21	0.501718	0.842257
Musculoskeletal	Trunk BMD bone mineral density	T	4100	NA	NA	NA	-0.05782	0.086229	-0.22683	0.111192	0.502538	0.843046
Metabolic	Other disorders of pancreatic internal secretion (HES)	T	378957	829	378272	0.881	-0.12678	0.18931	0.608	1.28	0.503066	0.843344
Medication	Intestinal antiinfectives	T	378957	693	378408	0.87	-0.13916	0.208001	0.579	1.31	0.503466	0.843428
Eye	Retinal vascular occlusions (HES)	T	378957	466	378635	0.845	-0.16877	0.254716	0.513	1.39	0.507603	0.843952
Digestive system	Diseases of salivary glands (HES)	T	378957	909	378192	0.887	-0.11989	0.180231	0.623	1.26	0.505912	0.843952
Operations and Procedures	Hernia surgery (self-reported)	T	378957	2430	376671	1.07	0.066738	0.100815	0.877	1.3	0.507983	0.843952
Operations and Procedures	Other operations on ear	T	378957	414	378687	1.17	0.155914	0.235109	0.737	1.85	0.507229	0.843952
Operations and Procedures	Division of fascia	T	378957	394	378707	0.829	-0.18805	0.28233	0.476	1.44	0.505381	0.843952
Haematology	Pernicious anaemia (HES and self-reported)	T	378957	1499	377602	1.09	0.084974	0.128158	0.847	1.4	0.507304	0.843952
Digestive system	Ulcerative colitis (HES and self-reported)	T	378957	3555	375546	0.943	-0.05881	0.088811	0.792	1.12	0.507861	0.843952
Cardiovascular	Wolff parkinson white wpw syndrome (HES and self-reported)	T	378957	228	378873	1.23	0.205475	0.309292	0.67	2.25	0.506472	0.843952
Cardiovascular	Svt supraventricular tachycardia (HES and self-reported)	T	378957	2281	376820	0.929	-0.07356	0.110926	0.748	1.15	0.507226	0.843952
Musculoskeletal	Fracture wrist colles fracture (HES and self-reported)	T	378957	3198	375903	0.94	-0.06223	0.09385	0.782	1.13	0.507285	0.843952
Family history	High blood pressure (family history)	T	378957	NA	NA	NA	0.005533	0.008305	-0.01075	0.021811	0.505304	0.843952
Biological assays	Microalbumin in urine	T	114244	NA	NA	NA	0.009892	0.014859	-0.01923	0.039016	0.505607	0.843952
Summary	Mal neo of lip oral cavity and pharynx (cancer register)	T	378957	799	378302	0.881	-0.12642	0.192659	0.604	1.29	0.511697	0.849061
Operations and Procedures	Diagnostic lower respiratory tract	T	378957	4809	374292	0.951	-0.05012	0.076393	0.819	1.1	0.511763	0.849061
Operations and Procedures	Diagnostic imaging of musculoskeletal system	T	378957	820	378281	0.883	-0.1241	0.189319	0.609	1.28	0.51216	0.849135
Operations and Procedures	Other non operations	T	378957	2978	376123	0.936	-0.06639	0.10142	0.767	1.14	0.512737	0.849507
Medication	Cicatrizants	T	378957	2968	376133	0.938	-0.06406	0.098027	0.774	1.14	0.513441	0.849536
Genitourinary	Female infertility (HES)	T	378957	923	378178	1.11	0.105418	0.161745	0.809	1.53	0.514559	0.849536
Medication	Antiinflammatory agents and antiinfectives in combination	T	378957	17347	361754	0.974	-0.02627	0.040303	0.9	1.05	0.514527	0.849536
Family history	Lung cancer (family history - sibling)	T	301109	6394	294842	0.958	-0.04337	0.066525	0.841	1.09	0.514454	0.849536
Operations and Procedures	Other excision of bone	T	378957	3701	375400	0.945	-0.05641	0.08662	0.798	1.12	0.51487	0.849536
ENT	Sleep apnoea (HES and self-reported)	T	378957	4559	374542	0.95	-0.05105	0.078342	0.815	1.11	0.51467	0.849536
Summary	Chronic lower respiratory diseases (HES)	T	378957	30351	348750	1.02	0.01984	0.030514	0.961	1.08	0.515577	0.85012
Eye	Other disorders of lens (HES)	T	378957	203	378898	1.23	0.210632	0.324557	0.653	2.33	0.516349	0.850229
Operations and Procedures	Primary repair of umbilical hernia	T	378957	3099	376002	0.94	-0.06178	0.095174	0.78	1.13	0.51624	0.850229
Infectious disease	Dermatophytosis (HES)	T	378957	227	378874	1.22	0.200454	0.309394	0.666	2.24	0.517055	0.850229
Symptoms	General sensations and perceptions (HES)	T	378957	472	378629	0.848	-0.16503	0.254537	0.515	1.4	0.516754	0.850229
Immuno-inflammation	Decubitus ulcer (HES)	T	378957	840	378261	0.887	-0.12045	0.186262	0.615	1.28	0.517855	0.850362
Operations and Procedures	Other operations on lip	T	378957	464	378637	1.16	0.144246	0.22357	0.745	1.79	0.518802	0.850362
Operations and Procedures	Harvest of tendon	T	378957	532	378569	0.856	-0.15523	0.240012	0.535	1.37	0.517777	0.850362
Eye	Diabetes related eye disease	T	124323	2369	122007	0.932	-0.06989	0.108173	0.754	1.15	0.518241	0.850362
Cardiovascular	Pericardial effusion (HES and self-reported)	T	378957	600	378501	0.866	-0.14345	0.222394	0.56	1.34	0.518901	0.850362
Mental health	Phobic anxiety disorders (HES)	T	378957	527	378574	1.14	0.134661	0.209198	0.759	1.72	0.519767	0.850624
Operations and Procedures	Removal of rodent ulcer basal cell carcinoma bcc (self-reported)	T	378957	957	378144	1.11	0.10071	0.156404	0.814	1.5	0.519635	0.850624
Summary	Glomerular diseases (HES)	T	378957	1942	377159	1.07	0.07227	0.112618	0.862	1.34	0.521048	0.851564
Cancer	Mal neo testis (cancer register)	T	378957	451	378650	1.16	0.147163	0.229264	0.739	1.82	0.520944	0.851564
Summary	Other joint disorders (HES)	T	378957	35549	343552	0.982	-0.01823	0.028742	0.928	1.04	0.525907	0.852572
Respiratory	Other interstitial pulmonary diseases (HES)	T	378957	930	378171	1.11	0.102407	0.160138	0.809	1.52	0.522504	0.852572
Genitourinary	Unspecified renal failure (HES)	T	378957	1276	377825	1.09	0.087916	0.138274	0.833	1.43	0.5249	0.852572
Symptoms	Fever of unknown origin (HES)	T	378957	3729	375372	1.05	0.052462	0.082269	0.897	1.24	0.523679	0.852572
Medication	Muscle relaxants centrally acting agents	T	378957	555	378546	0.861	-0.14933	0.233659	0.545	1.36	0.522771	0.852572
Operations and Procedures	Excision of thyroid gland	T	378957	1898	377203	0.925	-0.07743	0.122202	0.728	1.18	0.526336	0.852572
Operations and Procedures	Endoscopic extirpation of lesion of bladder	T	378957	2389	376712	0.934	-0.06847	0.108103	0.756	1.15	0.526504	0.852572
Operations and Procedures	Operations on prepuce	T	378957	2323	376778	1.07	0.066529	0.105032	0.87	1.31	0.52646	0.852572
Operations and Procedures	Split autograft of skin	T	378957	1318	377783	1.09	0.086765	0.135772	0.836	1.42	0.522791	0.852572
Operations and Procedures	Suture of skin of other site	T	378957	2266	376835	1.07	0.066928	0.105229	0.87	1.31	0.524761	0.852572
Operations and Procedures	Total prosthetic replacement of other joint not using cement	T	378957	305	378796	0.816	-0.20374	0.321781	0.434	1.53	0.52662	0.852572
Operations and Procedures	High cost immunology drugs	T	378957	340	378761	1.18	0.163291	0.256372	0.712	1.95	0.524171	0.852572
Gynaecology and Obstetrics	Breast disease not cancer (HES and self-reported)	T	378957	3704	375397	0.946	-0.05501	0.086858	0.798	1.12	0.526508	0.852572
Musculoskeletal	Fracture lower leg ankle (HES and self-reported)	T	378957	6609	372492	1.04	0.039757	0.062411	0.921	1.18	0.524113	0.852572
Respiratory	Pulmonary fibrosis (HES and self-reported)	T	378957	789	378312	0.885	-0.12164	0.192801	0.607	1.29	0.528093	0.854383
Operations and Procedures	Neurophysiological operations	T	378957	2161	376940	0.93	-0.07238	0.115008	0.742	1.17	0.529112	0.854597
Operations and Procedures	Other operations on conjunctiva	T	378957	712	378389	1.12	0.112204	0.178667	0.788	1.59	0.529999	0.854597
Operations and Procedures	Other connection of stomach to jejunum	T	378957	256	378845	0.797	-0.22648	0.359406	0.394	1.61	0.528602	0.854597
Operations and Procedures	Combined operations on varicose vein of leg	T	378957	1623	377478	0.92	-0.08354	0.132933	0.709	1.19	0.529733	0.854597
Respiratory	Other respiratory problems (HES and self-reported)	T	378957	5684	373417	1.04	0.042315	0.067306	0.914	1.19	0.529544	0.854597
Eye	Retinal detachments and breaks (HES)	T	378957	2997	376104	1.06	0.05674	0.090867	0.886	1.26	0.532345	0.856088
Medication	Anesthetics general	T	378957	221	378880	0.787	-0.23998	0.384282	0.37	1.67	0.532307	0.856088
Gynaecology and Obstetrics	Breast biopsy (self-reported)	T	378957	375	378726	0.838	-0.1765	0.282626	0.482	1.46	0.532312	0.856088
Genitourinary	Haemorrhoids piles (HES and self-reported)	T	378957	1010	378091	0.9	-0.10498	0.167845	0.648	1.25	0.531685	0.856088
Haematology	Polycythaemia vera (HES and self-reported)	T	378957	393	378708	1.16	0.150559	0.241628	0.724	1.87	0.533216	0.856916
Operations and Procedures	Diagnostic spinal puncture	T	378957	4837	374264	0.954	-0.04714	0.075863	0.822	1.11	0.534339	0.857006
Operations and Procedures	Diagnostic endoscopic examination of pharynx	T	378957	3123	375978	1.06	0.05596	0.09004	0.886	1.26	0.534268	0.857006
Operations and Procedures	Injection of therapeutic substance	T	378957	1288	377813	1.09	0.08595	0.138209	0.831	1.43	0.534017	0.857006
Medication	Antifibrinolytics	T	378957	502	378599	1.14	0.133026	0.214347	0.75	1.74	0.534856	0.857264
Musculoskeletal	Other enthesopathies (HES)	T	378957	1540	377561	0.92	-0.0838	0.135294	0.705	1.2	0.535658	0.857842
Summary	Mal neo of urinary tract (cancer register)	T	378957	1919	377182	1.07	0.069834	0.112821	0.86	1.34	0.535929	0.857842
Operations and Procedures	Other operations on middle ear	T	378957	277	378824	1.19	0.175301	0.284333	0.683	2.08	0.537542	0.859319
Operations and Procedures	Diagnostic endoscopic examination of uterus	T	378957	20331	358770	1.02	0.023052	0.037392	0.951	1.1	0.537565	0.859319
Medication	Emollients and protectives	T	378957	1288	377813	0.913	-0.09128	0.148903	0.682	1.22	0.539877	0.860161
Summary	Cerebrovascular diseases (HES)	T	378957	7435	371666	0.963	-0.03755	0.061166	0.854	1.09	0.539323	0.860161
Operations and Procedures	Diagnostic electrocardiography	T	378957	998	378103	0.9	-0.10571	0.172416	0.642	1.26	0.539817	0.860161
Operations and Procedures	Division of bone of foot	T	378957	4767	374334	0.954	-0.04695	0.076373	0.821	1.11	0.538751	0.860161
Musculoskeletal	Pelvis BMD bone mineral density	T	4100	NA	NA	NA	-0.05287	0.086183	-0.22179	0.116047	0.539564	0.860161
Immuno-inflammation	Cutaneous abscess furuncle and carbuncle (HES)	T	378957	2145	376956	1.07	0.066059	0.108074	0.864	1.32	0.541039	0.860306
Metabolic	Pituitary adenomatumour (HES and self-reported)	T	378957	466	378635	0.856	-0.15578	0.254603	0.52	1.41	0.540626	0.860306
Infectious disease	Chickenpox (HES and self-reported)	T	378957	1557	377544	1.08	0.075505	0.123519	0.847	1.37	0.541011	0.860306
Biological assays	Haematocrit percentage	T	367722	NA	NA	NA	0.005149	0.008437	-0.01139	0.021687	0.541666	0.860734
Medication	Irrigating solutions	T	378957	1449	377652	1.08	0.079205	0.130229	0.839	1.4	0.543056	0.862336
Digestive system	Diseases of pulp and periapical tissues (HES)	T	378957	2104	376997	1.07	0.066409	0.10928	0.863	1.32	0.54339	0.862336

Neurosciences	Other mononeuropathies (HES)	T	378957	306	378795	0.823	-0.1951	0.321731	0.438	1.55	0.544248	0.863129
Operations and Procedures	Other operations on anus	T	378957	1643	377458	0.923	-0.07981	0.131829	0.713	1.2	0.544886	0.863573
Cardiovascular	Other cerebrovascular diseases (HES)	T	378957	2188	376913	0.934	-0.06815	0.113049	0.748	1.17	0.546599	0.865182
Genitourinary	Other noninflammatory disorders of cervix uteri (HES)	T	378957	1621	377480	1.08	0.073968	0.122791	0.846	1.37	0.546912	0.865182
Family history	Severe depression (family history)	T	378957	NA	NA	NA	0.004998	0.008298	-0.01127	0.021261	0.546979	0.865182
Operations and Procedures	Foot surgery (self-reported)	T	378957	7430	371671	1.04	0.035423	0.058938	0.923	1.16	0.547828	0.865956
Operations and Procedures	Bladder surgery (self-reported)	T	378957	2401	376700	1.06	0.060961	0.10164	0.871	1.3	0.548659	0.866432
Neurosciences	Diabetic neuropathyulcers (HES and self-reported)	T	378957	546	378555	0.869	-0.14016	0.233803	0.55	1.37	0.548848	0.866432
Operations and Procedures	Photocoagulation of retina for detachment	T	378957	972	378129	0.902	-0.10327	0.17247	0.643	1.26	0.549319	0.866607
Symptoms	Polyuria (HES)	T	378957	3507	375594	1.05	0.050829	0.085049	0.891	1.24	0.550074	0.86693
Symptoms	Dizziness and giddiness (HES)	T	378957	4147	374954	1.05	0.046967	0.078619	0.898	1.22	0.550243	0.86693
Operations and Procedures	Extirpation of lesion of other part of mouth	T	378957	688	378413	0.883	-0.124	0.208024	0.588	1.33	0.55111	0.867728
Musculoskeletal	Total BMD bone mineral density	T	4100	NA	NA	NA	-0.05126	0.086307	-0.22042	0.117905	0.552587	0.869485
Operations and Procedures	Operations on other articular cartilage	T	378957	1114	377987	1.09	0.087564	0.147856	0.817	1.46	0.553698	0.870111
Operations and Procedures	Connection of organ NOC	T	378957	354	378747	0.84	-0.17407	0.293928	0.472	1.49	0.553707	0.870111
Family history	Lung cancer (family history - mother)	T	354043	14282	339899	1.03	0.02569	0.043486	0.942	1.12	0.554682	0.871075
Metabolic	Thyroiditis (HES)	T	378957	275	378826	1.18	0.167257	0.284452	0.677	2.06	0.556533	0.872844
Symptoms	Unspecified jaundice (HES)	T	378957	839	378262	0.896	-0.10952	0.186212	0.622	1.29	0.55643	0.872844
Metabolic	Volume depletion (HES)	T	378957	2592	376509	1.06	0.057628	0.098346	0.874	1.28	0.557896	0.874412
Infectious disease	Other bacterial intestinal infections (HES)	T	378957	2315	376786	1.06	0.061103	0.104668	0.866	1.31	0.559372	0.875586
Operations and Procedures	Skin operation or plastic surgery (self-reported)	T	378957	3215	375886	1.05	0.051809	0.088675	0.885	1.25	0.559051	0.875586
Operations and Procedures	Other operations on joint	T	378957	520	378581	0.869	-0.14003	0.2401	0.543	1.39	0.559764	0.875631
Summary	Diseases of oral cavity salivary glands and jaws (HES)	T	378957	14224	364877	0.974	-0.02583	0.044374	0.893	1.06	0.560486	0.876191
Mental health	Unspecified dementia (HES)	T	378957	432	378669	1.14	0.133366	0.229427	0.729	1.79	0.561038	0.876485
Medication	Antiinflammatory and antirheumatic products non steroids	T	378957	86308	292793	0.988	-0.01158	0.019967	0.951	1.03	0.561957	0.877352
Mental health	Probable Recurrent major depression severe 1	T	91467	6715	84800	1.04	0.036918	0.063724	0.916	1.18	0.562359	0.877411
Operations and Procedures	Removal of benign skin lesion (self-reported)	T	378957	629	378472	1.12	0.111884	0.193592	0.765	1.63	0.563308	0.877755
Operations and Procedures	Ventilation support	T	378957	2964	376137	0.945	-0.05627	0.097282	0.781	1.14	0.562962	0.877755
Operations and Procedures	Other operations on skin	T	378957	1061	378040	0.909	-0.09556	0.165531	0.657	1.26	0.563748	0.877873
Operations and Procedures	Graft to organ NOC	T	378957	624	378477	0.882	-0.12522	0.21729	0.576	1.35	0.564424	0.878358
Medication	Anti dementia drugs	T	378957	2265	376836	0.938	-0.06369	0.11102	0.755	1.17	0.566184	0.87996
Cancer	Chronic lymphocytic (self-reported)	T	378957	244	378857	0.813	-0.20645	0.359746	0.402	1.65	0.566052	0.87996
Summary	Other viral diseases (HES)	T	378957	1781	377320	1.07	0.067407	0.118325	0.848	1.35	0.568898	0.880272
Summary	Diseases of external ear (HES)	T	378957	1324	377777	1.08	0.076112	0.13572	0.827	1.41	0.574933	0.880272
Summary	Lung diseases due to external agents (HES)	T	378957	898	378203	1.1	0.092687	0.164176	0.795	1.51	0.572373	0.880272
Summary	HESCH Diseases of the blood forming organ immune mech BIN (HES)	T	378957	22345	356756	0.98	-0.02005	0.035664	0.914	1.05	0.574039	0.880272
Eye	Disorders of vitreous body (HES)	T	378957	1246	377855	1.08	0.077935	0.138324	0.824	1.42	0.573147	0.880272
Digestive system	Dentofacial anomalies (HES)	T	378957	538	378563	1.13	0.118075	0.209111	0.747	1.7	0.572312	0.880272
Digestive system	Abscess of anal and rectal regions (HES)	T	378957	1208	377893	1.09	0.081783	0.143444	0.819	1.44	0.568583	0.880272
Genitourinary	Other noninflammatory disorders of uterus except cervix (HES)	T	378957	4717	374384	0.958	-0.04273	0.076311	0.825	1.11	0.57552	0.880272
Gynaecology and Obstetrics	Single delivery by caesarean section (HES)	T	378957	504	378597	0.871	-0.13824	0.241675	0.542	1.4	0.567314	0.880272
Symptoms	Faecal incontinence (HES)	T	378957	1634	377467	1.07	0.069634	0.12342	0.842	1.37	0.572615	0.880272
Symptoms	Abnormalities of gait and mobility (HES)	T	378957	1676	377425	0.93	-0.07274	0.129793	0.721	1.2	0.5752	0.880272
Symptoms	Other lack of coordination (HES)	T	378957	322	378779	1.16	0.150161	0.264679	0.692	1.95	0.570487	0.880272
Medication	Hypnotics and sedatives	T	378957	3007	376094	1.05	0.052088	0.091497	0.881	1.26	0.569159	0.880272
Operations and Procedures	Spine or back surgery (self-reported)	T	378957	4637	374464	1.04	0.041843	0.074042	0.902	1.21	0.571986	0.880272
Family history	Bowel cancer (family history - mother)	T	354043	17920	336261	0.978	-0.02226	0.039607	0.905	1.06	0.574182	0.880272
Gynaecology and Obstetrics	Ever had hysterectomy womb removed	T	180479	14618	165925	0.975	-0.02546	0.04535	0.892	1.07	0.574516	0.880272
Operations and Procedures	Other operations on maxillary antrum	T	378957	2872	376229	0.946	-0.05557	0.098927	0.779	1.15	0.574303	0.880272
Operations and Procedures	Removal of other substance from skin	T	378957	434	378667	0.862	-0.14802	0.262962	0.515	1.44	0.573504	0.880272
Operations and Procedures	Diagnostic blood tests	T	378957	378	378723	1.15	0.14205	0.248448	0.708	1.88	0.567491	0.880272
Operations and Procedures	Release of contracture of joint	T	378957	1613	377488	1.07	0.069683	0.123387	0.842	1.37	0.572242	0.880272
Operations and Procedures	Excision of lesion of organ NOC	T	378957	2108	376993	1.06	0.061904	0.109278	0.859	1.32	0.571065	0.880272
Cardiovascular	Hypertension (HES and self-reported)	T	378957	98025	281076	0.989	-0.01107	0.019599	0.952	1.03	0.572307	0.880272
Cardiovascular	Pulse wave Arterial Stiffness index	T	124901	NA	NA	NA	0.008098	0.014377	-0.02008	0.036277	0.573228	0.880272
Cardiovascular	LV end systolic volume	T	3764	NA	NA	NA	0.050852	0.089794	-0.12514	0.226847	0.571178	0.880272
Cardiovascular	LVEDVi	T	3760	NA	NA	NA	0.051063	0.089766	-0.12488	0.227005	0.569462	0.880272
Other	Chest pain or discomfort	T	375050	59237	315955	0.987	-0.0129	0.02311	0.943	1.03	0.576766	0.881575
Neurosciences	Other neurological problem (HES and self-reported)	T	378957	8378	370723	1.03	0.031123	0.055814	0.925	1.15	0.577104	0.881575
Cancer	Non hodgkins lymphoma (self-reported)	T	378957	719	378382	0.895	-0.1113	0.200023	0.605	1.32	0.577902	0.882149
Operations and Procedures	General anaesthetic	T	378957	13536	365565	0.975	-0.02522	0.045365	0.892	1.07	0.578212	0.882149
Summary	Viral infections of the central nervous system (HES)	T	378957	233	378868	1.19	0.17129	0.309131	0.648	2.18	0.579509	0.882237
Cancer	Large bowel cancer colorectal cancer (self-reported)	T	378957	526	378575	1.12	0.116102	0.209321	0.745	1.69	0.579129	0.882237
Cancer	Hodgkins lymphoma hodgkins disease (self-reported)	T	378957	352	378749	1.15	0.141839	0.256132	0.698	1.9	0.579735	0.882237
Operations and Procedures	Other manipulation of joint	T	378957	2050	377051	1.06	0.060977	0.109957	0.857	1.32	0.579199	0.882237
Operations and Procedures	Open approach to contents of cranium	T	378957	800	378301	0.901	-0.10452	0.189405	0.621	1.31	0.581048	0.883677
Symptoms	Symptoms the circulatory and respiratory systems (HES)	T	378957	1000	378101	1.09	0.085932	0.156181	0.802	1.48	0.582177	0.884662
Other	Chest pain or discomfort walking normally	T	58703	10166	48566	1.03	0.030971	0.056363	0.924	1.15	0.582664	0.884662
Operations and Procedures	Perineal operations for prolapse of rectum	T	378957	257	378844	1.18	0.162551	0.295921	0.659	2.1	0.582797	0.884662
Gynaecology and Obstetrics	Lumpectomy (self-reported)	T	378957	12746	366355	0.975	-0.02581	0.047117	0.889	1.07	0.583811	0.884778
Operations and Procedures	Dupuytren s contracture surgery (self-reported)	T	378957	409	378692	1.14	0.128882	0.235371	0.717	1.8	0.583987	0.884778
Operations and Procedures	Diagnostic endoscopic examination of fallopian tube	T	378957	293	378808	1.17	0.155814	0.284799	0.669	2.04	0.584309	0.884778
Operations and Procedures	Approach to organ through artery	T	378957	284	378817	0.838	-0.17637	0.322406	0.446	1.58	0.584343	0.884778
Immuno-inflammation	Other disorders involving the immune mechanism (HES)	T	378957	290	378811	0.84	-0.17424	0.322058	0.447	1.58	0.588491	0.886922
Gynaecology and Obstetrics	Maternal care for known or suspected abnormality of pelvic organs (HES)	T	378957	1384	377717	1.08	0.072463	0.134012	0.827	1.4	0.588699	0.886922
Cardiovascular	Aortic valve repair replacement (self-reported)	T	378957	561	378540	0.884	-0.1233	0.228031	0.565	1.38	0.588704	0.886922
Operations and Procedures	Secondary open reduction of fracture of bone	T	378957	1013	378088	0.913	-0.09105	0.167779	0.657	1.27	0.587364	0.886922
Metabolic	Adrenocortical insufficiencyaddisons disease (HES and self-reported)	T	378957	349	378752	0.853	-0.15936	0.293983	0.479	1.52	0.587759	0.886922
Musculoskeletal	Ankylosing spondylitis (HES and self-reported)	T	378957	1308	377793	0.923	-0.07983	0.147362	0.692	1.23	0.588014	0.886922
Digestive system	Appendicitis (HES and self-reported)	T	378957	6308	372793	1.04	0.034785	0.063924	0.913	1.17	0.586331	0.886922
Eye	Corneal hysteresis left	T	81021	NA	NA	NA	0.009649	0.01777	-0.02518	0.044478	0.587141	0.886922
Metabolic	Other disorders of adrenal gland (HES)	T	378957	431	378670	0.869	-0.14065	0.262991	0.519	1.45	0.592795	0.887535
Neurosciences	Mononeuropathies of lower limb (HES)	T	378957	1479	377622	1.07	0.069095	0.129166	0.832	1.38	0.592697	0.887535
Medication	Corticosteroids	T	378957	6270	372831	1.04	0.034541	0.064192	0.913	1.17	0.590519	0.887535
Cancer	Kidney renal cell cancer (self-reported)	T	378957	493	378608	1.12	0.117163	0.218426	0.733	1.73	0.591684	0.887535
Operations and Procedures	Lymph node surgery (self-reported)	T	378957	763	378338	1.1	0.094261	0.175792	0.779	1.55	0.591817	0.887535
Family history	High blood pressure (family history - mother)	T	357389	106967	250562	1.01	0.010064	0.018802	0.974	1.05	0.592476	0.887535
Operations and Procedures	Reconstruction of ossicular chain	T	378957	220	378881	0.813	-0.2068	0.384318	0.383	1.73	0.590503	0.887535
Operations and Procedures	Other bypass of femoral artery	T	378957	274	378827	0.834	-0.18153	0.339343	0.429	1.62	0.592691	0.887535
Operations and Procedures	Normal delivery	T	378957	6515	372586	1.04	0.036871	0.068524	0.907	1.19	0.590519	0.887535
Operations and Procedures	Diagnostic echocardiography	T	378957	8855	370246	0.971	-0.02984	0.055757	0.87	1.08	0.592549	0.887535
Eye	Paralytic strabismus (HES)	T	378957	258	378843	1.17	0.157825	0.295868	0.656	2.09	0.593735	0.887838
Medication	Adrenergics for systemic use	T	378957	22332	356769	1.02	0.018746	0.035122	0.951	1.09	0.593532	0.887838
Genitourinary	Ureteric obstructionhydronephrosis (HES and self-reported)	T	378957	2626	376475	0.947	-0.05481	0.102886	0.774	1.16	0.594245	0.88805
Eye	Dry eyes (HES and self-reported)	T	378957	847	378254	0.908	-0.09701	0.183356	0.634	1.3	0.596745	0.891233
Operations and Procedures	Diagnostic imaging of vascular system	T	378957	1073	378028	1.08	0.079736	0.15102	0.806	1.46	0.597513	0.891827
Digestive system	Dental caries (HES)	T	378957	2856	376245	0.95	-0.0518	0.098504	0.783	1.15	0.599008	0.892082
Medication	Antihistamines for systemic use	T	378957	13321	365780	0.976	-0.02395	0.045571	0.893	1.07	0.599165	0.892082
Operations and Procedures	Correction of ptosis of eyelid	T	378957	747	378354	1.1	0.093835	0.178396	0.774	1.56	0.598892	0.892082
Mental health	Stress (HES and self-reported)	T	378957	662	378439	1.11	0.100045	0.190173	0.761	1.6	0.598836	0.892082
Summary	Mal neo of respiratory and intrathoracic organs (cancer register)	T	378957	2026	377075	1.06	0.058476	0.111414	0.852	1.32	0.599686	0.892306
Operations and Procedures	Examination of upper gastrointestinal tract	T	378957	65264	313837	0.988	-0.01168	0.022327	0.946	1.03	0.600817	0.892886
Operations and Procedures	Extirpation of nail bed	T	378957	679	378422	1.1	0.099478	0.190066	0.761	1.6	0.600707	0.892886
Summary	Other diseases of upper respiratory tract (HES)	T	378957	13139	365962	1.02	0.023471	0.045321	0.937	1.12	0.604536	0.893029
Metabolic	Hyperparathyroidism of parathyroid gland (HES)	T	378957	752	378349	1.1	0.092464	0.178363	0.773	1.56	0.604179	0.893029
Symptoms	Other abnormal findings of blood chemistry (HES)	T	378957	5815	373286	1.04	0.034645	0.066718	0.908	1.18	0.603564	0.893029
Medication	Cough suppressants excl combinations with expectorants	T	378957	2402	376699	0.945	-0.05626	0.107838	0.765	1.17	0.601899	0.893029
ENT	Tonsillectomy tonsil surgery (self-reported)	T	378957	17457	361644	0.98	-0.02057	0.039592	0.906	1.06	0.603325	0.893029
Eye	Wears glasses or contact lenses	T	378384	337207	41321	0.985	-0.01506	0.029109	0.93	1.04	0.604991	0.893029
Operations and Procedures	Other operations on external ear	T	378957	435	378666	0.872	-0.13719	0.263008	0.521	1.46	0.601928	0.893029
Operations and Procedures	Other operations on tooth	T	378957	286	378815	1.16	0.147425	0.284106	0.664	2.02	0.603826	0.893029
Operations and Procedures	Ligation of varicose vein of leg	T	378957	5765	373336	0.965	-0.03604	0.069504	0.842	1.11	0.604038	0.893029
Operations and Procedures	Primary decompression operations on cervical spine	T	378957	557	378544	0.889	-0.11816	0.227963	0.568	1.39	0.60424	0.893029
Musculoskeletal	Fracture rib (HES and self-reported)	T	378957	2085	377016	0.942	-0.05992	0.115816	0.751	1.18	0.604909	0.893029
Neurosciences	Other demyelinating disease not multiple sclerosi (HES and self-reported)	T	378957	1324	377777	1.07	0.070387	0.136876	0.82	1.4	0.607082	0.895019
ENT	Earvestibular disorder (HES and self-reported)	T	378957	12078	367023	1.02	0.02414	0.046916	0.934	1.12	0.606879	0.895019
Medication	Adrenergics inhalants	T	378957	26289	352812	1.02	0.016715	0.032572	0.954	1.08	0.607829	0.895024

Cardiovascular	Rheumatic fever (HES and self-reported)	T	378957	3026	376075	1.05	0.046865	0.09125	0.876	1.25	0.607536	0.895024
Symptoms	Abnormal findings on diagnostic imaging of lung (HES)	T	378957	2263	376838	1.06	0.05397	0.105854	0.858	1.3	0.610155	0.897901
Summary	Number of operations self reported	T	378896	NA	NA	NA	-0.00423	0.008316	-0.02053	0.012067	0.610754	0.898233
Infectious disease	Viral warts (HES)	T	378957	643	378458	1.1	0.098314	0.193486	0.755	1.61	0.61137	0.898591
Eye	Other disorders of eye and adnexa (HES)	T	378957	430	378671	0.875	-0.13342	0.262998	0.523	1.47	0.611954	0.898901
Cardiovascular	Gestational hypertensionpreeclampsia (HES and self-reported)	T	378957	2534	376567	1.05	0.050561	0.099892	0.865	1.28	0.612747	0.899517
Metabolic	Thyroid problem not cancer (HES and self-reported)	T	378957	1330	377771	1.07	0.068487	0.13582	0.821	1.4	0.61409	0.900794
Cardiovascular	Ventricular rate	T	8940	NA	NA	NA	-0.02873	0.057027	-0.14051	0.08304	0.614365	0.900794
Musculoskeletal	Osteomyelitis (HES)	T	378957	486	378615	0.883	-0.12428	0.247138	0.544	1.43	0.615061	0.9009
Operations and Procedures	Other endoscopic removal of calculus from ureter	T	378957	242	378859	0.835	-0.18085	0.359763	0.412	1.69	0.615185	0.9009
Digestive system	Peritonitis (HES)	T	378957	722	378379	1.1	0.092248	0.18387	0.765	1.57	0.615875	0.901363
Respiratory	Bronchiectasis (HES)	T	378957	1708	377393	0.938	-0.06396	0.127862	0.73	1.21	0.616922	0.902342
Operations and Procedures	Operations on septum of nose	T	378957	4412	374689	0.961	-0.03992	0.079885	0.822	1.12	0.617293	0.902342
ENT	Acute nasopharyngitis (HES)	T	378957	356	378745	1.14	0.127071	0.256052	0.687	1.88	0.619703	0.902578
Cancer	Larynx throat cancer (self-reported)	T	378957	289	378812	1.15	0.141319	0.284183	0.66	2.01	0.618991	0.902578
Operations and Procedures	Other operations on varicose vein of leg	T	378957	6971	372130	1.03	0.030515	0.06148	0.914	1.16	0.61965	0.902578
Operations and Procedures	Subcutaneous injection	T	378957	2578	376523	1.05	0.049001	0.098724	0.865	1.27	0.619655	0.902578
Immuno-inflammation	Allergy or anaphylactic reaction to drug (HES and self-reported)	T	378957	4200	374901	1.04	0.038755	0.077942	0.892	1.21	0.619028	0.902578
Immuno-inflammation	Rosacea (HES and self-reported)	T	378957	850	378251	0.913	-0.09132	0.183223	0.637	1.31	0.618207	0.902578
Operations and Procedures	Other operations on amniotic cavity	T	378957	308	378793	0.858	-0.15262	0.308198	0.469	1.57	0.620462	0.903138
Operations and Procedures	Creation of artificial opening into ileum	T	378957	1023	378078	1.08	0.076315	0.154439	0.797	1.46	0.621205	0.903568
Operations and Procedures	Instrumental correction of deformity of spine	T	378957	342	378759	0.865	-0.14489	0.294098	0.486	1.54	0.622258	0.903568
Cardiovascular	Primary hypertension I270	T	378957	416	378685	0.878	-0.12985	0.263282	0.524	1.47	0.621884	0.903568
Anthropometry	VAT visceral adipose tissue mass	T	4051	NA	NA	NA	-0.04245	0.086141	-0.21129	0.126385	0.622147	0.903568
Symptoms	Elevated blood glucose level (HES)	T	378957	625	378476	0.899	-0.10592	0.217302	0.588	1.38	0.625959	0.904984
Cancer	Mal neo kidney (cancer register)	T	378957	918	378183	0.918	-0.08574	0.175108	0.651	1.29	0.624387	0.904984
Operations and Procedures	Operations on canthus	T	378957	1131	377970	1.07	0.072132	0.147842	0.804	1.44	0.625621	0.904984
Operations and Procedures	Excision of redundant skin of eyelid	T	378957	1118	377983	1.07	0.072127	0.147914	0.804	1.44	0.625813	0.904984
Operations and Procedures	Other large intestine	T	378957	3469	375632	0.958	-0.04339	0.088864	0.804	1.14	0.625379	0.904984
Operations and Procedures	Curettage of uterus	T	378957	6842	372259	0.969	-0.03153	0.064446	0.854	1.1	0.624656	0.904984
Neurosciences	Cranial nerve problempalsy (HES and self-reported)	T	378957	1916	377185	1.06	0.055741	0.113909	0.846	1.32	0.624592	0.904984
Cardiovascular	Mean carotid IMT intima medial thickness at 240 degrees	T	1843	NA	NA	NA	0.061208	0.125737	-0.18524	0.307653	0.626403	0.904984
Biological assays	Mean corpuscular haemoglobin	T	367719	NA	NA	NA	0.004094	0.008416	-0.0124	0.020589	0.626614	0.904984
Symptoms	Symptoms and signs involving emotional state (HES)	T	378957	584	378517	1.1	0.099247	0.20468	0.739	1.65	0.627756	0.905173
Operations and Procedures	Other periarticular division of bone	T	378957	263	378838	0.848	-0.16461	0.339392	0.436	1.65	0.627668	0.905173
Respiratory	FEV1 FVC ratio ever smoked only	T	175212	NA	NA	NA	-0.00594	0.01226	-0.02997	0.018086	0.627872	0.905173
Operations and Procedures	Other thoracic surgery (self-reported)	T	378957	1195	377906	0.928	-0.07437	0.15392	0.687	1.26	0.628974	0.906219
Operations and Procedures	Rectal or colon polypectomy (self-reported)	T	378957	1567	377534	1.06	0.060805	0.126173	0.83	1.36	0.629862	0.906414
Operations and Procedures	Extirpation of lesion of palate	T	378957	297	378804	0.856	-0.15524	0.321901	0.456	1.61	0.629631	0.906414
Respiratory	CopdMappingLoose primAndSec	T	244506	25872	218728	1.02	0.016771	0.034862	0.95	1.09	0.630458	0.90673
Symptoms	Speech disturbances (HES)	T	378957	1366	377735	0.934	-0.06856	0.143029	0.705	1.24	0.631676	0.90794
Genitourinary	Benign mammary dysplasia (HES)	T	378957	1624	377477	0.939	-0.06265	0.131044	0.727	1.21	0.632578	0.908179
Neurosciences	Mean time to correctly identify matches	T	376313	NA	NA	NA	0.003984	0.008333	-0.01235	0.020317	0.632596	0.908179
Medication	Other drugs for obstructive airway diseases inhalants	T	378957	29711	349390	0.985	-0.01483	0.03115	0.927	1.05	0.634119	0.909003
Operations and Procedures	Other autograft replacement of coronary artery	T	378957	496	378605	0.892	-0.11455	0.240587	0.556	1.43	0.633999	0.909003
Operations and Procedures	Preparation for external beam radiotherapy	T	378957	655	378446	1.09	0.090491	0.190234	0.754	1.59	0.634302	0.909003
Medication	Corticosteroids for systemic use plain	T	378957	6943	372158	1.03	0.029059	0.061235	0.913	1.16	0.635107	0.909615
Musculoskeletal	Acquired deformities of fingers and toes (HES)	T	378957	8762	370339	1.03	0.025882	0.054928	0.921	1.14	0.637501	0.910341
Summary	In situ neo (cancer register)	T	378957	8358	370743	0.973	-0.02707	0.057453	0.87	1.09	0.637503	0.910341
Operations and Procedures	Other operations on nose	T	378957	294	378807	0.859	-0.15212	0.321957	0.457	1.61	0.636571	0.910341
Operations and Procedures	Harvest of random pattern flap of skin from limb	T	378957	233	378868	0.844	-0.16964	0.360023	0.417	1.71	0.637497	0.910341
Medication	Vitamin B	T	376892	15815	361221	0.98	-0.01982	0.042051	0.903	1.06	0.637325	0.910341
Genitourinary	Glomerular disorders in diseases classified elsewhere (HES)	T	378957	293	378808	0.86	-0.15098	0.321989	0.457	1.62	0.639149	0.910532
Cancer	Carc in situ of skin (cancer register)	T	378957	1157	377944	1.07	0.068721	0.146456	0.804	1.43	0.63891	0.910532
Operations and Procedures	Drainage of subdural space	T	378957	236	378865	0.845	-0.16892	0.359986	0.417	1.71	0.638895	0.910532
Digestive system	Noninfective hepatitis (HES and self-reported)	T	378957	1195	377906	1.07	0.068021	0.144477	0.806	1.42	0.637775	0.910532
Cancer	Melanoma in situ (cancer register)	T	378957	1026	378075	1.07	0.072263	0.154409	0.794	1.45	0.639787	0.910589
Operations and Procedures	Other graft of bone	T	378957	696	378405	1.09	0.087432	0.186911	0.757	1.57	0.639945	0.910589
Summary	Other obstetric conditions not elsewhere classified (HES)	T	378957	1624	377477	0.94	-0.06148	0.131618	0.727	1.22	0.640434	0.910747
Summary	Other diseases of the digestive system (HES)	T	378957	11473	367628	0.977	-0.0228	0.049142	0.888	1.08	0.642657	0.912384
Cardiovascular	Cardiac arrest (HES)	T	378957	863	378238	1.08	0.077917	0.168445	0.777	1.5	0.643675	0.912384
Cardiovascular	Venous surgery procedures (self-reported)	T	378957	246	378855	1.15	0.142603	0.308729	0.63	2.11	0.64415	0.912384
Operations and Procedures	Ercp endoscopic retrograde cholangiopancreatogram (self-reported)	T	378957	341	378760	1.13	0.122058	0.264317	0.673	1.9	0.644236	0.912384
Gynaecology and Obstetrics	Ever had stillbirth spontaneous miscarriage or termination	T	201175	64150	137096	1.01	0.011322	0.024397	0.964	1.06	0.6426	0.912384
Operations and Procedures	Other operations on pilonidal sinus	T	378957	206	378895	1.17	0.158147	0.341226	0.6	2.29	0.64303	0.912384
Medication	Laxatives e g Dulcolax Senokot	T	374624	10818	363950	0.977	-0.02348	0.050729	0.884	1.08	0.643427	0.912384
Summary	Musculoskeletal system and connective tissue (HES)	T	378957	844	378257	1.08	0.077889	0.170605	0.774	1.51	0.647998	0.914
Musculoskeletal	Myositis (HES)	T	378957	207	378894	0.838	-0.17662	0.384714	0.394	1.78	0.646169	0.914
Genitourinary	Salpingitis and oophoritis (HES)	T	378957	472	378629	1.11	0.102117	0.223697	0.714	1.72	0.648033	0.914
Operations and Procedures	Destruction of lesion of retina	T	378957	2270	376831	0.951	-0.05028	0.109778	0.767	1.18	0.646923	0.914
Operations and Procedures	Primary repair of femoral hernia	T	378957	527	378574	0.898	-0.10707	0.233935	0.568	1.42	0.647161	0.914
Operations and Procedures	Adjustment to length of tendon	T	378957	2142	376959	0.95	-0.05166	0.11304	0.761	1.19	0.64765	0.914
Metabolic	Graves disease (HES and self-reported)	T	378957	476	378625	0.893	-0.11304	0.247257	0.55	1.45	0.647536	0.914
Family history	Bowel cancer (family history - father)	T	338597	19587	319142	1.02	0.017031	0.03741	0.945	1.09	0.648918	0.914177
Neurosciences	Infection of nervous system (HES and self-reported)	T	378957	529	378572	0.899	-0.10653	0.233865	0.568	1.42	0.64873	0.914177
Medication	Other dermatological preparations	T	378957	20932	358169	1.02	0.016466	0.036339	0.947	1.09	0.650457	0.915423
Digestive system	Acute appendicitis (HES)	T	378957	2629	376472	1.05	0.044499	0.098237	0.862	1.27	0.650562	0.915423
Operations and Procedures	Excision of dental lesion of jaw	T	378957	624	378477	1.09	0.089062	0.197016	0.743	1.61	0.651229	0.915826
Eye	Disorders of lacrimal system (HES)	T	378957	1950	377151	0.948	-0.05328	0.118955	0.751	1.2	0.654242	0.917681
Genitourinary	Chronic renal failure (HES)	T	378957	4070	375031	0.964	-0.03647	0.081608	0.822	1.13	0.654934	0.917681
Medication	Other antineoplastic agents	T	378957	1117	377984	1.07	0.066689	0.149376	0.798	1.43	0.655275	0.917681
Operations and Procedures	Other operations on spinal cord	T	378957	218	378883	1.16	0.144873	0.323972	0.613	2.18	0.654748	0.917681
Operations and Procedures	Reconstruction of carotid artery	T	378957	506	378595	1.1	0.09742	0.218428	0.718	1.69	0.655595	0.917681
Operations and Procedures	Flap operations to relax contracture of skin	T	378957	656	378445	0.911	-0.09362	0.208245	0.605	1.37	0.653039	0.917681
Operations and Procedures	Re exploration of organ NOC	T	378957	217	378884	1.16	0.145198	0.324001	0.613	2.18	0.654052	0.917681
Digestive system	Incisional hernia (HES and self-reported)	T	378957	307	378794	1.13	0.122345	0.273849	0.661	1.93	0.655047	0.917681
Medication	Propulsives	T	378957	1384	377717	1.06	0.05943	0.134485	0.815	1.38	0.658554	0.918547
Cancer	Thyroid cancer (self-reported)	T	378957	362	378739	1.12	0.11292	0.255993	0.678	1.85	0.659137	0.918547
ENT	Maxillo facial surgery (self-reported)	T	378957	2821	376280	0.957	-0.04364	0.098978	0.788	1.16	0.659264	0.918547
Neurosciences	Motor neurone disease (HES and self-reported)	T	378957	231	378870	0.853	-0.1594	0.36005	0.421	1.73	0.657968	0.918547
Digestive system	Primary biliary cirrhosis (HES and self-reported)	T	378957	237	378864	0.853	-0.15949	0.359924	0.421	1.73	0.657671	0.918547
Infectious disease	Hepatitis c (HES and self-reported)	T	378957	331	378770	0.872	-0.1364	0.306993	0.478	1.59	0.656815	0.918547
Musculoskeletal	Epicondylitis (HES and self-reported)	T	378957	745	378356	1.08	0.080035	0.180986	0.76	1.54	0.658334	0.918547
Biological assays	Mean corpuscular haemoglobin concentration	T	367715	NA	NA	NA	0.003745	0.008434	-0.01279	0.020276	0.657036	0.918547
Medication	Anxiolytics	T	378957	1664	377437	1.06	0.054219	0.1233	0.829	1.34	0.66013	0.91904
Operations and Procedures	Minimal access to abdominal cavity	T	378957	14863	364238	1.02	0.018646	0.042436	0.938	1.11	0.660381	0.91904
Musculoskeletal	Neck probleminjury (HES and self-reported)	T	378957	520	378581	0.903	-0.10247	0.233951	0.571	1.43	0.661375	0.919892
Digestive system	Stomatitis and related lesions (HES)	T	378957	744	378357	0.918	-0.08573	0.196281	0.625	1.35	0.66227	0.920605
Cardiovascular	Aortic aneurysm and dissection (HES)	T	378957	1199	377902	1.06	0.062145	0.142557	0.805	1.41	0.662889	0.920934
Cardiovascular	Irregular heart beat (HES and self-reported)	T	378957	1395	377706	0.941	-0.06094	0.140416	0.715	1.24	0.664296	0.922357
Operations and Procedures	Other reconstruction of ligament	T	378957	749	378352	0.918	-0.08511	0.196359	0.625	1.35	0.664681	0.92236
Medication	Antimalarials	T	378957	3446	375655	1.04	0.037283	0.086294	0.876	1.23	0.665705	0.923249
Operations and Procedures	Excision reconstruction of joint	T	378957	1872	377229	1.05	0.049726	0.11542	0.838	1.32	0.666596	0.923953
Cardiovascular	Other nontraumatic intracranial haemorrhage (HES)	T	378957	336	378765	1.12	0.111911	0.264483	0.666	1.88	0.672199	0.926568
Symptoms	Unknown and unspecified causes of morbidity (HES)	T	378957	14557	364544	0.982	-0.01843	0.043569	0.901	1.07	0.672245	0.926568
Operations and Procedures	Anterior posterior repair bladder uterus (self-reported)	T	378957	3518	375583	1.04	0.03621	0.085494	0.877	1.23	0.671904	0.926568
Operations and Procedures	Lower limb surgery (self-reported)	T	378957	1770	377331	0.948	-0.05354	0.125805	0.741	1.21	0.670409	0.926568
Operations and Procedures	Filtering operations on iris	T	378957	833	378268	1.08	0.072883	0.170756	0.77	1.5	0.669509	0.926568
Operations and Procedures	Introduction of therapeutic substance into subcutaneous tissue	T	378957	610	378491	0.912	-0.09227	0.217447	0.595	1.4	0.67132	0.926568
Operations and Procedures	Diagnostic imaging of central nervous system	T	378957	13682	365419	1.02	0.018873	0.044225	0.934	1.11	0.669555	0.926568
Gynaecology and Obstetrics	Gynaecological disorder not c (HES and self-reported)	T	378957	16832	362269	1.02	0.017311	0.040761	0.939	1.1	0.671071	0.926568
Gynaecology and Obstetrics	Uterine fibroids (HES and self-reported)	T	378957	13674	365427	1.02	0.01892	0.044733	0.934	1.11	0.672329	0.926568
Gynaecology and Obstetrics	Menopausal symptoms menopause (HES and self-reported)	T	378957	9705	369396	1.02	0.022366	0.052716	0.922	1.13	0.671373	0.926568
Operations and Procedures	Other induction of labour	T	378957	2732	376369	0.958	-0.04332	0.102684	0.783	1.17	0.673125	0.926605
Biological assays	Red blood cell erythrocyte count	T	367722	NA	NA	NA	0.003557	0.008424	-0.01295	0.020067	0.672881	0.926605
Other	Pattern 3 f	T	173207	46281	126998	1.01	0.01182	0.028059	0.958	1.07	0.673578	0.926699
Operations and Procedures	Vacuum delivery	T	378957	1205	377896	0.938	-0.06379	0.15182	0.697	1.26	0.674353	0.926706

Operations and Procedures	Burrhole approach to contents of cranium	T	378957	411	378690	1.11	0.101489	0.241304	0.69	1.78	0.674056	0.926706
Cancer	Lung cancer (self-reported)	T	378957	205	378896	0.851	-0.16148	0.384862	0.4	1.81	0.674785	0.926771
Metabolic	Hyperparathyroidism (HES and self-reported)	T	378957	788	378313	1.08	0.073536	0.175658	0.763	1.52	0.675486	0.927206
Cancer	Skin cancer (self-reported)	T	378957	1189	377912	1.06	0.059948	0.143531	0.801	1.41	0.676193	0.927647
Metabolic	Thyrotoxicosis (HES)	T	378957	1663	377438	0.948	-0.05336	0.128837	0.736	1.22	0.678732	0.92822
Mental health	Sexual dysfunction not caused disorder or disease (HES)	T	378957	264	378837	0.871	-0.13797	0.339516	0.448	1.69	0.684471	0.92822
Neurosciences	Nerve root and plexus disorders (HES)	T	378957	316	378785	1.12	0.112181	0.273616	0.654	1.91	0.681809	0.92822
ENT	Other hearing loss (HES)	T	378957	2563	376538	0.959	-0.04207	0.102976	0.784	1.17	0.682899	0.92822
Cardiovascular	Sequelae of cerebrovascular disease (HES)	T	378957	1161	377940	1.06	0.060171	0.146349	0.797	1.41	0.680963	0.92822
Cardiovascular	Varicose veins of other sites (HES)	T	378957	460	378641	0.902	-0.10358	0.254752	0.547	1.49	0.684305	0.92822
Genitourinary	Inflammatory diseases of prostate (HES)	T	378957	1436	377665	0.944	-0.05764	0.13949	0.718	1.24	0.67943	0.92822
Medication	Agents against amoebiasis and other protozoal diseases	T	378957	660	378441	0.918	-0.08512	0.20818	0.611	1.38	0.682618	0.92822
Medication	Corticosteroids and antiinfectives in combination 2	T	378957	6221	372880	1.03	0.02647	0.064676	0.905	1.17	0.682342	0.92822
Cardiovascular	Pacemaker defibrillator insertion (self-reported)	T	378957	680	378421	0.92	-0.0829	0.204152	0.617	1.37	0.684702	0.92822
Cancer	Hodgkin s disease (cancer register)	T	378957	245	378856	1.14	0.128321	0.308754	0.621	2.08	0.677697	0.92822
Operations and Procedures	Microsurgical repair of peripheral nerve	T	378957	222	378879	1.14	0.132145	0.323858	0.605	2.15	0.68325	0.92822
Operations and Procedures	Other therapeutic endoscopic operations on larynx	T	378957	206	378895	0.855	-0.15686	0.384739	0.402	1.82	0.683499	0.92822
Operations and Procedures	Examination of sigmoid colon	T	378957	2690	376411	1.04	0.040163	0.09778	0.859	1.26	0.681256	0.92822
Operations and Procedures	Forceps cephalic delivery	T	378957	705	378396	1.08	0.076941	0.186088	0.75	1.56	0.679266	0.92822
Operations and Procedures	Exploration of other skin of head or neck	T	378957	445	378656	1.1	0.097463	0.234731	0.696	1.75	0.677987	0.92822
Operations and Procedures	Release of fascia	T	378957	204	378897	0.854	-0.15747	0.384847	0.402	1.82	0.682413	0.92822
Medication	Glucosamine	T	377706	73561	304289	0.991	-0.00876	0.021318	0.951	1.03	0.680989	0.92822
Metabolic	Thyroid radioablation therapy (HES and self-reported)	T	378957	2506	376595	0.959	-0.04233	0.104055	0.782	1.18	0.684155	0.92822
Metabolic	Disorder of adrenal gland (HES and self-reported)	T	378957	470	378631	0.903	-0.10239	0.247253	0.556	1.47	0.67878	0.92822
Gynaecology and Obstetrics	Ovarian cyst or cysts (HES and self-reported)	T	378957	6134	372967	0.973	-0.02763	0.066755	0.853	1.11	0.678922	0.92822
Medication	Antiadrenergic agents peripherally acting	T	378957	4727	374374	0.97	-0.03075	0.07585	0.836	1.13	0.685179	0.928344
Cardiovascular	Carotid artery angioplasty stent (self-reported)	T	378957	333	378768	0.888	-0.11867	0.294382	0.499	1.58	0.686876	0.929076
Gynaecology and Obstetrics	Ovarian tubal surgery (self-reported)	T	378957	1138	377963	0.939	-0.06288	0.155938	0.692	1.27	0.686786	0.929076
Summary	Neo of uncertain or unknown behaviour (cancer register)	T	378957	2117	376984	1.04	0.0439	0.108748	0.844	1.29	0.686445	0.929076
Cancer	Carc in situ of other digestive organs (cancer register)	T	378957	262	378839	1.13	0.118501	0.295806	0.63	2.01	0.688711	0.931035
Summary	Congenital malformations of the urinary system (HES)	T	378957	821	378280	1.07	0.068802	0.173032	0.763	1.5	0.690906	0.932955
Metabolic	Other hypothyroidism (HES)	T	378957	12869	366232	1.02	0.018198	0.045724	0.931	1.11	0.690639	0.932955
Gynaecology and Obstetrics	Gestational hypertension without significant proteinuria (HES)	T	378957	427	378674	0.903	-0.10177	0.256514	0.546	1.49	0.691562	0.933318
Digestive system	Irritable bowel syndrome (HES)	T	378957	5022	374079	0.971	-0.02922	0.073818	0.84	1.12	0.69218	0.933629
Musculoskeletal	Other disorders of bone density and structure (HES)	T	378957	998	378103	0.936	-0.06602	0.167887	0.674	1.3	0.694147	0.933668
Operations and Procedures	Neurostimulation of peripheral nerve	T	378957	545	378556	0.914	-0.08983	0.228082	0.585	1.43	0.6937	0.933668
NA	Ischaemic stroke	T	378957	3655	375446	0.966	-0.03408	0.086557	0.816	1.15	0.69378	0.933668
Genitourinary	Erectile dysfunction impotence (HES and self-reported)	T	378957	497	378604	0.909	-0.09487	0.240583	0.568	1.46	0.693323	0.933668
Musculoskeletal	Fracture metatarsal (HES and self-reported)	T	378957	436	378665	1.1	0.09245	0.23481	0.692	1.74	0.693787	0.933668
Immuno-inflammation	Other follicular disorders (HES)	T	378957	373	378728	1.11	0.100268	0.255788	0.67	1.83	0.695059	0.933852
Medication	Decongestants and other nasal preparations for topical use	T	378957	33775	345326	0.989	-0.01151	0.029345	0.933	1.05	0.694993	0.933852
Medication	Hemorrhoids and anal fissures for topical use	T	378957	11708	367393	1.02	0.018699	0.047852	0.928	1.12	0.695961	0.934543
Anthropometry	Visceral adipose tissue volume VAT	T	801	NA	NA	NA	-0.07798	0.199962	-0.46991	0.313943	0.696545	0.934806
Medication	Beta lactam antibacterials penicillins	T	378957	1102	377999	1.06	0.058641	0.150896	0.789	1.43	0.697556	0.935121
Operations and Procedures	Discectomy (self-reported)	T	378957	1381	377720	1.05	0.052281	0.134474	0.81	1.37	0.697438	0.935121
Operations and Procedures	Compensation for renal failure	T	378957	703	378398	0.925	-0.07767	0.200136	0.625	1.37	0.697952	0.935131
Medication	Angiotensin ii antagonists combinations	T	378957	30598	348503	0.988	-0.01207	0.031154	0.929	1.05	0.698554	0.935415
Medication	Other gynecologicals	T	378957	52465	326636	1.01	0.009265	0.02419	0.963	1.06	0.701697	0.935415
Summary	Viral infections skin and mucous memb (HES)	T	378957	1422	377679	1.05	0.050935	0.133293	0.81	1.37	0.702367	0.935415
Summary	Bacterial viral and other infectious agents (HES)	T	378957	11829	367272	1.02	0.018151	0.047689	0.927	1.12	0.703491	0.935415
Metabolic	Deficiency of other B group vitamins (HES)	T	378957	664	378437	1.08	0.073999	0.193361	0.737	1.57	0.701942	0.935415
Gynaecology and Obstetrics	Other maternal diseases complicating pregnancy (HES)	T	378957	1582	377519	0.951	-0.05076	0.132682	0.733	1.23	0.702034	0.935415
Symptoms	Localized swelling mass and lump of skin and subcutaneous tissue (HES)	T	378957	1921	377180	0.955	-0.04559	0.11967	0.756	1.21	0.703256	0.935415
Symptoms	Symptoms cognitive functions and awareness (HES)	T	378957	2889	376212	1.04	0.035812	0.094155	0.862	1.25	0.703681	0.935415
Operations and Procedures	Plastic repair of mitral valve	T	378957	528	378573	1.08	0.081239	0.213561	0.714	1.65	0.703648	0.935415
Operations and Procedures	Neuropsychology tests	T	378957	256	378845	0.879	-0.12901	0.339556	0.452	1.71	0.703988	0.935415
Eye	Eye trauma (HES and self-reported)	T	378957	816	378285	1.07	0.066781	0.175496	0.758	1.51	0.703552	0.935415
Neurosciences	Trigemminal neuralgia (HES and self-reported)	T	378957	464	378637	0.909	-0.09553	0.247351	0.56	1.48	0.699331	0.935415
Eye	Macular degeneration (HES and self-reported)	T	378957	2130	376971	0.958	-0.04304	0.112573	0.768	1.19	0.702226	0.935415
Other	Morning evening person chronotype	T	338480	NA	NA	NA	-0.00336	0.008821	-0.02065	0.013929	0.703201	0.935415
Cardiovascular	Mean carotid IMT intima medial thickness at 210 degrees	T	1850	NA	NA	NA	-0.04795	0.123983	-0.29095	0.195061	0.698971	0.935415
Cardiovascular	Other lymphatic vessels and lymph nodes (HES)	T	378957	601	378500	1.08	0.075959	0.200755	0.728	1.6	0.705159	0.935711
Digestive system	Other diseases of lip and oral mucosa (HES)	T	378957	2606	376495	0.962	-0.03891	0.102871	0.786	1.18	0.705275	0.935711
Operations and Procedures	Therapeutic endoscopic operations on uterus	T	378957	6827	372274	0.976	-0.02402	0.063521	0.862	1.11	0.705376	0.935711
Genitourinary	Cystic kidney disease (HES)	T	378957	414	378687	1.1	0.090913	0.241262	0.683	1.76	0.706305	0.936427
Biological assays	Nucleated red blood cell count	T	367058	NA	NA	NA	-0.00316	0.008418	-0.01966	0.01334	0.707388	0.937347
Musculoskeletal	Fracture upper arm humerus elbow (HES and self-reported)	T	378957	3361	375740	0.967	-0.03347	0.090218	0.81	1.15	0.71067	0.941179
Cardiovascular	Subarachnoid haemorrhage (HES)	T	378957	743	378358	1.07	0.067992	0.183752	0.747	1.53	0.711367	0.941584
Neurosciences	Multiple sclerosis (HES)	T	378957	1215	377886	1.05	0.052962	0.143401	0.796	1.4	0.711882	0.941653
Respiratory	Postprocedural respiratory disorders (HES)	T	378957	334	378767	0.898	-0.10778	0.294254	0.504	1.6	0.714155	0.941653
Gynaecology and Obstetrics	Postpartum haemorrhage (HES)	T	378957	1204	377897	1.05	0.053222	0.144808	0.794	1.4	0.713218	0.941653
Cancer	Cervical cancer (self-reported)	T	378957	1533	377568	1.05	0.0473	0.128253	0.815	1.35	0.712275	0.941653
Operations and Procedures	Colonoscopy sigmoidoscopy (self-reported)	T	378957	7510	371591	0.978	-0.02222	0.060548	0.869	1.1	0.713644	0.941653
Summary	Had major operations	T	173294	111364	62003	1.01	0.009582	0.026148	0.959	1.06	0.714041	0.941653
Operations and Procedures	Other examination of female genital tract	T	378957	3998	375103	0.97	-0.03043	0.083034	0.824	1.14	0.713988	0.941653
Digestive system	Unspecified appendicitis (HES)	T	378957	412	378689	0.908	-0.09615	0.263275	0.542	1.52	0.714958	0.942196
Medication	Calcium channel blockers and diuretics	T	378957	18780	360321	0.986	-0.01411	0.038961	0.913	1.06	0.71719	0.942262
Summary	HESCH Pregnancy childbirth and the puerperium (HES)	T	378957	12811	366290	0.98	-0.01974	0.054225	0.882	1.09	0.715883	0.942262
Operations and Procedures	Closure of cornea	T	378957	204	378897	0.87	-0.1397	0.384808	0.409	1.85	0.716571	0.942262
Operations and Procedures	Endoscopic retrograde placement of prosthesis in bile duct	T	378957	560	378541	1.08	0.075636	0.208942	0.716	1.62	0.717355	0.942262
Operations and Procedures	Other operations to facilitate delivery	T	378957	2119	376982	0.959	-0.04175	0.115202	0.765	1.2	0.717052	0.942262
Cardiovascular	Cardiac output	T	3764	NA	NA	NA	0.032491	0.089721	-0.14336	0.208344	0.717254	0.942262
Operations and Procedures	Other operations on peripheral nerve	T	378957	2062	377039	0.959	-0.04135	0.115085	0.766	1.2	0.719338	0.942938
Operations and Procedures	Other vaginal operations on uterus	T	378957	1552	377549	1.05	0.045384	0.126712	0.816	1.34	0.720218	0.942938
Operations and Procedures	Other route of administration of therapeutic substance	T	378957	241	378860	0.879	-0.12946	0.359784	0.434	1.78	0.718968	0.942938
Operations and Procedures	Immobilisation using plaster cast	T	378957	694	378407	1.07	0.068205	0.189962	0.738	1.55	0.71956	0.942938
Operations and Procedures	Biopsy of organ NOC	T	378957	7160	371941	1.02	0.021675	0.060466	0.908	1.15	0.71999	0.942938
Digestive system	Colitisnot crohns or ulcerative colitis (HES and self-reported)	T	378957	15433	363668	1.02	0.015101	0.041893	0.935	1.1	0.718496	0.942938
Summary	Complications of labour and delivery (HES)	T	378957	8512	370589	1.02	0.022282	0.062411	0.905	1.16	0.721076	0.943548
Symptoms	Isolated proteinuria (HES)	T	378957	277	378824	1.11	0.105238	0.295388	0.623	1.98	0.721639	0.943772
ENT	Acute pharyngitis (HES)	T	378957	757	378344	0.934	-0.06833	0.192818	0.64	1.36	0.723073	0.944375
Symptoms	Heartburn (HES)	T	378957	1828	377273	0.957	-0.04359	0.123087	0.752	1.22	0.723208	0.944375
Biological assays	Nucleated red blood cell percentage	T	367055	NA	NA	NA	-0.00298	0.008418	-0.01948	0.013519	0.723276	0.944375
Operations and Procedures	Other operations on haemorrhoid	T	378957	276	378825	1.11	0.104383	0.29541	0.622	1.98	0.723826	0.944581
Cardiovascular	Nonrheumatic mitral valve disorders (HES)	T	378957	2168	376933	1.04	0.038155	0.10817	0.84	1.28	0.724286	0.94467
Musculoskeletal	Cervical disc disorders (HES)	T	378957	1357	377744	0.951	-0.05022	0.143024	0.719	1.26	0.725497	0.945226
Operations and Procedures	Incision of iris	T	378957	599	378502	1.07	0.070559	0.200831	0.724	1.59	0.725338	0.945226
Neurosciences	Other polyneuropathies (HES)	T	378957	1097	378004	1.05	0.05249	0.150975	0.784	1.42	0.728083	0.948082
Cardiovascular	Other venouslymphatic disease (HES and self-reported)	T	378957	1107	377994	0.947	-0.05467	0.15761	0.695	1.29	0.728703	0.948377
Operations and Procedures	Other autograft of skin	T	378957	1822	377279	1.04	0.040661	0.117643	0.827	1.31	0.729622	0.948933
Operations and Procedures	Complex reconstruction of hindfoot	T	378957	342	378759	1.1	0.091249	0.264309	0.653	1.84	0.729918	0.948933
Neurosciences	Other demyelinating diseases of CNS (HES)	T	378957	283	378818	0.897	-0.10828	0.322199	0.477	1.69	0.736813	0.949189
Respiratory	Acute upper respiratory infections (HES)	T	378957	729	378372	0.936	-0.06601	0.196358	0.637	1.38	0.736751	0.949189
Digestive system	Other diseases of oesophagus	T	378957	8938	370163	1.02	0.018504	0.054639	0.915	1.13	0.734868	0.949189
Digestive system	Paralytic ileus and intestinal obstruction without hernia (HES)	T	378957	3590	375511	0.971	-0.02974	0.087004	0.819	1.15	0.732494	0.949189
Immuno-inflammation	Skin changes due to chronic exposure to nonionizing radiation (HES)	T	378957	2179	376922	1.04	0.036742	0.107603	0.84	1.28	0.732757	0.949189
Musculoskeletal	Kyphosis and lordosis (HES)	T	378957	201	378900	1.12	0.114875	0.341323	0.575	2.19	0.736449	0.949189
Musculoskeletal	Other bursopathies (HES)	T	378957	729	378372	1.06	0.062367	0.18383	0.742	1.53	0.734408	0.949189
Genitourinary	Hypertrophy of breast (HES)	T	378957	719	378382	1.07	0.063987	0.186804	0.739	1.54	0.731947	0.949189
Genitourinary	Dysplasia of cervix uteri (HES)	T	378957	1404	377697	1.05	0.045082	0.133676	0.805	1.36	0.73593	0.949189
Cancer	Uterine endometrial cancer (self-reported)	T	378957	936	378165	1.06	0.055467	0.162223	0.769	1.45	0.732414	0.949189
Metabolic	Thyroidectomy partial thyroidectomy (self-reported)	T	378957	3577	375524	0.971	-0.02985	0.086878	0.819	1.15	0.731126	0.949189
Operations and Procedures	Operations on eyebrow	T	378957	613	378488	1.07	0.067739	0.200674	0.722	1.59	0.735696	0.949189
Operations and Procedures	Therapeutic ureteroscopic operations on ureter	T	378957	1338	377763	1.05	0.047004	0.138068	0.8	1.37	0.733522	0.949189
Operations and Procedures	Denervation of spinal facet joint of vertebra	T	378957	675	378426	1.07	0.064174	0.190084	0.735	1.55	0.735659	0.949189
Eye	Eyeeyelid problem (HES and self-reported)	T	378957	17843	361258	1.01	0.013137	0.039073	0.939	1.09	0.736715	0.949189
Musculoskeletal	Fracture finger (HES and self-reported)	T	378957	1578	377523	1.04	0.043068	0.127045	0.814	1.34	0.73461	0.949189

ENT	Hear loss B	T	120977	NA	NA	NA	0.004957	0.014545	-0.02355	0.033466	0.733226	0.949189
Operations and Procedures	Repair of diaphragmatic hernia	T	378957	276	378825	0.898	-0.10779	0.322328	0.477	1.69	0.738063	0.950291
Metabolic	Thyroid radioablation therapy (self-reported)	T	378957	360	378741	0.91	-0.0941	0.282759	0.523	1.58	0.739287	0.95085
ENT	Chronic sinusitis (HES and self-reported)	T	378957	4361	374740	0.974	-0.02628	0.078934	0.834	1.14	0.739178	0.95085
Summary	Oed prot and hyprtn dis in preg chldbrth (HES)	T	378957	1199	377902	0.952	-0.04965	0.151531	0.707	1.28	0.743163	0.952697
Neurosciences	Other paralytic syndromes (HES)	T	378957	334	378767	0.908	-0.09678	0.29422	0.51	1.62	0.742207	0.952697
Symptoms	Oedema (HES)	T	378957	1490	377611	0.956	-0.04449	0.135432	0.733	1.25	0.742518	0.952697
Summary	Mal neo of mesothelial and soft tissue (cancer register)	T	378957	538	378563	0.928	-0.075	0.228155	0.593	1.45	0.74237	0.952697
Operations and Procedures	Endoscopic operations to increase capacity of bladder	T	378957	1067	378034	0.948	-0.05299	0.161504	0.691	1.3	0.742829	0.952697
Operations and Procedures	Other operations on penis	T	378957	452	378649	1.08	0.077189	0.234862	0.682	1.71	0.742417	0.952697
Operations and Procedures	Other operations on subcutaneous tissue	T	378957	494	378607	0.924	-0.07866	0.240375	0.577	1.48	0.743491	0.952697
Summary	Delivery (HES)	T	378957	2450	376651	0.966	-0.03445	0.107352	0.783	1.19	0.748261	0.95278
Haematology	Acute posthaemorrhagic anaemia (HES)	T	378957	226	378875	1.11	0.103539	0.323725	0.588	2.09	0.749093	0.95278
Genitourinary	Vulvovaginal ulceration and inflammation (HES)	T	378957	202	378899	1.12	0.110329	0.341402	0.572	2.18	0.746571	0.95278
Genitourinary	Erosion and ectropion of cervix uteri (HES)	T	378957	830	378271	0.943	-0.05817	0.180933	0.662	1.35	0.747839	0.95278
Genitourinary	Menopausal and other perimenopausal disorders (HES)	T	378957	9413	369688	1.02	0.017315	0.053625	0.916	1.13	0.746785	0.95278
Medication	Other antibacterials	T	378957	1343	377758	0.955	-0.04586	0.143071	0.722	1.26	0.748576	0.95278
Operations and Procedures	Achilles tendon repair (self-reported)	T	378957	613	378488	0.934	-0.06813	0.212809	0.616	1.42	0.748847	0.95278
Neurosciences	Neck shoulder pain for 3 months vs no pain	T	344799	58990	285942	1.01	0.00743	0.02319	0.963	1.05	0.748682	0.95278
Operations and Procedures	Other open operations on valve of heart	T	378957	301	378800	0.905	-0.09954	0.307443	0.496	1.65	0.746123	0.95278
Operations and Procedures	Diagnostic transluminal operations on coronary artery	T	378957	808	378293	0.942	-0.05975	0.186415	0.654	1.36	0.748574	0.95278
Operations and Procedures	Diagnostic transluminal operations on vein	T	378957	931	378170	1.05	0.053187	0.163886	0.765	1.45	0.745533	0.95278
Operations and Procedures	Diagnostic testing of genitourinary system	T	378957	578	378523	1.07	0.067699	0.208791	0.711	1.61	0.745754	0.95278
Eye	Retinal problem (HES and self-reported)	T	378957	7219	371882	1.02	0.019224	0.060062	0.906	1.15	0.748918	0.95278
Biological assays	White blood cell leukocyte count	T	367717	NA	NA	NA	0.002733	0.008435	-0.0138	0.019266	0.745932	0.95278
Other	Snoring	T	352620	131409	221344	0.994	-0.00581	0.018225	0.959	1.03	0.74975	0.95282
Gynaecology and Obstetrics	Fibrocystic disease (HES and self-reported)	T	378957	1743	377358	0.961	-0.03989	0.125136	0.752	1.23	0.749916	0.95282
Medication	Vitamin b12 and folic acid	T	378957	8262	370839	0.982	-0.01823	0.057815	0.877	1.1	0.752531	0.953127
Medication	Thyroid preparations	T	378957	21493	357608	1.01	0.011406	0.036193	0.942	1.09	0.752664	0.953127
Gynaecology and Obstetrics	Labour and delivery complicated by fetal stress (HES)	T	378957	3031	376070	1.03	0.029822	0.094742	0.856	1.24	0.75294	0.953127
Mental health	Probable recurrent major depression moderate	T	58804	11697	47141	0.983	-0.01701	0.054066	0.884	1.09	0.75304	0.953127
Operations and Procedures	Other therapeutic transluminal operations on coronary artery	T	378957	858	378243	1.06	0.053583	0.170787	0.755	1.47	0.753718	0.953127
Operations and Procedures	Prosthetic replacement of head of femur using cement	T	378957	222	378879	0.893	-0.11313	0.36043	0.441	1.81	0.753616	0.953127
Respiratory	Respiratory failure (HES and self-reported)	T	378957	1951	377150	0.964	-0.0371	0.118243	0.764	1.21	0.753713	0.953127
Genitourinary	Renalkidney failure (HES and self-reported)	T	378957	8356	370745	1.02	0.017887	0.056279	0.912	1.14	0.750616	0.953127
Musculoskeletal	Fracture patella knee (HES and self-reported)	T	378957	515	378586	0.929	-0.07349	0.233992	0.587	1.47	0.753463	0.953127
Operations and Procedures	Other operations on ventricle of brain	T	378957	227	378874	0.894	-0.1125	0.360157	0.441	1.81	0.754764	0.953764
Respiratory	GOLD tobin	T	18298	NA	NA	NA	-0.01153	0.036954	-0.08396	0.060898	0.755014	0.953764
Digestive system	Intestinal malabsorption (HES)	T	378957	1888	377213	0.964	-0.03717	0.119728	0.762	1.22	0.756216	0.954269
Genitourinary	Neuromuscular dysfunction of bladder (HES)	T	378957	1508	377593	1.04	0.040621	0.131059	0.806	1.35	0.756602	0.954269
Operations and Procedures	Operations on frontal sinus	T	378957	1944	377157	1.04	0.035805	0.115228	0.827	1.3	0.756003	0.954269
Medication	Antipropulsives	T	378957	2041	377060	0.965	-0.03527	0.115111	0.77	1.21	0.759299	0.954769
ENT	Otosclerosis (HES)	T	378957	275	378826	0.905	-0.09935	0.322356	0.481	1.7	0.757926	0.954769
Respiratory	Chronic sinusitis (HES)	T	378957	2225	376876	0.967	-0.03383	0.110955	0.778	1.2	0.760471	0.954769
Immuno-inflammation	Urticaria (HES)	T	378957	328	378773	1.09	0.084003	0.273388	0.636	1.86	0.758641	0.954769
Immuno-inflammation	Follicular cysts of skin and subcutaneous tissue (HES)	T	378957	7583	371518	1.02	0.018033	0.059164	0.907	1.14	0.760518	0.954769
Symptoms	Malaise and fatigue (HES)	T	378957	2616	376485	1.03	0.030342	0.099569	0.848	1.25	0.760565	0.954769
Digestive system	Oesophageal stricture (HES and self-reported)	T	378957	1781	377320	1.04	0.036804	0.119943	0.82	1.31	0.758961	0.954769
Cardiovascular	Aortic aneurysm (HES and self-reported)	T	378957	1220	377881	1.04	0.043481	0.142499	0.79	1.38	0.760267	0.954769
Other	NumCigarettes	T	111849	NA	NA	NA	0.004756	0.015477	-0.02558	0.035091	0.758596	0.954769
Gynaecology and Obstetrics	Cervix surgery (self-reported)	T	378957	1570	377531	1.04	0.038526	0.127241	0.81	1.33	0.762059	0.955469
Operations and Procedures	Other therapeutic transluminal operations on heart	T	378957	1372	377729	0.958	-0.04247	0.140413	0.728	1.26	0.762313	0.955469
Metabolic	Parathyroid gland problem not cancer (HES and self-reported)	T	378957	274	378827	1.09	0.089505	0.295471	0.613	1.95	0.761948	0.955469
Medication	Digestives incl enzymes	T	378957	255	378846	1.1	0.093133	0.308479	0.6	2.01	0.76272	0.955482
Summary	Influenza and pneumonia (HES)	T	378957	9013	370088	0.984	-0.01651	0.055066	0.883	1.1	0.7643	0.955507
Cardiovascular	Occlusion and stenosis not cerebral infarction (HES)	T	378957	928	378173	0.95	-0.05172	0.172778	0.677	1.33	0.764696	0.955507
Operations and Procedures	Clearance of external auditory canal	T	378957	325	378776	0.915	-0.08851	0.294377	0.514	1.63	0.76367	0.955507
Operations and Procedures	Total excision of bone	T	378957	1188	377913	0.956	-0.04497	0.150771	0.711	1.28	0.765516	0.955507
Operations and Procedures	Other open operations on joint	T	378957	1273	377828	0.957	-0.04363	0.145979	0.719	1.27	0.765047	0.955507
Medication	Ranitidine e g Zantac	T	374624	7205	367563	0.982	-0.0185	0.061906	0.869	1.11	0.765024	0.955507
Digestive system	Oesophageal disorder (HES and self-reported)	T	378957	9080	370021	1.02	0.016207	0.054269	0.914	1.13	0.765214	0.955507
Neurosciences	Polyneuropathy in diseases classified elsewhere (HES)	T	378957	353	378748	0.919	-0.08419	0.282866	0.528	1.6	0.765988	0.955601
Musculoskeletal	Muscle or soft tissue injuries (HES and self-reported)	T	378957	9386	369715	1.02	0.015801	0.053295	0.915	1.13	0.766861	0.956195
Medication	Antifungals for topical use	T	378957	3710	375391	0.975	-0.02522	0.085752	0.824	1.15	0.768705	0.957008
Respiratory	Other pleural conditions (HES)	T	378957	242	378859	0.905	-0.10009	0.339991	0.465	1.76	0.768452	0.957008
Cardiovascular	LVESVi	T	3760	NA	NA	NA	0.026442	0.089813	-0.14959	0.202475	0.768439	0.957008
Operations and Procedures	Incision of capsule of lens	T	378957	1497	377604	0.961	-0.03932	0.134397	0.739	1.25	0.76985	0.957013
Anthropometry	Abdominal subcutaneous adipose tissue volume ASAT	T	801	NA	NA	NA	0.058453	0.199835	-0.33322	0.450129	0.769901	0.957013
Biological assays	Mean corpuscular volume	T	367720	NA	NA	NA	0.002468	0.008419	-0.01403	0.01897	0.769429	0.957013
Genitourinary	Renal failure not requiring dialysis (HES and self-reported)	T	378957	483	378618	0.932	-0.07009	0.240482	0.582	1.49	0.770713	0.957528
Summary	Other degenerative diseases of the nervous system (HES)	T	378957	907	378194	1.05	0.047765	0.166135	0.757	1.45	0.773725	0.958762
Summary	Dermatitis and eczema (HES)	T	378957	2862	376239	0.972	-0.02792	0.097624	0.803	1.18	0.774869	0.958762
Infectious disease	Zoster (HES)	T	378957	425	378676	1.07	0.069363	0.241136	0.668	1.72	0.773613	0.958762
Infectious disease	Viral infection of unspecified site (HES)	T	378957	1590	377511	1.04	0.036272	0.126977	0.808	1.33	0.77514	0.958762
Eye	Other inflammation of eyelid (HES)	T	378957	538	378563	1.06	0.060959	0.213397	0.7	1.61	0.77514	0.958762
Cardiovascular	Haemorrhoids (HES)	T	378957	21509	357592	1.01	0.01026	0.035885	0.942	1.08	0.774943	0.958762
Eye	Squint correction (self-reported)	T	378957	5064	374037	0.979	-0.02099	0.073016	0.849	1.13	0.773757	0.958762
Operations and Procedures	Other operations on eye	T	378957	768	378333	1.05	0.05161	0.180877	0.739	1.5	0.775389	0.958762
Operations and Procedures	Release of organ NOC	T	378957	896	378205	0.951	-0.0499	0.175123	0.675	1.34	0.775686	0.958762
Medication	Vitamin C	T	376892	32744	344292	1.01	0.00855	0.029535	0.952	1.07	0.772209	0.958762
Metabolic	Deficiency of other nutrient elements (HES)	T	378957	216	378885	0.904	-0.10134	0.360498	0.446	1.83	0.778618	0.959796
Mental health	Schizophrenia (HES)	T	378957	496	378605	0.936	-0.06584	0.240328	0.585	1.5	0.784126	0.959796
Cardiovascular	Hypertensive renal disease (HES)	T	378957	1396	377705	1.04	0.037484	0.134569	0.798	1.35	0.780593	0.959796
Respiratory	Bacterial pneumonia (HES)	T	378957	433	378668	0.931	-0.07194	0.255013	0.565	1.53	0.777869	0.959796
Immuno-inflammation	Rosacea (HES)	T	378957	277	378824	0.914	-0.08941	0.322314	0.486	1.72	0.781475	0.959796
Musculoskeletal	Other acquired deformities of limbs (HES)	T	378957	1540	377561	0.964	-0.03653	0.132031	0.744	1.25	0.782045	0.959796
Genitourinary	Unspecified lump in breast (HES)	T	378957	1403	377698	1.04	0.037211	0.13462	0.797	1.35	0.782226	0.959796
Genitourinary	Other noninflammatory disorders of vulva and perineum (HES)	T	378957	1719	377382	1.03	0.034338	0.121787	0.815	1.31	0.777983	0.959796
Other	Poisoning by diuretics and other drugs (HES)	T	378957	335	378766	0.921	-0.0827	0.294243	0.517	1.64	0.778665	0.959796
Medication	Other drugs for disorders of the musculo skeletal system	T	378957	2679	376422	1.03	0.027468	0.09795	0.848	1.25	0.779149	0.959796
Mental health	Single episode of probable major depression	T	54605	7501	47141	0.982	-0.0178	0.064188	0.866	1.11	0.781492	0.959796
Neurosciences	Stomach abdominal pain for 3 months vs no pain	T	357669	17535	340271	1.01	0.010926	0.039703	0.935	1.09	0.783175	0.959796
Operations and Procedures	Destruction of peripheral nerve	T	378957	243	378858	0.91	-0.09379	0.339905	0.468	1.77	0.782604	0.959796
Operations and Procedures	Buckling operations for attachment of retina	T	378957	659	378442	0.945	-0.05701	0.204201	0.633	1.41	0.780201	0.959796
Operations and Procedures	Extended excision of right hemicolon	T	378957	250	378851	1.09	0.084626	0.30865	0.594	1.99	0.783945	0.959796
Operations and Procedures	Early operations NOC	T	378957	2989	376112	1.03	0.02541	0.092931	0.855	1.23	0.784528	0.959796
Operations and Procedures	Radiopharmaceutical imaging	T	378957	249	378852	0.91	-0.09385	0.339741	0.468	1.77	0.782371	0.959796
Immuno-inflammation	Acneacne vulgaris (HES and self-reported)	T	378957	277	378824	0.914	-0.09029	0.322336	0.486	1.72	0.77939	0.959796
Cardiovascular	Aortic regurgitation incompetence (HES and self-reported)	T	378957	711	378390	1.05	0.052752	0.186897	0.731	1.52	0.77775	0.959796
Musculoskeletal	Fracture vertebra crush fracture vertebral col (HES and self-reported)	T	378957	1036	378065	1.04	0.042599	0.156065	0.769	1.42	0.784889	0.959796
NA	Adult only asthma over16yo (self-reported)	T	367054	31918	335270	0.992	-0.00828	0.030154	0.935	1.05	0.783627	0.959796
Operations and Procedures	Introduction of radioactive material into organ NOC	T	378957	376	378725	0.928	-0.07424	0.27265	0.544	1.58	0.785405	0.959939
Operations and Procedures	Other operations on ossicle of ear	T	378957	327	378774	0.924	-0.07939	0.29434	0.519	1.64	0.787365	0.961847
Summary	Mal neo of female genital organs (cancer register)	T	378957	2734	376367	0.974	-0.02675	0.099401	0.801	1.18	0.787836	0.961935
Operations and Procedures	Abdominal operations to support outlet of female bladder	T	378957	1021	378080	1.04	0.042288	0.158102	0.765	1.42	0.789107	0.962998
Cardiovascular	Hypotension (HES)	T	378957	4415	374686	0.979	-0.02085	0.078165	0.84	1.14	0.789654	0.963046
Operations and Procedures	Anal fissure surgery (self-reported)	T	378957	526	378575	0.941	-0.0606	0.228235	0.602	1.47	0.790611	0.963046
Operations and Procedures	Mri magnetic resonance imaging (self-reported)	T	378957	522	378579	0.941	-0.0605	0.228247	0.602	1.47	0.790961	0.963046
Operations and Procedures	Excision of left hemicolon	T	378957	330	378771	0.925	-0.07825	0.294337	0.519	1.65	0.790355	0.963046
Operations and Procedures	Operations on Bartholin gland	T	378957	599	378502	1.06	0.054229	0.204775	0.707	1.58	0.791145	0.963046
ENT	Conductive and sensorineural hearing loss (HES)	T	378957	866	378235	0.955	-0.04612	0.177756	0.674	1.35	0.795271	0.964093
Cardiovascular	Cardiomyopathy (HES)	T	378957	1163	377938	0.961	-0.03962	0.152404	0.713	1.3	0.794878	0.964093
Genitourinary	Acute renal failure (HES)	T	378957	4117	374984	0.979	-0.02102	0.081118	0.835	1.15	0.795517	0.964093
Medication	Topical products for joint and muscular pain	T	378957	62133	316968	0.994	-0.00593	0.022616	0.951	1.04	0.793333	0.964093
Cardiovascular	Fem pop bypass leg artery bypass (self-reported)	T	378957	221	378880	0.91	-0.09438	0.360493	0.449	1.84	0.793476	0.964093
Cardiovascular	Cerebral artery aneurysm surgery or clipping (self-reported)	T	378957	222	378879	0.911	-0.09334	0.3603	0.45	1.85	0.79559	0.964093
ENT	Throat larynx surgery incl tracheostomy (self-reported)	T	378957	2251	376850	1.03	0.02818	0.107417	0.833	1.27	0.793054	0.964093

Operations and Procedures	Brain surgery (self-reported)	T		378957	1560	377541	1.03	0.033148	0.127966	0.804	1.33	0.795607	0.964093
Musculoskeletal	Fracture jaw (HES and self-reported)	T		378957	790	378311	1.05	0.046881	0.180805	0.735	1.49	0.795412	0.964093
Immuno-inflammation	Hypertrophic disorders of skin (HES)	T		378957	1003	378098	1.04	0.041167	0.159747	0.762	1.43	0.796639	0.964479
Operations and Procedures	Excision of tendon	T		378957	253	378848	1.08	0.07947	0.308517	0.591	1.98	0.796726	0.964479
Immuno-inflammation	Blisteringdesquamating skin disorder (HES and self-reported)	T		378957	748	378353	1.05	0.047131	0.183704	0.731	1.5	0.797519	0.964954
Infectious disease	Viral agents as the cause of diseases classified to other chapters (HES)	T		378957	505	378596	0.942	-0.05991	0.234156	0.595	1.49	0.798065	0.96513
Medication	Peripheral vasodilators	T		378957	334	378767	1.07	0.064158	0.273295	0.624	1.82	0.814398	0.9652
Summary	Disorders of thyroid gland (HES)	T		378957	15440	363661	0.99	-0.0103	0.042451	0.911	1.08	0.808328	0.9652
Summary	Organic including symptomatic mental disorders (HES)	T		378957	1447	377654	1.03	0.032531	0.132398	0.797	1.34	0.805909	0.9652
Summary	Other disorders of the nervous system (HES)	T		378957	3742	375359	1.02	0.020741	0.083268	0.867	1.2	0.803291	0.9652
Summary	Respiratory diseases interstitium (HES)	T		378957	1501	377600	0.969	-0.03146	0.134349	0.745	1.26	0.81485	0.9652
Summary	Inflammatory diseases of female pelvic organs (HES)	T		378957	5417	373684	1.02	0.016903	0.069796	0.887	1.17	0.808646	0.9652
Summary	Congenital malformations of the nervous system (HES)	T		378957	249	378852	0.918	-0.0851	0.339739	0.472	1.79	0.802213	0.9652
Summary	Symptoms nervous and musculoskeletal systems (HES)	T		378957	4763	374338	0.982	-0.0181	0.07517	0.848	1.14	0.809702	0.9652
Summary	Symptoms cognition perception emotional state (HES)	T		378957	8138	370963	1.01	0.013745	0.057263	0.906	1.13	0.810303	0.9652
Eye	Conjunctivitis (HES)	T		378957	221	378880	0.914	-0.0904	0.36034	0.451	1.85	0.801903	0.9652
Respiratory	Nasal polyp (HES)	T		378957	2942	376159	0.976	-0.0242	0.096413	0.808	1.18	0.801827	0.9652
Immuno-inflammation	Other dermatitis (HES)	T		378957	1379	377722	0.968	-0.03285	0.140378	0.735	1.27	0.814951	0.9652
Musculoskeletal	Polyarthrosis (HES)	T		378957	3571	375530	0.98	-0.02057	0.087157	0.826	1.16	0.813402	0.9652
Genitourinary	Inflammatory disorders of breast (HES)	T		378957	430	378671	0.939	-0.06264	0.25511	0.57	1.55	0.806036	0.9652
Gynaecology and Obstetrics	Maternal care fetal abnormality and damage (HES)	T		378957	238	378863	0.922	-0.08074	0.341038	0.473	1.8	0.812845	0.9652
Symptoms	Abnormal involuntary movements (HES)	T		378957	819	378282	0.956	-0.04519	0.183361	0.667	1.37	0.805344	0.9652
Symptoms	Symptoms and signs concerning food and fluid intake (HES)	T		378957	5849	373252	1.02	0.01613	0.067062	0.891	1.16	0.80993	0.9652
Gynaecology and Obstetrics	Vaginal prolapse colposuspension (self-reported)	T		378957	3479	375622	0.979	-0.02167	0.088467	0.823	1.16	0.8065	0.9652
Eye	Retinal operation vitrectomy (self-reported)	T		378957	2253	376848	0.974	-0.02651	0.109207	0.786	1.21	0.808168	0.9652
Operations and Procedures	Male circumcision (self-reported)	T		378957	3497	375604	1.02	0.020506	0.08753	0.86	1.21	0.814771	0.9652
Gynaecology and Obstetrics	Ovarian cyst removal surgery (self-reported)	T		378957	4517	374584	0.982	-0.01864	0.077352	0.843	1.14	0.809525	0.9652
Operations and Procedures	Prostate biopsy (self-reported)	T		378957	258	378843	0.924	-0.07943	0.323119	0.49	1.74	0.805813	0.9652
Operations and Procedures	Other operations on rectum through anus	T		378957	648	378453	0.952	-0.049	0.204238	0.638	1.42	0.810396	0.9652
Operations and Procedures	Dilation of anal sphincter	T		378957	444	378657	1.06	0.057297	0.240841	0.661	1.7	0.811955	0.9652
Operations and Procedures	Urinary diversion	T		378957	327	378774	1.07	0.069769	0.273464	0.627	1.83	0.798621	0.9652
Operations and Procedures	Extirpation of scrotum	T		378957	239	378862	1.08	0.077638	0.323468	0.573	2.04	0.810316	0.9652
Operations and Procedures	Surgical induction of labour	T		378957	2296	376805	0.974	-0.02592	0.110718	0.784	1.21	0.814894	0.9652
Operations and Procedures	Exploration of other skin of other site	T		378957	4464	374637	0.981	-0.01897	0.077855	0.842	1.14	0.807494	0.9652
Operations and Procedures	Rehabilitation for musculoskeletal disorders	T		378957	364	378737	1.06	0.061768	0.255964	0.644	1.76	0.809312	0.9652
Operations and Procedures	Closed reduction of fracture of bone and internal fixation	T		378957	2838	376263	0.976	-0.02452	0.097659	0.806	1.18	0.801789	0.9652
Operations and Procedures	Prosthetic replacement of other bone using cement	T		378957	559	378542	1.05	0.051737	0.208975	0.699	1.59	0.804464	0.9652
Operations and Procedures	Injection of radiocontrast material	T		378957	1644	377457	1.03	0.030272	0.125997	0.805	1.32	0.810132	0.9652
Operations and Procedures	Radiotherapy delivery	T		378957	2124	376977	0.973	-0.02778	0.111802	0.781	1.21	0.803742	0.9652
Operations and Procedures	Laser therapy to organ NOC	T		378957	2987	376114	0.978	-0.02231	0.094785	0.812	1.18	0.813957	0.9652
Operations and Procedures	Introduction of non removable material into organ NOC	T		378957	611	378490	0.95	-0.051	0.212925	0.626	1.44	0.810707	0.9652
Digestive system	Hepatitis (HES and self-reported)	T		378957	1662	377439	1.03	0.0312	0.125058	0.807	1.32	0.802987	0.9652
Genitourinary	Urinary frequency incontinence (HES and self-reported)	T		378957	11734	367367	1.01	0.011688	0.047861	0.921	1.11	0.807073	0.9652
ENT	Menieres disease (HES and self-reported)	T		378957	1305	377796	1.04	0.034761	0.13933	0.788	1.36	0.802981	0.9652
Musculoskeletal	Plantar fascitis (HES and self-reported)	T		378957	448	378653	1.06	0.057064	0.234671	0.668	1.68	0.807877	0.9652
Cardiovascular	Mitral regurgitation incompetence (HES and self-reported)	T		378957	2024	377077	0.973	-0.02755	0.115254	0.776	1.22	0.81109	0.9652
Other	Daytime dozing sleeping narcolepsy	T		377408	NA	NA	NA	0.00203	0.008315	-0.01427	0.018328	0.80715	0.9652
Biological assays	Eosinophill percentage	T		367073	NA	NA	NA	-0.002	0.008444	-0.01855	0.014552	0.812982	0.9652
Cancer	Mal neo rectosigmoid (cancer register)	T		378957	343	378758	0.936	-0.06589	0.283046	0.538	1.63	0.815925	0.965879
Genitourinary	Prostatitis (HES and self-reported)	T		378957	1555	377546	0.97	-0.03068	0.132459	0.748	1.26	0.816852	0.966501
Gynaecology and Obstetrics	Single spontaneous delivery (HES)	T		378957	1836	377265	1.03	0.027535	0.119818	0.813	1.3	0.81824	0.967478
Symptoms	Abnormal blood pressure reading without diagnosis (HES)	T		378957	2292	376809	1.02	0.024117	0.105756	0.833	1.26	0.819613	0.967478
Operations and Procedures	Fixation of retina	T		378957	1034	378067	1.04	0.035198	0.154414	0.765	1.4	0.819686	0.967478
Operations and Procedures	Excision of pilonidal sinus	T		378957	253	378848	0.925	-0.07773	0.339738	0.475	1.8	0.819019	0.967478
Operations and Procedures	Harvest of skin for graft	T		378957	2280	376821	1.02	0.02425	0.105806	0.833	1.26	0.818722	0.967478
Operations and Procedures	Other operations on internal nose	T		378957	2843	376258	1.02	0.021803	0.096073	0.847	1.23	0.82047	0.96793
Operations and Procedures	Excision of lesion of anus	T		378957	2708	376393	0.978	-0.02252	0.099913	0.804	1.19	0.821676	0.968484
ENT	Throat or larynx disorder (HES and self-reported)	T		378957	3484	375617	0.98	-0.01995	0.08853	0.824	1.17	0.821744	0.968484
Genitourinary	Other disorders of penis (HES)	T		378957	1896	377205	1.03	0.026298	0.11708	0.816	1.29	0.822279	0.968641
Eye	Blindness and low vision (HES)	T		378957	805	378296	1.04	0.038139	0.178045	0.733	1.47	0.830386	0.971371
Respiratory	Chronic rhinitis nasopharyngitis and pharyngitis (HES)	T		378957	770	378331	0.96	-0.04083	0.189544	0.662	1.39	0.829428	0.971371
Respiratory	Other chronic obstructive pulmonary disease (HES)	T		378957	7608	371493	1.01	0.012753	0.059627	0.901	1.14	0.830645	0.971371
Digestive system	Other diseases of appendix (HES)	T		378957	242	378859	0.928	-0.07425	0.339915	0.477	1.81	0.827083	0.971371
Genitourinary	Other congenital malformations of kidney (HES)	T		378957	214	378887	0.926	-0.07715	0.360552	0.457	1.88	0.830565	0.971371
ENT	Ear surgery (self-reported)	T		378957	6011	373090	1.01	0.014546	0.06631	0.891	1.16	0.826369	0.971371
Operations and Procedures	Total excision of colon and rectum	T		378957	212	378889	1.08	0.073025	0.340854	0.552	2.1	0.830359	0.971371
Operations and Procedures	Excision of bone	T		378957	3651	375450	1.02	0.018185	0.084374	0.863	1.2	0.829352	0.971371
Operations and Procedures	Other operations on tendon	T		378957	780	378321	0.96	-0.04037	0.186449	0.666	1.38	0.828564	0.971371
Operations and Procedures	Other total prosthetic replacement of hip joint	T		378957	1064	378037	0.966	-0.03482	0.157848	0.709	1.32	0.825426	0.971371
Operations and Procedures	Approach through thoracic cavity	T		378957	602	378499	0.955	-0.0461	0.212903	0.629	1.45	0.828563	0.971371
ENT	Dentures	T		377502	63135	314511	0.995	-0.00514	0.023401	0.95	1.04	0.826112	0.971371
Eye	Glaucoma (HES and self-reported)	T		378957	6262	372839	0.986	-0.01424	0.065242	0.868	1.12	0.827164	0.971371
Neurosciences	Fluid intelligence score	T		184451	NA	NA	NA	0.002543	0.011757	-0.0205	0.025587	0.828735	0.971371
Biological assays	Potassium in urine	T		367300	NA	NA	NA	-0.00182	0.008452	-0.01839	0.014743	0.829212	0.971371
Summary	Noninflammatory disorders of female genital tract (HES)	T		378957	43955	335146	1.01	0.005846	0.027441	0.953	1.06	0.8313	0.971665
Symptoms	Signs involving the nervous and musculoskeletal systems (HES)	T		378957	2611	376490	1.02	0.021051	0.099168	0.841	1.24	0.831894	0.971888
Eye	Hordeolum and chalazion (HES)	T		378957	1665	377436	0.973	-0.027	0.127797	0.758	1.25	0.832702	0.971889
Haematology	Myeloproliferative disorder (HES and self-reported)	T		378957	455	378646	1.05	0.04958	0.234689	0.663	1.66	0.832685	0.971889
Musculoskeletal	Other osteochondropathies (HES)	T		378957	239	378862	0.931	-0.07103	0.34003	0.478	1.81	0.834531	0.97214
Genitourinary	Unspecified nephritic syndrome (HES)	T		378957	244	378857	0.931	-0.07135	0.339873	0.478	1.81	0.833711	0.97214
ENT	Hearing difficulty problems with background noise	T		371341	140624	230856	1	0.003673	0.017558	0.97	1.04	0.834277	0.97214
Operations and Procedures	Vaginal excision of uterus	T		378957	4716	374385	1.02	0.015632	0.074643	0.878	1.18	0.834117	0.97214
Medication	Other beta lactam antibacterials	T		378957	374	378727	0.945	-0.05678	0.272697	0.554	1.61	0.835057	0.972282
Respiratory	Pneumonia organism unspecified (HES)	T		378957	8203	370898	0.988	-0.01182	0.057505	0.883	1.11	0.837091	0.973236
Gynaecology and Obstetrics	Sterilisation (self-reported)	T		378957	16084	363017	1.01	0.008549	0.041751	0.929	1.09	0.837756	0.973236
Operations and Procedures	Fixation of bone of face	T		378957	219	378882	1.07	0.069643	0.340675	0.55	2.09	0.838021	0.973236
Metabolic	Parathyroid hyperplasiaadenoma (HES and self-reported)	T		378957	419	378682	0.949	-0.05249	0.255217	0.575	1.56	0.837059	0.973236
Musculoskeletal	Giant celltemporal arteritis (HES and self-reported)	T		378957	395	378706	0.948	-0.05379	0.263606	0.565	1.59	0.8383	0.973236
Genitourinary	Diabetic nephropathy (HES and self-reported)	T		378957	242	378859	0.933	-0.06978	0.339941	0.479	1.82	0.837368	0.973236
Summary	Radiation related disorders of the skin and subcutaneous tissue (HES)	T		378957	2288	376813	1.02	0.021342	0.105847	0.83	1.26	0.840204	0.974276
Neurosciences	Spinal muscular atrophy and related syndromes (HES)	T		378957	213	378888	0.93	-0.07228	0.360644	0.459	1.89	0.841161	0.974276
Mental health	Single Probable major depression episode	T		91467	6104	85411	1.01	0.013443	0.066902	0.889	1.16	0.840748	0.974276
Operations and Procedures	Operations on gingiva	T		378957	429	378672	0.95	-0.0511	0.255051	0.576	1.57	0.841218	0.974276
Operations and Procedures	Procurement of drugs for chemotherapy for neoplasm in Bands 1 5	T		378957	5730	373371	1.01	0.013499	0.067238	0.888	1.16	0.84088	0.974276
Summary	Renal failure (HES)	T		378957	7743	371358	1.01	0.011702	0.058561	0.902	1.13	0.841623	0.974277
Metabolic	Hyperfunction of pituitary gland (HES)	T		378957	291	378810	0.941	-0.06119	0.3076	0.515	1.72	0.842308	0.974601
Summary	Cerebral palsy and other paralytic syndromes (HES)	T		378957	2036	377065	1.02	0.020579	0.113108	0.818	1.27	0.855632	0.975807
Summary	Other disorders of eye and adnexa (HES)	T		378957	876	378225	1.03	0.030599	0.170483	0.738	1.44	0.857557	0.975807
Summary	Noninfective enteritis and colitis (HES)	T		378957	17546	361555	1.01	0.007198	0.039528	0.932	1.09	0.855513	0.975807
Summary	Renal tubulo interstitial diseases (HES)	T		378957	3453	375648	1.02	0.015543	0.08703	0.856	1.2	0.858257	0.975807
Summary	HESCH Diseases of the digestive system (HES)	T		378957	137916	241185	0.997	-0.00333	0.01754	0.963	1.03	0.849265	0.975807
Infectious disease	Streptococcus and staphylococcus (HES)	T		378957	3735	375366	1.02	0.015454	0.083564	0.862	1.2	0.853277	0.975807
Eye	Keratitis (HES)	T		378957	236	378865	1.06	0.058344	0.323379	0.562	2	0.856822	0.975807
ENT	Other disorders of middle ear and mastoid (HES)	T		378957	400	378701	0.951	-0.05042	0.263361	0.567	1.59	0.84818	0.975807
Cardiovascular	Other cardiac arrhythmias (HES)	T		378957	2381	376720	1.02	0.019923	0.104149	0.832	1.25	0.848294	0.975807
Digestive system	Other noninfective gastroenteritis and colitis (HES)	T		378957	14793	364308	1.01	0.007627	0.042892	0.926	1.1	0.858866	0.975807
Musculoskeletal	Other specific joint derangements (HES)	T		378957	2363	376738	0.98	-0.02042	0.10672	0.795	1.21	0.848255	0.975807
Musculoskeletal	Osteopathies in diseases classified elsewhere (HES)	T		378957	292	378809	0.942	-0.05936	0.307582	0.516	1.72	0.846971	0.975807
Symptoms	Dysphagia (HES)	T		378957	5765	373336	1.01	0.012907	0.067614	0.887	1.16	0.848606	0.975807
Medication	Antiinfectives	T		378957	956	378145	1.03	0.029394	0.163781	0.747	1.42	0.85757	0.975807
Cancer	Testicular cancer (self-reported)	T		378957	670	378431	1.04	0.036968	0.196958	0.705	1.53	0.851115	0.975807
Operations and Procedures	Percutaneous open kidney stone surgery lithotripsy (self-reported)	T		378957	2177	376924	0.979	-0.02121	0.111096	0.787	1.22	0.848623	0.975807
Operations and Procedures	Bowel resection (self-reported)	T		378957	706	378395	1.03	0.03411	0.189901	0.713	1.5	0.857452	0.975807
Family history	Lung cancer (family history - father)	T		338597	30466	308263	0.994	-0.00598	0.030917	0.936	1.06	0.84656	0.975807
Family history	Prostate cancer (family history - sibling)	T		301109	4736	296500	0.986	-0.01425	0.075252	0.851	1.14	0.849783	0.975807
Operations and Procedures	Extirpation of lesion of peripheral nerve	T		378957	1390	377711	0.975	-0.02557	0.139171	0.742	1.28	0.854233	0.975807
Operations and Procedures	Extirpation of lesion of eyelid	T		378957	5517	373584	0.987	-0.01284	0.069974	0.861	1.13	0.854353	0.975807

Operations and Procedures	Recession of muscle of eye	T		378957	270	378831	0.941	-0.06065	0.322482	0.5	1.77	0.85082	0.975807
Operations and Procedures	Diagnostic fibreoptic endoscopic examination of oesophagus	T		378957	1468	377633	0.976	-0.02403	0.135432	0.749	1.27	0.859148	0.975807
Operations and Procedures	Therapeutic fibreoptic gastrointestinal tract	T		378957	1244	377857	0.973	-0.02737	0.147545	0.729	1.3	0.852826	0.975807
Operations and Procedures	Other open operations on other artery	T		378957	320	378781	0.944	-0.05786	0.294472	0.53	1.68	0.844239	0.975807
Operations and Procedures	Diagnostic transluminal operations on other artery	T		378957	397	378704	1.05	0.045669	0.255503	0.634	1.73	0.858141	0.975807
Operations and Procedures	Total excision of kidney	T		378957	1063	378038	0.97	-0.03092	0.159595	0.709	1.33	0.846358	0.975807
Operations and Procedures	Diagnostic endoscopic examination of ureter	T		378957	1627	377474	0.977	-0.0237	0.128852	0.759	1.26	0.85409	0.975807
Operations and Procedures	Therapeutic endoscopic operations on pleura	T		378957	310	378791	1.06	0.053653	0.283639	0.605	1.84	0.849968	0.975807
Operations and Procedures	Complex reconstruction of forefoot	T		378957	800	378301	0.966	-0.03471	0.183553	0.674	1.38	0.850015	0.975807
Operations and Procedures	External resuscitation	T		378957	3496	375605	1.02	0.016051	0.086209	0.858	1.2	0.852297	0.975807
Operations and Procedures	Cytology of organ NOC	T		378957	2554	376547	0.981	-0.01926	0.102928	0.802	1.2	0.851543	0.975807
Mental health	Ever unenthusiastic disinterested for a whole week	T		121342	44857	76548	0.994	-0.00575	0.030485	0.937	1.06	0.85038	0.975807
Medication	Zinc	T		377706	15460	362390	0.993	-0.0075	0.042171	0.914	1.08	0.858865	0.975807
Metabolic	Disorder or pituitary gland (HES and self-reported)	T		378957	409	378692	1.05	0.047435	0.247946	0.645	1.7	0.848283	0.975807
Digestive system	Duodenal ulcer (HES and self-reported)	T		378957	4066	375035	1.02	0.015891	0.081068	0.867	1.19	0.844597	0.975807
Immuno-inflammation	Lichen sclerosis (HES and self-reported)	T		378957	542	378559	0.96	-0.04109	0.222918	0.62	1.49	0.853745	0.975807
Infectious disease	Rubella german measles (HES and self-reported)	T		378957	452	378649	0.957	-0.04405	0.240798	0.597	1.53	0.854847	0.975807
Cardiovascular	Cardiac index during PWA	T		7999	NA	NA	NA	-0.01144	0.059948	-0.12894	0.106056	0.848633	0.975807
Haematology	Vitamin B12 deficiency anaemia (HES)	T		378957	809	378292	1.03	0.031107	0.178047	0.728	1.46	0.861308	0.977339
Haematology	Other coagulation defects (HES)	T		378957	874	378227	1.03	0.029809	0.170477	0.738	1.44	0.861193	0.977339
Summary	Other and unspecified disorders of the circulatory system (HES)	T		378957	4939	374162	0.987	-0.01278	0.073699	0.855	1.14	0.862279	0.977543
Cardiovascular	Varicose ulcer (HES and self-reported)	T		378957	232	378869	0.943	-0.05901	0.340233	0.484	1.84	0.862299	0.977543
Symptoms	Nausea and vomiting (HES)	T		378957	10633	368468	0.991	-0.00869	0.050798	0.897	1.1	0.86419	0.979226
Summary	Visual disturbances and blindness (HES)	T		378957	3428	375673	1.01	0.014786	0.087342	0.855	1.2	0.865573	0.979527
Operations and Procedures	Other urological surgery (self-reported)	T		378957	990	378111	0.972	-0.02814	0.16572	0.703	1.35	0.865187	0.979527
Operations and Procedures	Laparoscopy (self-reported)	T		378957	6734	372367	0.989	-0.01074	0.063657	0.873	1.12	0.86599	0.979527
Operations and Procedures	Delivery of chemotherapy for neoplasm	T		378957	7579	371522	1.01	0.009917	0.05899	0.9	1.13	0.866489	0.979527
Cardiovascular	LV ejection fraction	T		3764	NA	NA	NA	0.015104	0.089733	-0.16077	0.190979	0.866334	0.979527
Immuno-inflammation	Dermatitis due to substances taken internally (HES)	T		378957	436	378665	1.04	0.040154	0.240942	0.649	1.67	0.867642	0.979911
Operations and Procedures	Other operations on heart	T		378957	733	378368	1.03	0.031165	0.186697	0.716	1.49	0.867427	0.979911
Cardiovascular	Diseases of capillaries (HES)	T		378957	604	378497	0.965	-0.03516	0.212846	0.636	1.47	0.868789	0.980746
Medication	Antipsoriatics for topical use	T		378957	1197	377904	1.02	0.023783	0.14764	0.767	1.37	0.872026	0.981755
Operations and Procedures	Total prosthetic knee joint not using cement	T		378957	695	378406	1.03	0.030811	0.190054	0.711	1.5	0.871215	0.981755
Eye	Ever had eye surgery	T		84638	6217	78456	0.989	-0.01087	0.06688	0.868	1.13	0.870906	0.981755
Mental health	Maniabipolar disordermanic depression (HES and self-reported)	T		378957	1595	377506	1.02	0.020588	0.127911	0.794	1.31	0.872128	0.981755
Musculoskeletal	Fracture clavicle collar bone (HES and self-reported)	T		378957	1092	378009	0.975	-0.0255	0.157712	0.716	1.33	0.871549	0.981755
Eye	LogMAR final left	T		83932	NA	NA	NA	-0.00281	0.017453	-0.03702	0.031394	0.8719	0.981755
Cardiovascular	Cerebral aneurysm (HES and self-reported)	T		378957	545	378556	1.04	0.034781	0.217897	0.676	1.59	0.873179	0.982479
Musculoskeletal	Spontaneous rupture of synovium and tendon (HES)	T		378957	488	378613	0.964	-0.03704	0.234263	0.609	1.53	0.874382	0.983374
Medication	Other mineral supplements	T		378957	878	378223	0.973	-0.02737	0.175286	0.69	1.37	0.875922	0.984442
Musculoskeletal	Rheumatoid arthritis (HES and self-reported)	T		378957	6232	372869	0.99	-0.01026	0.06586	0.87	1.13	0.876149	0.984442
Cardiovascular	Other arterial surgery revascularisation procedures (self-reported)	T		378957	268	378833	0.952	-0.0496	0.32252	0.506	1.79	0.877783	0.9849
Operations and Procedures	Opening of skin	T		378957	3649	375452	0.987	-0.01327	0.086046	0.834	1.17	0.877462	0.9849
Cardiovascular	Cardiomyopathy (HES and self-reported)	T		378957	1272	377829	0.978	-0.02227	0.144659	0.737	1.3	0.877658	0.9849
Genitourinary	Cystitis (HES)	T		378957	2647	376454	0.985	-0.01518	0.100966	0.808	1.2	0.880504	0.985821
Gynaecology and Obstetrics	Maternal care for known or suspected malpresentation of fetus (HES)	T		378957	990	378111	0.976	-0.02477	0.164038	0.707	1.35	0.879966	0.985821
Operations and Procedures	Other operations on nail	T		378957	396	378705	1.04	0.03825	0.255439	0.63	1.71	0.880967	0.985821
Neurosciences	Chronicdegenerative neurological problem (HES and self-reported)	T		378957	4087	375014	1.01	0.012176	0.08	0.865	1.18	0.879033	0.985821
Musculoskeletal	Fracture skull head (HES and self-reported)	T		378957	2136	376965	0.983	-0.01702	0.113026	0.788	1.23	0.880287	0.985821
Respiratory	FEV1 FVC ratio ever smoked only strict	T		136671	NA	NA	NA	-0.00208	0.013909	-0.02934	0.02518	0.88106	0.985821
Operations and Procedures	Extirpation of lesion of meninges of brain	T		378957	289	378812	0.955	-0.04581	0.307654	0.523	1.75	0.881623	0.985993
Operations and Procedures	Therapeutic endoscopic operations on ovary	T		378957	717	378384	0.972	-0.02868	0.193502	0.665	1.42	0.882189	0.986094
Gynaecology and Obstetrics	Abnormal smear cervix (HES and self-reported)	T		378957	1903	377198	1.02	0.017191	0.11634	0.81	1.28	0.882532	0.986094
Medication	Bile therapy	T		378957	233	378868	0.96	-0.04111	0.340233	0.493	1.87	0.903829	0.98634
Medication	Other agents acting on the renin angiotensin system	T		378957	16224	362877	0.994	-0.00561	0.041561	0.917	1.08	0.892561	0.98634
Medication	Antiseptics and disinfectants	T		378957	239	378862	0.954	-0.04686	0.340017	0.49	1.86	0.89039	0.98634
Summary	Episodic and paroxysmal disorders (HES)	T		378957	12991	366110	0.994	-0.00651	0.045976	0.908	1.09	0.887481	0.98634
Summary	Disorders of eyelid lacrimal system and orbit (HES)	T		378957	8748	370353	0.993	-0.00752	0.055606	0.89	1.11	0.892389	0.98634
Summary	Other acute lower respiratory infections (HES)	T		378957	7023	372078	1.01	0.007501	0.061692	0.893	1.14	0.903225	0.98634
Summary	Infections of the skin and subcutaneous tissue (HES)	T		378957	9696	369405	0.993	-0.00725	0.053019	0.895	1.1	0.891307	0.98634
Metabolic	Disorders of porphyrin and bilirubin metabolism (HES)	T		378957	367	378734	0.967	-0.03314	0.272771	0.567	1.65	0.90331	0.98634
Neurosciences	Other degenerative diseases of nervous system (HES)	T		378957	567	378534	0.971	-0.02909	0.217776	0.634	1.49	0.893748	0.98634
ENT	Other disorders of ear (HES)	T		378957	566	378535	1.03	0.027551	0.213133	0.677	1.56	0.897148	0.98634
Cardiovascular	Subsequent myocardial infarction (HES)	T		378957	663	378438	1.03	0.025302	0.196975	0.697	1.51	0.897792	0.98634
Cardiovascular	Endocarditis valve unspecified (HES)	T		378957	363	378738	0.962	-0.03854	0.272856	0.564	1.64	0.887686	0.98634
Digestive system	Ulcerative colitis (HES)	T		378957	2940	376161	0.989	-0.01155	0.095533	0.82	1.19	0.903776	0.98634
Musculoskeletal	Postprocedural musculoskeletal disorders (HES)	T		378957	412	378689	1.03	0.030481	0.24793	0.634	1.68	0.902153	0.98634
Gynaecology and Obstetrics	Obstructed labour malpresentation of fetus (HES)	T		378957	382	378719	0.963	-0.03761	0.26512	0.573	1.62	0.887199	0.98634
Symptoms	Other skin changes (HES)	T		378957	565	378536	1.03	0.025914	0.213143	0.676	1.56	0.903232	0.98634
Symptoms	Abnormal findings in specimens from female genital organs (HES)	T		378957	1312	377789	1.02	0.019136	0.139568	0.775	1.34	0.890946	0.98634
Medication	Sulfonamides and trimethoprim	T		378957	595	378506	0.971	-0.02927	0.212914	0.64	1.47	0.890641	0.98634
Medication	Hormone antagonists and related agents	T		378957	2999	376102	1.01	0.01291	0.093154	0.844	1.22	0.889776	0.98634
Medication	Antigout preparations	T		378957	4574	374527	0.989	-0.01081	0.077135	0.85	1.15	0.888523	0.98634
Medication	Chapter p Chapter X Diseases of the respiratory system BIN (HES)	T		378957	9049	370052	0.993	-0.00698	0.054877	0.892	1.11	0.898762	0.98634
Operations and Procedures	Umbilical hernia repair (self-reported)	T		378957	3357	375744	0.988	-0.01256	0.089279	0.829	1.18	0.88811	0.98634
Operations and Procedures	Anal surgery (self-reported)	T		378957	2357	376744	1.01	0.013654	0.105125	0.825	1.25	0.896657	0.98634
Operations and Procedures	Laparotomy nos (self-reported)	T		378957	2106	376995	1.01	0.013537	0.111117	0.815	1.26	0.903035	0.98634
Operations and Procedures	Inguinal hernia repair (self-reported)	T		378957	5531	373570	1.01	0.009895	0.068965	0.882	1.16	0.885916	0.98634
Operations and Procedures	Orchidopexy (self-reported)	T		378957	293	378808	0.962	-0.03837	0.307742	0.526	1.76	0.900786	0.98634
Family history	Parkinson s disease (family history - sibling)	T		301109	1576	299660	1.02	0.018105	0.127286	0.793	1.31	0.886892	0.98634
Mental health	Bipolar I Disorder	T		91467	595	90920	0.97	-0.03087	0.213524	0.638	1.47	0.885039	0.98634
Cancer	Neo of urinary organs (cancer register)	T		378957	723	378378	1.03	0.027067	0.186893	0.712	1.48	0.884847	0.98634
Summary	Mal neo of thyroid and other endocrine glands (cancer register)	T		378957	468	378633	0.967	-0.03304	0.240613	0.604	1.55	0.890767	0.98634
Operations and Procedures	Other operations on lacrimal apparatus	T		378957	939	378162	0.98	-0.02071	0.168074	0.705	1.36	0.901942	0.98634
Operations and Procedures	Incision of sclera	T		378957	216	378885	1.05	0.044599	0.34082	0.536	2.04	0.895887	0.98634
Operations and Procedures	Microtherapeutic endoscopic operations on larynx	T		378957	579	378522	0.972	-0.02851	0.21762	0.634	1.49	0.895756	0.98634
Operations and Procedures	Diagnostic endoscopic examination of larynx	T		378957	3294	375807	0.988	-0.01206	0.090625	0.827	1.18	0.894104	0.98634
Operations and Procedures	Surgical removal of tooth	T		378957	5408	373693	0.991	-0.0087	0.07063	0.863	1.14	0.901937	0.98634
Operations and Procedures	Emergency excision of appendix	T		378957	2570	376531	1.01	0.012559	0.100971	0.831	1.23	0.901013	0.98634
Operations and Procedures	Other excision of colon	T		378957	242	378859	1.04	0.043561	0.323208	0.554	1.97	0.892789	0.98634
Operations and Procedures	Other operations on bowel	T		378957	379	378722	0.967	-0.03393	0.272561	0.567	1.65	0.900917	0.98634
Operations and Procedures	Other therapeutic endoscopic operations on bladder	T		378957	557	378544	1.03	0.029608	0.213329	0.678	1.56	0.889616	0.98634
Operations and Procedures	Photodestruction of lesion of skin	T		378957	418	378683	1.03	0.032169	0.247907	0.635	1.68	0.896755	0.98634
Operations and Procedures	Transposition of tendon	T		378957	795	378306	0.976	-0.02415	0.183468	0.681	1.4	0.895276	0.98634
Operations and Procedures	Other operations on muscle	T		378957	238	378863	0.958	-0.04261	0.340027	0.492	1.87	0.900277	0.98634
Operations and Procedures	Bone and extramedullary fixation	T		378957	5789	373312	1.01	0.008202	0.067581	0.883	1.15	0.903401	0.98634
Operations and Procedures	Stabilising operations on joint	T		378957	716	378385	0.975	-0.02489	0.193041	0.668	1.42	0.897407	0.98634
Operations and Procedures	Therapeutic endoscopic operations on other articular cartilage	T		378957	3095	376006	1.01	0.011603	0.091395	0.846	1.21	0.898978	0.98634
Eye	Glaucoma	T		124323	5306	119070	1.01	0.010216	0.070752	0.879	1.16	0.885194	0.98634
ENT	Bleeding gums	T		377502	49304	328342	0.996	-0.0036	0.024986	0.949	1.05	0.885353	0.98634
Neurosciences	Facial pain	T		378087	12842	365389	1.01	0.005903	0.046049	0.919	1.1	0.898004	0.98634
Respiratory	Asthma (HES and self-reported)	T		378957	48549	330552	1	0.003097	0.024888	0.955	1.05	0.900984	0.98634
Respiratory	Pneumonia (HES and self-reported)	T		378957	13446	365655	1.01	0.005412	0.044893	0.921	1.1	0.904043	0.98634
ENT	Nasal polyps (HES and self-reported)	T		378957	4016	375085	0.989	-0.01079	0.081963	0.842	1.16	0.895218	0.98634
Musculoskeletal	Fracture toe (HES and self-reported)	T		378957	413	378688	1.03	0.031159	0.24793	0.635	1.68	0.899987	0.98634
Medication	Other analgesics and antipyretics	T		378957	121055	258046	0.998	-0.00214	0.017983	0.963	1.03	0.905121	0.986954
Cancer	Diffuse non Hodgkin s lymphoma (cancer register)	T		378957	730	378371	1.02	0.021996	0.186758	0.709	1.47	0.906244	0.986954
Operations and Procedures	Other operations on sympathetic nerve	T		378957	470	378631	0.972	-0.02835	0.240575	0.607	1.56	0.906192	0.986954
Anthropometry	Appendicular lean mass	T		4100	NA	NA	NA	0.010145	0.086122	-0.15865	0.178944	0.906224	0.986954
Immuno-inflammation	Pruritus (HES)	T		378957	705	378396	1.02	0.022495	0.193109	0.7	1.49	0.907267	0.987621
Medication	Ascorbic acid vitamin c incl combinations	T		378957	6192	372909	0.993	-0.00728	0.066156	0.872	1.13	0.912368	0.987823
Medication	Antiadrenergic agents centrally acting	T		378957	1115	377986	1.02	0.016557	0.152513	0.754	1.37	0.91355	0.987823
Eye	Visual disturbances (HES)	T		378957	2695	376406	0.989	-0.01072	0.099497	0.814	1.2	0.914237	0.987823
Respiratory	Peritonsillar abscess (HES)	T		378957	376	378725	0.97	-0.03074	0.272661	0.568	1.65	0.910245	0.987823
Respiratory	Other respiratory disorders (HES)	T		378957	2907	376194	1.01	0.010178	0.094872	0.839	1.22	0.914565	0.987823
Digestive system	Duodenal ulcer (HES)	T		378957	2818	376283	0.989	-0.01112	0.098216	0.816	1.2	0.909825	0.987823
Digestive system	Diaphragmatic hernia (HES)	T		378957	24453	354648	1	0.003891	0.033985	0.939	1.07	0.908837	0.987823

Musculoskeletal	Pyogenic arthritis (HES)	T	378957	335	378766	0.97	-0.03028	0.28314	0.557	1.69	0.914833	0.987823
Medication	Expectorants excl combinations with cough suppressants	T	378957	1117	377984	0.983	-0.01719	0.155941	0.724	1.33	0.912207	0.987823
Gynaecology and Obstetrics	Hysterectomy with oophorectomy (self-reported)	T	378957	668	378433	1.02	0.021349	0.196978	0.694	1.5	0.913691	0.987823
Gynaecology and Obstetrics	Intrauterine contraceptive device insertion removal (self-reported)	T	378957	624	378477	0.978	-0.0226	0.204647	0.655	1.46	0.912074	0.987823
Operations and Procedures	Excision of cervix uteri	T	378957	3167	375934	0.99	-0.01002	0.091673	0.827	1.18	0.912927	0.987823
Operations and Procedures	Reduction of fracture of other bone of face	T	378957	686	378415	1.02	0.021042	0.196672	0.695	1.5	0.914796	0.987823
Operations and Procedures	Other destruction of organ NOC	T	378957	2176	376925	0.988	-0.01187	0.110415	0.796	1.23	0.914363	0.987823
Immuno-inflammation	Allergy or anaphylactic reaction to food (HES and self-reported)	T	378957	1833	377268	1.01	0.012876	0.11903	0.802	1.28	0.91386	0.987823
Gynaecology and Obstetrics	Female infertility (HES and self-reported)	T	378957	1328	377773	1.02	0.015452	0.140173	0.772	1.34	0.912225	0.987823
Cardiovascular	Hypertrophic cardiomyopathy hcm hocm (HES and self-reported)	T	378957	265	378836	1.04	0.034847	0.308225	0.566	1.89	0.909985	0.987823
Cardiovascular	P duration	T	8938	NA	NA	NA	0.006255	0.057058	-0.10558	0.118089	0.912705	0.987823
Gynaecology and Obstetrics	Multiple gestation (HES)	T	378957	258	378843	1.03	0.032816	0.309643	0.563	1.9	0.915599	0.987982
Summary	Benign neo (cancer register)	T	378957	784	378317	0.981	-0.01931	0.183531	0.685	1.41	0.916211	0.987982
Neurosciences	Pain all over the body	T	378087	5847	372384	0.993	-0.00717	0.067985	0.869	1.13	0.915994	0.987982
Symptoms	Rash and other nonspecific skin eruption (HES)	T	378957	1635	377466	0.987	-0.01333	0.12785	0.768	1.27	0.916948	0.988335
Operations and Procedures	Harvest of flap of skin and fascia	T	378957	546	378555	1.02	0.022555	0.217862	0.667	1.57	0.917542	0.988533
Respiratory	Status asthmaticus (HES)	T	378957	233	378868	0.966	-0.03442	0.340185	0.496	1.88	0.919402	0.988551
Gynaecology and Obstetrics	Coronary angiogram (self-reported)	T	378957	2517	376584	1.01	0.010367	0.10217	0.827	1.23	0.919179	0.988551
Cancer	Mal neo cervix uteri (cancer register)	T	378957	384	378717	0.973	-0.02715	0.263748	0.58	1.63	0.91802	0.988551
Gynaecology and Obstetrics	Cervical erosion (HES and self-reported)	T	378957	898	378203	0.983	-0.01723	0.170753	0.703	1.37	0.919611	0.988551
Neurosciences	Number of incorrect matches in round	T	93547	NA	NA	NA	0.00167	0.016368	-0.03041	0.033751	0.918729	0.988551
Medication	Antihypertensives and diuretics in combination	T	378957	1142	377959	1.01	0.014457	0.150846	0.755	1.36	0.923646	0.988791
Summary	Polyneuropathies and peripheral nervous system (HES)	T	378957	1669	377432	0.989	-0.01104	0.125976	0.773	1.27	0.930196	0.988791
Summary	Other disorders of ear (HES)	T	378957	4112	374989	0.992	-0.0079	0.080459	0.847	1.16	0.92179	0.988791
Summary	Chondropathies (HES)	T	378957	1094	378007	0.985	-0.01472	0.155856	0.726	1.34	0.924745	0.988791
ENT	Other disorders of external ear (HES)	T	378957	883	378218	0.985	-0.01504	0.172769	0.702	1.38	0.930643	0.988791
Immuno-inflammation	Pilonidal cyst (HES)	T	378957	452	378649	0.978	-0.0225	0.247574	0.602	1.59	0.927597	0.988791
Musculoskeletal	Osteoporosis with pathological fracture (HES)	T	378957	834	378267	1.02	0.016587	0.17546	0.721	1.43	0.924686	0.988791
Genitourinary	Obstructive and reflux uropathy (HES)	T	378957	2412	376689	0.991	-0.00933	0.105106	0.806	1.22	0.929301	0.988791
Gynaecology and Obstetrics	Excessive vomiting in pregnancy (HES)	T	378957	202	378899	0.965	-0.03521	0.362019	0.475	1.96	0.922523	0.988791
Operations and Procedures	Testicular scrotal operation (self-reported)	T	378957	2430	376671	0.99	-0.0104	0.10602	0.804	1.22	0.921888	0.988791
Cancer	Follicular (cancer register)	T	378957	389	378712	1.02	0.024253	0.255577	0.621	1.69	0.924399	0.988791
Cancer	Carc in situ of breast (cancer register)	T	378957	1900	377201	1.01	0.010787	0.11699	0.804	1.27	0.926539	0.988791
Operations and Procedures	Diagnostic retrograde exam pancreatic duct	T	378957	208	378893	0.969	-0.03109	0.360809	0.478	1.97	0.931327	0.988791
Operations and Procedures	Diagnostic imaging of heart	T	378957	1244	377857	0.987	-0.01315	0.144707	0.743	1.31	0.927608	0.988791
Operations and Procedures	Diagnostic imaging of hepatobiliary system	T	378957	861	378240	0.984	-0.01593	0.175274	0.698	1.39	0.927598	0.988791
Operations and Procedures	Skeletal traction of bone	T	378957	210	378891	0.967	-0.03379	0.360703	0.477	1.96	0.925364	0.988791
Operations and Procedures	Other external fixation of bone	T	378957	667	378434	0.983	-0.01747	0.200302	0.664	1.46	0.930491	0.988791
Operations and Procedures	Approach to spine through back	T	378957	309	378792	1.03	0.025402	0.283615	0.588	1.79	0.928632	0.988791
Medication	Vitamin E	T	376892	11056	365980	1	0.004685	0.049357	0.912	1.11	0.924371	0.988791
ENT	Otosclerosis (HES and self-reported)	T	378957	416	378685	1.02	0.023399	0.247859	0.63	1.66	0.924789	0.988791
Infectious disease	Malaria (HES and self-reported)	T	378957	480	378621	0.979	-0.02129	0.234334	0.618	1.55	0.927618	0.988791
Digestive system	Malabsorptioncoeliac disease (HES and self-reported)	T	378957	2523	376578	0.991	-0.00931	0.102448	0.81	1.21	0.927619	0.988791
Infectious disease	Shingles (HES and self-reported)	T	378957	734	378367	0.983	-0.017	0.189762	0.678	1.43	0.928597	0.988791
Digestive system	Bowel intestinal obstruction (HES and self-reported)	T	378957	3937	375164	0.993	-0.0071	0.082213	0.845	1.17	0.931205	0.988791
Neurosciences	Benign neuroma (HES and self-reported)	T	378957	590	378511	1.02	0.019567	0.208652	0.677	1.53	0.925285	0.988791
Musculoskeletal	Fracture head neck (HES and self-reported)	T	378957	2134	376967	1.01	0.009965	0.111732	0.811	1.26	0.928937	0.988791
Musculoskeletal	OA under age 50	T	326966	14899	312194	1	0.003707	0.042807	0.923	1.09	0.930991	0.988791
Cardiovascular	Cardiac index	T	3764	NA	NA	NA	0.00846	0.089808	-0.16756	0.184485	0.924949	0.988791
Eye	Other retinal disorders (HES)	T	378957	3106	375995	0.992	-0.00777	0.091915	0.829	1.19	0.932615	0.989069
Eye	Current eye infection	T	85144	898	84281	0.986	-0.01442	0.171101	0.705	1.38	0.932821	0.989069
Eye	Blepharitis eyelid infection (HES and self-reported)	T	378957	653	378448	0.983	-0.01692	0.200416	0.664	1.46	0.932738	0.989069
Summary	Atrophies affecting central nervous system (HES)	T	378957	368	378733	1.02	0.022086	0.263882	0.609	1.71	0.933299	0.989141
Eye	Glaucoma surgery trabeculectomy (self-reported)	T	378957	535	378566	1.02	0.017795	0.218035	0.664	1.56	0.934953	0.989625
Other	Chest pain or discomfort when walking uphill or hurrying	T	47239	12507	34751	0.996	-0.00449	0.055227	0.893	1.11	0.935145	0.989625
Operations and Procedures	Extirpation of lesion of external ear	T	378957	1684	377417	0.99	-0.01011	0.125176	0.775	1.27	0.935616	0.989625
Operations and Procedures	Operations on aneurysm of cerebral artery	T	378957	201	378900	1.03	0.029802	0.361012	0.508	2.09	0.934209	0.989625
Operations and Procedures	Other autograft of bone	T	378957	1078	378023	0.987	-0.0127	0.157661	0.725	1.34	0.93581	0.989625
Genitourinary	Undescended testicle (HES and self-reported)	T	378957	210	378891	0.972	-0.02877	0.360858	0.479	1.97	0.936455	0.989873
Digestive system	Gingivitis and periodontal diseases (HES)	T	378957	766	378335	0.986	-0.0144	0.186536	0.684	1.42	0.93845	0.991547
Medication	Angiotensin ii antagonists plain	T	378957	16106	362995	0.997	-0.00277	0.041485	0.919	1.08	0.946694	0.99158
Summary	Inflammatory diseases of the central nervous system (HES)	T	378957	536	378565	0.985	-0.01555	0.222849	0.636	1.52	0.944364	0.99158
Summary	HESCH Diseases of the respiratory system BIN (HES)	T	378957	55412	323689	1	0.001763	0.023668	0.956	1.05	0.940612	0.99158
Infectious disease	Diarrhoea presumed infectious (HES)	T	378957	5076	374025	0.995	-0.0048	0.072517	0.863	1.15	0.947254	0.99158
Metabolic	Postprocedural endocrine and metabolic disorders (HES)	T	378957	1206	377895	0.99	-0.00959	0.147684	0.742	1.32	0.948205	0.99158
Respiratory	Respiratory failure (HES)	T	378957	1845	377256	0.992	-0.00837	0.119927	0.784	1.25	0.94439	0.99158
Symptoms	Abdominal and pelvic pain (HES)	T	378957	28298	350803	1	0.002318	0.031773	0.942	1.07	0.941855	0.99158
Medication	Direct acting antivirals	T	378957	823	378278	1.01	0.01333	0.177972	0.715	1.44	0.940296	0.99158
Medication	Antiinfectives	T	378957	4005	375096	0.994	-0.0059	0.081884	0.847	1.17	0.942565	0.99158
Operations and Procedures	Elbow surgery (self-reported)	T	378957	2248	376853	1.01	0.007196	0.107972	0.815	1.24	0.946863	0.99158
Operations and Procedures	Bunion hallus valgus surgery (self-reported)	T	378957	4996	374105	1.01	0.005282	0.072716	0.872	1.16	0.942097	0.99158
Cancer	Mal neo larynx (cancer register)	T	378957	224	378877	1.02	0.023292	0.340653	0.525	2	0.945489	0.99158
Cancer	Mal neo corpus uteri (cancer register)	T	378957	1276	377825	1.01	0.009441	0.142262	0.764	1.33	0.947091	0.99158
Operations and Procedures	Operations on duct of breast	T	378957	761	378340	0.986	-0.01417	0.1867	0.684	1.42	0.939497	0.99158
Operations and Procedures	Extirpation of lesion of lip	T	378957	1563	377538	0.992	-0.00841	0.130941	0.767	1.28	0.948772	0.99158
Operations and Procedures	Primary closed reduction of traumatic dislocation of joint	T	378957	775	378326	0.987	-0.01275	0.186471	0.685	1.42	0.945468	0.99158
Operations and Procedures	Other operations on unspecified organ	T	378957	2194	376907	1.01	0.007561	0.115471	0.804	1.26	0.947791	0.99158
Neurosciences	Peripheral neuropathy (HES and self-reported)	T	378957	2013	377088	1.01	0.007683	0.113824	0.806	1.26	0.946187	0.99158
Cardiovascular	Lymphoedema (HES and self-reported)	T	378957	527	378574	0.984	-0.01615	0.222971	0.636	1.52	0.942259	0.99158
Respiratory	Respiratory infection (HES and self-reported)	T	378957	9656	369445	0.996	-0.00378	0.053085	0.898	1.11	0.943265	0.99158
Musculoskeletal	Tennis elbow lateral epicondylitis (HES and self-reported)	T	378957	796	378305	1.01	0.012318	0.180717	0.71	1.44	0.945656	0.99158
Musculoskeletal	Housemaids knee prepatellar bursitis (HES and self-reported)	T	378957	300	378801	1.02	0.019566	0.294881	0.572	1.82	0.947096	0.99158
Infectious disease	Scarlet fever scarlatina (HES and self-reported)	T	378957	435	378666	1.02	0.016838	0.241013	0.634	1.63	0.944303	0.99158
Anthropometry	Total thigh muscle volume	T	758	NA	NA	NA	-0.01302	0.202669	-0.41026	0.384206	0.948758	0.99158
Biological assays	Eosinophill count	T	367067	NA	NA	NA	-0.00064	0.008442	-0.01719	0.015906	0.939498	0.99158
Anthropometry	Total trunk fat	T	801	NA	NA	NA	0.012628	0.19988	-0.37914	0.404393	0.949626	0.992042
Medication	Corticosteroids combinations with antibiotics	T	378957	20857	358244	0.998	-0.0022	0.036439	0.929	1.07	0.9519	0.992276
Summary	Diseases of inner ear (HES)	T	378957	1848	377253	1.01	0.007106	0.119001	0.798	1.27	0.952385	0.992276
Musculoskeletal	Other soft tissue disorders (HES)	T	378957	11219	367882	0.997	-0.00292	0.049217	0.905	1.1	0.952623	0.992276
Gynaecology and Obstetrics	Uterine polypectomy uterine polyps removed (self-reported)	T	378957	529	378572	1.01	0.013562	0.218214	0.661	1.55	0.950443	0.992276
Neurosciences	General pain for 3 months vs no pain	T	377059	4819	372384	1	0.004417	0.074371	0.868	1.16	0.952642	0.992276
Operations and Procedures	Other operations on vagina	T	378957	544	378557	1.01	0.012923	0.218026	0.661	1.55	0.952733	0.992276
Operations and Procedures	External beam radiotherapy	T	378957	1376	377725	1.01	0.008352	0.135768	0.773	1.32	0.950947	0.992276
Summary	Haemolytic anaemias (HES)	T	378957	276	378825	1.02	0.017747	0.308009	0.557	1.86	0.954052	0.992791
Cancer	Mal neo other connect soft tissue (cancer register)	T	378957	247	378854	1.02	0.018777	0.323058	0.541	1.92	0.953652	0.992791
Operations and Procedures	Endoscopy gastroscopy (self-reported)	T	378957	7551	371550	0.997	-0.00331	0.059884	0.886	1.12	0.955905	0.99429
Medication	Antiinf and antiseptics excl comb with cortico	T	378957	8523	370578	1	0.002648	0.056327	0.898	1.12	0.9625	0.994359
Summary	Other diseases of the respiratory system (HES)	T	378957	4856	374245	1	0.003297	0.073937	0.868	1.16	0.96443	0.994359
Summary	Abnormal findings of urine without diagnosis (HES)	T	378957	652	378449	1.01	0.010688	0.2004	0.682	1.5	0.957467	0.994359
Respiratory	Unspecified acute lower respiratory infection (HES)	T	378957	6878	372223	1	0.002737	0.062457	0.887	1.13	0.965052	0.994359
Symptoms	Shock (HES)	T	378957	252	378849	0.985	-0.01478	0.322948	0.523	1.86	0.963489	0.994359
Medication	Magnetic resonance imaging contrast media	T	378957	1522	377579	0.994	-0.00582	0.132108	0.767	1.29	0.964851	0.994359
Operations and Procedures	Urethral surgery (self-reported)	T	378957	686	378415	0.99	-0.00986	0.196611	0.674	1.46	0.960001	0.994359
Operations and Procedures	Pilonidal sinus surgery anal (self-reported)	T	378957	2174	376927	1.01	0.005972	0.110476	0.81	1.25	0.95689	0.994359
Cardiovascular	Echocardiogram transoesophageal echo (self-reported)	T	378957	455	378646	1.01	0.010594	0.240795	0.63	1.62	0.964907	0.994359
Family history	Severe depression (family history - sibling)	T	301109	21287	279949	1	0.001967	0.036446	0.933	1.08	0.956951	0.994359
Operations and Procedures	Repair of obstetric laceration	T	378957	3617	375484	0.996	-0.00418	0.088757	0.837	1.19	0.962419	0.994359
Operations and Procedures	Rehabilitation for other disorders	T	378957	724	378377	0.992	-0.00833	0.189858	0.684	1.44	0.965008	0.994359
Operations and Procedures	High cost neurology drugs	T	378957	1209	377892	0.993	-0.00694	0.147589	0.744	1.33	0.962502	0.994359
Operations and Procedures	High cost ophthalmology drugs	T	378957	346	378755	1.01	0.013399	0.273177	0.593	1.73	0.96088	0.994359
ENT	Painful gums	T	377502	10716	366930	1	0.002375	0.050212	0.908	1.11	0.962276	0.994359
Medication	Iron	T	377706	11432	366418	1	0.00239	0.048689	0.911	1.1	0.960845	0.994359
Neurosciences	Psychologicalpsychiatric prob (HES and self-reported)	T	378957	2225	376876	1	0.004828	0.109137	0.811	1.24	0.964712	0.994359
Cardiovascular	Sick sinus syndrome (HES and self-reported)	T	378957	373	378728	0.988	-0.01198	0.263854	0.589	1.66	0.963799	0.994359
Genitourinary	Cystitis (HES and self-reported)	T	378957	2918	376183	0.995	-0.00506	0.095627	0.825	1.2	0.957763	0.994359
Musculoskeletal	Fracture foot (HES and self-reported)	T	378957	1193	377908	0.993	-0.00669	0.14911	0.742	1.33	0.964196	0.994359
Anthropometry	Total lean mass	T	4100	NA	NA	NA	0.003895	0.086175	-0.16501	0.172797	0.963952	0.994359

Eye	Corneal hysteresis right	T	81141	NA	NA	NA	-0.00082	0.017724	-0.03556	0.03392	0.963124	0.994359
Summary	Papulosquamous disorders (HES)	T	378957	2604	376497	0.996	-0.00428	0.10141	0.816	1.21	0.966351	0.994377
Medication	Aminoglycoside antibacterials	T	378957	622	378479	1.01	0.008528	0.204404	0.676	1.51	0.966721	0.994377
Medication	Antiinfectives	T	378957	1041	378060	0.993	-0.00684	0.159626	0.726	1.36	0.965818	0.994377
Eye	Eyelid surgery (self-reported)	T	378957	269	378832	1.01	0.012972	0.308099	0.554	1.85	0.966416	0.994377
Operations and Procedures	Excision of rectum	T	378957	2290	376811	1	0.004267	0.106882	0.814	1.24	0.968155	0.995427
Summary	HESCH Diseases of the ear and mastoid process (HES)	T	378957	8809	370292	0.998	-0.00202	0.055265	0.896	1.11	0.97078	0.995576
Respiratory	Other disorders of nose and nasal sinuses (HES)	T	378957	5991	373110	1	0.002467	0.067155	0.879	1.14	0.970692	0.995576
Symptoms	Other general symptoms and signs (HES)	T	378957	358	378743	0.99	-0.01024	0.272887	0.58	1.69	0.970071	0.995576
Operations and Procedures	Breath tests	T	378957	412	378689	0.99	-0.00967	0.255248	0.601	1.63	0.969793	0.995576
Operations and Procedures	Harvest of bone	T	378957	1453	377648	1	0.004922	0.134314	0.772	1.31	0.970767	0.995576
Gynaecology and Obstetrics	Cervical intraepithelial neoplasia cin precan (HES and self-reported)	T	378957	1670	377431	0.995	-0.00476	0.125368	0.778	1.27	0.969696	0.995576
Symptoms	Other abnormal findings in urine (HES)	T	378957	297	378804	1.01	0.010076	0.294931	0.567	1.8	0.972747	0.996745
ENT	Nasal polyp surgery nasal polypectomy (self-reported)	T	378957	1137	377964	1.01	0.005179	0.150877	0.748	1.35	0.972617	0.996745
Operations and Procedures	Vasectomy (self-reported)	T	378957	9426	369675	1	0.001711	0.054668	0.9	1.12	0.97503	0.997565
Operations and Procedures	Other diagnostic imaging of vascular system	T	378957	2957	376144	0.997	-0.00296	0.094059	0.829	1.2	0.974927	0.997565
Operations and Procedures	Diaphyseal division of bone	T	378957	396	378705	0.992	-0.00809	0.255565	0.601	1.64	0.974749	0.997565
Operations and Procedures	Other intravenous injection	T	378957	5047	374054	0.998	-0.00226	0.072731	0.865	1.15	0.975204	0.997565
Gynaecology and Obstetrics	Maternal care for related to pregnancy (HES)	T	378957	1641	377460	1	0.003788	0.128065	0.781	1.29	0.976404	0.998104
Symptoms	Gangrene (HES)	T	378957	510	378591	0.993	-0.00662	0.228402	0.635	1.55	0.97686	0.998104
Operations and Procedures	Drainage through perineal region	T	378957	1052	378049	1	0.004607	0.159648	0.735	1.37	0.976978	0.998104
Digestive system	Hiatus hernia (HES and self-reported)	T	378957	27678	351423	1	0.000911	0.032146	0.94	1.07	0.977388	0.998104
Operations and Procedures	Other excision of skin	T	378957	205	378896	1.01	0.009782	0.360872	0.498	2.05	0.978375	0.998689
Medication	Intestinal antiinflammatory agents	T	378957	25513	353588	1	0.00072	0.033174	0.938	1.07	0.982676	0.999046
Medication	I v solution additives	T	378957	936	378165	0.997	-0.00309	0.168112	0.717	1.39	0.985322	0.999046
Medication	Cardiac glycosides	T	378957	1042	378059	0.998	-0.00194	0.158089	0.732	1.36	0.990194	0.999046
Medication	Corticosteroids plain	T	378957	34978	344123	1	0.000432	0.02875	0.946	1.06	0.988015	0.999046
Summary	Disorders of choroid and retina (HES)	T	378957	6755	372346	1	-0.0005	0.062572	0.884	1.13	0.993655	0.999046
Summary	Diseases of appendix (HES)	T	378957	3245	375856	0.999	-0.00066	0.090279	0.837	1.19	0.994192	0.999046
Summary	HESCH Congen malfor deform and chrom abnormalities (HES)	T	378957	4981	374120	0.999	-0.00076	0.073033	0.866	1.15	0.991672	0.999046
Mental health	Bipolar affective disorder (HES)	T	378957	888	378213	0.998	-0.00175	0.172729	0.712	1.4	0.991899	0.999046
Neurosciences	Other headache syndromes (HES)	T	378957	459	378642	0.996	-0.0044	0.240677	0.621	1.6	0.985423	0.999046
Neurosciences	Other diseases of spinal cord (HES)	T	378957	528	378573	1.01	0.005799	0.222933	0.65	1.56	0.979248	0.999046
Neurosciences	Other disorders of CNS (HES)	T	378957	223	378878	1.01	0.006038	0.340478	0.516	1.96	0.985852	0.999046
Respiratory	Other diseases of upper respiratory tract (HES)	T	378957	650	378451	1	0.002444	0.20042	0.677	1.48	0.990269	0.999046
Operations and Procedures	Incisional hernia repair (self-reported)	T	378957	1461	377640	0.997	-0.0026	0.134342	0.767	1.3	0.984569	0.999046
Operations and Procedures	Shoulder surgery (self-reported)	T	378957	5679	373422	0.999	-0.0011	0.068317	0.874	1.14	0.987117	0.999046
Gynaecology and Obstetrics	Pelvic floor surgery (self-reported)	T	378957	271	378830	1.01	0.007805	0.308189	0.551	1.84	0.979795	0.999046
Operations and Procedures	Removal of malignant melanoma (self-reported)	T	378957	716	378385	0.998	-0.00161	0.189868	0.688	1.45	0.993229	0.999046
Cancer	Mal neo thyroid gland (cancer register)	T	378957	431	378670	0.996	-0.00387	0.247711	0.613	1.62	0.987523	0.999046
Cancer	Benign neoplasm of meninges (cancer register)	T	378957	350	378751	0.994	-0.00614	0.273033	0.582	1.7	0.982054	0.999046
Operations and Procedures	Excision of parathyroid gland	T	378957	695	378406	0.996	-0.00433	0.193221	0.682	1.45	0.982123	0.999046
Operations and Procedures	Drainage of middle ear	T	378957	1751	377350	0.998	-0.00221	0.123162	0.784	1.27	0.985709	0.999046
Operations and Procedures	Operations on unspecified nasal sinus	T	378957	717	378384	1	0.002253	0.193043	0.687	1.46	0.990689	0.999046
Operations and Procedures	Surgery on apex of tooth	T	378957	1147	377954	1	0.003271	0.152392	0.744	1.35	0.982877	0.999046
Operations and Procedures	Extirpation of lesion of tongue	T	378957	690	378411	0.996	-0.00385	0.196577	0.678	1.46	0.984389	0.999046
Operations and Procedures	Other operations on scrotum	T	378957	203	378898	1.01	0.007423	0.361088	0.496	2.04	0.983599	0.999046
Operations and Procedures	Biopsy of cervix uteri	T	378957	1749	377352	1	0.001411	0.122625	0.787	1.27	0.990822	0.999046
Operations and Procedures	Other open operations on bone	T	378957	605	378496	1	0.002241	0.208544	0.666	1.51	0.991427	0.999046
Operations and Procedures	Harvest of other tissue	T	378957	784	378317	1	0.004346	0.183525	0.701	1.44	0.981108	0.999046
Medication	Vitamin A	T	376892	7193	369843	1	0.000422	0.060985	0.888	1.13	0.994484	0.999046
Medication	Selenium	T	377706	9070	368780	1	0.000621	0.054299	0.9	1.11	0.990871	0.999046
Cardiovascular	Subdural haemorrhagehaematoma (HES and self-reported)	T	378957	380	378721	0.997	-0.00258	0.263736	0.595	1.67	0.992195	0.999046
Metabolic	Hypothyroidismmyxoedema (HES and self-reported)	T	378957	22541	356560	1	0.000279	0.035547	0.933	1.07	0.993744	0.999046
Eye	Eye infection (HES and self-reported)	T	378957	2800	376301	1	0.001864	0.097243	0.828	1.21	0.984711	0.999046
Immuno-inflammation	Allergyhypersensitivityanaph (HES and self-reported)	T	378957	3101	376000	0.998	-0.0015	0.09281	0.832	1.2	0.98709	0.999046
Immuno-inflammation	Hayfeverallergic rhinitis (HES and self-reported)	T	378957	22257	356844	1	-0.00032	0.035386	0.933	1.07	0.992811	0.999046
Musculoskeletal	Burns (HES and self-reported)	T	378957	765	378336	1	0.001869	0.186544	0.695	1.44	0.992008	0.999046
Immuno-inflammation	Chronic fatigue syndrome (HES and self-reported)	T	378957	1921	377180	0.997	-0.00273	0.11744	0.792	1.26	0.98146	0.999046
Infectious disease	Infectious mononucleosis glandular fever epste (HES and self-reported)	T	378957	517	378584	0.996	-0.00366	0.223039	0.644	1.54	0.98691	0.999046
Musculoskeletal	Fracture nose (HES and self-reported)	T	378957	1043	378058	0.997	-0.00259	0.159672	0.729	1.36	0.987038	0.999046
Summary	Other maternal disorders predominantly related to pregnancy (HES)	T	378957	2789	376312	1	-0.00038	0.100212	0.821	1.22	0.996992	0.999181
Summary	Complications predominantly related to the puerperium (HES)	T	378957	557	378544	1	0.000905	0.21503	0.657	1.53	0.996643	0.999181
Immuno-inflammation	Seborrhoeic keratosis (HES)	T	378957	2813	376288	1	-0.00031	0.096898	0.827	1.21	0.997472	0.999181
Genitourinary	Calculus of lower urinary tract (HES)	T	378957	726	378375	1	-0.00039	0.189963	0.689	1.45	0.998351	0.999181
Family history	Parkinson s disease (family history - mother)	T	354043	5725	348456	1	-0.0002	0.067791	0.875	1.14	0.997615	0.999181
Operations and Procedures	Other internal fixation of bone	T	378957	5999	373102	1	0.000225	0.066589	0.878	1.14	0.997307	0.999181
Operations and Procedures	Diagnostic endoscopic examination of other joint	T	378957	560	378541	0.999	-0.00067	0.217764	0.652	1.53	0.997554	0.999181
Operations and Procedures	Other methods of operation on organ NOC	T	378957	1830	377271	1	-0.00038	0.120615	0.789	1.27	0.997494	0.999181
Musculoskeletal	BIN combinedFractures	T	378957	28020	351081	1	-7.67E-05	0.031812	0.939	1.06	0.998076	0.999181
Musculoskeletal	Fracture pelvis (HES and self-reported)	T	378957	555	378546	1	0.0003	0.217799	0.653	1.53	0.998903	0.999318
Summary	Demyelinating diseases of the central nervous system (HES)	T	378957	1406	377695	1	2.26E-05	0.136707	0.765	1.31	0.999868	0.999868

Ordered by FDR. The category "Summary" indicates cases were anyone with admission for any code in the block. * Lung function measures described as "strict" only include individuals with passed-QC spirometry (see reference 16 in the main manuscript). Strict measures were not included in Figure 1.
