# Supplementary material for: Pleiotropic associations of heterozygosity for the SERPINA1 Z allele in the UK Biobank
Source: ERJ Open Res. 2021 May 10;7(2):00049-2021. doi: 10.1183/23120541.00049-2021 (PMC8107350; doi:10.1183/23120541.00049-2021)
Supplement: Supplementary file 4 [file 00049-2021.TableS3.pdf]

Table S3. Phenome-wide association study results for *SERPINA1* Z allele homozygosity in UK Biobank

Category	Phenotype	Effect allel	N	Cases	Controls	OR	Beta	SE	L95	U95	P	FDR
Respiratory	Alpha1 antitrypsin deficiency (HES and self-reported)	T	379101	223	378878	293	5.67848	0.260676	175	488	3.32E-105	7.90E-102
Metabolic	Other metabolic disorders (HES)	T	379101	309	378792	194	5.27007	0.262003	116	325	5.50E-90	6.55E-87
Respiratory	Emphysema (HES and self-reported)	T	379101	1662	377439	52.5	3.96133	0.240719	32.8	84.2	7.56E-61	6.00E-58
Respiratory	Emphysema (HES)	T	379101	1571	377530	52.5	3.96161	0.245029	32.5	84.9	8.49E-59	5.06E-56
Respiratory	Chronic obstructive airways disease (COPD) (HES and self-reported)	T	379101	8796	370305	13.3	2.59138	0.219958	8.67	20.5	4.87E-32	2.32E-29
Respiratory	Emphysemachronic bronchitis (HES and self-reported)	T	379101	6338	372763	13.9	2.62844	0.226642	8.88	21.6	4.25E-31	1.69E-28
Respiratory	COPDMappingStrict*	T	190073	16733	173340	13.2	2.57861	0.24978	8.08	21.5	5.52E-25	1.88E-22
Respiratory	COPDMappingLoose* primAndSec	T	244600	25872	218728	8.81	2.17549	0.221711	5.7	13.6	9.97E-23	2.97E-20
Respiratory	FEV1 FVC ratio ever smoked only	T	175269	NA	NA	NA	-1.1089	0.132374	-1.36835	-0.849447	5.43E-17	1.44E-14
Respiratory	FEV1 FVC ratio ever smoked only strict	T	136716	NA	NA	NA	-1.15924	0.148963	-1.45121	-0.867273	7.14E-15	1.70E-12
Summary	Chronic lower respiratory diseases (HES)	T	379101	30351	348750	4.36	1.47141	0.190988	3	6.33	1.32E-14	2.85E-12
Respiratory	FEV1 FVC ratio strict	T	221665	NA	NA	NA	-0.836435	0.111045	-1.05408	-0.618787	4.98E-14	9.88E-12
Respiratory	FEV1 FVC ratio	T	286167	NA	NA	NA	-0.706768	0.098487	-0.899803	-0.513733	7.17E-13	1.31E-10
Respiratory	Other chronic obstructive pulmonary disease (HES)	T	379101	7608	371493	7.23	1.97863	0.276163	4.21	12.4	7.79E-13	1.33E-10
Medication	Other drugs for obstructive airway diseases inhalants	T	379101	29711	349390	3.6	1.27976	0.199137	2.43	5.31	1.31E-10	2.07E-08
Family history	Chronic bronchitis emphysema (family history - sibling)	T	301029	8228	292801	5.67	1.73432	0.272396	3.32	9.66	1.93E-10	2.87E-08
Biological assays	Haemoglobin concentration	T	367863	NA	NA	NA	0.518894	0.084205	0.353852	0.683936	7.17E-10	1.00E-07
Respiratory	FEV1 percent pred	T	285936	NA	NA	NA	-0.599807	0.098453	-0.792774	-0.40684	1.11E-09	1.47E-07
Respiratory	FEV1 percent pred strict	T	221665	NA	NA	NA	-0.651361	0.11101	-0.868941	-0.433781	4.42E-09	5.54E-07
Summary	HESCH Diseases of the respiratory system BIN (HES)	T	379101	55412	323689	2.85	1.04713	0.180357	2	4.06	6.40E-09	7.62E-07
Medication	Adrenergics inhalants	T	379101	26289	352812	3.35	1.2098	0.211356	2.22	5.07	1.04E-08	1.18E-06
Respiratory	Other respiratory problems (HES and self-reported)	T	379101	5684	373417	5.63	1.72865	0.31691	3.03	10.5	4.91E-08	5.31E-06
Biological assays	Haematocrit percentage	T	367863	NA	NA	NA	0.455851	0.084203	0.290814	0.620888	6.17E-08	6.39E-06
Respiratory	Bring up phlegm sputum mucus on most days	T	95696	8125	87571	5.99	1.78964	0.337325	3.09	11.6	1.12E-07	1.11E-05
Respiratory	FEV1 ever smoked only	T	175269	NA	NA	NA	-0.700005	0.132125	-0.95897	-0.44104	1.17E-07	1.11E-05
Other	Wheeze or whistling in the chest in last year	T	371979	77179	294800	2.49	0.913788	0.174916	1.77	3.51	1.75E-07	1.60E-05
Respiratory	Bronchiectasis (HES and self-reported)	T	379101	2109	376992	8.57	2.14808	0.421477	3.75	19.6	3.46E-07	3.05E-05
Respiratory	PEF maximumValue	T	286167	NA	NA	NA	-0.494363	0.09839	-0.687207	-0.301519	5.05E-07	4.29E-05
Medication	Decongestants and other nasal preparations for topical use	T	379101	33775	345326	2.75	1.0132	0.205968	1.84	4.12	8.69E-07	7.13E-05
Operations and Procedures	Diagnostic imaging of chest	T	379101	2157	376944	7.82	2.05652	0.420494	3.43	17.8	1.00E-06	7.97E-05
Respiratory	PEF maximumValue strict	T	221665	NA	NA	NA	-0.532893	0.110972	-0.750398	-0.315388	1.57E-06	0.000121
Respiratory	Cough on most days	T	95696	13048	82648	4.65	1.53701	0.321706	2.48	8.74	1.77E-06	0.000132
Respiratory	Bronchiectasis (HES)	T	379101	1708	377393	8.78	2.17191	0.459648	3.56	21.6	2.30E-06	0.000166
Medication	Corticosteroids plain	T	379101	34978	344123	2.64	0.970822	0.205862	1.76	3.95	2.41E-06	0.000169
Respiratory	Respiratory disorders in diseases classified elsewhere (HES)	T	379101	201	378900	28.2	3.33759	0.717081	6.9	115	3.25E-06	0.000221
Respiratory	Pleurisy (HES and self-reported)	T	379101	1687	377414	8.21	2.10541	0.456218	3.36	20.1	3.93E-06	0.00026
Haematology	Other diseases of blood and blood forming organs (HES)	T	379101	563	378538	14.8	2.69252	0.586526	4.68	46.6	4.42E-06	0.000277
Eye	Optic neuritis (HES and self-reported)	T	379101	206	378895	26.9	3.29319	0.716627	6.61	110	4.32E-06	0.000277
Respiratory	FEV1 maximumValue	T	286167	NA	NA	NA	-0.449168	0.098291	-0.641819	-0.256517	4.88E-06	0.000298
Respiratory	FEV1 ever smoked only strict	T	136716	NA	NA	NA	-0.663217	0.148727	-0.954722	-0.371712	8.22E-06	0.000489
Operations and Procedures	Ureteric surgery (self-reported)	T	379101	604	378497	13.4	2.59432	0.58543	4.25	42.2	9.36E-06	0.000544
Medication	Adrenergics for systemic use	T	379101	22332	356769	2.83	1.04028	0.236892	1.78	4.5	1.13E-05	0.000639
Respiratory	FEV1 maximumValue strict	T	221665	NA	NA	NA	-0.469259	0.110836	-0.686498	-0.25202	2.30E-05	0.001272
Anthropometry	Standing height	T	378247	NA	NA	NA	0.344496	0.082736	0.182334	0.506658	3.13E-05	0.001694
Biological assays	Red blood cell erythrocyte count	T	367863	NA	NA	NA	0.337645	0.084064	0.17288	0.50241	5.91E-05	0.003125
Biological assays	Mean sphered cell volume	T	361769	NA	NA	NA	0.337383	0.08503	0.170725	0.504041	7.25E-05	0.003754
Digestive system	Pancreatitis (HES and self-reported)	T	379101	2304	376797	6.07	1.80395	0.456482	2.48	14.9	7.76E-05	0.003929
Digestive system	Pancreatic disease (HES and self-reported)	T	379101	2413	376688	5.79	1.75608	0.456633	2.37	14.2	0.00012	0.005891
Respiratory	Pneumonia (HES and self-reported)	T	379101	13446	365655	2.98	1.09042	0.283694	1.71	5.19	0.000121	0.005891
Summary	Other diseases of the respiratory system (HES)	T	379101	4856	374245	4.1	1.41056	0.390923	1.9	8.82	0.000308	0.013848
Cardiovascular	Other disorders of veins (HES)	T	379101	415	378686	13.2	2.58366	0.715184	3.26	53.8	0.000303	0.013848
Digestive system	Acute pancreatitis (HES)	T	379101	1763	377338	6.29	1.83819	0.508416	2.32	17	0.0003	0.013848
Haematology	Polycythaemia vera (HES and self-reported)	T	379101	393	378708	13.3	2.58713	0.716571	3.26	54.1	0.000306	0.013848
Biological assays	Platelet count	T	367861	NA	NA	NA	-0.295484	0.084168	-0.460453	-0.130515	0.000447	0.019709
Biological assays	Platelet crit	T	367858	NA	NA	NA	-0.290828	0.08416	-0.455782	-0.125874	0.000549	0.023766
Respiratory	Other respiratory disorders (HES)	T	379101	2907	376194	4.8	1.56886	0.457732	1.96	11.8	0.000609	0.025207
Respiratory	Respiratory failure (HES and self-reported)	T	379101	1951	377150	5.79	1.75557	0.511366	2.12	15.8	0.000597	0.025207
Respiratory	Spontaneous pneumothoraxrecurrent pneumothorax (HES and self-reported)	T	379101	1034	378067	7.46	2.00949	0.586653	2.36	23.6	0.000614	0.025207
Haematology	Myeloproliferative disorder (HES and self-reported)	T	379101	455	378646	11.6	2.44788	0.716259	2.84	47.1	0.000632	0.025495
Medication	Intestinal antiinflammatory agents	T	379101	25513	353588	2.28	0.822325	0.241521	1.42	3.65	0.000662	0.026278
Respiratory	Asthma (HES and self-reported)	T	379101	48549	330552	1.96	0.671757	0.200967	1.32	2.9	0.00083	0.032393
Operations and Procedures	Excision of lesion of organ NOC	T	379101	2108	376993	5.35	1.67746	0.507981	1.98	14.5	0.000959	0.036254
Cardiovascular	Cerebral aneurysm (HES and self-reported)	T	379101	545	378556	10.6	2.35938	0.714364	2.61	42.9	0.000957	0.036254
Summary	Metabolic disorders (HES)	T	379101	39911	339190	2.07	0.729215	0.221517	1.34	3.2	0.000995	0.036633
Respiratory	GOLD tobin	T	18309	NA	NA	NA	0.989849	0.300819	0.400244	1.57945	0.001	0.036633
Operations and Procedures	High cost neurology drugs	T	379101	1209	377892	6.77	1.91178	0.584733	2.15	21.3	0.001077	0.038869
Medication	Calcium	T	379101	6050	373051	3.62	1.28717	0.394623	1.67	7.85	0.001107	0.039348
Respiratory	Pneumonia organism unspecified (HES)	T	379101	8203	370898	3.09	1.12732	0.34749	1.56	6.1	0.001178	0.041245
Cancer	Large bowel cancer colorectal cancer (self-reported)	T	379101	526	378575	10	2.30443	0.716816	2.46	40.8	0.001305	0.045041
Cardiovascular	Congenital malformations of cardiac septa (HES)	T	379101	521	378580	9.81	2.28345	0.713913	2.42	39.8	0.001381	0.04699
Cardiovascular	Other cerebrovascular diseases (HES)	T	379101	2188	376913	5.09	1.62658	0.510896	1.87	13.8	0.001454	0.048747
Cardiovascular	Mitral valve prolapse (HES and self-reported)	T	379101	545	378556	9.68	2.26977	0.714999	2.38	39.3	0.001501	0.049635
Medication	Antacids	T	379101	6182	372919	3.45	1.23713	0.392166	1.6	7.43	0.001607	0.052421
Other	Birth weight	T	215536	NA	NA	NA	-0.335143	0.108465	-0.547734	-0.122552	0.002002	0.064426
Immuno-inflammation	Chronic skin ulcers (HES and self-reported)	T	379101	1331	377770	6.08	1.80545	0.586385	1.93	19.2	0.002077	0.065949
Cardiovascular	High cholesterol (HES and self-reported)	T	379101	62396	316705	0.362	-1.01528	0.334083	0.188	0.697	0.002374	0.074361
Summary	Other diseases of pleura (HES)	T	379101	6040	373061	3.21	1.16713	0.391468	1.49	6.92	0.002869	0.088574
Respiratory	FEV1 FVC ratio never smoked only strict	T	84281	NA	NA	NA	-0.495945	0.166538	-0.822359	-0.169531	0.002902	0.088574
Summary	Influenza and pneumonia (HES)	T	379101	9013	370088	2.8	1.03013	0.34725	1.42	5.53	0.003012	0.090748
Operations and Procedures	Extirpation of nail bed	T	379101	679	378422	8.29	2.11473	0.713772	2.05	33.6	0.003049	0.090748
Summary	Diseases of male genital organs (HES)	T	379101	17819	361282	2.39	0.873052	0.296203	1.34	4.28	0.003204	0.094174
Summary	Deforming dorsopathies (HES)	T	379101	2539	376562	4.42	1.48584	0.509335	1.63	12	0.003532	0.102547
Summary	Disorders of gallbladder biliary tract and pancreas (HES)	T	379101	16365	362736	2.34	0.850561	0.292807	1.32	4.16	0.003674	0.105403
Digestive system	Liver failurecirrhosis (HES and self-reported)	T	379101	1525	377576	5.45	1.69614	0.585817	1.73	17.2	0.003787	0.107355
Medication	Other systemic drugs for obstructive airway diseases	T	379101	21417	357684	2.15	0.766109	0.265836	1.28	3.62	0.003953	0.108185
Operations and Procedures	Diagnostic imaging procedures	T	379101	15463	363638	2.32	0.843638	0.292428	1.31	4.12	0.003915	0.108185
Musculoskeletal	BMD Combined	T	338180	NA	NA	NA	-0.249095	0.086357	-0.418354	-0.079836	0.003921	0.108185
Cardiovascular	Subarachnoid haemorrhage (HES)	T	379101	743	378358	7.78	2.05099	0.71369	1.92	31.5	0.004056	0.108504
Medication	Macrolides lincosamides and streptogramins	T	379101	736	378365	7.77	2.05078	0.71354	1.92	31.5	0.004052	0.108504
Operations and Procedures	Debridement and irrigation of joint	T	379101	1526	377575	5.27	1.66135	0.584383	1.68	16.6	0.00447	0.117026
Respiratory	COPDExacerbation GOLD2orHigher	T	8831	486	8345	8.05	2.08542	0.734492	1.91	34	0.004522	0.117026
Respiratory	COPDExacerbation NI HES SPIROMETRY	T	25110	2309	22801	3.25	1.17872	0.415011	1.44	7.33	0.004508	0.117026
Medication	Anti acne preparations for topical use	T	379101	1608	377493	5.16	1.64149	0.58425	1.64	16.2	0.004961	0.124573
Operations and Procedures	Radiology with contrast	T	379101	12447	366654	2.43	0.886668	0.315659	1.31	4.51	0.00497	0.124573
Operations and Procedures	Radiology procedures	T	379101	30410	348691	1.95	0.669682	0.237946	1.23	3.11	0.004886	0.124573
Biological assays	Monocyte percentage	T	367213	NA	NA	NA	0.235485	0.084526	0.069815	0.401155	0.005337	0.132363
Digestive system	Fibrosis and cirrhosis of liver (HES)	T	379101	756	378345	7.27	1.98376	0.714078	1.79	29.5	0.005468	0.134227
Operations and Procedures	Removal of malignant melanoma (self-reported)	T	379101	716	378385	7.24	1.97986	0.713946	1.79	29.3	0.005552	0.134894
Genitourinary	Bph benign prostatic hypertrophy (HES and self-reported)	T	379101	10618	368483	2.67	0.981941	0.354562	1.33	5.35	0.005615	0.135047
Symptoms	Abnormalities of gait and mobility (HES)	T	379101	1676	377425	5.01	1.61153	0.58581	1.59	15.8	0.005942	0.141488
Operations and Procedures	Excision of haemorrhoid	T	379101	2721	376380	4.01	1.38943	0.507919	1.48	10.9	0.006228	0.143964
Cardiovascular	Pulmonary embolism dvt (HES and self-reported)	T	379101	5241	373860	3.14	1.14562	0.418416	1.38	7.14	0.006182	0.143964
Biological assays	High light scatter reticulocyte percentage	T	361769	NA	NA	NA	-0.233033	0.085075	-0.39978	-0.066286	0.00616	0.143964
Operations and Procedures	Other operations on conjunctiva	T	379101	712	378389	7.06	1.95397	0.71641	1.73	28.7	0.006383	0.146124
Respiratory	Pneumothorax (HES)	T	379101	762	378339	6.86	1.92556	0.714609	1.69	27.8	0.007048	0.159825
Genitourinary	Other disorders of male genital organs (HES)	T	379101	2588	376513	3.96	1.37536	0.515269	1.44	10.9	0.007603	0.170781
Biological assays	Lymphocyte count	T	367207	NA	NA	NA	-0.221457	0.084475	-0.387027	-0.055887	0.008752	0.194759
Operations and Procedures	Inguinal femoral hernia repair (self-reported)	T	379101	12611	366490	2.33	0.844571	0.323548	1.23	4.39	0.009045	0.199412
Immuno-inflammation	Decubitus ulcer (HES)	T	379101	840	378261	6.37	1.85141	0.715541	1.57	25.9	0.009669	0.209298
Musculoskeletal	Kyphosis and lordosis (HES)	T	379101	201	378900	13.5	2.60571	1.00687	1.88	97.4	0.009656	0.209298
Respiratory	Respiratory failure (HES)	T	379101	1845	377256	4.54	1.51347	0.587404	1.44	14.4	0.009979	0.214063
Operations and Procedures	Diagnostic imaging of hepatobiliary system	T	379101	861	378240	6.21	1.82572	0.713551	1.53	25.1	0.010508	0.223395

NA	Pediatric asthma under16yo (self-reported)	T	350577	15307	335270	2.17	0.776859	0.30488	1.2	3.95	0.010832	0.228235
Cardiovascular	Subarachnoid haemorrhage (HES and self-reported)	T	379101	936	378165	6.06	1.80223	0.71331	1.5	24.5	0.011518	0.240567
Symptoms	Voice disturbances (HES)	T	379101	934	378167	6.03	1.79672	0.713908	1.49	24.4	0.011844	0.245231
Cardiovascular	Atrial fibrillation (HES and self-reported)	T	379101	2928	376173	3.62	1.28638	0.512621	1.33	9.89	0.012093	0.248225
Operations and Procedures	Release of fascia	T	379101	204	378897	12.3	2.51273	1.00666	1.72	88.7	0.012557	0.255536
Haematology	Acute posthaemorrhagic anaemia (HES)	T	379101	226	378875	12	2.48906	1.00674	1.68	86.7	0.013421	0.270815
NA	Subarachnoid haemorrhage	T	379101	982	378119	5.8	1.75793	0.713351	1.43	23.5	0.013727	0.274655
Medication	Blood glucose lowering drugs excl insulins	T	379101	49513	329588	0.426	-0.854162	0.35093	0.214	0.847	0.014933	0.28219
Summary	Diseases of liver (HES)	T	379101	4603	374498	3.04	1.11156	0.456502	1.24	7.44	0.014894	0.28219
Operations and Procedures	Incision of eyelid	T	379101	241	378860	11.6	2.45288	1.0064	1.62	83.5	0.014798	0.28219
Operations and Procedures	Diagnostic microendoscopic examination of larynx	T	379101	246	378855	11.7	2.46335	1.00757	1.63	84.6	0.014492	0.28219
Operations and Procedures	Diagnostic echocardiography	T	379101	8855	370246	2.45	0.898065	0.367569	1.19	5.05	0.014556	0.28219
Respiratory	Pneumothorax (HES and self-reported)	T	379101	906	378195	5.69	1.73951	0.714283	1.4	23.1	0.014878	0.28219
Biological assays	Reticulocyte percentage	T	361768	NA	NA	NA	-0.208663	0.085114	-0.375487	-0.041839	0.014224	0.28219
Operations and Procedures	Incision of sclera	T	379101	216	378885	11.6	2.45135	1.00848	1.61	83.8	0.015069	0.282507
Other	DIED	T	379101	10919	368182	2.34	0.849427	0.350112	1.18	4.64	0.01526	0.283852
Genitourinary	Hyperplasia of prostate (HES)	T	379101	10244	368857	2.44	0.891336	0.369212	1.18	5.03	0.015772	0.291104
Neurosciences	Back pain for 3 months vs no pain	T	341531	65312	276219	0.494	-0.704411	0.29296	0.278	0.878	0.016196	0.296643
Genitourinary	Prostate problem not cancer (HES and self-reported)	T	379101	11913	367188	2.33	0.847397	0.353098	1.17	4.66	0.0164	0.298079
Digestive system	Other diseases of pancreas (HES)	T	379101	1002	378099	5.44	1.69393	0.713639	1.34	22	0.017613	0.306861
Immuno-inflammation	Ulcer of lower limb (HES)	T	379101	989	378112	5.45	1.69496	0.715154	1.34	22.1	0.017785	0.306861
Family history	Chronic bronchitis emphysema (family history - mother)	T	357529	20553	336976	1.96	0.671889	0.282553	1.13	3.41	0.01741	0.306861
Operations and Procedures	Other operations on peripheral nerve	T	379101	2062	377039	4	1.38601	0.584192	1.27	12.6	0.017667	0.306861
Operations and Procedures	Secondary open reduction of fracture of bone	T	379101	1013	378088	5.49	1.70272	0.713669	1.36	22.2	0.017039	0.306861
Operations and Procedures	Harvest of random pattern flap of skin from limb	T	379101	233	378868	10.9	2.38973	1.0073	1.52	78.6	0.017672	0.306861
Eye	LogMAR final right	T	84003	NA	NA	NA	-0.400333	0.168893	-0.731363	-0.069303	0.017772	0.306861
Infectious disease	Bacterial agents as the cause of diseases (HES)	T	379101	7935	371166	2.51	0.920608	0.389321	1.17	5.39	0.018047	0.309136
Immuno-inflammation	Nail disorders (HES)	T	379101	1050	378051	5.33	1.67291	0.713216	1.32	21.6	0.018997	0.32079
NA	Adult only asthma over16yo (self-reported)	T	367188	31918	335270	1.79	0.583046	0.248518	1.1	2.92	0.018972	0.32079
Summary	HESCH Certain infectious and parasitic diseases BIN (HES)	T	379101	26714	352387	1.83	0.603365	0.259367	1.1	3.04	0.020003	0.330736
Operations and Procedures	Percutaneous examination of bile duct	T	379101	260	378841	10.4	2.34286	1.00659	1.45	74.9	0.019937	0.330736
Cardiovascular	Cholesterol lowering medication	T	376222	64923	311299	0.507	-0.67952	0.291517	0.286	0.897	0.019755	0.330736
Operations and Procedures	Plastic operations on cornea	T	379101	258	378843	10.3	2.33678	1.006	1.44	74.3	0.020188	0.331498
Neurosciences	Parkinson s disease (HES)	T	379101	1054	378047	5.25	1.65783	0.71687	1.29	21.4	0.020745	0.332122
Respiratory	Pleural effusion (HES)	T	379101	4725	374376	2.89	1.06222	0.458139	1.18	7.1	0.020419	0.332122
Cardiovascular	Varicose vein surgery (self-reported)	T	379101	15382	363719	2.08	0.732684	0.316653	1.12	3.87	0.020677	0.332122
Operations and Procedures	Extirpation of scrotum	T	379101	239	378862	10.3	2.33475	1.00989	1.43	74.7	0.020784	0.332122
Operations and Procedures	Recession of muscle of eye	T	379101	270	378831	10.2	2.32123	1.0059	1.42	73.2	0.021021	0.333667
Medication	Lipid modifying agents combinations	T	379101	72923	306178	0.54	-0.616855	0.268589	0.319	0.914	0.021639	0.341205
Operations and Procedures	Other diagnostic tests	T	379101	994	378107	5.08	1.62578	0.714074	1.25	20.6	0.0228	0.357155
Summary	Symptoms and signs involving the skin and subcutaneous tissue (HES)	T	379101	6510	372591	2.56	0.941261	0.417373	1.13	5.81	0.024121	0.366279
Cardiovascular	Nonrheumatic mitral valve disorders (HES)	T	379101	2168	376933	3.75	1.32291	0.586669	1.19	11.9	0.024137	0.366279
Digestive system	Other diseases of jaws (HES)	T	379101	301	378800	9.7	2.27191	1.00577	1.35	69.6	0.023892	0.366279
ENT	Dental tooth surgery (self-reported)	T	379101	999	378102	4.99	1.60748	0.71365	1.23	20.2	0.024292	0.366279
Operations and Procedures	Operations on canthus	T	379101	1131	377970	5.02	1.61357	0.713849	1.24	20.3	0.023798	0.366279
Respiratory	Pleural effusion (HES and self-reported)	T	379101	4887	374214	2.81	1.03192	0.458172	1.14	6.89	0.024306	0.366279
Mental health	Mental and behavioural disorders due to use of alcohol (HES)	T	379101	4907	374194	2.81	1.03424	0.460887	1.14	6.94	0.024831	0.366955
Operations and Procedures	Excision of gall bladder	T	379101	12079	367022	2.18	0.77793	0.346656	1.1	4.29	0.024826	0.366955
Operations and Procedures	Revisional decompression operations on lumbar spine	T	379101	287	378814	9.54	2.25594	1.00626	1.33	68.6	0.024967	0.366955
Musculoskeletal	Bursitis (HES and self-reported)	T	379101	2169	376932	3.71	1.31186	0.584149	1.18	11.7	0.024719	0.366955
Operations and Procedures	Harvest of other multiple tissue	T	379101	281	378820	9.47	2.24849	1.00681	1.32	68.2	0.025531	0.372233
Musculoskeletal	Scoliosis (HES and self-reported)	T	379101	1135	377966	4.93	1.5954	0.7149	1.21	20	0.025639	0.372233
Cardiovascular	Other venouslymphatic disease (HES and self-reported)	T	379101	1107	377994	4.9	1.58949	0.713202	1.21	19.8	0.025836	0.372816
Operations and Procedures	Removal of epididymal cyst (self-reported)	T	379101	269	378832	9.28	2.22828	1.00716	1.29	66.8	0.026937	0.38594
Biological assays	High light scatter reticulocyte count	T	361768	NA	NA	NA	-0.188124	0.085104	-0.354928	-0.02132	0.027069	0.38594
Summary	Congenital malformations of the circulatory system (HES)	T	379101	1083	378018	4.83	1.57402	0.712897	1.19	19.5	0.02725	0.386205
Respiratory	Pyothorax (HES)	T	379101	288	378813	9.01	2.19815	1.00651	1.25	64.8	0.028967	0.407326
Operations and Procedures	Anaesthetic without surgery	T	379101	299	378802	8.99	2.19586	1.00618	1.25	64.6	0.029083	0.407326
Summary	Diseases of arteries arterioles and capillaries (HES)	T	379101	6751	372350	2.51	0.918419	0.421749	1.1	5.73	0.029433	0.409819
Operations and Procedures	Liver surgery (self-reported)	T	379101	301	378800	8.86	2.18191	1.00581	1.23	63.6	0.030059	0.416105
Operations and Procedures	Therapeutic endoscopic operations on pleura	T	379101	310	378791	8.8	2.17437	1.00652	1.22	63.3	0.030751	0.423219
Respiratory	Empyema (HES and self-reported)	T	379101	299	378802	8.73	2.16647	1.00641	1.21	62.7	0.031345	0.428925
Digestive system	Incisional hernia (HES and self-reported)	T	379101	307	378794	8.69	2.16257	1.00593	1.21	62.4	0.03157	0.429531
Metabolic	Disorders of lipoprotein metabolism and other lipidaemias (HES)	T	379101	32636	346465	0.374	-0.984125	0.45975	0.152	0.92	0.032309	0.435566
Operations and Procedures	Gallstones removed (self-reported)	T	379101	315	378786	8.61	2.15332	1.00743	1.2	62	0.032562	0.435566
Mental health	Postnatal depression (HES and self-reported)	T	379101	311	378790	8.68	2.16119	1.01061	1.2	62.9	0.032477	0.435566
Neurosciences	Parkinsons disease (HES and self-reported)	T	379101	1191	377910	4.61	1.52717	0.716688	1.13	18.8	0.0331	0.440285
Respiratory	Unspecified acute lower respiratory infection (HES)	T	379101	6878	372223	2.44	0.890133	0.418963	1.07	5.54	0.033619	0.440342
Operations and Procedures	External resuscitation	T	379101	3496	375605	2.98	1.09119	0.513711	1.09	8.15	0.033659	0.440342
Other	Alcohol dependency (HES and self-reported)	T	379101	5185	373916	2.67	0.980374	0.460919	1.08	6.58	0.03342	0.440342
Operations and Procedures	Examination of oesophagus	T	379101	1296	377805	4.5	1.50464	0.714182	1.11	18.3	0.035134	0.457129
Family history	Chronic bronchitis emphysema (family history)	T	379101	NA	NA	NA	0.174729	0.083176	0.011703	0.337755	0.035667	0.461543
Medication	Antipsoriatics for topical use	T	379101	1197	377904	4.42	1.48526	0.713443	1.09	17.9	0.037358	0.473287
Cancer	Skin cancer (self-reported)	T	379101	1189	377912	4.43	1.48877	0.714906	1.09	18	0.037299	0.473287
Cardiovascular	Leg claudication intermittent claudication (HES and self-reported)	T	379101	2468	376633	3.43	1.23195	0.590667	1.08	10.9	0.037006	0.473287
Other	Pack years	T	110918	NA	NA	NA	-0.406884	0.195458	-0.789982	-0.023786	0.03737	0.473287
Summary	Other acute lower respiratory infections (HES)	T	379101	7023	372078	2.38	0.86796	0.418913	1.05	5.41	0.038271	0.479601
Cardiovascular	Aortic aneurysm and dissection (HES)	T	379101	1199	377902	4.46	1.49575	0.721875	1.08	18.4	0.038262	0.479601
Operations and Procedures	Other operations on blood vessel	T	379101	317	378784	8	2.07943	1.00733	1.11	57.6	0.038989	0.486041
Neurosciences	Alzheimer s disease (HES)	T	379101	359	378742	7.94	2.07186	1.01005	1.1	57.5	0.040243	0.496675
Cardiovascular	Endocarditis valve unspecified (HES)	T	379101	363	378738	7.88	2.06459	1.00659	1.1	56.7	0.04026	0.496675
Family history	Heart disease (family history - father)	T	343592	109130	234462	0.657	-0.419799	0.205104	0.44	0.982	0.040681	0.49673
Cardiovascular	Aortic aneurysm (HES and self-reported)	T	379101	1220	377881	4.38	1.47773	0.721841	1.06	18	0.040641	0.49673
Summary	HESCH Endocrine nutritional and metabolic diseases BIN (HES)	T	379101	66768	312333	1.51	0.410243	0.200979	1.02	2.23	0.041228	0.500838
Operations and Procedures	Partial excision of liver	T	379101	334	378767	7.75	2.04763	1.00633	1.08	55.7	0.041877	0.503578
Metabolic	Thyroid problem not cancer (HES and self-reported)	T	379101	1330	377771	4.29	1.45545	0.715232	1.06	17.4	0.041857	0.503578
Digestive system	Peptic ulcer site unspecified (HES)	T	379101	370	378731	7.66	2.03622	1.00834	1.06	55.3	0.043448	0.519843
Operations and Procedures	Other excision of appendix	T	379101	1270	377831	4.21	1.43746	0.712959	1.04	17	0.04378	0.5212
Summary	Suppurative and necrotic conditions of lower respiratory tract (HES)	T	379101	346	378755	7.57	2.0246	1.00617	1.05	54.4	0.044201	0.523478
Digestive system	Cholelithiasis (HES)	T	379101	12988	366113	2.01	0.696427	0.346448	1.02	3.96	0.044411	0.523478
Eye	Iridocyclitis (HES)	T	379101	333	378768	7.52	2.01754	1.00555	1.05	54	0.044814	0.525627
Symptoms	Haemorrhage from respiratory passages (HES)	T	379101	4000	375101	2.77	1.02015	0.509545	1.02	7.53	0.045276	0.528443
Operations and Procedures	Introduction of radioactive material into organ NOC	T	379101	376	378725	7.37	1.99709	1.00619	1.03	52.9	0.047167	0.547825
Operations and Procedures	Intramuscular injection	T	379101	1308	377793	4.09	1.40818	0.713571	1.01	16.6	0.048448	0.559972
Anthropometry	Body mass index BMI	T	377835	NA	NA	NA	-0.163861	0.083277	-0.327085	-0.000637	0.049107	0.564854
Operations and Procedures	Diagnostic transluminal operations on other artery	T	379101	397	378704	7.19	1.97307	1.00722	0.999	51.8	0.050122	0.573756
Musculoskeletal	Other disorders of bone (HES)	T	379101	1394	377707	4.02	1.39212	0.712929	0.995	16.3	0.050858	0.574356
Family history	Bowel cancer (family history - mother)	T	354181	17920	336261	1.8	0.590492	0.302769	0.997	3.27	0.051139	0.574356
Other	Chest pain or discomfort walking normally	T	58732	10166	48566	2.21	0.791538	0.406274	0.995	4.89	0.051381	0.574356
Operations and Procedures	Transluminal operations on pulmonary artery	T	379101	411	378690	7.17	1.96962	1.01069	0.989	52	0.05132	0.574356
Operations and Procedures	Diagnostic blood tests	T	379101	378	378723	7.09	1.95915	1.00549	0.988	50.9	0.05136	0.574356
Operations and Procedures	Puncture of pleura	T	379101	2582	376519	3.12	1.13685	0.585572	0.989	9.82	0.052206	0.580856
Musculoskeletal	Osteoporosis (HES and self-reported)	T	379101	10107	368994	2.16	0.768221	0.397743	0.989	4.7	0.053428	0.588939
Biological assays	Mean reticulocyte volume	T	361768	NA	NA	NA	0.164472	0.085075	-0.002275	0.331219	0.053204	0.588939
Neurosciences	Hemiplegia (HES)	T	379101	1363	377738	3.95	1.3746	0.713631	0.976	16	0.05408	0.590999
Musculoskeletal	Other soft tissue disorders (HES)	T	379101	11219	367882	2.02	0.702055	0.364524	0.988	4.12	0.054111	0.590999
Operations and Procedures	Minimal access to other body cavity	T	379101	8899	370202	2.11	0.745559	0.387898	0.985	4.51	0.0546	0.593619
Summary	Disorders of optic nerve and visual pathways (HES)	T	379101	383	378718	6.83	1.92095	1.00541	0.952	49	0.056053	0.603903
Cancer	Follicular (cancer register)	T	379101	389	378712	6.84	1.92331	1.00562	0.953	49.1	0.055804	0.603903
Summary	Other diseases of blood and blood forming organs (HES)	T	379101	4191	374910	2.63	0.966133	0.507985	0.971	7.11	0.057185	0.605758
Summary	Cerebrovascular diseases (HES)	T	379101	7435	371666	2.23	0.802797	0.421082	0.978	5.09	0.056584	0.605758
Symptoms	Senility (HES)	T	379101	414	378687	6.8	1.91623	1.00777	0.943	49	0.057243	0.605758
ENT	Nasal polyps (HES and self-reported)	T	379101	4016	375085	2.63	0.967908	0.508906	0.971	7.14	0.057179	0.605758
Metabolic	Other nontoxic goitre (HES)	T	379101	1508	377593	3.86	1.35103	0.714707	0.951	15.7	0.058715	0.617831
Operations and Procedures	Operations on gingiva	T	379101	429	378672	6.68	1.89923	1.00546	0.931	47.9	0.058903	0.617831

Symptoms	Ascites (HES)	T	379101	1446	377655	3.8	1.33473	0.713333	0.939	15.4	0.061329	0.637662
Digestive system	Liverbiliarypancreas problem (HES and self-reported)	T	379101	4283	374818	2.59	0.951177	0.508218	0.956	7.01	0.061263	0.637662
Infectious disease	Zoster (HES)	T	379101	425	378676	6.55	1.87905	1.00584	0.912	47	0.061742	0.63916
Biological assays	Immature reticulocyte fraction	T	361768	NA	NA	NA	-0.158043	0.084985	-0.324614	0.008528	0.062935	0.648694
Operations and Procedures	Incision of capsule of lens	T	379101	1497	377604	3.75	1.32261	0.715894	0.923	15.3	0.064675	0.660909
Mental health	Ever unenthusiastic disinterested for a whole week	T	121405	44857	76548	0.583	-0.539354	0.291694	0.329	1.03	0.064452	0.660909
Medication	Corticosteroids combinations with antibiotics	T	379101	20857	358244	1.71	0.536403	0.291184	0.966	3.03	0.065454	0.666006
Operations and Procedures	Image controlled operations on abdominal cavity	T	379101	419	378682	6.34	1.84663	1.00527	0.884	45.5	0.066217	0.670905
Digestive system	Unspecified appendicitis (HES)	T	379101	412	378689	6.26	1.8336	1.00541	0.872	44.9	0.068192	0.682461
Medication	Hormone antagonists and related agents	T	379101	2999	376102	2.93	1.07612	0.589661	0.923	9.32	0.068004	0.682461
Medication	Vitamin B	T	377036	15815	361221	0.164	-1.80782	0.992391	0.0234	1.15	0.068504	0.682461
Infectious disease	Whooping cough pertussis (HES and self-reported)	T	379101	415	378686	6.25	1.83319	1.00581	0.871	44.9	0.068365	0.682461
Musculoskeletal	Soft tissue disorders related to use overuse and pressure (HES)	T	379101	1500	377601	3.63	1.28852	0.712902	0.897	14.7	0.070696	0.695564
Digestive system	Bile duct disease (HES and self-reported)	T	379101	13856	365245	1.87	0.625991	0.3463	0.949	3.69	0.070661	0.695564
Cardiovascular	Primary hypertension I270	T	379101	416	378685	6.18	1.82191	1.00683	0.859	44.5	0.070366	0.695564
Family history	High blood pressure (family history - father)	T	343592	74698	268894	0.649	-0.431596	0.239398	0.406	1.04	0.071414	0.698221
Operations and Procedures	Diagnostic endoscopic examination of lower bowel	T	379101	29456	349645	0.44	-0.820414	0.455291	0.18	1.07	0.071552	0.698221
ENT	Nasalsinus disorder (HES and self-reported)	T	379101	11324	367777	1.93	0.656002	0.364601	0.943	3.94	0.071982	0.699548
Cardiovascular	Other aneurysm (HES)	T	379101	436	378665	6.09	1.80716	1.00664	0.847	43.8	0.072616	0.702843
Mental health	Overall health rating	T	377441	NA	NA	NA	0.149492	0.083363	-0.0139	0.312884	0.072931	0.703031
Musculoskeletal	Fractured broken bones in last 5 years	T	377028	36458	340570	1.54	0.430829	0.24123	0.959	2.47	0.074105	0.70316
Operations and Procedures	Other operations on sympathetic nerve	T	379101	470	378631	6.04	1.79767	1.00501	0.842	43.3	0.073663	0.70316
Operations and Procedures	Other operations on maxillary antrum	T	379101	2872	376229	2.85	1.04613	0.584105	0.906	8.94	0.073295	0.70316
Digestive system	Gall bladder disease (HES and self-reported)	T	379101	13958	365143	1.86	0.618528	0.346352	0.941	3.66	0.074126	0.70316
Summary	Disorders of sclera cornea iris and ciliary body (HES)	T	379101	1464	377637	3.56	1.27061	0.713108	0.881	14.4	0.074784	0.70526
Operations and Procedures	Removal of repair material from skin	T	379101	460	378641	5.99	1.78979	1.00503	0.835	42.9	0.074939	0.70526
Respiratory	Other disorders of nose and nasal sinuses (HES)	T	379101	5991	373110	2.25	0.81111	0.456263	0.92	5.5	0.075449	0.707259
Neurosciences	Hip pain for 3 months vs no pain	T	362682	32691	329991	0.478	-0.737719	0.418358	0.211	1.09	0.077839	0.726799
Operations and Procedures	Other operations on internal nose	T	379101	2843	376258	2.8	1.03058	0.585191	0.89	8.82	0.078221	0.727517
Summary	Bacterial viral and other infectious agents (HES)	T	379101	11829	367272	1.9	0.642204	0.365104	0.929	3.89	0.078584	0.727522
Summary	Noninflammatory disorders of female genital tract (HES)	T	379101	43955	335146	0.534	-0.626863	0.357043	0.265	1.08	0.079138	0.727522
Musculoskeletal	Other deforming dorsopathies (HES)	T	379101	1558	377543	3.5	1.25329	0.713777	0.864	14.2	0.079112	0.727522
Eye	Retinal vascular occlusions (HES)	T	379101	466	378635	5.84	1.76482	1.00688	0.812	42	0.079643	0.727528
Operations and Procedures	Removal of other substance from skin	T	379101	434	378667	5.82	1.76109	1.00511	0.811	41.7	0.07975	0.727528
Summary	Had major operations	T	173367	111364	62003	1.6	0.469253	0.268941	0.944	2.71	0.081017	0.736262
Gynaecology and Obstetrics	Uterine problem (HES and self-reported)	T	379101	29689	349412	0.447	-0.805553	0.463947	0.18	1.11	0.08251	0.746984
Digestive system	Other diseases of gallbladder (HES)	T	379101	1619	377482	3.43	1.23335	0.713363	0.848	13.9	0.083822	0.755986
Operations and Procedures	Ventilation support	T	379101	2964	376137	2.75	1.01216	0.586358	0.872	8.68	0.084316	0.75757
Operations and Procedures	Total prosthetic replacement of hip joint using cement	T	379101	4901	374200	2.42	0.885797	0.513827	0.886	6.64	0.084723	0.758362
Haematology	Diseases of spleen (HES)	T	379101	463	378638	5.63	1.72816	1.00506	0.785	40.4	0.085529	0.762717
Genitourinary	Renal failure not requiring dialysis (HES and self-reported)	T	379101	483	378618	5.62	1.72568	1.00557	0.783	40.3	0.08614	0.765297
Digestive system	Other diseases of anus and rectum (HES)	T	379101	21218	357883	0.368	-0.999652	0.583888	0.117	1.16	0.086885	0.768643
Respiratory	FEV1 FVC ratio never smoked only	T	109851	NA	NA	NA	-0.25205	0.147349	-0.540854	0.036754	0.087162	0.768643
Summary	HESCH Diseases of the blood forming organ immune mech BIN (HES)	T	379101	22345	356756	1.64	0.497231	0.291836	0.928	2.91	0.088418	0.771145
Operations and Procedures	Operations on turbinate of nose	T	379101	2985	376116	2.71	0.99654	0.584497	0.862	8.52	0.088203	0.771145
Operations and Procedures	Primary fusion of other joint of spine	T	379101	465	378636	5.55	1.71407	1.00521	0.774	39.8	0.088159	0.771145
Symptoms	Rash and other nonspecific skin eruption (HES)	T	379101	1635	377466	3.35	1.20988	0.712829	0.829	13.6	0.089642	0.771305
Operations and Procedures	Diagnostic lower respiratory tract	T	379101	4809	374292	2.38	0.865439	0.509684	0.875	6.45	0.089509	0.771305
Operations and Procedures	Other diagnostic imaging of vascular system	T	379101	2957	376144	2.7	0.994041	0.585014	0.858	8.51	0.089286	0.771305
Biological assays	Microalbumin in urine	T	114296	NA	NA	NA	-0.235276	0.138657	-0.507044	0.036492	0.089732	0.771305
Respiratory	Nasal polyp (HES)	T	379101	2942	376159	2.69	0.990506	0.585259	0.855	8.48	0.090565	0.773486
Biological assays	White blood cell leukocyte count	T	367858	NA	NA	NA	-0.142424	0.084172	-0.307402	0.022554	0.090635	0.773486
Other	Getting up in morning	T	378063	NA	NA	NA	-0.14059	0.083349	-0.303954	0.022774	0.09165	0.779351
Neurosciences	Hip pain	T	378231	48240	329991	0.59	-0.527772	0.314409	0.319	1.09	0.093227	0.788288
Genitourinary	Enlarged prostate (HES and self-reported)	T	379101	13062	366039	1.86	0.620536	0.369825	0.901	3.84	0.093363	0.788288
Cardiovascular	Leg artery angioplasty stent (self-reported)	T	379101	443	378658	5.43	1.69178	1.01059	0.749	39.4	0.09412	0.788362
Operations and Procedures	Other operations on retina	T	379101	498	378603	5.37	1.67998	1.0063	0.746	38.6	0.095027	0.788362
Medication	Glucosamine	T	377850	73561	304289	0.665	-0.407606	0.243785	0.413	1.07	0.094526	0.788362
Cardiovascular	Angina (HES and self-reported)	T	379101	20423	358678	0.374	-0.984646	0.588132	0.118	1.18	0.094093	0.788362
Eye	Iritis (HES and self-reported)	T	379101	479	378622	5.35	1.67786	1.00487	0.747	38.4	0.094972	0.788362
Digestive system	Other diseases of liver (HES)	T	379101	3122	375979	2.64	0.970318	0.584492	0.839	8.3	0.096893	0.797605
Operations and Procedures	Other operations on fallopian tube	T	379101	950	378151	5.45	1.6949	1.02089	0.736	40.3	0.096871	0.797605
Biological assays	Reticulocyte count	T	361768	NA	NA	NA	-0.14122	0.085132	-0.308078	0.025638	0.097146	0.797605
Symptoms	Other symptoms and signs involving the digestive system and abdomen (HES)	T	379101	13806	365295	0.193	-1.64394	0.992317	0.0276	1.35	0.097587	0.798468
Symptoms	Nausea and vomiting (HES)	T	379101	10633	368468	1.9	0.640931	0.388402	0.887	4.06	0.098907	0.80123
Medication	Antiinflammatory and antirheumatic products non steroids	T	379101	86308	292793	0.69	-0.370769	0.224347	0.445	1.07	0.098401	0.80123
Neurosciences	Peripheral nerve disorder (HES and self-reported)	T	379101	16736	362365	0.309	-1.17361	0.711969	0.0766	1.25	0.09927	0.80123
Digestive system	Anal problem (HES and self-reported)	T	379101	23889	355212	0.434	-0.835548	0.506408	0.161	1.17	0.098954	0.80123
Neurosciences	Knee pain for 3 months vs no pain	T	354701	62522	292179	0.644	-0.439752	0.267054	0.382	1.09	0.099624	0.801367
Summary	Symptoms nervous and musculoskeletal systems (HES)	T	379101	4763	374338	2.29	0.830502	0.508365	0.847	6.21	0.102328	0.802491
Cardiovascular	Angina pectoris (HES)	T	379101	16491	362610	0.311	-1.16808	0.714789	0.0766	1.26	0.102224	0.802491
Musculoskeletal	Other necrotizing vasculopathies (HES)	T	379101	542	378559	5.19	1.64674	1.00651	0.722	37.3	0.101823	0.802491
Cancer	Rodent ulcer (self-reported)	T	379101	540	378561	5.19	1.64728	1.00617	0.723	37.3	0.101593	0.802491
Operations and Procedures	Blood withdrawal	T	379101	3031	376070	2.6	0.956402	0.584281	0.828	8.18	0.101654	0.802491
Metabolic	Thyroid goitre (HES and self-reported)	T	379101	1793	377308	3.22	1.16923	0.714835	0.793	13.1	0.101911	0.802491
Biological assays	Mean corpuscular haemoglobin	T	367860	NA	NA	NA	0.137148	0.083983	-0.027459	0.301755	0.10246	0.802491
Respiratory	FEV1 never smoked only strict	T	84281	NA	NA	NA	-0.272768	0.166279	-0.598675	0.053139	0.100917	0.802491
Operations and Procedures	Rectal sigmoid resection (self-reported)	T	379101	534	378567	5.15	1.63941	1.0054	0.718	37	0.102974	0.803872
Symptoms	Gangrene (HES)	T	379101	510	378591	5.14	1.63675	1.00567	0.716	36.9	0.103626	0.806319
Eye	Hordeolum and chalazion (HES)	T	379101	1665	377436	3.18	1.15741	0.712734	0.787	12.9	0.104397	0.807043
Medication	Drugs affecting bone structure and mineralization	T	379101	7218	371883	2.13	0.755425	0.464743	0.856	5.29	0.104063	0.807043
Eye	Intra ocular pressure Goldmann correlated right	T	81176	NA	NA	NA	-0.273691	0.169056	-0.605041	0.057659	0.105461	0.81263
Immuno-inflammation	Granulomatous disorders of skin and subcutaneous tissue (HES)	T	379101	534	378567	5.04	1.61809	1.00479	0.704	36.1	0.107315	0.8229
Gynaecology and Obstetrics	Uterine polypectomy uterine polyps removed (self-reported)	T	379101	529	378572	5.08	1.62446	1.00923	0.702	36.7	0.107485	0.8229
Cardiovascular	Other peripheral vascular diseases (HES)	T	379101	3304	375797	2.56	0.941595	0.586937	0.812	8.1	0.108658	0.828805
Musculoskeletal	Osteoporosis without pathological fracture (HES)	T	379101	5396	373705	2.28	0.823023	0.514668	0.831	6.24	0.109791	0.828805
Gynaecology and Obstetrics	Medical abortion (HES)	T	379101	998	378103	5.14	1.63664	1.02385	0.691	38.2	0.109927	0.828805
Other	Chest pain or discomfort when walking uphill or hurrying	T	47258	12507	34751	2.15	0.765793	0.480095	0.839	5.51	0.110693	0.828805
Operations and Procedures	Diagnostic endoscopic examination of larynx	T	379101	3294	375807	2.55	0.934349	0.584722	0.809	8.01	0.110057	0.828805
Operations and Procedures	Diagnostic percutaneous operations on liver	T	379101	1755	377346	3.13	1.14176	0.712931	0.774	12.7	0.109267	0.828805
Eye	Intra ocular pressure Goldmann correlated left	T	81056	NA	NA	NA	-0.269843	0.169065	-0.60121	0.061524	0.110468	0.828805
Summary	Nerve nerve root and plexus disorders (HES)	T	379101	16103	362998	0.322	-1.13235	0.71203	0.0798	1.3	0.111764	0.834201
Other	Poisoning by narcotics and psychodysleptics (HES)	T	379101	598	378503	4.93	1.59523	1.00541	0.687	35.4	0.112594	0.83777
Symptoms	Other skin changes (HES)	T	379101	565	378536	4.86	1.58099	1.00485	0.678	34.8	0.115637	0.856598
Family history	Heart disease (family history - sibling)	T	301029	29868	271161	0.539	-0.618146	0.393107	0.249	1.16	0.115844	0.856598
Family history	High blood pressure (family history)	T	379101	NA	NA	NA	-0.13037	0.083196	-0.293434	0.032694	0.11711	0.863278
Respiratory	Pneumonitis due to solids and liquids (HES)	T	379101	560	378541	4.83	1.5747	1.00635	0.672	34.7	0.117639	0.86357
Operations and Procedures	Anal fissure surgery (self-reported)	T	379101	526	378575	4.81	1.57138	1.00488	0.672	34.5	0.117875	0.86357
Symptoms	Convulsions (HES)	T	379101	1802	377299	3.04	1.11348	0.713095	0.753	12.3	0.118413	0.864851
Mental health	Single Probable major depression episode	T	91515	6104	85411	1.98	0.682062	0.438061	0.838	4.67	0.11947	0.869902
Medication	Antispasmodics in combination with analgesics	T	379101	607	378494	4.75	1.55768	1.00513	0.662	34	0.121205	0.872912
Summary	Extrapyramidal and movement disorders (HES)	T	379101	1814	377287	3.02	1.10514	0.714525	0.744	12.3	0.121942	0.872912
Gynaecology and Obstetrics	Hysterectomy with oophorectomy (self-reported)	T	379101	668	378433	4.8	1.56842	1.01055	0.662	34.8	0.120652	0.872912
Operations and Procedures	Other methods of operation on organ NOC	T	379101	1830	377271	3.02	1.10381	0.713373	0.745	12.2	0.121788	0.872912
Other	Morning evening person chronotype	T	338613	NA	NA	NA	0.134202	0.086801	-0.035929	0.304333	0.122083	0.872912
Eye	Intra ocular pressure corneal compensated right	T	81176	NA	NA	NA	-0.261818	0.168994	-0.593046	0.06941	0.121316	0.872912
Cancer	Non melanoma skin cancer (self-reported)	T	379101	550	378551	4.73	1.55291	1.00556	0.658	33.9	0.12251	0.873342
Operations and Procedures	Transluminal operations on iliac artery	T	379101	580	378521	4.73	1.55336	1.00935	0.654	34.2	0.123811	0.879982
Operations and Procedures	Excision reconstruction of joint	T	379101	1872	377229	3	1.09725	0.714604	0.738	12.2	0.124671	0.883457
Infectious disease	Diarrhoea presumed infectious (HES)	T	379101	5076	374025	2.17	0.776513	0.507751	0.804	5.88	0.126185	0.88619
Haematology	Iron deficiency anaemia (HES)	T	379101	6915	372186	2.01	0.698116	0.455953	0.822	4.91	0.125741	0.88619
Cardiovascular	Aortic valve repair replacement (self-reported)	T	379101	561	378540	4.64	1.53402	1.00659	0.645	33.3	0.127515	0.88619
Other	Chest pain or discomfort	T	375192	59237	315955	1.37	0.317826	0.208632	0.913	2.07	0.127662	0.88619
Summary	Mal neo of central nervous system (cancer register)	T	379101	562	378539	4.64	1.53493	1.00493	0.647	33.3	0.126663	0.88619
Operations and Procedures	Diagnostic testing of genitourinary system	T	379101	578	378523	4.66	1.53867	1.00604	0.648	33.5	0.126159	0.88619

Operations and Procedures	Prosthetic replacement of other bone using cement	T	379101	559	378542	4.63	1.53302	1.00617	0.645	33.3	0.127603	0.88619
Immuno-inflammation	Cellulitis (HES and self-reported)	T	379101	6668	372433	2	0.693277	0.456408	0.818	4.89	0.128766	0.891255
Eye	Other disorders of cornea (HES)	T	379101	582	378519	4.59	1.52448	1.00507	0.641	32.9	0.129319	0.892056
Cardiovascular	Diastolic blood pressure mean	T	357246	NA	NA	NA	0.129927	0.085729	-0.038101	0.297955	0.129631	0.892056
Summary	HESCH Congen malfor deform and chrom abnormalities (HES)	T	379101	4981	374120	2.15	0.765167	0.507369	0.795	5.81	0.131527	0.89221
Operations and Procedures	Endoscopic ultrasound examination of pancreas	T	379101	605	378496	4.57	1.51947	1.00507	0.637	32.8	0.130584	0.89221
Operations and Procedures	Excision of nail	T	379101	608	378493	4.56	1.51693	1.00466	0.636	32.7	0.131069	0.89221
Operations and Procedures	Injection of other substance into organ NOC	T	379101	1838	377263	2.93	1.07611	0.713389	0.725	11.9	0.131439	0.89221
Immuno-inflammation	Allergy or anaphylactic reaction to food (HES and self-reported)	T	379101	1833	377268	2.93	1.0757	0.713014	0.725	11.9	0.131383	0.89221
Medication	Iron	T	377850	11432	366418	0.226	-1.48783	0.991979	0.0323	1.58	0.13365	0.901475
Cardiovascular	Mitral valve disease (HES and self-reported)	T	379101	3385	375716	2.41	0.880128	0.586735	0.763	7.62	0.133603	0.901475
Operations and Procedures	Urethral catheterisation of bladder	T	379101	10309	368792	1.79	0.584905	0.39091	0.834	3.86	0.134585	0.905217
Infectious disease	Other septicaemia (HES)	T	379101	3387	375714	2.39	0.872661	0.585169	0.76	7.54	0.135883	0.90991
Mental health	Probable recurrent major depression moderate	T	58838	11697	47141	0.403	-0.90992	0.610408	0.122	1.33	0.136047	0.90991
Other	Snoring	T	352753	131409	221344	1.3	0.264232	0.178399	0.918	1.85	0.138572	0.913961
Operations and Procedures	Lung removal pneumonectomy lobectomy (self-reported)	T	379101	614	378487	4.44	1.49109	1.00585	0.619	31.9	0.138228	0.913961
Operations and Procedures	Diagnostic endoscopic examination of ileum	T	379101	590	378511	4.45	1.49319	1.00462	0.621	31.9	0.137194	0.913961
Eye	Retinal arteryvein occlusion (HES and self-reported)	T	379101	614	378487	4.45	1.49307	1.0063	0.619	32	0.137881	0.913961
Cardiovascular	Pericardial effusion (HES and self-reported)	T	379101	600	378501	4.43	1.48919	1.00543	0.618	31.8	0.138568	0.913961
Operations and Procedures	Introduction of non removable material into organ NOC	T	379101	611	378490	4.44	1.4896	1.00701	0.616	31.9	0.139077	0.914758
Symptoms	Hepatomegaly and splenomegaly (HES)	T	379101	590	378511	4.41	1.48395	1.00563	0.614	31.7	0.140039	0.918548
Operations and Procedures	High cost immunosuppressant drugs	T	379101	602	378499	4.4	1.48072	1.00483	0.613	31.5	0.140588	0.919615
Genitourinary	Diseases of Bartholin s gland (HES)	T	379101	665	378436	4.39	1.47931	1.00932	0.607	31.7	0.142744	0.93116
Summary	Diabetes mellitus (HES)	T	379101	17982	361119	0.425	-0.856221	0.585735	0.135	1.34	0.143799	0.935479
Haematology	Anaemia in chronic diseases classified elsewhere (HES)	T	379101	657	378444	4.33	1.46447	1.00553	0.603	31	0.145279	0.94134
Musculoskeletal	Psoriatic and enteropathic arthropathies (HES)	T	379101	650	378451	4.31	1.46181	1.0046	0.602	30.9	0.145637	0.94134
Operations and Procedures	Excision of organ NOC	T	379101	1832	377269	2.82	1.03636	0.712657	0.697	11.4	0.145886	0.94134
Infectious disease	Candidiasis (HES)	T	379101	1983	377118	2.8	1.0283	0.713094	0.691	11.3	0.149295	0.956511
ENT	Tonsillectomy adenoids (self-reported)	T	379101	59321	319780	0.682	-0.383031	0.265451	0.405	1.15	0.149036	0.956511
Operations and Procedures	Open drainage of bladder	T	379101	606	378495	4.26	1.44876	1.00505	0.594	30.5	0.14945	0.956511
Digestive system	Haemochromatosis (HES and self-reported)	T	379101	635	378466	4.26	1.44989	1.00681	0.592	30.7	0.149844	0.956511
Operations and Procedures	Excision of dental lesion of jaw	T	379101	624	378477	4.2	1.43608	1.00454	0.587	30.1	0.152837	0.970413
Operations and Procedures	Excision of sigmoid colon	T	379101	638	378463	4.22	1.43868	1.00539	0.587	30.2	0.152437	0.970413
Summary	Other congenital malformations of the digestive system (HES)	T	379101	632	378469	4.19	1.43164	1.00495	0.584	30	0.154276	0.976945
Operations and Procedures	Suture of organ NOC	T	379101	640	378461	4.16	1.42615	1.00454	0.581	29.8	0.155693	0.983302
Immuno-inflammation	Pruritus (HES)	T	379101	705	378396	4.16	1.42475	1.00459	0.58	29.8	0.156121	0.983397
Gynaecology and Obstetrics	Mastectomy (self-reported)	T	379101	3844	375257	2.31	0.835868	0.591352	0.724	7.35	0.157513	0.987445
Musculoskeletal	Grip strength maximumValue	T	378322	NA	NA	NA	-0.117497	0.083142	-0.280455	0.045461	0.157593	0.987445
Summary	HESCH Diseases of the eye and adnexa (HES)	T	379101	35574	343527	1.44	0.362717	0.257085	0.868	2.38	0.158279	0.98914
Operations and Procedures	Other operations on spermatic cord	T	379101	598	378503	4.14	1.42065	1.00905	0.573	29.9	0.159158	0.98952
Operations and Procedures	Other large intestine	T	379101	3469	375632	2.28	0.822278	0.58406	0.724	7.15	0.159171	0.98952
Respiratory	Pleural plaque (HES)	T	379101	645	378456	4.11	1.4135	1.01329	0.564	30	0.163026	0.995679
Eye	Eye surgery (self-reported)	T	379101	3495	375606	2.26	0.815711	0.583944	0.72	7.1	0.162444	0.995679
Neurosciences	Stomach abdominal pain for 3 months vs no pain	T	357806	17535	340271	0.443	-0.814884	0.584247	0.141	1.39	0.163089	0.995679
Operations and Procedures	Freeing of tendon	T	379101	652	378449	4.08	1.40662	1.00453	0.57	29.2	0.16143	0.995679
Neurosciences	Back pain	T	378231	102012	276219	0.753	-0.284297	0.202874	0.506	1.12	0.16111	0.995679
Metabolic	Type 2 diabetes (HES and self-reported)	T	379101	17351	361750	0.441	-0.819551	0.58602	0.14	1.39	0.161962	0.995679
Gynaecology and Obstetrics	Vaginal prolapseuterine prolapse (HES and self-reported)	T	379101	11476	367625	0.243	-1.41275	1.00829	0.0337	1.76	0.161174	0.995679
Medication	Stomatological preparations	T	379101	59623	319478	0.914	-0.089727	0.243209	0.568	1.47	0.712182	0.998648
Medication	Drugs for peptic ulcer and gastro oesophageal reflux disease gord	T	379101	45634	333467	1.18	0.16366	0.248861	0.723	1.92	0.510771	0.998648
Medication	Drugs for functional gastrointestinal disorders	T	379101	3769	375332	0.752	-0.284907	1.00434	0.105	5.38	0.776658	0.998648
Medication	Propulsives	T	379101	1384	377717	2.06	0.72186	1.00443	0.287	14.7	0.472339	0.998648
Medication	Antiemetics and antinauseants	T	379101	234	378867	0.000121	-9.01858	197.217	1.62E-172	9.07E+163	0.963526	0.998648
Medication	Bile therapy	T	379101	233	378868	0.00012	-9.02821	194.156	6.46E-170	2.23E+161	0.962912	0.998648
Medication	Drugs for constipation	T	379101	6110	372991	0.46	-0.777074	1.00437	0.0642	3.29	0.439113	0.998648
Medication	Intestinal antiinfectives	T	379101	693	378408	0.000103	-9.18444	120.214	4.82E-107	2.19E+98	0.9391	0.998648
Medication	Antipropulsives	T	379101	2041	377060	8.91E-05	-9.32607	73.2745	3.78E-67	2.10E+58	0.898722	0.998648
Medication	Antiobesity preparations excl diet products	T	379101	958	378143	7.51E-05	-9.49719	120.415	2.38E-107	2.37E+98	0.937135	0.998648
Medication	Digestives incl enzymes	T	379101	255	378846	9.96E-05	-9.21478	197.598	6.30E-173	1.57E+164	0.962805	0.998648
Medication	Multivitamins combinations	T	379101	19000	360101	1.03	0.028753	0.388049	0.481	2.2	0.940935	0.998648
Medication	Vitamin a and d incl combinations of the two	T	379101	10919	368182	0.522	-0.650789	0.715904	0.128	2.12	0.363327	0.998648
Medication	Vitamin b1 plain and in combination with vitamin b6 and b12	T	379101	3169	375932	6.20E-05	-9.68835	73.1822	3.15E-67	1.22E+58	0.894678	0.998648
Medication	Ascorbic acid vitamin c incl combinations	T	379101	6192	372909	1.81	0.592913	0.507561	0.669	4.89	0.242742	0.998648
Medication	Other plain vitamin preparations	T	379101	4840	374261	4.11E-05	-10.0997	72.8733	3.83E-67	4.41E+57	0.889772	0.998648
Medication	Other mineral supplements	T	379101	878	378223	8.93E-05	-9.32369	117.648	6.41E-105	1.24E+96	0.936833	0.998648
Medication	Antithrombotic agents	T	379101	59142	319959	0.812	-0.208092	0.255159	0.493	1.34	0.414764	0.998648
Medication	Antifibrinolytics	T	379101	502	378599	8.14E-05	-9.41629	176.66	3.42E-155	1.93E+146	0.957491	0.998648
Medication	Iron preparations	T	379101	19982	359119	0.824	-0.193824	0.417581	0.363	1.87	0.642534	0.998648
Medication	Vitamin b12 and folic acid	T	379101	8262	370839	0.343	-1.06999	1.00387	0.048	2.45	0.286485	0.998648
Medication	Irrigating solutions	T	379101	1449	377652	5.24E-05	-9.85731	118.734	4.47E-106	6.13E+96	0.933836	0.998648
Medication	I v solution additives	T	379101	936	378165	8.36E-05	-9.38894	117.979	3.14E-105	2.23E+96	0.93657	0.998648
Medication	Cardiac glycosides	T	379101	1042	378059	2.56	0.941801	1.00876	0.355	18.5	0.350498	0.998648
Medication	Antiarrhythmics class i and iii	T	379101	1297	377804	2.05	0.717523	1.00513	0.286	14.7	0.475315	0.998648
Medication	Cardiac stimulants excl cardiac glycosides	T	379101	304	378797	8.27E-05	-9.40081	196.614	3.60E-172	1.90E+163	0.961865	0.998648
Medication	Vasodilators used in cardiac diseases	T	379101	5385	373716	3.75E-05	-10.1915	69.8385	1.34E-64	1.05E+55	0.883977	0.998648
Medication	Other cardiac preparations	T	379101	50254	328847	0.812	-0.208005	0.266936	0.481	1.37	0.435844	0.998648
Medication	Antiadrenergic agents centrally acting	T	379101	1115	377986	6.65E-05	-9.61791	119.382	1.60E-106	2.77E+97	0.935788	0.998648
Medication	Antiadrenergic agents peripherally acting	T	379101	4727	374374	1.69	0.522233	0.588188	0.532	5.34	0.374612	0.998648
Medication	Antihypertensives and diuretics in combination	T	379101	1142	377959	6.48E-05	-9.64448	119.41	1.47E-106	2.85E+97	0.935627	0.998648
Medication	Low ceiling diuretics thiazides	T	379101	23557	355544	1.04	0.041959	0.349333	0.526	2.07	0.904395	0.998648
Medication	Low ceiling diuretics excl thiazides	T	379101	1449	377652	1.83	0.602175	1.00549	0.254	13.1	0.549247	0.998648
Medication	High ceiling diuretics	T	379101	3930	375171	2.19	0.784943	0.587279	0.693	6.93	0.181361	0.998648
Medication	Potassium sparing agents	T	379101	950	378151	7.25E-05	-9.53198	118.63	7.59E-106	6.92E+96	0.935959	0.998648
Medication	Diuretics and potassium sparing agents in combination	T	379101	27954	351147	1.2	0.181227	0.307636	0.656	2.19	0.555798	0.998648
Medication	Peripheral vasodilators	T	379101	334	378767	8.08E-05	-9.42343	197.871	2.99E-173	2.18E+164	0.962016	0.998648
Medication	Hemorrhoids and anal fissures for topical use	T	379101	11708	367393	0.681	-0.383599	0.58506	0.216	2.14	0.512045	0.998648
Medication	Beta blocking agents	T	379101	27196	351905	0.986	-0.01435	0.332678	0.514	1.89	0.965595	0.998648
Medication	Beta blocking agents and thiazides	T	379101	27196	351905	0.987	-0.01348	0.332651	0.514	1.89	0.967677	0.998648
Medication	Beta blocking agents and other diuretics	T	379101	15555	363546	0.677	-0.390219	0.510779	0.249	1.84	0.444886	0.998648
Medication	Beta blocking agents thiazides and other diuretics	T	379101	15312	363789	0.689	-0.373021	0.510746	0.253	1.87	0.465179	0.998648
Medication	Beta blocking agents other combinations	T	379101	42301	336800	0.863	-0.14694	0.28774	0.491	1.52	0.609582	0.998648
Medication	Selective calcium channel blockers with mainly vascular effects	T	379101	24151	354950	0.747	-0.291183	0.392843	0.346	1.61	0.458559	0.998648
Medication	Selective calcium channel blockers with direct cardiac effects	T	379101	3557	375544	0.77	-0.26119	1.0058	0.107	5.53	0.795107	0.998648
Medication	Calcium channel blockers and diuretics	T	379101	18780	360321	0.988	-0.012457	0.392708	0.457	2.13	0.974695	0.998648
Medication	Ace inhibitors plain	T	379101	38569	340532	0.937	-0.064849	0.286854	0.534	1.64	0.821147	0.998648
Medication	Ace inhibitors combinations	T	379101	54539	324562	0.897	-0.108943	0.253821	0.545	1.47	0.667768	0.998648
Medication	Angiotensin ii antagonists plain	T	379101	16106	362995	1.02	0.020787	0.420369	0.448	2.33	0.960562	0.998648
Medication	Angiotensin ii antagonists combinations	T	379101	30598	348503	0.774	-0.256587	0.349348	0.39	1.53	0.462659	0.998648
Medication	Other agents acting on the renin angiotensin system	T	379101	16224	362877	0.807	-0.214342	0.459578	0.328	1.99	0.640937	0.998648
Medication	Lipid modifying agents plain	T	379101	93186	285915	0.754	-0.282661	0.217421	0.492	1.15	0.193579	0.998648
Medication	Antifungals for topical use	T	379101	3710	375391	1.48	0.39142	0.712688	0.366	5.98	0.582857	0.998648
Medication	Antifungals for systemic use	T	379101	843	378258	3.17	1.15518	1.00444	0.443	22.7	0.250114	0.998648
Medication	Emollients and protectives	T	379101	1288	377813	2.05	0.717535	1.00429	0.286	14.7	0.474939	0.998648
Medication	Cicatrizants	T	379101	2968	376133	6.69E-05	-9.61206	73.0307	4.58E-67	9.78E+57	0.895287	0.998648
Medication	Antipruritics incl antihistamines anesthetics etc	T	379101	592	378509	0.000112	-9.0976	120.598	2.48E-107	5.06E+98	0.939866	0.998648
Medication	Antibiotics for topical use	T	379101	1432	377669	4.81E-05	-9.9427	120.938	5.46E-108	4.23E+98	0.934477	0.998648
Medication	Chemotherapeutics for topical use	T	379101	1020	378081	2.62	0.961398	1.00413	0.365	18.7	0.338341	0.998648
Medication	Corticosteroids combinations with antiseptics	T	379101	6117	372984	1.31	0.27277	0.584076	0.418	4.13	0.640491	0.998648
Medication	Corticosteroids other combinations	T	379101	9688	369413	1.4	0.334665	0.455798	0.572	3.41	0.462802	0.998648
Medication	Antiseptics and disinfectants	T	379101	239	378862	0.000114	-9.0801	197.355	1.16E-172	1.12E+164	0.963303	0.998648
Medication	Other dermatological preparations	T	379101	20932	358169	0.928	-0.075089	0.388495	0.433	1.99	0.846737	0.998648
Medication	Antiinf and antiseptics excl comb with cortico	T	379101	8523	370578	1.3	0.260982	0.507391	0.48	3.51	0.606999	0.998648
Medication	Other gynecologicals	T	379101	52465	326636	0.775	-0.254432	0.26677	0.46	1.31	0.340211	0.998648
Medication	Hormonal contraceptives for systemic use	T	379101	5576	373525	0.61	-0.493905	1.01903	0.0828	4.5	0.627902	0.998648
Medication	Androgens	T	379101	555	378546	4.51E-05	-10.0074	192.89	2.90E-169	7.00E+159	0.958623	0.998648

Medication	Estrogens	T	379101	10090	369011	0.85	-0.162924	0.590095	0.267	2.7	0.782473	0.998648
Medication	Progestogens	T	379101	2010	377091	1.64	0.4943	1.01399	0.225	12	0.625918	0.998648
Medication	Androgens and female sex hormones in combination	T	379101	421	378680	5.85E-05	-9.74627	194.714	1.06E-170	3.24E+161	0.960079	0.998648
Medication	Progestogens and estrogens in combination	T	379101	8666	370435	0.326	-1.11978	1.00927	0.0451	2.36	0.267215	0.998648
Medication	Other sex hormones and modulators of the genital system	T	379101	368	378733	9.92E-05	-9.21842	177.305	1.18E-155	8.35E+146	0.958535	0.998648
Medication	Urologicals	T	379101	6031	373070	3.00E-05	-10.4132	72.4913	5.91E-67	1.53E+57	0.885779	0.998648
Medication	Drugs used in benign prostatic hypertrophy	T	379101	6797	372304	0.359	-1.02343	1.00917	0.0497	2.6	0.310522	0.998648
Medication	Corticosteroids for systemic use plain	T	379101	6943	372158	1.16	0.147556	0.584069	0.369	3.64	0.800551	0.998648
Medication	Thyroid preparations	T	379101	21493	357608	0.915	-0.089022	0.392721	0.424	1.98	0.820673	0.998648
Medication	Antithyroid preparations	T	379101	334	378767	7.78E-05	-9.46088	196.772	2.49E-172	2.44E+163	0.961652	0.998648
Summary	Intestinal infectious diseases (HES)	T	379101	8022	371079	1.37	0.318309	0.507756	0.508	3.72	0.53073	0.998648
Summary	Other bacterial diseases (HES)	T	379101	4393	374708	1.84	0.611209	0.585039	0.585	5.8	0.296147	0.998648
Summary	Infections sexual mode of transmission (HES)	T	379101	253	378848	0.00011	-9.11253	197.003	2.24E-172	5.43E+163	0.963106	0.998648
Summary	Viral infections of the central nervous system (HES)	T	379101	233	378868	9.99E-05	-9.21125	199.143	3.06E-174	3.26E+165	0.963108	0.998648
Summary	Viral infections skin and mucous memb (HES)	T	379101	1422	377679	1.91	0.645779	1.00406	0.267	13.6	0.520115	0.998648
Summary	Viral hepatitis (HES)	T	379101	525	378576	4.81E-05	-9.94293	197.057	8.78E-173	2.63E+163	0.959758	0.998648
Summary	Other viral diseases (HES)	T	379101	1781	377320	1.53	0.426758	1.00391	0.214	11	0.670766	0.998648
Summary	Mycoses (HES)	T	379101	2468	376633	2.22	0.798837	0.712946	0.55	8.99	0.262512	0.998648
Summary	Nutritional anaemias (HES)	T	379101	7757	371344	1.8	0.585498	0.456011	0.735	4.39	0.199158	0.998648
Summary	Haemolytic anaemias (HES)	T	379101	276	378825	9.25E-05	-9.28837	197.535	6.62E-173	1.29E+164	0.962496	0.998648
Summary	Aplastic and other anaemias (HES)	T	379101	11319	367782	1.22	0.198057	0.45613	0.499	2.98	0.664134	0.998648
Summary	Coagulation defects purpura and other haemorrhagic conditions (HES)	T	379101	2426	376675	7.50E-05	-9.49785	73.2987	3.03E-67	1.85E+58	0.896901	0.998648
Summary	Certain disorders involving the immune mechanism (HES)	T	379101	976	378125	6.70E-05	-9.61048	120.765	1.07E-107	4.20E+98	0.936571	0.998648
Summary	Disorders of thyroid gland (HES)	T	379101	15440	363661	0.905	-0.099705	0.459064	0.368	2.23	0.828059	0.998648
Summary	Glucose regulation and pancreatic internal secretion (HES)	T	379101	831	378270	8.33E-05	-9.39351	119.694	1.08E-106	6.40E+97	0.937447	0.998648
Summary	Disorders of other endocrine glands (HES)	T	379101	2354	376747	1.15	0.140199	1.00392	0.161	8.23	0.888935	0.998648
Summary	Other nutritional deficiencies (HES)	T	379101	1249	377852	5.56E-05	-9.79699	120.802	8.25E-108	3.75E+98	0.935363	0.998648
Summary	Obesity and other hyperalimentation (HES)	T	379101	9921	369180	1.1	0.094774	0.507518	0.407	2.97	0.851864	0.998648
Summary	Organic including symptomatic mental disorders (HES)	T	379101	1447	377654	5.05E-05	-9.89428	115.32	3.47E-103	7.33E+93	0.931627	0.998648
Summary	Mental disorders due to psychoactive sub (HES)	T	379101	14024	365077	1.58	0.460355	0.366183	0.773	3.25	0.208691	0.998648
Summary	Schizophrenia schizotypal and delusional disorders (HES)	T	379101	899	378202	7.56E-05	-9.48943	120.336	2.80E-107	2.05E+98	0.937146	0.998648
Summary	Mood affective disorders (HES)	T	379101	11549	367552	0.701	-0.354542	0.583751	0.223	2.2	0.543617	0.998648
Summary	Neurotic stress related and somatoform disorders (HES)	T	379101	6748	372353	0.4	-0.91529	1.00357	0.056	2.86	0.361752	0.998648
Summary	Behav syn assoc with phys disturb and phys fact (HES)	T	379101	424	378677	5.92E-05	-9.73418	197.241	7.54E-173	4.65E+163	0.960639	0.998648
Summary	Disorders of adult personality and behaviour (HES)	T	379101	350	378751	7.12E-05	-9.54961	196.77	2.28E-172	2.22E+163	0.961292	0.998648
Summary	Disorders of psychological development (HES)	T	379101	287	378814	8.05E-05	-9.4269	198.566	7.64E-174	8.49E+164	0.962135	0.998648
Summary	Inflammatory diseases of the central nervous system (HES)	T	379101	536	378565	4.52E-05	-10.0052	199.594	5.71E-175	3.57E+165	0.96002	0.998648
Summary	Atrophies affecting central nervous system (HES)	T	379101	368	378733	6.69E-05	-9.61265	197.447	5.69E-173	7.86E+163	0.961171	0.998648
Summary	Other degenerative diseases of the nervous system (HES)	T	379101	907	378194	2.99	1.0953	1.00654	0.416	21.5	0.276516	0.998648
Summary	Demyelinating diseases of the central nervous system (HES)	T	379101	1406	377695	1.95	0.669318	1.00443	0.273	14	0.505178	0.998648
Summary	Episodic and paroxysmal disorders (HES)	T	379101	12991	366110	1.02	0.019298	0.455708	0.417	2.49	0.966222	0.998648
Summary	Polyneuropathies and peripheral nervous system (HES)	T	379101	1669	377432	3.99E-05	-10.1294	119.826	4.01E-107	3.97E+97	0.932632	0.998648
Summary	Diseases of myoneural junction and muscle (HES)	T	379101	482	378619	5.07E-05	-9.88982	199.215	1.35E-174	1.91E+165	0.960406	0.998648
Summary	Cerebral palsy and other paralytic syndromes (HES)	T	379101	2036	377065	2.63	0.967368	0.713073	0.65	10.6	0.174902	0.998648
Summary	Other disorders of the nervous system (HES)	T	379101	3742	375359	4.77E-05	-9.95088	73.4486	1.44E-67	1.58E+58	0.892232	0.998648
Summary	Disorders of eyelid lacrimal system and orbit (HES)	T	379101	8748	370353	0.938	-0.063566	0.584387	0.299	2.95	0.913382	0.998648
Summary	Disorders of conjunctiva (HES)	T	379101	908	378193	7.52E-05	-9.49577	120.518	1.95E-107	2.90E+98	0.937198	0.998648
Summary	Disorders of lens (HES)	T	379101	18482	360619	1.37	0.316731	0.352973	0.687	2.74	0.369546	0.998648
Summary	Disorders of choroid and retina (HES)	T	379101	6755	372346	1.15	0.14139	0.585728	0.365	3.63	0.809252	0.998648
Summary	Glaucoma (HES)	T	379101	4048	375053	1.32	0.276403	0.714777	0.325	5.35	0.69898	0.998648
Summary	Disorders of vitreous body and globe (HES)	T	379101	1565	377536	1.6	0.467832	1.00507	0.223	11.4	0.641593	0.998648
Summary	Ocular musclesocular movement (HES)	T	379101	2672	376429	0.969	-0.031748	1.00412	0.135	6.93	0.974777	0.998648
Summary	Visual disturbances and blindness (HES)	T	379101	3428	375673	0.777	-0.25266	1.00368	0.109	5.55	0.801248	0.998648
Summary	Other disorders of eye and adnexa (HES)	T	379101	876	378225	7.66E-05	-9.47685	120.579	1.76E-107	3.34E+98	0.937355	0.998648
Summary	Diseases of external ear (HES)	T	379101	1324	377777	4.96E-05	-9.91224	120.745	8.22E-108	2.99E+98	0.934573	0.998648
Summary	Diseases of middle ear and mastoid (HES)	T	379101	2903	376198	0.934	-0.067886	1.00366	0.131	6.68	0.946074	0.998648
Summary	Diseases of inner ear (HES)	T	379101	1848	377253	3.69E-05	-10.2077	120.734	6.25E-108	2.18E+98	0.932621	0.998648
Summary	Other disorders of ear (HES)	T	379101	4112	374989	0.642	-0.442865	1.00396	0.0898	4.59	0.659127	0.998648
Summary	Chronic rheumatic heart diseases (HES)	T	379101	1969	377132	3.66E-05	-10.2165	117.166	6.75E-105	1.98E+95	0.930515	0.998648
Summary	Hypertensive diseases (HES)	T	379101	70604	308497	0.922	-0.08116	0.227978	0.59	1.44	0.721841	0.998648
Summary	Ischaemic heart diseases (HES)	T	379101	27652	351449	0.643	-0.442215	0.39494	0.296	1.39	0.262841	0.998648
Summary	Pulmonary heart disease and diseases of pulmonary circulation (HES)	T	379101	3992	375109	2	0.693109	0.585203	0.635	6.3	0.236259	0.998648
Summary	Other forms of heart disease (HES)	T	379101	26042	353059	1.24	0.21531	0.30828	0.678	2.27	0.484912	0.998648
Summary	Diseases of veins lymphatic vessels (HES)	T	379101	35400	343701	1.16	0.144423	0.273377	0.676	1.97	0.597296	0.998648
Summary	Other and unspecified disorders of the circulatory system (HES)	T	379101	4939	374162	1.64	0.494876	0.585308	0.521	5.17	0.397834	0.998648
Summary	Acute upper respiratory infections (HES)	T	379101	2654	376447	6.94E-05	-9.57562	73.4857	1.95E-67	2.48E+58	0.896324	0.998648
Summary	Other diseases of upper respiratory tract (HES)	T	379101	13139	365962	1.44	0.367489	0.388204	0.675	3.09	0.343822	0.998648
Summary	Lung diseases due to external agents (HES)	T	379101	898	378203	2.97	1.08955	1.00702	0.413	21.4	0.279271	0.998648
Summary	Respiratory diseases interstitium (HES)	T	379101	1501	377600	1.81	0.593767	1.00568	0.252	13	0.554914	0.998648
Summary	Diseases of oral cavity salivary glands and jaws (HES)	T	379101	14224	364877	0.561	-0.578788	0.583604	0.179	1.76	0.32132	0.998648
Summary	Diseases of oesophagus stomach and duodenum (HES)	T	379101	55390	323711	1.1	0.098072	0.233605	0.698	1.74	0.674616	0.998648
Summary	Diseases of appendix (HES)	T	379101	3245	375856	1.61	0.473679	0.712504	0.397	6.49	0.506173	0.998648
Summary	Hernia (HES)	T	379101	42709	336392	0.772	-0.25931	0.294257	0.433	1.37	0.378191	0.998648
Summary	Noninfective enteritis and colitis (HES)	T	379101	17546	361555	0.764	-0.269145	0.455589	0.313	1.87	0.554679	0.998648
Summary	Other diseases of intestines (HES)	T	379101	59649	319452	0.739	-0.301971	0.260069	0.444	1.23	0.245594	0.998648
Summary	Diseases of peritoneum (HES)	T	379101	3415	375686	1.61	0.475456	0.712552	0.398	6.5	0.504607	0.998648
Summary	Other diseases of the digestive system (HES)	T	379101	11473	367628	1.17	0.154746	0.455791	0.478	2.85	0.734225	0.998648
Summary	Infections of the skin and subcutaneous tissue (HES)	T	379101	9696	369405	1.37	0.314332	0.456004	0.56	3.35	0.490623	0.998648
Summary	Dermatitis and eczema (HES)	T	379101	2862	376239	1.94	0.660405	0.712521	0.479	7.82	0.354001	0.998648
Summary	Papulosquamous disorders (HES)	T	379101	2604	376497	1.05	0.048407	1.00391	0.147	7.51	0.961542	0.998648
Summary	Urticaria and erythema (HES)	T	379101	948	378153	7.16E-05	-9.54463	120.976	7.55E-108	6.79E+98	0.937115	0.998648
Summary	Radiation related disorders of the skin and subcutaneous tissue (HES)	T	379101	2288	376813	8.22E-05	-9.40647	71.3925	1.39E-65	4.85E+56	0.895176	0.998648
Summary	Disorders of skin appendages (HES)	T	379101	9390	369711	0.869	-0.140353	0.583669	0.277	2.73	0.809969	0.998648
Summary	Other disorders of the skin and subcutaneous tissue (HES)	T	379101	14568	364533	1.12	0.10986	0.417867	0.492	2.53	0.792623	0.998648
Summary	Infectious arthropathies (HES)	T	379101	428	378673	5.50E-05	-9.8081	198.105	1.29E-173	2.35E+164	0.960513	0.998648
Summary	Inflammatory polyarthropathies (HES)	T	379101	17341	361760	0.957	-0.043623	0.419095	0.421	2.18	0.917099	0.998648
Summary	Arthrosis (HES)	T	379101	40307	338794	0.938	-0.064404	0.284985	0.536	1.64	0.821208	0.998648
Summary	Other joint disorders (HES)	T	379101	35549	343552	1.15	0.136524	0.273284	0.671	1.96	0.617379	0.998648
Summary	Spondylopathies (HES)	T	379101	10706	368395	1.03	0.029828	0.508524	0.38	2.79	0.953226	0.998648
Summary	Other dorsopathies (HES)	T	379101	18099	361002	0.891	-0.11512	0.417248	0.393	2.02	0.782622	0.998648
Summary	Disorders of muscles (HES)	T	379101	1025	378076	2.63	0.965184	1.0044	0.367	18.8	0.336575	0.998648
Summary	Disorders of synovium and tendon (HES)	T	379101	8361	370740	0.96	-0.041108	0.583795	0.306	3.01	0.943863	0.998648
Summary	Other soft tissue disorders (HES)	T	379101	25100	354001	1.31	0.273554	0.302245	0.727	2.38	0.365425	0.998648
Summary	Disorders of bone density and structure (HES)	T	379101	8188	370913	1.42	0.350573	0.51095	0.522	3.87	0.492637	0.998648
Summary	Other osteopathies (HES)	T	379101	2866	376235	1.92	0.654918	0.71251	0.476	7.78	0.358006	0.998648
Summary	Chondropathies (HES)	T	379101	1094	378007	2.38	0.867263	1.00417	0.333	17	0.387775	0.998648
Summary	Musculoskeletal system and connective tissue (HES)	T	379101	844	378257	7.95E-05	-9.43992	121.144	6.03E-108	1.05E+99	0.937889	0.998648
Summary	Glomerular diseases (HES)	T	379101	1942	377159	3.50E-05	-10.2592	119.411	7.94E-107	1.55E+97	0.931534	0.998648
Summary	Renal tubulo interstitial diseases (HES)	T	379101	3453	375648	0.769	-0.263111	1.00369	0.107	5.5	0.79321	0.998648
Summary	Renal failure (HES)	T	379101	7743	371358	1.04	0.03733	0.586812	0.329	3.28	0.949276	0.998648
Summary	Urolithiasis (HES)	T	379101	5984	373117	2.95E-05	-10.4308	72.3716	7.35E-67	1.19E+57	0.885399	0.998648
Summary	Other disorders of kidney and ureter (HES)	T	379101	2376	376725	7.70E-05	-9.47121	72.2875	2.26E-66	2.62E+57	0.895758	0.998648
Summary	Other diseases of urinary system (HES)	T	379101	26755	352346	1.13	0.119251	0.315364	0.607	2.09	0.705329	0.998648
Summary	Disorders of breast (HES)	T	379101	4857	374244	0.604	-0.503674	1.00654	0.084	4.35	0.616794	0.998648
Summary	Inflammatory diseases of female pelvic organs (HES)	T	379101	5417	373684	1.65	0.50127	0.591622	0.518	5.26	0.396839	0.998648
Summary	Other disorders of the genitourinary system (HES)	T	379101	1063	378038	6.63E-05	-9.62131	118.654	6.63E-106	6.64E+96	0.935373	0.998648
Summary	Pregnancy with abortive outcome (HES)	T	379101	3363	375738	1.3	0.259559	1.02381	0.174	9.64	0.799864	0.998648
Summary	Oed prot and hyprtn dis in preg chldbrth (HES)	T	379101	1199	377902	4.92E-08	-16.8274	5340.98	0	Inf	0.997486	0.998648
Summary	Other maternal disorders predominantly related to pregnancy (HES)	T	379101	2789	376312	5.89E-08	-16.6478	3227.25	0	Inf	0.995884	0.998648
Summary	Maternal care related to the fetus (HES)	T	379101	6059	373042	2.45E-08	-17.5246	3167.92	0	Inf	0.995586	0.998648
Summary	Complications of labour and delivery (HES)	T	379101	8512	370589	0.47	-0.755685	1.03598	0.0617	3.58	0.465733	0.998648
Summary	Delivery (HES)	T	379101	2450	376651	6.07E-08	-16.6176	3233.63	0	Inf	0.9959	0.998648
Summary	Complications predominantly related to the puerperium (HES)	T	379101	557	378544	1.09E-07	-16.0317	5305.27	0	Inf	0.997589	0.998648
Summary	Other obstetric conditions not elsewhere classified (HES)	T	379101	1624	377477	3.04	1.11077	1.03097	0.403	22.9	0.281302	0.998648
Summary	Congenital malformations of the nervous system (HES)	T	379101	249	378852	0.000106	-9.15587	198.368	1.48E-173	7.55E+164	0.963186	0.998648

Summary	Congenital malformations of eye ear face and neck (HES)	T	379101	307	378794	7.79E-05	-9.46057	198.918	3.71E-174	1.64E+165	0.962067	0.998648
Summary	Congenital malformations of genital organs (HES)	T	379101	628	378473	0.00011	-9.11309	120.445	3.29E-107	3.69E+98	0.939688	0.998648
Summary	Congenital malformations of the urinary system (HES)	T	379101	821	378280	7.89E-05	-9.44687	120.804	1.17E-107	5.34E+98	0.937669	0.998648
Summary	Congenital malformations musculoskeletal system (HES)	T	379101	711	378390	3.73	1.3165	1.00481	0.521	26.7	0.190128	0.998648
Summary	Other congenital malformations (HES)	T	379101	615	378486	0.000108	-9.13309	120.961	1.17E-107	9.95E+98	0.939814	0.998648
Summary	Symptoms circulatory and respiratory systems (HES)	T	379101	45652	333449	1.07	0.070645	0.253542	0.653	1.76	0.780528	0.998648
Summary	Symptoms and signs involving the digestive system and abdomen (HES)	T	379101	52090	327011	0.767	-0.26512	0.273615	0.449	1.31	0.332569	0.998648
Summary	Symptoms and signs involving the urinary system (HES)	T	379101	26525	352576	1.09	0.082935	0.316711	0.584	2.02	0.793426	0.998648
Summary	Symptoms cognition perception emotional state (HES)	T	379101	8138	370963	1.35	0.299731	0.508322	0.498	3.65	0.555427	0.998648
Summary	Symptoms and signs involving speech and voice (HES)	T	379101	2293	376808	2.37	0.860792	0.71316	0.584	9.57	0.227428	0.998648
Summary	General symptoms and signs (HES)	T	379101	41556	337545	1.11	0.107817	0.258863	0.671	1.85	0.677043	0.998648
Summary	Abnormal findings on examination of blood without diagnosis (HES)	T	379101	7295	371806	1.45	0.369622	0.511881	0.531	3.95	0.470242	0.998648
Summary	Abnormal findings of urine without diagnosis (HES)	T	379101	652	378449	0.000106	-9.15415	120.62	2.24E-107	4.99E+98	0.939505	0.998648
Summary	Abn exm of fluids subst wo diag (HES)	T	379101	1857	377244	4.03E-05	-10.1196	118.4	6.62E-106	2.45E+96	0.931888	0.998648
Summary	Abnormal findings on diagnostic imaging (HES)	T	379101	8914	370187	0.924	-0.078864	0.584628	0.294	2.91	0.892694	0.998648
Summary	HESCH Chapter IX Diseases of the circulatory system BIN (HES)	T	379101	117038	262063	0.93	-0.072119	0.190176	0.641	1.35	0.704521	0.998648
Summary	HESCH Diseases of the ear and mastoid process (HES)	T	379101	8809	370292	0.602	-0.506841	0.712051	0.149	2.43	0.476585	0.998648
Summary	HESCH Diseases of the nervous system (HES)	T	379101	36198	342903	0.871	-0.138663	0.301985	0.482	1.57	0.64611	0.998648
Summary	HESCH Mental and behavioural disorders (HES)	T	379101	29106	349995	1.02	0.024552	0.314288	0.554	1.9	0.937733	0.998648
Summary	HESCH Diseases of the musculo and connec tissue (HES)	T	379101	100763	278338	1.26	0.231302	0.183241	0.88	1.8	0.206846	0.998648
Summary	HESCH Diseases of the skin and subcutaneous tissue (HES)	T	379101	37154	341947	1.1	0.09225	0.273504	0.642	1.87	0.735898	0.998648
Summary	HESCH Diseases of the genitourinary system (HES)	T	379101	91318	287783	0.945	-0.056343	0.202953	0.635	1.41	0.781306	0.998648
Summary	HESCH Diseases of the digestive system (HES)	T	379101	137916	241185	1.08	0.074747	0.174499	0.765	1.52	0.668395	0.998648
Summary	HESCH Pregnancy childbirth and the puerperium (HES)	T	379101	12811	366290	0.583	-0.540169	0.75443	0.133	2.56	0.473994	0.998648
Infectious disease	Other bacterial intestinal infections (HES)	T	379101	2315	376786	8.33E-05	-9.39326	72.829	8.46E-67	8.20E+57	0.897376	0.998648
Infectious disease	Viral and other specified intestinal infections (HES)	T	379101	770	378331	9.02E-05	-9.31316	120.69	1.67E-107	4.88E+98	0.938491	0.998648
Infectious disease	Streptococcal septicaemia (HES)	T	379101	235	378866	0.000104	-9.17306	196.103	1.23E-171	8.76E+162	0.962691	0.998648
Infectious disease	Bacterial infection of unspecified site (HES)	T	379101	696	378405	3.9	1.36126	1.00506	0.544	28	0.175608	0.998648
Infectious disease	Herpesviral (HES)	T	379101	232	378869	0.000109	-9.12555	198.698	7.97E-174	1.49E+165	0.963369	0.998648
Infectious disease	Viral warts (HES)	T	379101	643	378458	0.000105	-9.16035	121.121	8.35E-108	1.32E+99	0.939714	0.998648
Infectious disease	Chronic viral hepatitis (HES)	T	379101	343	378758	7.65E-05	-9.47852	195.154	5.82E-171	1.00E+162	0.961263	0.998648
Infectious disease	Viral infection of unspecified site (HES)	T	379101	1590	377511	1.73	0.545373	1.00397	0.241	12.3	0.586982	0.998648
Infectious disease	Dermatophytosis (HES)	T	379101	227	378874	0.000109	-9.12585	195.35	5.64E-171	2.10E+162	0.96274	0.998648
Infectious disease	Streptococcus and staphylococcus (HES)	T	379101	3735	375366	0.69	-0.370845	1.00383	0.0965	4.94	0.711807	0.998648
Infectious disease	Viral agents as the cause of diseases classified to other chapters (HES)	T	379101	505	378596	5.45E-05	-9.81642	194.803	8.26E-171	3.60E+161	0.95981	0.998648
Infectious disease	Helicobacter pylori (HES)	T	379101	748	378353	0.000101	-9.20198	119.157	3.76E-106	2.70E+97	0.938444	0.998648
Haematology	Vitamin B12 deficiency anaemia (HES)	T	379101	809	378292	9.19E-05	-9.29465	119.45	1.93E-106	4.38E+97	0.937978	0.998648
Haematology	Other aplastic anaemias (HES)	T	379101	409	378692	6.32E-05	-9.66951	195.995	9.25E-172	4.31E+162	0.960652	0.998648
Haematology	Other anaemias (HES)	T	379101	10478	368623	1.05	0.046771	0.507939	0.387	2.84	0.926635	0.998648
Haematology	Other coagulation defects (HES)	T	379101	874	378227	7.64E-05	-9.47969	121.137	5.88E-108	9.93E+98	0.937624	0.998648
Haematology	Purpura and other haemorrhagic conditions (HES)	T	379101	1530	377571	4.42E-05	-10.0257	120.326	1.67E-107	1.17E+98	0.933596	0.998648
Haematology	Agranulocytosis (HES)	T	379101	2906	376195	6.39E-05	-9.6585	73.0767	3.99E-67	1.02E+58	0.89485	0.998648
Haematology	Other disorders of white blood cells (HES)	T	379101	309	378792	8.27E-05	-9.40052	197.512	6.19E-173	1.10E+164	0.962039	0.998648
Immuno-inflammation	Sarcoidosis (HES)	T	379101	489	378612	4.91E-05	-9.92227	199.601	6.12E-175	3.93E+165	0.960353	0.998648
Immuno-inflammation	Other disorders involving the immune mechanism (HES)	T	379101	290	378811	8.84E-05	-9.33325	195.357	4.52E-171	1.73E+162	0.961895	0.998648
Metabolic	Other hypothyroidism (HES)	T	379101	12869	366232	0.425	-0.856415	0.714756	0.105	1.72	0.230843	0.998648
Metabolic	Thyrotoxicosis (HES)	T	379101	1663	377438	1.7	0.52781	1.00497	0.236	12.2	0.599443	0.998648
Metabolic	Thyroiditis (HES)	T	379101	275	378826	0.000105	-9.16501	191.43	1.18E-167	9.29E+158	0.961815	0.998648
Metabolic	Other disorders of thyroid (HES)	T	379101	357	378744	7.58E-05	-9.48765	195.033	7.31E-171	7.85E+161	0.961201	0.998648
Metabolic	Insulin dependent diabetes mellitus (HES)	T	379101	2572	376529	1.01	0.012314	1.00411	0.141	7.25	0.990216	0.998648
Metabolic	Non insulin dependent diabetes mellitus (HES)	T	379101	16601	362500	0.462	-0.771861	0.586078	0.147	1.46	0.187841	0.998648
Metabolic	Unspecified diabetes mellitus (HES)	T	379101	2120	376981	1.29	0.253916	1.00534	0.18	9.25	0.800604	0.998648
Metabolic	Other disorders of pancreatic internal secretion (HES)	T	379101	829	378272	8.35E-05	-9.39126	119.701	1.07E-106	6.50E+97	0.937466	0.998648
Metabolic	Hyperparathyroidism of parathyroid gland (HES)	T	379101	752	378349	3.68	1.30232	1.00568	0.512	26.4	0.195335	0.998648
Metabolic	Hyperfunction of pituitary gland (HES)	T	379101	291	378810	8.58E-05	-9.36329	198.996	3.51E-174	2.10E+165	0.962471	0.998648
Metabolic	Hypofunction and other disorders of pituitary gland (HES)	T	379101	359	378742	6.88E-05	-9.5838	198.213	1.30E-173	3.63E+164	0.961437	0.998648
Metabolic	Other disorders of adrenal gland (HES)	T	379101	431	378670	5.83E-05	-9.75059	199.029	2.23E-174	1.52E+165	0.960927	0.998648
Metabolic	Ovarian dysfunction (HES)	T	379101	239	378862	5.87E-08	-16.6504	9398.25	0	Inf	0.998586	0.998648
Metabolic	Deficiency of other B group vitamins (HES)	T	379101	664	378437	0.000108	-9.13663	120.542	2.66E-107	4.36E+98	0.939581	0.998648
Metabolic	Vitamin D deficiency (HES)	T	379101	359	378742	7.42E-05	-9.50887	198.186	1.48E-173	3.71E+164	0.961733	0.998648
Metabolic	Deficiency of other nutrient elements (HES)	T	379101	216	378885	0.000108	-9.13344	198.418	1.37E-173	8.52E+164	0.963285	0.998648
Metabolic	Obesity (HES)	T	379101	9864	369237	1.11	0.100141	0.507522	0.409	2.99	0.843582	0.998648
Metabolic	Disorders of porphyrin and bilirubin metabolism (HES)	T	379101	367	378734	6.66E-05	-9.61673	196.748	2.23E-172	1.99E+163	0.961016	0.998648
Metabolic	Disorders of mineral metabolism (HES)	T	379101	1975	377126	1.38	0.324204	1.0047	0.193	9.91	0.746933	0.998648
Metabolic	Volume depletion (HES)	T	379101	2592	376509	2.13	0.75676	0.713645	0.526	8.63	0.288955	0.998648
Metabolic	Other disorders of fluid electrolyte and acid base balance (HES)	T	379101	4789	374312	1.72	0.543922	0.585776	0.547	5.43	0.353122	0.998648
Metabolic	Postprocedural endocrine and metabolic disorders (HES)	T	379101	1206	377895	2.3	0.834428	1.00548	0.321	16.5	0.406608	0.998648
Mental health	Dementia in Alzheimer s disease (HES)	T	379101	223	378878	5.03E-05	-9.89816	302.275	2.51E-262	1.01E+253	0.973877	0.998648
Mental health	Unspecified dementia (HES)	T	379101	432	378669	6.75E-05	-9.60267	184.899	2.76E-162	1.66E+153	0.958581	0.998648
Mental health	Delirium not induced by alcohol and other psychoactive substances (HES)	T	379101	538	378563	5.08E-05	-9.88789	188.995	6.76E-166	3.82E+156	0.958275	0.998648
Mental health	Other mental disorders due to brain damage (HES)	T	379101	212	378889	0.000119	-9.03281	195.125	9.62E-171	1.48E+162	0.963077	0.998648
Mental health	Mental and behavioural disorders due to use of tobacco (HES)	T	379101	10134	368967	0.802	-0.22039	0.584502	0.255	2.52	0.706132	0.998648
Mental health	Schizophrenia (HES)	T	379101	496	378605	5.04E-05	-9.89507	197.294	5.79E-173	4.39E+163	0.96	0.998648
Mental health	Bipolar affective disorder (HES)	T	379101	888	378213	7.55E-05	-9.4914	121.079	6.51E-108	8.76E+98	0.937518	0.998648
Mental health	Depressive episode (HES)	T	379101	10665	368436	0.763	-0.270875	0.583772	0.243	2.39	0.642641	0.998648
Mental health	Recurrent depressive disorder (HES)	T	379101	679	378422	9.97E-05	-9.21352	121.015	9.74E-108	1.02E+99	0.939312	0.998648
Mental health	Phobic anxiety disorders (HES)	T	379101	527	378574	4.88E-05	-9.92798	198.559	4.69E-174	5.07E+164	0.960122	0.998648
Mental health	Other anxiety disorders (HES)	T	379101	5413	373688	0.504	-0.685279	1.00361	0.0705	3.6	0.494726	0.998648
Mental health	Reaction to severe stress and adjustment disorders (HES)	T	379101	481	378620	5.18E-05	-9.86797	197.286	6.04E-173	4.44E+163	0.960108	0.998648
Mental health	Somatoform disorders (HES)	T	379101	381	378720	6.56E-05	-9.63247	199.301	1.47E-174	2.92E+165	0.961452	0.998648
Mental health	Sexual dysfunction not caused disorder or disease (HES)	T	379101	264	378837	0.000106	-9.1506	185.43	1.53E-162	7.36E+153	0.960642	0.998648
Mental health	Specific personality disorders (HES)	T	379101	293	378808	8.61E-05	-9.36048	195.904	1.51E-171	4.92E+162	0.961891	0.998648
Neurosciences	Spinal muscular atrophy and related syndromes (HES)	T	379101	213	378888	0.000116	-9.05856	195.603	3.68E-171	3.69E+162	0.963062	0.998648
Neurosciences	Dystonia (HES)	T	379101	231	378870	0.000112	-9.09526	198.656	8.92E-174	1.41E+165	0.963483	0.998648
Neurosciences	Other extrapyramidal and movement disorders (HES)	T	379101	562	378539	4.52E-05	-10.0047	197.954	1.42E-173	1.44E+164	0.959692	0.998648
Neurosciences	Other degenerative diseases of nervous system (HES)	T	379101	567	378534	4.53E-05	-10.002	194.108	2.68E-170	7.66E+160	0.958905	0.998648
Neurosciences	Multiple sclerosis (HES)	T	379101	1215	377886	2.27	0.818044	1.00463	0.316	16.2	0.415487	0.998648
Neurosciences	Other demyelinating diseases of CNS (HES)	T	379101	283	378818	8.99E-05	-9.31699	198.27	1.52E-173	5.30E+164	0.96252	0.998648
Neurosciences	Epilepsy (HES)	T	379101	3387	375714	0.792	-0.23279	1.00363	0.111	5.67	0.816577	0.998648
Neurosciences	Migraine (HES)	T	379101	2516	376585	2.23	0.800667	0.713573	0.55	9.02	0.26184	0.998648
Neurosciences	Other headache syndromes (HES)	T	379101	459	378642	5.55E-05	-9.79866	199.293	1.27E-174	2.43E+165	0.960786	0.998648
Neurosciences	Transient cerebral ischaemic attacks and related syndromes (HES)	T	379101	2348	376753	1.15	0.140922	1.00574	0.16	8.27	0.888566	0.998648
Neurosciences	Sleep disorders (HES)	T	379101	4841	374260	0.525	-0.644634	1.00427	0.0733	3.76	0.520944	0.998648
Neurosciences	Disorders of trigeminal nerve (HES)	T	379101	419	378682	6.22E-05	-9.68576	197.848	2.41E-173	1.60E+164	0.960955	0.998648
Neurosciences	Facial nerve disorders (HES)	T	379101	777	378324	8.45E-05	-9.37877	120.828	1.19E-107	5.99E+98	0.938129	0.998648
Neurosciences	Nerve root and plexus disorders (HES)	T	379101	316	378785	7.74E-05	-9.46712	199.728	7.53E-175	7.95E+165	0.962194	0.998648
Neurosciences	Nerve root and plexus compressions (HES)	T	379101	3581	375520	0.724	-0.323087	1.00356	0.101	5.18	0.747498	0.998648
Neurosciences	Mononeuropathies of upper limb (HES)	T	379101	9665	369436	0.273	-1.29945	1.00385	0.0381	1.95	0.195506	0.998648
Neurosciences	Mononeuropathies of lower limb (HES)	T	379101	1479	377622	4.98E-05	-9.90709	118.589	5.65E-106	4.39E+96	0.933421	0.998648
Neurosciences	Other mononeuropathies (HES)	T	379101	306	378795	8.22E-05	-9.40617	199.146	2.50E-174	2.70E+165	0.962328	0.998648
Neurosciences	Mononeuropathy in diseases classified elsewhere (HES)	T	379101	205	378896	0.000126	-8.98302	193.903	1.11E-169	1.42E+161	0.963049	0.998648
Neurosciences	Inflammatory polyneuropathy (HES)	T	379101	268	378833	9.42E-05	-9.27015	198.155	2.00E-173	4.44E+164	0.962687	0.998648
Neurosciences	Other polyneuropathies (HES)	T	379101	1097	378004	6.11E-05	-9.70346	119.588	9.78E-107	3.81E+97	0.93533	0.998648
Neurosciences	Polyneuropathy in diseases classified elsewhere (HES)	T	379101	353	378748	7.02E-05	-9.56419	195.663	1.97E-171	2.50E+162	0.961014	0.998648
Neurosciences	Paraplegia and tetraplegia (HES)	T	379101	297	378804	8.17E-05	-9.41268	197.625	4.90E-173	1.36E+164	0.962012	0.998648
Neurosciences	Other paralytic syndromes (HES)	T	379101	334	378767	7.37E-05	-9.51549	199.266	1.77E-174	3.06E+165	0.961913	0.998648
Neurosciences	Disorders of autonomic nervous system (HES)	T	379101	241	378860	0.000107	-9.14177	197.094	1.82E-172	6.30E+163	0.963005	0.998648
Neurosciences	Hydrocephalus (HES)	T	379101	402	378699	6.11E-05	-9.70314	199.277	1.44E-174	2.59E+165	0.961165	0.998648
Neurosciences	Other disorders of brain (HES)	T	379101	1438	377663	4.59E-05	-9.9882	121.177	3.27E-108	6.46E+98	0.934308	0.998648
Neurosciences	Other diseases of spinal cord (HES)	T	379101	528	378573	4.69E-05	-9.96647	198.088	1.14E-173	1.94E+164	0.959873	0.998648
Neurosciences	Other disorders of CNS (HES)	T	379101	223	378878	0.00011	-9.11906	199.367	2.16E-174	5.55E+165	0.963517	0.998648
Neurosciences	Other nervous system in diseases classified elsewhere (HES)	T	379101	960	378141	6.96E-05	-9.57236	120.513	1.82E-107	2.66E+98	0.936691	0.998648
Eye	Other inflammation of eyelid (HES)	T	379101	538	378563	4.62E-05	-9.98253	197.648	2.65E-173	8.05E+163	0.959719	0.998648
Eye	Other disorders of eyelid (HES)	T	379101	5168	373933	1.06	0.061289	0.71321	0.263	4.3	0.931518	0.998648
Eye	Disorders of lacrimal system (HES)	T	379101	1950	377151	3.79E-05	-10.1801	118.683	3.58E-106	4.02E+96	0.931645	0.998648

Eye	Disorders of orbit (HES)	T	379101	250	378851	9.87E-05	-9.22348	199.46	1.62E-174	6.00E+165	0.963117	0.998648
Eye	Conjunctivitis (HES)	T	379101	221	378880	0.000116	-9.0599	198.225	2.15E-173	6.28E+164	0.963545	0.998648
Eye	Other disorders of conjunctiva (HES)	T	379101	672	378429	0.000101	-9.19945	120.482	2.81E-107	3.64E+98	0.939136	0.998648
Eye	Keratitis (HES)	T	379101	236	378865	0.000103	-9.18234	199.145	3.14E-174	3.37E+165	0.963223	0.998648
Eye	Other disorders of iris and ciliary body (HES)	T	379101	272	378829	8.61E-05	-9.36047	197.285	1.01E-172	7.37E+163	0.962158	0.998648
Eye	Senile cataract (HES)	T	379101	7529	371572	1.47	0.387418	0.513961	0.538	4.03	0.450976	0.998648
Eye	Other cataract (HES)	T	379101	13094	366007	1.27	0.239654	0.423606	0.554	2.92	0.571566	0.998648
Eye	Other disorders of lens (HES)	T	379101	203	378898	0.000118	-9.04761	197.657	6.63E-173	2.09E+164	0.96349	0.998648
Eye	Retinal detachments and breaks (HES)	T	379101	2997	376104	0.821	-0.197786	1.00462	0.115	5.88	0.843925	0.998648
Eye	Other retinal disorders (HES)	T	379101	3106	375995	1.74	0.553908	0.715095	0.428	7.07	0.438579	0.998648
Eye	Retinal disorders in diseases classified elsewhere (HES)	T	379101	1229	377872	5.47E-05	-9.81361	118.642	5.60E-106	5.35E+96	0.934077	0.998648
Eye	Glaucoma (HES)	T	379101	4047	375054	1.32	0.276751	0.714777	0.325	5.35	0.698619	0.998648
Eye	Disorders of vitreous body (HES)	T	379101	1246	377855	2.03	0.709123	1.00524	0.283	14.6	0.480546	0.998648
Eye	Disorders of globe (HES)	T	379101	389	378712	5.79E-05	-9.7576	196.481	3.27E-172	1.02E+163	0.960392	0.998648
Eye	Other disorders of optic (HES)	T	379101	285	378816	8.43E-05	-9.38099	197.893	2.99E-173	2.38E+164	0.962191	0.998648
Eye	Paralytic strabismus (HES)	T	379101	258	378843	9.18E-05	-9.2964	198.58	8.47E-174	9.94E+164	0.962661	0.998648
Eye	Other strabismus (HES)	T	379101	823	378278	8.16E-05	-9.4142	121.081	7.00E-108	9.50E+98	0.938026	0.998648
Eye	Disorders of refraction and accommodation (HES)	T	379101	1639	377462	1.56	0.446886	1.00489	0.218	11.2	0.656527	0.998648
Eye	Visual disturbances (HES)	T	379101	2695	376406	6.72E-05	-9.60795	73.3706	2.36E-67	1.91E+58	0.895814	0.998648
Eye	Blindness and low vision (HES)	T	379101	805	378296	3.34	1.20596	1.00467	0.466	23.9	0.230005	0.998648
Eye	Other disorders of eye and adnexa (HES)	T	379101	430	378671	5.93E-05	-9.7332	199.203	1.61E-174	2.18E+165	0.96103	0.998648
ENT	Otitis externa (HES)	T	379101	488	378613	5.04E-05	-9.89489	199.397	9.39E-175	2.71E+165	0.960422	0.998648
ENT	Other disorders of external ear (HES)	T	379101	883	378218	7.35E-05	-9.51802	120.327	2.77E-107	1.95E+98	0.936952	0.998648
ENT	Nonsuppurative otitis media (HES)	T	379101	959	378142	2.77	1.01845	1.00432	0.387	19.8	0.310552	0.998648
ENT	Suppurative and unspecified otitis media (HES)	T	379101	700	378401	9.91E-05	-9.2196	120.776	1.55E-107	6.35E+98	0.939152	0.998648
ENT	Cholesteatoma of middle ear (HES)	T	379101	393	378708	6.22E-05	-9.68453	199.242	1.57E-174	2.47E+165	0.961233	0.998648
ENT	Perforation of tympanic membrane (HES)	T	379101	870	378231	7.91E-05	-9.445	120.935	9.04E-108	6.92E+98	0.937749	0.998648
ENT	Other disorders of tympanic membrane (HES)	T	379101	333	378768	7.34E-05	-9.51901	199.383	1.41E-174	3.84E+165	0.961922	0.998648
ENT	Other disorders of middle ear and mastoid (HES)	T	379101	400	378701	6.26E-05	-9.67887	199.298	1.41E-174	2.77E+165	0.961266	0.998648
ENT	Otosclerosis (HES)	T	379101	275	378826	8.65E-05	-9.35548	199.283	2.01E-174	3.72E+165	0.962556	0.998648
ENT	Disorders of vestibular function (HES)	T	379101	871	378230	8.10E-05	-9.42128	120.121	4.56E-107	1.44E+98	0.937485	0.998648
ENT	Other diseases of inner ear (HES)	T	379101	734	378367	9.29E-05	-9.28422	120.813	1.35E-107	6.40E+98	0.938745	0.998648
ENT	Conductive and sensorineural hearing loss (HES)	T	379101	866	378235	7.70E-05	-9.4714	120.816	1.11E-107	5.34E+98	0.937514	0.998648
ENT	Other hearing loss (HES)	T	379101	2563	376538	1.02	0.02443	1.00469	0.143	7.34	0.980601	0.998648
ENT	Otalgia and effusion of ear (HES)	T	379101	479	378622	5.25E-05	-9.85486	199.157	1.56E-174	1.76E+165	0.960534	0.998648
ENT	Other disorders of ear (HES)	T	379101	566	378535	4.44E-05	-10.0232	198.834	2.49E-174	7.90E+164	0.959796	0.998648
Cardiovascular	Rheumatic mitral valve diseases (HES)	T	379101	286	378815	9.87E-05	-9.22372	192.592	1.14E-168	8.55E+159	0.961802	0.998648
Cardiovascular	Rheumatic tricuspid valve diseases (HES)	T	379101	262	378839	9.96E-05	-9.21442	195.011	1.00E-170	9.88E+161	0.962313	0.998648
Cardiovascular	Multiple valve diseases (HES)	T	379101	1492	377609	4.83E-05	-9.93741	116.909	1.48E-104	1.58E+95	0.93226	0.998648
Cardiovascular	Essential primary hypertension (HES)	T	379101	70382	308719	0.926	-0.076894	0.227978	0.592	1.45	0.735902	0.998648
Cardiovascular	Hypertensive renal disease (HES)	T	379101	1396	377705	4.99E-05	-9.90512	117.597	3.96E-105	6.29E+95	0.932874	0.998648
Cardiovascular	Acute myocardial infarction (HES)	T	379101	7305	371796	0.348	-1.05449	1.00602	0.0485	2.5	0.294559	0.998648
Cardiovascular	Subsequent myocardial infarction (HES)	T	379101	663	378438	4.03E-05	-10.1188	187.227	1.72E-164	9.47E+154	0.956899	0.998648
Cardiovascular	Other acute ischaemic heart diseases (HES)	T	379101	1278	377823	5.55E-05	-9.79983	116.337	5.20E-104	5.92E+94	0.932869	0.998648
Cardiovascular	Chronic ischaemic heart disease (HES)	T	379101	22951	356150	0.543	-0.610109	0.462592	0.219	1.35	0.187205	0.998648
Cardiovascular	Pulmonary embolism (HES)	T	379101	3296	375805	1.6	0.471255	0.713334	0.396	6.48	0.508845	0.998648
Cardiovascular	Other pulmonary heart diseases (HES)	T	379101	745	378356	3.59	1.27775	1.00631	0.499	25.8	0.204178	0.998648
Cardiovascular	Other diseases of pericardium (HES)	T	379101	973	378128	2.69	0.9886	1.00513	0.375	19.3	0.325333	0.998648
Cardiovascular	Nonrheumatic aortic valve disorders (HES)	T	379101	2314	376787	1.14	0.12939	1.00642	0.158	8.18	0.897702	0.998648
Cardiovascular	Cardiomyopathy (HES)	T	379101	1163	377938	5.62E-05	-9.78609	118.97	3.02E-106	1.05E+97	0.934443	0.998648
Cardiovascular	Atrioventricular and left bundle branch block (HES)	T	379101	3286	375815	0.79	-0.236193	1.00611	0.11	5.67	0.814397	0.998648
Cardiovascular	Other conduction disorders (HES)	T	379101	1981	377120	2.62	0.962985	0.715097	0.645	10.6	0.178093	0.998648
Cardiovascular	Cardiac arrest (HES)	T	379101	863	378238	3.07	1.12288	1.00642	0.428	22.1	0.264543	0.998648
Cardiovascular	Paroxysmal tachycardia (HES)	T	379101	2977	376124	1.79	0.580289	0.713112	0.442	7.23	0.415793	0.998648
Cardiovascular	Atrial fibrillation and flutter (HES)	T	379101	13484	365617	1.61	0.47339	0.372336	0.774	3.33	0.203585	0.998648
Cardiovascular	Other cardiac arrhythmias (HES)	T	379101	2381	376720	7.84E-05	-9.45305	72.2306	2.57E-66	2.39E+57	0.895876	0.998648
Cardiovascular	Heart failure (HES)	T	379101	4854	374247	3.99E-05	-10.1296	69.9549	1.13E-64	1.40E+55	0.884868	0.998648
Cardiovascular	Complications and ill defined descriptions of heart disease (HES)	T	379101	3773	375328	1.41	0.346807	0.715589	0.348	5.75	0.627928	0.998648
Cardiovascular	Intracerebral haemorrhage (HES)	T	379101	660	378441	0.000104	-9.17099	118.276	2.18E-105	4.96E+96	0.938195	0.998648
Cardiovascular	Other nontraumatic intracranial haemorrhage (HES)	T	379101	336	378765	7.44E-05	-9.50572	193.041	3.56E-169	1.55E+160	0.960726	0.998648
Cardiovascular	Cerebral infarction (HES)	T	379101	2917	376184	1.85	0.61645	0.715023	0.456	7.52	0.38861	0.998648
Cardiovascular	Stroke not specified as haemorrhage or infarction (HES)	T	379101	968	378133	7.30E-05	-9.52544	117.354	9.32E-105	5.71E+95	0.935308	0.998648
Cardiovascular	Occlusion and stenosis not cerebral infarction (HES)	T	379101	928	378173	8.00E-05	-9.43314	115.013	1.01E-102	6.37E+93	0.934632	0.998648
Cardiovascular	Sequelae of cerebrovascular disease (HES)	T	379101	1161	377940	2.3	0.831502	1.00533	0.32	16.5	0.408185	0.998648
Cardiovascular	Atherosclerosis (HES)	T	379101	1127	377974	6.43E-05	-9.65269	115.032	7.77E-103	5.31E+93	0.933125	0.998648
Cardiovascular	Arterial embolism and thrombosis (HES)	T	379101	839	378262	8.70E-05	-9.3491	116.593	4.94E-104	1.53E+95	0.936089	0.998648
Cardiovascular	Other disorders of arteries and arterioles (HES)	T	379101	1296	377805	5.61E-05	-9.78899	116.8	2.12E-104	1.48E+95	0.933207	0.998648
Cardiovascular	Diseases of capillaries (HES)	T	379101	604	378497	0.000116	-9.05851	120.19	5.73E-107	2.36E+98	0.939921	0.998648
Cardiovascular	Phlebitis and thrombophlebitis (HES)	T	379101	3502	375599	1.5	0.408723	0.713135	0.372	6.09	0.566553	0.998648
Cardiovascular	Other venous embolism and thrombosis (HES)	T	379101	367	378734	6.64E-05	-9.6198	199.511	9.89E-175	4.46E+165	0.961543	0.998648
Cardiovascular	Varicose veins of lower extremities (HES)	T	379101	9985	369116	1.12	0.116379	0.507881	0.415	3.04	0.818756	0.998648
Cardiovascular	Haemorrhoids (HES)	T	379101	21509	357592	1.25	0.226939	0.328284	0.659	2.39	0.489384	0.998648
Digestive system	Oesophageal varices (HES)	T	379101	488	378613	5.18E-05	-9.86818	195.946	8.35E-172	3.21E+162	0.959834	0.998648
Cardiovascular	Varicose veins of other sites (HES)	T	379101	460	378641	5.64E-05	-9.78321	193.824	5.82E-170	5.46E+160	0.959744	0.998648
Cardiovascular	Other lymphatic vessels and lymph nodes (HES)	T	379101	601	378500	0.00011	-9.11425	120.872	1.42E-107	8.51E+98	0.939893	0.998648
Cardiovascular	Hypotension (HES)	T	379101	4415	374686	1.85	0.612906	0.585389	0.586	5.81	0.295097	0.998648
Cardiovascular	Disorders of circulatory system in disease (HES)	T	379101	256	378845	9.88E-05	-9.22199	193.677	1.36E-169	7.18E+160	0.962023	0.998648
ENT	Acute nasopharyngitis (HES)	T	379101	356	378745	6.95E-05	-9.57406	199.128	2.19E-174	2.20E+165	0.961652	0.998648
ENT	Acute pharyngitis (HES)	T	379101	757	378344	8.98E-05	-9.31841	121.151	6.72E-108	1.20E+99	0.938691	0.998648
ENT	Acute tonsillitis (HES)	T	379101	512	378589	5.45E-05	-9.81782	193.663	7.71E-170	3.85E+160	0.959568	0.998648
Respiratory	Acute upper respiratory infections (HES)	T	379101	729	378372	9.37E-05	-9.27525	121.053	8.50E-108	1.03E+99	0.938925	0.998648
Respiratory	Pneumonia due to Streptococcus pneumoniae (HES)	T	379101	234	378867	0.000109	-9.12683	197.84	4.28E-173	2.76E+164	0.963205	0.998648
Respiratory	Bacterial pneumonia (HES)	T	379101	433	378668	5.82E-05	-9.75184	196.558	2.83E-172	1.20E+163	0.960431	0.998648
Respiratory	Vasomotor and allergic rhinitis (HES)	T	379101	952	378149	2.79	1.02776	1.00424	0.39	20	0.306111	0.998648
Respiratory	Chronic rhinitis nasopharyngitis and pharyngitis (HES)	T	379101	770	378331	8.90E-05	-9.32704	120.898	1.09E-107	7.24E+98	0.938506	0.998648
Respiratory	Chronic sinusitis (HES)	T	379101	2225	376876	1.21	0.192281	1.00383	0.169	8.67	0.848097	0.998648
Respiratory	Chronic diseases of tonsils and adenoids (HES)	T	379101	904	378197	7.89E-05	-9.44757	119.164	2.90E-106	2.14E+97	0.936808	0.998648
Respiratory	Peritonsillar abscess (HES)	T	379101	376	378725	6.98E-05	-9.56933	195.708	1.80E-171	2.72E+162	0.961002	0.998648
Respiratory	Diseases of vocal cords and larynx (HES)	T	379101	1561	377540	4.51E-05	-10.0074	120.079	2.76E-107	7.36E+97	0.933581	0.998648
Respiratory	Other diseases of upper respiratory tract (HES)	T	379101	650	378451	0.000102	-9.19331	120.38	3.45E-107	3.00E+98	0.939126	0.998648
Respiratory	Bronchitis not specified as acute or chronic (HES)	T	379101	570	378531	4.80E-05	-9.94345	197.297	5.48E-173	4.21E+163	0.959805	0.998648
Respiratory	Unspecified chronic bronchitis (HES)	T	379101	243	378858	0.000121	-9.01596	192.541	1.55E-168	9.52E+159	0.962652	0.998648
Respiratory	Asthma (HES)	T	379101	23838	355263	1.38	0.324136	0.302217	0.765	2.5	0.283483	0.998648
Respiratory	Status asthmaticus (HES)	T	379101	233	378868	0.000111	-9.10334	198.551	1.09E-173	1.14E+165	0.963431	0.998648
Respiratory	Pulmonary oedema (HES)	T	379101	428	378673	6.12E-05	-9.70135	194.672	1.20E-170	3.13E+161	0.960254	0.998648
Respiratory	Other interstitial pulmonary diseases (HES)	T	379101	930	378171	7.73E-05	-9.46781	117.042	1.82E-104	3.28E+95	0.935528	0.998648
Respiratory	Pleural effusion in conditions classified elsewhere (HES)	T	379101	225	378876	5.08E-05	-9.88747	308.037	3.16E-267	8.17E+257	0.974394	0.998648
Respiratory	Other pleural conditions (HES)	T	379101	242	378859	9.89E-05	-9.22166	193.455	2.10E-169	4.65E+160	0.961981	0.998648
Respiratory	Postprocedural respiratory disorders (HES)	T	379101	334	378767	7.69E-05	-9.473	195.233	5.01E-171	1.18E+162	0.961301	0.998648
Digestive system	Embedded and impacted teeth (HES)	T	379101	1708	377393	3.96E-05	-10.1361	119.253	1.22E-106	1.28E+97	0.932264	0.998648
Digestive system	Dental caries (HES)	T	379101	2856	376245	6.15E-05	-9.69688	73.3339	2.32E-67	1.63E+58	0.894803	0.998648
Digestive system	Diseases of pulp and periapical tissues (HES)	T	379101	2104	376997	1.33	0.285341	1.00394	0.186	9.52	0.776241	0.998648
Digestive system	Gingivitis and periodontal diseases (HES)	T	379101	766	378335	8.62E-05	-9.3584	120.331	3.22E-107	2.31E+98	0.938009	0.998648
Digestive system	Other disorders of gingiva and edentulous alveolar ridge (HES)	T	379101	283	378818	9.15E-05	-9.29872	198.395	1.21E-173	6.90E+164	0.962617	0.998648
Digestive system	Dentofacial anomalies (HES)	T	379101	538	378563	4.68E-05	-9.97014	197.206	6.38E-173	3.43E+163	0.959678	0.998648
Digestive system	Other disorders of teeth and supporting structures (HES)	T	379101	2410	376691	7.74E-05	-9.46696	73.4023	2.55E-67	2.34E+58	0.897378	0.998648
Digestive system	Cysts of oral region (HES)	T	379101	377	378724	6.18E-05	-9.69101	199.439	1.06E-174	3.61E+165	0.961245	0.998648
Digestive system	Diseases of salivary glands (HES)	T	379101	909	378192	3.08	1.12465	1.00434	0.43	22	0.262803	0.998648
Digestive system	Stomatitis and related lesions (HES)	T	379101	744	378357	9.33E-05	-9.27923	120.979	9.79E-108	8.90E+98	0.938861	0.998648
Digestive system	Other diseases of lip and oral mucosa (HES)	T	379101	2606	376495	1.06	0.061559	1.00399	0.149	7.61	0.951109	0.998648
Digestive system	Diseases of tongue (HES)	T	379101	1106	377995	6.13E-05	-9.70025	120.944	6.88E-108	5.46E+98	0.936075	0.998648
Digestive system	Oesophagitis (HES)	T	379101	8251	370850	0.974	-0.026324	0.584608	0.31	3.06	0.964084	0.998648
Digestive system	Gastro oesophageal reflux disease (HES)	T	379101	20891	358210	0.764	-0.269064	0.417856	0.337	1.73	0.51963	0.998648
Digestive system	Other diseases of oesophagus	T	379101	8938	370163	1.21	0.188056	0.509078	0.445	3.27	0.711826	0.998648
Digestive system	Gastric ulcer (HES)	T	379101	3863	375238	4.92E-05	-9.92012	72.5092	9.35E-67	2.59E+57	0.891179	0.998648

Digestive system	Duodenal ulcer (HES)	T	379101	2818	376283	1.92	0.654745	0.714426	0.474	7.81	0.359425	0.998648
Digestive system	Gastritis and duodenitis (HES)	T	379101	26187	352914	1.07	0.064288	0.329561	0.559	2.03	0.845337	0.998648
Digestive system	Dyspepsia (HES)	T	379101	10739	368362	1.03	0.030031	0.507889	0.381	2.79	0.952849	0.998648
Digestive system	Other diseases of stomach and duodenum (HES)	T	379101	6601	372500	1.27	0.239654	0.58462	0.404	4	0.681857	0.998648
Digestive system	Acute appendicitis (HES)	T	379101	2629	376472	0.986	-0.014198	1.00385	0.138	7.05	0.988715	0.998648
Digestive system	Other diseases of appendix (HES)	T	379101	242	378859	1.00E-04	-9.20969	199.186	2.82E-174	3.55E+165	0.963122	0.998648
Digestive system	Inguinal hernia (HES)	T	379101	14392	364709	1.21	0.18674	0.398329	0.552	2.63	0.639207	0.998648
Digestive system	Femoral hernia (HES)	T	379101	558	378543	4.79E-05	-9.94704	196.59	2.18E-172	1.05E+163	0.959646	0.998648
Digestive system	Umbilical hernia (HES)	T	379101	3437	375664	0.721	-0.326648	1.00477	0.101	5.17	0.745108	0.998648
Digestive system	Ventral hernia (HES)	T	379101	3093	376008	5.86E-05	-9.7448	73.2314	2.70E-67	1.27E+58	0.894139	0.998648
Digestive system	Diaphragmatic hernia (HES)	T	379101	24453	354648	0.658	-0.418091	0.418612	0.29	1.5	0.317913	0.998648
Digestive system	Unspecified abdominal hernia (HES)	T	379101	276	378825	8.96E-05	-9.31964	196.604	3.98E-172	2.02E+163	0.962192	0.998648
Digestive system	Crohn s disease (HES)	T	379101	1547	377554	4.31E-05	-10.0528	121.213	2.85E-108	6.50E+98	0.933903	0.998648
Digestive system	Ulcerative colitis (HES)	T	379101	2940	376161	6.09E-05	-9.70647	73.3589	2.19E-67	1.69E+58	0.894735	0.998648
Digestive system	Other noninfective gastroenteritis and colitis (HES)	T	379101	14793	364308	0.92	-0.083645	0.455709	0.377	2.25	0.854368	0.998648
Digestive system	Vascular disorders of intestine (HES)	T	379101	936	378165	7.47E-05	-9.50222	118.86	4.98E-106	1.12E+97	0.936281	0.998648
Digestive system	Paralytic ileus and intestinal obstruction without hernia (HES)	T	379101	3590	375511	0.762	-0.271332	1.0042	0.107	5.46	0.787008	0.998648
Digestive system	Diverticular disease of intestine (HES)	T	379101	24930	354171	1.11	0.103303	0.331781	0.579	2.12	0.755528	0.998648
Digestive system	Irritable bowel syndrome (HES)	T	379101	5022	374079	1.13	0.119489	0.713248	0.278	4.56	0.866955	0.998648
Digestive system	Other functional intestinal disorders (HES)	T	379101	10172	368929	0.54	-0.616945	0.712208	0.134	2.18	0.386357	0.998648
Digestive system	Fissure and fistula of anal and rectal regions (HES)	T	379101	2863	376238	0.91	-0.094473	1.00401	0.127	6.51	0.925033	0.998648
Digestive system	Abscess of anal and rectal regions (HES)	T	379101	1208	377893	5.57E-05	-9.79516	119.396	1.30E-106	2.39E+97	0.934615	0.998648
Digestive system	Other diseases of intestine (HES)	T	379101	13086	366015	0.816	-0.202868	0.508795	0.301	2.21	0.690097	0.998648
Digestive system	Peritonitis (HES)	T	379101	722	378379	3.77	1.32644	1.00477	0.526	27	0.186788	0.998648
Digestive system	Other disorders of peritoneum (HES)	T	379101	2824	376277	1.96	0.671119	0.712703	0.484	7.91	0.346371	0.998648
Digestive system	Alcoholic liver disease (HES)	T	379101	799	378302	3.36	1.21056	1.0077	0.466	24.2	0.229632	0.998648
Digestive system	Hepatic failure (HES)	T	379101	298	378803	8.56E-05	-9.36573	196.459	5.05E-172	1.45E+163	0.961977	0.998648
Digestive system	Other inflammatory liver diseases (HES)	T	379101	623	378478	0.000112	-9.09595	120.386	3.76E-107	3.34E+98	0.939772	0.998648
Digestive system	Cholecystitis (HES)	T	379101	2442	376659	7.73E-05	-9.46812	73.0198	5.40E-67	1.11E+58	0.896831	0.998648
Digestive system	Other diseases of biliary tract (HES)	T	379101	1752	377349	3.95E-05	-10.139	119.958	3.06E-107	5.09E+97	0.932642	0.998648
Digestive system	Intestinal malabsorption (HES)	T	379101	1888	377213	3.51E-05	-10.2575	120.633	7.25E-108	1.70E+98	0.932237	0.998648
Digestive system	Postprocedural disorders of digestive system (HES)	T	379101	1429	377672	1.85	0.614188	1.0043	0.258	13.2	0.540831	0.998648
Digestive system	Other diseases of digestive system (HES)	T	379101	8428	370673	1.28	0.246447	0.507798	0.473	3.46	0.627446	0.998648
Immuno-inflammation	Cutaneous abscess furuncle and carbuncle (HES)	T	379101	2145	376956	1.25	0.220728	1.00396	0.174	8.92	0.825983	0.998648
Immuno-inflammation	Cellulitis (HES)	T	379101	6570	372531	1.61	0.477069	0.508198	0.595	4.36	0.347861	0.998648
Immuno-inflammation	Pilonidal cyst (HES)	T	379101	452	378649	5.75E-05	-9.7643	192.941	3.35E-169	9.87E+159	0.959638	0.998648
Immuno-inflammation	Other local infections of skin and subcutaneous tissue (HES)	T	379101	1106	377995	6.10E-05	-9.70391	120.457	1.78E-107	2.09E+98	0.935793	0.998648
Immuno-inflammation	Dermatitis due to substances taken internally (HES)	T	379101	436	378665	5.84E-05	-9.74799	198.916	2.79E-174	1.22E+165	0.960915	0.998648
Immuno-inflammation	Other dermatitis (HES)	T	379101	1379	377722	4.91E-05	-9.92178	120.983	5.11E-108	4.72E+98	0.934639	0.998648
Immuno-inflammation	Psoriasis (HES)	T	379101	2058	377043	1.31	0.266626	1.00406	0.182	9.34	0.790587	0.998648
Immuno-inflammation	Lichen planus (HES)	T	379101	501	378600	5.64E-05	-9.78273	196.969	1.22E-172	2.60E+163	0.960388	0.998648
Immuno-inflammation	Urticaria (HES)	T	379101	328	378773	7.69E-05	-9.47304	199.396	1.43E-174	4.12E+165	0.962108	0.998648
Immuno-inflammation	Other erythematous conditions (HES)	T	379101	481	378620	5.28E-05	-9.84945	198.611	4.59E-174	6.07E+164	0.960448	0.998648
Immuno-inflammation	Skin changes due to chronic exposure to nonionizing radiation (HES)	T	379101	2179	376922	8.62E-05	-9.35905	71.3434	1.61E-65	4.61E+56	0.895631	0.998648
Immuno-inflammation	Rosacea (HES)	T	379101	277	378824	9.31E-05	-9.282	197.559	6.36E-173	1.36E+164	0.962527	0.998648
Immuno-inflammation	Follicular cysts of skin and subcutaneous tissue (HES)	T	379101	7583	371518	0.354	-1.03957	1.00351	0.0495	2.53	0.300233	0.998648
Immuno-inflammation	Other follicular disorders (HES)	T	379101	373	378728	7.25E-05	-9.53256	198.233	1.32E-173	3.98E+164	0.961646	0.998648
Immuno-inflammation	Other disorders of pigmentation (HES)	T	379101	706	378395	9.46E-05	-9.26604	120.531	2.39E-107	3.75E+98	0.938722	0.998648
Immuno-inflammation	Seborrhoeic keratosis (HES)	T	379101	2813	376288	0.976	-0.023973	1.00443	0.136	6.99	0.980958	0.998648
Immuno-inflammation	Corns and callosities (HES)	T	379101	257	378844	0.00011	-9.11907	194.93	1.29E-170	9.28E+161	0.962688	0.998648
Immuno-inflammation	Other epidermal thickening (HES)	T	379101	608	378493	4.21E-05	-10.0757	196.764	1.37E-172	1.30E+163	0.95916	0.998648
Immuno-inflammation	Atrophic disorders of skin (HES)	T	379101	2542	376559	1.07	0.066131	1.00424	0.149	7.65	0.947496	0.998648
Immuno-inflammation	Hypertrophic disorders of skin (HES)	T	379101	1003	378098	6.82E-05	-9.59275	120.986	7.06E-108	6.60E+98	0.936803	0.998648
Immuno-inflammation	Other disorders of skin and subcutaneous tissue (HES)	T	379101	5075	374026	1.06	0.062258	0.712557	0.263	4.3	0.930375	0.998648
Musculoskeletal	Pyogenic arthritis (HES)	T	379101	335	378766	7.08E-05	-9.5556	198.119	1.61E-173	3.11E+164	0.961532	0.998648
Musculoskeletal	Seropositive rheumatoid arthritis (HES)	T	379101	529	378572	5.13E-05	-9.87741	195.746	1.22E-171	2.15E+162	0.959756	0.998648
Musculoskeletal	Other rheumatoid arthritis (HES)	T	379101	3989	375112	4.99E-05	-9.90624	72.3742	1.23E-66	2.01E+57	0.891129	0.998648
Musculoskeletal	Gout (HES)	T	379101	2920	376181	1.75	0.560092	0.717425	0.429	7.14	0.43498	0.998648
Musculoskeletal	Other crystal arthropathies (HES)	T	379101	369	378732	6.62E-05	-9.62235	194.898	8.33E-171	5.27E+161	0.960624	0.998648
Musculoskeletal	Other arthritis (HES)	T	379101	10538	368563	0.803	-0.218942	0.585373	0.255	2.53	0.708389	0.998648
Musculoskeletal	Polyarthrosis (HES)	T	379101	3571	375530	0.833	-0.183293	1.00538	0.116	5.97	0.855338	0.998648
Musculoskeletal	Coxarthrosis (HES)	T	379101	10780	368321	1.28	0.24356	0.458933	0.519	3.14	0.595619	0.998648
Musculoskeletal	Gonarthrosis (HES)	T	379101	18217	360884	0.721	-0.327682	0.45696	0.294	1.76	0.473318	0.998648
Musculoskeletal	Arthrosis of first carpometacarpal joint (HES)	T	379101	1179	377922	2.42	0.883509	1.00573	0.337	17.4	0.379685	0.998648
Musculoskeletal	Other arthrosis (HES)	T	379101	14991	364110	0.734	-0.309906	0.509195	0.27	1.99	0.542777	0.998648
Musculoskeletal	Acquired deformities of fingers and toes (HES)	T	379101	8762	370339	0.982	-0.018648	0.587282	0.31	3.1	0.974669	0.998648
Musculoskeletal	Other acquired deformities of limbs (HES)	T	379101	1540	377561	1.72	0.544803	1.00436	0.241	12.3	0.587518	0.998648
Musculoskeletal	Disorders of patella (HES)	T	379101	841	378260	3.03	1.10838	1.00467	0.423	21.7	0.26993	0.998648
Musculoskeletal	Internal derangement of knee (HES)	T	379101	13926	365175	1.31	0.26929	0.388118	0.612	2.8	0.487786	0.998648
Musculoskeletal	Other specific joint derangements (HES)	T	379101	2363	376738	2.22	0.79674	0.712578	0.549	8.97	0.26352	0.998648
Musculoskeletal	Other joint disorders (HES)	T	379101	11928	367173	1.14	0.128521	0.455565	0.466	2.78	0.777856	0.998648
Musculoskeletal	Systemic lupus erythematosus (HES)	T	379101	324	378777	9.35E-05	-9.27806	192.945	5.40E-169	1.62E+160	0.961647	0.998648
Musculoskeletal	Other systemic involvement of connective tissue (HES)	T	379101	1710	377391	4.33E-05	-10.0476	117.454	4.54E-105	4.12E+95	0.931828	0.998648
Musculoskeletal	Scoliosis (HES)	T	379101	942	378159	2.96	1.08655	1.00584	0.413	21.3	0.280032	0.998648
Musculoskeletal	Ankylosing spondylitis (HES)	T	379101	573	378528	4.30E-05	-10.0553	197.173	6.25E-173	2.95E+163	0.959328	0.998648
Musculoskeletal	Other inflammatory spondylopathies (HES)	T	379101	1156	377945	6.43E-05	-9.65244	119.845	6.22E-107	6.64E+97	0.935807	0.998648
Musculoskeletal	Spondylosis (HES)	T	379101	6891	372210	0.813	-0.207452	0.71323	0.201	3.29	0.771157	0.998648
Musculoskeletal	Other spondylopathies (HES)	T	379101	3685	375416	0.724	-0.32256	1.00453	0.101	5.19	0.74813	0.998648
Musculoskeletal	Cervical disc disorders (HES)	T	379101	1357	377744	4.94E-05	-9.91625	121.149	3.71E-108	6.57E+98	0.934765	0.998648
Musculoskeletal	Other intervertebral disc disorders (HES)	T	379101	6634	372467	0.802	-0.221108	0.712239	0.198	3.24	0.756225	0.998648
Musculoskeletal	Other dorsopathies (HES)	T	379101	482	378619	5.36E-05	-9.83425	197.221	7.10E-173	4.05E+163	0.960231	0.998648
Musculoskeletal	Dorsalgia (HES)	T	379101	13010	366091	0.834	-0.181513	0.507375	0.309	2.25	0.720532	0.998648
Musculoskeletal	Myositis (HES)	T	379101	207	378894	0.000119	-9.03912	198.312	1.85E-173	7.60E+164	0.963645	0.998648
Musculoskeletal	Other disorders of muscle (HES)	T	379101	806	378295	3.39	1.21936	1.00461	0.473	24.2	0.224838	0.998648
Musculoskeletal	Synovitis and tenosynovitis (HES)	T	379101	4728	374373	0.561	-0.57823	1.00373	0.0784	4.01	0.56456	0.998648
Musculoskeletal	Spontaneous rupture of synovium and tendon (HES)	T	379101	488	378613	4.87E-05	-9.92906	198.231	8.92E-174	2.66E+164	0.960052	0.998648
Musculoskeletal	Other disorders of synovium and tendon (HES)	T	379101	3447	375654	1.59	0.464338	0.7125	0.394	6.43	0.514593	0.998648
Musculoskeletal	Other bursopathies (HES)	T	379101	729	378372	9.40E-05	-9.272	120.361	3.31E-107	2.67E+98	0.938596	0.998648
Musculoskeletal	Fibroblastic disorders (HES)	T	379101	3613	375488	5.19E-05	-9.86655	70.8475	2.56E-65	1.05E+56	0.889241	0.998648
Musculoskeletal	Shoulder lesions (HES)	T	379101	8336	370765	0.635	-0.454242	0.711889	0.157	2.56	0.523422	0.998648
Musculoskeletal	Enthesopathies of lower limb excluding foot (HES)	T	379101	393	378708	6.31E-05	-9.67147	199.601	7.87E-175	5.05E+165	0.961354	0.998648
Musculoskeletal	Other enthesopathies (HES)	T	379101	1540	377561	1.76	0.564248	1.00415	0.246	12.6	0.574174	0.998648
Musculoskeletal	Osteoporosis with pathological fracture (HES)	T	379101	834	378267	8.60E-05	-9.36104	117.861	4.07E-105	1.82E+96	0.936695	0.998648
Musculoskeletal	Disorders of continuity of bone (HES)	T	379101	1398	377703	4.75E-05	-9.95563	121.154	3.53E-108	6.38E+98	0.934509	0.998648
Musculoskeletal	Other disorders of bone density and structure (HES)	T	379101	998	378103	7.71E-05	-9.47002	118.206	1.85E-105	3.21E+96	0.936146	0.998648
Musculoskeletal	Osteomyelitis (HES)	T	379101	486	378615	5.18E-05	-9.868	197.301	5.87E-173	4.57E+163	0.96011	0.998648
Musculoskeletal	Osteonecrosis (HES)	T	379101	512	378589	4.93E-05	-9.91713	198.889	2.48E-174	9.79E+164	0.960232	0.998648
Musculoskeletal	Paget s disease of bone (HES)	T	379101	243	378858	0.000108	-9.13477	196.032	1.47E-171	7.92E+162	0.962833	0.998648
Musculoskeletal	Osteopathies in diseases classified elsewhere (HES)	T	379101	292	378809	8.53E-05	-9.36889	198.291	1.39E-173	5.25E+164	0.962315	0.998648
Musculoskeletal	Other osteochondropathies (HES)	T	379101	239	378862	9.34E-05	-9.27867	197.391	8.87E-173	9.84E+163	0.962508	0.998648
Musculoskeletal	Other disorders of cartilage (HES)	T	379101	677	378424	9.75E-05	-9.23515	120.947	1.09E-107	8.74E+98	0.939135	0.998648
Musculoskeletal	Acquired deformities of musculoskeletal (HES)	T	379101	329	378772	8.02E-05	-9.43094	197.336	8.48E-173	7.59E+163	0.961883	0.998648
Musculoskeletal	Postprocedural musculoskeletal disorders (HES)	T	379101	412	378689	6.03E-05	-9.71652	199.219	1.59E-174	2.28E+165	0.9611	0.998648
Genitourinary	Recurrent and persistent haematuria (HES)	T	379101	660	378441	0.000104	-9.17489	119.532	1.85E-106	5.80E+97	0.938817	0.998648
Genitourinary	Chronic nephritic syndrome (HES)	T	379101	791	378310	9.14E-05	-9.30057	116.8	3.46E-104	2.42E+95	0.936533	0.998648
Genitourinary	Unspecified nephritic syndrome (HES)	T	379101	244	378857	0.000102	-9.1879	197.338	1.08E-172	9.71E+163	0.962865	0.998648
Genitourinary	Glomerular disorders in diseases classified elsewhere (HES)	T	379101	293	378808	8.50E-05	-9.37305	197.094	1.44E-172	5.00E+163	0.96207	0.998648
Genitourinary	Tubulo interstitial nephritis not specified as acute or chronic (HES)	T	379101	837	378264	8.66E-05	-9.35425	119.91	7.38E-107	1.02E+98	0.93782	0.998648
Genitourinary	Obstructive and reflux uropathy (HES)	T	379101	2412	376689	1.08	0.0781	1.00432	0.151	7.74	0.938016	0.998648
Genitourinary	Acute renal failure (HES)	T	379101	4117	374984	1.98	0.683791	0.587341	0.627	6.27	0.244337	0.998648
Genitourinary	Chronic renal failure (HES)	T	379101	4070	375031	4.74E-05	-9.95752	71.0556	1.56E-65	1.44E+56	0.888552	0.998648
Genitourinary	Unspecified renal failure (HES)	T	379101	1276	377825	2.11	0.744374	1.00582	0.293	15.1	0.459257	0.998648
Genitourinary	Calculus of kidney and ureter (HES)	T	379101	4404	374697	4.04E-05	-10.1157	72.3913	9.68E-67	1.69E+57	0.888868	0.998648
Genitourinary	Calculus of lower urinary tract (HES)	T	379101	726	378375	9.67E-05	-9.24371	115.174	8.86E-103	1.06E+94	0.936032	0.998648
Genitourinary	Unspecified renal colic (HES)	T	379101	1752	377349	3.75E-05	-10.1903	119.622	5.62E-107	2.50E+97	0.932112	0.998648

Genitourinary	Other disorders of kidney and ureter (HES)	T	379101	2178	376923	8.44E-05	-9.3804	72.1904	3.00E-66	2.38E+57	0.896614	0.998648
Genitourinary	Cystitis (HES)	T	379101	2647	376454	1.06	0.060052	1.00453	0.148	7.61	0.95233	0.998648
Genitourinary	Neuromuscular dysfunction of bladder (HES)	T	379101	1508	377593	1.89	0.636192	1.0046	0.264	13.5	0.526552	0.998648
Genitourinary	Other disorders of bladder (HES)	T	379101	8086	371015	1.61	0.476428	0.45924	0.655	3.96	0.299536	0.998648
Genitourinary	Urethral stricture (HES)	T	379101	2967	376134	0.883	-0.124199	1.0044	0.123	6.32	0.901589	0.998648
Genitourinary	Other disorders of urethra (HES)	T	379101	692	378409	9.86E-05	-9.22396	119.861	9.25E-107	1.05E+98	0.938659	0.998648
Genitourinary	Other disorders of urinary system (HES)	T	379101	17515	361586	0.947	-0.054301	0.418736	0.417	2.15	0.896821	0.998648
Genitourinary	Inflammatory diseases of prostate (HES)	T	379101	1436	377665	1.7	0.531593	1.00952	0.235	12.3	0.598485	0.998648
Genitourinary	Other disorders of prostate (HES)	T	379101	1235	377866	5.88E-08	-16.6491	3547.2	0	Inf	0.996255	0.998648
Genitourinary	Hydrocele and spermatocele (HES)	T	379101	1221	377880	5.49E-08	-16.7175	3645.35	0	Inf	0.996341	0.998648
Genitourinary	Orchitis and epididymitis (HES)	T	379101	769	378332	3.18	1.15827	1.00798	0.442	23	0.250513	0.998648
Genitourinary	Redundant prepuce phimosis and paraphimosis (HES)	T	379101	1890	377211	3.53E-08	-17.1592	3654.34	0	Inf	0.996253	0.998648
Genitourinary	Other disorders of penis (HES)	T	379101	1896	377205	3.75E-05	-10.192	110.294	4.89E-99	2.87E+89	0.926374	0.998648
Genitourinary	Benign mammary dysplasia (HES)	T	379101	1624	377477	5.45E-05	-9.81817	110.889	2.22E-99	1.34E+90	0.929447	0.998648
Genitourinary	Inflammatory disorders of breast (HES)	T	379101	430	378671	7.26E-05	-9.52996	187.019	4.65E-164	1.13E+155	0.95936	0.998648
Genitourinary	Hypertrophy of breast (HES)	T	379101	719	378382	0.000106	-9.15208	119.215	3.53E-106	3.19E+97	0.938807	0.998648
Genitourinary	Unspecified lump in breast (HES)	T	379101	1403	377698	6.15E-05	-9.69727	112.239	1.77E-100	2.13E+91	0.93115	0.998648
Genitourinary	Other disorders of breast (HES)	T	379101	1110	377991	2.62	0.964691	1.00767	0.364	18.9	0.33839	0.998648
Genitourinary	Salpingitis and oophoritis (HES)	T	379101	472	378629	6.46E-08	-16.5548	5955.72	0	Inf	0.997782	0.998648
Genitourinary	Inflammatory disease of uterus except cervix (HES)	T	379101	260	378841	1.19E-07	-15.9463	5977.54	0	Inf	0.997871	0.998648
Genitourinary	Inflammatory disease of cervix uteri (HES)	T	379101	1044	378057	7.70E-08	-16.3796	3631.58	0	Inf	0.996401	0.998648
Genitourinary	Other female pelvic inflammatory diseases (HES)	T	379101	2493	376608	2.42	0.883426	0.719734	0.59	9.92	0.21966	0.998648
Genitourinary	Other inflammation of vagina and vulva (HES)	T	379101	690	378411	4.37E-08	-16.9467	6017.17	0	Inf	0.997753	0.998648
Genitourinary	Vulvovaginal ulceration and inflammation (HES)	T	379101	202	378899	5.91E-08	-16.6447	9824.75	0	Inf	0.998648	0.998648
Genitourinary	Endometriosis (HES)	T	379101	3557	375544	6.54E-08	-16.5429	2172.91	0	Inf	0.993926	0.998648
Genitourinary	Female genital prolapse (HES)	T	379101	10411	368690	0.269	-1.31134	1.00816	0.0374	1.94	0.19335	0.998648
Genitourinary	Noninflammatory ovary fallop (HES)	T	379101	4666	374435	4.69E-08	-16.8758	2195.01	0	Inf	0.993866	0.998648
Genitourinary	Polyp of female genital tract (HES)	T	379101	9793	369308	1.49	0.396364	0.464129	0.598	3.69	0.393107	0.998648
Genitourinary	Other noninflammatory disorders of uterus except cervix (HES)	T	379101	4717	374384	4.48E-08	-16.9215	2205.74	0	Inf	0.993879	0.998648
Genitourinary	Erosion and ectropion of cervix uteri (HES)	T	379101	830	378271	1.00E-07	-16.114	3558.13	0	Inf	0.996387	0.998648
Genitourinary	Dysplasia of cervix uteri (HES)	T	379101	1404	377697	5.97E-08	-16.6338	3608.08	0	Inf	0.996322	0.998648
Genitourinary	Other noninflammatory disorders of cervix uteri (HES)	T	379101	1621	377480	1.78	0.577471	1.00785	0.247	12.8	0.566663	0.998648
Genitourinary	Other noninflammatory disorders of vagina (HES)	T	379101	1392	377709	6.08E-05	-9.70856	110.219	9.19E-99	4.02E+89	0.929809	0.998648
Genitourinary	Other noninflammatory disorders of vulva and perineum (HES)	T	379101	1719	377382	1.74	0.556652	1.00758	0.242	12.6	0.580629	0.998648
Genitourinary	Excessive frequent and irregular menstruation (HES)	T	379101	11974	367127	1.02	0.016792	0.520058	0.367	2.82	0.974242	0.998648
Genitourinary	Other abnormal uterine and vaginal bleeding (HES)	T	379101	3736	375365	1.67	0.511886	0.718865	0.408	6.83	0.476418	0.998648
Genitourinary	Pain with female genital (HES)	T	379101	3073	376028	7.53E-08	-16.4015	2164.36	0	Inf	0.993954	0.998648
Genitourinary	Menopausal and other perimenopausal disorders (HES)	T	379101	9413	369688	0.607	-0.498599	0.719649	0.148	2.49	0.488412	0.998648
Genitourinary	Female infertility (HES)	T	379101	923	378178	5.84E-08	-16.6556	5491.54	0	Inf	0.99758	0.998648
Genitourinary	Postprocedural disorders of genitourinary system (HES)	T	379101	1063	378038	6.63E-05	-9.62131	118.654	6.63E-106	6.64E+96	0.935373	0.998648
Gynaecology and Obstetrics	Ectopic pregnancy (HES)	T	379101	264	378837	8.77E-08	-16.2489	8918.05	0	Inf	0.998546	0.998648
Gynaecology and Obstetrics	Other abnormal products of conception (HES)	T	379101	1191	377910	4.67E-05	-9.97171	165.918	2.74E-146	7.97E+136	0.952076	0.998648
Gynaecology and Obstetrics	Spontaneous abortion (HES)	T	379101	1258	377843	1.08E-07	-16.0433	3332.68	0	Inf	0.996159	0.998648
Gynaecology and Obstetrics	Gestational hypertension without significant proteinuria (HES)	T	379101	427	378674	5.07E-08	-16.797	8807.67	0	Inf	0.998478	0.998648
Gynaecology and Obstetrics	Gestational hypertension with significant proteinuria (HES)	T	379101	289	378812	7.46E-08	-16.4105	8877.31	0	Inf	0.998525	0.998648
Gynaecology and Obstetrics	Unspecified maternal hypertension (HES)	T	379101	540	378561	1.10E-07	-16.0232	5337.75	0	Inf	0.997605	0.998648
Gynaecology and Obstetrics	Haemorrhage in early pregnancy (HES)	T	379101	836	378265	7.74E-08	-16.3746	5369.23	0	Inf	0.997567	0.998648
Gynaecology and Obstetrics	Excessive vomiting in pregnancy (HES)	T	379101	202	378899	1.13E-07	-15.9969	8827.78	0	Inf	0.998554	0.998648
Gynaecology and Obstetrics	Infections of genitourinary tract in pregnancy (HES)	T	379101	212	378889	1.24E-07	-15.9038	8726.66	0	Inf	0.998546	0.998648
Gynaecology and Obstetrics	Maternal care for related to pregnancy (HES)	T	379101	1641	377460	1.03E-07	-16.093	3231.98	0	Inf	0.996027	0.998648
Gynaecology and Obstetrics	Multiple gestation (HES)	T	379101	258	378843	8.59E-08	-16.2705	8796.48	0	Inf	0.998524	0.998648
Gynaecology and Obstetrics	Maternal care for known or suspected malpresentation of fetus (HES)	T	379101	990	378111	5.99E-08	-16.6309	5313.48	0	Inf	0.997503	0.998648
Gynaecology and Obstetrics	Maternal care for known or suspected abnormality of pelvic organs (HES)	T	379101	1384	377717	4.13E-08	-17.0026	5338.78	0	Inf	0.997459	0.998648
Gynaecology and Obstetrics	Maternal care fetal abnormality and damage (HES)	T	379101	238	378863	9.18E-08	-16.2035	8892.25	0	Inf	0.998546	0.998648
Gynaecology and Obstetrics	Maternal care for other known or suspected fetal problems (HES)	T	379101	1716	377385	9.51E-08	-16.1682	3220.73	0	Inf	0.995995	0.998648
Gynaecology and Obstetrics	Other disorders of amniotic fluid and membranes (HES)	T	379101	236	378865	8.64E-08	-16.2647	8810.36	0	Inf	0.998527	0.998648
Gynaecology and Obstetrics	Premature rupture of membranes (HES)	T	379101	1011	378090	6.22E-08	-16.5924	5279.74	0	Inf	0.997493	0.998648
Gynaecology and Obstetrics	Placental disorders (HES)	T	379101	225	378876	1.09E-07	-16.0276	8711.78	0	Inf	0.998532	0.998648
Gynaecology and Obstetrics	Antepartum haemorrhage (HES)	T	379101	588	378513	1.00E-07	-16.1178	5324.11	0	Inf	0.997585	0.998648
Gynaecology and Obstetrics	False labour (HES)	T	379101	973	378128	6.47E-08	-16.5538	5290.43	0	Inf	0.997503	0.998648
Gynaecology and Obstetrics	Prolonged pregnancy (HES)	T	379101	1102	377999	5.49E-08	-16.7182	5323.05	0	Inf	0.997494	0.998648
Gynaecology and Obstetrics	Preterm delivery (HES)	T	379101	602	378499	1.04E-07	-16.0817	5350.17	0	Inf	0.997602	0.998648
Gynaecology and Obstetrics	Abnormalities of forces of labour (HES)	T	379101	405	378696	5.80E-08	-16.6636	8759.15	0	Inf	0.998482	0.998648
Gynaecology and Obstetrics	Long labour (HES)	T	379101	1773	377328	9.76E-08	-16.1427	3188.33	0	Inf	0.99596	0.998648
Gynaecology and Obstetrics	Obstructed labour malpresentation of fetus (HES)	T	379101	382	378719	6.38E-08	-16.5678	8800.67	0	Inf	0.998498	0.998648
Gynaecology and Obstetrics	Other obstructed labour (HES)	T	379101	309	378792	7.56E-08	-16.3972	8740.77	0	Inf	0.998503	0.998648
Gynaecology and Obstetrics	Labour and delivery complicated by fetal stress (HES)	T	379101	3031	376070	1.49	0.400899	1.03342	0.197	11.3	0.698065	0.998648
Gynaecology and Obstetrics	Labour and delivery complicated by umbilical cord complications (HES)	T	379101	570	378531	9.17E-08	-16.2042	5413.19	0	Inf	0.997612	0.998648
Gynaecology and Obstetrics	Perineal laceration during delivery (HES)	T	379101	4945	374156	0.896	-0.109482	1.03505	0.118	6.82	0.915761	0.998648
Gynaecology and Obstetrics	Postpartum haemorrhage (HES)	T	379101	1204	377897	5.25E-08	-16.7623	5299.5	0	Inf	0.997476	0.998648
Gynaecology and Obstetrics	Retained placenta and membranes without haemorrhage (HES)	T	379101	203	378898	1.01E-07	-16.1076	8889.16	0	Inf	0.998554	0.998648
Gynaecology and Obstetrics	Other complications of labour and delivery (HES)	T	379101	696	378405	9.05E-08	-16.2179	5336.12	0	Inf	0.997575	0.998648
Gynaecology and Obstetrics	Single spontaneous delivery (HES)	T	379101	1836	377265	8.55E-08	-16.2745	3233.88	0	Inf	0.995985	0.998648
Gynaecology and Obstetrics	Single delivery by caesarean section (HES)	T	379101	504	378597	1.00E-07	-16.116	5393.15	0	Inf	0.997616	0.998648
Gynaecology and Obstetrics	Other maternal diseases complicating pregnancy (HES)	T	379101	1582	377519	3.15	1.14688	1.03119	0.417	23.8	0.266057	0.998648
Genitourinary	Cystic kidney disease (HES)	T	379101	414	378687	5.88E-05	-9.74063	198.465	6.81E-174	5.09E+164	0.960856	0.998648
Genitourinary	Other congenital malformations of kidney (HES)	T	379101	214	378887	0.000111	-9.10409	199.125	3.53E-174	3.51E+165	0.963533	0.998648
Symptoms	Abnormalities of heart beat (HES)	T	379101	7716	371385	0.7	-0.356262	0.712812	0.173	2.83	0.617218	0.998648
Symptoms	Cardiac murmurs and other cardiac sounds (HES)	T	379101	965	378136	7.19E-05	-9.53983	119.924	5.96E-107	8.68E+97	0.936596	0.998648
Symptoms	Abnormal blood pressure reading without diagnosis (HES)	T	379101	2292	376809	8.02E-05	-9.43064	72.7625	9.28E-67	6.93E+57	0.896876	0.998648
Symptoms	Cough (HES)	T	379101	2614	376487	2.13	0.757831	0.712989	0.527	8.63	0.28783	0.998648
Symptoms	Abnormalities of breathing (HES)	T	379101	8413	370688	1.64	0.494835	0.456225	0.671	4.01	0.278085	0.998648
Symptoms	Pain in throat and chest (HES)	T	379101	28963	350138	0.829	-0.187731	0.345183	0.421	1.63	0.586538	0.998648
Symptoms	Symptoms the circulatory and respiratory systems (HES)	T	379101	1000	378101	6.76E-05	-9.60177	120.74	1.13E-107	4.04E+98	0.936616	0.998648
Symptoms	Abdominal and pelvic pain (HES)	T	379101	28298	350803	1.08	0.08056	0.314486	0.585	2.01	0.797824	0.998648
Symptoms	Heartburn (HES)	T	379101	1828	377273	3.96E-05	-10.1362	120.19	1.95E-107	8.05E+97	0.93279	0.998648
Symptoms	Dysphagia (HES)	T	379101	5765	373336	3.22E-05	-10.3445	73.0929	1.95E-67	5.31E+57	0.887455	0.998648
Symptoms	Flatulence and related conditions (HES)	T	379101	1544	377557	1.79	0.583702	1.00414	0.25	12.8	0.56104	0.998648
Symptoms	Faecal incontinence (HES)	T	379101	1634	377467	4.65E-05	-9.97554	118.912	2.80E-106	7.72E+96	0.933144	0.998648
Symptoms	Unspecified jaundice (HES)	T	379101	839	378262	8.10E-05	-9.42076	119.71	1.02E-106	6.42E+97	0.937274	0.998648
Symptoms	Disturbances of skin sensation (HES)	T	379101	2617	376484	2.09	0.736183	0.712477	0.517	8.44	0.301476	0.998648
Symptoms	Localized swelling mass and lump of skin and subcutaneous tissue (HES)	T	379101	1921	377180	1.41	0.342228	1.00391	0.197	10.1	0.733184	0.998648
Symptoms	Abnormal involuntary movements (HES)	T	379101	819	378282	8.15E-05	-9.41477	120.877	1.04E-107	6.36E+98	0.937918	0.998648
Symptoms	Other lack of coordination (HES)	T	379101	322	378779	7.28E-05	-9.52823	198.917	3.47E-174	1.53E+165	0.961796	0.998648
Symptoms	Signs involving the nervous and musculoskeletal systems (HES)	T	379101	2611	376490	1.03	0.026084	1.00429	0.143	7.35	0.979279	0.998648
Symptoms	Pain associated with micturition (HES)	T	379101	1067	378034	6.38E-05	-9.65906	120.736	1.08E-107	3.78E+98	0.936236	0.998648
Symptoms	Unspecified haematuria (HES)	T	379101	14238	364863	0.716	-0.333723	0.508673	0.264	1.94	0.511782	0.998648
Symptoms	Unspecified urinary incontinence (HES)	T	379101	2162	376939	2.68	0.985309	0.713366	0.662	10.8	0.167214	0.998648
Symptoms	Retention of urine (HES)	T	379101	6149	372952	1.23	0.211014	0.588391	0.39	3.91	0.719872	0.998648
Symptoms	Polyuria (HES)	T	379101	3507	375594	0.758	-0.277089	1.00422	0.106	5.43	0.782607	0.998648
Symptoms	Other symptoms and signs involving the urinary system (HES)	T	379101	4602	374499	1.13	0.123256	0.714067	0.279	4.59	0.862957	0.998648
Symptoms	Somnolence stupor and coma (HES)	T	379101	636	378465	0.000106	-9.15591	120.177	5.33E-107	2.09E+98	0.93927	0.998648
Symptoms	Symptoms cognitive functions and awareness (HES)	T	379101	2889	376212	0.929	-0.073333	1.00493	0.13	6.66	0.941827	0.998648
Symptoms	Dizziness and giddiness (HES)	T	379101	4147	374954	1.34	0.295838	0.712836	0.332	5.44	0.678131	0.998648
Symptoms	General sensations and perceptions (HES)	T	379101	472	378629	5.20E-05	-9.86464	198.417	6.61E-174	4.09E+164	0.960348	0.998648
Symptoms	Symptoms and signs involving emotional state (HES)	T	379101	584	378517	4.40E-05	-10.0315	198.632	3.67E-174	5.28E+164	0.959722	0.998648
Symptoms	Speech disturbances (HES)	T	379101	1366	377735	4.89E-05	-9.92652	120.173	2.49E-107	9.60E+97	0.934168	0.998648
Symptoms	Fever of unknown origin (HES)	T	379101	3729	375372	1.42	0.35218	0.712542	0.352	5.75	0.621123	0.998648
Symptoms	Headache (HES)	T	379101	6991	372110	0.388	-0.947811	1.00357	0.0542	2.77	0.344946	0.998648
Symptoms	Pain (HES)	T	379101	1292	377809	5.29E-05	-9.84793	121.029	5.02E-108	5.56E+98	0.935149	0.998648
Symptoms	Malaise and fatigue (HES)	T	379101	2616	376485	1.06	0.062649	1.004	0.149	7.62	0.950245	0.998648
Symptoms	Syncope and collapse (HES)	T	379101	8267	370834	0.318	-1.14565	1.00406	0.0444	2.28	0.253861	0.998648
Symptoms	Shock (HES)	T	379101	252	378849	0.000103	-9.17934	194.93	1.22E-170	8.73E+161	0.962441	0.998648
Symptoms	Enlarged lymph nodes (HES)	T	379101	2200	376901	8.32E-05	-9.39382	73.2464	3.73E-67	1.86E+58	0.897951	0.998648
Symptoms	Oedema (HES)	T	379101	1490	377611	1.8	0.589838	1.00478	0.252	12.9	0.557181	0.998648

Symptoms	Hyperhidrosis (HES)	T	379101	544	378557	4.62E-05	-9.9826	198.325	7.03E-174	3.04E+164	0.959856	0.998648
Symptoms	Symptoms and signs concerning food and fluid intake (HES)	T	379101	5849	373252	0.934	-0.068165	0.712785	0.231	3.78	0.923813	0.998648
Symptoms	Other general symptoms and signs (HES)	T	379101	358	378743	7.45E-05	-9.50447	197.343	7.77E-173	7.15E+163	0.961587	0.998648
Symptoms	Unknown and unspecified causes of morbidity (HES)	T	379101	14557	364544	1.48	0.393145	0.364559	0.725	3.03	0.28085	0.998648
Symptoms	Abnormality of white blood cells (HES)	T	379101	236	378865	0.000109	-9.12305	199.132	3.41E-174	3.49E+165	0.963458	0.998648
Symptoms	Elevated blood glucose level (HES)	T	379101	625	378476	0.000114	-9.07554	119.747	1.34E-106	9.76E+97	0.939587	0.998648
Symptoms	Abnormal serum enzyme levels (HES)	T	379101	226	378875	0.000115	-9.06636	195.043	1.09E-170	1.22E+162	0.962925	0.998648
Symptoms	Other abnormal immunological findings in serum (HES)	T	379101	247	378854	0.000107	-9.1419	188.6	3.09E-165	3.71E+156	0.96134	0.998648
Symptoms	Other abnormal findings of blood chemistry (HES)	T	379101	5815	373286	1.81	0.592584	0.513176	0.662	4.95	0.248198	0.998648
Symptoms	Isolated proteinuria (HES)	T	379101	277	378824	9.17E-05	-9.29735	196.703	3.35E-172	2.51E+163	0.962301	0.998648
Symptoms	Other abnormal findings in urine (HES)	T	379101	297	378804	8.63E-05	-9.35712	197.875	3.17E-173	2.35E+164	0.962284	0.998648
Symptoms	Abnormal findings in specimens from female genital organs (HES)	T	379101	1312	377789	6.32E-08	-16.5773	3623.52	0	Inf	0.99635	0.998648
Symptoms	Abnormal findings on diagnostic imaging of CNS (HES)	T	379101	319	378782	7.99E-05	-9.43512	198.884	4.06E-174	1.57E+165	0.962162	0.998648
Symptoms	Abnormal findings on diagnostic imaging of lung (HES)	T	379101	2263	376838	8.83E-05	-9.33463	71.7457	7.50E-66	1.04E+57	0.896482	0.998648
Symptoms	Abnormal findings on diagnostic imaging of other body structures (HES)	T	379101	2807	376294	0.952	-0.049551	1.00418	0.133	6.81	0.960645	0.998648
Symptoms	Abnormal results of function studies (HES)	T	379101	3857	375244	1.44	0.362982	0.712984	0.355	5.81	0.61068	0.998648
Other	Poisoning by nonopioid analgesics antipyretics and antirheumatics (HES)	T	379101	1567	377534	4.64E-05	-9.97714	119.569	7.72E-107	2.79E+97	0.9335	0.998648
Other	Poisoning antiepileptic hypnotic antiparkinsonism drugs (HES)	T	379101	851	378250	8.19E-05	-9.40957	120.019	5.64E-107	1.19E+98	0.937509	0.998648
Other	Poisoning by psychotropic drugs (HES)	T	379101	1062	378039	7.03E-05	-9.56267	119.001	3.56E-106	1.39E+97	0.935953	0.998648
Other	Poisoning by diuretics and other drugs (HES)	T	379101	335	378766	8.26E-05	-9.40158	196.329	6.29E-172	1.09E+163	0.961806	0.998648
Other	Toxic effect of alcohol (HES)	T	379101	912	378189	8.25E-05	-9.40278	118.85	5.61E-106	1.21E+97	0.936941	0.998648
Medication	Tetracyclines	T	379101	2224	376877	1.24	0.216231	1.0039	0.174	8.88	0.829462	0.998648
Medication	Beta lactam antibacterials penicillins	T	379101	1102	377999	6.03E-05	-9.71545	121.151	4.52E-108	8.06E+98	0.936084	0.998648
Medication	Other beta lactam antibacterials	T	379101	374	378727	7.86E-05	-9.45126	192.94	4.59E-169	1.35E+160	0.960931	0.998648
Medication	Sulfonamides and trimethoprim	T	379101	595	378506	4.47E-05	-10.0159	197.714	2.25E-173	8.87E+163	0.959598	0.998648
Medication	Aminoglycoside antibacterials	T	379101	622	378479	0.000108	-9.13035	120.357	3.84E-107	3.05E+98	0.93953	0.998648
Medication	Combinations of antibacterials	T	379101	595	378506	0.000117	-9.05453	120.95	1.30E-107	1.05E+99	0.940325	0.998648
Medication	Other antibacterials	T	379101	1343	377758	5.26E-05	-9.85374	120.344	1.91E-107	1.44E+98	0.934742	0.998648
Medication	Antimycotics for systemic use	T	379101	389	378712	6.32E-05	-9.66922	198.809	3.72E-174	1.07E+165	0.96121	0.998648
Medication	Direct acting antivirals	T	379101	823	378278	3.04	1.11027	1.00468	0.424	21.7	0.269113	0.998648
Medication	Antimetabolites	T	379101	2405	376696	8.01E-05	-9.43263	72.995	5.87E-67	1.09E+58	0.897181	0.998648
Medication	Other antineoplastic agents	T	379101	1117	377984	6.68E-05	-9.61331	119.682	8.90E-107	5.02E+97	0.93598	0.998648
Medication	Hormones and related agents	T	379101	756	378345	8.99E-05	-9.31647	119.948	7.11E-107	1.14E+98	0.93809	0.998648
Medication	Immunosuppressants	T	379101	3722	375379	0.73	-0.315269	1.00365	0.102	5.22	0.753429	0.998648
Medication	Antiinflammatory antirheumatic agents in combination	T	379101	53678	325423	0.803	-0.219909	0.265981	0.477	1.35	0.408358	0.998648
Medication	Topical products for joint and muscular pain	T	379101	62133	316968	0.779	-0.25024	0.247234	0.48	1.26	0.311463	0.998648
Medication	Muscle relaxants centrally acting agents	T	379101	555	378546	4.51E-05	-10.0074	199.404	8.27E-175	2.45E+165	0.959974	0.998648
Medication	Antigout preparations	T	379101	4574	374527	0.53	-0.634893	1.00766	0.0735	3.82	0.528653	0.998648
Medication	Other drugs for disorders of the musculo skeletal system	T	379101	2679	376422	7.63E-05	-9.48033	71.1345	2.15E-65	2.71E+56	0.893977	0.998648
Medication	Anesthetics general	T	379101	221	378880	0.000111	-9.10356	199.074	3.90E-174	3.17E+165	0.963526	0.998648
Medication	Opioids	T	379101	102262	276839	1.1	0.098727	0.186249	0.766	1.59	0.596055	0.998648
Medication	Other analgesics and antipyretics	T	379101	121055	258046	1.13	0.118501	0.177269	0.795	1.59	0.503826	0.998648
Medication	Antimigraine preparations	T	379101	5449	373652	3.54E-05	-10.2479	71.2128	8.55E-66	1.47E+56	0.885575	0.998648
Medication	Antiepileptics	T	379101	6896	372205	1.18	0.166402	0.583682	0.376	3.71	0.775574	0.998648
Medication	Anticholinergic agents	T	379101	240	378861	0.000104	-9.16664	198.383	1.42E-173	7.69E+164	0.963145	0.998648
Medication	Dopaminergic agents	T	379101	1188	377913	2.32	0.839691	1.00456	0.323	16.6	0.403222	0.998648
Medication	Antipsychotics	T	379101	2920	376181	0.916	-0.08747	1.00362	0.128	6.55	0.930549	0.998648
Medication	Anxiolytics	T	379101	1664	377437	4.11E-05	-10.1	120.939	4.66E-108	3.62E+98	0.933443	0.998648
Medication	Hypnotics and sedatives	T	379101	3007	376094	1.85	0.614547	0.713172	0.457	7.48	0.388848	0.998648
Medication	Antidepressants	T	379101	29214	349887	1.04	0.04014	0.315019	0.561	1.93	0.898607	0.998648
Medication	Psycholeptics and psychoanaleptics in combination	T	379101	12227	366874	0.445	-0.810005	0.712283	0.11	1.8	0.255456	0.998648
Medication	Anti dementia drugs	T	379101	2265	376836	8.78E-05	-9.34078	72.0914	3.78E-66	2.04E+57	0.896908	0.998648
Medication	Drugs used in addictive disorders	T	379101	648	378453	0.000109	-9.12763	120.538	2.70E-107	4.36E+98	0.939638	0.998648
Medication	Antivertigo preparations	T	379101	1504	377597	4.89E-05	-9.92596	119.359	1.23E-106	1.95E+97	0.933724	0.998648
Medication	Agents against amoebiasis and other protozoal diseases	T	379101	660	378441	0.000105	-9.15705	120.877	1.35E-107	8.24E+98	0.939614	0.998648
Medication	Antimalarials	T	379101	3446	375655	5.86E-05	-9.74508	71.8869	3.77E-66	9.10E+56	0.892168	0.998648
Medication	Throat preparations	T	379101	49826	329275	0.821	-0.197469	0.267077	0.486	1.39	0.459682	0.998648
Medication	Expectorants excl combinations with cough suppressants	T	379101	1117	377984	6.91E-05	-9.58016	116.62	3.72E-104	1.28E+95	0.934529	0.998648
Medication	Cough suppressants excl combinations with expectorants	T	379101	2402	376699	8.12E-05	-9.41915	72.9549	6.44E-67	1.02E+58	0.897271	0.998648
Medication	Antihistamines for systemic use	T	379101	13321	365780	1.2	0.18222	0.417437	0.529	2.72	0.662459	0.998648
Medication	Antiinfectives	T	379101	4005	375096	2.06	0.722084	0.583841	0.656	6.47	0.216168	0.998648
Medication	Antiinflammatory agents	T	379101	18165	360936	0.884	-0.122833	0.41773	0.39	2.01	0.768721	0.998648
Medication	Antiinflammatory agents and antiinfectives in combination	T	379101	17347	361754	0.767	-0.265364	0.455816	0.314	1.87	0.56045	0.998648
Medication	Antiglaucoma preparations and miotics	T	379101	4850	374251	1.11	0.106394	0.71396	0.274	4.51	0.881538	0.998648
Medication	Mydriatics and cycloplegics	T	379101	287	378814	9.16E-05	-9.29771	198.607	8.02E-174	1.05E+165	0.962661	0.998648
Medication	Decongestants and antiallergics	T	379101	420	378681	6.08E-05	-9.70738	198.98	2.56E-174	1.44E+165	0.96109	0.998648
Medication	Surgical aids	T	379101	1057	378044	7.63E-05	-9.48039	116.704	3.49E-104	1.67E+95	0.935256	0.998648
Medication	Chapter p Chapter X Diseases of the respiratory system BIN (HES)	T	379101	9049	370052	1.57	0.448144	0.456081	0.64	3.83	0.325806	0.998648
Medication	Antiinfectives	T	379101	1041	378060	6.66E-05	-9.61644	120.822	9.50E-108	4.67E+98	0.936562	0.998648
Medication	Corticosteroids	T	379101	6270	372831	1.28	0.245137	0.58406	0.407	4.01	0.674695	0.998648
Medication	Corticosteroids and antiinfectives in combination 1	T	379101	5806	373295	1.39	0.329638	0.584049	0.443	4.37	0.572481	0.998648
Medication	Antiinfectives	T	379101	956	378145	7.32E-05	-9.52206	120.717	1.28E-107	4.18E+98	0.937129	0.998648
Medication	Corticosteroids	T	379101	4111	374990	1.31	0.268545	0.712821	0.323	5.29	0.70637	0.998648
Medication	Corticosteroids and antiinfectives in combination 2	T	379101	6221	372880	1.29	0.254503	0.584071	0.411	4.05	0.663026	0.998648
Medication	All other therapeutic products	T	379101	3557	375544	1.57	0.448109	0.713057	0.387	6.33	0.52972	0.998648
Medication	Other diagnostic agents	T	379101	392	378709	6.46E-05	-9.6476	198.439	7.86E-174	5.31E+164	0.961224	0.998648
Medication	Magnetic resonance imaging contrast media	T	379101	1522	377579	4.71E-05	-9.96416	119.298	1.33E-106	1.66E+97	0.933435	0.998648
Medication	Hepatic and reticulo endothelial system	T	379101	1728	377373	1.64	0.496679	1.00457	0.229	11.8	0.621011	0.998648
Medication	Other diagnostic radiopharmaceuticals	T	379101	3824	375277	0.728	-0.317666	1.00377	0.102	5.21	0.751644	0.998648
Cancer	Lung cancer (self-reported)	T	379101	205	378896	5.07E-05	-9.88868	314.636	7.62E-273	3.38E+263	0.974927	0.998648
Cancer	Breast cancer (self-reported)	T	379101	8389	370712	1.4	0.33797	0.517771	0.508	3.87	0.513924	0.998648
Cancer	Larynx throat cancer (self-reported)	T	379101	289	378812	9.14E-05	-9.30045	188.78	1.85E-165	4.50E+156	0.960707	0.998648
Cancer	Colon cancer sigmoid cancer (self-reported)	T	379101	1220	377881	5.77E-05	-9.76042	116.882	1.86E-104	1.79E+95	0.933449	0.998648
Cancer	Rectal cancer (self-reported)	T	379101	274	378827	9.20E-05	-9.29418	196.328	7.01E-172	1.21E+163	0.962242	0.998648
Cancer	Kidney renal cell cancer (self-reported)	T	379101	493	378608	5.02E-05	-9.89936	196.078	6.25E-172	4.03E+162	0.959734	0.998648
Cancer	Bladder cancer (self-reported)	T	379101	892	378209	8.12E-05	-9.41919	115.461	4.24E-103	1.55E+94	0.934982	0.998648
Cancer	Ovarian cancer (self-reported)	T	379101	645	378456	4.70E-08	-16.8723	5980.34	0	Inf	0.997749	0.998648
Cancer	Uterine endometrial cancer (self-reported)	T	379101	936	378165	3.16	1.15121	1.01048	0.436	22.9	0.254588	0.998648
Cancer	Cervical cancer (self-reported)	T	379101	1533	377568	5.24E-08	-16.7645	3646.6	0	Inf	0.996332	0.998648
Cancer	Prostate cancer (self-reported)	T	379101	2722	376379	0.867	-0.143011	1.01458	0.119	6.33	0.887905	0.998648
Cancer	Testicular cancer (self-reported)	T	379101	670	378431	3.62E-08	-17.1349	6008.79	0	Inf	0.997725	0.998648
Cancer	Hodgkins lymphoma hodgkins disease (self-reported)	T	379101	352	378749	6.80E-05	-9.59636	198.656	5.41E-174	8.55E+164	0.961472	0.998648
Cancer	Non hodgkins lymphoma (self-reported)	T	379101	719	378382	9.08E-05	-9.30649	120.251	3.97E-107	2.08E+98	0.938312	0.998648
Cancer	Chronic lymphocytic (self-reported)	T	379101	244	378857	0.000108	-9.1336	194.227	5.06E-170	2.31E+161	0.962493	0.998648
Cancer	Malignant melanoma (self-reported)	T	379101	2994	376107	1.78	0.576842	0.712869	0.44	7.2	0.418409	0.998648
Cancer	Basal cell carcinoma (self-reported)	T	379101	3552	375549	5.10E-05	-9.88293	72.5919	8.25E-67	3.16E+57	0.891708	0.998648
Cancer	Squamous cell carcinoma (self-reported)	T	379101	470	378631	5.41E-05	-9.82504	195.036	5.19E-171	5.64E+161	0.959823	0.998648
Cancer	Thyroid cancer (self-reported)	T	379101	362	378739	7.39E-05	-9.51308	195.593	2.38E-171	2.29E+162	0.961208	0.998648
Cancer	Cin pre cancer cells cervix (self-reported)	T	379101	1313	377788	6.48E-05	-9.64472	109.869	1.95E-98	2.16E+89	0.930049	0.998648
Neurosciences	Headaches not migraine (self-reported)	T	379101	3224	375877	0.815	-0.204531	1.00415	0.114	5.83	0.8386	0.998648
Cardiovascular	Heart surgery (self-reported)	T	379101	749	378352	8.71E-05	-9.3482	120.723	1.51E-107	5.03E+98	0.938277	0.998648
Cardiovascular	Coronary angioplasty ptca stent (self-reported)	T	379101	3922	375179	0.653	-0.425974	1.00755	0.0906	4.71	0.672454	0.998648
Cardiovascular	Other arterial surgery revascularisation procedures (self-reported)	T	379101	268	378833	9.26E-05	-9.28775	197.63	5.50E-173	1.56E+164	0.962517	0.998648
Cardiovascular	Coronary artery bypass grafts cabg (self-reported)	T	379101	2799	376302	0.932	-0.070927	1.01066	0.129	6.75	0.944052	0.998648
Cardiovascular	Pacemaker defibrillator insertion (self-reported)	T	379101	680	378421	0.000106	-9.15114	116.295	1.08E-103	1.04E+95	0.93728	0.998648
Cardiovascular	Mitral valve repair replacement (self-reported)	T	379101	354	378747	7.19E-05	-9.54091	192.825	5.25E-169	9.83E+159	0.960537	0.998648
Cardiovascular	Fem pop bypass leg artery bypass (self-reported)	T	379101	221	378880	4.49E-05	-10.0104	305.975	1.59E-265	1.27E+256	0.973901	0.998648
Cardiovascular	Aortic aneurysm repair or stent (self-reported)	T	379101	277	378824	9.94E-05	-9.21628	185.486	1.28E-162	7.70E+153	0.960372	0.998648
Cardiovascular	Carotid artery surgery endarterectomy (self-reported)	T	379101	226	378875	4.49E-05	-10.0115	309.477	1.66E-268	1.21E+259	0.974193	0.998648
Cardiovascular	Cerebral artery aneurysm surgery or clipping (self-reported)	T	379101	222	378879	0.000122	-9.00972	198.292	1.98E-173	7.53E+164	0.963759	0.998648
Cardiovascular	Carotid artery angioplasty stent (self-reported)	T	379101	333	378768	7.71E-05	-9.47027	188.435	3.08E-165	1.93E+156	0.959917	0.998648
ENT	Ent surgery (self-reported)	T	379101	283	378818	8.39E-05	-9.38632	199.321	1.81E-174	3.88E+165	0.96244	0.998648
ENT	Ear surgery (self-reported)	T	379101	6011	373090	0.436	-0.830953	1.00354	0.0609	3.11	0.407658	0.998648
ENT	Nasal sinus nose surgery (self-reported)	T	379101	13231	365870	0.974	-0.026298	0.456085	0.398	2.38	0.954018	0.998648
ENT	Throat larynx surgery incl tracheostomy (self-reported)	T	379101	2251	376850	1.18	0.164604	1.00426	0.165	8.44	0.869806	0.998648

ENT	Mouth salivary gland surgery (self-reported)	T	379101	2330	376771	7.94E-05	-9.44042	73.3989	2.64E-67	2.39E+58	0.89766	0.998648
Operations and Procedures	Renal kidney transplant (self-reported)	T	379101	252	378849	9.53E-05	-9.25888	198.356	1.36E-173	6.65E+164	0.96277	0.998648
Operations and Procedures	Percutaneous open kidney stone surgery lithotripsy (self-reported)	T	379101	2177	376924	8.22E-05	-9.40587	72.3246	2.24E-66	3.01E+57	0.896526	0.998648
Operations and Procedures	Prostate operation (self-reported)	T	379101	859	378242	3.32E-08	-17.2196	5708.95	0	Inf	0.997593	0.998648
Operations and Procedures	Radical prostatectomy (self-reported)	T	379101	1237	377864	1.93	0.659243	1.01356	0.265	14.1	0.51542	0.998648
Operations and Procedures	Transurethral resection of prostate turp (self-reported)	T	379101	1932	377169	2.67	0.980877	0.729818	0.638	11.1	0.178948	0.998648
Operations and Procedures	Testicular scrotal operation (self-reported)	T	379101	2430	376671	0.962	-0.038977	1.00768	0.133	6.93	0.969146	0.998648
Operations and Procedures	Removal of testicle orchidectomy (self-reported)	T	379101	1211	377890	5.88E-05	-9.74183	110.501	5.11E-99	6.75E+89	0.929749	0.998648
Operations and Procedures	Vasectomy (self-reported)	T	379101	9426	369675	1.26	0.228651	0.463981	0.506	3.12	0.622153	0.998648
Operations and Procedures	Other urological surgery (self-reported)	T	379101	990	378111	2.64	0.969225	1.00457	0.368	18.9	0.334637	0.998648
Metabolic	Thyroid surgery (self-reported)	T	379101	586	378515	4.57E-05	-9.99264	192.84	3.25E-169	6.44E+159	0.958673	0.998648
Metabolic	Thyroid radioablation therapy (self-reported)	T	379101	360	378741	7.85E-05	-9.45217	194.521	2.07E-170	2.98E+161	0.961244	0.998648
Metabolic	Parathyroidectomy (self-reported)	T	379101	531	378570	4.80E-05	-9.94363	194.571	1.15E-170	2.01E+161	0.959242	0.998648
Operations and Procedures	Bone surgery joint surgery (self-reported)	T	379101	3136	375965	5.52E-05	-9.80535	73.4398	1.69E-67	1.80E+58	0.893786	0.998648
Operations and Procedures	Muscle soft tissue surgery (self-reported)	T	379101	10926	368175	0.467	-0.76074	0.711989	0.116	1.89	0.285308	0.998648
Operations and Procedures	Hip replacement revision (self-reported)	T	379101	6037	373064	1.36	0.308711	0.587682	0.43	4.31	0.599373	0.998648
Operations and Procedures	Knee replacement revision (self-reported)	T	379101	4614	374487	0.574	-0.554633	1.00593	0.08	4.12	0.581385	0.998648
Operations and Procedures	Spine or back surgery (self-reported)	T	379101	4637	374464	1.11	0.10301	0.712368	0.274	4.48	0.885025	0.998648
Gynaecology and Obstetrics	Gynaecological surgery (self-reported)	T	379101	2168	376933	2.59	0.951003	0.718023	0.634	10.6	0.185346	0.998648
Gynaecology and Obstetrics	Bilateral oophorectomy (self-reported)	T	379101	15863	363238	1.13	0.125207	0.429906	0.488	2.63	0.770866	0.998648
Gynaecology and Obstetrics	Unilateral oophorectomy (self-reported)	T	379101	2254	376847	3.92E-05	-10.1469	110.3	5.06E-99	3.04E+89	0.926703	0.998648
Gynaecology and Obstetrics	Hysterectomy (self-reported)	T	379101	37549	341552	0.746	-0.293616	0.346683	0.378	1.47	0.397035	0.998648
Gynaecology and Obstetrics	Endometrial ablation (self-reported)	T	379101	1925	377176	4.49E-05	-10.0115	109.853	1.39E-98	1.45E+89	0.927385	0.998648
Gynaecology and Obstetrics	Vaginal prolapse colposuspension (self-reported)	T	379101	3479	375622	6.77E-08	-16.5088	2174.72	0	Inf	0.993943	0.998648
Gynaecology and Obstetrics	Sterilisation (self-reported)	T	379101	16084	363017	0.897	-0.108583	0.464712	0.361	2.23	0.815252	0.998648
Gynaecology and Obstetrics	Intrauterine contraceptive device insertion removal (self-reported)	T	379101	624	378477	4.91E-05	-9.92167	179.032	1.98E-157	1.22E+148	0.955805	0.998648
Gynaecology and Obstetrics	Breast surgery (self-reported)	T	379101	1320	377781	6.27E-05	-9.6778	112.045	2.64E-100	1.48E+91	0.931169	0.998648
Gynaecology and Obstetrics	Lumpectomy (self-reported)	T	379101	12746	366355	0.66	-0.415114	0.589865	0.208	2.1	0.481591	0.998648
Gynaecology and Obstetrics	Mammoplasty cosmetic operation on breast (self-reported)	T	379101	2699	376402	8.54E-05	-9.36827	67.3328	4.14E-62	1.76E+53	0.889344	0.998648
Cardiovascular	Venous surgery procedures (self-reported)	T	379101	246	378855	0.000107	-9.13857	197.792	4.65E-173	2.48E+164	0.963148	0.998648
Operations and Procedures	Hernia surgery (self-reported)	T	379101	2430	376671	1.01	0.006845	1.00548	0.14	7.23	0.994569	0.998648
Operations and Procedures	Umbilical hernia repair (self-reported)	T	379101	3357	375744	1.54	0.430596	0.712938	0.38	6.22	0.545862	0.998648
Operations and Procedures	Incisional hernia repair (self-reported)	T	379101	1461	377640	1.77	0.572506	1.00454	0.247	12.7	0.568732	0.998648
Operations and Procedures	Bladder surgery (self-reported)	T	379101	2401	376700	7.84E-05	-9.45362	72.6581	1.11E-66	5.52E+57	0.896479	0.998648
Operations and Procedures	Transurethral resection bladder tumour turbt (self-reported)	T	379101	458	378643	5.83E-05	-9.74963	190.128	8.42E-167	4.04E+157	0.959103	0.998648
Operations and Procedures	Cystectomy (self-reported)	T	379101	1986	377115	9.05E-05	-9.30984	73.4485	2.73E-67	3.00E+58	0.899136	0.998648
Operations and Procedures	Urethral surgery (self-reported)	T	379101	686	378415	3.72	1.31383	1.00458	0.519	26.7	0.19093	0.998648
Metabolic	Thyroidectomy partial thyroidectomy (self-reported)	T	379101	3577	375524	1.58	0.454571	0.715364	0.388	6.4	0.525142	0.998648
Eye	Cataract extraction lens implant (self-reported)	T	379101	4095	375006	1.94	0.663061	0.586453	0.615	6.13	0.258211	0.998648
Eye	Glaucoma surgery trabeculectomy (self-reported)	T	379101	535	378566	4.71E-05	-9.96274	194.428	1.49E-170	1.49E+161	0.959133	0.998648
Eye	Retinal operation vitrectomy (self-reported)	T	379101	2253	376848	1.12	0.11655	1.00472	0.157	8.05	0.907651	0.998648
Operations and Procedures	Reduction or fixation of bone fracture (self-reported)	T	379101	17474	361627	1.47	0.386565	0.329028	0.772	2.81	0.240047	0.998648
Operations and Procedures	Other thoracic surgery (self-reported)	T	379101	1195	377906	2.12	0.749804	1.00449	0.296	15.2	0.455395	0.998648
Operations and Procedures	Stomach surgery (self-reported)	T	379101	2197	376904	1.24	0.214709	1.00451	0.173	8.88	0.830746	0.998648
Operations and Procedures	Oesophageal surgery (self-reported)	T	379101	447	378654	5.28E-05	-9.84981	195.972	8.08E-172	3.44E+162	0.959914	0.998648
Operations and Procedures	Pancreas surgery (self-reported)	T	379101	205	378896	0.000118	-9.04867	198.56	1.13E-173	1.22E+165	0.963652	0.998648
Operations and Procedures	Splenectomy (self-reported)	T	379101	791	378310	8.22E-05	-9.40658	120.918	9.71E-108	6.95E+98	0.937993	0.998648
Operations and Procedures	Cholecystectomy gall bladder removal (self-reported)	T	379101	14872	364229	0.558	-0.582852	0.586275	0.177	1.76	0.320144	0.998648
Operations and Procedures	Appendicectomy (self-reported)	T	379101	45428	333673	1.35	0.297854	0.232485	0.854	2.12	0.200132	0.998648
Operations and Procedures	Bowel resection (self-reported)	T	379101	706	378395	9.84E-05	-9.22653	120.279	4.07E-107	2.38E+98	0.938855	0.998648
Operations and Procedures	Anal surgery (self-reported)	T	379101	2357	376744	2.17	0.776097	0.71285	0.537	8.79	0.276276	0.998648
Operations and Procedures	Large bowel resection colostomy (self-reported)	T	379101	1039	378062	2.55	0.935675	1.00476	0.356	18.3	0.351729	0.998648
Operations and Procedures	Small bowel resection (self-reported)	T	379101	657	378444	0.000103	-9.18546	120.853	1.38E-107	7.64E+98	0.939415	0.998648
Operations and Procedures	Rectal or colon polypectomy (self-reported)	T	379101	1567	377534	1.74	0.555386	1.00488	0.243	12.5	0.580475	0.998648
Operations and Procedures	Colectomy hemicolectomy (self-reported)	T	379101	1636	377465	1.63	0.486556	1.0045	0.227	11.7	0.62812	0.998648
Operations and Procedures	Other bowel surgery (self-reported)	T	379101	2282	376819	8.19E-05	-9.40967	73.1597	4.35E-67	1.54E+58	0.89766	0.998648
Operations and Procedures	Brain surgery (self-reported)	T	379101	1560	377541	4.15E-05	-10.0889	121.18	2.94E-108	5.87E+98	0.933649	0.998648
Operations and Procedures	Intracranial haematoma drainage (self-reported)	T	379101	205	378896	0.000123	-9.00456	196.703	4.49E-172	3.36E+163	0.963488	0.998648
Operations and Procedures	Spinal cord surgery (self-reported)	T	379101	508	378593	4.77E-05	-9.95088	199.073	1.68E-174	1.36E+165	0.960134	0.998648
Operations and Procedures	Peripheral nerve surgery (self-reported)	T	379101	794	378307	8.45E-05	-9.3786	121.114	6.80E-108	1.05E+99	0.938277	0.998648
Operations and Procedures	Pituitary surgery (self-reported)	T	379101	215	378886	0.00012	-9.0311	198.821	6.89E-174	2.08E+165	0.96377	0.998648
Operations and Procedures	Skin operation or plastic surgery (self-reported)	T	379101	3215	375886	0.806	-0.215827	1.00365	0.113	5.76	0.829734	0.998648
Gynaecology and Obstetrics	Fertility treatment procedures (self-reported)	T	379101	966	378135	8.63E-05	-9.35741	110.994	2.86E-99	2.61E+90	0.932813	0.998648
Gynaecology and Obstetrics	Caesarean section caesarian section (self-reported)	T	379101	19341	359760	1.06	0.059135	0.399847	0.485	2.32	0.882427	0.998648
ENT	Wisdom teeth surgery (self-reported)	T	379101	15032	364069	0.985	-0.014751	0.418893	0.434	2.24	0.971909	0.998648
ENT	Maxillo facial surgery (self-reported)	T	379101	2821	376280	6.19E-05	-9.69046	73.1792	3.16E-67	1.21E+58	0.894651	0.998648
Operations and Procedures	Oesphageal fundoplication hiatus hernia surgery (self-reported)	T	379101	906	378195	7.23E-05	-9.53448	120.513	1.89E-107	2.77E+98	0.936941	0.998648
Operations and Procedures	Haemorroidectomy piles surgery banding of piles (self-reported)	T	379101	6713	372388	0.389	-0.945444	1.00428	0.0543	2.78	0.346494	0.998648
Operations and Procedures	Pilonidal sinus surgery anal (self-reported)	T	379101	2174	376927	8.13E-05	-9.41697	72.3268	2.21E-66	2.99E+57	0.896408	0.998648
Operations and Procedures	Abdominal pelvic adhesion surgery (self-reported)	T	379101	1192	377909	5.99E-05	-9.72306	119.543	1.05E-106	3.42E+97	0.935176	0.998648
Operations and Procedures	Laparotomy nos (self-reported)	T	379101	2106	376995	1.3	0.265729	1.00425	0.182	9.34	0.791315	0.998648
Operations and Procedures	Nephrectomy kidney removed (self-reported)	T	379101	1353	377748	1.95	0.66788	1.00433	0.272	14	0.506052	0.998648
Operations and Procedures	Anterior posterior repair bladder uterus (self-reported)	T	379101	3518	375583	0.852	-0.159915	1.00877	0.118	6.16	0.874044	0.998648
Operations and Procedures	Testicular hydrocoele surgery drainage (self-reported)	T	379101	1140	377961	5.69E-08	-16.6824	3658.24	0	Inf	0.996361	0.998648
Operations and Procedures	Male circumcision (self-reported)	T	379101	3497	375604	4.95E-08	-16.8214	2210.62	0	Inf	0.993929	0.998648
Eye	Squint correction (self-reported)	T	379101	5064	374037	3.45E-05	-10.2755	73.4619	1.01E-67	1.17E+58	0.888759	0.998648
Operations and Procedures	Spinal laminectomy (self-reported)	T	379101	3219	375882	5.58E-05	-9.79388	73.1272	3.16E-67	9.86E+57	0.893458	0.998648
Operations and Procedures	Arthroscopy nos (self-reported)	T	379101	8377	370724	0.901	-0.104711	0.584073	0.287	2.83	0.857721	0.998648
Operations and Procedures	Foot surgery (self-reported)	T	379101	7430	371671	0.708	-0.345863	0.712392	0.175	2.86	0.627325	0.998648
Operations and Procedures	Lower limb surgery (self-reported)	T	379101	1770	377331	3.75E-05	-10.1916	120.176	1.90E-107	7.41E+97	0.932416	0.998648
Operations and Procedures	Knee surgery not replacement (self-reported)	T	379101	12555	366546	1.66	0.506956	0.365901	0.81	3.4	0.1659	0.998648
Operations and Procedures	Hip surgery not replacement (self-reported)	T	379101	1523	377578	1.65	0.503783	1.00405	0.231	11.8	0.615842	0.998648
Operations and Procedures	Upper limb surgery (self-reported)	T	379101	2145	376956	2.5	0.917679	0.713009	0.619	10.1	0.198077	0.998648
Operations and Procedures	Shoulder surgery (self-reported)	T	379101	5679	373422	0.896	-0.110075	0.712517	0.222	3.62	0.877225	0.998648
Operations and Procedures	Elbow surgery (self-reported)	T	379101	2248	376853	7.77E-05	-9.46305	73.2011	3.80E-67	1.59E+58	0.89714	0.998648
Operations and Procedures	Carpal tunnel surgery (self-reported)	T	379101	3953	375148	4.65E-05	-9.97634	72.506	8.89E-67	2.43E+57	0.890562	0.998648
Operations and Procedures	Cruciate ligament surgery (self-reported)	T	379101	1888	377213	3.48E-05	-10.2661	118.534	4.40E-106	2.75E+96	0.930982	0.998648
Operations and Procedures	Bunion hallus valgus surgery (self-reported)	T	379101	4996	374105	4.19E-05	-10.0797	69.5382	2.69E-64	6.52E+54	0.884749	0.998648
Operations and Procedures	Removal of mole skin lesion (self-reported)	T	379101	5310	373791	0.501	-0.690453	1.00355	0.0701	3.58	0.491447	0.998648
Gynaecology and Obstetrics	Ovarian tubal surgery (self-reported)	T	379101	1138	377963	7.06E-08	-16.4657	3646.84	0	Inf	0.996398	0.998648
Gynaecology and Obstetrics	Ovarian cyst removal surgery (self-reported)	T	379101	4517	374584	4.64E-08	-16.8854	2207.97	0	Inf	0.993898	0.998648
Gynaecology and Obstetrics	Ectopic pregnancy surgery (self-reported)	T	379101	2218	376883	3.47E-08	-17.177	3639.59	0	Inf	0.996234	0.998648
Gynaecology and Obstetrics	Uterine surgery (self-reported)	T	379101	1594	377507	5.03E-08	-16.8054	3645.59	0	Inf	0.996322	0.998648
Gynaecology and Obstetrics	Myomectomy fibroids removed (self-reported)	T	379101	2071	377030	4.08E-05	-10.1066	110.266	5.63E-99	2.96E+89	0.92697	0.998648
Gynaecology and Obstetrics	Dilatation and curettage d c (self-reported)	T	379101	21385	357716	0.64	-0.445905	0.464581	0.258	1.59	0.337156	0.998648
Gynaecology and Obstetrics	Cervix surgery (self-reported)	T	379101	1570	377531	5.69E-05	-9.77431	110.436	5.62E-99	5.76E+89	0.929474	0.998648
Gynaecology and Obstetrics	Cone biopsy (self-reported)	T	379101	3152	375949	7.53E-05	-9.49367	66.868	9.07E-62	6.25E+52	0.887099	0.998648
Gynaecology and Obstetrics	Breast cyst abscess removal (self-reported)	T	379101	3620	375481	0.822	-0.196193	1.00729	0.114	5.92	0.845571	0.998648
Gynaecology and Obstetrics	Coronary angiogram (self-reported)	T	379101	2517	376584	1.06	0.059205	1.00602	0.148	7.62	0.953071	0.998648
Cardiovascular	Angiogram not coronary (self-reported)	T	379101	345	378756	7.78E-05	-9.46082	196.9	1.93E-172	3.13E+163	0.961677	0.998648
Cardiovascular	Echocardiogram transoesophageal echo (self-reported)	T	379101	455	378646	5.97E-05	-9.72595	195.792	1.30E-171	2.74E+162	0.960381	0.998648
Operations and Procedures	Endoscopy gastroscopy (self-reported)	T	379101	7551	371550	1.86	0.622268	0.456198	0.762	4.56	0.172558	0.998648
Operations and Procedures	Ercp endoscopic retrograde cholangiopancreatogram (self-reported)	T	379101	341	378760	7.77E-05	-9.46241	198.15	1.67E-173	3.62E+164	0.961913	0.998648
Operations and Procedures	Colonoscopy sigmoidoscopy (self-reported)	T	379101	7510	371591	0.736	-0.306989	0.712576	0.182	2.97	0.666602	0.998648
Operations and Procedures	Laparoscopy (self-reported)	T	379101	6734	372367	0.424	-0.858386	1.00782	0.0588	3.06	0.394366	0.998648
Gynaecology and Obstetrics	Hysteroscopy biopsy (self-reported)	T	379101	1325	377776	6.24E-05	-9.68248	113.46	1.64E-101	2.37E+92	0.931992	0.998648
Operations and Procedures	Colposcopy biopsy (self-reported)	T	379101	1641	377460	5.18E-05	-9.86853	113.829	6.62E-102	4.05E+92	0.930913	0.998648
Cardiovascular	Triple heart bypass (self-reported)	T	379101	526	378575	5.43E-05	-9.82064	178.677	4.39E-157	6.73E+147	0.956168	0.998648
Gynaecology and Obstetrics	Mastoidectomy mastoid surgery (self-reported)	T	379101	700	378401	9.80E-05	-9.2306	119.432	2.13E-106	4.51E+97	0.938395	0.998648
Operations and Procedures	Discectomy (self-reported)	T	379101	1381	377720	1.87	0.627784	1.00403	0.262	13.4	0.531799	0.998648
Operations and Procedures	Ankle surgery (self-reported)	T	379101	1003	378098	6.53E-05	-9.63692	120.798	9.76E-108	4.37E+98	0.936414	0.998648
Operations and Procedures	Achilles tendon repair (self-reported)	T	379101	613	378488	0.000103	-9.18274	119.415	2.31E-106	4.57E+97	0.938705	0.998648
Operations and Procedures	Hand finger surgery (self-reported)	T	379101	1664	377437	3.84E-05	-10.1673	120.493	1.04E-107	1.41E+98	0.932754	0.998648
Operations and Procedures	Dupuytren s contracture surgery (self-reported)	T	379101	409	378692	6.46E-05	-9.64764	188.71	1.50E-165	2.78E+156	0.959227	0.998648
Operations and Procedures	Lipoma removed excision of lipoma (self-reported)	T	379101	600	378501	0.000108	-9.13253	120.756	1.75E-107	6.66E+98	0.939715	0.998648

Operations and Procedures	Lymph node surgery (self-reported)	T	379101	763	378338	3.49	1.25088	1.00561	0.487	25.1	0.213538	0.998648
Gynaecology and Obstetrics	Salpingectomy (self-reported)	T	379101	258	378843	1.16E-07	-15.972	6003.6	0	Inf	0.997877	0.998648
Gynaecology and Obstetrics	Termination of pregnancy top (self-reported)	T	379101	945	378156	7.66E-08	-16.3842	3624.1	0	Inf	0.996393	0.998648
Gynaecology and Obstetrics	Cervical polyps removed (self-reported)	T	379101	370	378731	8.37E-05	-9.3883	181.533	2.50E-159	2.80E+150	0.958754	0.998648
Gynaecology and Obstetrics	Laser treatment cervix (self-reported)	T	379101	611	378490	4.81E-08	-16.8506	5958.21	0	Inf	0.997743	0.998648
Gynaecology and Obstetrics	Pelvic floor surgery (self-reported)	T	379101	271	378830	0.000116	-9.05849	182.661	3.82E-160	3.55E+151	0.960448	0.998648
Gynaecology and Obstetrics	Breast biopsy (self-reported)	T	379101	375	378726	7.89E-05	-9.44742	182.043	8.69E-160	7.16E+150	0.958611	0.998648
Cardiovascular	Ecg electrocardiogram (self-reported)	T	379101	201	378900	0.000129	-8.95617	197.416	1.17E-172	1.43E+164	0.963815	0.998648
Operations and Procedures	Cystoscopy (self-reported)	T	379101	345	378756	7.08E-05	-9.55553	197.161	1.06E-172	4.75E+163	0.961345	0.998648
Cardiovascular	Pacemaker insertion (self-reported)	T	379101	287	378814	8.66E-05	-9.3541	190.981	2.35E-167	3.19E+158	0.960936	0.998648
Cardiovascular	Cardiac ablation (self-reported)	T	379101	269	378832	8.58E-05	-9.3638	197.158	1.29E-172	5.72E+163	0.96212	0.998648
ENT	Tympanic membrane surgery ear drum repair (self-reported)	T	379101	602	378499	0.000108	-9.13208	120.918	1.28E-107	9.15E+98	0.939799	0.998648
ENT	Rhinoplasty nose surgery (self-reported)	T	379101	2193	376908	7.80E-05	-9.45934	72.8527	7.56E-67	8.04E+57	0.896692	0.998648
ENT	Sinus surgery (self-reported)	T	379101	1301	377800	4.86E-05	-9.93289	120.767	7.71E-108	3.06E+98	0.934449	0.998648
ENT	Nasal polyp surgery nasal polypectomy (self-reported)	T	379101	1137	377964	5.74E-05	-9.76527	119.439	1.23E-106	2.68E+97	0.934838	0.998648
ENT	Tonsillectomy tonsil surgery (self-reported)	T	379101	17457	361644	0.841	-0.1734	0.418071	0.371	1.91	0.678316	0.998648
ENT	Adenoid surgery adenoidectomy (self-reported)	T	379101	2205	376896	7.53E-05	-9.49372	73.1843	3.81E-67	1.49E+58	0.896785	0.998648
Operations and Procedures	Inguinal hernia repair (self-reported)	T	379101	5531	373570	1.78	0.579192	0.514596	0.651	4.89	0.260365	0.998648
Operations and Procedures	Femoral hernia repair (self-reported)	T	379101	326	378775	7.15E-05	-9.54569	196.954	1.60E-172	3.20E+163	0.961344	0.998648
Operations and Procedures	Urethral stricture surgery dilatation (self-reported)	T	379101	205	378896	0.00012	-9.02758	198.357	1.72E-173	8.40E+164	0.963699	0.998648
Operations and Procedures	Orchidopexy (self-reported)	T	379101	293	378808	8.08E-08	-16.3312	5990.82	0	Inf	0.997825	0.998648
Operations and Procedures	Spinal fusion (self-reported)	T	379101	517	378584	4.48E-05	-10.0122	198.918	2.13E-174	9.42E+164	0.959857	0.998648
Eye	Eyelid surgery (self-reported)	T	379101	269	378832	8.81E-05	-9.33754	199.276	2.08E-174	3.73E+165	0.962627	0.998648
Operations and Procedures	Removal of benign skin lesion (self-reported)	T	379101	629	378472	0.000101	-9.2011	121.131	7.86E-108	1.30E+99	0.939451	0.998648
Operations and Procedures	Removal of rodent ulcer basal cell carcinoma bcc (self-reported)	T	379101	957	378144	2.77	1.01816	1.0051	0.386	19.8	0.311066	0.998648
Operations and Procedures	Skin graft (self-reported)	T	379101	649	378452	9.80E-05	-9.23031	120.694	1.80E-107	5.35E+98	0.93904	0.998648
Operations and Procedures	Mri magnetic resonance imaging (self-reported)	T	379101	522	378579	4.50E-05	-10.0083	199.192	1.25E-174	1.62E+165	0.959928	0.998648
Operations and Procedures	Ct scan (self-reported)	T	379101	307	378794	7.96E-05	-9.43912	198.573	7.45E-174	8.50E+164	0.962087	0.998648
Operations and Procedures	Bone scan (self-reported)	T	379101	213	378888	5.02E-05	-9.8986	314.914	4.37E-273	5.77E+263	0.974924	0.998648
Operations and Procedures	Ultrasound scan (self-reported)	T	379101	230	378871	0.000107	-9.13865	199.172	3.11E-174	3.71E+165	0.963403	0.998648
Operations and Procedures	Barium meal barium swallow (self-reported)	T	379101	221	378880	0.000107	-9.13992	198.145	2.32E-173	4.95E+164	0.963209	0.998648
Operations and Procedures	Biopsy (self-reported)	T	379101	275	378826	8.62E-05	-9.35887	199.236	2.20E-174	3.38E+165	0.962534	0.998648
Operations and Procedures	Prostate biopsy (self-reported)	T	379101	258	378843	3.86E-08	-17.071	9507.9	0	Inf	0.998567	0.998648
Family history	Alzheimer s disease dementia (family history - father)	T	343592	15771	327821	0.479	-0.735175	0.584235	0.153	1.51	0.208263	0.998648
Family history	Parkinson s disease (family history - father)	T	338729	8344	330385	0.298	-1.20943	0.995627	0.0424	2.1	0.224466	0.998648
Family history	Severe depression (family history - father)	T	338729	12638	326091	0.792	-0.233072	0.508233	0.293	2.14	0.646526	0.998648
Family history	Prostate cancer (family history - father)	T	338729	25442	313287	0.869	-0.140782	0.345514	0.441	1.71	0.683672	0.998648
Family history	Stroke (family history - father)	T	343592	51292	292300	1.13	0.121414	0.233603	0.714	1.78	0.603242	0.998648
Family history	Lung cancer (family history - father)	T	338729	30466	308263	0.959	-0.042365	0.315719	0.516	1.78	0.893256	0.998648
Family history	Bowel cancer (family history - father)	T	338729	19587	319142	1.05	0.046285	0.364974	0.512	2.14	0.899085	0.998648
Family history	Chronic bronchitis emphysema (family history - father)	T	343592	37574	306018	1.17	0.158932	0.26793	0.693	1.98	0.553058	0.998648
Family history	Diabetes (family history - father)	T	343592	31628	311964	0.774	-0.256252	0.329701	0.406	1.48	0.437027	0.998648
Family history	Alzheimer s disease dementia (family history - mother)	T	357529	29815	327714	0.946	-0.055457	0.316575	0.509	1.76	0.86094	0.998648
Family history	Parkinson s disease (family history - mother)	T	354181	5725	348456	1.34	0.294448	0.584439	0.427	4.22	0.614392	0.998648
Family history	Severe depression (family history - mother)	T	354181	23297	330884	0.879	-0.128495	0.36482	0.43	1.8	0.724678	0.998648
Family history	Heart disease (family history - mother)	T	357529	69487	288042	0.807	-0.213964	0.233704	0.511	1.28	0.359912	0.998648
Family history	Stroke (family history - mother)	T	357529	49690	307839	1.06	0.057826	0.24353	0.657	1.71	0.812308	0.998648
Family history	Lung cancer (family history - mother)	T	354181	14282	339899	0.938	-0.064246	0.456116	0.384	2.29	0.887985	0.998648
Family history	Breast cancer (family history - mother)	T	350614	12118	338496	0.628	-0.464939	0.584019	0.2	1.97	0.425973	0.998648
Family history	High blood pressure (family history - mother)	T	357529	106967	250562	0.841	-0.173638	0.1932	0.576	1.23	0.368787	0.998648
Family history	Diabetes (family history - mother)	T	357529	32382	325147	0.851	-0.161761	0.314356	0.459	1.58	0.606847	0.998648
Family history	Alzheimer s disease dementia (family history - sibling)	T	301029	1677	299352	3.48E-05	-10.2664	123.064	6.13E-110	1.97E+100	0.933515	0.998648
Family history	Parkinson s disease (family history - sibling)	T	301236	1576	299660	3.38E-05	-10.2946	124.634	2.74E-111	4.17E+101	0.934171	0.998648
Family history	Severe depression (family history - sibling)	T	301236	21287	279949	0.799	-0.224484	0.389536	0.372	1.71	0.564421	0.998648
Family history	Prostate cancer (family history - sibling)	T	301236	4736	296500	1.98	0.680626	0.513306	0.722	5.4	0.184851	0.998648
Family history	Stroke (family history - sibling)	T	301029	9517	291512	0.77	-0.261321	0.587049	0.244	2.43	0.656216	0.998648
Family history	Lung cancer (family history - sibling)	T	301236	6394	294842	0.748	-0.290672	0.716304	0.184	3.04	0.684894	0.998648
Family history	Bowel cancer (family history - sibling)	T	301236	7001	294235	1.02	0.017837	0.587534	0.322	3.22	0.975781	0.998648
Family history	Breast cancer (family history - sibling)	T	298653	4866	293787	3.06E-05	-10.3935	76.8364	1.21E-70	7.77E+60	0.8924	0.998648
Family history	High blood pressure (family history - sibling)	T	301029	61052	239977	0.996	-0.003754	0.225962	0.64	1.55	0.986746	0.998648
Family history	Diabetes (family history - sibling)	T	301029	24485	276544	0.994	-0.006021	0.331201	0.519	1.9	0.985496	0.998648
Mental health	Single episode of probable major depression	T	54642	7501	47141	1.21	0.189594	0.450399	0.5	2.92	0.673793	0.998648
Mental health	Probable recurrent major depression severe 2	T	54213	7072	47141	0.436	-0.83099	0.729194	0.104	1.82	0.254453	0.998648
Mental health	Bipolar I Disorder	T	91515	595	90920	2.68E-05	-10.5259	208.411	1.06E-182	6.79E+172	0.95972	0.998648
Mental health	Bipolar II Disorder	T	91515	531	90984	2.91E-05	-10.4442	208.973	3.83E-183	2.22E+173	0.960139	0.998648
Mental health	Probable Recurrent major depression severe 1	T	91515	6715	84800	0.54	-0.617052	0.72308	0.131	2.23	0.393456	0.998648
Mental health	Probable Recurrent major depression moderate	T	91515	11528	79987	0.445	-0.809761	0.599102	0.138	1.44	0.176495	0.998648
Mental health	Risk taking	T	365648	96122	269526	1.02	0.023915	0.193605	0.701	1.5	0.901691	0.998648
Mental health	Seen doctor GP for nerves anxiety tension or depression	T	376439	129804	246635	0.889	-0.117802	0.183884	0.62	1.27	0.521763	0.998648
Mental health	Seen a psychiatrist for nerves anxiety tension or depression	T	377320	43632	333688	0.837	-0.177722	0.281533	0.482	1.45	0.527867	0.998648
Eye	Wears glasses or contact lenses	T	378528	337207	41321	0.752	-0.285564	0.267739	0.445	1.27	0.286162	0.998648
Eye	Other eye problems	T	377958	54312	323646	1.02	0.023358	0.236947	0.643	1.63	0.921473	0.998648
ENT	Hearing difficulty problems	T	363644	94775	268869	0.805	-0.216853	0.21039	0.533	1.22	0.302671	0.998648
ENT	Hearing difficulty problems with background noise	T	371480	140624	230856	0.825	-0.192618	0.181576	0.578	1.18	0.288777	0.998648
Other	Hair balding pattern	T	173279	117813	55466	0.795	-0.228994	0.24481	0.492	1.29	0.349584	0.998648
Other	Pattern 1 f	T	173279	55466	117813	1.26	0.228994	0.24481	0.778	2.03	0.349584	0.998648
Other	Pattern 2 f	T	173279	39959	133320	0.726	-0.320811	0.307894	0.397	1.33	0.297434	0.998648
Other	Pattern 3 f	T	173279	46281	126998	1.15	0.139075	0.261663	0.688	1.92	0.59507	0.998648
Other	Pattern 4 f	T	173279	31573	141706	0.821	-0.197367	0.328705	0.431	1.56	0.548214	0.998648
Gynaecology and Obstetrics	Ever had stillbirth spontaneous miscarriage or termination	T	201246	64150	137096	1.36	0.310036	0.243563	0.846	2.2	0.203045	0.998648
Gynaecology and Obstetrics	Bilateral oophorectomy both ovaries removed	T	201585	16364	185221	1.09	0.089657	0.430442	0.47	2.54	0.835002	0.998648
Neurosciences	General pain for 3 months vs no pain	T	377203	4819	372384	0.572	-0.558517	1.00374	0.08	4.09	0.577911	0.998648
ENT	Hearing aid user	T	230555	11351	219204	0.698	-0.359241	0.592056	0.219	2.23	0.544005	0.998648
Neurosciences	Neck shoulder pain for 3 months vs no pain	T	344932	58990	285942	0.889	-0.117952	0.243007	0.552	1.43	0.627403	0.998648
Gynaecology and Obstetrics	Ever had hysterectomy womb removed	T	180543	14618	165925	0.58	-0.543986	0.601126	0.179	1.89	0.365494	0.998648
Neurosciences	Headaches for 3 months vs no pain	T	330531	33891	296640	1.21	0.189505	0.279521	0.699	2.09	0.497795	0.998648
Cancer	Mal neo oesophagus (cancer register)	T	379101	516	378585	5.17E-05	-9.86928	190.314	5.19E-167	5.16E+157	0.958642	0.998648
Cancer	Mal neo stomach (cancer register)	T	379101	377	378724	7.04E-05	-9.56098	191.509	6.79E-168	7.30E+158	0.960183	0.998648
Cancer	Mal neo colon (cancer register)	T	379101	2575	376526	1.03	0.027575	1.00544	0.143	7.38	0.97812	0.998648
Cancer	Mal neo rectosigmoid (cancer register)	T	379101	343	378758	7.34E-05	-9.51903	193.022	3.65E-169	1.48E+160	0.960668	0.998648
Cancer	Mal neo rectum (cancer register)	T	379101	1203	377898	5.76E-05	-9.76263	117.997	2.08E-105	1.59E+96	0.934061	0.998648
Cancer	Mal neo liver (cancer register)	T	379101	217	378884	0.000122	-9.01114	192.765	1.00E-168	1.48E+160	0.962715	0.998648
Cancer	Mal neo pancreas (cancer register)	T	379101	463	378638	5.48E-05	-9.81228	193.782	6.14E-170	4.89E+160	0.959616	0.998648
Cancer	Mal neo larynx (cancer register)	T	379101	224	378877	4.66E-05	-9.97401	302.88	7.11E-263	3.05E+253	0.97373	0.998648
Cancer	Mal neo bronchus and lung (cancer register)	T	379101	1742	377359	4.41E-05	-10.03	116.251	4.89E-104	3.97E+94	0.931244	0.998648
Cancer	Mal melanoma of skin (cancer register)	T	379101	2611	376490	2	0.695261	0.712721	0.496	8.1	0.329311	0.998648
Cancer	Mal neo other connect soft tissue (cancer register)	T	379101	247	378854	9.67E-05	-9.24408	198.351	1.40E-173	6.69E+164	0.962828	0.998648
Cancer	Mal neo breast (cancer register)	T	379101	10158	368943	1.47	0.383264	0.465272	0.589	3.65	0.410086	0.998648
Cancer	Mal neo cervix uteri (cancer register)	T	379101	384	378717	7.74E-08	-16.3747	6004.91	0	Inf	0.997824	0.998648
Cancer	Mal neo corpus uteri (cancer register)	T	379101	1276	377825	2.33	0.847916	1.01044	0.322	16.9	0.401382	0.998648
Cancer	Mal neo ovary (cancer register)	T	379101	893	378208	9.29E-08	-16.192	3630.88	0	Inf	0.996442	0.998648
Cancer	Mal neo prostate (cancer register)	T	379101	6546	372555	1.11	0.105743	0.598312	0.344	3.59	0.859716	0.998648
Cancer	Mal neo testis (cancer register)	T	379101	451	378650	5.50E-08	-16.7155	5965.21	0	Inf	0.997764	0.998648
Cancer	Mal neo kidney (cancer register)	T	379101	918	378183	2.76	1.01528	1.00567	0.384	19.8	0.312709	0.998648
Cancer	Mal neo bladder (cancer register)	T	379101	919	378182	7.69E-05	-9.47255	113.278	2.90E-101	2.04E+92	0.933357	0.998648
Cancer	Mal neo brain (cancer register)	T	379101	407	378694	5.86E-05	-9.74515	198.644	4.77E-174	7.19E+164	0.960873	0.998648
Cancer	Mal neo thyroid gland (cancer register)	T	379101	431	378670	6.19E-05	-9.68988	197.727	3.04E-173	1.26E+164	0.960914	0.998648
Cancer	Hodgkin s disease (cancer register)	T	379101	245	378856	9.71E-05	-9.23994	198.515	1.02E-173	9.26E+164	0.962876	0.998648
Cancer	Diffuse non Hodgkin s lymphoma (cancer register)	T	379101	730	378371	9.24E-05	-9.28934	118.886	5.86E-106	1.46E+97	0.937719	0.998648
Cancer	Other types of non Hodgkin s lymphoma (cancer register)	T	379101	408	378693	5.98E-05	-9.72399	197.593	3.82E-173	9.36E+163	0.96075	0.998648
Cancer	Multiple myeloma (cancer register)	T	379101	459	378642	5.66E-05	-9.77867	195.128	4.54E-171	7.07E+161	0.960031	0.998648
Cancer	Lymphoid leukaemia (cancer register)	T	379101	519	378582	4.94E-05	-9.91615	194.51	1.33E-170	1.84E+161	0.959341	0.998648
Cancer	Myeloid leukaemia (cancer register)	T	379101	321	378780	7.73E-05	-9.46842	197.528	5.61E-173	1.06E+164	0.961768	0.998648
Cancer	Carc in situ of other digestive organs (cancer register)	T	379101	262	378839	9.50E-05	-9.26151	195.253	5.96E-171	1.52E+162	0.962168	0.998648
Cancer	Melanoma in situ (cancer register)	T	379101	1026	378075	2.54	0.933049	1.00458	0.355	18.2	0.352994	0.998648

Cancer	Carc in situ of skin (cancer register)	T	379101	1157	377944	6.84E-05	-9.58975	115.551	2.99E-103	1.56E+94	0.933859	0.998648
Cancer	Carc in situ of breast (cancer register)	T	379101	1900	377201	4.57E-05	-9.99368	109.987	1.09E-98	1.92E+89	0.927602	0.998648
Cancer	Carc in situ of cervix (cancer register)	T	379101	2991	376110	7.97E-08	-16.3448	2184.63	0	Inf	0.99403	0.998648
Cancer	Car in situ other genital (cancer register)	T	379101	466	378635	5.28E-05	-9.8487	194.977	5.69E-171	4.90E+161	0.959714	0.998648
Cancer	Carc in situ of other sites (cancer register)	T	379101	561	378540	4.93E-05	-9.91846	188.321	2.46E-165	9.88E+155	0.957996	0.998648
Cancer	Benign neoplasm of meninges (cancer register)	T	379101	350	378751	7.50E-05	-9.49746	195.508	2.85E-171	1.97E+162	0.961255	0.998648
Cancer	Neo of oral cavity and digestive (cancer register)	T	379101	341	378760	7.20E-05	-9.53877	196.017	1.01E-171	5.13E+162	0.961188	0.998648
Cancer	Neo of urinary organs (cancer register)	T	379101	723	378378	9.54E-05	-9.25725	116.187	1.20E-103	7.59E+94	0.936495	0.998648
Cancer	Neo of lymphoid haematopoietic (cancer register)	T	379101	292	378809	9.04E-05	-9.31178	195.435	3.97E-171	2.06E+162	0.961998	0.998648
Cancer	Neo of other sites (cancer register)	T	379101	206	378895	0.000109	-9.12172	198.301	1.74E-173	6.85E+164	0.963311	0.998648
Summary	Mal neo of lip oral cavity and pharynx (cancer register)	T	379101	799	378302	8.38E-05	-9.38767	119.648	1.19E-106	5.88E+97	0.937462	0.998648
Summary	Mal neo of digestive organs (cancer register)	T	379101	5957	373144	0.438	-0.825382	1.00505	0.0611	3.14	0.411511	0.998648
Summary	Mal neo of respiratory and intrathoracic organs (cancer register)	T	379101	2026	377075	3.73E-05	-10.1965	116.301	3.75E-104	3.71E+94	0.930136	0.998648
Summary	Melanoma and other mal neo of skin (cancer register)	T	379101	2611	376490	2	0.695261	0.712721	0.496	8.1	0.329311	0.998648
Summary	Mal neo of mesothelial and soft tissue (cancer register)	T	379101	538	378563	4.70E-05	-9.96617	196.294	3.83E-172	5.76E+162	0.959507	0.998648
Summary	Mal neoplasm of breast (cancer register)	T	379101	10158	368943	1.47	0.383264	0.465272	0.589	3.65	0.410086	0.998648
Summary	Mal neo of female genital organs (cancer register)	T	379101	2734	376367	1.08	0.077513	1.00833	0.15	7.8	0.938725	0.998648
Summary	Mal neo of male genital organs (cancer register)	T	379101	7046	372055	1.03	0.025753	0.596185	0.319	3.3	0.965545	0.998648
Summary	Mal neo of urinary tract (cancer register)	T	379101	1919	377182	1.31	0.271644	1.00668	0.182	9.44	0.787282	0.998648
Summary	Mal neo of thyroid and other endocrine glands (cancer register)	T	379101	468	378633	5.61E-05	-9.78811	198.078	1.39E-173	2.27E+164	0.960588	0.998648
Summary	Mal neo of ill secondary unspecified (cancer register)	T	379101	433	378668	5.90E-05	-9.73818	197.188	8.34E-173	4.17E+163	0.960612	0.998648
Summary	Mal neo lymphoid haematopoietic (cancer register)	T	379101	3145	375956	0.83	-0.186345	1.00458	0.116	5.95	0.852841	0.998648
Summary	In situ neo (cancer register)	T	379101	8358	370743	0.332	-1.10248	1.00431	0.0464	2.38	0.272313	0.998648
Summary	Benign neo (cancer register)	T	379101	784	378317	8.49E-05	-9.37375	120.695	1.55E-107	4.64E+98	0.938094	0.998648
Summary	Neo of uncertain or unknown behaviour (cancer register)	T	379101	2117	376984	1.21	0.192562	1.00474	0.169	8.69	0.848015	0.998648
Summary	Neoplasms BIN (cancer register)	T	379101	45225	333876	0.814	-0.205852	0.28377	0.467	1.42	0.468195	0.998648
Neurosciences	Facial pains for 3 months vs no pain	T	368676	3287	365389	5.70E-05	-9.7728	73.2772	2.40E-67	1.35E+58	0.893903	0.998648
Operations and Procedures	Excision of lesion of tissue of brain	T	379101	434	378667	5.50E-05	-9.80741	199.425	9.70E-175	3.12E+165	0.960777	0.998648
Operations and Procedures	Other operations on ventricle of brain	T	379101	227	378874	0.000109	-9.12603	199.041	4.07E-174	2.91E+165	0.96343	0.998648
Operations and Procedures	Extirpation of lesion of meninges of brain	T	379101	289	378812	9.06E-05	-9.30919	197.083	1.57E-172	5.22E+163	0.962326	0.998648
Operations and Procedures	Drainage of subdural space	T	379101	236	378865	0.000108	-9.136	191.71	7.01E-168	1.66E+159	0.961991	0.998648
Operations and Procedures	Other operations on spinal cord	T	379101	218	378883	0.000115	-9.06845	198.811	6.77E-174	1.96E+165	0.963618	0.998648
Operations and Procedures	Therapeutic epidural injection	T	379101	4695	374406	3.89E-05	-10.1543	73.2697	1.67E-67	9.09E+57	0.889776	0.998648
Operations and Procedures	Therapeutic spinal puncture	T	379101	253	378848	0.000101	-9.20442	199.093	3.40E-174	2.98E+165	0.963126	0.998648
Operations and Procedures	Diagnostic spinal puncture	T	379101	4837	374264	1.12	0.110771	0.712366	0.277	4.51	0.876429	0.998648
Operations and Procedures	Operations on spinal nerve root	T	379101	2302	376799	1.16	0.15257	1.00393	0.163	8.33	0.879208	0.998648
Operations and Procedures	Destruction of peripheral nerve	T	379101	243	378858	9.70E-05	-9.24038	198.249	1.71E-173	5.49E+164	0.962824	0.998648
Operations and Procedures	Extirpation of lesion of peripheral nerve	T	379101	1390	377711	5.22E-05	-9.86011	118.991	2.70E-106	1.01E+97	0.93396	0.998648
Operations and Procedures	Microsurgical repair of peripheral nerve	T	379101	222	378879	0.000101	-9.20526	196.001	1.45E-171	6.94E+162	0.962541	0.998648
Operations and Procedures	Other repair of peripheral nerve	T	379101	526	378575	4.87E-05	-9.93076	197.763	2.23E-173	1.06E+164	0.959951	0.998648
Operations and Procedures	Release of entrapment of peripheral nerve at wrist	T	379101	8745	370356	0.304	-1.19069	1.00399	0.0425	2.18	0.23564	0.998648
Operations and Procedures	Release of entrapment of peripheral nerve at other site	T	379101	778	378323	8.37E-05	-9.38806	120.898	1.03E-107	6.81E+98	0.938104	0.998648
Operations and Procedures	Other release of peripheral nerve	T	379101	324	378777	7.56E-05	-9.48953	198.517	7.90E-174	7.24E+164	0.961874	0.998648
Operations and Procedures	Neurostimulation of peripheral nerve	T	379101	545	378556	4.77E-05	-9.95	197.293	5.49E-173	4.15E+163	0.959778	0.998648
Operations and Procedures	Neurophysiological operations	T	379101	2161	376940	8.73E-05	-9.34619	72.9227	7.38E-67	1.03E+58	0.898018	0.998648
Operations and Procedures	Excision of thyroid gland	T	379101	1898	377203	1.5	0.406797	1.00503	0.209	10.8	0.685653	0.998648
Operations and Procedures	Excision of parathyroid gland	T	379101	695	378406	0.000104	-9.17512	117.958	4.05E-105	2.65E+96	0.938	0.998648
Operations and Procedures	Total excision of breast	T	379101	4061	375040	2.19	0.782537	0.589738	0.688	6.95	0.184534	0.998648
Operations and Procedures	Other excision of breast	T	379101	9523	369578	1.24	0.212441	0.515105	0.451	3.39	0.680029	0.998648
Operations and Procedures	Reconstruction of breast	T	379101	1391	377710	5.71E-08	-16.6781	3648.16	0	Inf	0.996352	0.998648
Operations and Procedures	Prosthesis for breast	T	379101	1450	377651	5.88E-05	-9.74162	110.435	5.82E-99	5.94E+89	0.929709	0.998648
Operations and Procedures	Other plastic operations on breast	T	379101	1690	377411	5.43E-05	-9.82109	110.347	6.39E-99	4.61E+89	0.929081	0.998648
Operations and Procedures	Biopsy of breast	T	379101	1566	377535	5.76E-05	-9.76246	110.694	3.43E-99	9.66E+89	0.929723	0.998648
Operations and Procedures	Incision of breast	T	379101	615	378486	5.03E-05	-9.8973	186.17	1.70E-163	1.49E+154	0.957602	0.998648
Operations and Procedures	Operations on duct of breast	T	379101	761	378340	3.87	1.35212	1.00815	0.536	27.9	0.179858	0.998648
Operations and Procedures	Operations on nipple	T	379101	610	378491	5.15E-05	-9.87322	187.963	5.19E-165	5.12E+155	0.958108	0.998648
Operations and Procedures	Reconstruction of nipple and areola	T	379101	637	378464	5.00E-05	-9.90291	181.816	8.60E-160	2.91E+150	0.956563	0.998648
Operations and Procedures	Other operations on breast	T	379101	910	378191	9.71E-05	-9.24024	110.682	5.92E-99	1.59E+90	0.933466	0.998648
Operations and Procedures	Reconstruction of breast using abdominal flap	T	379101	275	378826	1.08E-07	-16.0414	5978.55	0	Inf	0.997859	0.998648
Operations and Procedures	Operations on eyebrow	T	379101	613	378488	0.000112	-9.09931	120.216	5.23E-107	2.39E+98	0.939665	0.998648
Operations and Procedures	Extirpation of lesion of eyelid	T	379101	5517	373584	0.988	-0.012001	0.712516	0.245	3.99	0.986562	0.998648
Operations and Procedures	Excision of redundant skin of eyelid	T	379101	1118	377983	6.49E-05	-9.64338	118.739	5.49E-106	7.67E+96	0.935271	0.998648
Operations and Procedures	Reconstruction of eyelid	T	379101	382	378719	6.77E-05	-9.60077	196.541	3.40E-172	1.35E+163	0.96104	0.998648
Operations and Procedures	Correction of deformity of eyelid	T	379101	816	378285	3.21	1.16599	1.00746	0.445	23.1	0.247126	0.998648
Operations and Procedures	Correction of ptosis of eyelid	T	379101	747	378354	3.53	1.26131	1.00588	0.492	25.4	0.209864	0.998648
Operations and Procedures	Other operations on eyelid	T	379101	931	378170	2.99	1.09569	1.00481	0.417	21.4	0.27552	0.998648
Operations and Procedures	Connection between lacrimal apparatus and nose	T	379101	711	378390	0.000105	-9.15894	118.418	1.67E-105	6.63E+96	0.93835	0.998648
Operations and Procedures	Operations on nasolacrimal duct	T	379101	416	378685	6.69E-05	-9.61261	196.252	5.92E-172	7.56E+162	0.960935	0.998648
Operations and Procedures	Other operations on lacrimal apparatus	T	379101	939	378162	7.76E-05	-9.4637	118.891	4.87E-106	1.24E+97	0.936556	0.998648
Operations and Procedures	Combined operations on muscles of eye	T	379101	370	378731	6.71E-05	-9.60954	199.584	8.66E-175	5.20E+165	0.961598	0.998648
Operations and Procedures	Other adjustment to muscle of eye	T	379101	211	378890	0.000117	-9.05179	199.101	3.90E-174	3.52E+165	0.963738	0.998648
Operations and Procedures	Extirpation of lesion of conjunctiva	T	379101	351	378750	7.18E-05	-9.54164	198.577	6.67E-174	7.73E+164	0.961676	0.998648
Operations and Procedures	Closure of cornea	T	379101	204	378897	0.000122	-9.01187	198.676	9.33E-174	1.59E+165	0.963821	0.998648
Operations and Procedures	Buckling operations for attachment of retina	T	379101	659	378442	3.83	1.34392	1.0053	0.534	27.5	0.181277	0.998648
Operations and Procedures	Filtering operations on iris	T	379101	833	378268	3.19	1.15936	1.006	0.444	22.9	0.249134	0.998648
Operations and Procedures	Other operations on trabecular meshwork of eye	T	379101	236	378865	0.000113	-9.08543	195.404	5.28E-171	2.43E+162	0.962915	0.998648
Operations and Procedures	Incision of iris	T	379101	599	378502	4.24E-05	-10.0677	195.431	1.88E-171	9.59E+161	0.958915	0.998648
Operations and Procedures	Other operations on anterior chamber of eye	T	379101	405	378696	6.25E-05	-9.67956	195.602	1.98E-171	1.98E+162	0.960532	0.998648
Operations and Procedures	Extracapsular extraction of lens	T	379101	17912	361189	1.25	0.222891	0.372165	0.603	2.59	0.549237	0.998648
Operations and Procedures	Prosthesis of lens	T	379101	17908	361193	1.25	0.222107	0.372086	0.602	2.59	0.550559	0.998648
Operations and Procedures	Operations on vitreous body	T	379101	3853	375248	4.73E-05	-9.95839	72.0345	2.28E-66	9.82E+56	0.890047	0.998648
Operations and Procedures	Operations on retinal membrane	T	379101	983	378118	7.23E-05	-9.53462	116.981	1.92E-104	2.73E+95	0.93504	0.998648
Operations and Procedures	Photocoagulation of retina for detachment	T	379101	972	378129	2.66	0.980152	1.00494	0.372	19.1	0.329396	0.998648
Operations and Procedures	Destruction of lesion of retina	T	379101	2270	376831	1.13	0.122189	1.00473	0.158	8.1	0.903205	0.998648
Operations and Procedures	Fixation of retina	T	379101	1034	378067	5.84E-05	-9.74876	119.693	7.61E-107	4.48E+97	0.935086	0.998648
Operations and Procedures	Other operations on eye	T	379101	768	378333	8.96E-05	-9.31995	119.759	1.03E-106	7.82E+97	0.937969	0.998648
Operations and Procedures	Local anaesthetics for ophthalmology procedures	T	379101	5563	373538	1.4	0.337494	0.587952	0.443	4.44	0.565957	0.998648
Operations and Procedures	Extirpation of lesion of external ear	T	379101	1684	377417	3.99E-05	-10.1288	117.198	6.92E-105	2.30E+95	0.931128	0.998648
Operations and Procedures	Plastic operations on external ear	T	379101	299	378802	8.49E-05	-9.37404	194.387	2.91E-170	2.48E+161	0.961538	0.998648
Operations and Procedures	Other operations on external ear	T	379101	435	378666	5.78E-05	-9.75843	196.885	1.48E-172	2.26E+163	0.96047	0.998648
Operations and Procedures	Clearance of external auditory canal	T	379101	325	378776	7.54E-05	-9.49284	198.624	6.38E-174	8.90E+164	0.961881	0.998648
Operations and Procedures	Exenteration of mastoid air cells	T	379101	445	378656	5.74E-05	-9.76527	198.497	6.24E-174	5.29E+164	0.960763	0.998648
Operations and Procedures	Other operations on mastoid	T	379101	342	378759	7.46E-05	-9.50394	198.208	1.43E-173	3.90E+164	0.961757	0.998648
Operations and Procedures	Repair of eardrum	T	379101	1054	378047	6.66E-05	-9.6174	120.787	1.02E-107	4.36E+98	0.936537	0.998648
Operations and Procedures	Drainage of middle ear	T	379101	1751	377350	1.56	0.445882	1.00396	0.218	11.2	0.656953	0.998648
Operations and Procedures	Reconstruction of ossicular chain	T	379101	220	378881	0.000117	-9.05366	198.896	5.81E-174	2.35E+165	0.963693	0.998648
Operations and Procedures	Other operations on ossicle of ear	T	379101	327	378774	7.52E-05	-9.49524	199.486	1.18E-174	4.81E+165	0.962036	0.998648
Operations and Procedures	Other operations on middle ear	T	379101	277	378824	8.84E-05	-9.33338	199.674	9.56E-175	8.18E+165	0.962718	0.998648
Operations and Procedures	Other operations on ear	T	379101	414	378687	5.92E-05	-9.73413	199.012	2.34E-174	1.50E+165	0.960989	0.998648
Operations and Procedures	Plastic operations on nose	T	379101	781	378320	9.07E-05	-9.30768	120.374	3.11E-107	2.64E+98	0.938366	0.998648
Operations and Procedures	Operations on septum of nose	T	379101	4412	374689	1.83	0.606579	0.584769	0.583	5.77	0.299597	0.998648
Operations and Procedures	Surgical arrest of bleeding from internal nose	T	379101	782	378319	3.33	1.20176	1.00567	0.463	23.9	0.232093	0.998648
Operations and Procedures	Packing of cavity of nose	T	379101	1016	378085	2.64	0.971515	1.00539	0.368	19	0.333892	0.998648
Operations and Procedures	Operations on external nose	T	379101	3310	375791	0.813	-0.20742	1.00437	0.113	5.82	0.836386	0.998648
Operations and Procedures	Other operations on nose	T	379101	294	378807	8.64E-05	-9.35687	198.162	1.81E-173	4.12E+164	0.962339	0.998648
Operations and Procedures	Operations on frontal sinus	T	379101	1944	377157	1.38	0.325293	1.00407	0.193	9.91	0.745958	0.998648
Operations and Procedures	Operations on unspecified nasal sinus	T	379101	717	378384	9.82E-05	-9.22802	120.576	2.27E-107	4.25E+98	0.938995	0.998648
Operations and Procedures	Diagnostic endoscopic examination of pharynx	T	379101	3123	375978	1.72	0.543014	0.712866	0.426	6.96	0.446219	0.998648
Operations and Procedures	Other operations on pharynx	T	379101	503	378598	4.90E-05	-9.92378	199.194	1.36E-174	1.77E+165	0.960266	0.998648
Operations and Procedures	Microtherapeutic endoscopic operations on larynx	T	379101	579	378522	4.57E-05	-9.9932	197.503	3.48E-173	6.00E+163	0.959646	0.998648
Operations and Procedures	Other therapeutic endoscopic operations on larynx	T	379101	206	378895	0.000123	-9.00403	197.438	1.06E-172	1.42E+164	0.963626	0.998648
Operations and Procedures	Exteriorisation of trachea	T	379101	817	378284	8.41E-05	-9.38403	118.778	6.59E-106	1.07E+97	0.937029	0.998648
Operations and Procedures	Operations on lower respiratory tract	T	379101	209	378892	0.000132	-8.93309	193.588	2.16E-169	8.05E+160	0.963195	0.998648
Operations and Procedures	Diagnostic endos exam of lwr resp trct rigid brnchscpe	T	379101	452	378649	5.96E-05	-9.72732	195.619	1.82E-171	1.95E+162	0.960341	0.998648
Operations and Procedures	Excision of lung	T	379101	810	378291	9.09E-05	-9.30601	118.17	2.34E-105	3.52E+96	0.93723	0.998648

Operations and Procedures	Other operations on lung	T	379101	801	378300	8.96E-05	-9.32049	118.44	1.36E-105	5.89E+96	0.937276	0.998648
Operations and Procedures	Diagnostic endoscopic examination of mediastinum	T	379101	587	378514	4.63E-05	-9.97982	195.222	3.09E-171	6.95E+161	0.95923	0.998648
Operations and Procedures	Extirpation of lesion of lip	T	379101	1563	377538	1.83	0.602413	1.00421	0.255	13.1	0.548583	0.998648
Operations and Procedures	Other repair of lip	T	379101	211	378890	0.00012	-9.02439	198.589	1.09E-173	1.33E+165	0.963755	0.998648
Operations and Procedures	Other operations on lip	T	379101	464	378637	5.52E-05	-9.80545	198.808	3.26E-174	9.34E+164	0.960663	0.998648
Operations and Procedures	Surgical removal of tooth	T	379101	5408	373693	0.485	-0.722725	1.00374	0.0679	3.47	0.471506	0.998648
Operations and Procedures	Simple extraction of tooth	T	379101	2335	376766	7.90E-05	-9.44656	73.4007	2.62E-67	2.38E+58	0.897596	0.998648
Operations and Procedures	Preprosthetic oral surgery	T	379101	274	378827	9.75E-05	-9.23565	198.494	1.07E-173	8.92E+164	0.962889	0.998648
Operations and Procedures	Surgery on apex of tooth	T	379101	1147	377954	6.38E-05	-9.65988	120.597	1.41E-107	2.88E+98	0.936157	0.998648
Operations and Procedures	Other operations on tooth	T	379101	286	378815	8.95E-05	-9.32172	198.89	4.50E-174	1.78E+165	0.962618	0.998648
Operations and Procedures	Extirpation of lesion of tongue	T	379101	690	378411	0.000101	-9.20507	120.905	1.22E-107	8.29E+98	0.939312	0.998648
Operations and Procedures	Incision of tongue	T	379101	994	378107	6.86E-05	-9.58722	120.84	9.45E-108	4.98E+98	0.936763	0.998648
Operations and Procedures	Extirpation of lesion of palate	T	379101	297	378804	8.87E-05	-9.33053	198.68	6.73E-174	1.17E+165	0.962543	0.998648
Operations and Procedures	Other operations on palate	T	379101	1051	378050	6.48E-05	-9.64354	118.999	3.30E-106	1.28E+97	0.935411	0.998648
Operations and Procedures	Excision of tonsil	T	379101	1168	377933	6.35E-05	-9.66455	118.605	6.98E-106	5.77E+96	0.935056	0.998648
Operations and Procedures	Other operations on tonsil	T	379101	503	378598	4.95E-05	-9.91365	198.619	4.23E-174	5.79E+164	0.960192	0.998648
Operations and Procedures	Extirpation of lesion of other part of mouth	T	379101	688	378413	0.000105	-9.16329	120.515	2.73E-107	4.03E+98	0.939391	0.998648
Operations and Procedures	Other operations on mouth	T	379101	1260	377841	5.72E-05	-9.76881	120.218	2.67E-107	1.23E+98	0.935236	0.998648
Operations and Procedures	Excision of salivary gland	T	379101	935	378166	7.17E-05	-9.54241	120.917	8.49E-108	6.06E+98	0.937099	0.998648
Operations and Procedures	Excision of oesophagus and stomach	T	379101	219	378882	0.00012	-9.02913	190.448	9.25E-167	1.55E+158	0.962187	0.998648
Operations and Procedures	Other therapeutic operations on oesophagus	T	379101	331	378770	7.58E-05	-9.48714	194.571	1.81E-170	3.18E+161	0.961111	0.998648
Operations and Procedures	Diagnostic fibreoptic endoscopic examination of oesophagus	T	379101	1468	377633	4.61E-05	-9.98492	120.287	1.88E-107	1.13E+98	0.933844	0.998648
Operations and Procedures	Other operations on oesophagus	T	379101	1839	377262	1.55	0.437119	1.00409	0.216	11.1	0.663316	0.998648
Operations and Procedures	Repair of diaphragmatic hernia	T	379101	276	378825	9.06E-05	-9.30889	198.656	7.21E-174	1.14E+165	0.962625	0.998648
Operations and Procedures	Antireflux operations	T	379101	820	378281	8.29E-05	-9.39749	121.062	7.39E-108	9.31E+98	0.938126	0.998648
Operations and Procedures	Other connection of stomach to jejunum	T	379101	256	378845	0.000106	-9.15058	197.751	4.98E-173	2.26E+164	0.963093	0.998648
Operations and Procedures	Artificial opening into stomach	T	379101	356	378745	7.06E-05	-9.55835	196.041	9.45E-172	5.28E+162	0.961113	0.998648
Operations and Procedures	Extirpation of lesion of upper gastrointestinal tract	T	379101	1015	378086	2.67	0.98182	1.00565	0.372	19.2	0.328915	0.998648
Operations and Procedures	Therapeutic fibreoptic gastrointestinal tract	T	379101	1244	377857	5.49E-05	-9.81003	119.568	9.15E-107	3.30E+97	0.93461	0.998648
Operations and Procedures	Examination of upper gastrointestinal tract	T	379101	65264	313837	0.91	-0.093923	0.233427	0.576	1.44	0.687414	0.998648
Operations and Procedures	Intubation of stomach	T	379101	329	378772	8.37E-05	-9.38787	194.59	1.93E-170	3.64E+161	0.961522	0.998648
Operations and Procedures	Artificial opening into jejunum	T	379101	294	378807	8.57E-05	-9.36458	194.078	5.38E-170	1.37E+161	0.961516	0.998648
Operations and Procedures	Excision of ileum	T	379101	732	378369	3.77	1.32688	1.00481	0.526	27	0.186659	0.998648
Operations and Procedures	Creation of artificial opening into ileum	T	379101	1023	378078	2.57	0.944013	1.00484	0.359	18.4	0.347493	0.998648
Operations and Procedures	Attention to artificial opening into ileum	T	379101	998	378103	2.61	0.958374	1.00478	0.364	18.7	0.340178	0.998648
Operations and Procedures	Emergency excision of appendix	T	379101	2570	376531	7.00E-05	-9.56688	73.3144	2.75E-67	1.78E+58	0.896178	0.998648
Operations and Procedures	Total excision of colon and rectum	T	379101	212	378889	0.00012	-9.03085	197.78	5.30E-173	2.70E+164	0.96358	0.998648
Operations and Procedures	Extended excision of right hemicolon	T	379101	250	378851	0.000101	-9.19899	194.321	3.94E-170	2.60E+161	0.962243	0.998648
Operations and Procedures	Other excision of right hemicolon	T	379101	1421	377680	4.85E-05	-9.93416	119.333	1.28E-106	1.84E+97	0.933655	0.998648
Operations and Procedures	Excision of left hemicolon	T	379101	330	378771	8.21E-05	-9.40756	193.43	1.83E-169	3.68E+160	0.96121	0.998648
Operations and Procedures	Other excision of colon	T	379101	242	378859	0.000103	-9.18372	197.606	6.40E-173	1.65E+164	0.962932	0.998648
Operations and Procedures	Other exteriorisation of colon	T	379101	934	378167	2.91	1.0672	1.00498	0.406	20.8	0.288272	0.998648
Operations and Procedures	Endoscopic extirpation of lesion of colon	T	379101	14469	364632	1.33	0.283029	0.390943	0.617	2.86	0.469088	0.998648
Operations and Procedures	Diagnostic endoscopic examination of colon	T	379101	48889	330212	0.881	-0.127092	0.266608	0.522	1.49	0.633575	0.998648
Operations and Procedures	Endoscopic of lower bowel	T	379101	3536	375565	5.19E-05	-9.86578	72.359	1.32E-66	2.04E+57	0.891549	0.998648
Operations and Procedures	Examination of sigmoid colon	T	379101	2690	376411	6.93E-05	-9.57668	73.1253	3.94E-67	1.22E+58	0.895805	0.998648
Operations and Procedures	Excision of rectum	T	379101	2290	376811	8.13E-05	-9.41722	72.1698	3.01E-66	2.20E+57	0.896181	0.998648
Operations and Procedures	Fixation of rectum for prolapse	T	379101	293	378808	9.67E-05	-9.24348	187.941	1.02E-164	9.21E+155	0.960774	0.998648
Operations and Procedures	Other operations on rectum through anus	T	379101	648	378453	0.000102	-9.18831	119.873	9.37E-107	1.12E+98	0.938902	0.998648
Operations and Procedures	Perineal operations for prolapse of rectum	T	379101	257	378844	0.000106	-9.15606	195.907	1.84E-171	6.07E+162	0.962723	0.998648
Operations and Procedures	Manipulation of rectum	T	379101	1760	377341	3.84E-05	-10.1665	120.949	4.27E-108	3.46E+98	0.933012	0.998648
Operations and Procedures	Other operations on rectum	T	379101	894	378207	8.86E-05	-9.3314	118.813	6.48E-106	1.21E+97	0.9374	0.998648
Operations and Procedures	Excision of lesion of anus	T	379101	2708	376393	1.99	0.688376	0.712438	0.493	8.04	0.333931	0.998648
Operations and Procedures	Destruction of haemorrhoid	T	379101	5384	373717	3.48E-05	-10.2659	73.2182	1.65E-67	7.35E+57	0.888495	0.998648
Operations and Procedures	Other operations on haemorrhoid	T	379101	276	378825	8.47E-05	-9.37609	197.718	4.24E-173	1.69E+164	0.962177	0.998648
Operations and Procedures	Dilation of anal sphincter	T	379101	444	378657	6.09E-05	-9.70622	198.801	3.65E-174	1.02E+165	0.96106	0.998648
Operations and Procedures	Other operations on perianal region	T	379101	1153	377948	5.77E-05	-9.75973	119.776	6.40E-107	5.21E+97	0.935058	0.998648
Operations and Procedures	Other operations on anus	T	379101	1643	377458	4.09E-05	-10.1048	121.146	3.09E-108	5.41E+98	0.933525	0.998648
Operations and Procedures	Drainage through perineal region	T	379101	1052	378049	6.53E-05	-9.63716	119.268	1.96E-106	2.18E+97	0.935599	0.998648
Operations and Procedures	Excision of pilonidal sinus	T	379101	253	378848	0.000105	-9.15736	191.993	3.94E-168	2.82E+159	0.961958	0.998648
Operations and Procedures	Other operations on pilonidal sinus	T	379101	206	378895	5.18E-05	-9.86828	314.749	6.23E-273	4.31E+263	0.974988	0.998648
Operations and Procedures	Other operations on bowel	T	379101	379	378722	7.29E-05	-9.52703	197.511	5.47E-173	9.71E+163	0.961529	0.998648
Operations and Procedures	Other puncture of liver	T	379101	403	378698	6.21E-05	-9.68716	199.223	1.63E-174	2.37E+165	0.961218	0.998648
Operations and Procedures	Other open operations on bile duct	T	379101	1260	377841	2.3	0.832438	1.00485	0.321	16.5	0.407432	0.998648
Operations and Procedures	Endoscopic incision of sphincter of Oddi	T	379101	1801	377300	3.97E-05	-10.1344	119.344	1.03E-106	1.54E+97	0.932326	0.998648
Operations and Procedures	Endoscopic retrograde placement of prosthesis in bile duct	T	379101	560	378541	4.64E-05	-9.97721	195.611	1.44E-171	1.49E+162	0.959321	0.998648
Operations and Procedures	Other therapeutic operations on bile duct	T	379101	284	378817	9.28E-05	-9.28532	196.342	6.88E-172	1.25E+163	0.962281	0.998648
Operations and Procedures	Examination of bile duct and pancreatic duct	T	379101	2192	376909	8.88E-05	-9.32955	72.433	1.96E-66	4.02E+57	0.897514	0.998648
Operations and Procedures	Diagnostic endoscopic retrograde examination of bile duct	T	379101	309	378792	8.61E-05	-9.35971	196.801	2.60E-172	2.85E+163	0.962067	0.998648
Operations and Procedures	Diagnostic retrograde exam pancreatic duct	T	379101	208	378893	0.000126	-8.97881	196.16	1.34E-171	1.19E+163	0.963491	0.998648
Operations and Procedures	Endoscopic ultrasound examination of bile duct	T	379101	228	378873	0.000117	-9.05018	197.201	1.62E-172	8.52E+163	0.963395	0.998648
Operations and Procedures	Total excision of spleen	T	379101	281	378820	8.56E-05	-9.36529	198.431	1.06E-173	6.93E+164	0.962357	0.998648
Operations and Procedures	Plastic repair of mitral valve	T	379101	528	378573	4.87E-05	-9.92975	192.812	3.65E-169	6.49E+159	0.958927	0.998648
Operations and Procedures	Plastic repair of aortic valve	T	379101	1074	378027	2.44	0.891593	1.00697	0.339	17.6	0.375931	0.998648
Operations and Procedures	Other open operations on valve of heart	T	379101	301	378800	8.49E-05	-9.37432	192.038	2.90E-168	2.48E+159	0.961067	0.998648
Operations and Procedures	Saphenous vein graft replacement of coronary artery	T	379101	3223	375878	6.30E-05	-9.67267	67.5264	2.09E-62	1.90E+53	0.886098	0.998648
Operations and Procedures	Other autograft replacement of coronary artery	T	379101	496	378605	5.36E-05	-9.83429	184.318	6.83E-162	4.20E+152	0.957449	0.998648
Operations and Procedures	Connection of thoracic artery to coronary artery	T	379101	3408	375693	5.88E-05	-9.74055	67.5104	2.01E-62	1.72E+53	0.885278	0.998648
Operations and Procedures	Transluminal balloon angioplasty of coronary artery	T	379101	2913	376188	6.79E-05	-9.5981	69.0828	1.06E-63	4.33E+54	0.889501	0.998648
Operations and Procedures	Other therapeutic transluminal operations on coronary artery	T	379101	858	378243	7.77E-05	-9.46211	114.536	2.49E-102	2.43E+93	0.93416	0.998648
Operations and Procedures	Diagnostic transluminal operations on coronary artery	T	379101	808	378293	8.76E-05	-9.34329	117.315	1.21E-104	6.35E+95	0.936521	0.998648
Operations and Procedures	Transluminal heart assist operations	T	379101	275	378826	9.65E-05	-9.24635	190.199	1.21E-166	7.67E+157	0.961227	0.998648
Operations and Procedures	Other therapeutic transluminal operations on heart	T	379101	1372	377729	1.89	0.636257	1.00431	0.264	13.5	0.526388	0.998648
Operations and Procedures	Diagnostic transluminal operations on heart	T	379101	1107	377994	5.94E-05	-9.73109	120.188	2.94E-107	1.20E+98	0.935469	0.998648
Operations and Procedures	Cardioverter defibrillator introduced through the vein	T	379101	599	378502	4.32E-05	-10.0508	189.073	4.93E-166	3.78E+156	0.957606	0.998648
Operations and Procedures	Cardiac pacemaker system introduced through vein	T	379101	2286	376815	1.14	0.135174	1.00679	0.159	8.24	0.893195	0.998648
Operations and Procedures	Other cardiac pacemaker system	T	379101	1141	377960	5.99E-05	-9.72283	115.758	1.75E-103	2.05E+94	0.933062	0.998648
Operations and Procedures	Therapeutic transluminal operations on heart	T	379101	862	378239	7.72E-05	-9.46909	118.299	1.55E-105	3.85E+96	0.936202	0.998648
Operations and Procedures	Contrast radiology of heart	T	379101	21382	357719	0.597	-0.516444	0.459947	0.242	1.47	0.261508	0.998648
Operations and Procedures	Catheterisation of heart	T	379101	776	378325	8.98E-05	-9.31771	117.591	7.21E-105	1.12E+96	0.936843	0.998648
Operations and Procedures	Other operations on heart	T	379101	733	378368	3.56	1.26864	1.00453	0.496	25.5	0.206617	0.998648
Operations and Procedures	Percutaneous transluminal balloon	T	379101	5594	373507	0.449	-0.801083	1.00626	0.0625	3.23	0.425977	0.998648
Operations and Procedures	Other replacement of aneurysmal segment of aorta	T	379101	295	378806	9.84E-05	-9.22642	182.772	2.60E-160	3.73E+151	0.95974	0.998648
Operations and Procedures	Transluminal operations on aorta	T	379101	858	378243	3.17	1.15305	1.00764	0.44	22.8	0.252493	0.998648
Operations and Procedures	Reconstruction of carotid artery	T	379101	506	378595	5.48E-05	-9.8126	187.541	1.26E-164	2.38E+155	0.958272	0.998648
Operations and Procedures	Operations on aneurysm of cerebral artery	T	379101	201	378900	0.000138	-8.88855	197.738	6.63E-173	2.87E+164	0.964146	0.998648
Operations and Procedures	Transluminal operations on cerebral artery	T	379101	1001	378100	2.74	1.00855	1.00425	0.383	19.6	0.315243	0.998648
Operations and Procedures	Transluminal operations on renal artery	T	379101	241	378860	0.000101	-9.20416	198.175	2.05E-173	4.93E+164	0.962956	0.998648
Operations and Procedures	Other bypass of femoral artery	T	379101	274	378827	0.000106	-9.15284	184.902	4.30E-162	2.61E+153	0.96052	0.998648
Operations and Procedures	Reconstruction of femoral artery	T	379101	231	378870	4.68E-05	-9.9702	299.197	9.74E-260	2.25E+250	0.973417	0.998648
Operations and Procedures	Transluminal operations on femoral artery	T	379101	1076	378025	6.86E-05	-9.58674	114.98	9.20E-103	5.12E+93	0.933552	0.998648
Operations and Procedures	Excision of other artery	T	379101	498	378603	5.54E-05	-9.80098	190.3	5.71E-167	5.37E+157	0.958925	0.998648
Operations and Procedures	Other open operations on other artery	T	379101	320	378781	7.40E-05	-9.51094	198.504	7.93E-174	6.91E+164	0.961785	0.998648
Operations and Procedures	Therapeutic transluminal operations on other artery	T	379101	774	378327	9.00E-05	-9.31554	120.903	1.10E-107	7.40E+98	0.938584	0.998648
Operations and Procedures	Arteriovenous shunt	T	379101	297	378804	8.29E-05	-9.3979	196.497	4.54E-172	1.51E+163	0.961854	0.998648
Operations and Procedures	Endovascular placement of stent	T	379101	370	378731	7.09E-05	-9.55478	190.927	2.14E-167	2.35E+158	0.960087	0.998648
Operations and Procedures	Other operations on vena cava	T	379101	207	378894	0.000114	-9.076	197.819	4.69E-173	2.79E+164	0.963406	0.998648
Operations and Procedures	Combined operations on varicose vein of leg	T	379101	1623	377478	1.72	0.542986	1.004	0.241	12.3	0.588629	0.998648
Operations and Procedures	Ligation of varicose vein of leg	T	379101	5765	373336	1.5	0.404823	0.584624	0.477	4.71	0.488654	0.998648
Operations and Procedures	Injection into varicose vein of leg	T	379101	823	378278	9.53E-05	-9.25887	119.38	2.29E-106	3.96E+97	0.93818	0.998648
Operations and Procedures	Other operations on varicose vein of leg	T	379101	6971	372130	1.24	0.211123	0.584601	0.393	3.88	0.717995	0.998648
Operations and Procedures	Transluminal operations on varicose vein of leg	T	379101	1198	377903	5.94E-05	-9.73177	120.789	9.03E-108	3.90E+98	0.935785	0.998648
Operations and Procedures	Other vein related operations	T	379101	4293	374808	1.86	0.621912	0.584344	0.593	5.85	0.287197	0.998648
Operations and Procedures	Therapeutic transluminal operations on vein	T	379101	314	378787	7.23E-05	-9.53464	198.95	3.23E-174	1.62E+165	0.961776	0.998648
Operations and Procedures	Diagnostic transluminal operations on vein	T	379101	931	378170	7.43E-05	-9.50706	120.413	2.36E-107	2.34E+98	0.937069	0.998648

Operations and Procedures	Other therapeutic transluminal operations on vein	T	379101	1371	377730	1.83	0.604975	1.00429	0.256	13.1	0.546914	0.998648
Operations and Procedures	Transplantation of kidney	T	379101	266	378835	9.08E-05	-9.30729	197.834	3.62E-173	2.28E+164	0.962477	0.998648
Operations and Procedures	Total excision of kidney	T	379101	1063	378038	6.32E-05	-9.66962	119.635	9.23E-107	4.32E+97	0.93558	0.998648
Operations and Procedures	Therapeutic endoscopic operations on calculus of kidney	T	379101	417	378684	5.95E-05	-9.72893	196.641	2.46E-172	1.44E+163	0.96054	0.998648
Operations and Procedures	Percutaneous puncture of kidney	T	379101	1437	377664	4.63E-05	-9.97998	120.048	3.01E-107	7.12E+97	0.933746	0.998648
Operations and Procedures	Extracorporeal fragmentation of calculus of kidney	T	379101	1421	377680	1.78	0.576125	1.00485	0.248	12.8	0.566413	0.998648
Operations and Procedures	Other operations on kidney	T	379101	281	378820	9.18E-05	-9.29576	197.132	1.45E-172	5.82E+163	0.96239	0.998648
Operations and Procedures	Urinary diversion	T	379101	327	378774	7.83E-05	-9.4553	193.641	1.16E-169	5.30E+160	0.961056	0.998648
Operations and Procedures	Therapeutic ureteroscopic operations on ureter	T	379101	1338	377763	5.01E-05	-9.90126	119.369	1.23E-106	2.04E+97	0.933894	0.998648
Operations and Procedures	Other endoscopic removal of calculus from ureter	T	379101	242	378859	0.000101	-9.19546	196.735	3.49E-172	2.96E+163	0.96272	0.998648
Operations and Procedures	Other therapeutic endoscopic operations on ureter	T	379101	1932	377169	9.27E-05	-9.28645	72.9141	7.97E-67	1.08E+58	0.898654	0.998648
Operations and Procedures	Diagnostic endoscopic examination of ureter	T	379101	1627	377474	1.61	0.478645	1.00441	0.225	11.6	0.633689	0.998648
Operations and Procedures	Extracorporeal fragmentation of calculus of ureter	T	379101	563	378538	4.20E-05	-10.0781	193.821	4.36E-170	4.04E+160	0.958531	0.998648
Operations and Procedures	Total excision of bladder	T	379101	315	378786	8.14E-05	-9.41617	190.968	2.27E-167	2.92E+158	0.960674	0.998648
Operations and Procedures	Other repair of bladder	T	379101	216	378885	0.000123	-9.00324	198.186	2.46E-173	6.16E+164	0.963766	0.998648
Operations and Procedures	Endoscopic extirpation of lesion of bladder	T	379101	2389	376712	8.09E-05	-9.42286	70.1885	1.45E-64	4.50E+55	0.893204	0.998648
Operations and Procedures	Endoscopic operations to increase capacity of bladder	T	379101	1067	378034	7.15E-05	-9.54628	118.755	5.86E-106	8.72E+96	0.93593	0.998648
Operations and Procedures	Other therapeutic endoscopic operations on bladder	T	379101	557	378544	4.57E-05	-9.99407	192.51	6.19E-169	3.37E+159	0.958597	0.998648
Operations and Procedures	Diagnostic endoscopic examination of bladder	T	379101	30109	348992	1.06	0.059458	0.304113	0.585	1.93	0.844993	0.998648
Operations and Procedures	Other operations on bladder	T	379101	1638	377463	1.6	0.468659	1.00512	0.223	11.5	0.64102	0.998648
Operations and Procedures	Abdominal operations to support outlet of female bladder	T	379101	1021	378080	8.57E-08	-16.2727	3625.47	0	Inf	0.996419	0.998648
Operations and Procedures	Vaginal operations to support outlet of female bladder	T	379101	3234	375867	6.82E-08	-16.5002	2210.83	0	Inf	0.994045	0.998648
Operations and Procedures	Therapeutic on outlet of female bladder	T	379101	299	378802	0.000112	-9.09286	182.002	1.34E-159	9.42E+150	0.960154	0.998648
Operations and Procedures	Open excision of prostate	T	379101	2046	377055	1.14	0.132379	1.0108	0.157	8.28	0.895803	0.998648
Operations and Procedures	Endoscopic resection of outlet of male bladder	T	379101	3901	375200	0.634	-0.456466	1.01268	0.087	4.61	0.652169	0.998648
Operations and Procedures	Other therapeutic on outlet of male bladder	T	379101	850	378251	2.95	1.08033	1.0102	0.407	21.3	0.284876	0.998648
Operations and Procedures	Other operations on outlet of male bladder	T	379101	6418	372683	1.56	0.445483	0.521329	0.562	4.34	0.392821	0.998648
Operations and Procedures	Therapeutic endoscopic operations on urethra	T	379101	2234	376867	8.13E-05	-9.41785	73.1155	4.71E-67	1.40E+58	0.89751	0.998648
Operations and Procedures	Diagnostic endoscopic examination of urethra	T	379101	369	378732	6.76E-05	-9.6026	190.288	7.13E-167	6.40E+157	0.959753	0.998648
Operations and Procedures	Other operations on urethra	T	379101	1489	377612	4.79E-05	-9.94562	120.029	3.24E-107	7.10E+97	0.933963	0.998648
Operations and Procedures	Operations on urethral orifice	T	379101	464	378637	5.35E-05	-9.83573	191.839	2.70E-168	1.06E+159	0.95911	0.998648
Operations and Procedures	Other operations on scrotum	T	379101	203	378898	4.36E-08	-16.9477	9907.79	0	Inf	0.998635	0.998648
Operations and Procedures	Other excision of testis	T	379101	508	378593	4.89E-08	-16.8326	6003.28	0	Inf	0.997763	0.998648
Operations and Procedures	Operations on hydrocele sac	T	379101	1029	378072	6.64E-08	-16.528	3643.01	0	Inf	0.99638	0.998648
Operations and Procedures	Operations on epididymis	T	379101	1069	378032	2.32	0.843436	1.00797	0.322	16.8	0.402725	0.998648
Operations and Procedures	Excision of vas deferens	T	379101	4286	374815	1.81	0.593321	0.606944	0.551	5.95	0.328295	0.998648
Operations and Procedures	Extirpation of lesion of penis	T	379101	221	378880	4.10E-08	-17.0106	9928.78	0	Inf	0.998633	0.998648
Operations and Procedures	Plastic operations on penis	T	379101	394	378707	6.28E-08	-16.5835	6024.54	0	Inf	0.997804	0.998648
Operations and Procedures	Operations on prepuce	T	379101	2323	376778	2.87E-08	-17.3681	3651.94	0	Inf	0.996205	0.998648
Operations and Procedures	Other operations on penis	T	379101	452	378649	5.62E-08	-16.6945	5965.72	0	Inf	0.997767	0.998648
Operations and Procedures	Other upper digestive tract	T	379101	488	378613	4.87E-05	-9.92945	197.272	5.84E-173	4.06E+163	0.959856	0.998648
Operations and Procedures	Branch of external carotid artery	T	379101	271	378830	1.00E-04	-9.20657	190.771	4.11E-167	2.45E+158	0.961509	0.998648
Operations and Procedures	Other leg region	T	379101	287	378814	9.70E-05	-9.24121	194.81	1.45E-170	6.49E+161	0.962165	0.998648
Operations and Procedures	Other lymph node	T	379101	2930	376171	0.962	-0.038861	1.00664	0.134	6.92	0.969205	0.998648
Operations and Procedures	Body region	T	379101	6404	372697	1.66	0.506208	0.508393	0.612	4.49	0.319395	0.998648
Operations and Procedures	Excision of bone	T	379101	3651	375450	0.717	-0.332121	1.0036	0.1	5.13	0.740697	0.998648
Operations and Procedures	Operations on Bartholin gland	T	379101	599	378502	5.13E-08	-16.7853	5974.92	0	Inf	0.997759	0.998648
Operations and Procedures	Excision of vulva	T	379101	1147	377954	2.64	0.970377	1.00797	0.366	19	0.335695	0.998648
Operations and Procedures	Other operations on vulva	T	379101	1300	377801	6.62E-08	-16.5305	3636.81	0	Inf	0.996373	0.998648
Operations and Procedures	Other operations on female perineum	T	379101	887	378214	8.98E-08	-16.2261	3651.73	0	Inf	0.996455	0.998648
Operations and Procedures	Extirpation of lesion of vagina	T	379101	675	378426	4.42E-08	-16.9353	6018.89	0	Inf	0.997755	0.998648
Operations and Procedures	Other repair of prolapse of vagina	T	379101	7298	371803	3.02E-08	-17.3141	2175.08	0	Inf	0.993649	0.998648
Operations and Procedures	Repair of vault of vagina	T	379101	1152	377949	7.65E-08	-16.3863	3589.57	0	Inf	0.996358	0.998648
Operations and Procedures	Other repair of vagina	T	379101	332	378769	9.17E-08	-16.2051	6003.23	0	Inf	0.997846	0.998648
Operations and Procedures	Exploration of vagina	T	379101	1868	377233	4.42E-08	-16.9355	3624.09	0	Inf	0.996271	0.998648
Operations and Procedures	Other operations on vagina	T	379101	544	378557	5.46E-08	-16.7241	6015.45	0	Inf	0.997782	0.998648
Operations and Procedures	Excision of cervix uteri	T	379101	3167	375934	6.86E-08	-16.4947	2208.72	0	Inf	0.994041	0.998648
Operations and Procedures	Destruction of lesion of cervix uteri	T	379101	2136	376965	3.93E-08	-17.0532	3628.7	0	Inf	0.99625	0.998648
Operations and Procedures	Biopsy of cervix uteri	T	379101	1749	377352	4.65E-08	-16.8844	3629.55	0	Inf	0.996288	0.998648
Operations and Procedures	Other operations on cervix uteri	T	379101	247	378854	1.31E-07	-15.8444	5991.08	0	Inf	0.99789	0.998648
Operations and Procedures	Abdominal excision of uterus	T	379101	9231	369870	6.53E-08	-16.5447	1332.72	0	Inf	0.990095	0.998648
Operations and Procedures	Vaginal excision of uterus	T	379101	4716	374385	4.59E-08	-16.8961	2201.81	0	Inf	0.993877	0.998648
Operations and Procedures	Other open operations on uterus	T	379101	285	378816	1.16E-07	-15.9722	5897.56	0	Inf	0.997839	0.998648
Operations and Procedures	Curettage of uterus	T	379101	6842	372259	1.4	0.339831	0.592123	0.44	4.48	0.566022	0.998648
Operations and Procedures	Other evacuation of contents of uterus	T	379101	2583	376518	5.80E-05	-9.75549	102.326	4.59E-92	7.33E+82	0.924047	0.998648
Operations and Procedures	Intrauterine contraceptive device	T	379101	5122	373979	1.24	0.215659	0.722507	0.301	5.11	0.765331	0.998648
Operations and Procedures	Introduction of abortifacient into uterine cavity	T	379101	623	378478	9.76E-08	-16.142	5461.54	0	Inf	0.997642	0.998648
Operations and Procedures	Other vaginal operations on uterus	T	379101	1552	377549	1.9	0.63972	1.01106	0.261	13.8	0.526916	0.998648
Operations and Procedures	Therapeutic endoscopic operations on uterus	T	379101	6827	372274	1.28	0.24871	0.59037	0.403	4.08	0.673552	0.998648
Operations and Procedures	Diagnostic endoscopic examination of uterus	T	379101	20331	358770	0.998	-0.001677	0.398319	0.457	2.18	0.996641	0.998648
Operations and Procedures	Other operations on uterus	T	379101	1103	377998	2.51	0.919488	1.00851	0.347	18.1	0.361912	0.998648
Operations and Procedures	Bilateral excision of adnexa of uterus	T	379101	8743	370358	6.75E-08	-16.5112	1334.29	0	Inf	0.990127	0.998648
Operations and Procedures	Unilateral excision of adnexa of uterus	T	379101	1862	377239	4.66E-08	-16.8808	3606.22	0	Inf	0.996265	0.998648
Operations and Procedures	Other excision of adnexa of uterus	T	379101	224	378877	4.82E-08	-16.8481	9818.83	0	Inf	0.998631	0.998648
Operations and Procedures	Endoscopic bilateral occlusion of fallopian tubes	T	379101	2687	376414	4.57E-08	-16.9003	3469.03	0	Inf	0.996113	0.998648
Operations and Procedures	Diagnostic endoscopic examination of fallopian tube	T	379101	293	378808	6.87E-08	-16.4928	9376.31	0	Inf	0.998597	0.998648
Operations and Procedures	Partial excision of ovary	T	379101	433	378668	8.76E-08	-16.2502	5877.98	0	Inf	0.997794	0.998648
Operations and Procedures	Therapeutic endoscopic operations on ovary	T	379101	717	378384	4.85E-08	-16.8427	5838.83	0	Inf	0.997698	0.998648
Operations and Procedures	Other examination of female genital tract	T	379101	3998	375103	1.52	0.41764	0.71785	0.372	6.2	0.560706	0.998648
Operations and Procedures	Other operations on amniotic cavity	T	379101	308	378793	5.06E-08	-16.7997	9097.69	0	Inf	0.998527	0.998648
Operations and Procedures	Surgical induction of labour	T	379101	2296	376805	8.05E-08	-16.3347	3184.06	0	Inf	0.995907	0.998648
Operations and Procedures	Other induction of labour	T	379101	2732	376369	6.21E-08	-16.5944	3188.66	0	Inf	0.995848	0.998648
Operations and Procedures	Elective caesarean delivery	T	379101	1697	377404	9.19E-08	-16.2029	3246.12	0	Inf	0.996017	0.998648
Operations and Procedures	Other caesarean delivery	T	379101	1946	377155	8.40E-08	-16.2919	3219	0	Inf	0.995962	0.998648
Operations and Procedures	Forceps cephalic delivery	T	379101	705	378396	8.80E-08	-16.2463	5307.56	0	Inf	0.997558	0.998648
Operations and Procedures	Vacuum delivery	T	379101	1205	377896	5.08E-08	-16.7944	5291.17	0	Inf	0.997467	0.998648
Operations and Procedures	Normal delivery	T	379101	6515	372586	0.649	-0.431911	1.03481	0.0854	4.93	0.6764	0.998648
Operations and Procedures	Other operations to facilitate delivery	T	379101	2119	376982	7.78E-08	-16.3691	3204.11	0	Inf	0.995924	0.998648
Operations and Procedures	Removal of products of delivered uterus	T	379101	339	378762	6.21E-08	-16.5953	8831.07	0	Inf	0.998501	0.998648
Operations and Procedures	Other operations on delivered uterus	T	379101	225	378876	9.65E-08	-16.154	8912.07	0	Inf	0.998554	0.998648
Operations and Procedures	Repair of obstetric laceration	T	379101	3617	375484	1.27	0.236602	1.03527	0.167	9.64	0.819226	0.998648
Operations and Procedures	Plastic excision of skin of head or neck	T	379101	208	378893	0.000121	-9.02025	198.304	1.92E-173	7.63E+164	0.963719	0.998648
Operations and Procedures	Plastic excision of skin of abdominal wall	T	379101	228	378873	0.000144	-8.84251	189.335	9.88E-166	2.11E+157	0.96275	0.998648
Operations and Procedures	Other excision of skin	T	379101	205	378896	0.000137	-8.89907	198.083	3.34E-173	5.58E+164	0.964166	0.998648
Operations and Procedures	Microscopically controlled excision of lesion of skin	T	379101	920	378181	7.09E-05	-9.55374	119.018	3.47E-106	1.45E+97	0.936021	0.998648
Operations and Procedures	Other excision of lesion of skin	T	379101	31445	347656	0.665	-0.408006	0.364251	0.326	1.36	0.262662	0.998648
Operations and Procedures	Curettage of lesion of skin	T	379101	1788	377313	3.69E-05	-10.2065	119.575	6.07E-107	2.25E+97	0.931978	0.998648
Operations and Procedures	Photodestruction of lesion of skin	T	379101	418	378683	6.43E-05	-9.65167	195.946	1.04E-171	3.99E+162	0.960715	0.998648
Operations and Procedures	Other destruction of lesion of skin of head or neck	T	379101	325	378776	7.47E-05	-9.50195	198.658	5.92E-174	9.43E+164	0.961851	0.998648
Operations and Procedures	Other destruction of lesion of skin of other site	T	379101	282	378819	8.81E-05	-9.33708	198.13	1.96E-173	3.95E+164	0.962413	0.998648
Operations and Procedures	Punch biopsy of skin	T	379101	3326	375775	1.66	0.506625	0.713418	0.41	6.72	0.477618	0.998648
Operations and Procedures	Shave biopsy of skin	T	379101	626	378475	0.000106	-9.15579	120.289	4.28E-107	2.60E+98	0.939328	0.998648
Operations and Procedures	Other biopsy of skin	T	379101	1597	377504	1.67	0.514776	1.00435	0.234	12	0.608269	0.998648
Operations and Procedures	Flap operations to relax contracture of skin	T	379101	656	378445	0.000102	-9.18606	118.985	5.35E-106	1.96E+97	0.938462	0.998648
Operations and Procedures	Other local flap of skin	T	379101	1956	377145	1.39	0.327489	1.005	0.194	9.95	0.744529	0.998648
Operations and Procedures	Split autograft of skin	T	379101	1318	377783	1.99	0.687173	1.00461	0.278	14.2	0.493962	0.998648
Operations and Procedures	Other autograft of skin	T	379101	1822	377279	1.44	0.367112	1.00533	0.201	10.4	0.714988	0.998648
Operations and Procedures	Suture of skin of head or neck	T	379101	774	378327	8.59E-05	-9.36229	119.945	6.84E-107	1.08E+98	0.937784	0.998648
Operations and Procedures	Suture of skin of other site	T	379101	2266	376835	7.82E-05	-9.45667	73.0057	5.61E-67	1.09E+58	0.896936	0.998648
Operations and Procedures	Removal of other inorganic substance from skin	T	379101	431	378670	5.95E-05	-9.72907	198.708	4.28E-174	8.29E+164	0.96095	0.998648
Operations and Procedures	Opening of skin	T	379101	3649	375452	1.47	0.385143	0.712447	0.364	5.94	0.588789	0.998648
Operations and Procedures	Insertion of skin expander into subcutaneous tissue	T	379101	446	378655	7.19E-05	-9.54069	183.324	6.43E-161	8.04E+151	0.958495	0.998648
Operations and Procedures	Attention to skin expander in subcutaneous tissue	T	379101	431	378670	7.42E-05	-9.50928	183.843	2.40E-161	2.29E+152	0.958748	0.998648
Operations and Procedures	Introduction of therapeutic substance into subcutaneous tissue	T	379101	610	378491	4.67E-05	-9.97182	194.477	1.34E-170	1.63E+161	0.959106	0.998648
Operations and Procedures	Introduction of substance into skin	T	379101	217	378884	0.00011	-9.11202	198.702	8.02E-174	1.52E+165	0.963424	0.998648

Operations and Procedures	Exploration of other skin of head or neck	T	379101	445	378656	5.55E-05	-9.79989	197.176	8.02E-173	3.83E+163	0.96036	0.998648
Operations and Procedures	Exploration of other skin of other site	T	379101	4464	374637	1.15	0.140371	0.712729	0.285	4.65	0.843868	0.998648
Operations and Procedures	Other operations on skin	T	379101	1061	378040	7.20E-05	-9.53942	118.9	4.44E-106	1.17E+97	0.936054	0.998648
Operations and Procedures	Other operations on subcutaneous tissue	T	379101	494	378607	5.68E-05	-9.77547	196.548	2.82E-172	1.15E+163	0.960333	0.998648
Operations and Procedures	Other operations on nail bed	T	379101	686	378415	9.35E-05	-9.27768	119.953	7.32E-107	1.19E+98	0.93835	0.998648
Operations and Procedures	Other operations on nail	T	379101	396	378705	6.61E-05	-9.62469	198.596	5.91E-174	7.39E+164	0.961347	0.998648
Operations and Procedures	Opening of chest	T	379101	257	378844	0.000104	-9.17464	190.309	1.05E-166	1.02E+158	0.96155	0.998648
Operations and Procedures	Primary repair of inguinal hernia	T	379101	13724	365377	1.27	0.2403	0.398174	0.583	2.78	0.546173	0.998648
Operations and Procedures	Repair of recurrent inguinal hernia	T	379101	1175	377926	6.23E-05	-9.68361	111.043	1.87E-99	2.07E+90	0.930508	0.998648
Operations and Procedures	Primary repair of femoral hernia	T	379101	527	378574	5.08E-05	-9.88847	196.368	3.58E-172	7.20E+162	0.959838	0.998648
Operations and Procedures	Primary repair of umbilical hernia	T	379101	3099	376002	5.63E-05	-9.78399	71.9634	3.12E-66	1.02E+57	0.891855	0.998648
Operations and Procedures	Primary repair of incisional hernia	T	379101	1622	377479	4.20E-05	-10.077	120.31	1.64E-107	1.08E+98	0.933248	0.998648
Operations and Procedures	Repair of recurrent incisional hernia	T	379101	225	378876	0.000112	-9.09778	197.929	3.70E-173	3.38E+164	0.963338	0.998648
Operations and Procedures	Repair of other hernia of abdominal wall	T	379101	1344	377757	4.90E-05	-9.9233	121.07	4.30E-108	5.59E+98	0.934676	0.998648
Operations and Procedures	Opening of abdomen	T	379101	940	378161	7.39E-05	-9.51264	120.734	1.25E-107	4.36E+98	0.9372	0.998648
Operations and Procedures	Other operations on anterior abdominal wall	T	379101	600	378501	0.000112	-9.0928	121.044	1.04E-107	1.22E+99	0.940119	0.998648
Operations and Procedures	Open drainage of peritoneum	T	379101	297	378804	8.54E-05	-9.36855	199.068	3.03E-174	2.41E+165	0.962464	0.998648
Operations and Procedures	Operations on omentum	T	379101	1948	377153	4.22E-05	-10.0738	114.556	1.30E-102	1.37E+93	0.929926	0.998648
Operations and Procedures	Other open operations on peritoneum	T	379101	2184	376917	1.26	0.232692	1.00425	0.176	9.03	0.816766	0.998648
Operations and Procedures	Therapeutic endoscopic operations on peritoneum	T	379101	1599	377502	4.70E-05	-9.96576	117.985	1.74E-105	1.27E+96	0.932685	0.998648
Operations and Procedures	Diagnostic endoscopic examination of peritoneum	T	379101	4723	374378	0.658	-0.418743	1.00776	0.0913	4.74	0.677762	0.998648
Operations and Procedures	Other drainage of peritoneal cavity	T	379101	2011	377090	1.39	0.328408	1.00432	0.194	9.94	0.743671	0.998648
Operations and Procedures	Excision of other fascia	T	379101	2947	376154	6.49E-05	-9.6428	70.098	1.39E-64	3.03E+55	0.890587	0.998648
Operations and Procedures	Division of fascia	T	379101	394	378707	6.66E-05	-9.61678	192.229	1.57E-168	2.83E+159	0.9601	0.998648
Operations and Procedures	Excision of ganglion	T	379101	2429	376672	2.34	0.85135	0.712845	0.579	9.47	0.232361	0.998648
Operations and Procedures	Operations on bursa	T	379101	1825	377276	1.52	0.4157	1.00405	0.212	10.8	0.678858	0.998648
Operations and Procedures	Transposition of tendon	T	379101	795	378306	8.45E-05	-9.37919	120.989	8.68E-108	8.21E+98	0.938209	0.998648
Operations and Procedures	Excision of tendon	T	379101	253	378848	0.000102	-9.1932	198.422	1.28E-173	8.08E+164	0.963046	0.998648
Operations and Procedures	Primary repair of tendon	T	379101	2574	376527	0.979	-0.021592	1.00453	0.137	7.01	0.982851	0.998648
Operations and Procedures	Adjustment to length of tendon	T	379101	2142	376959	8.94E-05	-9.32242	72.4137	2.05E-66	3.90E+57	0.897565	0.998648
Operations and Procedures	Other operations on sheath of tendon	T	379101	2908	376193	0.929	-0.074055	1.00415	0.13	6.65	0.94121	0.998648
Operations and Procedures	Other operations on tendon	T	379101	780	378321	3.43	1.23139	1.00437	0.478	24.5	0.220185	0.998648
Operations and Procedures	Excision of muscle	T	379101	476	378625	5.02E-05	-9.89952	199.436	8.66E-175	2.91E+165	0.960411	0.998648
Operations and Procedures	Repair of muscle	T	379101	2257	376844	8.08E-05	-9.42338	72.8034	8.63E-67	7.57E+57	0.897013	0.998648
Operations and Procedures	Biopsy of muscle	T	379101	421	378680	5.74E-05	-9.76497	199.531	8.22E-175	4.01E+165	0.960967	0.998648
Operations and Procedures	Other operations on muscle	T	379101	238	378863	0.000105	-9.16094	199.388	1.99E-174	5.55E+165	0.963354	0.998648
Operations and Procedures	Block dissection of lymph nodes	T	379101	5157	373944	1.1	0.093985	0.714224	0.271	4.45	0.895308	0.998648
Operations and Procedures	Sampling of lymph nodes	T	379101	2952	376149	1.97	0.678013	0.717131	0.483	8.03	0.344428	0.998648
Operations and Procedures	Excision or biopsy of lymph node	T	379101	6160	372941	0.45	-0.797556	1.0043	0.0629	3.22	0.427112	0.998648
Operations and Procedures	Operations on sentinel lymph node	T	379101	1143	377958	2.52	0.925338	1.00749	0.35	18.2	0.35838	0.998648
Operations and Procedures	Other operations on soft tissue	T	379101	1277	377824	2.13	0.754358	1.00407	0.297	15.2	0.45247	0.998648
Operations and Procedures	Diagnostic imaging of central nervous system	T	379101	13682	365419	0.763	-0.270445	0.507886	0.282	2.06	0.594386	0.998648
Operations and Procedures	Diagnostic imaging of abdomen	T	379101	2458	376643	7.64E-05	-9.47919	72.8811	7.01E-67	8.33E+57	0.896516	0.998648
Operations and Procedures	Diagnostic imaging of pelvis	T	379101	1273	377828	2.04	0.714192	1.00418	0.285	14.6	0.476948	0.998648
Operations and Procedures	Diagnostic imaging of heart	T	379101	1244	377857	5.19E-05	-9.86662	119.733	6.25E-107	4.30E+97	0.934324	0.998648
Operations and Procedures	Diagnostic imaging of vascular system	T	379101	1073	378028	6.32E-05	-9.66972	120.403	2.05E-107	1.95E+98	0.93599	0.998648
Operations and Procedures	Diagnostic imaging of genitourinary system	T	379101	293	378808	9.48E-05	-9.26356	193.57	1.61E-169	5.58E+160	0.961831	0.998648
Operations and Procedures	Diagnostic imaging of musculoskeletal system	T	379101	820	378281	8.09E-05	-9.42266	120.905	9.80E-108	6.67E+98	0.93788	0.998648
Operations and Procedures	Diagnostic imaging of digestive tract	T	379101	243	378858	0.00012	-9.02635	195.271	7.28E-171	1.99E+162	0.963131	0.998648
Operations and Procedures	Diagnostic electrocardiography	T	379101	998	378103	2.8	1.02868	1.00602	0.389	20.1	0.306534	0.998648
Operations and Procedures	Neuropsychology tests	T	379101	256	378845	9.47E-05	-9.26494	198.9	4.67E-174	1.92E+165	0.962847	0.998648
Operations and Procedures	Breath tests	T	379101	412	378689	6.54E-05	-9.63567	198.422	8.22E-174	5.19E+164	0.961269	0.998648
Operations and Procedures	Diagnostic endocrinology	T	379101	1212	377889	5.73E-05	-9.7675	121.094	4.79E-108	6.84E+98	0.935712	0.998648
Operations and Procedures	Autonomic cardiovascular testing	T	379101	235	378866	0.000114	-9.0797	196.773	3.63E-172	3.58E+163	0.963196	0.998648
Operations and Procedures	Other diagnostic imaging of genitourinary system	T	379101	209	378892	0.000113	-9.08376	199.395	2.12E-174	6.07E+165	0.963664	0.998648
Operations and Procedures	Rehabilitation for musculoskeletal disorders	T	379101	364	378737	6.70E-05	-9.61131	196.148	7.27E-172	6.17E+162	0.960919	0.998648
Operations and Procedures	Rehabilitation for neurological disorders	T	379101	314	378787	7.61E-05	-9.48326	199.192	2.12E-174	2.74E+165	0.962028	0.998648
Operations and Procedures	Rehabilitation for other disorders	T	379101	724	378377	9.43E-05	-9.26877	118.209	2.25E-105	3.95E+96	0.937502	0.998648
Operations and Procedures	Reduction of fracture of other bone of face	T	379101	686	378415	0.000102	-9.18744	118.771	8.13E-106	1.29E+97	0.938342	0.998648
Operations and Procedures	Fixation of bone of face	T	379101	219	378882	0.000117	-9.05392	194.236	5.38E-170	2.54E+161	0.962822	0.998648
Operations and Procedures	Fixation of mandible	T	379101	258	378843	9.99E-05	-9.21179	194.397	3.35E-170	2.97E+161	0.962205	0.998648
Operations and Procedures	Primary decompression operations on cervical spine	T	379101	557	378544	4.22E-05	-10.072	199.077	1.47E-174	1.21E+165	0.95965	0.998648
Operations and Procedures	Primary decompression operations on lumbar spine	T	379101	2461	376640	1.06	0.054543	1.0042	0.148	7.56	0.956684	0.998648
Operations and Procedures	Primary excision of cervical intervertebral disc	T	379101	805	378296	8.13E-05	-9.41676	120.82	1.16E-107	5.68E+98	0.937876	0.998648
Operations and Procedures	Primary excision of lumbar intervertebral disc	T	379101	1796	377305	3.73E-05	-10.1958	120.879	4.76E-108	2.93E+98	0.932781	0.998648
Operations and Procedures	Stabilisation of spine	T	379101	284	378817	8.21E-05	-9.40747	198.659	6.49E-174	1.04E+165	0.96223	0.998648
Operations and Procedures	Instrumental correction of deformity of spine	T	379101	342	378759	7.02E-05	-9.56406	198.971	3.01E-174	1.64E+165	0.961662	0.998648
Operations and Procedures	Denervation of spinal facet joint of vertebra	T	379101	675	378426	9.59E-05	-9.25175	120.685	1.79E-107	5.14E+98	0.938894	0.998648
Operations and Procedures	Manipulation of spine	T	379101	218	378883	0.000116	-9.06228	198.258	2.01E-173	6.68E+164	0.963542	0.998648
Operations and Procedures	Other operations on intervertebral disc	T	379101	298	378803	8.51E-05	-9.37174	198.238	1.54E-173	4.72E+164	0.962294	0.998648
Operations and Procedures	Other operations on spine	T	379101	4622	374479	1.18	0.163894	0.712639	0.291	4.76	0.818106	0.998648
Operations and Procedures	Levels of spine	T	379101	6828	372273	1.17	0.152764	0.583858	0.371	3.66	0.793595	0.998648
Operations and Procedures	Complex reconstruction of forefoot	T	379101	800	378301	9.65E-05	-9.24595	116.793	3.70E-104	2.52E+95	0.936901	0.998648
Operations and Procedures	Complex reconstruction of hindfoot	T	379101	342	378759	7.14E-05	-9.547	198.342	1.05E-173	4.85E+164	0.961609	0.998648
Operations and Procedures	Total excision of bone	T	379101	1188	377913	2.31	0.835344	1.00568	0.321	16.6	0.406187	0.998648
Operations and Procedures	Other excision of bone	T	379101	3701	375400	1.48	0.389491	0.712642	0.365	5.97	0.584692	0.998648
Operations and Procedures	Extirpation of lesion of bone	T	379101	466	378635	5.08E-05	-9.88837	199.364	1.01E-174	2.56E+165	0.960441	0.998648
Operations and Procedures	Other periarticular division of bone	T	379101	263	378838	9.99E-05	-9.21141	196.339	7.45E-172	1.34E+163	0.96258	0.998648
Operations and Procedures	Diaphyseal division of bone	T	379101	396	378705	7.43E-05	-9.5077	187.449	2.05E-164	2.70E+155	0.959547	0.998648
Operations and Procedures	Division of bone of foot	T	379101	4767	374334	0.605	-0.503235	1.00621	0.0841	4.34	0.616983	0.998648
Operations and Procedures	Other division of bone	T	379101	1162	377939	5.94E-05	-9.73176	119.361	1.48E-106	2.38E+97	0.935019	0.998648
Operations and Procedures	Bone and intramedullary fixation	T	379101	2523	376578	7.12E-05	-9.55028	73.4948	1.96E-67	2.58E+58	0.89661	0.998648
Operations and Procedures	Bone and extramedullary fixation	T	379101	5789	373312	1.89	0.636048	0.507655	0.698	5.11	0.210237	0.998648
Operations and Procedures	Primary open reduction of intra articular fracture of bone	T	379101	292	378809	8.71E-05	-9.34831	199.553	1.19E-174	6.35E+165	0.962636	0.998648
Operations and Procedures	Closed reduction of fracture of bone and internal fixation	T	379101	2838	376263	0.954	-0.047384	1.0039	0.133	6.82	0.962354	0.998648
Operations and Procedures	Closed reduction of fracture of bone and external fixation	T	379101	391	378710	6.68E-05	-9.61414	198.316	1.03E-173	4.31E+164	0.961335	0.998648
Operations and Procedures	Other closed reduction of fracture of bone	T	379101	1496	377605	4.97E-05	-9.90907	119.82	5.05E-107	4.89E+97	0.93409	0.998648
Operations and Procedures	Other internal fixation of bone	T	379101	5999	373102	0.443	-0.813569	1.00362	0.062	3.17	0.417575	0.998648
Operations and Procedures	Skeletal traction of bone	T	379101	210	378891	0.000122	-9.00921	198.486	1.36E-173	1.10E+165	0.963797	0.998648
Operations and Procedures	Other external fixation of bone	T	379101	667	378434	9.96E-05	-9.21468	120.908	1.20E-107	8.26E+98	0.93925	0.998648
Operations and Procedures	Other autograft of bone	T	379101	1078	378023	2.55	0.935412	1.00416	0.356	18.2	0.351576	0.998648
Operations and Procedures	Other graft of bone	T	379101	696	378405	9.99E-05	-9.21172	120.55	2.43E-107	4.11E+98	0.93909	0.998648
Operations and Procedures	Other open operations on bone	T	379101	605	378496	0.00011	-9.11468	120.53	2.78E-107	4.35E+98	0.93972	0.998648
Operations and Procedures	Diagnostic puncture of bone	T	379101	3796	375305	0.698	-0.359662	1.00448	0.0974	5	0.720299	0.998648
Operations and Procedures	Total prosthetic replacement of hip joint not using cement	T	379101	4094	375007	1.95	0.668645	0.585887	0.619	6.15	0.253765	0.998648
Operations and Procedures	Other total prosthetic replacement of hip joint	T	379101	1064	378037	6.63E-05	-9.62203	116.884	2.13E-104	2.06E+95	0.934392	0.998648
Operations and Procedures	Total prosthetic replacement of knee joint using cement	T	379101	7800	371301	0.346	-1.06109	1.00572	0.0482	2.48	0.291399	0.998648
Operations and Procedures	Total prosthetic knee joint not using cement	T	379101	695	378406	0.000103	-9.17766	117.394	1.22E-104	8.75E+95	0.937686	0.998648
Operations and Procedures	Other total prosthetic replacement of knee joint	T	379101	844	378257	3.14	1.1453	1.00644	0.437	22.6	0.255132	0.998648
Operations and Procedures	Total prosthetic replacement of other joint not using cement	T	379101	305	378796	9.27E-05	-9.28658	194.09	5.68E-170	1.51E+161	0.961838	0.998648
Operations and Procedures	Other total prosthetic replacement of other joint	T	379101	332	378769	8.06E-05	-9.4254	195.796	1.74E-171	3.73E+162	0.961605	0.998648
Operations and Procedures	Prosthetic replacement of head of femur using cement	T	379101	222	378879	5.16E-05	-9.87152	307.544	8.44E-267	3.16E+257	0.974394	0.998648
Operations and Procedures	Other interposition reconstruction of joint	T	379101	307	378794	8.82E-05	-9.33627	191.622	6.81E-168	1.14E+159	0.961141	0.998648
Operations and Procedures	Other reconstruction of joint	T	379101	2374	376727	1.06	0.059833	1.00456	0.148	7.6	0.952505	0.998648
Operations and Procedures	Fusion of joint of toe	T	379101	3193	375908	0.905	-0.100105	1.00559	0.126	6.49	0.920703	0.998648
Operations and Procedures	Other primary fusion of other joint	T	379101	1462	377639	1.88	0.631162	1.00447	0.262	13.5	0.529773	0.998648
Operations and Procedures	Primary open reduction of traumatic dislocation of joint	T	379101	347	378754	7.42E-05	-9.50872	199.443	1.26E-174	4.36E+165	0.961974	0.998648
Operations and Procedures	Primary closed reduction of traumatic dislocation of joint	T	379101	775	378326	3.59	1.27838	1.00444	0.501	25.7	0.203112	0.998648
Operations and Procedures	Secondary reduction of traumatic dislocation of joint	T	379101	235	378866	0.000116	-9.05972	198.896	5.78E-174	2.34E+165	0.963669	0.998648
Operations and Procedures	Open operations on synovial membrane of joint	T	379101	853	378248	7.60E-05	-9.48448	121.007	7.55E-108	7.66E+98	0.937526	0.998648
Operations and Procedures	Other open operations on intra articular structure	T	379101	1473	377628	4.58E-05	-9.9916	120.567	1.08E-107	1.95E+98	0.933954	0.998648
Operations and Procedures	Other reconstruction of ligament	T	379101	749	378352	9.09E-05	-9.30543	117.827	4.60E-105	1.80E+96	0.937052	0.998648
Operations and Procedures	Other open repair of ligament	T	379101	420	378681	5.94E-05	-9.73191	198.798	3.57E-174	9.86E+164	0.960956	0.998648
Operations and Procedures	Other operations on ligament	T	379101	254	378847	9.40E-05	-9.2727	199.083	3.24E-174	2.73E+165	0.96285	0.998648
Operations and Procedures	Stabilising operations on joint	T	379101	716	378385	8.85E-05	-9.33291	120.896	1.09E-107	7.17E+98	0.938466	0.998648

Operations and Procedures	Release of contracture of joint	T	379101	1613	377488	1.68	0.515959	1.00403	0.234	12	0.607332	0.998648
Operations and Procedures	Soft tissue operations on joint of toe	T	379101	2721	376380	7.96E-05	-9.43885	70.0244	1.97E-64	3.21E+55	0.892775	0.998648
Operations and Procedures	Other open operations on joint	T	379101	1273	377828	5.26E-05	-9.8528	120.835	7.31E-108	3.78E+98	0.935013	0.998648
Operations and Procedures	Therapeutic endoscopic operations on semilunar cartilage	T	379101	12492	366609	1.47	0.387607	0.388189	0.688	3.15	0.318037	0.998648
Operations and Procedures	Therapeutic endoscopic operations on other articular cartilage	T	379101	3095	376006	0.798	-0.225154	1.00388	0.112	5.71	0.822536	0.998648
Operations and Procedures	Therapeutic endoscopic operations on other joint structure	T	379101	4753	374348	1.63	0.48922	0.583789	0.519	5.12	0.402026	0.998648
Operations and Procedures	Therapeutic endoscopic operations on cavity of knee joint	T	379101	4112	374989	0.615	-0.48689	1.00366	0.0859	4.39	0.627595	0.998648
Operations and Procedures	Therapeutic endoscopic operations on cavity of other joint	T	379101	583	378518	0.000113	-9.09175	120.844	1.54E-107	8.24E+98	0.940027	0.998648
Operations and Procedures	Diagnostic endoscopic examination of knee joint	T	379101	3016	376085	0.873	-0.13546	1.00368	0.122	6.24	0.89264	0.998648
Operations and Procedures	Diagnostic endoscopic examination of other joint	T	379101	560	378541	4.37E-05	-10.0374	199.027	1.68E-174	1.14E+165	0.959778	0.998648
Operations and Procedures	Operations on other articular cartilage	T	379101	1114	377987	5.80E-05	-9.75565	120.87	7.53E-108	4.46E+98	0.935671	0.998648
Operations and Procedures	Puncture of joint	T	379101	10120	368981	0.558	-0.584201	0.712233	0.138	2.25	0.41208	0.998648
Operations and Procedures	Other manipulation of joint	T	379101	2050	377051	9.22E-05	-9.29206	73.106	5.44E-67	1.56E+58	0.898858	0.998648
Operations and Procedures	Other operations on joint	T	379101	520	378581	4.78E-05	-9.94908	199.575	6.27E-175	3.64E+165	0.960241	0.998648
Operations and Procedures	Hybrid prosthetic replacement of hip joint	T	379101	286	378815	0.00011	-9.11909	191.643	8.12E-168	1.48E+159	0.962048	0.998648
Operations and Procedures	Replacement of hip joint	T	379101	1139	377962	5.87E-05	-9.74344	116.171	7.62E-104	4.52E+94	0.933159	0.998648
Operations and Procedures	Continuous Infusion of therapeutic substance	T	379101	8393	370708	0.985	-0.015346	0.58433	0.313	3.1	0.979048	0.998648
Operations and Procedures	Injection of therapeutic substance	T	379101	1288	377813	5.71E-05	-9.77077	119.167	2.09E-106	1.56E+97	0.934653	0.998648
Operations and Procedures	Injection of radiocontrast material	T	379101	1644	377457	4.15E-05	-10.0892	120.149	2.22E-107	7.78E+97	0.933078	0.998648
Operations and Procedures	Other blood transfusion	T	379101	4631	374470	1.17	0.154426	0.712759	0.289	4.72	0.828474	0.998648
Operations and Procedures	Other intravenous transfusion	T	379101	222	378879	0.000113	-9.08951	198.902	5.54E-174	2.30E+165	0.963551	0.998648
Operations and Procedures	Other intravenous injection	T	379101	5047	374054	3.61E-05	-10.2292	72.9444	2.92E-67	4.46E+57	0.888477	0.998648
Operations and Procedures	Subcutaneous injection	T	379101	2578	376523	1.05	0.046716	1.00397	0.146	7.5	0.962887	0.998648
Operations and Procedures	Other route of administration of therapeutic substance	T	379101	241	378860	0.000123	-9.0018	194.461	3.65E-170	4.16E+161	0.963078	0.998648
Operations and Procedures	Compensation for renal failure	T	379101	703	378398	3.71	1.31098	1.00538	0.517	26.6	0.192246	0.998648
Operations and Procedures	Immobilisation using plaster cast	T	379101	694	378407	0.000102	-9.19075	120.583	2.32E-107	4.48E+98	0.939245	0.998648
Operations and Procedures	Other operations on unspecified organ	T	379101	2194	376907	4.36E-05	-10.0404	108.296	2.86E-97	6.65E+87	0.926132	0.998648
Operations and Procedures	Radiotherapy delivery	T	379101	2124	376977	2.27	0.818235	0.715415	0.558	9.21	0.25274	0.998648
Operations and Procedures	Preparation for external beam radiotherapy	T	379101	655	378446	9.98E-05	-9.21252	120	7.13E-107	1.40E+98	0.938806	0.998648
Operations and Procedures	Procurement of drugs for chemotherapy for neoplasm in Bands 1 5	T	379101	5730	373371	3.06E-05	-10.3939	72.9295	2.55E-67	3.67E+57	0.886669	0.998648
Operations and Procedures	Procurement of drugs for chemotherapy for neoplasm in Bands 6 10	T	379101	4078	375023	1.25	0.224469	0.713082	0.309	5.06	0.752923	0.998648
Operations and Procedures	Delivery of chemotherapy for neoplasm	T	379101	7579	371522	0.693	-0.367178	0.713045	0.171	2.8	0.606592	0.998648
Operations and Procedures	Delivery of oral chemotherapy for neoplasm	T	379101	560	378541	4.48E-05	-10.014	197.459	3.72E-173	5.39E+163	0.959553	0.998648
Operations and Procedures	High cost other cardiovascular drugs	T	379101	535	378566	4.77E-05	-9.94992	196.01	6.79E-172	3.36E+162	0.959515	0.998648
Operations and Procedures	High cost haematology and nutrition drugs	T	379101	606	378495	0.000112	-9.09985	120.331	4.17E-107	2.99E+98	0.939719	0.998648
Operations and Procedures	High cost musculoskeletal drugs	T	379101	570	378531	4.35E-05	-10.0435	199.133	1.36E-174	1.39E+165	0.959775	0.998648
Operations and Procedures	High cost ophthalmology drugs	T	379101	346	378755	8.46E-05	-9.37714	190.448	6.53E-167	1.10E+158	0.96073	0.998648
Operations and Procedures	High cost immunology drugs	T	379101	340	378761	6.98E-05	-9.56956	197.777	3.11E-173	1.57E+164	0.961409	0.998648
Operations and Procedures	Placement of prosthesis in organ NOC	T	379101	3165	375936	0.82	-0.198485	1.00596	0.114	5.89	0.843587	0.998648
Operations and Procedures	Attention to prosthesis in organ NOC	T	379101	2929	376172	0.904	-0.100979	1.00396	0.126	6.47	0.919883	0.998648
Operations and Procedures	Obliteration of cavity of organ NOC	T	379101	637	378464	0.000104	-9.1738	119.619	1.56E-106	6.88E+97	0.938869	0.998648
Operations and Procedures	Laser therapy to organ NOC	T	379101	2987	376114	1.78	0.57789	0.713251	0.44	7.21	0.417814	0.998648
Operations and Procedures	Other destruction of organ NOC	T	379101	2176	376925	8.42E-05	-9.38207	73.0689	5.34E-67	1.33E+58	0.897832	0.998648
Operations and Procedures	Chemical destruction of lesion of organ NOC	T	379101	228	378873	0.000119	-9.03969	192.069	3.82E-168	3.69E+159	0.962462	0.998648
Operations and Procedures	Other destruction of lesion of organ NOC	T	379101	2552	376549	1.02	0.017224	1.00396	0.142	7.28	0.986312	0.998648
Operations and Procedures	Placement of stent in organ NOC	T	379101	1320	377781	5.10E-05	-9.88385	117.628	3.81E-105	6.83E+95	0.933036	0.998648
Operations and Procedures	Attention to stent in organ NOC	T	379101	390	378711	6.23E-05	-9.68349	197.955	1.96E-173	1.98E+164	0.960985	0.998648
Operations and Procedures	Connection of organ NOC	T	379101	354	378747	7.06E-05	-9.55861	197.464	5.81E-173	8.58E+163	0.961392	0.998648
Operations and Procedures	Release of organ NOC	T	379101	896	378205	3.07	1.12086	1.00441	0.428	22	0.264451	0.998648
Operations and Procedures	Biopsy of organ NOC	T	379101	7160	371941	0.739	-0.303132	0.713051	0.183	2.99	0.67075	0.998648
Operations and Procedures	Cytology of organ NOC	T	379101	2554	376547	2.21	0.791353	0.713042	0.545	8.93	0.267074	0.998648
Operations and Procedures	Drainage of organ NOC	T	379101	2627	376474	6.77E-05	-9.60047	73.4522	2.03E-67	2.26E+58	0.89601	0.998648
Operations and Procedures	Other repair of organ NOC	T	379101	893	378208	2.95	1.08024	1.00433	0.411	21.1	0.282115	0.998648
Operations and Procedures	Graft to organ NOC	T	379101	624	378477	0.000107	-9.1403	121.005	1.07E-107	1.08E+99	0.939788	0.998648
Operations and Procedures	Re exploration of organ NOC	T	379101	217	378884	0.000108	-9.13138	199.089	3.68E-174	3.18E+165	0.963417	0.998648
Operations and Procedures	Puncture of organ NOC	T	379101	315	378786	8.26E-05	-9.40094	198.648	6.68E-174	1.02E+165	0.962254	0.998648
Operations and Procedures	Injection of therapeutic substance into organ NOC	T	379101	608	378493	0.000118	-9.04634	120.707	2.11E-107	6.59E+98	0.940259	0.998648
Operations and Procedures	Dilation of organ NOC	T	379101	512	378589	4.77E-05	-9.95115	197.954	1.50E-173	1.51E+164	0.959907	0.998648
Operations and Procedures	Examination of organ NOC	T	379101	581	378520	4.25E-05	-10.0668	198.743	2.85E-174	6.33E+164	0.959603	0.998648
Operations and Procedures	Open approach to contents of cranium	T	379101	800	378301	8.03E-05	-9.42931	120.995	8.16E-108	7.91E+98	0.937882	0.998648
Operations and Procedures	Burrhole approach to contents of cranium	T	379101	411	378690	5.98E-05	-9.72486	197.552	4.14E-173	8.63E+163	0.960739	0.998648
Operations and Procedures	Approach to spine through back	T	379101	309	378792	7.37E-05	-9.51558	198.285	1.21E-173	4.48E+164	0.961725	0.998648
Operations and Procedures	Approach through thoracic cavity	T	379101	602	378499	4.07E-05	-10.1101	195.137	3.20E-171	5.17E+161	0.95868	0.998648
Operations and Procedures	Approach through abdominal cavity	T	379101	6488	372613	1.75	0.557048	0.508635	0.644	4.73	0.273437	0.998648
Operations and Procedures	Opening into gastrointestinal tract	T	379101	207	378894	0.000126	-8.97718	196.854	3.43E-172	4.64E+163	0.963626	0.998648
Operations and Procedures	Approach to organ through other opening	T	379101	1441	377660	4.69E-05	-9.96745	120.735	7.93E-108	2.77E+98	0.934204	0.998648
Operations and Procedures	Approach to organ under image control	T	379101	45067	334034	1.19	0.173253	0.244236	0.737	1.92	0.478098	0.998648
Operations and Procedures	Harvest of skin for graft	T	379101	2280	376821	1.13	0.125208	1.00481	0.158	8.12	0.900833	0.998648
Operations and Procedures	Harvest of flap of skin and fascia	T	379101	546	378555	4.57E-05	-9.99248	199.548	6.33E-175	3.30E+165	0.960062	0.998648
Operations and Procedures	Harvest of tendon	T	379101	532	378569	4.66E-05	-9.97304	198.152	9.96E-174	2.18E+164	0.959859	0.998648
Operations and Procedures	Harvest of bone	T	379101	1453	377648	1.84	0.611691	1.00393	0.258	13.2	0.542327	0.998648
Operations and Procedures	Harvest of other tissue	T	379101	784	378317	8.47E-05	-9.37604	120.914	1.01E-107	7.11E+98	0.938191	0.998648
Operations and Procedures	Early operations NOC	T	379101	2989	376112	6.09E-05	-9.70631	72.6669	8.50E-67	4.36E+57	0.89374	0.998648
Operations and Procedures	Late operations NOC	T	379101	5407	373694	3.29E-05	-10.3231	73.4057	1.08E-67	1.00E+58	0.888161	0.998648
Operations and Procedures	Facilitating operations NOC	T	379101	2533	376568	7.22E-05	-9.53539	69.9011	2.28E-64	2.29E+55	0.891495	0.998648
Operations and Procedures	Minimal access to thoracic cavity	T	379101	726	378375	3.59	1.2781	1.00532	0.5	25.8	0.203609	0.998648
Operations and Procedures	Minimal access to abdominal cavity	T	379101	14863	364238	0.524	-0.646211	0.58365	0.167	1.65	0.268213	0.998648
Operations and Procedures	Arteriotomy approach to organ under image control	T	379101	426	378675	5.80E-05	-9.75448	191.401	6.92E-168	4.87E+158	0.959354	0.998648
Operations and Procedures	Approach to organ through artery	T	379101	284	378817	7.22E-05	-9.53586	186.968	5.11E-164	1.02E+155	0.959324	0.998648
Operations and Procedures	General anaesthetic	T	379101	13536	365565	0.885	-0.122038	0.438109	0.375	2.09	0.780585	0.998648
Operations and Procedures	Spinal anaesthetic	T	379101	6624	372477	2.81E-05	-10.4801	70.6346	2.10E-65	3.75E+55	0.88205	0.998648
Operations and Procedures	Local anaesthetic	T	379101	23563	355538	1.28	0.243493	0.306893	0.699	2.33	0.427538	0.998648
Operations and Procedures	Other anaesthetic	T	379101	5945	373156	1.62	0.480927	0.5133	0.591	4.42	0.348794	0.998648
Operations and Procedures	Other non operations	T	379101	2978	376123	7.94E-05	-9.4405	68.165	7.53E-63	8.38E+53	0.889849	0.998648
Operations and Procedures	External beam radiotherapy	T	379101	1376	377725	4.25E-05	-10.0669	117.542	3.75E-105	4.81E+95	0.931748	0.998648
Operations and Procedures	Radiopharmaceutical imaging	T	379101	249	378852	0.000107	-9.1465	198.572	1.00E-173	1.14E+165	0.963261	0.998648
Operations and Procedures	Gestational age	T	379101	395	378706	0.000107	-9.14637	258.733	6.16E-225	1.84E+216	0.9718	0.998648
NA	Myocardial infarction	T	379101	13488	365613	0.563	-0.574524	0.589543	0.177	1.79	0.329797	0.998648
NA	STEMI	T	379101	3811	375290	5.05E-05	-9.89353	69.7651	2.08E-64	1.23E+55	0.887228	0.998648
NA	NSTEMI	T	379101	4419	374682	0.586	-0.533583	1.00625	0.0816	4.22	0.595925	0.998648
NA	Stroke	T	379101	8703	370398	1.57	0.447902	0.458561	0.637	3.84	0.32869	0.998648
NA	Ischaemic stroke	T	379101	3655	375446	1.47	0.386713	0.715175	0.362	5.98	0.588697	0.998648
NA	Intracerebral haemorrhage	T	379101	792	378309	8.60E-05	-9.36083	118.797	6.50E-106	1.14E+97	0.937194	0.998648
Mental health	Ever depressed for a whole week	T	124064	66758	57306	0.784	-0.24298	0.255204	0.476	1.29	0.341047	0.998648
Mental health	Ever manic hyper for 2 days	T	123498	7031	116467	0.267	-1.3204	1.00923	0.0369	1.93	0.190765	0.998648
Mental health	Ever highly irritable argumentative for 2 days	T	123759	22263	101496	0.88	-0.127611	0.347937	0.445	1.74	0.713794	0.998648
Cardiovascular	Leg pain on walking	T	125416	27294	98122	0.841	-0.173318	0.321745	0.448	1.58	0.590107	0.998648
Eye	Ever had eye surgery	T	84673	6217	78456	0.372	-0.989964	1.01324	0.051	2.71	0.328555	0.998648
Eye	Current eye infection	T	85179	898	84281	4.47E-05	-10.0155	148.672	1.25E-131	1.59E+122	0.94629	0.998648
Eye	Diabetes related eye disease	T	124376	2369	122007	2.59E-05	-10.563	118.957	1.43E-106	4.69E+96	0.929243	0.998648
Eye	Glaucoma	T	124376	5306	119070	1.44	0.365644	0.60075	0.444	4.68	0.542759	0.998648
Eye	Cataract	T	124376	11723	112653	0.654	-0.425036	0.603522	0.2	2.13	0.481271	0.998648
Eye	Macular degeneration	T	124376	2870	121506	6.42E-05	-9.65336	71.6922	6.05E-66	6.81E+56	0.892889	0.998648
Eye	Other serious eye condition	T	124376	6929	117447	1.01	0.013166	0.595313	0.315	3.25	0.982356	0.998648
ENT	Mouth ulcers	T	377646	38476	339170	1.01	0.011366	0.27329	0.592	1.73	0.966825	0.998648
ENT	Painful gums	T	377646	10716	366930	0.495	-0.703941	0.711933	0.123	2	0.322774	0.998648
ENT	Bleeding gums	T	377646	49304	328342	0.962	-0.038265	0.253722	0.585	1.58	0.880121	0.998648
ENT	Loose teeth	T	377646	15091	362555	0.521	-0.651787	0.583968	0.166	1.64	0.264364	0.998648
ENT	Toothache	T	377646	15330	362316	1.38	0.319437	0.364653	0.673	2.81	0.38103	0.998648
ENT	Dentures	T	377646	63135	314511	0.746	-0.292616	0.261766	0.447	1.25	0.263629	0.998648
Cardiovascular	Blood pressure medication	T	376222	77608	298614	0.916	-0.087986	0.223369	0.591	1.42	0.693652	0.998648
Metabolic	Insulin	T	376222	3934	372288	1.36	0.30817	0.713083	0.336	5.51	0.665621	0.998648
Medication	Aspirin	T	374768	52613	322155	0.81	-0.211156	0.265545	0.481	1.36	0.426508	0.998648
Medication	Ibuprofen e g Nurofen	T	374768	55904	318864	0.762	-0.271734	0.260626	0.457	1.27	0.297124	0.998648
Medication	Paracetamol	T	374768	81842	292926	1.2	0.180352	0.195953	0.816	1.76	0.35737	0.998648

Medication	Ranitidine e g Zantac	T	374768	7205	367563	0.377	-0.974724	1.00369	0.0528	2.7	0.331481	0.998648
Medication	Omeprazole e g Zanprol	T	374768	22414	352354	1.38	0.319202	0.315961	0.741	2.56	0.312372	0.998648
Medication	Laxatives e g Dulcolax Senokot	T	374768	10818	363950	1.05	0.050506	0.509566	0.387	2.86	0.921047	0.998648
Medication	Vitamin A	T	377036	7193	369843	0.363	-1.01391	1.00363	0.0507	2.59	0.312376	0.998648
Medication	Vitamin C	T	377036	32744	344292	0.872	-0.136743	0.313915	0.471	1.61	0.663122	0.998648
Medication	Vitamin D	T	377036	14603	362433	0.539	-0.617751	0.584856	0.171	1.7	0.290856	0.998648
Medication	Vitamin E	T	377036	11056	365980	0.716	-0.334382	0.584023	0.228	2.25	0.56695	0.998648
Medication	Folic acid or Folate Vit B9	T	377036	8174	368862	0.327	-1.11788	1.0038	0.0457	2.34	0.26543	0.998648
Medication	Multivitamins minerals	T	377036	81736	295300	1.09	0.082511	0.199003	0.735	1.6	0.67842	0.998648
Neurosciences	Headache	T	378231	81591	296640	1.1	0.092408	0.201149	0.739	1.63	0.645948	0.998648
Neurosciences	Facial pain	T	378231	12842	365389	0.422	-0.863658	0.712016	0.104	1.7	0.225139	0.998648
Neurosciences	Neck or shoulder pain	T	378231	92289	285942	0.874	-0.135117	0.20296	0.587	1.3	0.505582	0.998648
Neurosciences	Stomach or abdominal pain	T	378231	37960	340271	0.69	-0.371428	0.328525	0.362	1.31	0.258227	0.998648
Neurosciences	Knee pain	T	378231	86052	292179	0.762	-0.271308	0.217145	0.498	1.17	0.211507	0.998648
Neurosciences	Pain all over the body	T	378231	5847	372384	0.469	-0.757062	1.00365	0.0656	3.35	0.450663	0.998648
Medication	Fish oil including cod liver oil	T	377850	119641	258209	1.03	0.024893	0.181749	0.718	1.46	0.891062	0.998648
Medication	Calcium	T	377850	25583	352267	1.09	0.08213	0.334131	0.564	2.09	0.805835	0.998648
Medication	Zinc	T	377850	15460	362390	0.499	-0.695071	0.583819	0.159	1.57	0.233827	0.998648
Medication	Selenium	T	377850	9070	368780	1.17	0.154095	0.507733	0.431	3.16	0.761512	0.998648
Cardiovascular	Hypertension (HES and self-reported)	T	379101	98025	281076	1.03	0.033992	0.194949	0.706	1.52	0.861578	0.998648
Cardiovascular	Heartcardiac problem (HES and self-reported)	T	379101	25675	353426	0.692	-0.367631	0.39514	0.319	1.5	0.352173	0.998648
Cardiovascular	Peripheral vascular disease (HES and self-reported)	T	379101	1859	377242	3.71E-05	-10.203	118.904	2.27E-106	6.05E+96	0.931619	0.998648
Cardiovascular	Venous thromboembolic disease (HES and self-reported)	T	379101	3983	375118	1.32	0.279195	0.713007	0.327	5.35	0.695373	0.998648
Cardiovascular	Essential hypertension (HES and self-reported)	T	379101	71076	308025	0.915	-0.088761	0.227943	0.585	1.43	0.696982	0.998648
Cardiovascular	Gestational hypertensionpreeclampsia (HES and self-reported)	T	379101	2534	376567	3.74E-05	-10.1929	107.609	9.43E-97	1.49E+87	0.924536	0.998648
Cardiovascular	Heart attackmyocardial infarction (HES and self-reported)	T	379101	12068	367033	0.632	-0.458306	0.589614	0.199	2.01	0.436983	0.998648
Cardiovascular	Heart failurepulmonary odema (HES and self-reported)	T	379101	5221	373880	3.68E-05	-10.2089	70.1286	7.44E-65	1.82E+55	0.884257	0.998648
Cardiovascular	Heart arrhythmia (HES and self-reported)	T	379101	21190	357911	1.26	0.234766	0.333938	0.657	2.43	0.482041	0.998648
Cardiovascular	Heart valve problemheart murmur (HES and self-reported)	T	379101	7532	371569	1.8	0.588838	0.457061	0.736	4.41	0.197637	0.998648
Cardiovascular	Cardiomyopathy (HES and self-reported)	T	379101	1272	377829	5.14E-05	-9.87492	119.07	2.27E-106	1.16E+97	0.933904	0.998648
Cardiovascular	Pericardial problem (HES and self-reported)	T	379101	1268	377833	2.06	0.721236	1.00492	0.287	14.7	0.472938	0.998648
Cardiovascular	Stroke (HES and self-reported)	T	379101	8571	370530	1.58	0.460568	0.458677	0.645	3.89	0.31532	0.998648
Cardiovascular	Transient ischaemic attack tia (HES and self-reported)	T	379101	3570	375531	0.759	-0.275716	1.00566	0.106	5.45	0.783958	0.998648
Cardiovascular	Subdural haemorrhagehaematoma (HES and self-reported)	T	379101	380	378721	6.67E-05	-9.61519	194.83	9.58E-171	4.64E+161	0.960639	0.998648
Cardiovascular	Arterial embolism (HES and self-reported)	T	379101	762	378339	9.70E-05	-9.24103	115.869	2.28E-103	4.13E+94	0.936433	0.998648
Cardiovascular	Deep venous thrombosis dvt (HES and self-reported)	T	379101	8917	370184	1.52	0.417009	0.456424	0.62	3.71	0.360904	0.998648
Respiratory	Interstitial lung disease (HES and self-reported)	T	379101	1083	378018	6.66E-05	-9.61722	116.837	2.34E-104	1.89E+95	0.934398	0.998648
Respiratory	Asbestosis (HES and self-reported)	T	379101	271	378830	4.11E-05	-10.0993	286.554	4.95E-249	3.42E+239	0.971885	0.998648
Respiratory	Pulmonary fibrosis (HES and self-reported)	T	379101	789	378312	9.40E-05	-9.27219	116.115	1.36E-103	6.49E+94	0.936354	0.998648
ENT	Sleep apnoea (HES and self-reported)	T	379101	4559	374542	0.554	-0.591111	1.00432	0.0773	3.96	0.556149	0.998648
Digestive system	Oesophageal disorder (HES and self-reported)	T	379101	9080	370021	1.19	0.171365	0.509036	0.438	3.22	0.736384	0.998648
Digestive system	Stomach disorder (HES and self-reported)	T	379101	7348	371753	1.53	0.426665	0.508251	0.566	4.15	0.401202	0.998648
Digestive system	Other abdominal problem (HES and self-reported)	T	379101	39890	339211	1.09	0.089126	0.266727	0.648	1.84	0.738268	0.998648
Digestive system	Gastrooesophageal reflux gord gastric reflux (HES and self-reported)	T	379101	32601	346500	0.91	-0.094823	0.314802	0.491	1.69	0.763251	0.998648
Digestive system	Oesophagitisbarretts oesophagus (HES and self-reported)	T	379101	10521	368580	1.02	0.021674	0.508528	0.377	2.77	0.966004	0.998648
Digestive system	Oesophageal stricture (HES and self-reported)	T	379101	1781	377320	3.84E-05	-10.1665	118.796	2.91E-106	5.08E+96	0.9318	0.998648
Digestive system	Oesophageal varicies (HES and self-reported)	T	379101	501	378600	5.05E-05	-9.89441	195.924	8.49E-172	3.00E+162	0.959723	0.998648
Digestive system	Gastricstomach ulcers (HES and self-reported)	T	379101	6128	372973	0.898	-0.107482	0.713436	0.222	3.64	0.880249	0.998648
Digestive system	Gastritisgastric erosions (HES and self-reported)	T	379101	21127	357974	1.06	0.06186	0.365356	0.52	2.18	0.865549	0.998648
Digestive system	Irritable bowel syndrome (HES and self-reported)	T	379101	12379	366722	0.665	-0.408564	0.584598	0.211	2.09	0.484626	0.998648
Digestive system	Hepatitis (HES and self-reported)	T	379101	1662	377439	4.08E-05	-10.107	120.708	7.28E-108	2.29E+98	0.93327	0.998648
Digestive system	Infectiveviral hepatitis (HES and self-reported)	T	379101	1240	377861	5.37E-05	-9.83251	120.723	9.30E-108	3.10E+98	0.935087	0.998648
Digestive system	Noninfective hepatitis (HES and self-reported)	T	379101	1195	377906	5.72E-05	-9.76918	120.754	9.32E-108	3.51E+98	0.935521	0.998648
Digestive system	Bile duct obstructionascending cholangitis (HES and self-reported)	T	379101	936	378165	7.12E-05	-9.54959	120.118	4.04E-107	1.26E+98	0.936633	0.998648
Digestive system	Cholelithiasisgall stones (HES and self-reported)	T	379101	16555	362546	1.56	0.446478	0.346968	0.792	3.08	0.198165	0.998648
Digestive system	Cholecystitis (HES and self-reported)	T	379101	2791	376310	6.76E-05	-9.60143	72.9683	5.23E-67	8.75E+57	0.895314	0.998648
Digestive system	Peritonitis (HES and self-reported)	T	379101	1989	377112	2.7	0.991897	0.712938	0.667	10.9	0.16414	0.998648
Digestive system	Gastrointestinal bleeding (HES and self-reported)	T	379101	8293	370808	1.64	0.49299	0.456031	0.67	4	0.279677	0.998648
Genitourinary	Renalkidney failure (HES and self-reported)	T	379101	8356	370745	0.958	-0.042568	0.586798	0.303	3.03	0.94217	0.998648
Genitourinary	Renal failure requiring dialysis (HES and self-reported)	T	379101	660	378441	0.000101	-9.19719	120.001	7.22E-107	1.42E+98	0.938908	0.998648
Genitourinary	Urinary tract infectionkidney infection (HES and self-reported)	T	379101	12655	366446	1.09	0.085028	0.456433	0.445	2.66	0.85222	0.998648
Genitourinary	Kidney stoneureter stonebladder stone (HES and self-reported)	T	379101	6694	372407	2.62E-05	-10.5503	72.3144	7.29E-67	9.40E+56	0.884004	0.998648
Genitourinary	Ureteric obstructionhydronephrosis (HES and self-reported)	T	379101	2626	376475	0.989	-0.010589	1.00429	0.138	7.08	0.991587	0.998648
Genitourinary	Bladder problem not cancer (HES and self-reported)	T	379101	10748	368353	1.23	0.208566	0.457707	0.502	3.02	0.648623	0.998648
Genitourinary	Urinary frequency incontinence (HES and self-reported)	T	379101	11734	367367	0.953	-0.048216	0.508625	0.352	2.58	0.924476	0.998648
Genitourinary	Scrotal problem not cancer (HES and self-reported)	T	379101	1382	377719	4.83E-08	-16.8457	3646.67	0	Inf	0.996314	0.998648
Genitourinary	Testicular problems not cancer (HES and self-reported)	T	379101	1283	377818	1.9	0.640398	1.00749	0.263	13.7	0.525012	0.998648
Metabolic	Diabetes (HES and self-reported)	T	379101	15907	363194	0.482	-0.729971	0.585752	0.153	1.52	0.212687	0.998648
Metabolic	Gestational diabetes (HES and self-reported)	T	379101	363	378738	8.20E-05	-9.40836	185.127	2.14E-162	3.14E+153	0.959468	0.998648
Metabolic	Type 1 diabetes (HES and self-reported)	T	379101	2663	376438	0.978	-0.022031	1.00407	0.137	7	0.982494	0.998648
Metabolic	Hyperthyroidismthyrotoxicosis (HES and self-reported)	T	379101	4020	375081	0.693	-0.366494	1.00496	0.0967	4.97	0.715348	0.998648
Metabolic	Hypothyroidismmyxoedema (HES and self-reported)	T	379101	22541	356560	0.734	-0.309824	0.421698	0.321	1.68	0.462519	0.998648
Metabolic	Thyroid radioablation therapy (HES and self-reported)	T	379101	2506	376595	1.98	0.685061	0.715262	0.488	8.06	0.338175	0.998648
Metabolic	Parathyroid gland problem not cancer (HES and self-reported)	T	379101	274	378827	9.41E-05	-9.27164	196.535	4.78E-172	1.85E+163	0.962373	0.998648
Metabolic	Parathyroid hyperplasiaadenoma (HES and self-reported)	T	379101	419	378682	6.66E-05	-9.61743	193.251	2.11E-169	2.10E+160	0.960308	0.998648
Metabolic	Disorder of adrenal gland (HES and self-reported)	T	379101	470	378631	5.29E-05	-9.84678	199.107	1.74E-174	1.61E+165	0.960557	0.998648
Metabolic	Adrenocortical insufficiencyaddisons disease (HES and self-reported)	T	379101	349	378752	7.00E-05	-9.56721	198.908	3.40E-174	1.44E+165	0.961638	0.998648
Metabolic	Disorder or pituitary gland (HES and self-reported)	T	379101	409	378692	6.21E-05	-9.68683	199.281	1.45E-174	2.66E+165	0.961231	0.998648
Metabolic	Pituitary adenomatumour (HES and self-reported)	T	379101	466	378635	5.34E-05	-9.83699	199.359	1.07E-174	2.66E+165	0.960646	0.998648
Eye	Eyeeyelid problem (HES and self-reported)	T	379101	17843	361258	0.897	-0.1092	0.418214	0.395	2.04	0.794007	0.998648
Neurosciences	Psychologicalpsychiatric prob (HES and self-reported)	T	379101	2225	376876	8.17E-05	-9.41305	73.421	2.60E-67	2.57E+58	0.897986	0.998648
Neurosciences	Infection of nervous system (HES and self-reported)	T	379101	529	378572	4.55E-05	-9.99841	199.567	6.07E-175	3.41E+165	0.960042	0.998648
Neurosciences	Encephalitis (HES and self-reported)	T	379101	415	378686	5.77E-05	-9.75995	199.731	5.58E-175	5.97E+165	0.961027	0.998648
Neurosciences	Meningitis (HES and self-reported)	T	379101	1714	377387	3.73E-05	-10.1954	121.105	3.06E-108	4.56E+98	0.932908	0.998648
Neurosciences	Cranial nerve problempalsy (HES and self-reported)	T	379101	1916	377185	9.46E-05	-9.26606	73.1864	4.77E-67	1.88E+58	0.89925	0.998648
Neurosciences	Bells palsyfacial nerve palsy (HES and self-reported)	T	379101	922	378179	7.13E-05	-9.54886	121.025	6.83E-108	7.44E+98	0.937112	0.998648
Neurosciences	Spinal cord disorder (HES and self-reported)	T	379101	1886	377215	9.52E-05	-9.25939	73.3121	3.75E-67	2.42E+58	0.899494	0.998648
Neurosciences	Paraplegia (HES and self-reported)	T	379101	312	378789	7.77E-05	-9.46272	197.777	3.46E-173	1.74E+164	0.961839	0.998648
Neurosciences	Peripheral neuropathy (HES and self-reported)	T	379101	2013	377088	1.31	0.269375	1.00445	0.183	9.38	0.788559	0.998648
Neurosciences	Acute infective polyneuritisguillainbarre syndro (HES and self-reported)	T	379101	290	378811	8.54E-05	-9.36848	198.623	7.24E-174	1.01E+165	0.96238	0.998648
Neurosciences	Trapped nervecompressed nerve (HES and self-reported)	T	379101	15824	363277	0.497	-0.700086	0.583936	0.158	1.56	0.230563	0.998648
Neurosciences	Chronicdegenerative neurological problem (HES and self-reported)	T	379101	4087	375014	2	0.692841	0.584602	0.636	6.29	0.235958	0.998648
Neurosciences	Motor neurone disease (HES and self-reported)	T	379101	231	378870	0.000106	-9.15291	196.013	1.50E-171	7.49E+162	0.962756	0.998648
Neurosciences	Multiple sclerosis (HES and self-reported)	T	379101	1635	377466	1.65	0.503592	1.00447	0.231	11.9	0.616124	0.998648
Neurosciences	Dementiaalzheimerscognitive impairment (HES and self-reported)	T	379101	906	378195	3.04	1.11099	1.00908	0.42	22	0.270898	0.998648
Neurosciences	Epilepsy (HES and self-reported)	T	379101	4619	374482	1.15	0.143389	0.712335	0.286	4.66	0.840468	0.998648
Neurosciences	Migraine (HES and self-reported)	T	379101	12905	366196	1.04	0.042475	0.457548	0.426	2.56	0.926037	0.998648
Eye	Eye infection (HES and self-reported)	T	379101	2800	376301	1.89	0.638915	0.712439	0.469	7.65	0.369826	0.998648
Eye	Retinal problem (HES and self-reported)	T	379101	7219	371882	1.08	0.072375	0.585553	0.341	3.39	0.901632	0.998648
Eye	Diabetic eye disease (HES and self-reported)	T	379101	1737	377364	3.87E-05	-10.1599	118.892	2.42E-106	6.17E+96	0.9319	0.998648
Eye	Glaucoma (HES and self-reported)	T	379101	6262	372839	1.27	0.24001	0.586414	0.403	4.01	0.68233	0.998648
Eye	Cataract (HES and self-reported)	T	379101	20791	358310	1.2	0.184635	0.352693	0.603	2.4	0.600626	0.998648
Eye	Eye trauma (HES and self-reported)	T	379101	816	378285	8.22E-05	-9.4067	119.373	2.01E-106	3.37E+97	0.937191	0.998648
Eye	Retinal detachment (HES and self-reported)	T	379101	3632	375469	0.678	-0.388782	1.00458	0.0946	4.86	0.698748	0.998648
Mental health	Depression (HES and self-reported)	T	379101	27882	351219	0.873	-0.135379	0.345025	0.444	1.72	0.694781	0.998648
Mental health	Anxietypanic attacks (HES and self-reported)	T	379101	10392	368709	0.79	-0.235347	0.583784	0.252	2.48	0.686845	0.998648
Mental health	Nervous breakdown (HES and self-reported)	T	379101	629	378472	0.00011	-9.11279	120.901	1.35E-107	9.02E+98	0.939917	0.998648
Mental health	Schizophrenia (HES and self-reported)	T	379101	686	378415	9.91E-05	-9.21943	119.614	1.51E-106	6.51E+97	0.938563	0.998648
Mental health	Deliberate selfharmsuicide attempt (HES and self-reported)	T	379101	2635	376466	1.07	0.070055	1.00454	0.15	7.68	0.944402	0.998648
Mental health	Maniabipolar disordermanic depression (HES and self-reported)	T	379101	1595	377506	4.21E-05	-10.0755	121.04	3.92E-108	4.52E+98	0.93366	0.998648
Musculoskeletal	Bone disorder (HES and self-reported)	T	379101	5041	374060	1.1	0.094457	0.712566	0.272	4.44	0.894542	0.998648
Musculoskeletal	Back problem (HES and self-reported)	T	379101	11524	367577	0.927	-0.075914	0.507379	0.343	2.51	0.881065	0.998648
Musculoskeletal	Joint disorder (HES and self-reported)	T	379101	27274	351827	1.17	0.159322	0.30175	0.649	2.12	0.597503	0.998648
Musculoskeletal	Musclesoft tissue problem (HES and self-reported)	T	379101	32458	346643	1.27	0.241318	0.273429	0.745	2.18	0.377474	0.998648
Musculoskeletal	Osteomyelitis (HES and self-reported)	T	379101	1062	378039	6.31E-05	-9.67033	120.238	2.83E-107	1.41E+98	0.935898	0.998648

Musculoskeletal	Pagets disease (HES and self-reported)	T	379101	272	378829	9.44E-05	-9.26828	197.317	1.04E-172	8.60E+163	0.962536	0.998648
Musculoskeletal	Spine arthritisspondylitis (HES and self-reported)	T	379101	10404	368697	0.538	-0.620245	0.713146	0.133	2.18	0.384447	0.998648
Musculoskeletal	Prolapsed discslipped disc (HES and self-reported)	T	379101	8220	370881	0.969	-0.031826	0.583617	0.309	3.04	0.956511	0.998648
Musculoskeletal	Ankylosing spondylitis (HES and self-reported)	T	379101	1308	377793	5.12E-05	-9.87941	120.325	1.94E-107	1.36E+98	0.934562	0.998648
Musculoskeletal	Myositismyopathy (HES and self-reported)	T	379101	338	378763	7.26E-05	-9.53044	199.339	1.51E-174	3.48E+165	0.961867	0.998648
Haematology	Low plateletsplatelet disorder (HES and self-reported)	T	379101	1618	377483	4.18E-05	-10.0835	120.566	9.84E-108	1.77E+98	0.933347	0.998648
Haematology	Iron deficiency anaemia (HES and self-reported)	T	379101	8597	370504	1.62	0.479385	0.455903	0.661	3.95	0.293026	0.998648
Haematology	Pernicious anaemia (HES and self-reported)	T	379101	1499	377602	5.06E-05	-9.89228	119.144	1.93E-106	1.32E+97	0.933829	0.998648
Haematology	Aplastic anaemia (HES and self-reported)	T	379101	429	378672	6.01E-05	-9.71898	196.316	4.69E-172	7.70E+162	0.960515	0.998648
Gynaecology and Obstetrics	Gynaecological disorder not c (HES and self-reported)	T	379101	16832	362269	1.03	0.027452	0.427006	0.445	2.37	0.94874	0.998648
Gynaecology and Obstetrics	Ovarian cyst or cysts (HES and self-reported)	T	379101	6134	372967	3.41E-08	-17.194	2203.33	0	Inf	0.993774	0.998648
Gynaecology and Obstetrics	Polycystic ovariespolycystic ovarian syndrome (HES and self-reported)	T	379101	580	378521	6.44E-08	-16.558	5715.8	0	Inf	0.997689	0.998648
Gynaecology and Obstetrics	Uterine fibroids (HES and self-reported)	T	379101	13674	365427	5.99E-06	-12.0252	109.115	7.89E-99	4.55E+87	0.912245	0.998648
Gynaecology and Obstetrics	Uterine polyps (HES and self-reported)	T	379101	8016	371085	1.43	0.358289	0.515108	0.521	3.93	0.486704	0.998648
Gynaecology and Obstetrics	Breast disease not cancer (HES and self-reported)	T	379101	3704	375397	0.795	-0.229754	1.00636	0.111	5.71	0.819412	0.998648
Gynaecology and Obstetrics	Fibrocystic disease (HES and self-reported)	T	379101	1743	377358	4.99E-05	-9.90514	111.372	7.88E-100	3.16E+90	0.929132	0.998648
Gynaecology and Obstetrics	Breast cysts (HES and self-reported)	T	379101	1673	377428	5.38E-05	-9.82981	110.975	1.85E-99	1.57E+90	0.929419	0.998648
Immuno-inflammation	Sarcoidosis (HES and self-reported)	T	379101	930	378171	6.70E-05	-9.61079	120.997	6.78E-108	6.62E+98	0.93669	0.998648
Musculoskeletal	Vasculitis (HES and self-reported)	T	379101	1756	377345	1.56	0.442687	1.00576	0.217	11.2	0.659828	0.998648
Immuno-inflammation	Connective tissue disorder (HES and self-reported)	T	379101	2304	376797	1.2	0.185095	1.00527	0.168	8.63	0.853915	0.998648
Immuno-inflammation	Allergyhypersensitivityanaph (HES and self-reported)	T	379101	3101	376000	0.902	-0.10319	1.00396	0.126	6.45	0.918135	0.998648
Musculoskeletal	Giant celltemporal arteritis (HES and self-reported)	T	379101	395	378706	7.48E-05	-9.50122	188.67	1.88E-165	2.97E+156	0.959836	0.998648
Musculoskeletal	Polymyalgia rheumatica (HES and self-reported)	T	379101	1540	377561	4.92E-05	-9.92014	114.771	9.92E-103	2.44E+93	0.931121	0.998648
Musculoskeletal	Systemic lupus erythematosissle (HES and self-reported)	T	379101	547	378554	5.51E-05	-9.806	192.993	2.90E-169	1.05E+160	0.959477	0.998648
Musculoskeletal	Sjogrens syndromesicca syndrome (HES and self-reported)	T	379101	614	378487	5.01E-05	-9.90225	189.163	4.79E-166	5.23E+156	0.958252	0.998648
Musculoskeletal	Sclerodermasystemic sclerosis (HES and self-reported)	T	379101	201	378900	5.02E-05	-9.90029	318.111	8.29E-276	3.03E+266	0.975172	0.998648
Immuno-inflammation	Allergy or anaphylactic reaction to drug (HES and self-reported)	T	379101	4200	374901	4.39E-05	-10.0338	73.2011	2.15E-67	8.96E+57	0.890974	0.998648
Immuno-inflammation	Hayfeverallergic rhinitis (HES and self-reported)	T	379101	22257	356844	1.27	0.24143	0.31463	0.687	2.36	0.442875	0.998648
Neurosciences	Other demyelinating disease not multiple sclerosi (HES and self-reported)	T	379101	1324	377777	5.11E-05	-9.88262	120.269	2.15E-107	1.21E+98	0.934511	0.998648
Digestive system	Peptic ulcer (HES and self-reported)	T	379101	770	378331	3.61	1.28245	1.00625	0.502	25.9	0.202495	0.998648
Gynaecology and Obstetrics	Endometriosis (HES and self-reported)	T	379101	5960	373141	0.484	-0.724908	1.00833	0.0671	3.5	0.472189	0.998648
Gynaecology and Obstetrics	Female infertility (HES and self-reported)	T	379101	1328	377773	7.90E-08	-16.354	3480.95	0	Inf	0.996251	0.998648
Genitourinary	Other renalkidney problem (HES and self-reported)	T	379101	5423	373678	3.26E-05	-10.3324	72.9979	2.37E-67	4.46E+57	0.88744	0.998648
Musculoskeletal	Muscle or soft tissue injuries (HES and self-reported)	T	379101	9386	369715	0.561	-0.577867	0.71193	0.139	2.26	0.416969	0.998648
Musculoskeletal	Burns (HES and self-reported)	T	379101	765	378336	3.4	1.22441	1.00478	0.475	24.4	0.223003	0.998648
Other	Other substance abusedependency (HES and self-reported)	T	379101	10328	368773	0.786	-0.240826	0.584511	0.25	2.47	0.68033	0.998648
Respiratory	Bronchitis (HES and self-reported)	T	379101	3358	375743	1.6	0.468215	0.712538	0.395	6.45	0.51111	0.998648
ENT	Throat or larynx disorder (HES and self-reported)	T	379101	3484	375617	5.29E-05	-9.8474	73.4089	1.72E-67	1.62E+58	0.893288	0.998648
ENT	Earvestibular disorder (HES and self-reported)	T	379101	12078	367023	0.884	-0.122956	0.50765	0.327	2.39	0.80862	0.998648
ENT	Chronic sinusitis (HES and self-reported)	T	379101	4361	374740	0.604	-0.50413	1.00355	0.0845	4.32	0.615423	0.998648
ENT	Chronic laryngitis (HES and self-reported)	T	379101	1014	378087	6.94E-05	-9.57496	119.614	1.06E-106	4.56E+97	0.936198	0.998648
ENT	Vocal cord polyp (HES and self-reported)	T	379101	392	378709	6.72E-05	-9.60749	198.354	9.66E-174	4.68E+164	0.961369	0.998648
ENT	Otosclerosis (HES and self-reported)	T	379101	416	378685	5.78E-05	-9.75819	199.274	1.37E-174	2.44E+165	0.960944	0.998648
ENT	Menieres disease (HES and self-reported)	T	379101	1305	377796	5.31E-05	-9.8424	120.099	3.13E-107	9.03E+97	0.934685	0.998648
Genitourinary	Polycystic kidney (HES and self-reported)	T	379101	1630	377471	4.13E-05	-10.0936	119.217	1.37E-106	1.25E+97	0.932527	0.998648
ENT	Thyroiditis (HES and self-reported)	T	379101	442	378659	6.33E-05	-9.66736	192.333	1.21E-168	3.30E+159	0.959912	0.998648
Metabolic	Hypopituitarism (HES and self-reported)	T	379101	446	378655	5.59E-05	-9.79206	198.972	2.39E-174	1.31E+165	0.960749	0.998648
Neurosciences	Other neurological problem (HES and self-reported)	T	379101	8378	370723	0.954	-0.046694	0.583762	0.304	3	0.936247	0.998648
Neurosciences	Myasthenia gravis (HES and self-reported)	T	379101	206	378895	0.000121	-9.01975	198.008	3.43E-173	4.27E+164	0.963667	0.998648
Infectious disease	Hivaids (HES and self-reported)	T	379101	309	378792	8.41E-05	-9.3833	183.133	1.09E-160	6.47E+151	0.959136	0.998648
Infectious disease	Tuberculosis tb (HES and self-reported)	T	379101	1905	377196	3.81E-05	-10.1761	117.845	1.86E-105	7.80E+95	0.931187	0.998648
Infectious disease	Malaria (HES and self-reported)	T	379101	480	378621	4.83E-05	-9.9388	198.499	5.22E-174	4.46E+164	0.960067	0.998648
Infectious disease	Helicobacter pylori (HES and self-reported)	T	379101	1773	377328	4.35E-05	-10.0436	118.938	2.49E-106	7.59E+96	0.932704	0.998648
Haematology	Clotting disorderexcessive bleeding (HES and self-reported)	T	379101	2706	376395	6.72E-05	-9.60717	73.3476	2.47E-67	1.83E+58	0.89579	0.998648
Haematology	Anaemia (HES and self-reported)	T	379101	17268	361833	1.46	0.377229	0.34526	0.741	2.87	0.274572	0.998648
Haematology	Pancytopenia (HES and self-reported)	T	379101	406	378695	6.36E-05	-9.66228	196.084	7.83E-172	5.17E+162	0.960699	0.998648
Haematology	Neutropenialymphopenia (HES and self-reported)	T	379101	2972	376129	6.23E-05	-9.68284	73.0829	3.85E-67	1.01E+58	0.894596	0.998648
Haematology	Hereditarygenetic haematological disorder (HES and self-reported)	T	379101	463	378638	5.33E-05	-9.83927	199.194	1.48E-174	1.92E+165	0.960604	0.998648
Immuno-inflammation	Eczemadermatitis (HES and self-reported)	T	379101	11296	367805	0.679	-0.387134	0.583653	0.216	2.13	0.507141	0.998648
Immuno-inflammation	Psoriasis (HES and self-reported)	T	379101	5583	373518	1.45	0.373993	0.583929	0.463	4.57	0.521862	0.998648
Immuno-inflammation	Blisteringdesquamating skin disorder (HES and self-reported)	T	379101	748	378353	8.70E-05	-9.35016	121.086	7.39E-108	1.02E+99	0.938449	0.998648
Digestive system	Malabsorptioncoeliac disease (HES and self-reported)	T	379101	2523	376578	7.17E-05	-9.54313	73.1688	3.74E-67	1.37E+58	0.896229	0.998648
Digestive system	Duodenal ulcer (HES and self-reported)	T	379101	4066	375035	1.34	0.291191	0.714662	0.33	5.43	0.683676	0.998648
Digestive system	Diverticular diseasediverticulitis (HES and self-reported)	T	379101	26531	352570	1.03	0.033819	0.33178	0.54	1.98	0.918812	0.998648
Digestive system	Colitisnot crohns or ulcerative colitis (HES and self-reported)	T	379101	15433	363668	1.06	0.059081	0.417574	0.468	2.4	0.887486	0.998648
Digestive system	Rectal or colon adenomapolyps (HES and self-reported)	T	379101	28246	350855	1.15	0.144031	0.303818	0.637	2.09	0.635451	0.998648
Digestive system	Inflammatory bowel disease (HES and self-reported)	T	379101	4246	374855	4.21E-05	-10.0765	73.4694	1.22E-67	1.45E+58	0.89091	0.998648
Digestive system	Crohns disease (HES and self-reported)	T	379101	1818	377283	3.63E-05	-10.2229	121.214	2.40E-108	5.49E+98	0.932788	0.998648
Digestive system	Ulcerative colitis (HES and self-reported)	T	379101	3555	375546	5.06E-05	-9.89186	73.4131	1.64E-67	1.56E+58	0.892815	0.998648
Musculoskeletal	Rheumatoid arthritis (HES and self-reported)	T	379101	6232	372869	0.908	-0.096913	0.713303	0.224	3.67	0.891928	0.998648
Musculoskeletal	Osteoarthritis (HES and self-reported)	T	379101	58523	320578	0.818	-0.20146	0.256627	0.494	1.35	0.432436	0.998648
Musculoskeletal	Gout (HES and self-reported)	T	379101	6858	372243	0.712	-0.33979	0.717124	0.175	2.9	0.635625	0.998648
Musculoskeletal	Other joint disorder (HES and self-reported)	T	379101	9807	369294	1.41	0.344377	0.455495	0.578	3.45	0.44962	0.998648
Neurosciences	Diabetic neuropathyulcers (HES and self-reported)	T	379101	546	378555	4.64E-05	-9.97884	195.135	3.66E-171	5.87E+161	0.959215	0.998648
Neurosciences	Posttraumatic stress disorder (HES and self-reported)	T	379101	723	378378	9.23E-05	-9.29026	120.178	4.65E-107	1.83E+98	0.938381	0.998648
Neurosciences	Anorexiabulimiaother eating disorder (HES and self-reported)	T	379101	336	378765	8.96E-05	-9.32056	185.892	5.22E-163	1.54E+154	0.960011	0.998648
Digestive system	Hiatus hernia (HES and self-reported)	T	379101	27678	351423	0.676	-0.391336	0.389132	0.315	1.45	0.314577	0.998648
Digestive system	Sclerosing cholangitis (HES and self-reported)	T	379101	454	378647	5.61E-05	-9.78832	195.459	2.35E-171	1.34E+162	0.96006	0.998648
Musculoskeletal	Sciatica (HES and self-reported)	T	379101	5455	373646	0.489	-0.715012	1.00354	0.0684	3.5	0.47616	0.998648
Musculoskeletal	Psoriatic arthropathy (HES and self-reported)	T	379101	1087	378014	2.54	0.930912	1.00421	0.354	18.2	0.353922	0.998648
Musculoskeletal	Cervical spondylosis (HES and self-reported)	T	379101	4865	374236	1.19	0.174905	0.71331	0.294	4.82	0.8063	0.998648
Cardiovascular	Rheumatic fever (HES and self-reported)	T	379101	3026	376075	6.34E-05	-9.6657	71.5455	7.97E-66	5.05E+56	0.892534	0.998648
Immuno-inflammation	Chronic fatigue syndrome (HES and self-reported)	T	379101	1921	377180	1.44	0.367269	1.00449	0.202	10.3	0.714642	0.998648
Cardiovascular	Wolff parkinson white wpw syndrome (HES and self-reported)	T	379101	228	378873	0.000106	-9.14794	198.899	5.26E-174	2.15E+165	0.963316	0.998648
Cardiovascular	Irregular heart beat (HES and self-reported)	T	379101	1395	377706	4.87E-05	-9.9303	119.39	1.15E-106	2.06E+97	0.933712	0.998648
Cardiovascular	Sick sinus syndrome (HES and self-reported)	T	379101	373	378728	6.96E-05	-9.57299	192.736	6.06E-169	7.99E+159	0.960386	0.998648
Cardiovascular	Svt supraventricular tachycardia (HES and self-reported)	T	379101	2281	376820	2.35	0.856439	0.7129	0.582	9.52	0.229617	0.998648
Cardiovascular	Aortic stenosis (HES and self-reported)	T	379101	1728	377373	4.15E-05	-10.0897	115.471	2.12E-103	8.11E+93	0.930371	0.998648
Neurosciences	Brain haemorrhage (HES and self-reported)	T	379101	1057	378044	6.42E-05	-9.65423	118.472	9.16E-106	4.49E+96	0.935053	0.998648
Cardiovascular	Varicose veins (HES and self-reported)	T	379101	11798	367303	1.18	0.165192	0.455874	0.483	2.88	0.71708	0.998648
Cardiovascular	Lymphoedema (HES and self-reported)	T	379101	527	378574	4.80E-05	-9.94352	197.071	8.54E-173	2.70E+163	0.959759	0.998648
ENT	Labyrinthitis (HES and self-reported)	T	379101	839	378262	8.02E-05	-9.43143	120.901	9.79E-108	6.56E+98	0.93782	0.998648
ENT	Vertigo (HES and self-reported)	T	379101	697	378404	9.99E-05	-9.211	119.639	1.45E-106	6.89E+97	0.938631	0.998648
Digestive system	Pyloric stenosis (HES and self-reported)	T	379101	250	378851	9.69E-05	-9.24209	196.39	6.54E-172	1.43E+163	0.962465	0.998648
Digestive system	Appendicitis (HES and self-reported)	T	379101	6308	372793	1.67	0.510019	0.507381	0.616	4.5	0.3148	0.998648
Genitourinary	Anal fissure (HES and self-reported)	T	379101	269	378832	8.85E-05	-9.33215	199.412	1.60E-174	4.90E+165	0.962674	0.998648
Genitourinary	Haemorrhoids piles (HES and self-reported)	T	379101	1010	378091	2.53	0.928268	1.0052	0.353	18.1	0.355766	0.998648
Digestive system	Primary biliary cirrhosis (HES and self-reported)	T	379101	237	378864	0.000124	-8.99863	193.589	2.02E-169	7.55E+160	0.962925	0.998648
Digestive system	Jaundice unknown cause (HES and self-reported)	T	379101	1143	377958	5.82E-05	-9.751	120.104	3.39E-107	1.00E+98	0.935292	0.998648
Digestive system	Gastroenteritisdysentry (HES and self-reported)	T	379101	8121	370980	1.36	0.304866	0.507734	0.501	3.67	0.548211	0.998648
Digestive system	Dyspepsia indigestion (HES and self-reported)	T	379101	11162	367939	1.24	0.218264	0.456034	0.509	3.04	0.632214	0.998648
Digestive system	Abdominal hernia (HES and self-reported)	T	379101	20644	358457	0.96	-0.041288	0.370727	0.464	1.98	0.911324	0.998648
Digestive system	Umbilical hernia (HES and self-reported)	T	379101	3644	375457	0.681	-0.383663	1.00469	0.0951	4.88	0.702556	0.998648
Digestive system	Inguinal hernia (HES and self-reported)	T	379101	15351	363750	1.12	0.115877	0.398194	0.514	2.45	0.771046	0.998648
Genitourinary	Cystitis (HES and self-reported)	T	379101	2918	376183	0.96	-0.040558	1.00451	0.134	6.88	0.967794	0.998648
Genitourinary	Pyelonephritis (HES and self-reported)	T	379101	1141	377960	6.32E-05	-9.66984	120.168	3.25E-107	1.23E+98	0.935864	0.998648
Genitourinary	Prostatitis (HES and self-reported)	T	379101	1555	377546	1.57	0.450202	1.00926	0.217	11.3	0.655547	0.998648
Genitourinary	Erectile dysfunction impotence (HES and self-reported)	T	379101	497	378604	5.24E-08	-16.7641	5960.94	0	Inf	0.997756	0.998648
Genitourinary	Kidney nephropathy (HES and self-reported)	T	379101	417	378684	5.81E-05	-9.75359	197.158	8.71E-173	3.88E+163	0.960544	0.998648
Genitourinary	Iga nephropathy (HES and self-reported)	T	379101	666	378435	0.000102	-9.18612	119.557	1.74E-106	6.02E+97	0.938755	0.998648
Metabolic	Graves disease (HES and self-reported)	T	379101	476	378625	5.67E-05	-9.77828	194.776	9.05E-171	3.55E+161	0.959961	0.998648
Neurosciences	Trigemminal neuralgia (HES and self-reported)	T	379101	464	378637	5.64E-05	-9.78243	197.5	4.33E-173	7.36E+163	0.960496	0.998648
Neurosciences	Benign essential tremor (HES and self-reported)	T	379101	270	378831	9.42E-05	-9.27019	195.345	4.93E-171	1.80E+162	0.96215	0.998648
Neurosciences	Polio poliomyelitis (HES and self-reported)	T	379101	280	378821	8.49E-05	-9.37394	194.109	5.01E-170	1.44E+161	0.961483	0.998648
Eye	Macular degeneration (HES and self-reported)	T	379101	2130	376971	1.29	0.251588	1.00698	0.179	9.26	0.802709	0.998648

Eye	Dry eyes (HES and self-reported)	T	379101	847	378254	3.42	1.23095	1.00648	0.476	24.6	0.22132	0.998648
Musculoskeletal	Disc problem (HES and self-reported)	T	379101	8039	371062	0.657	-0.420531	0.711772	0.163	2.65	0.554639	0.998648
Musculoskeletal	Disc degeneration (HES and self-reported)	T	379101	2914	376187	0.918	-0.085692	1.00386	0.128	6.57	0.931974	0.998648
Musculoskeletal	Back pain (HES and self-reported)	T	379101	14806	364295	0.722	-0.325414	0.507325	0.267	1.95	0.521243	0.998648
Musculoskeletal	Spinal stenosis (HES and self-reported)	T	379101	3421	375680	0.778	-0.250947	1.00453	0.109	5.57	0.80273	0.998648
Musculoskeletal	Joint pain (HES and self-reported)	T	379101	8344	370757	1.64	0.497514	0.455594	0.673	4.02	0.274828	0.998648
Musculoskeletal	Arthritis nos (HES and self-reported)	T	379101	13143	365958	0.849	-0.164097	0.50922	0.313	2.3	0.747262	0.998648
Musculoskeletal	Plantar fascitis (HES and self-reported)	T	379101	448	378653	5.43E-05	-9.82154	199.54	7.63E-175	3.86E+165	0.960743	0.998648
Musculoskeletal	Carpal tunnel syndrome (HES and self-reported)	T	379101	9101	370000	0.29	-1.23638	1.004	0.0406	2.08	0.218156	0.998648
Musculoskeletal	Fibromyalgia (HES and self-reported)	T	379101	1223	377878	6.14E-05	-9.69747	116.859	2.07E-104	1.82E+95	0.933864	0.998648
Musculoskeletal	Dupuytrens contracture (HES and self-reported)	T	379101	3300	375801	5.79E-05	-9.75647	70.0539	1.35E-64	2.48E+55	0.889236	0.998648
Musculoskeletal	Neck probleminjury (HES and self-reported)	T	379101	520	378581	4.52E-05	-10.0049	199.464	7.37E-175	2.77E+165	0.959996	0.998648
Immuno-inflammation	Acneacne vulgaris (HES and self-reported)	T	379101	277	378824	9.29E-05	-9.28398	195.907	1.62E-171	5.34E+162	0.962203	0.998648
Immuno-inflammation	Lichen planus (HES and self-reported)	T	379101	627	378474	0.00012	-9.02506	119.344	3.11E-106	4.66E+97	0.93972	0.998648
Immuno-inflammation	Lichen sclerosis (HES and self-reported)	T	379101	542	378559	5.25E-05	-9.85497	190.223	6.29E-167	4.38E+157	0.958682	0.998648
Gynaecology and Obstetrics	Ovarian problem (HES and self-reported)	T	379101	4956	374145	4.41E-08	-16.9362	2192.04	0	Inf	0.993835	0.998648
Gynaecology and Obstetrics	Cervical problem (HES and self-reported)	T	379101	6385	372716	0.442	-0.816537	1.00748	0.0613	3.18	0.417668	0.998648
Gynaecology and Obstetrics	Cervical intraepithelial neoplasia cin precan (HES and self-reported)	T	379101	1670	377431	4.95E-08	-16.8204	3614.32	0	Inf	0.996287	0.998648
Gynaecology and Obstetrics	Cervical polyps (HES and self-reported)	T	379101	3066	376035	6.97E-08	-16.4797	2210.57	0	Inf	0.994052	0.998648
Gynaecology and Obstetrics	Menorrhagia unknown cause (HES and self-reported)	T	379101	12816	366285	1.19	0.176032	0.468702	0.476	2.99	0.707235	0.998648
Gynaecology and Obstetrics	Pelvic inflammatory disease pid (HES and self-reported)	T	379101	4037	375064	1.46	0.381105	0.719056	0.358	5.99	0.596107	0.998648
Gynaecology and Obstetrics	Ectopic pregnancy (HES and self-reported)	T	379101	539	378562	5.78E-08	-16.6659	5873.41	0	Inf	0.997736	0.998648
Gynaecology and Obstetrics	Miscarriage (HES and self-reported)	T	379101	1976	377125	4.56E-08	-16.9027	3519.92	0	Inf	0.996169	0.998648
Gynaecology and Obstetrics	Breast fibroadenoma (HES and self-reported)	T	379101	1450	377651	6.02E-05	-9.71793	111.226	1.26E-99	2.86E+90	0.930377	0.998648
Musculoskeletal	Raynauds phenomenondisease (HES and self-reported)	T	379101	1315	377786	5.30E-05	-9.84471	119.957	4.12E-107	6.82E+97	0.934592	0.998648
Immuno-inflammation	Food intolerance (HES and self-reported)	T	379101	306	378795	7.84E-05	-9.4541	197.811	3.27E-173	1.88E+164	0.961881	0.998648
Immuno-inflammation	Urticaria (HES and self-reported)	T	379101	530	378571	4.73E-05	-9.95919	199.326	1.01E-174	2.21E+165	0.960151	0.998648
Infectious disease	Infectious mononucleosis glandular fever epste (HES and self-reported)	T	379101	517	378584	4.31E-05	-10.0527	197.975	1.30E-173	1.43E+164	0.959503	0.998648
Infectious disease	Measles morbillivirus (HES and self-reported)	T	379101	1296	377805	1.87	0.627701	1.00431	0.262	13.4	0.531968	0.998648
Infectious disease	Mumps epidemic parotitis (HES and self-reported)	T	379101	830	378271	2.9	1.06515	1.00463	0.405	20.8	0.289034	0.998648
Infectious disease	Rubella german measles (HES and self-reported)	T	379101	452	378649	4.95E-05	-9.91286	197.507	3.75E-173	6.55E+163	0.959971	0.998648
Infectious disease	Chickenpox (HES and self-reported)	T	379101	1557	377544	1.54	0.433447	1.00434	0.215	11	0.66605	0.998648
Infectious disease	Shingles (HES and self-reported)	T	379101	734	378367	3.65	1.29446	1.00492	0.509	26.2	0.197702	0.998648
Infectious disease	Herpes simplex (HES and self-reported)	T	379101	373	378728	6.59E-05	-9.62688	199.509	9.85E-175	4.41E+165	0.961515	0.998648
Infectious disease	Hepatitis c (HES and self-reported)	T	379101	331	378770	7.92E-05	-9.44345	194.792	1.23E-170	5.12E+161	0.961334	0.998648
Cardiovascular	Ischaemic stroke (HES and self-reported)	T	379101	2968	376133	1.82	0.598545	0.714984	0.448	7.39	0.402512	0.998648
Cardiovascular	Mitral regurgitation incompetence (HES and self-reported)	T	379101	2024	377077	2.65	0.973305	0.71497	0.652	10.7	0.173411	0.998648
Cardiovascular	Aortic valve disease (HES and self-reported)	T	379101	2405	376696	1.1	0.092737	1.00627	0.153	7.89	0.926572	0.998648
Cardiovascular	Aortic regurgitation incompetence (HES and self-reported)	T	379101	711	378390	3.71	1.3118	1.00593	0.517	26.7	0.19221	0.998648
Cardiovascular	Hypertrophic cardiomyopathy hcm hocm (HES and self-reported)	T	379101	265	378836	9.21E-05	-9.29304	196.543	4.61E-172	1.84E+163	0.962288	0.998648
Cardiovascular	Pericarditis (HES and self-reported)	T	379101	1193	377908	2.17	0.774761	1.005	0.303	15.6	0.440761	0.998648
Cardiovascular	Varicose ulcer (HES and self-reported)	T	379101	232	378869	0.000109	-9.12827	195.625	3.28E-171	3.59E+162	0.962783	0.998648
Respiratory	Respiratory infection (HES and self-reported)	T	379101	9656	369445	1.72	0.541402	0.417957	0.757	3.9	0.195199	0.998648
Respiratory	Pleural plaques not known asbestosis (HES and self-reported)	T	379101	690	378411	3.83	1.34379	1.01301	0.526	27.9	0.184666	0.998648
ENT	Tinnitus tiniitis (HES and self-reported)	T	379101	1088	378013	6.03E-05	-9.71655	120.024	4.11E-107	8.84E+97	0.935478	0.998648
ENT	Tonsiltis (HES and self-reported)	T	379101	3534	375567	4.87E-05	-9.93013	73.2454	2.19E-67	1.08E+58	0.892159	0.998648
Digestive system	Constipation (HES and self-reported)	T	379101	9731	369370	0.563	-0.573647	0.71221	0.14	2.28	0.420562	0.998648
Digestive system	Bowel intestinal perforation (HES and self-reported)	T	379101	387	378714	6.66E-05	-9.61692	197.703	3.43E-173	1.29E+164	0.961204	0.998648
Digestive system	Bowel intestinal infarction (HES and self-reported)	T	379101	264	378837	0.000101	-9.19561	196.723	3.57E-172	2.89E+163	0.962717	0.998648
Digestive system	Bowel intestinal obstruction (HES and self-reported)	T	379101	3937	375164	0.69	-0.370579	1.00393	0.0965	4.94	0.712033	0.998648
Digestive system	Rectal prolapse (HES and self-reported)	T	379101	1030	378071	7.18E-05	-9.54093	118.309	1.41E-105	3.66E+96	0.935725	0.998648
Digestive system	Alcoholic liver disease alcoholic cirrhosis (HES and self-reported)	T	379101	824	378277	3.25	1.17931	1.00763	0.451	23.4	0.241846	0.998648
Digestive system	Femoral hernia (HES and self-reported)	T	379101	643	378458	0.00011	-9.11801	119.528	1.98E-106	6.09E+97	0.939194	0.998648
Genitourinary	Diabetic nephropathy (HES and self-reported)	T	379101	242	378859	0.000103	-9.18119	194.167	5.42E-170	1.95E+161	0.962286	0.998648
Genitourinary	Nephritis (HES and self-reported)	T	379101	1824	377277	3.72E-05	-10.2004	119.378	8.98E-107	1.54E+97	0.931906	0.998648
Genitourinary	Glomerulnephritis (HES and self-reported)	T	379101	1746	377355	3.90E-05	-10.1526	119.351	9.93E-107	1.53E+97	0.93221	0.998648
Metabolic	Hyperparathyroidism (HES and self-reported)	T	379101	788	378313	3.49	1.25054	1.00558	0.487	25.1	0.213646	0.998648
Eye	Blepharitis eyelid infection (HES and self-reported)	T	379101	653	378448	0.000103	-9.18539	119.96	7.92E-107	1.33E+98	0.938965	0.998648
Mental health	Stress (HES and self-reported)	T	379101	662	378439	4.01	1.38982	1.00461	0.56	28.8	0.166528	0.998648
Mental health	Obsessive compulsive disorder ocd (HES and self-reported)	T	379101	208	378893	0.000112	-9.09806	198.055	2.89E-173	4.33E+164	0.96336	0.998648
Mental health	Insomnia (HES and self-reported)	T	379101	5225	373876	0.487	-0.718653	1.00414	0.0681	3.49	0.474182	0.998648
Musculoskeletal	Osteopenia (HES and self-reported)	T	379101	990	378111	8.11E-05	-9.42029	113.176	3.73E-101	1.76E+92	0.933664	0.998648
Musculoskeletal	Soft tissue inflammation (HES and self-reported)	T	379101	9317	369784	1.16	0.15037	0.507432	0.43	3.14	0.766973	0.998648
Musculoskeletal	Tendonitis tendinitis tenosynovitis (HES and self-reported)	T	379101	5613	373488	0.473	-0.749407	1.00364	0.0661	3.38	0.455253	0.998648
Musculoskeletal	Epicondylitis (HES and self-reported)	T	379101	745	378356	3.6	1.27989	1.00473	0.502	25.8	0.202711	0.998648
Musculoskeletal	Tennis elbow lateral epicondylitis (HES and self-reported)	T	379101	796	378305	3.35	1.20776	1.00457	0.467	24	0.22926	0.998648
Musculoskeletal	Housemaids knee prepatellar bursitis (HES and self-reported)	T	379101	300	378801	8.12E-05	-9.41845	194.804	1.23E-170	5.37E+161	0.961439	0.998648
Musculoskeletal	Fracture skull head (HES and self-reported)	T	379101	2136	376965	8.42E-05	-9.38224	72.6438	1.23E-66	5.77E+57	0.897236	0.998648
Musculoskeletal	Fracture jaw (HES and self-reported)	T	379101	790	378311	8.46E-05	-9.37793	118.276	1.77E-105	4.03E+96	0.936803	0.998648
Musculoskeletal	Fracture nose (HES and self-reported)	T	379101	1043	378058	6.12E-05	-9.70114	119.533	1.09E-106	3.43E+97	0.935316	0.998648
Musculoskeletal	Fracture face orbit eye socket (HES and self-reported)	T	379101	477	378624	4.96E-05	-9.91065	195.605	1.56E-171	1.58E+162	0.959591	0.998648
Musculoskeletal	Fracture neck cervical fracture (HES and self-reported)	T	379101	345	378756	6.74E-05	-9.60487	197.684	3.60E-173	1.26E+164	0.961248	0.998648
Musculoskeletal	Fracture clavicle collar bone (HES and self-reported)	T	379101	1092	378009	2.24	0.808323	1.00514	0.313	16.1	0.421287	0.998648
Musculoskeletal	Fracture shoulder scapula (HES and self-reported)	T	379101	2722	376379	1.96	0.674493	0.71267	0.486	7.94	0.343929	0.998648
Musculoskeletal	Fracture upper arm humerus elbow (HES and self-reported)	T	379101	3361	375740	1.57	0.451259	0.712555	0.389	6.35	0.52654	0.998648
Musculoskeletal	Fracture forearm wrist (HES and self-reported)	T	379101	8702	370399	0.932	-0.070292	0.583756	0.297	2.93	0.904156	0.998648
Musculoskeletal	Fracture radius (HES and self-reported)	T	379101	5082	374019	0.551	-0.596001	1.00439	0.0769	3.95	0.552914	0.998648
Musculoskeletal	Fracture ulna (HES and self-reported)	T	379101	1643	377458	4.10E-05	-10.1024	120.818	5.89E-108	2.85E+98	0.933361	0.998648
Musculoskeletal	Fracture wrist colles fracture (HES and self-reported)	T	379101	3198	375903	1.6	0.470977	0.712721	0.396	6.47	0.508731	0.998648
Musculoskeletal	Fracture hand (HES and self-reported)	T	379101	1148	377953	5.65E-05	-9.7804	120.434	1.73E-107	1.85E+98	0.935275	0.998648
Musculoskeletal	Fracture finger (HES and self-reported)	T	379101	1578	377523	1.58	0.457334	1.00456	0.221	11.3	0.648922	0.998648
Musculoskeletal	Fracture thumb (HES and self-reported)	T	379101	379	378722	6.12E-05	-9.70113	196.495	3.36E-172	1.11E+163	0.960624	0.998648
Musculoskeletal	Fracture rib (HES and self-reported)	T	379101	2085	377016	8.41E-05	-9.38311	72.5759	1.40E-66	5.04E+57	0.897131	0.998648
Musculoskeletal	Fracture sternum (HES and self-reported)	T	379101	240	378861	0.000106	-9.15107	197.994	3.09E-173	3.64E+164	0.963136	0.998648
Musculoskeletal	Fracture vertebra crush fracture vertebral col (HES and self-reported)	T	379101	1036	378065	2.53	0.928255	1.00437	0.353	18.1	0.355373	0.998648
Musculoskeletal	Fracture pelvis (HES and self-reported)	T	379101	555	378546	4.40E-05	-10.0322	198.902	2.16E-174	8.95E+164	0.959773	0.998648
Musculoskeletal	Fracture neck of femur hip (HES and self-reported)	T	379101	2221	376880	8.56E-05	-9.36592	72.1583	3.24E-66	2.26E+57	0.896727	0.998648
Musculoskeletal	Fracture shaft of femur (HES and self-reported)	T	379101	596	378505	0.000109	-9.12694	120.588	2.45E-107	4.82E+98	0.939668	0.998648
Musculoskeletal	Fracture patella knee (HES and self-reported)	T	379101	515	378586	4.78E-05	-9.94823	199.467	7.76E-175	2.95E+165	0.960223	0.998648
Musculoskeletal	Fracture lower leg ankle (HES and self-reported)	T	379101	6609	372492	1.2	0.185253	0.583719	0.383	3.78	0.750965	0.998648
Musculoskeletal	Fracture tibia (HES and self-reported)	T	379101	1942	377159	8.94E-05	-9.32255	73.3672	3.16E-67	2.53E+58	0.898887	0.998648
Musculoskeletal	Fracture fibula (HES and self-reported)	T	379101	749	378352	8.46E-05	-9.37728	120.718	1.48E-107	4.84E+98	0.938083	0.998648
Musculoskeletal	Fracture foot (HES and self-reported)	T	379101	1193	377908	5.43E-05	-9.82012	120.894	6.73E-108	4.39E+98	0.93526	0.998648
Musculoskeletal	Fracture metatarsal (HES and self-reported)	T	379101	436	378665	5.42E-05	-9.8221	199.744	5.11E-175	5.75E+165	0.960781	0.998648
Musculoskeletal	Fracture toe (HES and self-reported)	T	379101	413	378688	5.63E-05	-9.78531	198.798	3.39E-174	9.34E+164	0.960742	0.998648
Infectious disease	Septicaemia sepsis (HES and self-reported)	T	379101	3840	375261	2.1	0.740165	0.58504	0.666	6.6	0.205816	0.998648
Neurosciences	Meningioma benign meningeal tumour (HES and self-reported)	T	379101	499	378602	5.20E-05	-9.86412	196.305	4.15E-172	6.52E+162	0.959924	0.998648
Immuno-inflammation	Rosacea (HES and self-reported)	T	379101	850	378251	7.82E-05	-9.45562	121.09	6.60E-108	9.28E+98	0.937758	0.998648
Gynaecology and Obstetrics	Cervical erosion (HES and self-reported)	T	379101	898	378203	9.20E-08	-16.2016	3570.46	0	Inf	0.996379	0.998648
Gynaecology and Obstetrics	Abnormal smear cervix (HES and self-reported)	T	379101	1903	377198	4.29E-08	-16.9651	3613.5	0	Inf	0.996254	0.998648
Gynaecology and Obstetrics	Dysmenorrhoea dysmenorrhea (HES and self-reported)	T	379101	1606	377495	2.03	0.706037	1.01148	0.279	14.7	0.485164	0.998648
Gynaecology and Obstetrics	Menopausal symptoms menopause (HES and self-reported)	T	379101	9705	369396	0.587	-0.533491	0.719443	0.143	2.4	0.45837	0.998648
Gynaecology and Obstetrics	Benign breast lump (HES and self-reported)	T	379101	2336	376765	3.56E-05	-10.2422	111.505	4.33E-100	2.93E+90	0.926814	0.998648
Immuno-inflammation	Allergy to house dust mite (HES and self-reported)	T	379101	279	378822	8.32E-05	-9.394	199.284	1.93E-174	3.58E+165	0.962403	0.998648
Infectious disease	Scarlet fever scarlatina (HES and self-reported)	T	379101	435	378666	5.71E-05	-9.77072	196.494	3.14E-172	1.04E+163	0.960341	0.998648
Genitourinary	Undescended testicle (HES and self-reported)	T	379101	210	378891	4.20E-08	-16.9862	9881.82	0	Inf	0.998628	0.998648
Cancer	Bowens disease (HES and self-reported)	T	379101	617	378484	4.28E-05	-10.0585	193.202	1.50E-169	1.23E+160	0.958479	0.998648
Neurosciences	Benign neuroma (HES and self-reported)	T	379101	590	378511	0.000111	-9.10928	120.955	1.22E-107	1.01E+99	0.939967	0.998648
Musculoskeletal	Fracture head neck (HES and self-reported)	T	379101	2134	376967	8.46E-05	-9.37751	72.7102	1.08E-66	6.60E+57	0.897381	0.998648
Musculoskeletal	Fracture upper limb shoulder (HES and self-reported)	T	379101	10515	368586	1.04	0.03713	0.50748	0.384	2.81	0.941675	0.998648
Musculoskeletal	Other fractures (HES and self-reported)	T	379101	5304	373797	1.5	0.407883	0.584211	0.478	4.73	0.485066	0.998648
Musculoskeletal	Fracture pelvis lower limb (HES and self-reported)	T	379101	8374	370727	0.963	-0.037584	0.583902	0.307	3.02	0.948679	0.998648
Musculoskeletal	OA under age 50	T	327093	14899	312194	0.727	-0.319161	0.508436	0.268	1.97	0.53018	0.998648
Musculoskeletal	Osteoarthritis all	T	370717	58523	312194	0.825	-0.19291	0.257174	0.498	1.36	0.453185	0.998648
Musculoskeletal	Osteoarthritis hip	T	322974	10780	312194	1.22	0.201361	0.461562	0.495	3.02	0.662648	0.998648

Musculoskeletal	Osteoarthritis knee	T	330411	18217	312194	0.716	-0.333397	0.458885	0.291	1.76	0.46751	0.998648
Musculoskeletal	Osteoarthritis knee or hip	T	339616	27422	312194	0.958	-0.043051	0.333117	0.499	1.84	0.897171	0.998648
Musculoskeletal	Osteoarthritis non load bearing joints	T	317066	4872	312194	0.551	-0.595158	1.00542	0.0769	3.96	0.553886	0.998648
Musculoskeletal	BIN combinedFractures	T	379101	28020	351081	1.14	0.131443	0.301862	0.631	2.06	0.663244	0.998648
Cardiovascular	Pulse minimumValue	T	357246	NA	NA	NA	0.039048	0.085729	-0.128981	0.207077	0.64876	0.998648
Other	Sleep duration	T	376797	NA	NA	NA	0.016664	0.083626	-0.147244	0.180572	0.842051	0.998648
Other	Sleeplessness insomnia	T	378601	NA	NA	NA	0.088665	0.083279	-0.074561	0.251891	0.287021	0.998648
Other	Daytime dozing sleeping narcolepsy	T	377552	NA	NA	NA	-0.044199	0.083158	-0.207189	0.118792	0.595072	0.998648
Cardiovascular	Ventricular rate	T	8941	NA	NA	NA	0.600039	0.999205	-1.3584	2.55848	0.548162	0.998648
Cardiovascular	P duration	T	8939	NA	NA	NA	0.34455	0.99973	-1.61492	2.30402	0.730363	0.998648
Cardiovascular	QRS duration	T	8939	NA	NA	NA	-0.169224	0.999064	-2.12739	1.78894	0.865495	0.998648
Cardiovascular	Cardiac index during PWA	T	8000	NA	NA	NA	0.455614	0.999219	-1.50286	2.41408	0.648411	0.998648
Summary	Number of self reported non cancer illnesses	T	379040	NA	NA	NA	0.099992	0.083256	-0.063191	0.263174	0.229747	0.998648
Summary	Number of operations self reported	T	379040	NA	NA	NA	-0.029899	0.083296	-0.193159	0.133362	0.71964	0.998648
Summary	Number of treatments medications taken	T	379040	NA	NA	NA	0.1075	0.083261	-0.055692	0.270692	0.196662	0.998648
Family history	Father s age at death	T	278550	NA	NA	NA	0.082629	0.096508	-0.106527	0.271784	0.391894	0.998648
Other	Townsend deprivation index at recruitment	T	378649	NA	NA	NA	-0.085601	0.082809	-0.247907	0.076705	0.301271	0.998648
Neurosciences	Fluid intelligence score	T	184529	NA	NA	NA	0.06482	0.112999	-0.156658	0.286298	0.566218	0.998648
Neurosciences	Prospective memory result	T	126279	NA	NA	NA	-0.075415	0.124417	-0.319272	0.168443	0.544417	0.998648
ENT	Speech reception threshold SRT estimate left	T	114702	NA	NA	NA	0.063039	0.130992	-0.193706	0.319783	0.630345	0.998648
ENT	Speech reception threshold SRT estimate right	T	114815	NA	NA	NA	0.08644	0.130951	-0.170224	0.343104	0.509194	0.998648
Neurosciences	Mean time to correctly identify matches	T	376457	NA	NA	NA	0.037894	0.083199	-0.125176	0.200963	0.648776	0.998648
Family history	Alzheimer s disease dementia (family history)	T	379101	NA	NA	NA	-0.012237	0.08306	-0.175034	0.150561	0.882877	0.998648
Family history	Parkinson s disease (family history)	T	379101	NA	NA	NA	0.002697	0.083009	-0.160001	0.165395	0.974083	0.998648
Family history	Severe depression (family history)	T	379101	NA	NA	NA	0.036018	0.083117	-0.126891	0.198926	0.664769	0.998648
Family history	Prostate cancer (family history)	T	174719	NA	NA	NA	-0.049833	0.116354	-0.277887	0.178221	0.668441	0.998648
Family history	Heart disease (family history)	T	379101	NA	NA	NA	-0.112069	0.083269	-0.275276	0.051138	0.178346	0.998648
Family history	Stroke (family history)	T	379101	NA	NA	NA	0.075186	0.083158	-0.087804	0.238176	0.365925	0.998648
Family history	Lung cancer (family history)	T	379101	NA	NA	NA	0.044111	0.083151	-0.118865	0.207088	0.59577	0.998648
Family history	Bowel cancer (family history)	T	379101	NA	NA	NA	0.071277	0.082994	-0.091391	0.233944	0.390438	0.998648
Family history	Diabetes (family history)	T	379101	NA	NA	NA	0.004499	0.083122	-0.15842	0.167417	0.95684	0.998648
Mental health	Neuroticism score	T	307275	NA	NA	NA	-0.115042	0.090906	-0.293218	0.063134	0.205689	0.998648
Neurosciences	Number of incorrect matches in round	T	93587	NA	NA	NA	-0.028184	0.157776	-0.337424	0.281057	0.858228	0.998648
Anthropometry	Weight	T	377979	NA	NA	NA	-0.006043	0.083308	-0.169327	0.157241	0.942175	0.998648
Cardiovascular	Pulse wave Arterial Stiffness index	T	124962	NA	NA	NA	0.106462	0.128009	-0.144436	0.35736	0.405593	0.998648
Musculoskeletal	Falls in the last year	T	378169	NA	NA	NA	-0.02979	0.083366	-0.193186	0.133606	0.720836	0.998648
Anthropometry	Body fat percentage	T	372255	NA	NA	NA	0.101278	0.084023	-0.063408	0.265963	0.228067	0.998648
Anthropometry	Whole body fat mass	T	371851	NA	NA	NA	0.054896	0.084159	-0.110056	0.219849	0.514214	0.998648
Anthropometry	Whole body fat free mass	T	372439	NA	NA	NA	-0.067157	0.083966	-0.23173	0.097416	0.423818	0.998648
Anthropometry	Whole body water mass	T	372469	NA	NA	NA	-0.066664	0.083968	-0.231242	0.097914	0.427243	0.998648
Other	Relative age of first facial hair	T	168858	NA	NA	NA	-0.025601	0.117591	-0.256079	0.204877	0.827653	0.998648
Other	Relative age voice broke	T	161623	NA	NA	NA	-0.152158	0.120248	-0.387844	0.083528	0.20574	0.998648
Other	Age when periods started menarche	T	198489	NA	NA	NA	0.154358	0.121206	-0.083206	0.391922	0.202834	0.998648
Biological assays	Mean corpuscular volume	T	367861	NA	NA	NA	0.116624	0.084018	-0.048051	0.281298	0.165111	0.998648
Biological assays	Mean corpuscular haemoglobin concentration	T	367856	NA	NA	NA	0.032247	0.084169	-0.132724	0.197218	0.70163	0.998648
Biological assays	Red blood cell erythrocyte distribution width	T	367861	NA	NA	NA	0.039736	0.084211	-0.125318	0.20479	0.637027	0.998648
Biological assays	Mean platelet thrombocyte volume	T	367857	NA	NA	NA	0.081997	0.084223	-0.083079	0.247073	0.330269	0.998648
Biological assays	Platelet distribution width	T	367857	NA	NA	NA	0.039537	0.084213	-0.125521	0.204595	0.63872	0.998648
Biological assays	Monocyte count	T	367207	NA	NA	NA	0.107667	0.0845	-0.057953	0.273287	0.202605	0.998648
Biological assays	Neutrophill count	T	367207	NA	NA	NA	-0.103445	0.0845	-0.269066	0.062176	0.220879	0.998648
Biological assays	Eosinophill count	T	367207	NA	NA	NA	-0.042054	0.084486	-0.207646	0.123538	0.618649	0.998648
Biological assays	Basophill count	T	367207	NA	NA	NA	-0.053479	0.084186	-0.218484	0.111527	0.525271	0.998648
Biological assays	Nucleated red blood cell count	T	367198	NA	NA	NA	0.108471	0.084246	-0.056651	0.273593	0.197902	0.998648
Biological assays	Lymphocyte percentage	T	367213	NA	NA	NA	-0.10878	0.084522	-0.274443	0.056883	0.198094	0.998648
Biological assays	Neutrophill percentage	T	367213	NA	NA	NA	0.029618	0.084519	-0.136039	0.195276	0.726013	0.998648
Biological assays	Eosinophill percentage	T	367213	NA	NA	NA	0.020165	0.084508	-0.145471	0.1858	0.811405	0.998648
Biological assays	Basophill percentage	T	367213	NA	NA	NA	-0.110111	0.084511	-0.275752	0.05553	0.192604	0.998648
Biological assays	Nucleated red blood cell percentage	T	367195	NA	NA	NA	0.111997	0.084246	-0.053125	0.277119	0.183711	0.998648
Biological assays	Creatinine enzymatic in urine	T	368234	NA	NA	NA	-0.094377	0.08508	-0.261134	0.072379	0.267309	0.998648
Biological assays	Potassium in urine	T	367438	NA	NA	NA	-0.116307	0.085105	-0.283113	0.050499	0.171743	0.998648
Biological assays	Sodium in urine	T	367460	NA	NA	NA	-0.068134	0.085005	-0.234743	0.098476	0.422828	0.998648
Respiratory	FVC maximumValue	T	286167	NA	NA	NA	-0.117711	0.09821	-0.310203	0.074781	0.230697	0.998648
Respiratory	FVC maximumValue strict	T	221665	NA	NA	NA	-0.100342	0.110732	-0.317377	0.116693	0.364846	0.998648
Family history	Mother s age at death	T	223081	NA	NA	NA	-0.030213	0.104161	-0.234369	0.173942	0.771767	0.998648
Gynaecology and Obstetrics	Age started hormone replacement therapy HRT	T	71387	NA	NA	NA	-0.02596	0.20843	-0.434483	0.382563	0.90088	0.998648
Gynaecology and Obstetrics	Age at menopause last menstrual period	T	116962	NA	NA	NA	0.159462	0.154064	-0.142503	0.461427	0.30065	0.998648
Neurosciences	Incorect matches summed	T	379101	NA	NA	NA	-0.051901	0.083239	-0.215049	0.111247	0.532942	0.998648
Cancer	Reported occurrences of cancer	T	61110	NA	NA	NA	0.131772	0.213688	-0.287056	0.5506	0.53746	0.998648
Cardiovascular	Systolic blood pressure mean	T	357243	NA	NA	NA	-0.028394	0.085724	-0.196412	0.139624	0.740474	0.998648
Neurosciences	Maximum digits remembered correctly	T	116653	NA	NA	NA	-0.024217	0.136049	-0.290873	0.242439	0.858722	0.998648
Mental health	Happiness	T	126053	NA	NA	NA	0.069017	0.125023	-0.176028	0.314062	0.580926	0.998648
Anthropometry	Waist circumference	T	378432	NA	NA	NA	0.002993	0.083315	-0.160304	0.16629	0.971341	0.998648
Anthropometry	Hip circumference	T	378389	NA	NA	NA	0.06803	0.083305	-0.095248	0.231308	0.414135	0.998648
Eye	LogMAR final left	T	83967	NA	NA	NA	-0.198471	0.168868	-0.529452	0.13251	0.239873	0.998648
Eye	Corneal hysteresis right	T	81176	NA	NA	NA	0.019504	0.168939	-0.311617	0.350624	0.908091	0.998648
Eye	Corneal resistance factor right	T	81176	NA	NA	NA	-0.130219	0.168999	-0.461457	0.201019	0.440983	0.998648
Eye	Intra ocular pressure corneal compensated left	T	81056	NA	NA	NA	-0.225632	0.169022	-0.556915	0.105651	0.181899	0.998648
Eye	Corneal hysteresis left	T	81056	NA	NA	NA	-0.028389	0.169003	-0.359634	0.302857	0.866601	0.998648
Eye	Corneal resistance factor left	T	81056	NA	NA	NA	-0.167155	0.169039	-0.498471	0.164161	0.322736	0.998648
Other	Overall acceleration average	T	81471	NA	NA	NA	-0.031229	0.164351	-0.353357	0.290899	0.849297	0.998648
Respiratory	FEV1 never smoked only	T	109851	NA	NA	NA	-0.173301	0.147134	-0.461684	0.115082	0.238859	0.998648
ENT	Hear loss B	T	121038	NA	NA	NA	0.079169	0.127626	-0.170978	0.329316	0.535047	0.998648
Other	NumCigarettes	T	111875	NA	NA	NA	-0.159316	0.195939	-0.543356	0.224724	0.416165	0.998648
Biological assays	Urine albumin creatinine ratio	T	114295	NA	NA	NA	-0.058277	0.138632	-0.329996	0.213442	0.674213	0.998648

Ordered by FDR. The category "Summary" indicates cases were anyone with admission for any code in the block. * Lung function measures described as "strict" only include individuals with passed-QC spirometry (see reference 16 in the main
