# Supplementary material for: Pleiotropic associations of heterozygosity for the SERPINA1 Z allele in the UK Biobank
Source: ERJ Open Res. 2021 May 10;7(2):00049-2021. doi: 10.1183/23120541.00049-2021 (PMC8107350; doi:10.1183/23120541.00049-2021)
Supplement: Supplementary file 5 [file 00049-2021.TableS4.pdf]

Table S4. Interactions between the *SERPINA1* Z allele and smoking status or sex in UK Biobank participants

Interaction	Genetic model	FEV_1 (ml)			FEV_1/FVC			FVC (ml)			COPD		
		beta	se	p	beta	se	p	beta	se	p	OR	95% CI	p
Z*EVERSMK	Heterozygous	20.782	9.107	**0.022**	0.0044	0.0011	1.06×10^{-4}	6.587	10.899	0.546	1.31	1.10-1.58	0.003
Z*EVERSMK	Recessive	192.066	103.651	0.064	0.045	0.013	5.06×10^{-4}	60.088	124.033	0.628	1.46	0.51-4.28	0.484
Z*sex	Heterozygous	6.821	9.13	0.455	0.0042	0.0011	0.00021	-12.351	10.927	0.258	0.88	0.74-1.05	0.153
Z*sex	Recessive	57.606	100.578	0.567	0.026	0.013	0.041	-18.799	120.356	0.876	1.18	0.41-3.34	0.755

Results are based on linear (or logistic, in the case of COPD) regression adjusting for sex, age, age^2, height, ever-smoking, ancestry-based principal components, and genotyping array. The heterozygous or recessive genetic model was also included in the regression as shown. Males were coded as 1 and females as 2, so a positive interaction coefficient means there was a more positive coefficient in females compared to males and a negative interaction coefficient means there was a less positive coefficient in females compared to males. Ever-smoking status was coded 1 if the individual had ever-smoked and 2 if the individual had never smoked.
