# Supplementary material for: Pleiotropic associations of heterozygosity for the SERPINA1 Z allele in the UK Biobank
Source: ERJ Open Res. 2021 May 10;7(2):00049-2021. doi: 10.1183/23120541.00049-2021 (PMC8107350; doi:10.1183/23120541.00049-2021)
Supplement: Supplementary file 6 [file 00049-2021.TableS5.pdf]

Table S5. Association of heterozygosity for the *SERPINA1* Z allele with height and lung function in individuals aged 8, 15, and 24

Age (years)	Height (cm)				FEV_1/FVC[a]			
	N	beta	se	p	N	beta	se	p
**8**	5,162	0.064	0.385	0.869	4,838	0.023	0.071	0.744
**15**	4,023	0.452	0.512	0.378	2,930	0.016	0.086	0.848
**24**	2,766	0.493	0.558	0.376	2,131	0.078	0.096	0.415

Data derived from study participants in the ALSPAC study. [a]Age, sex, and height adjusted Z scores
