# Supplementary material for: Pleiotropic associations of heterozygosity for the SERPINA1 Z allele in the UK Biobank
Source: ERJ Open Res. 2021 May 10;7(2):00049-2021. doi: 10.1183/23120541.00049-2021 (PMC8107350; doi:10.1183/23120541.00049-2021)
Supplement: Supplementary file 7 [file 00049-2021.TableS6.pdf]

Table S6. Association between the *SERPINA1* Z allele and lung function traits in UK Biobank male and female never- and ever-smokers

Genetic model	Subgroup	FEV_1 (ml)			FEV_1/FVC			FVC (ml)			COPD	
		beta	se	p	beta	se	p	beta	se	p	OR (95% CI)	p
Heterozygous	Male never-smokers	17.013	11.346	0.134	0.00087	0.0012	0.478	15.941	13.751	0.246	1.04 (0.84-1.28)	0.689
	Female never-smokers	20.493	6.251	0.001	0.0045	0.00086	$1.75x10^{-7}$	10.265	7.72	0.184	0.73 (0.59-0.91)	0.005
	Male ever-smokers	-13.986	11.327	0.217	-0.0036	0.0013	$7.55x10^{-3}$	1.672	13.204	0.899	1.16 (1.01-1.33)	0.028
	Female ever-smokers	9.974	8.142	0.221	0.00027	0.0012	0.821	9.896	9.682	0.307	1.16 (0.98-1.36)	0.081
Recessive	Male never-smokers	-184.651	111.011	0.096	-0.033	0.012	$6.81x10^{-3}$	-155.016	134.511	0.249	5.26 (1.46-14.78)	0.004
	Female never-smokers	-158.256	67.643	0.019	-0.034	0.0093	$2.20x10^{-4}$	-55.679	83.539	0.505	6.28 (2.07-15.50)	$2.65x10^{-4}$
	Male ever-smokers	-422.885	133.09	0.0015	-0.106	0.016	$1.84x10^{-11}$	-103.271	155.115	0.506	8.37 (3.01-21.51)	$1.64x10^{-5}$
	Female ever-smokers	-314.24	102.273	0.0021	-0.05	0.015	$9.14x10^{-4}$	-258.857	121.59	0.033	10.77 (2.95-31.82)	$5.60x10^{-5}$

Results are based on linear (or logistic, in the case of COPD) regression adjusting for age, age^2, height, ancestry-based principal components, and genotyping array. The heterozygous or recessive genetic model was also included in the regression as shown
